# Investigating the impact of trial retractions on the healthcare evidence ecosystem (VITALITY Study I): retrospective cohort study

**DOI:** 10.1136/bmj-2024-082068

**Published:** 2025-04-23

**Authors:** Chang Xu, Shiqi Fan, Yuan Tian, Fuchen Liu, Luis Furuya-Kanamori, Justin Clark, Chao Zhang, Sheng Li, Lifeng Lin, Haitao Chu, Sheyu Li, Su Golder, Yoon Loke, Sunita Vohra, Paul Glasziou, Suhail A Doi, Hui Liu, VITALITY Collaborative Research Network, Yuqing Yang, Hong Cao, Zhen Peng, Ruixuan Tang, Limin Li, Zhuanlan Sun, Jiahui Yin, Rui Zhang, Yi Zhu, Xi Yang, Lijun Tang, Mengya Zhao, Manyu Zhang, Ruilin Zhang, Keke XuLiu, Ying Tang, Huoran Zhou, Ruoxi Wang, Qiannan Wang, Limin Li, Tolgor Bau, Zhihui Jia, Kexin Gao, Yafang Hao, Lijie Zhang, Liying Zhang, Kexin Fu, Shuting Xing, Ming Cheng, Xiaojie Li, Kai Liu, Na He, Mingyang Ma, Debei Hao, Shuya Sun, Ruixian Tang, Qianqian Ji, Chenglin Hu, Jiazhen Wang, Keer Jiang, Weina Li, Yuxuan Guo, Guifang Jin, Feifei Yan, Lizi Hu, Man Zhao, Tingkai Zhang, Feiyang Ye, Rui Zhou, Gaowei Yang, Xiaoqing Wu, Jin Qian, Jie Sheng, Minghao Ruan, Qingwen Ni, Hongbo Wang, Xiaocheng Feng, Shuxun Wei, Zhichao Wei, Jingjing Xu, Junhao Fan, Liuyu Zhou, Siwen Li, Hucheng Liu, Yuefan Wang, Jin Du, Wuyu Chen, Wei Dong

**Affiliations:** 1 Proof of Concept Center, Eastern Hepatobiliary Surgery Hospital, Third Affiliated Hospital, Second Military Medical University, Naval Medical University, Shanghai, China; 2 The Third Department of Hepatic Surgery, Eastern Hepatobiliary Surgery Hospital, Third Affiliated Hospital, Second Military Medical University, Naval Medical University, Shanghai, China; 3 UQ Centre for Clinical Research, University of Queensland, Herston, Queensland, Australia; 4 Institute for Evidence-Based Healthcare, Faculty of Health Sciences and Medicine, Bond University, Gold Coast, Queensland, Australia; 5 Center of Evidence-based Medicine, Taihe Hospital, Hubei University of Medicine, Shiyan, China; 6 Office of Research Affairs, Zhongnan Hospital of Wuhan University, Wuhan, China;; 7 Department of Epidemiology and Biostatistics, University of Arizona, Tucson, AZ, USA; 8 Statistical Research and Data Science Center, Global Biometrics and Data Management, Pfizer Inc, New York, NY, USA; 9 Division of Biostatistics and Health Data Science, University of Minnesota School of Public Health, Minneapolis, Minnesota, MN, USA; 10 Department of Endocrinology and Metabolism, Division of Guideline and Rapid Recommendation, Cochrane China Centre, MAGIC China Centre, Chinese Evidence-Based Medicine Centre, West China Hospital, Sichuan University, Chengdu, China; 11 Department of Health Sciences, University of York, York, UK; 12 Norwich Medical School, University of East Anglia, Norwich, UK; 13 Department of Pediatrics, Faculty of Medicine & Dentistry; Department of Psychiatry, Faculty of Medicine and Dentistry, University of Alberta, Edmonton, AB, Canada; 14 Department of Population Medicine, College of Medicine, QU Health, Qatar University, Doha, Qatar

## Abstract

**Objective:**

To investigate the impact of retracted trials on the production and use of healthcare evidence in the evidence ecosystem.

**Design:**

Retrospective cohort study based on forward citation searching.

**Data sources:**

Retraction Watch up to 5 November 2024.

**Study selection:**

Randomised controlled trials in humans that were retracted for any reason.

**Methods:**

Forward citation searching via Google Scholar and Scopus was used to identify evidence synthesis research (21 November 2024) that quantitatively incorporated retracted trials. Data were independently extracted by two groups of researchers. The results of meta-analyses were updated after exclusion of the retracted trials. The proportions of meta-analyses that changed direction of the pooled effect and/or the significance of the P value were estimated. A generalised linear mixed model was used to investigate the association between the number of included studies and the impact, measured by odds ratio and 95% confidence interval (CI). The impact of distorted evidence on clinical practice guidelines was also investigated on the basis of citation searching.

**Results:**

The searches identified 1330 retracted trials and 847 systematic reviews that quantitatively synthesised retracted trials, with a total of 3902 meta-analyses that could be replicated. After the potential clustering effects were accounted for, the exclusion of the retracted trials led to a change in the direction of the pooled effect in 8.4% (95% CI 6.8% to 10.1%), in its statistical significance in 16.0% (14.2% to 17.9%), and in both direction and significance in 3.9% (2.5% to 5.2%) and a >50% change in the magnitude of the effect in 15.7% (13.5% to 17.9%). An obvious non-linear association existed between the number of included studies and the impact on the results, with a lower number of studies having higher impact (eg, for 10 studies versus ≥20 studies, change of direction: odds ratio 2.63, 95% CI 1.29 to 5.38; P<0.001). Evidence from 68 systematic reviews with conclusions distorted by retracted trials was used in 157 guideline documents.

**Conclusion:**

Retracted trials have a substantial impact on the evidence ecosystem, including evidence synthesis, clinical practice guidelines, and evidence based clinical practice. Evidence generators, synthesisers, and users must pay attention to this problem, and feasible approaches that assist with easier identification and correction of such potential contamination are needed.

**Study registration:**

Open Science Framework (https://osf.io/7eazq/).

## Introduction

In evidence based medicine, effective decision making relies on the establishment of a trustworthy evidence ecosystem.^1^ Evidence generation and evidence synthesis are key elements for the establishment of such an ecosystem, and they are key drivers to affect implementation of evidence based healthcare policy and practice.^2^ Clinicians and policy makers use evidence or synthesise evidence from existing research to create clinical practice guidelines or conduct health technology assessments, which further support healthcare practice. The degree to which evidence is valid and robust determines its value in promoting evidence based clinical guidelines and healthcare.

Randomised controlled trials serve as the main source of high quality data for evidence synthesis.^3^ ^4^ The design of randomised controlled trials enables the minimisation of potential biases arising from methodological weaknesses such as confounding by indication.^5^ On this basis, synthesised evidence from randomised controlled trials is considered the highest level in the evidence pyramid, and such evidence has been widely adopted to inform clinical practice guidelines and health technology assessments.^6^ For this to hold, randomised controlled trials must report information transparently and honestly, without bias or distortion. However, this is not always the case, as an increasing number of retracted clinical trials have been documented owing to the questionable nature of their data and practices.^7^ ^8^

The retraction of randomised controlled trials poses a severe threat to evidence based medicine, and when retracted trials are included as part of evidence synthesis, a question arises as to whether the evidence ecosystem is still trustworthy. The incorporation of “contaminated” evidence into clinical practice guidelines could lead to incorrect conclusions, mislead healthcare practice, increase the epistemic cost, and potentially cause harm to patients.^9^ ^10^ To this end, investigating the impact of retracted trials and quantifying the extent of the potential contamination cascade on clinical practice guidelines is an urgent priority in the science of evidence synthesis. In this study, we aimed to answer the above question via a large scale investigation based on forward citation searching.

## Methods

### Design and settings

This study was based on forward citation searching. To start with, we identified a cohort of retracted randomised controlled trials from the Retraction Watch database (see below). We then used forward citation searching to identify systematic reviews and meta-analyses that had included these retracted trials. To quantify the influence of the retracted trials on the results of the systematic reviews and meta-analyses, we reanalysed the pooled data after excluding the retracted trials. Finally, we used a comprehensive literature search to identify relevant clinical practice guidelines that might be affected by evidence from contaminated systematic reviews. The conduct and analysis of this study were pre-specified by a pre-defined protocol on 13 April 2023, which can be accessed in the supplementary file or online via the Open Science Framework website (https://osf.io/7eazq/).^11^

### Data sources and inclusion criteria

The database created for this study was constructed from three data sources: retracted trials, evidence synthesis research that included retracted trials, and clinical practice guidelines that used evidence from contaminated evidence synthesis research.

#### Retracted trials

We searched the Retraction Watch database for retracted publications up to 26 April 2023 under the agreements of the dataset owners and updated the search on 5 November 2024. We used two separate searches: retrieved records labelled as “Clinical Study” and retrieved records labelled as “Research Article” which limited the subjects of “(HSC) Health Sciences”. The search strategy was developed by two information specialists (SG and JC) after discussions with the lead author (CX), and the literature search was run by the lead author. Only articles with notification of “retraction” were considered. Two researchers (CX and YT) reviewed the titles and abstracts of the retracted publications via the Rayyan online application (https://www.rayyan.ai/) and then reviewed the full texts independently, identifying and including those featured as published randomised controlled trials in humans (not including preprints) and presented in English. We broadly selected studies for which the full text reported the design to be a randomised trial; we did not delve into the exact methods of sequence generation or allocation concealment. For secondary analyses that used data from already published randomised controlled trials, we considered only those in which the data were analysed as per the originally randomised groups. Any disagreements were resolved through discussion with another senior methodologist (LFK). The search strategy is presented in supplementary table S1.

#### Evidence synthesis research that included retracted trials

To identify evidence synthesis research that may have been affected by data from retracted trials, we used forward citation searching of each retracted trial via Google Scholar on 19 July 2023, with the last update from 21 November to 2 December 2024. In addition, we used forward citation searching via Scopus on 11 June 2024, updating it on 21 November 2024. All citations of retracted trials from Google Scholar and Scopus were reviewed by title and abstract, and all types of reviews (for example, narrative reviews, systematic reviews, rapid reviews, and umbrella reviews) were selected at this stage. The screening of the citations from Google Scholar was done by 13 well trained postgraduate or doctoral student team members (RZ, YZ, XY, SQF, YT, LJT, MYZ, LKKX, YT, RLZ, HRZ, RXW, and QNW). Screening of the citations from Scopus was done by using a filter of 21 keywords (supplementary file, page 14), according to a previous study.^12^ A test of the filter based on 2846 citations (limited to “article” and “review”) of the PREDIMED study^13^ showed that its sensitivity for detecting systematic reviews was 98.8% (supplementary table S2).

Further full text review of records obtained from Google Scholar in the first stage was done by two students (SQF and YT) from the same group to verify whether quantitative evidence synthesis was conducted in these reviews; systematic reviews (including preprints) with at least one pair-wise meta-analyses were identified for inclusion. Full text review of records obtained from Scopus was independently done by two senior methodologists (CX and CZ). To avoid duplication, we did not include scoping reviews, umbrella reviews, reanalysis of meta-analyses, or other types of reviews on reviews or meta-analyses. We did not consider conference abstracts as they did not contain sufficient information to allow for replication. We did not consider systematic reviews that had been retracted. We set restrictions by language for English only.

#### Clinical practice guidelines that used contaminated evidence

To identify potentially affected clinical practice guidelines, we collected the topics of systematic reviews and meta-analyses with conclusions that were substantially affected by retracted trials (see definition in the outcome section). The lead author (CX) searched Scopus and Google Scholar for titles of related systematic reviews up to 1 January 2025, with a manual check of the Guidelines International Network (GIN) (https://g-i-n.net/) and TRIP (https://www.tripdatabase.com/) databases based on the systematic review topics. We took the definition of clinical practice guidelines from Kataoka as “statements that include recommendations intended to optimize patient care. They are informed by systematic reviews of evidence and an assessment of the benefits and harms of alternative care options.”^14^ We did not make a distinction between evidence based and consensus based guidelines as suggested by Benjamin and colleagues.^15^ Instead, we considered any guideline documents (including position statements) that summarised existing evidence and formed clear recommendations to be clinical practice guidelines. We restricted clinical practice guidelines to those written in English and identified them by using the following keywords: “clinical practice guideline”, “clinical guideline”, “guideline”, “consensus”, “statements”, and “recommendations”. Initial screening of the guidelines by one researcher (CX) via the Rayyan application was based on title and abstract; this was followed by full text assessment by two researchers independently (CX and LFK), with any disagreements resolved through discussion with an expert in clinical practice guidelines (SYL).

### Data collection

We extracted the following data from the retracted trials: Digital Object Identifier (DOI) number, total number of citations, title, journal of publication, date of publication, date of retraction, reasons for retraction (according to Retraction Watch), number of authors, geographical region of the corresponding author, trial registration information, data sharing statement, and source of funding.

We extracted the following information from systematic reviews and meta-analyses that included retracted trials in their evidence synthesis: DOI number, title, year of publication, synthesis approaches (quantitative versus qualitative), type of meta-analysis (for example, pair-wise, network, incidence), metadata of each meta-analysis and subgroups, methods for the data synthesis (for example, fixed effect model), effect estimate type (for example, odds ratio), software used for the synthesis, location of the meta-analyses with retracted trials, number of trials within each meta-analysis, number of retracted trials within each meta-analysis, and type of outcomes (benefit, harm) based on the categorisation of the review authors.

All data extraction was done by two groups of researchers independently. For the metadata of each contaminated meta-analysis, we recruited student volunteers to extract data on the basis of pre-designed data extraction forms in Excel. All student volunteers were trained for two rounds, and only those who achieved an accuracy of 90% and above in both rounds of training participated in data extraction. After training, 60 student volunteers met this criterion and extracted the metadata independently. Any disagreements were checked and resolved by one author (SQF).

### Replication

Three evidence synthesis methodologists (CX, SQF, and YT) replicated the meta-analyses that incorporated retracted trials. This was done in two steps. We first used the same data from the original meta-analyses with the same methods and effect estimates to replicate the results. After obtaining the same results as the original meta-analysis, we excluded retracted trials and reanalysed the remaining data under the same settings (for example, same synthesis methods, effect estimates). Minor differences would occur during the replication owing to the rounding settings. We set a strict tolerable absolute difference as 0.01; we treated differences in the effect, the low boundary, and the upper boundary not exceeding 0.01 (or not exceeding 0.03 together) as fully replicated.

We replicated only pair-wise meta-analyses that compared the health outcomes of two interventions (including placebo or usual care) as aforementioned. In some cases, meta-analyses within a systematic review had identical effects and confidence intervals because the review authors used different settings of stratification for subgroups within the same group of datasets. To avoid double counting, we removed the duplicates and treated each subgroup analysis with retracted trials as a separate meta-analysis. For Cochrane reviews, different versions of the same review were common; we replicated only the latest version that incorporated retracted trials to avoid duplication and exaggeration of effects. We did not replicate network meta-analyses as very few of them provided sufficient data for replication.^16^ We also did not replicate meta-analyses of incidence as this type of meta-analysis uses data from only one arm of the trial, which therefore cannot be considered randomised trial data and does not meet our inclusion criteria.^17^

### Outcomes

Our primary outcomes were the impact of retracted trials on the magnitude and direction of the effect sizes, as well as the significance of the P value, based on replication of the metadata. Our secondary outcome was an assessment of evidence syntheses that were substantially affected by retracted trials, which could potentially contaminate clinical practice guidelines. We defined “substantially affected” as those that changed both the direction of the effect size and the significance of the P value or those that changed the direction of the effect size while the P value remained significant, which would largely change the implied conclusion of such meta-analyses.

### Statistical analysis

We summarised the baseline characteristics of retracted trials as frequency and proportion for binary or categorical data or as median and interquartile range for continuous data. For the main outcomes, we summarised the following metrics of the impact of retracted trials on the meta-analyses results: the proportion of meta-analyses that changed the direction of the effect size (for example, shifted from odds ratio >1 to odds ratio <1 or vice versa) after exclusion of the retracted trials; the proportion of meta-analyses that changed the significance of the P value (that is, shifted from <0.05 to >0.05 or vice versa) after exclusion of the retracted trials; the proportion of meta-analyses that changed both the direction of the effect size and the significance of the P value after exclusion of the retracted trials; and the proportion of meta-analyses whose effect size magnitude changed by more than 50% after exclusion of the retracted trials.^16^ We calculated point estimates and confidence intervals for these proportions on the basis of a generalised linear mixed model with potential clustering of meta-analyses by retracted trial and systematic review accounted for by using random effects.^17^ The random effects are not nested, but instead crossed, meaning that the effect due to retracted trials is the same regardless of the systematic review of origin.^18^ We defined a special case for changes in direction and significance when all the studies in a meta-analysis were retracted. In such cases, the results shifted from providing evidence for the outcome to providing no evidence at all.

We also fitted the same generalised linear mixed model to investigate the association between the number of studies within a meta-analysis and the likelihood of changes in the effect size direction or magnitude or the P value significance.^19^ The regression removed the special case in which all studies in a meta-analysis were retracted. To account for the anticipated non-linear relation between the number of studies and the outcomes, we applied a restricted cubic spline with three knots, following Harrell's recommended percentiles.^20^ ^21^ As the proportion of retracted trials within a meta-analysis probably influences the likelihood of changes, we did stratified regression analyses by categorising meta-analyses into another four groups on the basis of the proportion of retracted trials: <25%, 25% to <50%, 50% to <75%, and 75% to <100%. We used the odds ratio with 95% confidence interval as the effect measure because it is not variation dependent and is thus portable across baseline risk.^22^

We used subgroup analyses to explore the impact of retracted trials under different settings, stratified by outcome data type (binary versus continuous) and nature (benefits versus harms).We used using the Stata SE/16 program for all analyses, with a two sided P value of 0.05 as the threshold for rejection of the null hypothesis of no effect. Visualisations were created using Excel 2016. The complete analysis codes are available in the supplementary file.

### Patient and public involvement

This study investigated a methodological question related to the evidence ecosystem. For this reason, no patients or members of the public were involved in designing the research question or the outcome measures, and nor were they involved in design, implementation, interpretation, or writing up of the results.

## Results

**Fig 1:**
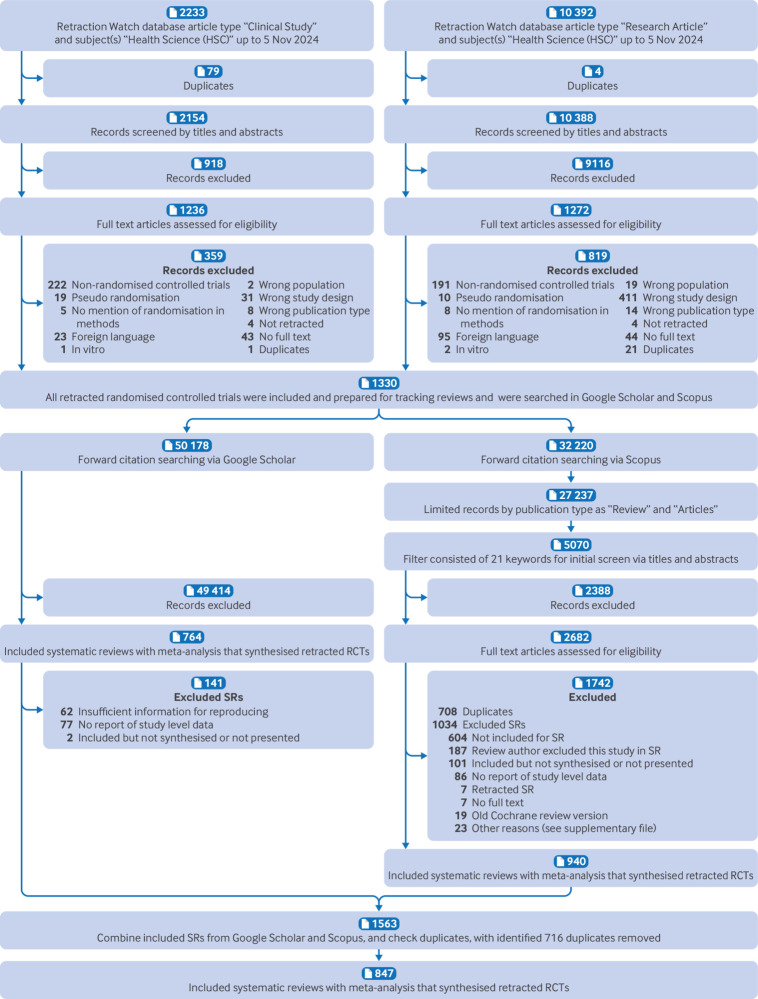
Flowchart of selection of retracted trials. RCT=randomised controlled trial; SR=systematic review

Our literature search on the Retraction Watch database resulted in 12 542 records. After reviewing the titles and abstracts, we excluded 10 034 of these; full text review of 2508 publications identified 1330 retracted trials that met the inclusion criteria (supplementary file, pages 39-114). Figure 1 shows the trial screening process. Supplementary table S3 shows the list of excluded publications with reasons for exclusion.

In terms of forward citation searching, the 1330 retracted trials had a total of 82 398 citations from Google Scholar and Scopus. We further identified 847 systematic reviews with meta-analyses that incorporated retracted trials into synthesised evidence (see supplementary tables S4 and S5 for exclusion and inclusion lists). The agreement rate of the metadata extraction between the two volunteer groups was 95.6%.

### Retracted randomised trials

Table 1 shows the baseline characteristics of the retracted trials; their median time to retraction was 21.3 (interquartile range 12.1-89.8) months from trial publication. Overall, 249 (18.7%) trials were registered and 34 (2.6%) were funded by industry. With regards to setting, 36.4% (n=484) of the retracted trials were single centre trials, 11.0% (n=146) were multicentre trials, and 52.6% (n=700) did not report such information. Only 27.8% (n=370) of the retracted trials had an explicit statement about data availability in the data sharing statements. The number of retracted trials increased rapidly in 2011 and 2023 (supplementary figure S1). Most of the retractions were initiated by journals (85.0%; n=1131), and only 9.2% (n=123) were initiated by the trial authors. The reported reasons for retraction involved a data related problem in 70.8% (n=942; see definition in table 1), and 12.9% (n=172) were marked as being from paper mills.

**Table 1:** Baseline characteristics of retracted trials. Values are numbers (percentages)

Characteristics	Summary of retracted trials	
	2010 and before (n=427)	2011 and after (n=903)
**Retraction procedure**		
Author initiated*	39 (9.1)	84 (9.3)
Journal initiated	366 (85.7)	765 (84.7)
Author and journal initiated	7 (1.6)	19 (2.1)
Unclear	15 (3.5)	35 (3.9)
**Reasons for retraction** **†**		
Data related retractions	284 (66.5)	658 (72.9)
Retractions for other reasons	143 (33.5)	245 (27.1)
**Centre information**		
Single centre	127 (29.7)	357 (39.5)
Multiple centres	38 (8.9)	108 (12.0)
Missing	262 (61.4)	438 (48.5)
**Trial registration**		
Yes	17 (4.0)	232 (25.7)
No	410 (96.0)	671 (74.3)
**Source of funding**		
Industry supported	10 (2.3)	24 (2.7)
Non-industry supported	45 (10.5)	267 (29.6)
Not funded	18 (4.2)	88 (9.7)
Not reported	354 (82.9)	524 (58.0)
**Raw data sharing statement**		
Data shared	0 (0.0)	320 (35.4)
Data not provided	0 (0.0)	50 (5.5)
Not reported	427 (100.0)	533 (59.0)
**From paper mills**		
Yes	0 (0.0)	172 (19.0)
No	427 (100.0)	731 (81.0)

Retracted trials increased rapidly from 2011; characteristics are summarised separately cut by year of 2010 (also year when Retraction Watch was founded).

* Refers to entities involved in retraction procedure including author of publication.

† Based on records from Retraction Watch; data related retractions are defined as those trials labelled with data problem, with or without other problems.

### Impact of retracted trials on evidence synthesis

#### Overall analyses

Of the 847 evidence synthesis publications that we identified, 324 (38.3%) were published after the earliest retraction within the review, and 4095 pair-wise meta-analyses that synthesised evidence from retracted trials were eligible for replication. With regards to the type of outcomes, 2372 (57.9%) were binary outcomes, and 1723 (42.1%) were continuous outcomes; 3689 (90.1%) investigated the benefits and 406 (9.9%) the harms of interventions. The number of retracted trials per meta-analysis ranged from one to 37, with a median number of 1 (interquartile range 1-1), and a minority of the meta-analyses (14.7%; 600/4095) had two or more retracted trials. The proportion of retracted trials per meta-analysis ranged from 0.7% to 100%, with a median proportion of 16.7% (interquartile range 9.1-33.3%); all included studies were retracted in 5.0% (205/4095) of the meta-analyses. We found that one fifth (687; 16.8%) of the meta-analyses potentially affected by retracted trials were from Cochrane reviews. We were able to fully replicate the original findings of 95.3% (3902/4095) meta-analyses from the reported data. However, despite our rigorous efforts, 193 (4.7%) could not be identically reconstructed to yield pooled estimates that exactly matched the original version. This was often because of incomplete and inconsistent reporting of the raw input data and the exact parameters of the meta-analytic model. The final analysis was based on the 3902 meta-analyses from 807 systematic reviews that we could fully replicate.

**Fig 2:**
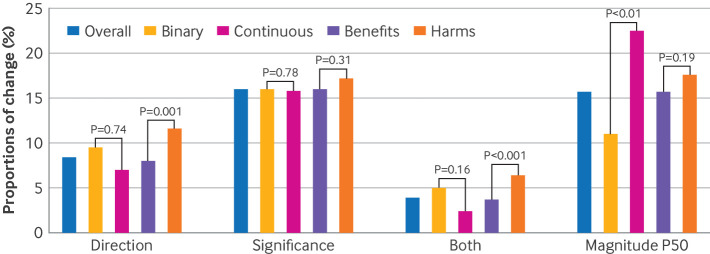
Impact of retracted trials on evidence synthesis research

Figure 2 shows the impact of retracted trials on the results of meta-analyses (n=3902). We found that the exclusion of the retracted trials from the meta-analyses led to a change in the direction of the pooled effect in 8.4% (95% confidence interval (CI) 6.8% to 10.1%; n=392) of the meta-analyses, and a change in the significance of the P value occurred in 16.0% (14.2% to 17.9%; n=692). The proportion of meta-analyses in which both the direction of the effects and the significance of the P value were altered was 3.9% (95% CI 2.5% to 5.2%; n=218), and the proportion of meta-analyses in which either the direction of the effects or the significance of the P value was altered was 20.6% (18.5% to 22.8%; n=866). In 15.7% (95% CI 13.5% to 17.9%; n=659) meta-analyses, the magnitude of the effects changed by more than 50% when retracted trials were excluded.

#### Subgroup analysis

When we stratified the outcomes by type of data, we found that 9.5% (95% CI 7.2% to 11.7%) of the meta-analyses had a change in the direction of effects for binary outcomes and 7.0% (5.1% to 8.9%) for continuous outcomes, after exclusion of retracted trials (P for interactio*n=*0.74). For the significance of the P value, 16.0% (95% CI 13.5% to 18.5%) changed in meta-analyses of binary outcomes and 15.8% (13.3% to 18.2%) changed in meta-analyses of continuous outcomes (P for interaction=0.78) after exclusion of retracted trials. For the magnitude of the effects, 11.0% (95% CI 8.4% to 13.5%) had a change by more than 50% for binary outcomes, and 22.5% (19.2% to 25.8%) had such a change for continuous outcomes (P for interaction<0.01) (fig 2).

When we stratified the outcomes by benefits and harms, we found that 8.0% (95% CI 6.4% to 9.7%) of the meta-analyses had a change in the direction of effect for benefit outcomes and 11.6% (7.0% to 16.2%) had a change for harms outcomes (P for interaction=0.001). For the significance of the P value, 16.0% (95% CI 14.1% to 18.0%) had a change in meta-analyses of benefit outcomes and 17.2% (12.2% to 22.2%) had a change for harms outcomes (P for interaction=0.31), after exclusion of retracted trials. For the magnitude of the effects, 15.7% (95% CI 13.5% to 18.0%) had a change by more than 50% for benefit outcomes and 17.6% (11.6% to 23.7%) for harms outcomes (P for interaction=0.19) (fig 2).

We did post hoc subgroup analysis by stratifying the meta-analyses on the basis of the reasons for retractions. For meta-analyses with retracted trials affected by data related problems (n=2885; 73.9%), 8.5% (95% CI 6.4% to 10.7%) had a change in the direction of the effects, 15.7% (13.5% to 17.8%) had a change in the significance of the P value, and 16.7% (14.0% to 19.5%) had a change in the magnitude of the effects by more than 50%. For meta-analyses with retracted trials without data problems (n=1017), 8.0% (95% CI 6.4% to 9.7%) had a change in the direction of the effects, 16.0% (14.1% to 18.0%) had a change in the significance of the P value, and 15.7% (13.5% to 18.0%) had a change in the magnitude of the effects by more than 50%. We observed no obvious between subgroup interactions (P for interaction=0.01, 0.30, and <0.001, respectively).

### Number of studies and extent of impact on results

We observed an obvious non-linear association between the number of studies and the extent of impact of the inclusion of retracted trials on the results. Meta-analyses with a lower number of studies had a higher impact of retracted trials, regardless of the outcome of interest (for example, for 10 studies versus ≥20 studies, change of direction: odds ratio 2.63, 95% CI 1.29 to 5.38; P<0.001). See supplementary figures S2-S7.

### Contaminated evidence in clinical practice guidelines

We investigated the extent of contamination in clinical practice guidelines via a comprehensive literature search of the titles and topics for those meta-analyses that were substantially impacted after exclusion retracted trials. We found 218 such meta-analyses from 68 systematic reviews,^23^ ^24^ ^25^ ^26^ ^27^ ^28^ ^29^ ^30^ ^31^ ^32^ ^33^ ^34^ ^35^ ^36^ ^37^ ^38^ ^39^ ^40^ ^41^ ^42^ ^43^ ^44^ ^45^ ^46^ ^47^ ^48^ ^49^ ^50^ ^51^ ^52^ ^53^ ^54^ ^55^ ^56^ ^57^ ^58^ ^59^ ^60^ ^61^ ^62^ ^63^ ^64^ ^65^ ^66^ ^67^ ^68^ ^69^ ^70^ ^71^ ^72^ ^73^ ^74^ ^75^ ^76^ ^77^ ^78^ ^79^ ^80^ ^81^ ^82^ ^83^ ^84^ ^85^ ^86^ ^87^ ^88^ ^89^ ^90^ consisting of 19 (28%) Cochrane reviews and 49 (72%) non-Cochrane reviews.

The database search yielded 17 731 records, with 157 clinical practice guidelines identified that summarised evidence from these contaminated reviews (supplementary tables S6 and S7; supplementary figure S8). When stratified by categories, 89 (57%) were clinical practice guidelines, 42 (27%) were consensus statements, 12 (8%) were position statements, nine (6%) were practice bulletins, and five (3%) were committee opinions. The year of the release of these guideline documents ranged from 2009 to 2025, and 89 (57%) were released after 2018. Most (135; 86%) were developed by global, regional, or national academic associations (for example, World Health Organization,^91^ National Comprehensive Cancer Network 92). The full lists of the guidelines are in supplementary table S8.

### Evidence contamination and evidence contamination chain

**Fig 3:**
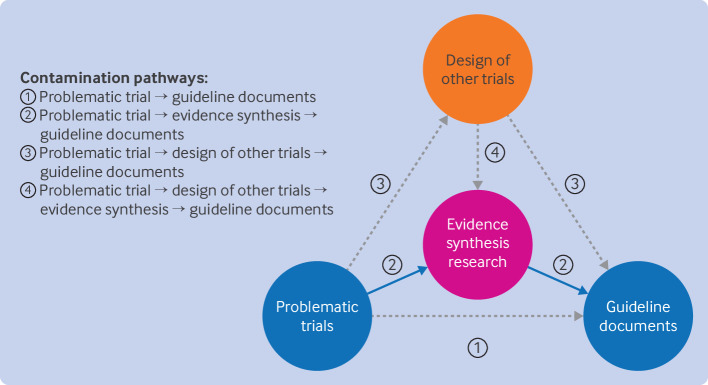
Contamination chain of retracted trials on evidence ecosystem

To show how retracted trials affect the evidence ecosystem, we established a simplified evidence contamination chain, shown in figure 3. Of the four potential contamination pathways, the one from retracted trials to evidence synthesis and then to clinical guideline documents may be the most important one. In terms of our quantitative analysis via forward citation searching, for the 1330 retracted trials, 312 (23.5%) caused contamination of 4095 meta-analyses from 847 systematic reviews, and the evidence from 218 meta-analyses (in 68 systematic reviews) that were substantially affected were further used in 157 English language clinical practice guidelines. This means that, on average, a single retracted trial would contaminate up to 13 meta-analyses from three systematic reviews, and each systematic review would further contaminate at least three clinical practice guidelines. Several examples are shown in table 2 and supplementary file, pages 391-393.

**Table 2:** Examples of retracted trials for clinical guideline documents via contaminating evidence synthesis

Systematic reviews	Outcomes substantially affected	Guidelines referring to contaminated evidence	Recommendations using evidence from review
Carlisle et al, 2006^93^	Comparisons: droperidol *v* granisetron; outcome (efficacy): nausea; with retracted trials (n=19): RR=1.36 (95% CI 1.05 to 1.77); without retracted trials (n=3): RR=0.94 (0.67 to 1.33)	Society for Ambulatory Anaesthesia guideline (2014): Management of Postoperative Nausea and Vomiting 94	Result of outcome not used in guideline
Carlisle et al, 2006^93^	Comparisons: placebo *v* droperidol; outcome (side effects): dizziness; with retracted trials (n=8): RR=1.01 (95% CI 0.40 to 2.54); without retracted trials (n=0): no evidence	Society for Ambulatory Anaesthesia guideline (2014): Management of Postoperative Nausea and Vomiting 94	Guideline 7. Ensure PONV prevention and treatment is implemented in the clinical setting. “The safety of antiemetics is well established considering the huge amount of clinical data available and their summary in valid meta-analyses”
Liu et al, 2014^95^	Comparisons: letrozole *v* clomiphene; outcome: No of dominant follicles; with retracted trials (n=4): MD=−0.40 (95% CI −1.68 to 0.89); without retracted trials (n=3): MD=0.20 (0.17 to 0.23)	CFAS Guideline (2019): The Management of Unexplained Infertility 96	Recommendation on: ovarian stimulation with oral agents alone. “Aromatase inhibitors alone do not offer any benefit in comparison to clomiphene citrate alone and should not be offered to couples with UEI (Level 1A).”
Barrons et al, 2016^97^	Comparisons: ACE inhibitors *v* placebo; outcome: walking distance; with retracted trials (n=4): MD=126 (95% CI −95 to 346); without retracted trials (n=2): MD=−47 (−71 to −24)	No guideline was identified via forward citation searching	NA

CI=confidence interval; MD=mean difference; NA=not applicable; RR=risk ratio.

## Discussion

### Principal findings

In this study, a large scale empirical investigation based on forward citation searching was conducted to evaluate the potential contamination cascade from retracted trials to the evidence ecosystem. The results suggest that, on average, a single retracted trial potentially contaminates approximately three systematic reviews, and each systematic review with meta-analyses contaminated by retracted trials would further contaminate almost three English language clinical practice guidelines. In addition, for meta-analyses in which retracted trials were present, the subsequent exclusion of the retracted trials led to a change in the direction of effects for 8.4% of the meta-analyses, a change in the magnitude by more than 50% in 15.7% of the meta-analyses, and a change in the significance of the P value in 16.0% of the pooled effect estimates. Our findings also indicate that this could lead to completely different conclusions (for example, change in both the direction of effects and the statistical significance) in at least 3.9% of meta-analyses, with subsequent contamination of 157 guideline documents.

The retracted trials (89.0%) were not usually multicentre studies, typically received academic funding or failed to report funding sources (89.5%), and were more likely to be unregistered (81.3%); these findings are in accordance with previous studies.^14^ ^98^ ^99^ In addition, we found that meta-analyses of harm outcomes were more susceptible to the impact of retracted trial data than were those of benefit outcomes; this could be expected as harm outcomes occur less frequently than benefits, involving larger random error and thus more fragile estimates.^100^ In addition, the numbers of studies reporting harm outcomes were often lower than for benefits (median: 6 *v* 7 in our dataset) owing to the less frequent nature and potential selective reporting problems.^101^ As meta-analyses with fewer studies were more likely to be affected, the impact of retracted trials on the results was more serious. The findings suggest that the nature of the data and the number of studies within a meta-analysis might be important factors in modulating the extent of the “contamination” of retracted trials on the results of the evidence synthesis.

Our forward citation searching showed that retracted trials continued to be cited after retraction, and the systematic reviews that synthesised these retractions also continued to be cited. Most the systematic reviews that synthesised retracted trials did not make a correction, and almost 40% of the reviews included retracted trials after the retraction, suggesting that these authors do not routinely assess the status of included studies and therefore remained unaware of the potential impact of retracted trials on their evidence synthesis. Although this problem has been identified previously,^102^ ^103^ ^104^ it has not yet been corrected. This problem becomes complex as we observed that even though some Cochrane reviews of the updated version removed the “old” retracted trials, new retractions occurred later (for example, Bordewijk et al^105^). Notably, we noticed that 172 of the retracted trials were from paper mills, which poses a big challenge to evidence synthesis research. The contamination cascade poses a serious risk, given the importance of data integrity to researchers, clinicians, and policy makers. More rigorous checks on the publication status of included trials should be required for authors of systematic reviews, whereas the burden of checking for retractions after publication of the systematic reviews would have to fall on the users of such meta-analyses or during updates of any systematic review.

### Implications for future evidence synthesis practice

Our findings have major implications for the field of evidence synthesis. One of the most challenging aspects of evidence synthesis practice is the identification of retracted trials.^106^ ^107^ Even where “signals” of concerns were observed, the investigation of problematic trials still took a long time. The median time from the submission of concern to the first journal correction is estimated to be 22.1 months.^108^ Existing databases keep current records of retracted trials, but almost inevitably many retractions will take place only after the completion and publication of associated systematic reviews. As such, we recommend the following approach for future evidence synthesis research. Firstly, authors of systematic review should identify already retracted trials before completion via multiple databases (for example, Retraction Watch, Scopus, Web of Science, PubMed) and pre-specify how such trials will be detected and handled in the review process. Secondly, many retractions occur well after publication of the evidence synthesis in question, so a priori evaluation of the integrity and trustworthiness of included trials may be useful for users of systematic reviews. Several important instruments have been released, such as the REAPPRIAISE checklist for checking publication integrity by Grey and colleagues,^109^ the Cochrane Pregnancy and Childbirth Trustworthiness Screening Tool (CPC‐TST) for assessment of trustworthiness,^110^ the TRACT checklist by Mol and colleagues for assessment of trustworthiness,^111^ and the ongoing project of INSPECT-SR for identifying problematic trials.^112^ Thirdly, for any evidence synthesis research, reviewers may wish to consider prioritising checking for retracted trials. Fourthly, authors of systematic reviews should work together with journal editors to publish updated systematic reviews and meta-analyses if any of the included trials have been retracted, although a method for flagging this is needed.

Further policy efforts should also make sure to keep up with the pace. For example, academic journals are expected to impose stricter policies on trial registration and data sharing. Although some journals (such as *The BMJ* and *PLoS Medicine*) already have such a policy,^113^ many journals still lack this. Some efforts have been made to track the potential retractions via forwards or backwards citation searching (for example, a systematic review accelerator called SpiderCite^114^) and to inform researchers when papers they have cited are retracted (for example, https://www.retracted.net/), and this may help the effort to correct the scientific record.

### Strengths and limitations of study

To the best of our knowledge, this is the first attempt to establish the “contamination chain” of retracted trials in the evidence ecosystem. We used established methods of comprehensive literature searches and forward citation searching to collect randomised controlled trials, subsequent systematic reviews that quantitatively synthesised evidence from these trials, and clinical practice guidelines that used the contaminated evidence from these systematic reviews, even if retractions occurred afterwards. We believe that our comprehensive search to obtain a broadly representative sample and the painstaking replication of each single meta-analysis are major strengths that lend greater credibility to our findings. We also used strict data collection and data analytic procedures that helped to ensure the reliability of our results.

The study has some limitations that need to be discussed. Firstly, our study did not include patients or members of the public. This may have some potential downsides on subsequent dissemination, which we plan to overcome through extensive use of lay friendly material for social media, both in Chinese and in English. Secondly, we were unable to track all clinical practice guidelines on the basis of a literature search of four databases, because some clinical practice guidelines may not have been indexed in bibliographic databases,^115^ ^116^ some were published in languages that were not included in our analysis, and some used evidence from randomised controlled trials directly, which would result in an underestimation of the number of affected guidelines. Thirdly, we focused on pair-wise meta-analyses only, so the impact of retracted trials on the results of network meta-analyses and any subsequent influence on relevant clinical practice guidelines needs to be further investigated. Fourthly, despite the use of Retraction Watch, we cannot be absolutely certain that we have identified all retracted trials included in systematic reviews. We also cannot be absolutely certain that we identified all systematic reviews that potentially synthesised evidence from retracted trials on the basis of forward citation searching, as some of the systematic reviews listed included studies in supplementary files (for example, Shi et al^117^). Moreover, the study focused only on retracted randomised trials, whereas some non-randomised studies of interventions would be retracted, which could also contaminate the evidence bodies. We may, therefore, have underestimated the impact of retracted trials on the results of systematic reviews and meta-analyses, as well as on clinical practice guidelines, and the real world contamination could possibly be even more serious.

### Conclusions

On the basis of empirical evidence, retracted trials had a substantial impact on the evidence ecosystem. The problematic influence of retracted trial data on the pooled effect estimates could distort clinical practice guidelines and mislead decision making by healthcare practitioners and policy makers. Evidence generators, evidence synthesisers, evidence users, and policy makers should pay serious attention to the “contamination” by retracted trials of the evidence ecosystem, and reasonable approaches that assist with easier identification and correction of such contamination are urgently needed.

## What is already known on this topic

In evidence based medicine, reliable decision making relies on the establishment of a trustworthy evidence ecosystem
Evidence generation and evidence synthesis are key elements for the establishment of such an ecosystem
The number of problematic or retracted studies has increased in the past decade, raising serious concern about the reliability of the data and conclusions of scientific research

## What this study adds

This study of 1330 retracted trials showed an “evidence contamination chain” from retracted trial to evidence synthesis research and clinical guideline documents
A quarter of the retracted trials were quantitatively synthesised in subsequent systematic reviews, and each trial could potentially “contaminate” three systematic reviews
Subsequently, each systematic review could further be used in three English language guideline documents

## Data Availability

The core datasets can be found at https://osf.io/ukg3j. The whole datasets could be obtained from the first author (xuchang2016@runbox.com) on request.

## Web extra

Extra material supplied by authors

Web appendix: Supplementary file

## Notes

Contributors: CX, HL, SD, YL, and SV conceived and designed the study. HL, FC, and CX contributed to funding acquisition and project management. CX contributed to literature screening, data collection, program coding, data analysis, visualisation, and manuscript drafting. SD contributed to statistical guidance, data checking, program coding, data analysis, and results interpretation. YT contributed to literature screening, data collection, data replication, data analysis, and results interpretation. SQF contributed to project management, literature screening, data collection, quality control, data replication, and data analysis. SQF and CX contributed to data verification. CX, SQF, and YT replicated the data. LFK, SYL, and CZ contributed to literature screening. SL, HL, SQF, and CX contributed to data checking. LL and HC provided statistical advice. SG and JC contributed to the search strategy. CX, SD, HL, PG, FCL, SL, SYL, SV, and YL contributed to results interpretation. HL, SV, LFK, SL, SYL, FCL, LL, HC, SD, PG, JC, SL, SG, and YL provided critical project advice and methodological comments. All authors carefully reviewed the manuscript and approved the final version to be published. CX and HL are the guarantors. The corresponding author attests that all listed authors meet authorship criteria and that no others meeting the criteria have been omitted.

Transparency: The lead authors (the manuscript’s guarantors) affirm that the manuscript is an honest, accurate, and transparent account of the study being reported; that no important aspects of the study have been omitted; and that any discrepancies from the study as originally planned (and, if relevant, registered) have been explained.

Dissemination to participants and related patient and public communities: We plan to present our findings at national and international scientific meetings. We also plan to use social media outlets to disseminate findings.

Provenance and peer review: Not commissioned; externally peer reviewed.
