# Supplementary material for: Investigating the impact of trial retractions on the healthcare evidence ecosystem (VITALITY Study I): retrospective cohort study
Source: BMJ. 2025 Apr 23;389:e082068. doi: 10.1136/bmj-2024-082068 (PMC12015725; doi:10.1136/bmj-2024-082068)
Supplement: Supplementary file 1 — Web appendix: Supplementary file [file xuch082068.ww1.pdf]

**Supplementary File**

**Protocol (Drafted in 13-April, 2023; Latesed updated in 16-May, 2024)**

# Impact of retracted randomized controlled trials on producing trustworthy healthcare evidence: a replicability study

Chang Xu[1], on behalf of the VITALITY Collaborative Research Network

1. Proof of Concept Center, Eastern Hepatobiliary Surgery Hospital, Third Affiliated Hospital, Second Military Medical University, Naval Medical University, Shanghai, China

**Correspondence:** Chang Xu, Proof of Concept Center, Eastern Hepatobiliary Surgery Hospital, Third Affiliated Hospital, Second Military Medical University, Naval Medical University, Shanghai, China; xuchang2016@runbox.com.

**Running title:** Retracted trials on evidence syntheses and clinical guidelines

**Word count:** 1,761

**Abstract**

**Background:** Evidence synthesis is a preferred method to summarize existing data of relevant studies for a certain topic to provide comprehensive evidence to support informed decision-making. However, the reliability of the synthesized evidence relies largely on the validity and quality of original studies. As an increasing number of published studies have been retracted due to insufficient validity, huge concerns around subsequent negative impacts have arisen. In this study, we aim to investigate the potential impact of retracted randomized controlled trials on the synthesized results and conclusions in healthcare practice.

**Methods and analyses:** Retracted randomized controlled trials will be searched via Web of Science and Retraction Watch databases up to May 1st, 2023. A data use agreement has been signed as required by Retraction Watch. Eligible retracted trials identified by two authors will be checked for their citations in Google Scholar, and any reviews with quantitative evidence syntheses that involved the retracted trials will be further identified. Re-analyses will be conducted by removing those studies that were retracted with the same statistical methods used by the review authors, and the direction of the effects as well as significance of *p*-values will be compared.

**Discussion:** This is the first study to investigate the potential impact of retracted trials on the results and conclusions of evidence synthesis research and their subsequent impact on healthcare practice guidelines. This study will have important implications for evidence synthesis practice, clinical practice guideline development, and healthcare decision-making.

**Ethics and dissemination:** No ethical approval is involved in this study. The findings of this study will be presented at an international scientific conference and published in a peer-reviewed academic journal.

**Keywords:** Evidence synthesis; Randomized controlled trial; Replicability; Research waste; Retracted publication.

## Introduction

Evidence synthesis summarizes existing data of relevant studies for a certain topic in order to provide comprehensive and precise, evidence to support informed decision-making [1]. However, it has long been recognized that the reliability of the synthesized evidence largely relies on the quality of original studies [2]. The concerns regarding study methodological quality pushes the research synthesis community to formulate regulations and guidelines and develop post-hoc measurement tools in an effort to promote the production of trustworthy evidence [3, 4]. Whilst such actions have resulted in significant improvements in the past decades, numerous new threats have arisen, such as reproducibility and replicability [5]. The increasing incidence of retractions of published research points to these new threats and has raised serious concerns over their subsequent negative impact [6].

Randomized trials serve as the main source of unbiased data for evidence synthesis, and systematic reviews/meta-analyses have been hierarchized as the highest level in the evidence pyramid [7]. Randomized trials enable the minimization of potential biases arising from methodological weaknesses relating to participant allocation, intervention delivery, and outcome measurement [8]. On this basis, synthesized evidence from randomized trials is widely adopted in clinical practice guidelines []. Researchers should note that the basic assumption underpinning such a paradigm is that all the included trials are valid and present unbiased and honest information in the publications. However, this is not always the case, as an increasing number of retracted clinical trials have been documented due to the questionable nature of their data [9, 10].

The increasing number s of randomized trial retractions raises the possibility of a severe threat to evidence-based medicine; When a retracted trial is included as part of evidence synthesis, is the evidence synthesis still reliable? The incorporation of retracted evidence in clinical practice guidelines may spread incorrect conclusions, mislead healthcare practice, cause pain and harm to patients, and contribute to substantial research waste [11]. Therefore, it is an urgent priority in the science of evidence synthesis to clarify how many evidence syntheses included trials that were already retracted and examine the impact of such trials on the robustness of the evidence. In this study, we aim to address these questions via a large-scale replicability study.

## Methods

### Design and context

This study will be comprised of five consecutive steps as shown in Figure 1. First, we will search for retracted studies via two databases and identify those that are clinical trials. Characteristics of these trials and the reasons for retraction will be collected and summarized. Second, retracted trials that were identified in the first step will be checked for their citations in Google Scholar, and any

types of quantitative evidence syntheses that involved those retracted trials will be identified. In the third step, the syntheses that included data from retracted trials will be entirely replicated after removing the retracted trials using the same statistical methods used in the original evidence synthesis, and the results will then be compared. Finally, several methods, for example, the quality-effect model, and subgroup analysis will be employed to re-analyze the metadata to see if any methods may mitigate the impact of retracted trials on the pooled evidence.

**Data sources**

***Identification of retracted clinical trials***

We used the definition by Charlesworth that a retraction '*is the removal of an article from the scientific record after the article is published.*', which differs from the concept of withdrawal, which refers to '*the removal of a manuscript from consideration by a journal upon request by the authors themselves before the article is published.*' [12].

Retraction Watch database and Web of Science will be searched for related retracted publications up to May 1st, 2023. For the Retraction Watch database, we will retrieve records labeled as "Clinical Study" directly, without any limitation on the topics. For Web of Science, a search strategy has been developed by an information specialist (ZLS) after discussions with the lead authors (JHY, CX); the literature search will be run by the information specialist. Only articles with notification of 'retraction' and 'expression of concern' will be considered; those with 'correction notice' contributing to enhancing scientific trust will be excluded as these are not retractions. Two authors (ZLS, JHY) will review the titles of the retracted publications obtained from Web of Science, and those featured as randomized controlled trials will be identified and included in this study The search strategy is presented in the Appendix.

***Identification of evidence syntheses involving retracted trials***

This study considers evidence synthesis as a method that combines information from multiple studies of the same topic, qualitatively or quantitatively, to obtain global knowledge for the given topic [13]. This includes all types of reviews (e.g., systematic reviews, rapid reviews, and umbrella reviews), meta-analyses, and pooled analyses. Current study will aim at quantitatively evidence synthesis (meta-analyses or pooled analyses, or reviews with meta-analyses or pooled analyses). To identify evidence syntheses researches that involved retracted trials, we will check the Google Scholar citations of the eligible retracted trials.

**Data collection**

Double independent data extraction will be done by 10 medical students. The following information will be collected: 1) information on retracted trials, including the DOI, total number of times it has

been cited, title, journal of publication, date of publication, date of retraction, reasons for retraction, number of authors, geographic region of the corresponding author, organizational affiliation of the corresponding author, interventions (e.g., medication, device, surgery), trial registration information, data sharing statement, source of funding, sample size, design information (e.g., parallel, cross-over, cluster), and risk of bias information; 2) information on evidence syntheses that involved retracted trials, including the total number of times the evidence synthesis was cited, journal of publication, year of publication, title, research type (e.g., systematic review, rapid review), methods of synthesis (quantitative or qualitative), type of meta-analysis (e.g., pairwise, multiple arms), location where retracted trial was cited (e.g., introduction, results, discussion), and data of each included studies used for the synthesis.

**Main outcomes**

The following outcomes will be of interest:

1) Characteristics of retracted trials and reasons for retraction;
2) Number and type of evidence syntheses that included retracted trials;
3) Proportion of retracted trials per pooled analysis and per synthesis;
4) Impact of retracted trials on the magnitude and direction of the pooled effects, impact of retracted trials on the significance of the *P* value;
5) Potential predictors for retracted trials.

**Statistical analysis**

For each evidence synthesis with quantitative data synthesis, we will replicate the analysis after removing retracted trials with the same synthesis method and the same effect estimates used in the original meta-analysis. If such information was not reported or insufficiently reported, we will refer to the forest plot and results for a subjective judgement by three senior methodologists (CX, LFL, LFK) separately. If consensus cannot be achieved, the replication will be done with the most appropriate method as determined by the three methodologists. The magnitude and direction of the effects and the significance of the *p*-value will be compared between the original synthesis and the replicated synthesis.

A generalized linear mixed model will be employed for potential predictors of retraction of clinical trials whenever feasible. In addition, a further re-analysis of the metadata by the quality-effects model [14] and different variants of the random-effects model (e.g., inverse variance random effects model, generalized linear mixed model) will be employed to see if any of these models could potentially mitigate the impact of retracted trials on the conclusions.

All analyses will be undertaken using the Stata SE/17 program and the R Studio program, with exact p values reported throughout

**Results**

None.

**Contact details for further information**

xuchang2016@runbox.com

**Conflicts of interest**

We declare no conflict of interest.

**Funding**

This study will be supported by the National Natural Science Foundation of China (72204003), three funding bodies from Anhui Medical University (9021783201, 0301001882, and 0301035204), the Teachers Research Foundation Project of Nanjing University of Posts and Telecommunications (NYY222042), the institutional funding by Shanghai Eastern Hepatobiliary Surgery Hospital of Navy Medical University, the National Natural Science Foundation of China (81970453，82270634, 82302906).

Stage

Ongoing: A pilot searching has been employed, while a formal literature screening for the current study has not been done (as of 20 April 2023).

**History**

Protocol drafting: 13-April, 2023
Version 1: 20-April, 2023
Version 2: 18-July, 2023 (edits on wording and structure, no substantial change on the methods)
Version 3: 4-Aug, 2023 (add limits of clinical study by randomized controlled trials)
Version 4: 12-Aug, 2023 (revision based on the comments of all authors)
Version 5: 8-Nov, 2023 (addressed the numerical error of the search results)
Version 6: 7-May, 2024 (some included retractions were identified as non-RCTs, the literature search for retractions will be redone, and further data will be updated)
Version 7: 10-May, 2024 (revise of the inclusion criteria, see below the amemdments)
Version 8: 16-May, 2024 (re-set the databases for literature search)

**Amendments of the protocol**

1) 1-Nov to 8-Nov, 2023: The research team finished the identification of systematic reviews with meta-analyses that synthesized retracted randomized trials, with the number of 871 (will be further checked). Based on our previous experience, there would be a huge amounts of work to extract metadata from the 871 systematic reviews. After an online discussion by email, all team members approved a plan that to invite another team which would provide us more research assistants and funding support for the next steps. And the invited team leader will share authorship with us upon discussion.
2) We consider to further investigate the potential impact of "contaminated meta-analyses" on clinical practical guidelines.
3) 16-Nov, 2023: We noted that some retracted RCTs were still cited during the process of our project (April, 2023 to present), thus, we hypothesis that the new citiations after the retraction may also involve systematic reviews with meta-analyses, and therefore, we plan to conduct a subgroup analysis of the impact of retracted RCTs on the results of meta-analyses by grouping all meta-analyses into two groups: one is meta-analyses published before the retraction of related retracted RCTs, and one is meta-analyses published after the retraction of those retracted RCTs.
4) 11-Dec, 2023: Change of the authorship. We have invited Professor Hui Liu from Shanghai Eastern Hepatobiliary Surgery Hospital of Navy Medical University to participant our projet, their team will participate for the following work: i) metadata extraction of the "contaminated meta-analyses", ii) provide guidance on the section of potential impact of "contaminated meta-analyses" on clinical practical guidelines, iii) drafting the manuscript together, iv) provide further funding for current project (one institutional funding by Shanghai Eastern Hepatobiliary Surgery Hospital of Navy Medical University, and three National Natural Science Foundation of China (81970453， 82270634, 82302906). And Professor Hui Liu will be the corresponding author of current project and Professor Chang Xu will be the first author.
5) 31-Dec, 2023: After several rounds discussion about the potential impact of "contaminated meta-analyses" on clinical practical guidelines, we plan to focus on the proportion of clinical practical guidelines that were possibly "contaminated" by those meta-analyses that were substantially impacted by retracted RCTs (i.e., those meta-analyses changed the direction of the effects or the significance of the P value). This will be done by a forward searching of the topic of these meta-analyses to identify relavant clinical practical guidelines that were released or published after the publication of these meta-analyses, and then identify the proportion of clinical practical guidelines cited these meta-analyses. The searching will be based on the following databases: National Guideline Clearinghouse, Guidelines international network, Turning Research into Practice database, Guideline Central, Sumsearch, Google scholar, Google, and PubMed. And one information specialist will perform the guideline searching.

6) 31-Dec, 2023: Considering the differencial nature of efficacy/effectiveness against harm outcomes, we also plan to conduct subgroup analysis about the impact of retracted RCTs on conclusions of meta-analyses based on the outcome type: benefits and harms.
7) 31-Dec, 2023: For the outcome of potential predictors of retracted RCTs, we will no longer treat this as an important outcome in this project, instead, we will compare the characteristics (e.g., sample size, design, risk of bias) of retracted RCTs and the remaining RCTs within a meta-analysis, based one generalized linear mixed model, in a separate paper.
8) 31-Dec, 2023: We plan to add an additional topic based on current project (where we refers to the "uncover the contamination" of retracted RCTs on clinical evidence). The additional topic will focus on "resolve the contamination" of retracted RCTs on clinical evidence. The topic will consist of three sections, with the plan of each section as follows.

   **Section 1:** For systematic reviews with meta-analyses that synthesized evidence from retracted randomized controlled trials (here as 'contaminated evidence'), searching for the following meta-analyses of the same topic based on PICO, and further checking: 1) whether these 'follow-up meta-analyses' corrected the contamination due to retracted randomized controlled trials; 2) when these 'follow-up meta-analyses' corrected the contamination; and 3) how these follow-up meta-analyses dealt with the retracted randomized controlled trials;

   **Section 2:** The potential methods for minimizing the contamination due to 'problematic' randomized controlled trials when review authors don't know which trials were or would be retracted, these include using conservative weighting scheme (e.g., random effect model, IVhet model), cooperating quality as part of the weighting scheme (Quality effect model), sensitivity/subgroup analysis based on quality, sensitivity analysis on the 'leave-one-out' basis, Grading evidence level of the meta-analysis.

   **Section 3:** Practical guidelines for future meta-analyses.
9) 5-May, 2024: Since the reviewers found some included retractions were non-RCTs, and one reviewer raised concerns of the search strategy, after a discussion with the core author teams (Sunita Vohra, Yoon Look, Suhail Doi, Luis Furuya-Kanamori, Su Golder, Chang Xu, and Hui Liu,), we plan to check all of the included retractions, and re do the literature search and screen on Retraction Watch Database based on to authors independently in a double-screen matter (Chang Xu, and Tian Yuan).
10) 8-May, 2024: After a carefully discussion with two senior informationilists (Su Golder and Justin Clark) of the search strategy, we decided to only search the Retraction Watch database, this is because as described in the Retraction Watch website this database has already searched for PubMed, Web of Science, Scopus, Google Scholar, publishers, and academic journals.

<u>**Notes:** The primary searching and screen of the retractions was conducted by Sun zhuanlan's team, and after a carefully discussion, the re-work of these steps will be took over by Chang' team.</u>

The revion project was developed as below:

**Revision project**

**Step 1:** Check the 764 included retractions, and exclude non-RCTs.

(*Participants:* Chang Xu, Yuan Tian; *Senior methodologist for disagreements:* Luis)

**Step 2:** Re-screen the results of the Retraction Watch (N = 1,641), and compare the inclusions with the final inclusion based on step 1.

(*Participants:* Chang Xu, Yuan Tian; *Senior methodologist for disagreements:* Luis)

**Step 3:** Re searching the Retraction Watch under the filter of: "(HSC) Health Sciences" and limited the article type as "Research Article", and limited the date of retraction as up to "April-26, 2023", and limited the Nature of notice as "Retraction".

(*Participants:* Chang Xu, Yuan Tian)

**Step 4:** Screen the records from step 3, by 6 to 8 team members, with each group 3-4 members, independently. First by titles and abstracts, and then by full-texts.

**Step 5:** Baseline information extraction for the final inclusion of the retractions in step 1, and new retracted RCTs in step 2 and 4. (Two groups of team members, independently)

**Step 6:** See below two sub-steps

1) For non-RCTs identified in step 1, re-check all of the relevant systematic reviews and meta-analyses involves these non-RCTs, exclude those SRs with retractions only contains these retracted non-RCTs, update the data, redo the replication (*Participants:* Chang Xu, Yuan Tian, Shiqi Fan).
2) For new retracted RCTs identified in step 2 and 4, forward citation searching via Google Scholar for these new retracted RCTs, identifying new systematic reviews and meta-analyses that synthesized these RCTs, check all the already included systematic reviews and meta-analyses to see if they also synthesis these RCTs, and update the data, do/redo the replication for these meta-analyses (*Participants:* Chang Xu, Yuan Tian, Shiqi Fan).

**Step 7:** Update all of the data analyses, figures, tables, supplementary file based on the requirement (including added any information of current study), and also update the third parts of the study — contamination of CPGs (*Participants:* Chang Xu, Suhail, Yuan Tian, Shiqi Fan).

11) 8-May, 2024: Changing of the inclusion criteria. After an online discussion of the core methodologists (Suhail Doi, Luis Furuya-Kanamori, and Chang Xu), we made two changes of the inclusion criteria. First, we excluded retractions without full-text due to the limited information for the judgement of whether it is an RCT; Second, we excluded retractions other than English, due to the fact that it is hard to accurately made a judgement of whether it is an RCT. Third, we excluded retractions of "Letter", "Conference abstract", "Oral communications", "Brief reports", even they were based on randomized controlled design,

because the information reported from these reports were also limited, and many such short reports would publish the full-length paper further. Fourth, we now only consider "retractions" and no longer consider "Expression of concerns". Fifth, for some retractions of the data were taken from an randomized trial (which refers to secondary analysis), we used the inclusion criteria after our discussion that when the method for secondary analysis do not broken the randomization (for example, use the same data from original trial), we would include, otherwise exclude. The change of the inclusion criteria would lead to a slight underestimation of the impact of problematic trials on the evidence ecosystem.

12) 16-May, 2024: An addition search on Retraction Watch under the filter of: "(HSC) Health Sciences" and limited the article type as "Research Article", and limited the date of retraction as up to "April-26, 2023", and limited the Nature of notice as "Retraction". (As planned above, see "Revision project" of the Step 3) .
13) 7-June, 2024: The individual (s) responsible for the section of retracted trial identification of the previous version chose to leave the the investigator team – they are no longer authors on the revised work.

**Reference**


1. Gurevitch J, Koricheva J, Nakagawa S, et al. Meta-analysis and the science of research synthesis. Nature. 2018;555(7695):175-182.
2. Murad MH, Montori VM. Synthesizing evidence: shifting the focus from individual studies to the body of evidence. JAMA. 2013;309(21):2217-8.
3. Moher D, Liberati A, Tetzlaff J, et al. Preferred reporting items for systematic reviews and meta-analyses: the PRISMA statement. PLoS Med. 2009;6(7):e1000097.
4. Guyatt GH, Oxman AD, Vist GE, et al. GRADE: an emerging consensus on rating quality of evidence and strength of recommendations. BMJ. 2008;336(7650):924-6.
5. Parker TH, Forstmeier W, Koricheva J, et al. Transparency in Ecology and Evolution: Real Problems, Real Solutions. Trends Ecol Evol. 2016;31(9):711-719.
6. Else H. Multimillion-dollar trade in paper authorships alarms publishers. Nature. 2023;613(7945):617-618.
7. Schulz KF, Altman DG, Moher Det al. CONSORT 2010 statement: updated guidelines for reporting parallel group randomised trials. BMJ. 2010;340:c332.
8. Higgins JPT, Savović J, Page MJ, et al. Cochrane Handbook for Systematic Reviews of Interventions version 6.3 (updated February 2022). Cochrane, 2022. Available from www.training.cochrane.org/handbook.
9. Wiedermann CJ, Joannidis M. The Boldt scandal still in need of action: the example of colloids 10 years after initial suspicion of fraud. Intensive Care Med. 2018;44(10):1735-1737.
10. Wadhwa RR, Rasendran C, Popovic ZB, et al. Temporal Trends, Characteristics, and Citations

of Retracted Articles in Cardiovascular Medicine. JAMA Netw Open. 2021; 4(7):e2118263.

11. Boughton SL, Wilkinson J, Bero L. When beauty is but skin deep: dealing with problematic studies in systematic reviews. Cochrane Database Syst Rev. 2021 Jun 3;6(6):ED000152.
12. Charlesworth Author Services. All about Article Retraction in academic publishing (updated 16 September 2020 ). Available from https://www.cwauthors.com/article/What-is-article-retraction-in-academic-publishing
13. Lortie CJ, Stewart G, Rothstein H,et al. How to critically read ecological meta-analyses. Res Synth Methods. 2015; 6(2):124-33.
14. Doi SA, Barendregt JJ, Khan S, Thalib L, Williams GM. Advances in the meta-analysis of heterogeneous clinical trials II: The quality effects model. Contemp Clin Trials. 2015; 45(Pt A):123-9.

**Supplementary Table S1. Search Strategy**

| Search strategy | Search date | Database | Number of records |
|---|---|---|---|
| Article Type(s) = “Clinical Study” AND ature of notice =”Retraction”, with the limits on Date of retraction as up to 2023/4/26. | 2023/4/26, updated in 2024/11/5 | Retreation Watch | 2,233 |
| Article Type(s) = “Research Artcile” AND Subjects = “(HSC) Health Sciences” AND Nature of notice =”Retraction”, with the limits on Date of retraction as up to 2023/4/26. | 2024/5/16 updated in 2024/11/5 | Retreation Watch | 10,392 |

**Filter for the automatic screen (abstracts and titles) for citations from Scopus**

1. Meta-
2. Meta-analysis
3. Metaanalysis
4. Meta-review
5. Systematic review
6. Scoping review
7. Umbrella review
8. Integrated review
9. Integrative review
10. Quantitative review
11. Comprehensive review
12. Literature review
13. Overview
14. Literature search
15. systematic search
16. Data synthesis
17. evidence synthesis
18. Research synthesis
19. Quantitative synthesis
20. Qualitative synthesis
21. Cochrane

**Test for the filter**

We manully screened 2,846 citations (limited on 'Article' and 'Review') of the PREDIMED study [1] that exported from Scopus to test the sensitivity of the above filter. By viewing the titles and abstracts of the 2,846 records via Rayyan, 175 were judged as 'included' and 91 were judged as 'maybe'. A further full-text screen for the 266 records yield a final inclusion of 161 systematic reviews.

At the same time, the filter was used to automatically screen the 2,846 records, where 2499 records were 'filtered out', and 347 were identified. We then compared the 161 records by manully to the 347 records by filter, 159 of the 161 records were 'coverred' in the 347 records, with a sensitivity of 98.76%, and the 2 were not covered were further checked and both did not had a quantitative synthesis of the data, see the following Table (Table S2).

**Reference**

[1] Estruch R, Ros E, Salas-Salvadó J, Covas MI, et al; PREDIMED Study Investigators. Primary prevention of cardiovascular disease with a Mediterranean diet. N Engl J Med. 2013 Apr 4;368(14):1279-90. doi: 10.1056/NEJMoa1200303.

**Supplementary Table S2.** Sensitivity test for the filter (159 of the 161 were covered in 347 records by filter).

| ID | Screened by key words (filter) | Screened by humans | Coverred |
|---|---|---|---|
| 1 | A comprehensive meta-analysis on evidence of Mediterranean diet and cardiovascular disease: Are individual components equal? | A comprehensive meta-analysis on evidence of Mediterranean diet and cardiovascular disease: Are individual components equal? | Yes |
| 2 | A greater flavonoid intake is associated with lower total and cause-specific mortality: A meta-analysis of cohort studies | A greater flavonoid intake is associated with lower total and cause-specific mortality: A meta-analysis of cohort studies | Yes |
| 3 | A journey into a Mediterranean diet and type 2 diabetes: A systematic review with meta-analyses | A journey into a Mediterranean diet and type 2 diabetes: A systematic review with meta-analyses | Yes |
| 4 | A Mediterranean diet improves HbA1c but not fasting blood glucose compared to alternative dietary strategies: A network meta-analysis | A Mediterranean diet improves HbA1c but not fasting blood glucose compared to alternative dietary strategies: A network meta-analysis | Yes |
| 5 | A systematic review and meta-analysis of nut consumption and incident risk of CVD and all-cause mortality | A systematic review and meta-analysis of nut consumption and incident risk of CVD and all-cause mortality | Yes |
| 6 | A systematic review of behavioural interventions promoting healthy eating among older people | A systematic review of behavioural interventions promoting healthy eating among older people | Yes |
| 7 | A systematic review of high-oleic vegetable oil substitutions for other fats and oils on cardiovascular disease risk factors: Implications for novel high-oleic soybean oils | A systematic review of high-oleic vegetable oil substitutions for other fats and oils on cardiovascular disease risk factors: Implications for novel high-oleic soybean oils | Yes |
| 8 | A systematic review of the effect of dietary saturated and polyunsaturated fat on heart disease | A systematic review of the effect of dietary saturated and polyunsaturated fat on heart disease | Yes |
| 9 | A Systematic Review of the Prevalence and Outcomes of Ideal Cardiovascular Health in US and Non-US Populations | A Systematic Review of the Prevalence and Outcomes of Ideal Cardiovascular Health in US and Non-US Populations | Yes |
| 10 | A systematic scoping review of how healthcare organizations are facilitating access to fruits and vegetables in their patient populations | A systematic scoping review of how healthcare organizations are facilitating access to fruits and vegetables in their patient populations | Yes |
| 11 | Adherence to a healthy diet according to the world health organization guidelines and all-cause mortality in elderly adults from Europe and the United States | Adherence to a healthy diet according to the world health organization guidelines and all-cause mortality in elderly adults from Europe and the United States | Yes |
| 12 | Adherence to Mediterranean diet and risk of cancer: A systematic review and meta-analysis of observational studies | Adherence to Mediterranean diet and risk of cancer: A systematic review and meta-analysis of observational studies | Yes |
| 13 | Adherence to mediterranean diet and risk of cancer: An updated systematic review and meta-analysis | Adherence to mediterranean diet and risk of cancer: An updated systematic review and meta-analysis | Yes |
| 14 | Adherence to Mediterranean diet and risk of developing cognitive disorders: An updated systematic review and meta-analysis of prospective cohort studies | Adherence to Mediterranean diet and risk of developing cognitive disorders: An updated systematic review and meta-analysis of prospective cohort studies | Yes |

| | | | |
|---|---|---|---|
| 15 | Adherence to the dietary approaches to stop hypertension diet and risk of stroke: A meta-analysis of prospective studies | Adherence to the dietary approaches to stop hypertension diet and risk of stroke: A meta-analysis of prospective studies | Yes |
| 16 | Adherence to the mediterranean diet during the covid-19 national lockdowns: A systematic review of observational studies | Adherence to the mediterranean diet during the covid-19 national lockdowns: A systematic review of observational studies | Yes |
| 17 | Adherence to the Mediterranean Diet in children and adolescents: A systematic review | Adherence to the Mediterranean Diet in children and adolescents: A systematic review | Yes |
| 18 | Adherence to the Mediterranean Diet in Pregnancy and Its Benefits on Maternal-Fetal Health: A Systematic Review of the Literature | Adherence to the Mediterranean Diet in Pregnancy and Its Benefits on Maternal-Fetal Health: A Systematic Review of the Literature | Yes |
| 19 | Anthocyanin Tissue Bioavailability in Animals: Possible Implications for Human Health. A Systematic Review | Anthocyanin Tissue Bioavailability in Animals: Possible Implications for Human Health. A Systematic Review | Yes |
| 20 | Anti-hyperlipidemia of garlic by reducing the level of total cholesterol and low-density lipoprotein | Anti-hyperlipidemia of garlic by reducing the level of total cholesterol and low-density lipoprotein | Yes |
| 21 | Antioxidant vitamin supplementation reduces arterial stiffness in adults: A systematic review and meta-analysis of randomized controlled trials | Antioxidant vitamin supplementation reduces arterial stiffness in adults: A systematic review and meta-analysis of randomized controlled trials | Yes |
| 22 | Association Between Work-Related Factors and Diet: A Review of the Literature | Association Between Work-Related Factors and Diet: A Review of the Literature | Yes |
| 23 | Association of behaviour change techniques with effectiveness of dietary interventions among adults of retirement age: A systematic review and meta-analysis of randomised controlled trials | Association of behaviour change techniques with effectiveness of dietary interventions among adults of retirement age: A systematic review and meta-analysis of randomised controlled trials | Yes |
| 24 | Association of Mediterranean diet with mild cognitive impairment and Alzheimer's disease: A systematic review and meta-analysis | Association of Mediterranean diet with mild cognitive impairment and Alzheimer's disease: A systematic review and meta-analysis | Yes |
| 25 | Association of metabolically healthy obesity and elevated risk of coronary artery calcification: A systematic review and meta-analysis | Association of metabolically healthy obesity and elevated risk of coronary artery calcification: A systematic review and meta-analysis | Yes |
| 26 | Associations between genotype–diet interactions and weight loss—a systematic review | Associations between genotype–diet interactions and weight loss—a systematic review | Yes |
| 27 | Baseline diabetes as a way to predict CV outcomes in a lipid-modifying trial: A meta-analysis of 330,376 patients from 47 landmark studies | Baseline diabetes as a way to predict CV outcomes in a lipid-modifying trial: A meta-analysis of 330,376 patients from 47 landmark studies | Yes |
| 28 | Behavioral Counseling to Promote a Healthy Diet and Physical Activity for Cardiovascular Disease Prevention in Adults with Cardiovascular Risk Factors: Updated Evidence Report and Systematic Review for the US Preventive Services Task Force | Behavioral Counseling to Promote a Healthy Diet and Physical Activity for Cardiovascular Disease Prevention in Adults with Cardiovascular Risk Factors: Updated Evidence Report and Systematic Review for the US Preventive Services Task Force | Yes |
| 29 | Caloric restriction in heart failure: A systematic review | Caloric restriction in heart failure: A systematic review | Yes |

| | | | |
|---|---|---|---|
| 30 | Cardiometabolic risk factors in vegans; A meta-analysis of observational studies | Cardiometabolic risk factors in vegans; A meta-analysis of observational studies | Yes |
| 31 | Carotid Intima-Media Thickness Progression as Surrogate Marker for Cardiovascular Risk: Meta-Analysis of 119 Clinical Trials Involving 100 667 Patients | Carotid Intima-Media Thickness Progression as Surrogate Marker for Cardiovascular Risk: Meta-Analysis of 119 Clinical Trials Involving 100 667 Patients | Yes |
| 32 | Central obesity and the Mediterranean diet: A systematic review of intervention trials | Central obesity and the Mediterranean diet: A systematic review of intervention trials | Yes |
| 33 | Consumption of nuts and legumes and risk of incident ischemic heart disease, stroke, and diabetes: A systematic review and meta-analysis | Consumption of nuts and legumes and risk of incident ischemic heart disease, stroke, and diabetes: A systematic review and meta-analysis | Yes |
| 34 | Consumption of nuts and legumes and risk of stroke: A meta-analysis of prospective cohort studies | Consumption of nuts and legumes and risk of stroke: A meta-analysis of prospective cohort studies | Yes |
| 35 | Consumption of yogurt and the incident risk of cardiovascular disease: A meta-analysis of nine cohort studies | Consumption of yogurt and the incident risk of cardiovascular disease: A meta-analysis of nine cohort studies | Yes |
| 36 | Dairy product consumption was associated with a lower likelihood of non-alcoholic fatty liver disease: A systematic review and meta-analysis | Dairy product consumption was associated with a lower likelihood of non-alcoholic fatty liver disease: A systematic review and meta-analysis | Yes |
| 37 | Dietary Change Interventions for Undergraduate Populations: Systematic Review and Recommendations | Dietary Change Interventions for Undergraduate Populations: Systematic Review and Recommendations | Yes |
| 38 | Dietary habits of adolescents living in North America, Europe or Oceania: A review on fruit, vegetable and legume consumption, sodium intake, and adherence to the Mediterranean Diet | Dietary habits of adolescents living in North America, Europe or Oceania: A review on fruit, vegetable and legume consumption, sodium intake, and adherence to the Mediterranean Diet | Yes |
| 39 | Dietary interventions for adults with chronic kidney disease | Dietary interventions for adults with chronic kidney disease | Yes |
| 40 | Dietary interventions for multiple sclerosis-related outcomes | Dietary interventions for multiple sclerosis-related outcomes | Yes |
| 41 | Dietary interventions in overweight and obese pregnant women: A systematic review of the content, delivery, and outcomes of randomized controlled trials | Dietary interventions in overweight and obese pregnant women: A systematic review of the content, delivery, and outcomes of randomized controlled trials | Yes |
| 42 | Dietary patterns and cognitive health in older adults: A systematic review | Dietary patterns and cognitive health in older adults: A systematic review | Yes |
| 43 | Dietary patterns and CVD: A systematic review and meta-analysis of observational studies | Dietary patterns and CVD: A systematic review and meta-analysis of observational studies | Yes |
| 44 | Dietary patterns and fatty liver disease | Dietary patterns and fatty liver disease | Yes |
| 45 | Dietary patterns and stroke: A systematic review and re-meta-analysis | Dietary patterns and stroke: A systematic review and re-meta-analysis | Yes |
| 46 | Dietary patterns, Mediterranean diet, and cardiovascular disease | Dietary patterns, Mediterranean diet, and cardiovascular disease | Yes |

| | | | |
|---|---|---|---|
| 47 | Dietary total fat, fatty acids intake, and risk of cardiovascular disease: A dose-response meta-analysis of cohort studies | Dietary total fat, fatty acids intake, and risk of cardiovascular disease: A dose-response meta-analysis of cohort studies | Yes |
| 48 | Do healthier foods and diet patterns cost more than less healthy options? A systematic review and meta-analysis | Do healthier foods and diet patterns cost more than less healthy options? A systematic review and meta-analysis | Yes |
| 49 | Does cooking with vegetable oils increase the risk of chronic diseases?: A systematic review | Does cooking with vegetable oils increase the risk of chronic diseases?: A systematic review | Yes |
| 50 | Does dietary fat affect inflammatory markers in overweight and obese individuals?—a review of randomized controlled trials from 2010 to 2016 | Does dietary fat affect inflammatory markers in overweight and obese individuals?—a review of randomized controlled trials from 2010 to 2016 | Yes |
| 51 | Effect of Breastfeeding in Early Life on Cardiorespiratory and Physical Fitness: A Systematic Review and Meta-Analysis | Effect of Breastfeeding in Early Life on Cardiorespiratory and Physical Fitness: A Systematic Review and Meta-Analysis | Yes |
| 52 | Effect of chronic consumption of pistachios (Pistacia vera L.) on glucose metabolism in pre-diabetics and type 2 diabetics: A systematic review | Effect of chronic consumption of pistachios (Pistacia vera L.) on glucose metabolism in pre-diabetics and type 2 diabetics: A systematic review | Yes |
| 53 | Effect of Dietary and Supplemental Lycopene on Cardiovascular Risk Factors: A Systematic Review and Meta-Analysis | Effect of Dietary and Supplemental Lycopene on Cardiovascular Risk Factors: A Systematic Review and Meta-Analysis | Yes |
| 54 | Effect of dietary patterns on cardiovascular risk factors in people with type 2 diabetes. A systematic review and network meta-analysis | Effect of dietary patterns on cardiovascular risk factors in people with type 2 diabetes. A systematic review and network meta-analysis | Yes |
| 55 | Effect of Palm Oil Consumption on Plasma Lipid Concentrations Related to Cardiovascular Disease: A Systematic Review and Meta-Analysis | Effect of Palm Oil Consumption on Plasma Lipid Concentrations Related to Cardiovascular Disease: A Systematic Review and Meta-Analysis | Yes |
| 56 | Effect of pasta in the context of low-glycaemic index dietary patterns on body weight and markers of adiposity: A systematic review and meta-analysis of randomised controlled trials in adults | Effect of pasta in the context of low-glycaemic index dietary patterns on body weight and markers of adiposity: A systematic review and meta-analysis of randomised controlled trials in adults | Yes |
| 57 | Effect of pistachio on brachial artery diameter and flow-mediated dilatation: A systematic review and meta-analysis of randomized, controlled-feeding clinical studies | Effect of pistachio on brachial artery diameter and flow-mediated dilatation: A systematic review and meta-analysis of randomized, controlled-feeding clinical studies | Yes |
| 58 | Effect of plant protein on blood lipids: A systematic review and meta-analysis of randomized controlled trials | Effect of plant protein on blood lipids: A systematic review and meta-analysis of randomized controlled trials | Yes |
| 59 | Effect of the Mediterranean diet on cognition and brain morphology and function: A systematic review of randomized controlled trials | Effect of the Mediterranean diet on cognition and brain morphology and function: A systematic review of randomized controlled trials | Yes |
| 60 | Effect of tree nuts on metabolic syndrome criteria: A systematic review and meta-analysis of randomised controlled trials | Effect of tree nuts on metabolic syndrome criteria: A systematic review and meta-analysis of randomised controlled trials | Yes |

| | | | |
|---|---|---|---|
| 61 | Effect of weight loss on blood pressure changes in overweight patients: A systematic review and meta-analysis | Effect of weight loss on blood pressure changes in overweight patients: A systematic review and meta-analysis | Yes |
| 62 | Effectiveness of dietary interventions among adults of retirement age: A systematic review and meta-analysis of randomized controlled trials | Effectiveness of dietary interventions among adults of retirement age: A systematic review and meta-analysis of randomized controlled trials | Yes |
| 63 | Effectiveness of interventions applicable to primary health care settings to promote Mediterranean diet or healthy eating adherence in adults: A systematic review | Effectiveness of interventions applicable to primary health care settings to promote Mediterranean diet or healthy eating adherence in adults: A systematic review | Yes |
| 64 | Effects of different dietary interventions on blood pressure: Systematic review and meta-analysis of randomized controlled trials | Effects of different dietary interventions on blood pressure: Systematic review and meta-analysis of randomized controlled trials | Yes |
| 65 | Effects of High and Low Fat Dairy Food on Cardio-Metabolic Risk Factors: A Meta-Analysis of Randomized Studies | Effects of High and Low Fat Dairy Food on Cardio-Metabolic Risk Factors: A Meta-Analysis of Randomized Studies | Yes |
| 66 | Effects of low-carbohydrate diet versus low-fat diet on non-alcoholic fatty liver disease: a meta-analysis of randomized controlled trials | Effects of low-carbohydrate diet versus low-fat diet on non-alcoholic fatty liver disease: a meta-analysis of randomized controlled trials | Yes |
| 67 | Effects of orange juice intake on cardiovascular risk factors: A systematic review and meta-analysis of randomized controlled clinical trials | Effects of orange juice intake on cardiovascular risk factors: A systematic review and meta-analysis of randomized controlled clinical trials | Yes |
| 68 | Effects of tart cherry juice consumption on cardio-metabolic risk factors: A systematic review and meta-analysis of randomized-controlled trials | Effects of tart cherry juice consumption on cardio-metabolic risk factors: A systematic review and meta-analysis of randomized-controlled trials | Yes |
| 69 | Effects of the dietary approach to stop hypertension (DASH) diet on cardiovascular risk factors: A systematic review and meta-analysis | Effects of the dietary approach to stop hypertension (DASH) diet on cardiovascular risk factors: A systematic review and meta-analysis | Yes |
| 70 | Effects of the mediterranean diet on cardiovascular outcomes-a systematic review and meta-analysis | Effects of the mediterranean diet on cardiovascular outcomes-a systematic review and meta-analysis | Yes |
| 71 | Effects of tree nuts on blood lipids, apolipoproteins, and blood pressure: Systematic review, meta-analysis, and dose-response of 61 controlled intervention trials | Effects of tree nuts on blood lipids, apolipoproteins, and blood pressure: Systematic review, meta-analysis, and dose-response of 61 controlled intervention trials | Yes |
| 72 | Effects of walnut consumption on blood lipids and other cardiovascular risk factors: An updated meta-analysis and systematic review of controlled trials | Effects of walnut consumption on blood lipids and other cardiovascular risk factors: An updated meta-analysis and systematic review of controlled trials | Yes |
| 73 | Effects on health outcomes of a mediterranean diet with no restriction on fat intake: A systematic review and meta-analysis | Effects on health outcomes of a mediterranean diet with no restriction on fat intake: A systematic review and meta-analysis | Yes |
| 74 | Evaluation of Dietary Patterns and All-Cause Mortality A Systematic Review | Evaluation of Dietary Patterns and All-Cause Mortality A Systematic Review | Yes |

| | | | |
|---|---|---|---|
| 75 | Evidence from randomised controlled trials does not support current dietary fat guidelines: A systematic review and meta-analysis | Evidence from randomised controlled trials does not support current dietary fat guidelines: A systematic review and meta-analysis | Yes |
| 76 | Evidence of the importance of dietary habits regarding depressive symptoms and depression | Evidence of the importance of dietary habits regarding depressive symptoms and depression | Yes |
| 77 | Food as medicine? Exploring the impact of providing healthy foods on adherence and clinical and economic outcomes | Food as medicine? Exploring the impact of providing healthy foods on adherence and clinical and economic outcomes | Yes |
| 78 | Food groups and intermediate disease markers: A systematic review and network meta-analysis of randomized trials | Food groups and intermediate disease markers: A systematic review and network meta-analysis of randomized trials | Yes |
| 79 | Food groups and risk of type 2 diabetes mellitus: a systematic review and meta-analysis of prospective studies | Food groups and risk of type 2 diabetes mellitus: a systematic review and meta-analysis of prospective studies | Yes |
| 80 | Food groups and the likelihood of non-alcoholic fatty liver disease: A systematic review and meta-analysis | Food groups and the likelihood of non-alcoholic fatty liver disease: A systematic review and meta-analysis | Yes |
| 81 | Fragility of cardiovascular outcome trials (CVOTs) examining nutrition interventions among patients with diabetes mellitus: a systematic review of randomized controlled trials | Fragility of cardiovascular outcome trials (CVOTs) examining nutrition interventions among patients with diabetes mellitus: a systematic review of randomized controlled trials | Yes |
| 82 | Health, not weight loss, focused programmes versus conventional weight loss programmes for cardiovascular risk factors: A systematic review and meta-analysis | Health, not weight loss, focused programmes versus conventional weight loss programmes for cardiovascular risk factors: A systematic review and meta-analysis | Yes |
| 83 | Healthy dietary patterns and risk of mortality and ESRD in CKD: A meta-analysis of cohort studies | Healthy dietary patterns and risk of mortality and ESRD in CKD: A meta-analysis of cohort studies | Yes |
| 84 | Impact of avocado-enriched diets on plasma lipoproteins: A meta-analysis | Impact of avocado-enriched diets on plasma lipoproteins: A meta-analysis | Yes |
| 85 | Impact of regulatory interventions to reduce intake of artificial trans-fatty acids: A systematic review | Impact of regulatory interventions to reduce intake of artificial trans-fatty acids: A systematic review | Yes |
| 86 | Interventions involving a major dietary component improve cognitive function in cognitively healthy adults: a systematic review and meta-analysis | Interventions involving a major dietary component improve cognitive function in cognitively healthy adults: a systematic review and meta-analysis | Yes |
| 87 | Japanese-Style Diet and Cardiovascular Disease Mortality: A Systematic Review and Meta-Analysis of Prospective Cohort Studies | Japanese-Style Diet and Cardiovascular Disease Mortality: A Systematic Review and Meta-Analysis of Prospective Cohort Studies | Yes |
| 88 | Legume consumption and CVD risk: A systematic review and meta-analysis | Legume consumption and CVD risk: A systematic review and meta-analysis | Yes |

| | | | |
|---|---|---|---|
| 89 | Long-term effects of increasing omega-3, omega-6 and total polyunsaturated fats on inflammatory bowel disease and markers of inflammation: a systematic review and meta-analysis of randomized controlled trials | Long-term effects of increasing omega-3, omega-6 and total polyunsaturated fats on inflammatory bowel disease and markers of inflammation: a systematic review and meta-analysis of randomized controlled trials | Yes |
| 90 | Long-term impact of mediterranean diet on cardiovascular disease prevention: A systematic review and meta-analysis of randomized controlled trials | Long-term impact of mediterranean diet on cardiovascular disease prevention: A systematic review and meta-analysis of randomized controlled trials | Yes |
| 91 | Lycopene and tomato and risk of cardiovascular diseases: A systematic review and meta-analysis of epidemiological evidence | Lycopene and tomato and risk of cardiovascular diseases: A systematic review and meta-analysis of epidemiological evidence | Yes |
| 92 | Medication therapy management challenges in patients with diabetes: A systematic review | Medication therapy management challenges in patients with diabetes: A systematic review | Yes |
| 93 | Mediterranean diet adherence, gut microbiota, and Alzheimer's or Parkinson's disease risk: A systematic review | Mediterranean diet adherence, gut microbiota, and Alzheimer's or Parkinson's disease risk: A systematic review | Yes |
| 94 | Mediterranean diet and cardiodiabesity: A review | Mediterranean diet and cardiodiabesity: A review | Yes |
| 95 | Mediterranean diet and cardiodiabesity: A systematic review through evidence-based answers to key clinical questions | Mediterranean diet and cardiodiabesity: A systematic review through evidence-based answers to key clinical questions | Yes |
| 96 | Mediterranean diet and cardiovascular disease: a systematic review and meta-analysis of observational studies | Mediterranean diet and cardiovascular disease: a systematic review and meta-analysis of observational studies | Yes |
| 97 | Mediterranean diet and health status: An updated meta-analysis and a proposal for a literature-based adherence score | Mediterranean diet and health status: An updated meta-analysis and a proposal for a literature-based adherence score | Yes |
| 98 | Mediterranean diet and its benefits on health and mental health: A literature review | Mediterranean diet and its benefits on health and mental health: A literature review | Yes |
| 99 | Mediterranean diet and mortality in people with cardiovascular disease: A meta-analysis of prospective cohort studies | Mediterranean diet and mortality in people with cardiovascular disease: A meta-analysis of prospective cohort studies | Yes |
| 100 | Mediterranean diet and mortality in the elderly: A prospective cohort study and a meta-analysis | Mediterranean diet and mortality in the elderly: A prospective cohort study and a meta-analysis | Yes |
| 101 | Mediterranean Diet and Musculoskeletal-Functional Outcomes in Community-Dwelling Older People: A Systematic Review and Meta-Analysis | Mediterranean Diet and Musculoskeletal-Functional Outcomes in Community-Dwelling Older People: A Systematic Review and Meta-Analysis | Yes |
| 102 | Mediterranean diet and structural neuroimaging biomarkers of Alzheimer's and cerebrovascular disease: A systematic review | Mediterranean diet and structural neuroimaging biomarkers of Alzheimer's and cerebrovascular disease: A systematic review | Yes |
| 103 | Mediterranean Diet for Primary and Secondary Prevention of Cardiovascular Disease and Mortality: An Updated Systematic Review | Mediterranean Diet for Primary and Secondary Prevention of Cardiovascular Disease and Mortality: An Updated Systematic Review | Yes |

| | | | |
|---|---|---|---|
| 104 | Mediterranean diet, cardiovascular disease and mortality in diabetes: A systematic review and meta-analysis of prospective cohort studies and randomized clinical trials | Mediterranean diet, cardiovascular disease and mortality in diabetes: A systematic review and meta-analysis of prospective cohort studies and randomized clinical trials | Yes |
| 105 | Mediterranean diet, cognitive function, and dementia: A systematic review of the evidence | Mediterranean diet, cognitive function, and dementia: A systematic review of the evidence | Yes |
| 106 | Mediterranean diet, stroke, cognitive impairment, and depression: A meta-analysis | Mediterranean diet, stroke, cognitive impairment, and depression: A meta-analysis | Yes |
| 107 | 'Mediterranean' dietary pattern for the primary prevention of cardiovascular disease | 'Mediterranean' dietary pattern for the primary prevention of cardiovascular disease | Yes |
| 108 | Mediterranean dietary pattern, inflammation and endothelial function: A systematic review and meta-analysis of intervention trials | Mediterranean dietary pattern, inflammation and endothelial function: A systematic review and meta-analysis of intervention trials | Yes |
| 109 | Mediterranean-style diet for the primary and secondary prevention of cardiovascular disease | Mediterranean-style diet for the primary and secondary prevention of cardiovascular disease | Yes |
| 110 | Meta-analysis and machine learning-augmented mixed effects cohort analysis of improved diets among 5847 medical trainees, providers and patients | Meta-analysis and machine learning-augmented mixed effects cohort analysis of improved diets among 5847 medical trainees, providers and patients | Yes |
| 111 | Meta-analysis of effect of vegetarian diet on ischemic heart disease and all-cause mortality | Meta-analysis of effect of vegetarian diet on ischemic heart disease and all-cause mortality | Yes |
| 112 | Metabolic effects of monounsaturated fatty acid-enriched diets compared with carbohydrate or polyunsaturated fatty acid-enriched diets in patients with type 2 diabetes: A systematic review and meta-analysis of randomized controlled trials | Metabolic effects of monounsaturated fatty acid-enriched diets compared with carbohydrate or polyunsaturated fatty acid-enriched diets in patients with type 2 diabetes: A systematic review and meta-analysis of randomized controlled trials | Yes |
| 113 | Monounsaturated fatty acids, olive oil and health status: A systematic review and meta-analysis of cohort studies | Monounsaturated fatty acids, olive oil and health status: A systematic review and meta-analysis of cohort studies | Yes |
| 114 | Natural history of venous thromboembolism in patients from the Mediterranean region. A systematic review | Natural history of venous thromboembolism in patients from the Mediterranean region. A systematic review | Yes |
| 115 | Nut consumption and risk of cardiovascular disease, total cancer, all-cause and cause-specific mortality: A systematic review and dose-response meta-analysis of prospective studies | Nut consumption and risk of cardiovascular disease, total cancer, all-cause and cause-specific mortality: A systematic review and dose-response meta-analysis of prospective studies | Yes |
| 116 | Nut consumption and risk of stroke | Nut consumption and risk of stroke | Yes |
| 117 | Nut consumption and risk of type 2 diabetes, cardiovascular disease, and all-cause mortality: A systematic review and meta-analysis | Nut consumption and risk of type 2 diabetes, cardiovascular disease, and all-cause mortality: A systematic review and meta-analysis | Yes |
| 118 | Nut consumption in relation to all-cause and cause-specific mortality: A meta-analysis 18 prospective studies | Nut consumption in relation to all-cause and cause-specific mortality: A meta-analysis 18 prospective studies | Yes |

| | | | |
|---|---|---|---|
| 119 | Nut consumption in relation to cardiovascular disease risk and type 2 diabetes: A systematic review and meta-analysis of prospective studies | Nut consumption in relation to cardiovascular disease risk and type 2 diabetes: A systematic review and meta-analysis of prospective studies | Yes |
| 120 | Nut intake and stroke risk: A dose-response meta-analysis of prospective cohort studies | Nut intake and stroke risk: A dose-response meta-analysis of prospective cohort studies | Yes |
| 121 | Nutritional Intake after Liver Transplant: Systematic Review and Meta-Analysis | Nutritional Intake after Liver Transplant: Systematic Review and Meta-Analysis | Yes |
| 122 | Oligella spp.: A systematic review on an uncommon urinary pathogen | Oligella spp.: A systematic review on an uncommon urinary pathogen | Yes |
| 123 | Olive oil consumption and risk of CHD and/or stroke: A meta-analysis of case-control, cohort and intervention studies | Olive oil consumption and risk of CHD and/or stroke: A meta-analysis of case-control, cohort and intervention studies | Yes |
| 124 | Omega-3 and polyunsaturated fat for prevention of depression and anxiety symptoms: Systematic review and meta-analysis of randomised trials | Omega-3 and polyunsaturated fat for prevention of depression and anxiety symptoms: Systematic review and meta-analysis of randomised trials | Yes |
| 125 | Physical activity, diet, and weight loss in patients recruited from primary care settings: An update on obesity management interventions | Physical activity, diet, and weight loss in patients recruited from primary care settings: An update on obesity management interventions | Yes |
| 126 | Polyunsaturated fatty acids for the primary and secondary prevention of cardiovascular disease | Polyunsaturated fatty acids for the primary and secondary prevention of cardiovascular disease | Yes |
| 127 | Portfolio Dietary Pattern and Cardiovascular Disease: A Systematic Review and Meta-analysis of Controlled Trials | Portfolio Dietary Pattern and Cardiovascular Disease: A Systematic Review and Meta-analysis of Controlled Trials | Yes |
| 128 | Randomised controlled trials addressing how the clinical application of information and communication technology impacts the quality of patient care—A systematic review and meta-analysis | Randomised controlled trials addressing how the clinical application of information and communication technology impacts the quality of patient care—A systematic review and meta-analysis | Yes |
| 129 | Reduction in saturated fat intake for cardiovascular disease | Reduction in saturated fat intake for cardiovascular disease | Yes |
| 129 | Reduction in saturated fat intake for cardiovascular disease | Reduction in saturated fat intake for cardiovascular disease | Yes |
| 131 | Relationship of tree nut, peanut and peanut butter intake with total and cause-specific mortality: A cohort study and meta-analysis | Relationship of tree nut, peanut and peanut butter intake with total and cause-specific mortality: A cohort study and meta-analysis | Yes |
| 132 | Research is still limited on nutrition and quality of life among older adults | Research is still limited on nutrition and quality of life among older adults | Yes |
| 133 | Resistance training and stroke: A critical analysis of different training programs | Resistance training and stroke: A critical analysis of different training programs | Yes |
| 134 | School and family-based interventions for promoting a healthy lifestyle among children and adolescents in Italy: A systematic review | School and family-based interventions for promoting a healthy lifestyle among children and adolescents in Italy: A systematic review | Yes |
| 135 | Social, economic, technological, and environmental factors affecting cardiovascular diseases: A systematic review and thematic analysis | Social, economic, technological, and environmental factors affecting cardiovascular diseases: A systematic review and thematic analysis | Yes |

| | | | |
|---|---|---|---|
| 136 | Systematic review of palm oil consumption and the risk of cardiovascular disease | Systematic review of palm oil consumption and the risk of cardiovascular disease | Yes |
| 137 | The Comparative Effects of Different Types of Oral Vitamin Supplements on Arterial Stiffness: A Network Meta-Analysis | The Comparative Effects of Different Types of Oral Vitamin Supplements on Arterial Stiffness: A Network Meta-Analysis | Yes |
| 138 | The Effect of Berry-Based Food Interventions on Markers of Cardiovascular and Metabolic Health: A Systematic Review of Randomized Controlled Trials | The Effect of Berry-Based Food Interventions on Markers of Cardiovascular and Metabolic Health: A Systematic Review of Randomized Controlled Trials | Yes |
| 139 | The Effect of Dietary Patterns on Reducing Falls and Falls Risk in Adults: A Systematic Review | The Effect of Dietary Patterns on Reducing Falls and Falls Risk in Adults: A Systematic Review | Yes |
| 140 | The effect of nut consumption on markers of inflammation and endothelial function: A systematic review and meta-analysis of randomised controlled trials | The effect of nut consumption on markers of inflammation and endothelial function: A systematic review and meta-analysis of randomised controlled trials | Yes |
| 141 | The Effect of the Mediterranean Diet on Hypertension: A Systematic Review and Meta-Analysis | The Effect of the Mediterranean Diet on Hypertension: A Systematic Review and Meta-Analysis | Yes |
| 142 | The effects of Anethum graveolens (dill) supplementation on lipid profile and glycemic control: a systematic review and meta-analysis of randomized controlled trials | The effects of Anethum graveolens (dill) supplementation on lipid profile and glycemic control: a systematic review and meta-analysis of randomized controlled trials | Yes |
| 143 | The effects of diet on weight and metabolic outcomes in patients with double diabetes: A systematic review | The effects of diet on weight and metabolic outcomes in patients with double diabetes: A systematic review | Yes |
| 144 | The effects of low-carbohydrate diets on cardiovascular risk factors: A meta-analysis | The effects of low-carbohydrate diets on cardiovascular risk factors: A meta-analysis | Yes |
| 145 | The impact of dietary factors on indices of chronic disease in older people: A systematic review | The impact of dietary factors on indices of chronic disease in older people: A systematic review | Yes |
| 146 | The impact of nutrition on the development and progression of peripheral artery disease: A systematic review | The impact of nutrition on the development and progression of peripheral artery disease: A systematic review | Yes |
| 147 | The impact of the mediterranean diet on the cognitive functioning of healthy older adults: A systematic review and meta-analysis | The impact of the mediterranean diet on the cognitive functioning of healthy older adults: A systematic review and meta-analysis | Yes |
| 148 | The prospective impact of food pricing on improving dietary consumption: A systematic review and meta-analysis | The prospective impact of food pricing on improving dietary consumption: A systematic review and meta-analysis | Yes |
| 149 | The Relationship Between Omega-3, Omega-6 and Total Polyunsaturated Fat and Musculoskeletal Health and Functional Status in Adults: A Systematic Review and Meta-analysis of RCTs | The Relationship Between Omega-3, Omega-6 and Total Polyunsaturated Fat and Musculoskeletal Health and Functional Status in Adults: A Systematic Review and Meta-analysis of RCTs | Yes |

| | | | |
|---|---|---|---|
| 150 | The short-and long-term effects of dietary patterns on cardiometabolic health in adults aged 65 years or older: A systematic review | The short-and long-term effects of dietary patterns on cardiometabolic health in adults aged 65 years or older: A systematic review | Yes |
| 151 | The Theory of Planned Behaviour and dietary patterns: A systematic review and meta-analysis | The Theory of Planned Behaviour and dietary patterns: A systematic review and meta-analysis | Yes |
| 152 | The use of psychological methodologies in cardiovascular disease interventions promoting a Mediterranean style diet: A systematic review | The use of psychological methodologies in cardiovascular disease interventions promoting a Mediterranean style diet: A systematic review | Yes |
| 153 | Total red meat intake of ⩾0.5 servings/d does not negatively influence cardiovascular disease risk factors: A systemically searched meta-analysis of randomized controlled trials | Total red meat intake of ⩾0.5 servings/d does not negatively influence cardiovascular disease risk factors: A systemically searched meta-analysis of randomized controlled trials | Yes |
| 154 | Transcriptomics and the mediterranean diet: A systematic review | Transcriptomics and the mediterranean diet: A systematic review | Yes |
| 155 | Transferability of the Mediterranean diet to non-Mediterranean countries. What is and what is not the Mediterranean diet | Transferability of the Mediterranean diet to non-Mediterranean countries. What is and what is not the Mediterranean diet | Yes |
| 156 | Transition from metabolically healthy to unhealthy overweight/obesity and risk of cardiovascular disease incidence: A systematic review and meta-analysis | Transition from metabolically healthy to unhealthy overweight/obesity and risk of cardiovascular disease incidence: A systematic review and meta-analysis | Yes |
| 157 | Tree Nut and Peanut Consumption and Risk of Cardiovascular Disease: A Systematic Review and Meta-Analysis of Randomized Controlled Trials | Tree Nut and Peanut Consumption and Risk of Cardiovascular Disease: A Systematic Review and Meta-Analysis of Randomized Controlled Trials | Yes |
| 158 | Vegan-vegetarian diets in pregnancy: Danger or panacea? A systematic narrative review | Vegan-vegetarian diets in pregnancy: Danger or panacea? A systematic narrative review | Yes |
| 159 | What's new in stroke? Phase III randomized clinical trials of 2012-2014 | What's new in stroke? Phase III randomized clinical trials of 2012-2014 | Yes |
| 160 | From Mediterranean diet to Mediterranean lifestyle: A narrative review | Diet and Healthy Patterns in the Elderly | No |
| 161 | Mediterranean diet and health outcomes: A systematic meta-review | Lifestyle changes for the treatment of nonalcoholic fatty liver disease-a 2015-19 update | No |
| 162 | Definition of the mediterranean diet: A literature review | | NA |
| 163 | Biomarkers of food intake for nuts and vegetable oils: an extensive literature search | | NA |
| 164 | Role of warburg effect in cardiovascular diseases: A potential treatment option | | NA |
| 165 | Atherogenic dyslipidemia in Latin America: Prevalence, causes and treatment. Consensus | | NA |

| | | |
|---|---|---|
| 166 | Nuts as a Part of Dietary Strategy to Improve Metabolic Biomarkers: A Narrative Review | NA |
| 167 | Non-systematic review of diet and nutritional risk factors of cardiovascular disease in obesity | NA |
| 168 | Lifestyle changes for stroke prevention | NA |
| 169 | Reviewing the cardiovascular and other health effects of olive oil: Limitations and future directions of current supplement formulations | NA |
| 170 | Lifestyle advice and interventions for cardiovascular risk reduction: A systematic review of guidelines | NA |
| 171 | Non-pharmacological management of hypertension | NA |
| 172 | Coconut oil intake and its effects on the cardiometabolic profile – A structured literature review | NA |
| 173 | Sodium–Glucose Cotransporter-2 Inhibition in Type 2 Diabetes Mellitus A Review of Large-Scale Cardiovascular Outcome Studies and Possible Mechanisms of Benefit | NA |
| 174 | Practical dietary recommendations for the prevention andmanagement of nonalcoholic fatty liver disease in adults | NA |
| 175 | Complementary and Integrative Medicine for Neurocognitive Disorders and Caregiver Health | NA |
| 176 | Oxidative stress, aging, antioxidant supplementation and their impact on human health: An overview | NA |
| 177 | Role of immune responses in the development of NAFLD-associated liver cancer and prospects for therapeutic modulation | NA |
| 178 | Olive Oil-related anti-inflammatory effects on atherosclerosis: Potential clinical implications | NA |
| 179 | A systematic review of precision nutrition and Mediterranean Diet: A personalized nutrition approaches for prevention and management of obesity related disorders | NA |
| 180 | The Influences of Macronutrients on Bone Mineral Density, Bone Turnover Markers, and Fracture Risk in Elderly People: A Review of Human Studies | NA |
| 181 | Greenhouse gases emissions from the diet and risk of death and chronic diseases in the EPIC-Spain cohort | NA |
| 182 | Diet and Men's Sexual Health | |

| | | |
|---|---|---|
| 183 | Dietary Risk Factors and Eating Behaviors in Peripheral Arterial Disease (PAD) | NA |
| 184 | Health benefits of legumes and pulses with a focus on Australian sweet lupins | NA |
| 185 | Nuts and cardio-metabolic disease: A review of meta-analyses | NA |
| 186 | Clinical implications of oxidative stress and potential role of natural antioxidants in diabetic vascular complications | NA |
| 187 | Improving the scientific rigour of nutritional recommendations for adults with type 2 diabetes: A comprehensive review of the American Diabetes Association guideline-recommended eating patterns | NA |
| 188 | Psychosocial Factors in Diabetes and Cardiovascular Risk | NA |
| 189 | Healthy diet in primary and secondary prevention of stroke | NA |
| 190 | Cashew nut allergy; immune health challenge | NA |
| 191 | Cardiovascular risk in climacteric women: focus on diet | NA |
| 192 | Economic benefit of dietetic-nutritional treatment in the multidisciplinary primary care team | NA |
| 193 | Diet and prevention of type 2 diabetes mellitus: beyond weight loss and exercise | NA |
| 194 | Determination of the level of evidence for the association between different food groups/items and dietary fiber intake and the risk of cardiovascular diseases and hypertension: An umbrella review | NA |
| 195 | Can study of the ADRB3 gene help improve weight loss programs in obese individuals? | NA |
| 196 | Universal depression prevention: An umbrella review of meta-analyses | NA |
| 197 | Replacement of saturated and trans-fatty acids in the diet v. CVD risk in the light of the most recent studies | NA |
| 198 | Saturated Fat: Part of a Healthy Diet | NA |
| 199 | Mediterranean diet and cognitive health: An update of available knowledge | NA |
| 200 | Dietary interventions to contrast the onset and progression of diabetic nephropathy: A critical survey of new data | NA |

| | | |
|---|---|---|
| 201 | The effect of on-shelf sugar labeling on beverage sales in the supermarket: a comparative interrupted time series analysis of a natural experiment | NA |
| 202 | The Role of Macronutrient Content in the Diet for Weight Management | NA |
| 203 | Impact of diet on cardiometabolic health in children and adolescents | NA |
| 204 | Development of a peer support intervention to encourage dietary behaviour change towards a Mediterranean diet in adults at high cardiovascular risk | NA |
| 205 | Dietary profiles and cardiovascular diseases | NA |
| 206 | Lipoprotein(a): Current Evidence for a Physiologic Role and the Effects of Nutraceutical Strategies | NA |
| 207 | Saturated fat: villain and bogeyman in the development of cardiovascular disease? | NA |
| 208 | Mediterranean-style diet for the primary and secondary prevention of cardiovascular disease: Summary of a Cochrane review | NA |
| 209 | Global Perspectives on the Medicinal Implications of Green Walnut and Its Benefits: A Comprehensive Review | NA |
| 210 | Influence of mediterranean diet on blood pressure | NA |
| 211 | Dietary patterns and components to prevent and treat heart failure: a comprehensive review of human studies | NA |
| 212 | Eating, diet, and nutrition for the treatment of non-alcoholic fatty liver disease | NA |
| 213 | The Mediterranean Diets' Effect on Gut Microbial Composition in Comparison with the Western Diet: A Literature Review | NA |
| 214 | Flavor chemistry of virgin olive oil: An overview | NA |
| 215 | Optimal cholesterol levels in patients in real-life. A systematic review | NA |
| 216 | The Mediterranean Diet and Cardiovascular Health: A Critical Review | NA |
| 217 | The Role of the Skin and Gut Microbiome in Psoriatic Disease | NA |
| 218 | Food for Thought or Feeding a Dogma? Diet and Coronary Artery Disease: a Clinician's Perspective | NA |
| 219 | Nitrate, the oral microbiome, and cardiovascular health: A systematic literature review of human and animal studies | NA |

| | | |
|---|---|---|
| 220 | Diet and primary prevention of stroke: Systematic review and dietary recommendations by the ad hoc Working Group of the Italian Society of Human Nutrition | NA |
| 221 | Intracellular lipid accumulation and shift during diabetes progression | NA |
| 222 | Metabolic syndrome in bipolar disorder: A review with a focus on bipolar depression | NA |
| 223 | Current meta-analysis: Omega-3 fatty acids and cardiovascular risk | NA |
| 224 | A review of the relative efficacy of dietary, nutritional supplements, lifestyle, and drug therapies in the management of hypertension | NA |
| 225 | Anti-inflammatory effects of omega 3 and omega 6 polyunsaturated fatty acids in cardiovascular disease and metabolic syndrome | NA |
| 226 | Obesity and cardiometabolic defects in heart failure pathology | NA |
| 227 | Healthy diet: Health impact, prevalence, correlates, and interventions | NA |
| 228 | Improvement of myocardial infarction risk prediction via inflammation-associated metabolite biomarkers | NA |
| 229 | Etiologic effects and optimal intakes of foods and nutrients for risk of cardiovascular diseases and diabetes: Systematic reviews and meta-analyses from the nutrition and chronic diseases expert group (NutriCoDE) | NA |
| 230 | Cardiovascular risk protection from the Mediterranean diet and olive oil. A transcriptomic update in humans | NA |
| 231 | Food groups and risk of chronic disease: A protocol for a systematic review and network meta-analysis of cohort studies | NA |
| 232 | Impact of nonoptimal intakes of saturated, polyunsaturated, and trans fat on global burdens of coronary heart disease | NA |
| 233 | Dietary factors associated with stroke: A literature review | NA |
| 234 | Doubly blind: A systematic review of gender in randomised controlled trials | NA |
| 235 | Dietary modifications for refractory chronic rhinosinusitis? Manipulating diet for the modulation of inflammation | NA |
| 236 | Effectiveness of psychological and/or educational interventions to prevent the onset of episodes of depression: A systematic review of systematic reviews and meta-analyses | NA |

| | | |
|---|---|---|
| 237 | PON1 and mediterranean diet | NA |
| 238 | The diet-heart hypothesis, obesity and diabetes | NA |
| 239 | Protective effects of dietary PUFA against chronic disease: Evidence from epidemiological studies and intervention trials | NA |
| 240 | Positive effects of the mediterranean diet in the prevention and management of cardiovascular disease: A literature review | NA |
| 241 | New findings in primary and secondary stroke prevention | NA |
| 242 | Position statement on nutrition therapy for overweight and obesity: nutrition department of the Brazilian association for the study of obesity and metabolic syndrome (ABESO—2022) | NA |
| 243 | Effect of Nuts on Markers of Inflammation and Oxidative Stress: A Narrative Review | NA |
| 244 | The pivotal role of oleuropein in the anti-diabetic action of the mediterranean diet: A concise review | NA |
| 245 | Processed meat consumption and the risk of cancer: A critical evaluation of the constraints of current evidence from epidemiological studies | NA |
| 246 | Diet as a possible influencing factor in thyroid cancer incidence: the point of view of the nutritionist | NA |
| 247 | The Anti-Inflammatory Properties of Phytochemicals and Their Effects on Epigenetic Mechanisms Involved in TLR4/NF-κB-Mediated Inflammation | NA |
| 248 | Nutrition and physical activity: An Obesity Medicine Association (OMA) Clinical Practice Statement 2022 | NA |
| 249 | Epigenetics: A New Link Between Nutrition and Health | NA |
| 250 | The attention on gender in studies on the treatment of hypertension: Was it enough? | NA |
| 251 | The role of short-chain fatty acids in the interplay between gut microbiota and diet in cardio-metabolic health | NA |
| 252 | Perspective: Design and Conduct of Human Nutrition Randomized Controlled Trials | NA |
| 253 | Dietary bioactive ingredients to modulate the gut microbiota-derived metabolite TMAO. New opportunities for functional food development | NA |
| 254 | Structure–biological activity relationships of extra-virgin olive oil phenolic compounds: Health properties and bioavailability | NA |

| | | |
|---|---|---|
| 255 | Effects of olive oil on blood pressure: Epidemiological, clinical, and mechanistic evidence | NA |
| 256 | Social marketing-based interventions to promote healthy nutrition behaviors: a systematic review protocol | NA |
| 257 | Sustainability of food systems in the mediterranean region | NA |
| 258 | Influence of the mediterranean and ketogenic diets on cognitive status and decline: A narrative review | NA |
| 259 | Mediterranean diet and oxidative stress | NA |
| 260 | Nutrition and frailty: Current knowledge | NA |
| 261 | The Mediterranean Diet and Cardiovascular Disease: Gaps in the Evidence and Research Challenges | NA |
| 262 | Lifestyle interventions and nutraceuticals: Guideline-based approach to cardiovascular disease prevention | NA |
| 263 | A comprehensive policy for reducing sugar beverages for healthy life extension | NA |
| 264 | The mediterranean diet and cardioprotection: Historical overview and current research | NA |
| 265 | Emerging Complementary and Integrative Therapies for Geriatric Mental Health | NA |
| 266 | Individual Evidence-Based Medical Nutrition in Medical In-Patients: Where do we Stand Today? | NA |
| 267 | Dietary Approaches to Stop Hypertension (DASH) for the primary and secondary prevention of cardiovascular diseases | NA |
| 268 | Frailty of the elderly: An overview of the role of nutrition | NA |
| 269 | Weight reduction and cardiovascular benefits: Protocol for a systematic review and meta-analysis | NA |
| 270 | The Mediterranean Diet: Lost in Translation | NA |
| 271 | Tackling frailty and functional decline: Background of the action group A3 of the European innovation partnership for active and healthy ageing | NA |
| 272 | Advancing Nutrition Education, Training, and Research for Medical Students, Residents, Fellows, Attending Physicians, and Other Clinicians: Building Competencies and Interdisciplinary Coordination | NA |

| | | |
|---|---|---|
| 273 | Dietary Patterns and Cardiovascular Disease Prevention among Patients with Diabetes | NA |
| 274 | A review of the relationship between pulse consumption and reduction of cardiovascular disease risk factors | NA |
| 275 | Mediterranean-style diet in pregnant women with metabolic risk factors (ESTEEM): A pragmatic multicentre randomised trial | NA |
| 276 | Reducing the global burden of cardiovascular disease, part 1: The epidemiology and risk factors | NA |
| 277 | Serum low-density lipoprotein as a dietary responsive biomarker of cardiovascular disease risk: Consensus and confusion | NA |
| 278 | Utilizing nutritional genomics to tailor diets for the prevention of cardiovascular disease: a guide for upcoming studies and implementations | NA |
| 279 | Dietary inflammatory index and all-cause mortality in large cohorts: The SUN and PREDIMED studies | NA |
| 280 | Are diabetes guidelines truly evidence based? | NA |
| 281 | Cardio-oncology Related to Heart Failure: Common Risk Factors Between Cancer and Cardiovascular Disease | NA |
| 282 | Estimating Longitudinal Risks and Benefits from Cardiovascular Preventive Therapies among Medicare Patients: The Million Hearts Longitudinal ASCVD Risk Assessment Tool: A Special Report from the American Heart Association and American College of Cardiology | NA |
| 283 | Cardiovascular disease risk reduction in diabetes through conventional and natural approaches | NA |
| 284 | Omega-3 fatty acids and cytochrome P450-derived eicosanoids in cardiovascular diseases: Which actions and interactions modulate hemodynamics? | NA |
| 285 | Effect of linoleic acid on ischemic heart disease and its risk factors: A Mendelian randomization study | NA |
| 286 | Marginal structural models and other analyses allow multiple estimates of treatment effects in randomized clinical trials: Meta-epidemiological analysis | NA |
| 287 | Principles of Healthful Eating | NA |
| 288 | The 2015 Dutch food-based dietary guidelines | NA |

| | | |
|---|---|---|
| 289 | Cholesterol metabolism: A review of how ageing disrupts the biological mechanisms responsible for its regulation | NA |
| 290 | Methodologic quality of meta-analyses and systematic reviews on the Mediterranean diet and cardiovascular disease outcomes: A review | NA |
| 291 | Frailty of the elderly: An overview of the role of nutrition | NA |
| 292 | The Mediterranean Diet: its definition and evaluation of a priori dietary indexes in primary cardiovascular prevention | NA |
| 293 | Cardiovascular risks and benefits of moderate and heavy alcohol consumption | NA |
| 294 | Oily fish, coffee and walnuts: Dietary treatment for nonalcoholic fatty liver disease | NA |
| 295 | Dyslipidemia and Cardiovascular Disease in Women | NA |
| 296 | Diet heart controversies-Quality of fat matters | NA |
| 297 | Role of Physical Activity and Diet After Colorectal Cancer Diagnosis | NA |
| 298 | Diagnosis and management of cardiovascular risk in nonalcoholic fatty liver disease | NA |
| 299 | Polyunsaturated fatty acids and risk of melanoma: A Mendelian randomisation analysis | NA |
| 300 | Occurrence of variable levels of health-promoting fruit compounds in horn-shaped Italian sweet pepper varieties assessed by a comprehensive approach | NA |
| 301 | Mediterranean diet interventions to prevent cognitive decline-opportunities and challenges | NA |
| 302 | Hydrogen sulfide as a potent cardiovascular protective agent | NA |
| 303 | Ten years later, another view upon nutrition | NA |
| 304 | Health, not weight loss, focused programmes versus conventional weight loss programmes for cardiovascular risk factors | NA |
| 305 | Primary prevention of alzheimer's disease: Is it an attainable goal? | NA |
| 306 | Sexual Dysfunction Among Young Men: Overview of Dietary Components Associated With Erectile Dysfunction | NA |
| 307 | Nutraceuticals and dyslipidaemia: Beyond the common therapeutics | NA |
| 308 | Mediterranean Diet and Cardiovascular Risk - Are We There Yet? | NA |

| | | |
|---|---|---|
| 309 | Approach to identifying and managing atherogenic dyslipidemia: A metabolic consequence of obesity and diabetes | NA |
| 310 | Nutritional management of insulin resistance in nonalcoholic fatty liver disease (NAFLD) | NA |
| 311 | Machine Learning-Augmented Propensity Score-Adjusted Multilevel Mixed Effects Panel Analysis of Hands-On Cooking and Nutrition Education versus Traditional Curriculum for Medical Students as Preventive Cardiology: Multisite Cohort Study of 3,248 Trainees over 5 Years | NA |
| 312 | Antiplatelet properties of natural products | NA |
| 313 | Mediterranean Diet and Prevention of Chronic Diseases | NA |
| 314 | Flavonols reduce aortic atherosclerosis lesion area in apolipoprotein E deficient mice: A systematic review and meta-analysis | NA |
| 315 | The PREDIMED trial, Mediterranean diet and health outcomes: How strong is the evidence? | NA |
| 316 | Virgin Olive Oil as Frying Oil | NA |
| 317 | Reducing US cardiovascular disease burden and disparities through national and targeted dietary policies: A modelling study | NA |
| 318 | Present food shopping habits in the Spanish adult population: A cross-sectional study | NA |
| 319 | Estimating Longitudinal Risks and Benefits From Cardiovascular Preventive Therapies Among Medicare Patients: The Million Hearts Longitudinal ASCVD Risk Assessment Tool: A Special Report From the American Heart Association and American College of Cardiology | NA |
| 320 | Association between dietary factors and mortality from heart disease, stroke, and type 2 diabetes in the United States | NA |
| 321 | An overview on the role of nutrition and food groups in the prevention of cardiovascular diseases | NA |
| 322 | Review on cell models to evaluate the potential antioxidant activity of polysaccharides | NA |
| 323 | Subclinical diabetes | NA |
| 324 | Nutrition and mental diseases: Focus depressive disorders | NA |

| | | |
|---|---|---|
| 325 | Impact of dietary and metabolic risk factors on cardiovascular and diabetes mortality in South Asia: Analysis from the 2010 global burden of disease study | NA |
| 326 | Recommended Dietary Pattern to Achieve Adherence to the American Heart Association/American College of Cardiology (AHA/ACC) Guidelines: A Scientific Statement from the American Heart Association | NA |
| 327 | Associations of the MCM6-rs3754686 proxy for milk intake in Mediterranean and American populations with cardiovascular biomarkers, disease and mortality: Mendelian randomization | NA |
| 328 | Dietary guidelines for the Spanish population (SENC, December 2016); the new graphic icon of healthy nutrition | NA |
| 329 | Mediterranean Diet and cancer risk: an open issue | NA |
| 330 | Worldwide Exposures to Cardiovascular Risk Factors and Associated Health Effects: Current Knowledge and Data Gaps | NA |
| 331 | Health, not weight loss, focused programmes versus conventional weight loss programmes for cardiovascular risk factors | NA |
| 332 | Protective effects of the mediterranean diet on type 2 diabetes and metabolic syndrome | NA |
| 333 | Exclusive olive oil consumption has a protective effect on coronary artery disease; Overview of the THISEAS study | NA |
| 334 | Global, regional, and national comparative risk assessment of 79 behavioural, environmental and occupational, and metabolic risks or clusters of risks in 188 countries, 1990-2013: A systematic analysis for the Global Burden of Disease Study 2013 | NA |
| 335 | Nutritional therapy of obesity and type 2 diabetes | NA |
| 336 | Aspirin for primary prevention of cardiovascular disease and cancer. A benefit and harm analysis | NA |
| 337 | Mediterranean Diet: From a Healthy Diet to a Sustainable Dietary Pattern | NA |
| 338 | Can the Mediterranean diet slow down age-related cognitive decline? | NA |
| 339 | Dietary flavonoid intakes and CVD incidence in the Framingham Offspring Cohort | NA |
| 340 | Noncommunicable diseases: Three decades of global data show a mixture of increases and decreases in mortality rates | NA |

| | | |
|---|---|---|
| 341 | Understanding nutritional epidemiology and its role in policy | NA |
| 342 | Non-communicable diseases and adherence to mediterranean diet | NA |
| 343 | Nutrition and cardiovascular health | NA |
| 344 | MicroRNA-410 regulated lipoprotein lipase variant rs13702 is associated with stroke incidence and modulated by diet in the randomized controlled PREDIMED trial | NA |
| 345 | Overview of epidemiology and contribution of obesity to cardiovascular disease | NA |
| 346 | Dietary approaches to prevent hypertension | NA |
| 347 | The State of US health, 1990-2010: Burden of diseases, injuries, and risk factors | NA |

**Data extraction forms for meta-analyses**

The following data types were considered when designing the data extraction forms: 1) binary outcomes composed of 2 by 2 table data; 2) binary outcomes composed of effect sizes and confidence intervals; 3) binary outcomes composed of effect sizes and standard errors; 4) continuous outcomes composed of 2 by 3 table data; 5) continuous outcomes composed of effect sizes and confidence intervals; and 6) continuous outcomes composed of effect sizes and standard errors. For Cochrane reviews, the data file was downloaded automatically via the .rm5 files.

**Retracted clinical trails eligible for analysis (n = 1330)**

1. Lu T, Wang W, Wang Y. Improvement of Acupuncture Based on Smart Medical Care for Post-Stroke Hemiplegic Shoulder Pain and Upper Limb Motor Function . J Healthc Eng.2021:9940645.
2. Wang N, Zhang J, Zhao R, Zhao B, Li Y, Zhang X, Liu P. Effects of Sevoflurane versus Propofol on Endogenous Nitric Oxide Metabolism during Laparoscopic Surgery. J Healthc Eng. 2021:6691943.
3. Sun F, Liu Z, Zhang W. Clinical Acupoint Selection for the Treatment of Functional Constipation by Massage and Acupuncture Based on Smart Medical Big Data Analysis. J Healthc Eng. 2021:9930412.
4. Li Y, Zhang S, Song J, Tuo M, Sun C, Yang F. Effects of Self-Management Intervention Programs Based on the Health Belief Model and Planned Behavior Theory on Self-Management Behavior and Quality of Life in Middle-Aged Stroke Patients. Evid Based Complement Alternat Med. 2021:8911143.
5. Chen M, Wang X, Deng P. Clinical Impact of Nurses-Physicians Collaboration Intervention on the Treatment of Immune Recurrent Spontaneous Abortion with Low-Molecular-Weight Heparin. Evid Based Complement Alternat Med. 2021:9290720.
6. Wang W. Image Analysis Application of Motherwort Total Alkaloid Injection in the Treatment of Postabortion Hemorrhage. J Healthc Eng. 2022:8725030.
7. Cheng Z, Lin H, Zhou Z. Effects of Sports Functional Food on Physical Function of Athletes under Ultrasound Observation. Scanning. 2022:7769653.
8. Yan J, Li D, Liu Q, Xie Y. Effect of Yiqi Huayu Pinggan Zishen Formula Combined with Valsartan in the Treatment of Hypertension and Its Effect on MMP-9, Ang II, and MCP-1. Comput Math Methods Med. 2022:7982023.
9. Zhang W, Deng G, Hu J, Yan R, Hu J, Fan J. Effects of Carvedilol on Blood Pressure, Blood Sugar, and Blood Lipids in Elderly Patients with Refractory Hypertension. Comput Math Methods Med. 2022:7889024.
10. Tian Y, Li N, Wang W, Liu L. Preoperative Cryopreservation Promotes Digital Survival after Digit Replantation. Comput Math Methods Med.2022:2003618.
11. Mishra SM, Ravishankar PL, Pramod V, et al. Effect of Supplementation of Vitamin D in Patients with Periodontitis Evaluated before and after Nonsurgical Therapy .Biomed Res Int. 2023:9850874.
12. Bi W, Xu J, Dong Z, et al. Clinical Analysis of Surgical Treatment of Senile Intertrochanteric Fracture Based on Intelligent Knowledge of Health Care. Contrast Media Mol Imaging. 2023:9847415.
13. Liu Z, Xiang J, Luo F, Hu X, Luo P. The Study of Maslow's Hierarchy of Needs Theory in the Doctor-Nurse Integration Teaching Method on Clinical Interns.J Healthc Eng.2023:9892145.
14. Hou G, Chen Y, Zhu H, et al. Cortical Plasticity Mechanism and Efficacy Prediction of Repeated Transcranial Magnetic Stimulation in the Treatment of Depression with Continuous Short Bursts of Rapid Pulse Stimulation (cTBS) . Mediators Inflamm.2023:9804270.
15. Yi X, Xu W, Li A. The Clinical Application of Remimazolam Benzenesulfonate Combined with Esketamine Intravenous Anesthesia in Endoscopic Retrograde Cholangiopancreatography . Biomed Res Int. 2024:9758358.
16. Fox M P, Pascoe S, Huber A N, et al. Short-term outcomes from a cluster randomized evaluation of adherence clubs as part of differentiated HIV Care in South Africa. JAIDS Journal of Acquired Immune Deficiency Syndromes, 2021.
17. Boldt J, Zickmann B, Ballesteros M, et al. Influence of acute preoperative plasmapheresis on platelet function in cardiac surgery. J Cardiothorac Vasc Anesth. 1993;7(1):4-9.

18. Rezk M, Hamza H, El-Shamy ES. Luteal support with vaginal dydrogesterone increases pregnancy rate in patients with clomifene resistant polycystic ovary syndrome receiving letrozole for ovulation induction. Gynecol Endocrinol. 2019;35(3):217-219.
19. Technology A S. Clinical Efficacy of Subhypothermia in the Treatment of Neonatal Hypoxic-Ischemic Encephalopathy Combined with Myocardial Damage. 2023.: 9782530.
20. Wang D, Chen B. Construction of Sports Nutrition Dynamic Intervention Mechanism Based on the Improvement of College Students' Physical Health. Biomed Res Int. 2022:2417523.
21. Zhu N, Gu F, Hu Y, Bian W. Effects of Stress Psychological Intervention on the Cardiopulmonary Function, Negative Emotion, Self-Efficacy, and Quality of Life in Patients with Acute Respiratory Failure. Evid Based Complement Alternat Med. 2021:9359102.
22. Luo F, Qi J, Zhao L, Wang Y, Yang M. Effects of Personalized Nursing plus Dietary Nursing Management on LP-PLA2, Hcy Levels, and Quality of Life in Elderly Patients with Acute Coronary Syndrome. Evid Based Complement Alternat Med. 2022:8770996.
23. Cheng J, Dou X, Zhao N. The Effects of Sequential Ventilation Therapy on Blood Gas Indexes, Pulmonary Function Indexes, Clinical Efficacy, and Safety in Patients with Severe Cor Pulmonale. Contrast Media Mol Imaging.2022:3618592.
24. Li C, Wei J, Huang X, Duan Q, Zhang T. Effects of a Brain-Computer Interface-Operated Lower Limb Rehabilitation Robot on Motor Function Recovery in Patients with Stroke. J Healthc Eng.2021:4710044.
25. Lai X, Wang A. Clinical Study on Prevention of Irinotecan-Induced Delayed-Onset Diarrhea by Hot Ironing with Moxa Salt Packet on Tianshu and Shangjuxu. Emerg Med Int. 2022:6587884.
26. Mohammadzadeh A, Towfighi F, Jafari N. Effect of magnesium on arrhythmia incidence in patients undergoing coronary artery bypass grafting. ANZ J Surg. 2018 ;88(6):612-615.
27. Lu Q, Yu Q. Feedforward Control Combined with 4F Management on Postoperative Nursing Effects and Motor Function of Meniscus Sports Injuries: Based on a Prospective Case Analysis. Comput Math Methods Med.2022:5447509.
28. Zhang JW, Wang Y, Shan HH, Duan JY, Yu J. The Application of Focused Care Model in the Management of Hepatitis B Patients in a Tertiary Care Hospital and the Impact on Patients' Quality of Life. Comput Math Methods Med.2022:7770955.
29. Huang J, Su Y, Mao X. Analysis of the Application Effect of Multidisciplinary Team Cooperation Model in Chronic Heart Failure under WeChat Platform. Comput Intell Neurosci.2022:4051955.
30. Youssef H, Atallah MM. Unilateral ovarian drilling in polycystic ovarian syndrome: a prospective randomized study. Reprod Biomed Online. 2007;15(4):457-62.
31. Lin G, Yang D, Sui W. Clinical Effect of Open Reduction and Internal Fixation for Femoral Neck Fracture in Young Adults and Related Factors of Femoral Head Necrosis. J Environ Public Health.2022:2974830.
32. Parsanezhad ME, Alborzi S, Namavar Jahromi B. A prospective, double-blind, randomized, placebo-controlled clinical trial of bromocriptin in clomiphene-resistant patients with polycystic ovary syndrome and normal prolactin level. Arch Gynecol Obstet. 2004;269(2):125-9.
33. Yang HH, Zhang FF. Magnetic Resonance Imaging Features in Diagnosis of Breast Cancer and Evaluation of Effect of Epidermal Growth Factor Receptor-Targeted Therapy. Biomed Res Int.2022:3127058.
34. Wang J, Cui X, Zhang Y, et al. The effects of intermittent bolus paravertebral block on analgesia and recovery in open hepatectomy: a randomized, double-blinded, controlled study. BMC Surg. 2023;23(1):218.

35. Zhang X, Chi J. Clinical Study of Different Treatment Methods for Tuberculous Pleuritis Complicated with Pleural Tuberculoma. Comput Math Methods Med.2022:5666067.
36. Yu H, Zhao Y, Li Y. Analysis of Early Warning Diagnostic Indexes and Influencing Factors of Anxiety and Depression in Patients with Arrhythmia. Evid Based Complement Alternat Med.2022:2061340.
37. Li X, Zheng H, Zhou Z,et al. Clinical Effect of Mudan Granule on Peripheral Neuritis Caused by Chronic Renal Insufficiency. Comput Math Methods Med.2022:1052744.
38. Wang L, Wu D, Wu S, et al. The Effect of Narrative Nursing Intervention on Shame in Elderly Patients with Bladder Cancer after Ileal Bladder Replacement: A Cohort Study. Comput Math Methods Med.2022:4299919.
39. Zhang F, Wu S, Qu M, et al. Application of a Remotely Controlled Artificial Intelligence Analgesic Pump Device in Painless Treatment of Children. Contrast Media Mol Imaging. 2022:1013241.
40. Xu Z, Liu K, Lv J, Zhang Y. Application of CTU-Assisted Doppler Ultrasound Puncture in Nontube Percutaneous Nephrolithotomy, Its Effect on Patients' Complications, and Its Clinical Value. Biomed Res Int. 2022:7810062.
41. Serebrovska ZO, Serebrovska TV, Kholin VA, et al. Intermittent Hypoxia-Hyperoxia Training Improves Cognitive Function and Decreases Circulating Biomarkers of Alzheimer's Disease in Patients with Mild Cognitive Impairment: A Pilot Study [retracted in: Int J Mol Sci. 2024;25(9):5039.
42. El-Gharib M N, El-Hawary T M. Matched sample comparison of intramuscular versus vaginal micronized progesterone for prevention of preterm birth. J Matern Fetal Neonatal Med, 2013, 26(7): 716-9.
43. Vivas-Fernandez M, Garcia-Lopez L J, Piqueras J A, et al. Randomized controlled trial for selective preventive transdiagnostic intervention for adolescents at risk for emotional disorders. Child and adolescent psychiatry and mental health, 2023, 17(1): 77.
44. Wang X, Zhu C, Liu H, Sun L, Zhu W, Gu C. The effects of a midwife-led weight management program for pregnant women: A randomized controlled trial. Int J Nurs Stud. 2023;148:104558.
45. Jin F, Li XQ, Tan WF, et al. Effects of ultrasound-guided stellate-ganglion block on sleep and regional cerebral oxygen saturation in patients undergoing breast cancer surgery: a randomized, controlled, double-blinded trial. J Clin Monit Comput. 2024;38(1):239.
46. Maleki BH, Tartibian B. High-intensity interval training modulates male factor infertility though anti-inflammatory and antioxidative mechanisms in infertile men: A randomized controlled tria.Cytokine. 2023;168:156250.
47. Rezk M, Elshamy E, Shaheen AE,et al. Effects of a levonorgestrel intrauterine system versus a copper intrauterine device on menstrual changes and uterine artery Doppler.Int J Gynaecol Obstet. 2023;162(3):1128.
48. Seal SL, Dey A, Barman SC, et al. Randomized controlled trial of elevation of the fetal head with a fetal pillow during cesarean delivery at full cervical dilatation. Int J Gynaecol Obstet. 2023;162(3):1129.
49. Boldt J, Knothe C, Zickmann B, et al. Phosphodiesterase-inhibitors enoximone and piroximone in cardiac surgery: influence on platelet count and function. Intensive Care Med. 1992;18(8):449-54.
50. Tan WF, Wang ZL, Ma H,et al. Changes in the first postoperative night bispectral index of patients after thyroidectomy with different types of primary anesthetic management: a randomized controlled tria.J Clin Monit Comput. 2023;37(6):1645.
51. Boldt J, Müller M, Heesen M, Heyn S, Hempelmann G. Does long-term continuous administration of pentoxifylline affect platelet function in the critically ill patient? Intensive Care Med. 1996;22(7):644-50.

52. Pavelka K, Trc T, Karpas K, et al. The efficacy and safety of diacerein in the treatment of painful osteoarthritis of the knee: a randomized, multicenter, double-blind, placebo-controlled study with primary end points at two months after the end of a three-month treatment period. Arthritis Rheum. 2007;56(12):4055-64.
53. Li Y, Zhou L, Wei Q. Value of Intensive Nursing Detail Management in Intensive Care Unit Nursing. Evid Based Complement Alternat Med.2022:9115639.
54. Li X, Ge J, He L. Influence of Self-Practice Oriented Teaching plus Psychological Intervention on Blood Glucose Level and Psychological State in Patients with Type 2 Diabetes Mellitus on Insulin Therapy. Evid Based Complement Alternat Med.2022:5606697.
55. Ye Z, Wang W, Sun P. Effectiveness of Nursing Risk Management in Neonatal Asphyxia Resuscitation Care. Evid Based Complement Alternat Med. 2022:5465472.
56. Wang L, Chang R, Chen X. Impact of Intraspinal Nerve Block Anesthesia on Intrapartum Fever and the Neonate. Evid Based Complement Alternat Med. 2022:2600755.
57. Wei L, Meng Y, Zhang G, et al.Endovascular Repair of the Thoracic Aorta Combined with Drug Therapy in Acute Uncomplicated Type B Aortic Dissection. Disease Markers, 2022: 3021599.
58. Pan Z, Zhang M, Wang L. Efficacy of Laparoscopic Totally Extraperitoneal Repair for Inguinal Hernia. Dis Markers.2022:2970257.
59. Hao S, Liu Y, Yu M, Sun F, Wang D. The Efficacy of Targeted Perioperative Management for Diabetic Patients with Traumatic Calcaneal Fractures. Evid Based Complement Alternat Med.2022:1294416.
60. Li L, Xu F, Ye J. Effect of Family Participatory Nursing Model Based on WeChat Platform on Psychological Elasticity and Quality of Life of Patients with Lung Cancer. Biomed Res Int.2022:4704107.
61. Dai Y, Peng C, Li P. Observation and Nursing of Adverse Reactions in Severe Patients with Enhanced MRI. J Healthc Eng. 2022:5319179.
62. Zhu X, He X, Fan B, et al. Effect of Propofol Intravenous Anesthesia Combined with Press-Needle Therapy on Analgesic Effect during Painless Abortion. Comput Math Methods Med.2023:9858364.
63. Lv Y, Zhou Y, Qiao Y, et al. Effect of Dexmedetomidine on Cardiac Output among Parturient with Severe Preeclampsia after Cesarean Section. Comput Math Methods Med.2023:9753817.
64. Sun L, Liu C, Li Y. Effect of PARP Inhibitor Combined with Bevacizumab on Platinum-Resistant Recurrent Ovarian Epithelial Carcinoma. Comput Math Methods Med.2023:9823491.
65. Li S, Sun Y, Liu J, et al. Nursing Research on Benign Prostatic Hyperplasia Based on Continuous Nursing Care.Comput Math Methods Med.2023:9828659.
66. Zhou L, Wu Z, Jiang C, Dai S. Efficacy, Safety, and Impact on Patient Survival of PDL1/PD-1 Inhibitors versus FOLFIRINOX Regimens for Advanced Pancreatic Cancer.Comput Math Methods Med.2023:9849436.
67. Wang Y, Yan T, Mu X, Dong H, Su J. Effects of Moxibustion Combined with Ultrashort Wave on Pain and Oxidative Stress in Elderly Patients with Knee Osteoarthritis. Comput Math Methods Med.2023:9769207.
68. Liu P, Xing L. Effect of ICD/CRT-D Implantation on Adverse Events and Readmission Rate in Patients with Chronic Heart Failure (CHF).Comput Math Methods Med. 2023:9759310.
69. Wang J, Zhao X, Huang J, et al. A Comparative Study on the Suitability and Treatment Compliance of an Improved Wristband Wearing Method Compared with the Traditional Method.Comput Math Methods Med. 2023:9858567.
70. Luo L, Wang F, Wang L,et al. Clinical Efficacy and Psychological Impact of Omaha-Based Continuing Care for Prostate Cancer Patients.Comput Math Methods Med. 2023:9793821.

71. Wang L, Shi L, Wang L, et al. The Effect of Psychological Support Intervention Based on Structure-Process-Result Three-Dimensional Quality Assessment on Maternal Role Adaptation and Pelvic Floor Rehabilitation Exercise Compliance of Women in Spontaneous Labor. Comput Math Methods Med. 2023:9837402.
72. Jiang L, Yang X, Xi L, Zhang C. Nursing Effect and Prognosis Analysis of Self-Management Education Model Based on Protective Motivation Theory on Patients with Hematological Tumor after PICC Catheterization .Comput Math Methods Med. 2023:9860862.
73. Zhang X, Zhang D, Yu P, Li X. Effects of Continuous Care Combined with Evidence-Based Nursing on Mental Status and Quality of Life and Self-Care Ability in Patients with Liver from Breast Cancer: A Single-Center Randomized Controlled Study . Comput Math Methods Med. 2023:9768561.
74. Shan T, Wang P, Fang F. Effects of Low-Dose Aspirin Combined with Vitamin E on the Incidence of Intrauterine Growth Restriction and Hemorheological Indexes of Pregnant Women in Patients with Gestational Hypertension.Comput Math Methods Med.2023:9898473.
75. Dai J, Li Y. Effect of Nursing in Operating Room Combined with Intraoperative Heat Preservation Intervention on Prevention of Incision Infection and Improvement of Hemodynamics in Patients with Anterior Cruciate Ligament Injury and Reconstruction under Knee Arthroscopy.Comput Math Methods Med. 2023:9864849.
76. Hu H, Zhang A, Wang Z. Effect of CICARE Communication Mode on Disease Uncertainty, Self-Nursing Ability, and Quality of Life in Patients with Coronary Atherosclerotic Heart Disease after Percutaneous Coronary Intervention. Comput Math Methods Med. 2022:8654449.
77. Meng X, Yu Y. Effect of Rehabilitation Nursing under the Guidance of the Health Action Process Approach Model on Perioperative Nursing Effect of Artificial Hip Arthroplasty: Effect on Promoting Quality of Life and Postoperative Rehabilitation. Comput Math Methods Med. 2022:1247002.
78. Lin H, Xu A, Wu H,et al. Effect of Proprioception and Balance Training Combined with Continuous Nursing on BBS Score and HSS Score of Patients Undergoing Total Knee Arthroplasty. Comput Math Methods Med. 2023:9863489.
79. Jia Z, Chen X, Sun P, et al. The Protective Mechanism of Dexmedetomidine on Renal in Hemorrhagic Shock. Computational and Mathematical Methods in Medicine, 2022: 6394544.
80. Wang W, Li X, Ren Y.Correlation Analysis and Intervention Study on Disturbance of Lipid Metabolism and Diabetic Peripheral Neuropathy. Computational and mathematical methods in medicine, 2022: 2579692.
81. Pan J, Li H, Shi J. Clinical Application of the Classical Theory of Traditional Chinese Medicine in Diabetic Nephropathy. Computational and mathematical methods in medicine, 2022: 4066385.
82. Zhang C, Deng Q, Zhang J, et al. Effect of Laparoscopy Combined with Choledochoscope for the Treatment of Cholecystolithiasis and Choledocholithiasis. Comput Math Methods Med. 2023:9871264.
83. Hu L, Zhang X, Song Z, et al.Study on the Conduction Analysis and Blocking Intervention Scheme of Emotional Disorders between Cancer Patients and Their Families. Computational and Mathematical Methods in Medicine, 2022: 4820090.
84. Qi J, Jia F, Tian H, et al. Efficacy of Gamma Globulin Combined with Azithromycin Sequential Therapy in the Treatment of RMPP and Its Effect on Th1/Th2 Cytokine Levels. Computational and Mathematical Methods in Medicine, 2022: 5162768.
85. Doran J A, Sajjad W, Schneider M D, et al.Aminophylline and caffeine for reversal of adverse symptoms associated with regadenoson SPECT MPI. Journal of Nuclear Cardiology, 2017, 24(3): 1062-1070.
86. El Sharkwy I A, Abd El Aziz W M.Randomized controlled trial of N-acetylcysteine versus l-carnitine among women with clomiphene-citrate-resistant polycystic ovary syndrome. International Journal of Gynecology & Obstetrics, 2019, 147(1): 59-64.

87. Zhang L, Li X. Staged Nursing Intervention: The Effect of the Compliance in Liver Cancer Patients with Interventional Therapy. Journal of Oncology,2022: 7517821.
88. Gou X, Zhang X, Zheng X, et al. Effect of Hand Intensive Training on Upper Limb Function of Stroke Patients with Hemiplegia. Computational and Mathematical Methods in Medicine, 2022: 6844680.
89. Liu Y, Chen L. Comparison of Clinical Effects of Temozolomide Single Agent and Combined Doxorubicin in the Treatment of Glioma. Journal of Healthcare Engineering, 2022: 7995385.
90. Yu M, Wang X, Zhou X.Analysis of the Effect of Acupuncture and Pressing of Traditional Chinese Medicine on Recovery of Delayed Muscle Soreness in Athletes. Journal of Healthcare Engineering,2022: 7875068.
91. Chen X, Wang T, Li Q, et al.Comparison of Improved Surgical Eight-Step Handwashing Combined with ATP Fluorescence in Detecting the Infection Rate at the Site of Seven-Step Surgical Handwashing and 30-Day Orthopaedic Surgery: A Randomized Study. Scanning, 2022: 3123565.
92. Xu Y, Zhou Y, Lv F, et al. Clinical Efficacy of Single-Port Thoracoscopic Lobectomy versus Three-Port Thoracoscopic Lobectomy for Lung Cancer. Journal of oncology, 2022: 3434430.
93. Zhao Y, Zhu R, Bai J, et al.The Application of Whole-Process Case Management in Patients with Triple-Negative Breast Cancer. Journal of Oncology,2022: 1794288.
94. Ji Y, Zhang B, Zhang X, et al.The Effect of High-Quality Nursing Management on Thyroid Tumor Patients after Bipolar Coagulation. Journal of Oncology, 2022: 1035971.
95. Zhang M, Liu Y, Han L, et al. Application Value of Contrast-Enhanced Ultrasound Combined with Enhanced MR Scanning in Patients with Intrahepatic Cholangiocarcinoma. Journal of Healthcare Engineering, 2022: 6402646.
96. Lv Z, Ma K.A Prospective, Randomized, Double-Masked Controlled Clinical Trial of Postoperative Pain after Transepithelial Photorefractive Keratectomy (Trans-PRK). Journal of Healthcare Engineering,2022: 2718785.
97. He X, Dai Z, Shi P, et al.Intravenous Drip of Somatostatin Followed by Restricted Fluid Resuscitation to Treat Upper Gastrointestinal Bleeding in Patients with Liver Cirrhosis. Evidence-Based Complementary and Alternative Medicine,2021: 6548479.
98. Shi Y, Meng X.Insulin Aspart Combined with Exercise Therapy in Spleen Deficiency Type Gestational Diabetes Mellitus: The Effect on Disease Control and Pregnancy Outcomes. Evidence-Based Complementary and Alternative Medicine,2022: 3084522.
99. Liao J, Kui C, Zhou Y, et al.Effect of Programmed Comprehensive Nursing for Postoperative Delirium in Intensive Care Unit Patients. Evidence-Based Complementary and Alternative Medicine,2022: 1227659.
100. Han X, Froilan U Garma P, Quan H, et al.To Explore the Application Effect and Value of Evidence-Based Nursing in Patients with Pregnancy-Induced Hypertension Syndrome. Contrast Media & Molecular Imaging, 2022: 6476031.
101. Gu L, An H, Zhang X, et al.Clinical Application of Ultrasound Microscopy-Guided Pediatric Brachial Plexus Nerve Block Anesthesia. Contrast Media & Molecular Imaging,2022: 3383898.
102. Zhu X, Song C, Lu T, et al.Proposal and Efficacy of a Nurse-Led Pain Management Model for Neurointensive Care Based on the Precede-Proceed Model. Computational and Mathematical Methods in Medicine, 2022: 5686433.
103. Yuan Z, An H S, Yang B, et al.Clinical Effect of Side-Approach Laparoscopic Splenectomy for Neuropathic Splenomegaly. Computational and Mathematical Methods in Medicine,2022: 1224916.

104. Wu Z, Lu Q, Cheng H, et al.Effects of Different Doses of Rimazolam on Oxidative Stress Level Sedation Score and Recovery Time of Patients during Hip Replacement. BioMed Research International,2022: 6414722.
105. Shi L, Liu L, He Y, et al.Comparison of a Small Dose of Oxycodone and Sufentanil for the Prevention of Sufentanil-Induced Cough during General Anesthesia Induction: A Prospective Randomized Controlled Trial. Computational and Mathematical Methods in Medicine,2022: 7924643.
106. Elkhouly N I, Abdelaal N K, Solyman A E, et al.A new technique for uterine incision closure at the time of cesarean section: does it make a difference?. Journal of Obstetrics and Gynaecology, 2022, 42: 416-423.
107. Wang Z, Xuan Y, Shao Y, et al.Effects of Camrelizumab Combined with First-Line Chemotherapy on Serum SCC, VEGF Levels, and Adverse Reactions in Patients with Advanced Squamous Cell Carcinoma of the Lung. Computational Intelligence and Neuroscience,2022: 3137370.
108. Wang H, An X, Wang Z.Effect and Safety of ALA-PDT Combined with 1550 nm Fractional Therapy Laser in Treating Rosacea. Evidence-Based Complementary and Alternative Medicine,2022: 3335074.
109. Feng M, He B, Wang B, et al.Clinical Study of Heart Failure with Left Ventricular Ejection Fraction Regimen Treated with Entresto. Contrast Media & Molecular Imaging,2022: 4164089.
110. Ma J, Luo J, Cheng Z J, et al.Clinical Efficacy of Sanfeng Tongqiao Diwan in the Treatment of Allergic Rhinitis: A Randomized Controlled Trial. Disease Markers,2022: 2916223.
111. Fang L.Clinical Efficacy Analysis of Fast Rehabilitation Nursing on Pain Mitigation after Lumbar Discectomy and Bone Graft Fusion and Internal Fixation.Evidence-Based Complementary and Alternative Medicine, 2022: 3665919.
112. Zhou Y, Liu L, Gao L.Clinical Effect of Fuzheng Guben Decoction in the Treatment of Localized Prostate Cancer and Its Influence on Immune Function under Continuous Nursing Intervention. Contrast Media & Molecular Imaging, 2022: 3472722.
113. Wang G, Zhang P, Li M, et al.Effect of Combined Spinal-Epidural Anesthesia and Total Intravenous Anesthesia on Hemodynamics and Pregnancy Outcomes of Severe Preeclampsia Pregnant Patients Undergoing Cesarean Section. Evidence-Based Complementary and Alternative Medicine,2022: 2655858.
114. Harorani M, Noruzi Zamenjani M, Golitaleb M, et al. Effects of relaxation on self-esteem of patients with cancer: a randomized clinical trial Supportive Care in Cancer, 2020,28: 405-411.
115. Boldt J, Müller M, Heesen M, et al. The effects of pentoxifylline on circulating adhesion molecules in critically ill patients with acute renal failure treated by continuous veno-venous hemofiltration. Intensive care medicine, 1996, 22: 305-311.
116. Shi X L, Dong N, Liang Y, et al. 23G Minimally Invasive Vitrectomy Combined with Glaucoma Drainage Valve Implantation and Phacoemulsification Cataract Extraction for Neovascular Glaucoma Secondary to Proliferative Diabetic Retinopathy with Vitreous Hemorrhage. Computational and Mathematical Methods in Medicine,2022: 7393661.
117. Qu L, Yin Y, Zhao N, et al. Analysis of Intervention Effect and Satisfaction of Holistic Nursing after Oral Tumor Resection. Computational and Mathematical Methods in Medicine,2022: 3788605.
118. Wang Y.The Efficacy and Safety of Bisoprolol in the Treatment of Myocardial Infarction with Cardiac Insufficiency. Computational and Mathematical Methods in Medicine,2022: 3098726.
119. Badawy A, Baker El Nashar A, El Totongy M. Clomiphene citrate plus N-acetyl cysteine versus clomiphene citrate for augmenting ovulation in the management of unexplained infertility: a randomized double-blind controlled trial.Fertil Steril. 2023;120(2):395.

120. Badawy A, Elnashar A, El-Ashry M, et al.Gonadotropin-releasing hormone agonists for prevention of chemotherapy-induced ovarian damage: prospective randomized study. Fertility and sterility, 2009, 91(3): 694-697.
121. Badawy A, Khiary M, Ragab A, et al. Ultrasound-guided transvaginal ovarian needle drilling (UTND) for treatment of polycystic ovary syndrome: a randomized controlled trial. Fertility and sterility, 2008, 91(4): 1164-1167.
122. Zakherah M S, Kamal M M, Hamed H O. Laparoscopic ovarian drilling in polycystic ovary syndrome: efficacy of adjusted thermal dose based on ovarian volume. Fertility and sterility, 2011, 95(3): 1115.
123. Hekmatpou D, Pourandish Y, Farahani P V, et al. The effect of aromatherapy with the essential oil of orange on pain and vital signs of patients with fractured limbs admitted to the emergency ward: A randomized clinical trial. Indian journal of palliative care, 2017, 23(4): 431.
124. Li Y, Tao T, Song D, et al.Effects of Xuefu Zhuyu Granules on Patients with Stable Coronary Heart Disease: A Double-Blind, Randomized, and Placebo-Controlled Study. Oxidative Medicine and Cellular Longevity, 2021: 8877296.
125. Lu W, Pan Q, Zhou Y, et al.Development and Application of One Separation-Free Safety Tube on the Disposable Infusion Needle. Computational and Mathematical Methods in Medicine,2020: 6896517.
126. Lakshmi T C, Tiwari T, Agrawal J, et al. Comparison of the clinical performance of the i-gelTM, LMA SupremeTM, and Ambu AuraGainTM in adult patients during general anesthesia: a prospective and randomized study. Korean Journal of Anesthesiology, 2021, 75(4): 316.
127. Zuo M, Zuo N, Lin J, et al.The Effect of Nonpharmacological Integrated Care Protocols on Patients with Fatigue Undergoing Hemodialysis: A Randomized Controlled Trial. Computational And Mathematical Methods In Medicine,2022: 1047959.
128. Wang W, Liu Z, Wu Y.Acupuncture Combined with Traditional Chinese Medicine and Drug Therapy for the Treatment of Cerebral Infarction (Phlegm-Blood Stasis Syndrome) and Carotid Atherosclerotic Plaque: A Preliminary Randomized Controlled Study. Applied Bionics and Biomechanics,2022: 5143408.
129. Kosar O, Ozaksit G, Taskin MI. Luteal phase clomiphene citrate for ovulation induction in women with polycystic ovary syndrome [retracted in: Arch Gynecol Obstet. 2023;308(5):1661.
130. Boldt J, Muller M, Heyn S, et al. Influence of long-term continuous intravenous administration of pentoxifylline on endothelial-related coagulation in critically ill patients. Critical care medicine, 1996, 24(6): 940-946.
131. Boldt J, Muller M, Heesen M, et al. Influence of different volume therapies and pentoxifylline infusion on circulating soluble adhesion molecules in critically ill patients. Critical care medicine, 1996, 24(3): 385-391.
132. BOLDT J, BORMANN B V, KLING D, et al. Influence of acute normovolemic hemodilution on extravascular lung water in cardiac surgery. Critical care medicine, 1988, 16(4): 336-339.
133. Abbas AM, Abd Ellah NH, Hosny MA, et al. Self-administrated vaginal 2% lidocaine in-situ gel for pain relief during copper intrauterine device insertion in women with previous caesarean delivery only: a randomised, double-blind placebo-controlled trial. Eur J Contracept Reprod Health Care. 2023;28(5):283.
134. Shaaban OM, Saber T, Youness E,et al. Effect of a mobile phone-assisted postpartum family planning service on the use of long-acting reversible contraception: a randomised controlled trial.Eur J Contracept Reprod Health Care. 2023;28(5):282.
135. Deng X, Shi R, Zhan J, et al.Application Effect of External and Internal Elevation of Maxillary Sinus in Implant Restoration of Posterior Maxilla. Emergency Medicine International,2022: 7879633.
136. Huang T, Sang Y, Zhang J.Efficacy of Modified Nonpneumatic Transaxillary Approach in the Treatment of Thyroid Cancer and Its Effect on Immune Function and Parathyroid Function. Emergency Medicine International,2022: 3336880.

137. Guo L, Hu Y. Clinical Observation of Low-Temperature Plasma Knife Tonsil Adenoidectomy for Pediatric Snoring and Analysis of Influencing Factors. Emergency Medicine International,2022: 1691583.
138. Maher M A. Different analgesics prior to intrauterine device insertion: is there any evidence of efficacy?. The European Journal of Contraception & Reproductive Health Care, 2018, 23(2): 164-164.
139. Xiong J, Zhang Q, Li Y.Clinical Study of Neuromuscular Electrical Stimulation in the Prevention of Deep Venous Thrombosis of Lower Extremities after Anterior Cruciate Ligament Reconstruction. Journal of Healthcare Engineering,2022: 7857272.
140. Dong A, Zhang Y, Lu S, et al.Influence of Dexmedetomidine on Myocardial Injury in Patients with Simultaneous Pancreas-Kidney Transplantation. Evidence-Based Complementary and Alternative Medicine,2022: 7196449.
141. Hekmatpou D, Pourandish Y, Farahani P V, et al. The effect of aromatherapy with orange essential oil on anxiety and pain in patients with fractured limbs admitted to an emergency ward: A randomized clinical trial. Central European Journal of Nursing and Midwifery, 2017, 8(4): 717-722.
142. El Refaeey A, Selem A, Badawy A. Combined coenzyme Q10 and clomiphene citrate for ovulation induction in clomiphene-citrate-resistant polycystic ovary syndrome. Reprod Biomed Online, 2014, 29(1): 119-24.
143. Badawy A, Goda H, Ragab A. Induction of ovulation in idiopathic premature ovarian failure: a randomized double-blind trial.Reprod Biomed Online. 2023;47(5):103377.
144. Shohayeb A, El-Khayat W. Does a single endometrial biopsy regimen (S-EBR) improve ICSI outcome in patients with repeated implantation failure? A randomised controlled trial. European journal of obstetrics, gynecology, and reproductive biology, 2012, 164(2): 176-179.
145. Badawy A, Allam A, Abulatta M. Extending clomiphene treatment in clomiphene-resistant women with PCOS: a randomized controlled trial.Reprod Biomed Online. 2023;47(5):103374.
146. Badawy A, Metwally M, Fawzy M. Randomized controlled trial of three doses of letrozole for ovulation induction in patients with unexplained infertility.Reprod Biomed Online. 2023;47(5):103375.
147. Gerli S, Papaleo E, Ferrari A, et al. Randomized, double blind placebo-controlled trial: effects of myo-inositol on ovarian function and metabolic factors in women with PCOS. European Review for Medical & Pharmacological Sciences, 2007, 11(5):347-54.
148. Yao J, Liu D.Clinical Effect of Qili Qiangxin Capsule Combined with Sacubitril-Valsartan in Patients with Chronic Heart Failure. Journal of Healthcare Engineering,2022: 8598806.
149. Chen X, Li H, Wang S, et al.Effects of Quantitative Nursing Combined with Psychological Intervention in Operating Room on Stress Response, Psychological State, and Prognosis of Patients Undergoing Laparoscopic Endometrial Cancer Surgery. Computational and Mathematical Methods in Medicine,2022: 6735100.
150. Sun Y, Jiang M, Ji Y, et al. Impact of Postoperative Dexmedetomidine Infusion on Incidence of Delirium in Elderly Patients Undergoing Major Elective Noncardiac Surgery: a Randomized Clinical Trial.Drug Design, Development and Therapy, 2023, 17: 2897-2898.
151. Hussein A, Torky H, Aly R, et al. Lidocaine vs. tramadol vs. placebo wound infiltration for post-cesarean section pain relief: a randomized controlled trial. Journal of Perinatal Medicine, 2022, 50(8): 1073-1077.
152. Torky H, El-Desouky E S, Abo-Elmagd I, et al. Pre-operative tranexemic acid vs. etamsylate in reducing blood loss during elective cesarean section: randomized controlled trial. Journal of Perinatal Medicine, 2021, 49(3): 353-356.
153. Zheng Y, Xia Y, Ye W, et al.The Effect of Skin-to-Skin Contact on Postoperative Depression and Physical Recovery of Parturients after Cesarean Section in Obstetrics and Gynecology Department. Computational and Mathematical Methods in Medicine,2022: 9927805.

154. Song G, Jiang T, Wang Y, et al.Observation of the Curative Effect of Acupuncture for Tonifying Kidney and Removing Blood Stasis Combined with Radiofrequency Surgery in Patients with NSCLC and the Diagnostic Efficacy of Combined Detection of NTx, BGP, and CYFRA21-1 in the Occurrence of Bone Metastases. Contrast Media & Molecular Imaging,2022: 8157157.
155. Liu B, Wang Y, Zhang Y. Efficacy of Posterior Cervical Laminectomy and Decompression plus Lateral Mass Screw-Rod Internal Fixation in the Treatment of Multisegment Cervical Spinal Canal Stenosis and Effects on Cervical Curvature and Range of Motion Parameters. Evidence-Based Complementary and Alternative Medicine,2021: 6001877.
156. Lu Q, Wu L H, Qi L Y, et al. Effect of Comprehensive Care Based on Appropriate Chinese Medicine Techniques on Urinary Retention and Bladder Function Recovery after Total Hysterectomy in Patients with Cervical Cancer. Computational and Mathematical Methods in Medicine,2022: 7495418.
157. Li L, Chen H.Application of Intelligent Exercise Training Equipment in Clinical Nursing of Neurology Department. Contrast Media & Molecular Imaging, 2021: 8432868.
158. Yan F, Peng C. Analysis of the Effect of DPL Combined with Clarithromycin in the Therapy and Improvement of Rosacea. Contrast Media Mol Imaging.2023:9842640.
159. Zhang Q, Wan L, Chen Q, et al.Prevention of Severe Respiratory Tract Infection and Prognosis in Neurosurgical Patients with Severe Tracheotomy Based on 5E Rehabilitation Nursing Model. Computational and Mathematical Methods in Medicine,2022: 2727679.
160. Gao W, Wang J, Zhang Z, et al.Opioid-Free Labor Analgesia: Dexmedetomidine as an Adjuvant Combined with Ropivacaine. Journal of Healthcare Engineering,2022: 2235025.
161. Zhang S, Xu X, Yu M, et al. Efficacy and Safety of Minimally Invasive Transcatheter Closure of Congenital Heart Disease under the Guidance of Transesophageal Ultrasound: A Randomized Controlled Trial. Computational and Mathematical Methods in Medicine,2022: 2969979.
162. Sun Y, Li C, Yan Y, et al.Effects of Hemp Sanitary Pads on the Vaginal Microecology. Computational and Mathematical Methods in Medicine,2022: 4435722.
163. Weng B, Chen C. Effects of Bisphosphonate on Osteocyte Proliferation and Bone Formation in Patients with Diabetic Osteoporosis. Computational and Mathematical Methods in Medicine,2022: 2368564.
164. Duan X, Liu M, Wang P.Effects of the Concept of Fast-Track Surgery Combined with Empathic Nursing on Perioperative Complication Prevention and Postoperative Recovery of Gynecological Malignant Tumor Patient. Computational and Mathematical Methods in Medicine,2022: 4381297.
165. Chen B, Luo T, Cai Q, et al.Effect of Psychological Intervention-Assisted Comfort Nursing Based on PERMA Model on Stress and Psychological Changes of Patients after Breast Cancer Surgery. Computational and Mathematical Methods in Medicine,2022: 1853754.
166. Huang Y, Liang H, Yang Z, et al.Effect Evaluation of Bronchial Artery Embolization for Hemoptysis of Lung Cancer and Changes in Serum Tumor Markers and miR-34 Levels. Contrast Media & Molecular Imaging,2022: 2471039.
167. Zheng Y, Xu X, Zheng B.Clinical Observation on the Effect of Systematic Nursing Intervention on Cognitive Function, Life Activity Ability, and Quality of Life of Senile Dementia Patients. Evidence-Based Complementary and Alternative Medicine, 2021: 2839142.
168. Li Y, Yu J, Zhang M, et al.Clinical Application of Remifentanil Combined with Sevoflurane in Manual Reduction of Humeral Supracondylar Fracture in Children. Computational and Mathematical Methods in Medicine, 2022: 2410433.
169. Hu Q, Yang X, Wang W, et al. Analysis of the Effectiveness of the Nurse-Led “Outpatient-Ward-Home” Management Model in Chronic Kidney Patients. Evidence-Based Complementary and Alternative Medicine, 2022: 4229436.

170. Li J, Gu M, Jiang Y. Analysis of Influencing Factors of Medication Compliance in Patients with Recurrent Vertebral Fractures after Percutaneous Kyphoplasty and the Role of Family-Centered Education Intervention. Evidence-Based Complementary and Alternative Medicine,2021: 3974674.
171. Luo S, Xie Y, He S, et al. A Study on the Effect of Nursing Intervention Based on Health Behavior Change Integration Theory on Patients with Limb Fracture and Its Effect on Limb Function and Self-Efficacy. Computational and Mathematical Methods in Medicine, 2022: 1621210.
172. Saad H, Maged AM, Meshaal H,et al. Delayed versus early pushing during the second stage of labour in primigravidas under epidural anaesthesia with occipitoposterior malposition: a randomised controlled study .J Obstet Gynaecol. 2023;43(2):2259731.
173. Zhu Z, Wu Q, Hu G, et al. Efficacy of Ginkgo biloba Extract Combined with Hormones in the Treatment of Sudden Deafness and Its Effect on the Reactivity of Peripheral Blood T Cell Subsets . Comput Math Methods Med.2023:9839531.
174. Du H, Fu H, Yu J, Cheng Z, Zhang Y. Efficacy of Buqi Huoxue Decoction Combined with Cardiac Rehabilitation Nursing after Coronary Intervention in Patients with Acute ST-Segment Elevation Myocardial Infarction and Its Influence on Prognosis.J Healthc Eng.2022:4008966.
175. Li P, He L. Clinical Treatment Analysis and Imaging Study of Patients with Acute Angina in Cardiovascular Medicine. Journal of Healthcare Engineering, 2021: 1458621.
176. Shen L. Effects of Pelvic Floor Muscle Massage on the Pregnancy Outcome of Frozen Embryo Transfer in Patients with Thin Endometrium. Computational and mathematical methods in medicine,2022: 2803363.
177. Ismail A M, Hamed A H, Saso S, et al.Adding l-carnitine to clomiphene resistant PCOS women improves the quality of ovulation and the pregnancy rate. A randomized clinical trial. European Journal of Obstetrics and Gynecology and Reproductive Biology, 2014, 180: 148-152.
178. Abdelbasset WK, Elsayed SH, Nambi G, et al. Effect of Moderate-Intensity Aerobic Exercise on Hepatic Fat Content and Visceral Lipids in Hepatic Patients with Diabesity: A Single-Blinded Randomised Controlled Trial . Evid Based Complement Alternat Med.2023:9829387.
179. Yu XR, Huang ST, Xu N, et al. The effect of early oral stimulation with breast milk on the feeding behavior of infants after congenital cardiac surgery.J Cardiothorac Surg. 2020;15(1):309.
180. Rezk M, Emarh M, Masood A. Methyldopa versus labetalol or no medication for treatment of mild and moderate chronic hypertension during pregnancy: a randomized clinical trial. HYPERTENSION IN PREGNANCY, 2022, 41(3-4): 206-206.
181. Maged A M, Fawzi T, Shalaby M A, et al. A randomized controlled trial of the safety and efficacy of preoperative rectal misoprostol for prevention of intraoperative and postoperative blood loss at elective cesarean delivery. International Journal of Gynecology & Obstetrics, 2019, 147(1): 102-107.
182. Nasri K, Akrami S, Rahimi M, et al. The effects of vitamin D and evening primrose oil co-supplementation on lipid profiles and biomarkers of oxidative stress in vitamin D-deficient women with polycystic ovary syndrome: A randomized, double-blind, placebo-controlled trial. Endocr Res. 2018;43(1):1-10.
183. El-Tahan MR, Warda OM, Yasseen AM, et al. A randomized study of the effects of preoperative ketorolac on general anaesthesia for caesarean section. Int J Obstet Anesth. 2007;16(3):214-20.
184. Kamel H H. Role of phyto-oestrogens in ovulation induction in women with polycystic ovarian syndrome. European Journal of Obstetrics, Gynecology, and Reproductive Biology, 2013, 168(1): 60-63.
185. Wafa A, El Rakhawy M. Combined metformin and clomiphene citrate versus highly purified FSH for ovulation induction in clomiphene-resistant PCOS women: a randomised controlled trial. Gynecological endocrinology: the official journal of the International Society of Gynecological Endocrinology, 2010, 27(3): 190-196.

186. Bazeed M, Abd Elaal I. Minimal stimulation or clomiphene citrate as first-line therapy in women with polycystic ovary syndrome: a randomized controlled trial. Gynecological Endocrinology: the Official Journal of the International Society of Gynecological Endocrinology, 2011, 28(2): 87-90.
187. Hosny TA, Azzam AZ, Said MA, Hammad BS. Comparing delayed cord clamping and umbilical cord milking during elective cesarean section for the neonatal outcome. Eur J Obstet Gynecol Reprod Biol X. 2023; 26;18:100200.
188. Ali MK, Ramadan AK, Abu-Elhassan AM, Sobh AMA. Ultrasound-guided versus uterine sound-sparing approach during copper intrauterine device insertion: a randomised clinical trial. Eur J Contracept Reprod Health Care. 2022 ;27(1):3-8.
189. Hajizadeh Maleki B, Tartibian B. Moderate aerobic exercise training for improving reproductive function in infertile patients: A randomized controlled trial. Cytokine. 2017;92:55-67.
190. Hajizadeh Maleki B, Tartibian B, Mooren FC,et al. Low-to-moderate intensity aerobic exercise training modulates irritable bowel syndrome through antioxidative and inflammatory mechanisms in women: Results of a randomized controlled trial. Cytokine. 2018;102:18-25.
191. Nabhan A F, Rabie N H. Isosorbide mononitrate versus alendronate for postmenopausal osteoporosis. International Journal of Gynecology & Obstetrics, 2008, 103(3): 213-216.
192. Ali M K, Ahmed S E, Sayed G H, et al.Effect of adjunctive vaginal progesterone after McDonald cerclage on the rate of second-trimester abortion in singleton pregnancy: a randomized controlled trial. International Journal of Gynecology & Obstetrics, 2020, 149(3): 370-376.
193. Polat R, Peker K, Baran I, et al. Comparison between dexmedetomidine and remifentanil infusion in emergence agitation during recovery after nasal surgery: A randomized double-blind trial. Der Anaesthesist, 2015, 64(10): 740-746.
194. Yuan X, Xu F, Zhu S L, et al.Clinical Significance of Protective Motivation Intervention Nursing on Functional Recovery of Patients after Hip Arthroplasty. BioMed Research International,2022: 4219131.
195. El-Morsy G Z, El-Deeb A. The outcome of thoracic epidural anesthesia in elderly patients undergoing coronary artery bypass graft surgery. Saudi journal of anaesthesia, 2012, 6(1): 16-21.
196. Sweed M, El-Said M, Abou-Gamrah A, et al.Comparison between 200, 400 and 600 microgram rectal misoprostol before cesarian section: A randomized clinical trial. Journal of Obstetrics and Gynaecology Research, 2019, 45(3): 585-591.
197. Uyar B S, Polat R, Bolat M, et al. Which is good for pre-operative anxiety? Midazolam, video games or teaching with cartoons: a randomised trial. European Journal of Anaesthesiology| EJA, 2021, 38(7): 744-750.
198. Hao J, Shen Z.The Efficacy and Safety of Oral Nutrient Solution Supplementation in Patients with Esophageal Cancer under Nutritional, Psychological, Physical, and Prognostic Survival Differences. BioMed Research International,2023: 5055869.
199. Wang H, Gao X, Chen N.Psychological Nursing Effect of Patients with Gynecological Malignant Tumor. BioMed Research International,2022: 1569656.
200. Liu Q, Wang H F, Xing J H. Efficacy of Mesotympanum Injection and Posterior Auricular Injection in Sudden Hearing Loss of Diabetes Patients. BioMed Research International,2022: 8494868.
201. Cao W, Zhang B, Liu Y.Efficacy and Safety of rCCK96-104PE38 Targeted Drug in the General Surgical Treatment of Colon Cancer. BioMed Research International,2022: 7145606.
202. Chen J, Wang J, Duan X, et al.Clinical Observation of General Anesthesia Combined with Spinal Anesthesia in Elderly Patients with Chronic Obstructive Pulmonary Disease. BioMed Research International, 2022: 9160145.

203. Sato Y, Honda Y, Iwamoto J, et al. Amelioration by mecobalamin of subclinical carpal tunnel syndrome involving unaffected limbs in stroke patients. Journal of the neurological sciences, 2005, 231(1-2): 13-18.
204. Sato Y, Kaji M, Kondo I, et al. Hyperhomocysteinemia in Japanese patients with convalescent stage ischemic stroke: effect of combined therapy with folic acid and mecobalamine. Journal of the neurological sciences,202(1-2): 65-68.
205. Huang J, Hong W, Yang Z, Ding J, Ren Y. Efficacy of pramipexole combined with levodopa for Parkinson's disease treatment and their effects on QOL and serum TNF-α levels. J Int Med Res. 2020;48(7):300060520922449.
206. Zhong X, Shan A, Xu J,et al. Hyperbaric oxygen for severe traumatic brain injury: a randomized trial. J Int Med Res. 2020;48(10):300060520939824.
207. Mohamed Amine M, Selma M, Adel S, et al. 2-Day versus C-reactive protein guided antibiotherapy with levofloxacin in acute COPD exacerbation: A randomized controlled trial. Plos one, 2021, 16(5): e0251716.
208. Wang J, Wang C, Wu X, et al.Effect of Hyperbaric Oxygen Therapy on Sleep Quality, Drug Dosage, and Nerve Function in Patients with Sleep Disorders after Ischemic Cerebral Stroke. Emergency Medicine International,2022: 8307865.
209. Jia R, Liu N, Zhu Y, Li Q. Curative Effect of Prebiotics/Probiotics Preparations Combined with Zoledronic Acid + Calcitriol Regimen on Patients with Primary Osteoporosis and Their Influences on Bone Metabolism Markers. Emerg Med Int.2022:3293362.
210. Tang Y, Zhou Q.Changes in Serum CRP and PCT Levels in Patients with Acute Simple Lower Urinary Tract Infection and Evaluation of the Efficacy of Treatment with Shuangdong Capsules. Emergency Medicine International,2022: 9750237.
211. Hou J, Zhang R.Clinical Analysis on the Effects of Tandospirone Citrate Assisted by Drawing Therapy on Medication Compliance and Sleep Quality in Patients with Anxiety Disorders. Emergency medicine international,2022: 9295627.
212. Wei N, Du Y, Chen S.Application of Doctor-Nurse-Patient Co-Decision-Making Nursing Intervention Based on Evidence-Based Problems in the Rehabilitation of Acute Ankle Lateral Collateral Ligament Injury. Emergency Medicine International,2022: 2363230.
213. Liao Y, Jiang J, Luo J, et al. A Study on the Impact of Perioperative Pain Care Management on Pain, Comfort, and Defecation of Patients in Anorectal Surgery. Emerg Med Int.2022:9885540.
214. Badawy A, Elnashar A, Totongy M. Clomiphene citrate or aromatase inhibitors combined with gonadotropins for superovulation in women undergoing intrauterine insemination: a prospective randomised trial. J Obstet Gynaecol. 2010;30(6):617-21.
215. Ibrahim Z M, Sayed Ahmed W A.Sublingual misoprostol prior to insertion of a T380A intrauterine device in women with no previous vaginal delivery. The European Journal of Contraception & Reproductive Health Care, 2013, 18(4): 300-308.
216. Said E M, Abdulaziz B A, El Kassas M, et al. High success rates for the use of sofosbuvir/ombitasvir/paritaprevir/ritonavir+ ribavirin and sofosbuvir/simeprevir/daclatasvir+ ribavirin in retreatment of chronic hepatitis C infection after unsuccessful sofosbuvir/daclatasvir therapy: a real-life experience. Archives of Virology, 2020, 165: 1633-1639.
217. Jørgensen S F, Macpherson M E, Bjørnetrø T, et al.Rifaximin alters gut microbiota profile, but does not affect systemic inflammation-a randomized controlled trial in common variable immunodeficiency. ScIentIfIc RepoRtS, 2019, 9(1): 167.
218. Dawood A S, Dawood A S, Shazly S A, et al.A randomized controlled study comparing two uterine sparing techniques in conservative management of placenta accreta spectrum. International Journal of Gynecology & Obstetrics, 2024, 165(1): 1-8.
219. Abdelshafy A, Awwad H, Abo-Gamra A, et al. Sublingual vs vaginal misoprostol for completion of first trimester missed abortion: a randomised controlled trial. Eur J Contracept Reprod Health Care. 2019;24(2):134-139.

220. Alanwar A, Abbas AM, Hussain SH, et al. Oral micronised flavonoids versus tranexamic acid for treatment of heavy menstrual bleeding secondary to copper IUD use: a randomised double-blind clinical trial. Eur J Contracept Reprod Health Care. 2018;23(5):365-370.
221. Yang Q, Li G, Wu X,et al. A novel therapeutic strategy of combined camrelizumab and apatinib for the treatment of advanced hepatocellular carcinoma. Front Oncol. 2023;13:1136366.
222. Monticone M, Ferrante S, Rocca B, et al. Effect of a Long-lasting Multidisciplinary Program on Disability and Fear-Avoidance Behaviors in Patients With Chronic Low Back Pain Results of a Randomized Controlled Trial. The Clinical journal of pain, 2013, 29(11): 929-938.
223. He N, Shi H, Hu Y, et al. Effectiveness of Intraoperative Dexmedetomidine on Early Postoperative Cognitive Dysfunction in Elderly Patients Undergoing Video-Assisted Thoracoscopic Lobectomy: A Randomized Controlled Trial. 2020.
224. Hsieh R L, Lee W C. No difference between one-shot co-injection of corticosteroids and hyaluronic acid in a three-injection regimen for knee osteoarthritis: a randomized, double-blind trial. Clinical Orthopaedics and Related Research, 2022: 10.1097.
225. Salimian J, Ahmadi A, Amani J, et al.Safety and immunogenicity of a recombinant receptor-binding domain-based protein subunit vaccine (Noora vaccine™) against COVID-19 in adults: A randomized, double-blind, placebo-controlled, Phase 1 trial. Journal of Medical Virology, 2023, 95(2):10.
226. Abdallah M S, Ramadan A N, Omara-Reda H, et al.Double-blind, randomized, placebo-controlled pilot study of the phosphodiesterase-3 inhibitor cilostazol as an adjunctive to antidepressants in patients with major depressive disorder. CNS neuroscience & therapeutics, 2021, 27(12): 1540-1548.
227. Abdallah M S, Mosalam E M, Hassan A, et al.Pentoxifylline as an adjunctive in treatment of negative symptoms in chronic schizophrenia: A double-blind, randomized, placebo-controlled trial. CNS Neuroscience & Therapeutics, 2023, 29(1): 354-364.
228. Sweed M S, El-Saied M M, Abou-Gamrah A E, et al. Rectal vs. sublingual misoprostol before cesarean section: double-blind, three-arm, randomized clinical trial. Archives of gynecology and obstetrics, 2018, 298: 1115-1122.
229. Shi H, Du X, Wu F, et al. Dexmedetomidine improves early postoperative neurocognitive disorder in elderly male patients undergoing thoracoscopic lobectomy. Experimental and Therapeutic Medicine, 2020, 20(4): 3868-3877.
230. Ghofrani H A, Simonneau G, D'Armini A M, et al. Macitentan for the treatment of inoperable chronic thromboembolic pulmonary hypertension (MERIT-1): results from the multicentre, phase 2, randomised, double-blind, placebo-controlled study. The Lancet Respiratory Medicine, 2024, 12(4): e21-e30.
231. Maged AM, Sorour EH, ElSadek MM, et al. A randomized controlled study of the effect of hyoscine butylbromide on duration of labor in primigravida women with prolonged labor. Arch Gynecol Obstet. 2021;304(6):1513-1518.
232. Lin HX, Liu Z, Hajek P, et al. Efficacy of Electronic Cigarettes vs Varenicline and Nicotine Chewing Gum as an Aid to Stop Smoking: A Randomized Clinical Trial. JAMA Intern Med. 2024;184(3):291-299.
233. Boldt J, Müller M, Uphus D, et al. Cardiorespiratory changes in patients undergoing pulmonary resection using different anesthetic management techniques. Journal of cardiothoracic and vascular anesthesia, 1996, 10(7): 854-859.
234. Boldt J, Schindler E, Wollbrück M, et al. Cardiorespiratory response of intravenous angiotensin-converting enzyme inhibitor enalaprilat in hypertensive cardiac surgery patients. Journal of cardiothoracic and vascular anesthesia, 1995, 9(1): 44-49.
235. Menges T, Wagner R M, Welters I, et al. The role of the protein C-thrombomodulin system and fibrinolysis during cardiovascular surgery: influence of acute preoperative plasmapheresis. Journal of cardiothoracic and vascular anesthesia, 1996, 10(4): 482-489.
236. Afifi A N, Taymour M A, El-Khayat W M.Isosorbide mononitrate for cervical ripening in induction of labor for pregnant women with PROM at or post term. International Journal of Gynecology & Obstetrics, 2021, 155(3): 512-517.

237. Jamilian H. Comparison of myo-inositol and metformin on mental health parameters and biomarkers of oxidative stress in women with polycystic ovary syndrome: a randomized, double-blind, placebo-controlled trial. JOURNAL OF PSYCHOSOMATIC OBSTETRICS & GYNECOLOGY, 2020, 41(4): II.
238. Maged A M, Rashwan H, AbdelAziz S, et al.Randomized controlled trial of the effect of endometrial injury on implantation and clinical pregnancy rates during the first ICSI cycle. International Journal of Gynecology & Obstetrics, 2018, 140(2): 211-216.
239. Giacomello L, Bordignon S, Salm D, et al.Effects of the application of a food processing-based classification system in obese women: A randomized controlled pilot study. Nutrition and health, 2023: 2601060231153947.
240. Shoab A Y, Maged A M, Ramadan W, et al.The value of endocervical and endometrial lidocaine flushing before office hysteroscopy: A randomized controlled trial. International Journal of Gynecology & Obstetrics, 2020, 148(1): 113-117.
241. Rezk M, Shaheen A E, Saif El-Nasr I. Clomiphene citrate combined with metformin versus letrozole for induction of ovulation in clomiphene-resistant polycystic ovary syndrome: a randomized clinical trial. Gynecological Endocrinology, 2018, 34(4): 298-300.
242. Sharif F, Ahmad A, Gilani S A. Effectiveness of ultrasound guided dry needling in management of jumper's knee: a randomized controlled trial. Scientific Reports, 2023, 13(1):4736.
243. Elshafie A H, Elsawah H K, Hammad M, et al. Ivermectin role in COVID-19 treatment (IRICT): single-center, adaptive, randomized, double-blind, placebo-controlled, clinical trial. Expert Review of Anti-infective Therapy, 2022, 20(10): 1341-1350.
244. Boldt J, Lehmann A, Römpert R, et al. Volume therapy with a new hydroxyethyl starch solution in cardiac surgical patients before cardiopulmonary bypass. Journal of cardiothoracic and vascular anesthesia, 2000, 14(3): 264-268.
245. Boldt J, Schindler E, Knothe C, et al. Does aprotinin influence endothelial-associated coagulation in cardiac surgery?. Journal of cardiothoracic and vascular anesthesia, 1994, 8(5): 527-531.
246. Boldt J, Osmer C, Linke L C, et al. Hypothermic versus normothermic cardiopulmonary bypass: influence on circulating adhesion molecules. Journal of cardiothoracic and vascular anesthesia, 1996, 10(3): 342-347.
247. Zickmann B, Hofmann H C, Pottkämper C, et al. Changes in heart rate variability during induction of anesthesia with fentanyl and midazolam. Journal of cardiothoracic and vascular anesthesia, 1996, 10(5): 609-613.
248. Boldt J, Thaler E, Lehmann A, et al. Pain management in cardiac surgery patients: comparison between standard therapy and patient-controlled analgesia regimen. Journal of cardiothoracic and vascular anesthesia, 1998, 12(6): 654-658.
249. Mora-Plazas M, Aida Higgins I C, Gomez L F, et al. Impact of nutrient warning labels on choice of ultra-processed food and drinks high in sugar, sodium, and saturated fat in Colombia: A randomized controlled trial. PloS one, 2022, 17(2): e0263324.
250. El-Sayed M A, Saleh S A A, Maher M A, et al.Utero-placental perfusion Doppler indices in growth restricted fetuses: effect of sildenafil citrate. The Journal of Maternal-Fetal & Neonatal Medicine, 2018, 31(8): 1045-1050.
251. Ahmadi S, Jamilian M, Karamali M, et al. Probiotic supplementation and the effects on weight loss, glycaemia and lipid profiles in women with polycystic ovary syndrome: a randomized, double-blind, placebo-controlled trial. Human Fertility, 2017, 20(4): 254-261.
252. Cetingoz E, Cam C, Sakallı M, et al. Progesterone effects on preterm birth in high-risk pregnancies: a randomized placebo-controlled trial. Archives of gynecology and obstetrics, 2011, 283: 423-429.
253. Monticone M, Ambrosini E, Ferrante S, et al. Regent Suit'training improves recovery of motor and daily living activities in subjects with subacute stroke: a randomized controlled trial. Clinical rehabilitation, 2013, 27(9): 792.

254. Monticone M, Ambrosini E, Brunati R, et al. How balance task-specific training contributes to improving physical function in older subjects undergoing rehabilitation following hip fracture: a randomized controlled trial. Clinical Rehabilitation, 2018, 32(3): 340-351.
255. Monticone M, Ambrosini E, Rocca B, et al. Task-oriented exercises and early full weight-bearing contribute to improving disability after total hip replacement: a randomized controlled trial. Clinical rehabilitation, 2014, 28(7): 658-668.
256. Cetin C, Tanoglu F B, Hanligil E, et al. Carbetocin versus Oxytocin with or without Tranexamic Acid for Prophylactic Prevention of Postpartum Hemorrhage after a Vaginal Delivery: A Randomized Clinical Trial. Gynecologic & Obstetric Investigation, 2023;88(6):366-374.
257. Torky H, El-Desouky E S, El-Baz A, et al. Effect of intra uterine granulocyte colony stimulating factor vs. human chorionic gonadotropin at ovum pick up day on pregnancy rate in IVF/ICSI cases with recurrent implantation failure. JBRA assisted reproduction, 2022, 26(2): 274.
258. Shehata M M A, Maged A M, Kotb A, et al. Whole-body vibration versus supervised aerobic exercise on hormonal parameters and inflammatory status in women with premenstrual syndrome: A randomized controlled trial. International Journal of Gynecology & Obstetrics, 2023, 162(2): 493-501.
259. Ragab A, Mesbah Y. To do or not to do emergency cervical cerclage (a rescue stitch) at 24–28 weeks gestation in addition to progesterone for patients coming early in labor? A prospective randomized trial for efficacy and safety. Archives of gynecology and obstetrics, 2015, 292: 1255-1260.
260. Yildiz G, Perdecioglu G R G, Yuruk D, et al. Comparison of the efficacy of genicular nerve phenol neurolysis and radiofrequency ablation for pain management in patients with knee osteoarthritis. The Korean Journal of Pain, 2023, 36(4): 450-457.
261. Abdou A M, Eldesouky E, Farag E, et al.Oxytocin versus a combination of tranexamic acid and ethamsylate in reducing intraoperative bleeding during abdominal myomectomy: a randomized clinical trial. BMC Women's Health, 2023, 23(1): 398.
262. Boldt J, Kling D, Dieterich H A, et al. The new phosphodiesterase inhibitor enoximone in patients following cardiac surgery—pharmacokinetics and influence on parameters of coagulation. Intensive care medicine, 1990, 16: 54-59.
263. Liu L, Zhou J, Xiao H, et al. The Effect of Routine Management Combined with Case Management Model on Social Support, Self-Efficacy, Self-Management Ability, and Psychological Flexibility of AIDS Patients. Computational and Mathematical Methods in Medicine, 2022: 9213877.
264. Wang J. Study on BCVA, CMT, CME, Curative Effect, and Prognostic Value of DR Sufferers Based on Surgical Therapy Combined with VEGF Therapy at Different Times. Contrast Media Mol Imaging.2022:1415659.
265. Badawy A, Mosbah A, Tharwat A, Eid M. Extended letrozole therapy for ovulation induction in clomiphene-resistant women with polycystic ovary syndrome: a novel protocol. Fertil Steril. 2009;92(1):236-9.
266. Badawy A, Inany H, Mosbah A, et al.Luteal phase clomiphene citrate for ovulation induction in women with polycystic ovary syndrome: a novel protocol. Fertility and sterility, 2009, 91(3): 838-841.
267. Badawy A, Aal I A, Abulatta M.Clomiphene citrate or anastrozole for ovulation induction in women with polycystic ovary syndrome? A prospective controlled trial. Fertility and sterility, 2009, 92(3): 860-863.
268. Wang L, Tang X.Implications of Ezetimibe in Combination with Low-to Moderate-Intensity Atorvastatin Adjuvant Aspirin Therapy for Cerebrovascular Disease. Computational and Mathematical Methods in Medicine,2022: 3369226.
269. Zhao Y, Liu W, Wang Z, et al. The Value of CBL-Based Teaching Mode in Training Medical Students' Achievement Rate, Practical Ability, and Psychological Quality. Contrast Media & Molecular Imaging,2022: 2121463.
270. He Z, Du J, Xue X, et al.Effects of Dexmedetomidine on Hemodynamics and Anesthesia Effect of Different Doses of General Anesthesia in Patients Undergoing Hepatobiliary Surgery. Contrast Media & Molecular Imaging, 2022: 2311869.

271. Khalili L, Alipour B, Asghari Jafarabadi M, et al. Probiotic assisted weight management as a main factor for glycemic control in patients with type 2 diabetes: a randomized controlled trial. Diabetology & metabolic syndrome, 2019, 11(1): 1-9.
272. Cheng X, Wu S, Wang W.Efficacy of Otomicroscopy Combined with Otoendoscopy Double-Lens Technology-Assisted Tympanic Membrane Repair on Elderly Patients with Chronic Suppurative Otitis Media. Evidence-Based Complementary and Alternative Medicine, 2021: 5164907.
273. Zhang Q, Zhang Z, Wang B, et al. Effects of Dexmedetomidine on Postoperative Pain and Recovery Time in Obese Patients. Disease Markers,2022: 9715704.
274. Peng Z, Niu Z, Zhang R, et al. Antimicrobial Step-Down Therapy versus Conventional Antimicrobial Therapy in the Treatment of Patients with Sepsis. Dis Markers.2022:3117805.
275. Deng W. Effects of Vibration Training on Weight Loss and Heart Rate Variability in the Obese Female College Students. Biomed Res Int.2022:1041688.
276. Zhang H, Liu Z. Effects of Dapagliflozin in Combination with Metoprolol Sustained-Release Tablets on Prognosis and Cardiac Function in Patients with Acute Myocardial Infarction after PCI. Comput Math Methods Med.2022:5734876.
277. Wang R, Yu J, Yan Z, et al. Cluster-Based Immunotherapy for Patients with Recurrent Abortion Caused by Antiphospholipid Syndrome. J Healthc Eng.2021:4581900.
278. Zhang Y, Lu Q, Li N, Lu Y. Effect of Intensive Psychological Nursing Intervention on HAMD and SF-36 Scores in Patients with Severe Liver Cancer in ICU. J Healthc Eng. 2022:4452308.
279. Duan H, Luo S, Yu Y, Yan Q. Image Analysis of TVCDS in Infertile Patients with Polycystic Ovary Syndrome. Contrast Media Mol Imaging. 2022:1234983.
280. Cao W, Zhang P, Dong N, et al. Efficacy Evaluation of Zoledronic Acid Combined with Chemotherapy in the Treatment of Lung Cancer Spinal Metastases on Computed Tomography Images on Intelligent Algorithms. Computational and Mathematical Methods in Medicine,2022: 6431852.
281. Zhang J Z, Gao X, Chen Y, et al. Clinical Observation of Ropinirole Hydrochloride in the Treatment of Parkinson’s Disease. Computational and Mathematical Methods in Medicine,2022: 3989770.
282. Zheng Y, Dou H, Li Q,et al. Efficacy and Safety of Cetuximab Plus Cisplatin Alone or in Combination With Paclitaxel in Patients With Head and Neck Squamous Cell Carcinoma: A Randomized Trial.Cancer Control. 2021;28:1073274821997444.
283. Wu B, Zhang F, Jiang W, et al.Nanosilver Dressing in Treating Deep II Degree Burn Wound Infection in Patients with Clinical Studies. Computational and Mathematical Methods in Medicine, 2021: 3171547.
284. Zheng F, Zhang P, Zhao M, et al. Effect of Roxadustat on Factors Associated with Renal Fibrosis and Efficacy. Computational and Mathematical Methods in Medicine,2022: 4764254.
285. Li Y. Analysis of Hepatic Artery Infusion (HAI) Chemotherapy Using Randomized Trials of Floxuridine (FUDR) for Colon Cancer Patients with Multiple Liver Metastases. Gastroenterology Research and Practice,2022: 3546455.
286. Jin X, Weng Q, Min J.To Explore the Haemostatic Effect of Compression Haemostasis Using an Ultrasonic Probe under the Guidance of Ultrasound after Radial Artery Puncture. Disease Markers, 2021: 7423101.
287. Liang L, Qiang F.Observation on the Clinical Effect of Acupuncture and Moxibustion Combined with Repeated Transcranial Magnetic Stimulation on Facial Paralysis. Computational and Mathematical Methods in Medicine,2021: 9642677.
288. Wang Y, Qi S. Study on the Application of the Concept of Childlike Interest with Refined Nursing Intervention in the Treatment of Children with Severe Pneumonia. Computational and Mathematical Methods in Medicine,2022: 5360733.

289. Li A, Gao J, Ni J, et al.Influence of Case Management Model Combined with Continuous Nursing Care on Compliance Behavior and Adverse Emotions in Elderly Patients with Lung Cancer: A Prospective Single-Center Case-Control Study. Computational and Mathematical Methods in Medicine,2022: 2601852.
290. Qu L, Li K, Liu K, et al. Effects of Gemcitabine and Oxaliplatin Combined with Apatinib on Immune Function and Levels of SIL-2R and sicAM-1 in Patients with Gallbladder Cancer. Computational Intelligence and Neuroscience,2022: 4959840.
291. Zhang L, Tian Y, Ren H, et al.Effect of PCI Standardized Telephone Follow-Up Service Mode on Out-of-Hospital Complications, Rehospitalization Rate, and Quality of Life of Discharged Patients with Acute Coronary Syndrome after PCI. Computational and Mathematical Methods in Medicine,2022: 4319887.
292. Wang Z, Chen Q, Yu L, et al. Anesthesia, Sedation, and Unplanned Extubation of Tracheal Intubation in Children with Severe Pneumonia. Evidence-Based Complementary and Alternative Medicine,2021: 4802389.
293. Dong Y, Duan G, Wang H, et al.A Cohort Study of Rivaroxaban Combined with D-Dimer Dynamic Monitoring in the Prevention of Deep Venous Thrombosis after Knee Arthroplasty. Computational and Mathematical Methods in Medicine,2022: 3965039.
294. Qi R.Relationship of Table Tennis Sports Nutritional Food to Sports Athletes' Training and Physical Health. Journal of Healthcare Engineering,2021: 1873312.
295. Hotchandani K D, Thangadurai J, Parate A S, et al. The Effects of Fixed Versus Removable Orthodontic Retainers on Stability and Periodontal Health: 4-Year Follow-Up of a Randomized Controlled Trial. Journal of Pharmacy and Bioallied Sciences, 2023, 15(Suppl 1): S502-S507.
296. Xia L, Ma J, Hu L, et al.Application of Visual Artificial Airway in Patients with ARDS Assisted by Pulmonary Ultrasound. BioMed Research International,2022: 2719016.
297. Wang J, Zhu Q, Zhang S, et al.Observation of Clinical Efficacy of Anisodamine and Chlorpromazine in the Treatment of Intractable Hiccup after Stroke. BioMed Research International,2022: 6563193.
298. Boldt J, Knothe C, Zickmann B, et al. Volume loading with hypertonic saline solution: endocrinologic and circulatory responses. Journal of cardiothoracic and vascular anesthesia, 1994, 8(3): 317-323.
299. Seifi M, Ebadifar A, Kabiri S, et al. Comparative effectiveness of low level laser therapy and transcutaneous electric nerve stimulation on temporomandibular joint disorders. Journal of lasers in medical sciences, 2017, 8(Suppl 1): S27.
300. Khalil MA, Abdel Azeem MS. The impact of dexmedetomidine infusion in sparing morphine consumption in off-pump coronary artery bypass grafting. Semin Cardiothorac Vasc Anesth. 2013;17(1):66-71.
301. Lin Z, Wu Z, Wang Y, et al. Comparison of the Efficacy and Safety of Neuroendoscopic Endonasal Transsphenoidal Surgeries and Intracranial Endoscopic Pterional Approach in Resection of Tuberculum Sellae Meningiomas. Evidence-Based Complementary and Alternative Medicine,2022: 4542815.
302. Boldt J, Bormann B, Kling D, et al. Influence of nimodipine and nifedipine on intrapulmonary shunting—a comparison to other vasoactive drugs. Intensive care medicine, 1987, 13: 52-56.
303. Liu M, Wang H, Du S, et al. Laparoscopic Radical Hysterectomy Combined with Neoadjuvant Chemotherapy for Cervical Cancer Patients Effectively Improves Immune Function. Disease Markers,2022: 3611174.
304. Ji X, Jing X, Liu Y, et al.Clinical Application of Anlotinib Combined with Docetaxel: Safe and Effective Treatment for Lung Carcinoma. Disease Markers,2022: 2483816.
305. He X, Geng X, Sha M, et al.Effect of Targeted Care plus Exercise Intervention on Blood Glucose Levels and Maternal and Newborn Outcomes in Patients with Gestational Diabetes Mellitus. Disease Markers, 2022: 7584936.

306. Sun J, Wang X, Liu Y, et al.An Analysis of the Effect of Noninvasive Positive Pressure Ventilation on Patients with Respiratory Failure Complicated by Diabetes Mellitus. Disease Markers, 2022: 3597200.
307. Zeng G, An H, Li W, et al. Correlation between Glycated Haemoglobin Level, Cardiac Function, and Prognosis in Patients with Diabetes Mellitus Combined with Myocardial Infarction. Disease Markers,2022: 2191294.
308. Huang H, Zhang A, Gao H, et al.Application of Preoperative Adductor Canal Block Coupled with General Anaesthesia in Elderly Patients Undergoing Total Knee Arthroplasty. Disease Markers, 2022: 3071665.
309. Fu J, Wang T, Li B, et al. The Efficacy of Rosuvastatin, Amlodipine, and Aspirin in the Treatment of Hypertension with Coronary Heart Disease and Its Effect on Platelet Aggregation. Disease Markers, 2022: 1111438.
310. Ma F, Zhang J, Wang H, et al.Efficacy of Dapagliflozin in Patients with Diabetes Mellitus Complicated with Coronary Artery Disease and Its Impact on the Vascular Endothelial Function. Disease Markers,2022: 4829750.
311. Zhang D, Liang Z, Wang D, et al. The Clinical Efficacy and Safety of Extracorporeal Shock Wave Lithotripsy in the Treatment of Patients with Urinary Calculi. Disease Markers,2022: 3468692.
312. Zhang S, Ye H, Sun J, et al.To Explore and Analyze the Safety and Clinical Efficacy of Periocline-Assisted Periodontal Basic Therapy on Chronic Periodontitis. Disease Markers,2022: 4601259.
313. Liu Y, Sun Y, Wang X, et al. Efficacy of the Panax Notoginseng Ejiao Suppository in the Treatment of Patients with Ulcerative Proctitis and Its Effect on Inflammatory Response and Immune Function. Disease Markers, 2022: 1479964.
314. Gao G, Su J.Nursing Intervention Based on Smart Medical Care on the Sleep Quality of Cardiology Patients. Journal of Healthcare Engineering, 2021: 9947438.
315. Cheng Z, Zheng W.Clinical Effect and Aesthetic Observation of All-on-4 Immediate Loading Implant Denture in Severe Periodontitis. Evidence-Based Complementary and Alternative Medicine,2021: 3120260.
316. Zhang L, Du H, Song J.Effect of Holistic Nursing Intervention Combined with Humanized Nursing Intervention on Activities of Daily Living and Limb Movement Ability of Elderly Patients with Cerebral Hemorrhage after Surgery. Evidence-Based Complementary and Alternative Medicine, 2021: 2480551.
317. Zhang Y, Li M, Li L, et al.Randomized Controlled Study of the Effects of DHEA on the Outcome of IVF in Endometriosis. Evidence-Based Complementary and Alternative Medicine,2021: 3569697.
318. Li H, Xu D.The Effect of Azithromycin Combined with Palm Massage on Pulmonary Function of Children with Mycoplasma Pneumonia. Evidence-Based Complementary and Alternative Medicine, 2022: 1857055.
319. Hong Z, Zheng M, Li Y, et al.Acute Thrombolytic Therapy Combined with the Green Channel Can Reduce the Thrombolytic Time and Improve Neurological Function in Acute Stroke Patients. Evidence-Based Complementary and Alternative Medicine, 2022: 1127159.
320. Chang L, Lan H.Effect of Neoadjuvant Chemotherapy on Angiogenesis and Cell Proliferation of Breast Cancer Evaluated by Dynamic Enhanced Magnetic Resonance Imaging. BioMed Research International, 2022: 3156093.
321. Cui Y, Ni Y, Yan H.The Application Value of Clinical Intervention Approaches Based on the Guidance of Knowledge, Belief, and Action Theory for Children with AB and the Analysis of Risk Factors for Poor Prognosis. BioMed Research International, 2023: 1816818.
322. Pei X, Hu X, Xu Z, et al.Clinical Effect of Retroperitoneal Laparoscopic Radical Nephrectomy on Renal Cell Carcinoma, the Influence of Renal Function, and the Influencing Factors of Recurrence. Evidence-Based Complementary and Alternative Medicine, 2022: 4182853.

323. Yin L.Targeted Nursing Combined with Endoscopic Submucosal Injection of Carbon Nanoparticles in the Treatment of Colorectal Cancer. Evidence-Based Complementary and Alternative Medicine, 2022: 8663645.
324. Chen J, Feng L, Sheng Q, et al.Efficacy, Safety, and Tumor Marker Inhibition of Apatinib Combined with Conventional Chemotherapy Regimens for Patients with Advanced Triple-Negative Breast Cancer. Evidence-Based Complementary and Alternative Medicine, 2021: 8720679.
325. Zhao H, Li X, Zhao X, et al.Comparative Analysis of the Effects of the Anti-VEGF Drug and Glucocorticoid by Injection before the End of Vitrectomy for Proliferative Diabetic Retinopathy. Evidence-Based Complementary and Alternative Medicine, 2021: 1285372.
326. Zhang J X, Wang W Y.Clinical Efficacy of Tuomin Zhiti Decoction in Allergic Rhinitis. Evidence-Based Complementary and Alternative Medicine, 2022: 8616075.
327. Shen W, Liu X, Zhou A.Analysis of Continuous Nursing Intervention on Aplastic Anemia Patients Based on the "Information-Motivation-Behavioral Skills Model". Evidence-Based Complementary and Alternative Medicine,2021: 8204522.
328. Ma Y, Zhang Y.Nifedipine plus Systematic Nursing on Pregnancy Outcomes in Patients with Gestational Hypertension. Evidence-Based Complementary and Alternative Medicine,2022: 4417790.
329. Zhang X, Wu Y.Acupoint Massage plus Recombinant Bovine Basic Fibroblast Growth Factor Ophthalmic Gel and Limbal Stem Cell Transplantation on Visual Quality, Corneal Refraction, and Aesthetic Outcome in Patients with Pterygium. Evidence-Based Complementary and Alternative Medicine, 2022: 7103496.
330. Zhang L, Zhang W, Jiang Y, et al.Effects and Satisfaction of Comfort Nursing plus Psychological Nursing in the Clinical Nursing of Neurology Patients: A Comparative Study. Evidence-Based Complementary and Alternative Medicine,2022: 8013787.
331. Jin H, Zhang H.Comparative Analysis of Clinical Effects of Insulin Aspart Combined with Acarbose and Metformin in the Treatment of Diabetes Mellitus. Evidence-Based Complementary and Alternative Medicine, 2022: 3541931.
332. Yang J, Zou J, Zhang Q.Therapeutic Value of Traditional Chinese Massage plus Moxibustion for Degenerative Knee Osteoarthritis. Evidence-Based Complementary and Alternative Medicine,2022: 8305561.
333. Zhang F, Guo P.Effect of Calcium Carbonate Preparation on Malnutrition in Preschool Children. Evidence-Based Complementary and Alternative Medicine,2022: 4025972.
334. Yang S, Liu Y, Wen J, et al.Clinical Efficacy of Dienogest versus Levonorgestrel-Releasing Intrauterine System for Adenomyosis. Evidence-Based Complementary and Alternative Medicine,2022: 1995472.
335. Rao L, Zhou G, Gao Y, et al.Effectiveness of Laparoscopic Cholecystectomy in Patients with Gallbladder Stones with Chronic Cholecystitis. Evidence-Based Complementary and Alternative Medicine,2022: 1434410.
336. Li H, Wang K, Qiu Y, Liu X, Ma X, Li T, Cao Q, Wang Z. Application of General Anesthesia Combined with Saphenous Nerve-Tibial Nerve Block in Total Knee Arthroplasty. Evid Based Complement Alternat Med.2022:7010492.
337. Chen R, Zhang M, Zhang Y.The Effect of TCM Syndrome Type and Western Medicine Detection on Patients with Hypertension and Diabetes Mellitus. Evidence-Based Complementary and Alternative Medicine, 2022: 2430806.
338. Shi J, Wang R, Qin S, et al.The Application of Dopamine Combined with Intravenous Furosemide Infusion Therapy Has an Apparent Clinical Effect in Treating Patients with Heart Failure. Evidence-Based Complementary and Alternative Medicine,2022: 1055160.
339. Xiao H, Zhao Z, Zhang C, et al.Influence of Standardized Nursing Intervention Combined with Mindfulness Stress Reduction Training on the Curative Effect, Negative Emotion, and Quality of Life in Patients with Chronic Gastritis and Gastric Ulcer. Evidence-Based Complementary and Alternative Medicine,2021: 2131405.

340. Luo M, Zhang H.Vitamin AD Drops are More Effective than Intramuscular Injection of Thymosin in Reducing the Rate of Growth Retardation in Children. Evidence-Based Complementary and Alternative Medicine, 2022: 7799111.
341. Gan L, Hu J, Xia L, et al.Therapeutic Effect of Continuous Blood Purification Combined with Humanized Nursing in Patients with Severe Sepsis. Evidence-Based Complementary and Alternative Medicine,2022: 1411371.
342. Zhu X, Wei Z, Liu X.Efficacy of Ambroxol Hydrochloride Combined with Amoxicillin Potassium Clavulanate Combination on Children with Bronchopneumonia and Its Impact on the Level of Inflammatory Factors. Evidence-Based Complementary and Alternative Medicine, 2022: 2604114.
343. Chen Z, Chen J.The Efficacy of Calcium Carbonate-Vitamin D3 in Pregnant Women for the Prevention of Hypertensive Disorders in Pregnancy. Evidence-Based Complementary and Alternative Medicine, 2022: 7971976.
344. Zhao Z, Ma Y, Liu Q, et al.Effects of Different Doses of Clopidogrel plus Early Rehabilitation Therapy on Motor Function and Inflammatory Factors in Patients with Ischemic Stroke. Evidence-Based Complementary and Alternative Medicine, 2022: 9692382.
345. Peng Y, Guan Q.Comparison of Dexmedetomidine and Etomidate on Intraoperative Wake-Up Equality, Hemodynamics, and Cerebral Protection in Operation of the Brain Functional Area. Evidence-Based Complementary and Alternative Medicine,2021: 6363188.
346. Zhao Z, Chen Z.Efficacy and Safety of Endoscopic Tympanic Membrane Catheterization Plus Ofloxacin Ear Drops in the Treatment of Secretory Otitis Media in Infants and Toddlers. Evidence-Based Complementary and Alternative Medicine, 2022: 3732243.
347. Wu L, Jiang W, Zhang M, et al.Observation of the Effect of TTM-Based Health Information Behavior Combined with Continuous Nursing on Cognitive and Motor Function, Living Ability, and the Quality of Life of Cerebral Stroke Patients. Evidence-Based Complementary and Alternative Medicine, 2022: 1850033.
348. Zhang X, Liu M, Mao Y.Efficacy of Fluticasone and Salmeterol Dry Powder in Treating Patients with Bronchial Asthma and Its Effect on Inflammatory Factors and Pulmonary Function. Evidence-Based Complementary and Alternative Medicine, 2022: 8555417.
349. Zhuang Z, Cui Y N, Cai G, et al.Clinical Efficacy of Auricular Acupressure plus Eye Exercises in the Treatment of Adolescent Pseudomyopia. Evidence-Based Complementary and Alternative Medicine,2022: 9208478.
350. Gao J, Zhang Q, Zhao X, et al.Influence of Early Predictive Nursing on Complications and Quality of Life in Patients after Colorectal Cancer Surgery. Evidence-Based Complementary and Alternative Medicine, 2022: 8410664.
351. Fang L, Xu Y, Tong X, et al.Efficacy of Risperidone Orally Disintegrating Tablets Combined with Oxazepam in the Treatment of Schizophrenia. Evidence-Based Complementary and Alternative Medicine, 2022: 2344946.
352. Huang K, Zhang Y, Yang F, et al.Effect of Enalapril Combined with Bisoprolol on Cardiac Function and Inflammatory Indexes in Patients with Acute Myocardial Infarction. Evidence-Based Complementary and Alternative Medicine, 2022: 6062450.
353. Xu D, Cao H, Fan Y, et al.Comparative Analysis of the Effect of Low-Frequency Repeated Transcranial Magnetic Stimulation and Extracorporeal Shock Wave on Improving the Spasm of Flexor after Stroke. Evidence-Based Complementary and Alternative Medicine,2021: 7769581.
354. Dai S, Sun W, Xu H, et al.Effect of Applying Binocular Visual Training after Slanted Lateral Rectus Recession on Orthophoric Rate and Binocular Visual Function Recovery on Patients with Convergence Insufficiency-Type Intermittent Exotropia. Evidence-Based Complementary and Alternative Medicine,2021: 7202319.
355. Wen Q, Yao S, Yao B.Effectiveness of Comprehensive Nursing in Hemodialysis of Patients with Chronic Renal Failure and the Impact on Their Quality of Life. Evidence-Based Complementary and Alternative Medicine, 2022: 1399650.
356. Zhao X, Zhang C, Lou H, et al.Clinical Efficacy and Safety Study of Mifepristone with Misoprostol Treatment in Patients with Missed Abortion. Evidence-Based Complementary and Alternative Medicine, 2021: 9983023.

357. Yu H, Dong W.Effect of Human-Oriented Fine Nursing on Psychological Emotion, Life Quality, and Nursing Satisfaction of Patients Undergoing Laparoscopic Radical Nephrectomy. Evidence-Based Complementary and Alternative Medicine,2022: 5016967.
358. Ping H, Ling X, Xue Y, et al.Effect of ERAS Combined with Comfortable Nursing on Quality of Life and Complications in Femoral Neck Fractures of the Aged People. Evidence-Based Complementary and Alternative Medicine,2021: 8753076.
359. Jun W, Tian Y.Application of Meditation Relaxation Training and Rosenthal Effect in Patients with Adenoidectomy. Evidence-Based Complementary and Alternative Medicine, 2022: 1420639.
360. Zhang H, Jing Z, Li F, et al.The Clinical Effects of Metronidazole Vaginal Effervescent Tablets Combined with Kushen Suppository in the Treatment of Trichomonas Vaginitis. Evidence-Based Complementary and Alternative Medicine, 2022: 7730474.
361. Ding H, Jiang Y.Effect of High-Quality Nursing Intervention on the Quality of Life and Psychological State of Tumor Patients Undergoing First Chemotherapy. Evidence-Based Complementary and Alternative Medicine, 2022: 9466665.
362. Zhang S, Zhu Y, Jin Y, et al.Difference between Acyclovir and Ganciclovir in the Treatment of Children with Epstein–Barr Virus-Associated Infectious Mononucleosis. Evidence-Based Complementary and Alternative Medicine,2021: 8996934.
363. Ai D, Gu Y, Xu S.Effectiveness of Multidisciplinary Nursing Based on Fever, Blood Sugar, and Swallowing Function Management in Patients with Acute Stroke. Evidence-Based Complementary and Alternative Medicine, 2022: 5949760.
364. Qian X, Gong L, Zhou F, et al.High-Quality Nursing Combined with the Whole-Course Responsibility Nursing Intervention Reduces the Incidence of Complications in Severe Aneurysmal Subarachnoid Hemorrhage. Evidence-Based Complementary and Alternative Medicine, 2022: 3252718.
365. Yi C, Feng X, Yuan Y. Study on the Influence of PDCA Cycle Nursing Based on Network Service on the Quality of Life and Nutritional Status of Hypertension Patients in Home Care. Evid Based Complement Alternat Med.2021:6068876.
366. Qian F, Zhang Y, Chen Y.Effect of Grading Rehabilitation Nursing Mode on Limb Function, Speech Rehabilitation, and Quality of Life of Stroke Patients. Evidence-Based Complementary and Alternative Medicine, 2022: 6956406.
367. Gao F, Wang H, Wang Z.Clinical Application of Microsurgery Using the Cerebellar Medulla Fissure Approach in Severe Ventricular Hemorrhage with Casting of the Fourth Ventricle and Its Influence on Neurological Recovery. Evidence-Based Complementary and Alternative Medicine,2021: 3699233.
368. Cao C, Chen L, Li S, et al.Clinical Efficacy of Carbon Dioxide Laser Combined with ALA Photodynamics in the Treatment of Condyloma Acuminatum. Evidence-Based Complementary and Alternative Medicine, 2021: 7211055.
369. Shen X, Yang Q, Li L, et al.Clinical Pregnancy and Incidence of Ovarian Hyperstimulation Syndrome in High Ovarian Responders Receiving Different Doses of hCG Supplementation in a GnRH-Agonist Trigger Protocol. Evidence-Based Complementary and Alternative Medicine,2021: 2180933.
370. Wei L, Yang H, Sun X.The Effect of Oxytocin plus Carboprost Methylate in Preventing Postpartum Hemorrhage in High-Risk Pregnancy and Its Effect on Blood Pressure. Evidence-Based Complementary and Alternative Medicine,2022: 9878482.
371. Lin F, Chen Q, Lin M, et al.Clinical Effect of Nursing Based on the Kano Model in Emergency Multiple Injuries. Evidence-Based Complementary and Alternative Medicine, 2022: 3586290.
372. Zhang Z, Dong M, Han Y, et al.Application Effect of Medical Care Integration Combined with Family Intervention under the Evidence-Based Nursing Mode on Child Patients with Severe Hand-Foot-Mouth Disease and Its Influence on Intestinal Function. Evidence-Based Complementary and Alternative Medicine,2021: 9599711.

373. Zheng W, Sun X, Liu J.Efficacy of Donepezil Hydrochloride plus Olanzapine for Senile Dementia and Its Effect on the Recovery of Cognitive Function. Evidence-Based Complementary and Alternative Medicine, 2022: 4156312.
374. Wang G, Liu X, Guo Z, et al.Effect of Entresto on Clinical Symptoms, Ventricular Remodeling, Rehabilitation, and Hospitalization Rate in Patients with Both Acute Myocardial Infarction and Acute Heart Failure. Evidence-Based Complementary and Alternative Medicine,2022: 7650937.
375. Ge G, Wang C.Effect of Percutaneous Nephrolithotomy Combined with Needle Nephrolithotomy on Renal Function and Complication Rate in Patients with Complex Renal Calculi. Evidence-Based Complementary and Alternative Medicine,2022: 7312960.
376. Chen B, Qu X, Fang X, et al.Effect of Oral Tranexamic Acid on the Blood Transfusion Rate and the Incidence of Deep Vein Thromboembolism in Patients after TKA. Evidence-Based Complementary and Alternative Medicine,2022: 6041827.
377. Yu D, Liu Z, Wang H, et al.Analysis on the Effect of Different Surgical Methods on the Treatment of Senile Osteoporotic Spinal Compression Fractures and the Influencing Factors of Complications. Evidence-Based Complementary and Alternative Medicine,2021: 1599470.
378. Wang M, Zhang Y, Zhong A, et al.Care Bundles plus Detailed Nursing on Mortality and Nursing Satisfaction of Patients with Septic Shock in ICU. Evidence-Based Complementary and Alternative Medicine, 2022: 1177961.
379. Wu C, Chen B, Wang M, et al.Chemotherapy with a TP Regimen in Combination with Stereotactic Radiotherapy Could Significantly Optimize the Clinical Efficacy of NSCLC Treatment. Evidence-Based Complementary and Alternative Medicine,2022: 8495452.
380. Peng N, Li J.Application Effect of Case Management Mode Combined with ERAS in Elderly Patients with Hip Fracture. Evidence-Based Complementary and Alternative Medicine,2021: 1175020.
381. Tang X, Ren Z, Miao Y, et al.The Infection Control Route in the Operating Room Effectively Reduces the Wound Infection of Patients. Evidence-Based Complementary and Alternative Medicine, 2022: 9270045.
382. Zhang L, Wang Y, Zhang L, et al.The Effect of Propofol plus Remifentanil for PostoperativePain and Heart Rate Management in Patients Undergoing Abdominal Hysterectomy. Evidence-Based Complementary and Alternative Medicine, 2022: 7646361.
383. Yuan Y, Zhao J, He N.Observation on the Effect of Bone Grafting Alone and Guided Tissue Regeneration Combined with Bone Grafting to Repair Periodontal Intraosseous Defects. Evidence-Based Complementary and Alternative Medicine,2021: 1743677.
384. Lu Y, Wu N, Ma B, et al.Effect of Root Canal Therapy Combined with Full Crown Restoration on the Level of Inflammatory Factors and Chewing Function in Patients with Cracked Teeth and Chronic Pulpitis. Evidence-Based Complementary and Alternative Medicine,2021: 3299349.
385. Liu Y, Zeng Q.Intensive Health Care plus Vitamin D Administration Benefits the Growth and Development of Young Children and Reduces the Incidence of Nutritional Disorders. Evidence-Based Complementary and Alternative Medicine, 2022: 8097035.
386. Zhang Y, Li K, Li N.The Efficacy of Orthodontics plus Implant Anchorage in Orthodontic Treatment: A Randomized Controlled Study. Evidence-Based Complementary and Alternative Medicine, 2022: 4049076.
387. Lian Y, Fu H, Xu X, et al.Application Effect and Accuracy Analysis of Electrochemiluminescence Immunoassay and Enzyme-Linked Immunosorbent Assay in the Serological Test of Hepatitis B Virus. Evidence-Based Complementary and Alternative Medicine,2022: 9371497.
388. Zhang L, Yang H, Yang P.Comparison of the Ameliorating Effects of Valsartan and Amlodipine on Vascular Endothelial Dysfunction and Oxidative Stress in Elderly Patients with Type H Hypertension. Evidence-Based Complementary and Alternative Medicine, 2022: 5054511.
389. Zhou C, Tang J, Sun F, et al.Continuity of Care plus Whole Process Psychological Intervention for Lung Cancer Patients undergoing Chemotherapy. Evidence-Based Complementary and Alternative Medicine, 2022: 4330059.

390. Chen P, Yang J, Hu D, et al.Safety of Different Anesthesia Methods Combined with Intravenous Fast Channel Anesthesia in Lower Extremity Orthopedic Surgery of the Elderly. Evidence-Based Complementary and Alternative Medicine,2021: 9787879.
391. Lu M, Jin Y.Efficacy Evaluation of the Combined Platelet-Rich Plasma and Hyaluronic Acid after Arthroscopic Joint Debridement in Treating Knee Osteoarthritis. Scanning, 2022: 6994017.
392. Feng D, Liu Z, Chen H, et al.Clinical Efficacy and Safety of Ibuprofen plus Traction, Reposition, and Hip Spica Cast in the Treatment of Developmental Dysplasia of the Hip. Evidence-Based Complementary and Alternative Medicine,2022: 1213133.
393. Sun C, Yang Q, Wang C, et al.Efficacy of Different Preemptive Analgesia on Postoperative Analgesia, Oxidative Stress, and Inflammatory Response after Gynecological Laparoscopic Surgery. Evidence-Based Complementary and Alternative Medicine, 2021: 4233716.
394. Hu X, Wang H, Lin Y.Effects of Collagen Antibacterial Functional Dressing plus Continuous Nursing on Lower Extremity Skin Injury Caused by Norepinephrine in Patients with Septic Shock. Evidence-Based Complementary and Alternative Medicine,2022: 4160637.
395. Zhang X.Effects of Targeted Intervention plus Comprehensive Nursing on the Quality of Life and Nursing Satisfaction in Multiple Traumas. Evidence-Based Complementary and Alternative Medicine, 2022: 8929418.
396. Shu J, Li D, Tao W, et al.Observation on the Curative Effect of Massage Manipulation Combined with Core Strength Training in Patients with Chronic Nonspecific Low Back Pain. Evidence-Based Complementary and Alternative Medicine, 2021: 7534577.
397. Chen J, Qian L, Chen C, et al.The Characteristics of Fear of Recurrence and the Effect of Cognitive-Behavioral Stress Management Intervention in Patients after Radiofrequency Ablation of Atrial Fibrillation. Evidence-Based Complementary and Alternative Medicine,2022: 6916302.
398. He F, Xia Z, Wang H, et al.Clinical Efficacy and Safety of Tenofovir in the Treatment of Patients with Chronic Hepatitis B. Evidence-Based Complementary and Alternative Medicine, 2022: 1673453.
399. Gao P, Wang W, Yang W, et al. Effect of Cryotherapy plus Flurbiprofen Axetil for Pain Management in Children Undergoing Tonsillectomy. Evidence-Based Complementary and Alternative Medicine,2022: 7687437.
400. Wang Y, Lin H, Wang L, et al. Clinical Observation on the Nursing Effect of Mindfulness-Based Stress Reduction Combined with Solution-Focused Brief Therapy in Uremic Peritoneal Dialysis Patients and Influence on Nutritional Status. Evidence-Based Complementary and Alternative Medicine,2021: 3751585.
401. Liu S, Chu Z. Ropivacaine and Lidocaine in Double Eyelid Blepharoplasty: A Comparative Analysis. Evidence-Based Complementary and Alternative Medicine,2022: 7029570.
402. Yang H, Chen Y, Fu M. Research on the Application Effect of Strengthening Risk Management in Continuous Renal Replacement Therapy Nursing of Critically Ill Patients. Evidence-Based Complementary and Alternative Medicine,2022: 2363877.
403. Zhong M, He L, Chen M, et al. Effect Evaluation of Comfort Nursing Materials Assisted Nursing for Patients with Advanced Malignant Tumor. Scanning,2022: 4766252.
404. Zhu S, Gao J. Effect of Rehabilitation Training Based on Automatic Extraction Algorithm on Knee Anterior Cruciate Ligament Injury Caused by Exercise. Scanning, 2022: 8304071.
405. Wu H, Wen Y, Guo S. Role of Nutritional Support under Clinical Nursing Path on the Efficacy, Quality of Life, and Nutritional Status of Elderly Patients with Alzheimer's Disease. Evidence-Based Complementary and Alternative Medicine, 2022: 9712330.
406. Li Y, Hong M, Liu G. Changes in the Quality of Life, Psychological Status, Medication Compliance, and Prognosis of Patients with Acute Myocardial Infarction after PCI by Applying PDCA Cycle Management Model. Evidence-Based Complementary and Alternative Medicine, 2021: 7318653.

407. Xu D, Chu T, Tao G. Clinical Study on the Efficacy of Silver Ion Dressing Combined with Prontosan Gel Dressing in the Treatment of Diabetic Foot Ulcers and the Effect on Serum Inflammatory Factors. Evidence-Based Complementary and Alternative Medicine, 2021: 2938625.
408. Xue J, Fu X, Hu Z. Continuous Lumbar Plexus Block under the Guidance of the "Shamrock Method" Ultrasound: Analgesic Effects and Hemodynamic Effects after Total Knee Arthroplasty in Elderly Patients. Evidence-Based Complementary and Alternative Medicine, 2021: 3531236.
409. Cui L, Bai Z. Effect of Moxibustion Instrument Combined with Intermediate Frequency Electrostatic Therapy on Pain and Joint Function in Elderly Patients with Cold-Dampness Knee Arthritis. Computational and Mathematical Methods in Medicine, 2022: 2613901.
410. Chen X. Analysis of the Effect of Nursing Intervention for Thyroid Diseases Based on Family Nursing Methods. Computational and Mathematical Methods in Medicine, 2022: 1766544.
411. Shi W, Shen Y, Zhang B, et al. Analysis of the Nursing Effect of Respiratory Critical Illness Based on Refined Nursing Management. Computational and Mathematical Methods in Medicine, 2022: 6458705.
412. Chen Y, Ding J, Li C, et al. Study on Nursing Effect of Psychological Intervention on Uremic Hemodialysis Patients. Computational and mathematical methods in medicine, 2022: 8040656.
413. Gong T, Wang Y, Pu H, et al. Study on the Application Value of PBL Combined with Situational Simulation Teaching Method in Clinical Practice Teaching of Radiology Department. Computational and Mathematical Methods in Medicine, 2022: 6808648.
414. Li S, Fu L. Application Effect of Robot-Assisted Laparoscopy in Hepatectomy for Colorectal Cancer Patients with Liver Metastases. Computational and Mathematical Methods in Medicine, 2022: 5492943.
415. Liu D, Ruan M, Tong C, et al. Effect of Shugan Jianpi Recipe Combined with Cross Moxibustion on Biochemical Examination Indexes and Total Score of TCM Symptoms in Patients with Spleen-Stomach Damp-Heat Diarrhea Irritable Bowel Syndrome. Computational and Mathematical Methods in Medicine, 2022: 8286146.
416. Wang L, Lei X, Wang X. Efficacy and Safety of PD-1/PD-L1 Inhibitor Chemotherapy Combined with Lung Cancer Fang No. 1 in Relapsed and Refractory SCLC: A Retrospective Observational Study. Computational and Mathematical Methods in Medicine, 2022: 2848220.
417. Shan L, Li J, Yang P R, et al. Application of Touching Combined with Intelligent Interaction of Voice and Rhythm in Nursing Care of Newborns with Feeding Intolerance and Its Influence on Quality of Life. Computational and Mathematical Methods in Medicine, 2022: 4747337.
418. Wang X Q, Hu Y, Hong K. A Case-Control Study of Photodynamic Therapy Combined with Thymosin in the Treatment of Condyloma Acuminatum in Anal Canal. Journal of Healthcare Engineering, 2022: 3019379.
419. He X. Observation on the Rehabilitation Effect of Athletes with Anterior Cruciate Ligament Injury of the Knee Based on Multidisciplinary Combined Nondrug Therapy. Journal of Healthcare Engineering, 2022: 9528354.
420. Zhu J, Chen S, Wang Y, et al. Clinical Study on the Efficacy of Microwave Ablation (MA) in the Treatment of Stage I Renal Clear Cell Carcinoma by CT and MRI Imaging. Journal of Healthcare Engineering, 2022: 8446294.
421. Yu D, Jiang J, Jiang Z, et al. Application of Ultrasound-Guided Upper Trunk Brachial Plexus Block in Observation of Lower Shoulder Surgery. Contrast Media & Molecular Imaging,2022: 9168566.
422. Wang X, Wang J, Xie W. Effects of CT Combined with Modified Qinfan Decoction on Improving Sores and Promoting Angiogenesis. Contrast Media & Molecular Imaging,2022: 1649904.

423. Zhang W, Xu R L. Effect of Exercise Intervention on Internet Addiction and Autonomic Nervous Function in College Students. BioMed Research International,2022: 5935353.
424. Gu X, Yang M, Liu F, et al. Effects of Adding Ultrasound Biofeedback to Individualized Pelvic Floor Muscle Training on Extensibility of the Pelvic Floor Muscle and Anterior Pelvic Organ Prolapse in Postmenopausal Women. Contrast Media & Molecular Imaging, 2022: 4818011.
425. Zhou Y, Cai W, Wang X. Auricular Point Pressing Beans + Continuous Intervention on β2-MG, Curative Effect, and Relationship with Prognosis in Elderly MHD Sufferers. Contrast Media Mol Imaging. 2022:4166420.
426. Wang F, Gan X, Zhou X, et al. Application of Self-Adhesive Soft Silicone Common Foam Dressing in Reducing Intraoperative Pressure Ulcers in Elderly ICU Patients. Computational and Mathematical Methods in Medicine, 2021: 4482201.
427. Wang H, Ma D, Hu J, et al. Analysis of the Effects of Humidified High Flow Nasal Oxygen Therapy Combined with Noninvasive Mechanical Ventilation on Treatment Outcomes. Computational Intelligence and Neuroscience,2022: 2910813.
428. Lu W W, Duan X Y, Ni J L, et al. A Prospective Study of Using Chaihu Shugan Powder Combined with Zu San Li Acupoint Stimulation to Improve the Prognosis of Liver Stagnation and Qi Stagnation Syndrome in Acute Pancreatitis. Evidence-Based Complementary and Alternative Medicine,2022: 3177201.
429. Zou T, Liu J, Tan L. Study on the Effect of Hope Theory Combined with Psychological Intervention on the Improvement of Prognosis. Contrast Media & Molecular Imaging,2022: 1153071.
430. Yin M, Li J, Wang J, et al. Observation of the Effect of Focused Psychological Intervention Combined with Standardized Pain Nursing on Postoperative Pain Levels and Depression and Anxiety in Patients with Intestinal Obstruction. Disease Markers,2022: 2467887.
431. Wang F, Wen S, Chen J, et al. Modified Lamaze Breathing Reduces Abdominal Pain in Patients during Colonoscopy. Computational and Mathematical Methods in Medicine, 2022: 1557861.
432. Ding M, Xu X, Xia L, et al. Magnetic Resonance Imaging Characteristic Evaluation of Dexmedetomidine on Neurocognitive Dysfunction in Elderly Patients with Colorectal Tumors after Laparoscopic Operation. Computational and Mathematical Methods in Medicine,2022: 1345695.
433. Zhu B, Zhang S. Flexible Ureteroscopy and Nephroscopy for Stone Removal in Patients with Multiple Renal Calculi. Disease Markers, 2022: 2078979.
434. Ou J, Wang L. Efficacy of Self-Made Hewei Decoction for Chronic Atrophic Gastritis and Its Effect on Gastrin and Pepsinogen Expression Levels. Contrast Media & Molecular Imaging, 2022: 1092695.
435. Chen Z, Zheng B, Yang H, et al. Analysis of the Effect of Laparoscopic and Open Surgical Treatment in Children with Congenital Megacolon. Computational Intelligence and Neuroscience, 2022: 2669294.
436. He L, Huang L. A Study on the Effects of a Cartoon Text Version of Health Education Manual with Sandplay on the Psychological Status and Cognitive Function of Children with Attention Deficit Hyperactivity Disorder. Evidence-Based Complementary and Alternative Medicine, 2022: 1816391.
437. Feng Y, Ren J, Zhang Y, et al. A Cohort Study of Surgical Indexes, Postoperative Complications, Recovery Speed, and Prognosis of Stanford Type A Aortic Dissection Compared with Traditional Sun’s Operation. Evidence-Based Complementary and Alternative Medicine,2022: 9516922.
438. Jiang W, Wang J, Ni X, et al. Anesthesia Effect of Remifentanil Combined with Propofol in Laparoscopic Cholecystectomy and Its Impact on Postoperative Cognitive Recovery. Evidence-Based Complementary and Alternative Medicine,2022: 9147416.
439. Zu B, Yang Y, Shi S. Effects of Different Doses of Dexmedetomidine Combined with Thoracic Paravertebral Nerve Block Anesthesia on Agitation and Hemodynamics in Patients Undergoing Thoracotomy during Recovery. Evidence-Based Complementary and Alternative Medicine,2022: 7804584.

440. Yu K, Zhou H. Clinical Curative Effects and Influencing Factors of Uterine Artery Chemoembolization Combined with Uterine Curettage Treating with Cesarean Scar Pregnancy Patients. Evidence-Based Complementary and Alternative Medicine,2022: 7785573.
441. Sun X, Lin T, Fang J, et al. Clinical Efficacy Analysis of Biofeedback Electrical Stimulation Combined with Doxycycline in the Treatment of Type IIIA Chronic Prostatitis. Evidence-Based Complementary and Alternative Medicine, 2022: 7150204.
442. Zhao X, He J, Liu J. Effects of Positive Psychological Nursing Combined with Free Posture on the Prognosis of Primipara with Singleton Spontaneous Delivery. Evidence-Based Complementary and Alternative Medicine, 2022: 6393050.
443. Liu Q, Guo Y, Wei P, et al. The Application of Nurse Stratified Management in Nursing Management. Evidence-Based Complementary and Alternative Medicine,2022: 6368765.
444. Xu Z, Li S, Wu J, et al. Posterior Lumbar Plexus Block Anesthesia for Elderly Patients with Lower Limb Fracture. Disease Markers, 2022: 8494796.
445. Wang Y, Lin S, Chen Z, et al. Effects of Infrared Combined with Methylcobalamin on the Vibratory Sensory Threshold and Nerve Conduction Velocity of the Lower Extremity in Patients with Diabetic Foot Treatment. Disease Markers,2022: 8287192.
446. Yu X, Lyu H, Yu B, et al. Analysis of the Clinical Effect of Implementing Health Education in the Process of Ministration Elderly Hypertensive Sufferers. Contrast Media & Molecular Imaging,2022: 8357617.
447. Liu R, Suo S, Wang Y, et al. Effects of Dexmedetomidine and Propofol on Postoperative Analgesia and the Cellular Immune Function of Patients Undergoing Radical Gastrectomy for Gastric Cancer. Contrast Media & Molecular Imaging, 2022: 7440015.
448. Zha J, Zhang G, Zhang J, et al. Analysis of the Effect of Posterior Lateral Malleolus Plate Fixation on the Curative Effect and Mechanical Stability of Complex Ankle Fractures. Contrast Media & Molecular Imaging, 2022: 7101007.
449. Li Z, Liu J, Liu P, et al. Effects of Electroacupuncture with Different Waveforms on Chronic Prostatitis/Chronic Pelvic Pain Syndromes: A Randomized Controlled Trial. Contrast Media & Molecular Imaging, 2022: 6866000.
450. Xiao L, Xiao T, Yu F. Effect Analysis of Clinical Pathway Nursing Combined with Humanized Nursing in Cerebral Infarction. Computational Intelligence and Neuroscience, 2022: 8270137.
451. Zhu X, Sun Y. CRP and ALT Levels in Individuals with Acute Biliary Pancreatitis after Endoscopic Retrograde Cholangiopancreatography and Endoscopic Sphincterotomy. Computational Intelligence and Neuroscience, 2022: 6492551.
452. Yu Z, Shu H, An X, et al. Application and Effect Evaluation of Needle Tract Nursing after External Fixation with 2% Chlorhexanol Gluconate Gauze. Computational Intelligence and Neuroscience, 2022: 6299435.
453. Zhang D, Liao M, Chen J, et al. Application of Nursing Intervention Based on Intelligent Grip Strength System in Patients with Tumor PICC: A Case-Control Study on Promoting Functional Exercise and Quality of Life. Computational and Mathematical Methods in Medicine, 2022: 8016567.
454. Shen Y, Dai L, Zhu Y, et al. The Impact of Improved Oral Care Methods on the Oral Health of Patients Undergoing Transoral Mechanical Ventilation. Computational and Mathematical Methods in Medicine, 2022: 7596654.
455. Zeng R, Zhang X, Su L, et al. Effect Evaluation of Preoperative Psychological Nursing Intervention on Sinusitis Patients Undergoing General Anesthesia by Multiplanner Reformation-Based CT. Computational and Mathematical Methods in Medicine, 2022: 7516339.
456. Xu X, Gu F. Safety and Efficacy of Mild Moxibustion on Cancer-Related Fatigue in Non-Small-Cell Lung Cancer Patients Undergoing Chemotherapy. Computational and Mathematical Methods in Medicine, 2022: 6530454.

457. Liu J, Wang J, Guo Y, et al. The Efficacy of Psychological Care and Chinese Herbal Decoction in Postoperative Chemotherapy Patients with Endometrial Cancer. Journal of Healthcare Engineering, 2022: 5700637.
458. Niu Q, Sun H, Wu H, et al. Evaluation of the Quality and Effect of 360° Safe Indwelling Infusion of Peripheral Venous Indwelling Needle in Pediatric Clinic. Journal of Healthcare Engineering, 2022: 1499927.
459. Lan L, Zhu X, Ye B, et al. Effects of Individualized Nursing Based on Zero-Defect Theory on Perioperative Patients Undergoing Laparoscopic Cholecystectomy. Disease Markers, 2022: 5086350.
460. Qiu W, Bai F. Study on the Relationship between AAD and Clinical Features in Emergency Ward Patients and the Application Effect of Probiotics. Contrast Media & Molecular Imaging, 2022: 6164843.
461. Chen Z, Wang Y. Analysis of Interventional Application Effect of Ultrasound-Guided QLB and TAPB in the Treatment and Analgesia of Patients Undergoing Laparoscopic Colorectal Surgery. Contrast Media & Molecular Imaging, 2022: 4199868.
462. Liu M. Effects of Chinese Herbal Formula on Immune Function and Nutritional Status of Breast Cancer Patients. Computational and Mathematical Methods in Medicine, 2022: 5900024.
463. Li J, Kou Y, Zhang S, et al. Effect of Acupotomy Combined with Electroacupuncture Therapy on Finger Mobility and Pain Relief in Patients with Carpal Tunnel Syndrome. Computational and Mathematical Methods in Medicine, 2022: 2550875.
464. Wang Q, Xiao M, Sun H, et al. A Study on the Preventive Effect of Esketamine on Postpartum Depression (PPD) after Cesarean Section. Computational and mathematical methods in medicine, 2022: 1524198.
465. Yang X, Lv H, Jiang W, et al. Clinical Value Analysis of Xiaozheng Decoction Combined with Bladder Perfusion for Postoperative Treatment of Bladder Cancer and Its Effect on Serum miR-143 and miR-92a. Journal of Healthcare Engineering, 2022: 8177674.
466. Xie A, Zhang X, Ju F, et al. Effects of the Ultrasound-Guided Stellate Ganglion Block on Hemodynamics, Stress Response, and Gastrointestinal Function in Postoperative Patients with Colorectal Cancer. Computational Intelligence and Neuroscience, 2022: 2056969.
467. Xiang L, Liu W, Jin Y. Effect of Comprehensive Nursing on the Recovery of Gastrointestinal Function in Patients Undergoing Abdominal Operation. Computational Intelligence and Neuroscience, 2022: 1179321.
468. Lixian W, Yanfang Y, Chengzong C, et al. Application of Different Ventilation Modes Combined with AutoFlow Technology in Thoracic Surgery. Journal of Healthcare Engineering, 2022: 2507149.
469. Zhao Y. Effect Evaluation of Artificial Intelligence-Based Electronic Health PDCA Nursing Model in the Treatment of Mycoplasma Pneumonia in Children. Journal of Healthcare Engineering, 2022: 1956944.
470. Zhu Y, Pang G, Lu B, et al. Anesthetic Effect of Dexmedetomidine in Clinical Functional Neurosurgery. Disease Markers, 2022: 6000388.
471. Zhang S. Study on the Adjustment of Cervical Spondylopathy in Middle-Aged and Elderly People Based on CT Image Analysis. Contrast Media & Molecular Imaging, 2022: 2291835.
472. Chen S, He S. Analysis of Therapeutic Effect of Elderly Patients with Severe Heart Failure Based on LSTM Neural Model. Computational Intelligence and Neuroscience, 2022: 7250791.
473. Yang X, Zhao J G, Liu M, et al. Management of Patients with Cervicofacial Edema and Paresthesia during Perioperative Period of Transoral Endoscopic Thyroidectomy. Computational and Mathematical Methods in Medicine, 2022: 4775264.

474. Zheng X, Peng Y, Liu C, et al. Application of VIP Care in Patients with Advanced Tumors in the Western Region of China. BioMed Research International, 2022: 7834620.
475. Jiang S, Wang R, Zhang H. Integrated Learning Model-Based Assessment of Enteral Nutrition Support in Neurosurgical Intensive Care Patients. BioMed Research International, 2022: 4061043.
476. Zhang H, Yang J, Xiong Y. Influence of Nutritional Support Program on Gastrointestinal Function, Complication Rate, and Prognosis in Elderly Sufferers with CI. BioMed Research International, 2022: 3198272.
477. Dou Z, Xu Z, Wang Q, et al. Mosapride Citrate Combined with Divine Qu Disinfectant Oral Liquid for Children Function Dyspepsia and the Influence of Serum Factors. Journal of Healthcare Engineering, 2022: 3053277.
478. Li N, Li J, Gai P. Effects of Modified Wenjing Decoction Combined with Online Publicity and Education on the Treatment of Primary Dysmenorrhea of Cold Coagulation and Blood Stasis. Journal of Healthcare Engineering, 2022: 1899356.
479. Xiang Y, Xi L. Application of Quantitative Assessment Strategy-Based Nursing Combined with Empathic Nursing in Patients Undergoing Tension-Free Inguinal Herniorrhaphy. Evidence-Based Complementary and Alternative Medicine, 2022: 7897027.
480. Feng J, Zhang C, Chen H, et al. Shen-Ling-Bai-Zhu-San Enhances the Antipneumonia Effect of Cefixime in Children by Ameliorating Gut Microflora, Inflammation, and Immune Response. Evidence-Based Complementary and Alternative Medicine, 2022: 7752426.
481. Jin X, Liu X, Xie H, et al. Effect of Narrative Nursing on Family Resilience and Psychosocial Adaptation of Middle-Aged Patients with Breast Cancer. Evidence-Based Complementary and Alternative Medicine, 2022: 5499298.
482. Zhao Y, Wang C, Dai H. Effects of Rapid Rehabilitation Nursing Based on the Syndrome Differentiation and Treatment Theory of TCM on Sleep and Life Quality of Patients Undergoing Multi-Endoscope Gallbladder-Preserving Cholecystolithotomy. Evidence-Based Complementary and Alternative Medicine, 2022: 5339525.
483. Guo X, Sun L, Wang S, et al. Effects of Irrational Use of Antibiotics on Intestinal Health of Children with Extraintestinal Infectious Diseases. Contrast media & molecular imaging, 2022: 9506490.
484. Zhu W, Li Q, Huang J. The Effects of ERAS Concept Combined with Postoperative Leg Pad Elevation on Knee Enhancement, Quality of Life, and Pain in Sufferers after HTO Surgery. Contrast Media & Molecular Imaging, 2022: 8440977.
485. Yang J, Shi Y, Chen H. Clinical Efficacy of Topical Tacrolimus on Conjunctival Hyperemia Caused by Prostaglandin Analogues. Evidence-Based Complementary and Alternative Medicine, 2022: 3700720.
486. Li C Q, Xiang Y, Wang Y, et al. Study on Nano Drug Particles in the Diagnosis and Treatment of Alzheimer’s Disease in the Elderly. Bioinorganic Chemistry and Applications, 2022: 3335581.
487. Zhang C, Min K, Zhao H, et al. Study of CT-Guided Localization in Pulmonary Nodule Resection. Applied Bionics and Biomechanics, 2022: 3751400.
488. Guo L, Zeng J, Liu Z, et al. Observation of Curative Effect of Lung Recruitment in Patients with Acute Respiratory Distress Syndrome after Cardiopulmonary Bypass Surgery. Applied Bionics and Biomechanics, 2022: 2693500.
489. Lu X, Han H, Zhang Z B, et al. The Application Effect of Jiawei Sanyu Shengjing Decoction Combined with High Ligation of the Spermatic Vein in Varicocele Male Infertility Patients. Applied Bionics and Biomechanics, 2022: 2629140.
490. Zhao L, Yang J, Nie M, et al. The Application and Evaluation of Effective Quality Management by Objectives in Patient Care for Persistent Vacuum Sealing Drainage. Journal of Healthcare Engineering, 2021: 1234003.

491. Li X, Wang F, Li R, et al. Quality Care Alleviates Behavioral Cognitive Impairment and Reduces Complications in Elderly Patients with Cardiovascular and Cerebrovascular Diseases. Journal of Healthcare Engineering, 2022: 8958099.
492. Lin S, Wang S, Zhang J, et al. Efficacy of Jiedu Pingsou Decoction Combined with Azithromycin in the Treatment of Children with Mycoplasma Pneumonia and Its Effects on Inflammatory Factors and Immune Function. Journal of Healthcare Engineering, 2022: 9102727.
493. Shi J, Zhao G, Liu P, et al. Effects of Dexmedetomidine Combined with Intravenous Anesthesia on Oxidative Stress Index, Postoperative Sleep Quality, and Brain Function in HICH Patients. Journal of Healthcare Engineering, 2022: 5463986.
494. Dang J, Li J, Li H, et al. The Value of a Management Plan Based on Risk Factors for Cerebral Infarction Patients with Cerebral Hemorrhage. Journal of Healthcare Engineering, 2022: 8635487.
495. Chang R, Miao H, Cui A, et al. Clinical Effect of Nimodipine Combined with Magnesium Sulfate on Pregnancy-Induced Hypertension Syndrome. Journal of Healthcare Engineering, 2022: 7217543.
496. Yu X, Zhang N, Jin J, et al. Evaluation of Therapeutic Effect and Prognosis of Danzhi Xiaoyao Powder Combined with Photodynamic Therapy in the Treatment of Rose Acne. Computational and Mathematical Methods in Medicine, 2022: 1636839.
497. Liu Z, Jin Y, Feng C, et al. Renoprotective Effect of Intraoperative Dexmedetomidine in Renal Transplantation. Computational and Mathematical Methods in Medicine, 2022: 9275406.
498. Wang W, Sun H, Ye Y, et al. Influence and Effect of Acupoint Application of Chinese Medicine on Height and Bone Age of Children with Short Stature. Evidence-Based Complementary and Alternative Medicine, 2021: 7459593.
499. Hu L, Hu L, Xu J, et al. Efficacy of Respiratory Training in Relieving Postoperative Pain in Patients with Spinal Nerve Root Entrapment Syndrome. Computational and Mathematical Methods in Medicine, 2022: 7703835.
500. Wang Y, Xu H. Exploration of the Effect of Competence-Oriented Simulated Teaching Training on Comprehensive Competitiveness of Nursing Staff. Contrast Media & Molecular Imaging, 2022: 9505764.
501. Zhou L, Wang H, Yao S, et al. Efficacy of Human Adipose Derived Mesenchymal Stem Cells in Promoting Skin Wound Healing. Journal of Healthcare Engineering, 2022: 6590025.
502. Ma L, Pan Y, Wu Z, et al. Effect of Tegretol on Oxidative Stress, Serum Inflammatory Factors, and Left Ventricular Function in AMI Patients after Emergency PCI. Computational and Mathematical Methods in Medicine, 2022: 8929058.
503. Wan W, Zhou J, Lu R, et al. Clinical Efficacy of Huangkui Capsule plus Methylprednisolone in the Treatment of Nephropathy and the Effect on Urinary Protein and Serum Inflammatory Factors in Patients. Evidence-Based Complementary and Alternative Medicine, 2022: 6232264.
504. Yang W, Tian X, Liang J. A Comprehensive Nursing Model Combined with High-Quality Nursing Intervention for Antiviral Therapy in Patients with Chronic Hepatitis B. Evidence-Based Complementary and Alternative Medicine, 2022: 6244637.
505. Liu Y, Wang B, Zhang Q, et al. A Case-Control Study of Continuous Veno-Venous Hemofiltration Combined with Xuebijing Injection in the Treatment of Severe Sepsis. Contrast Media & Molecular Imaging, 2022: 7884508.
506. Zou Z, Liu K, Li Y, et al. The Application of the GP Model to Manage Controllable Risk Factors in Stroke Patients with Diabetes Can Effectively Improve the Prognosis and Reduce the Recurrence Rate. Evidence-Based Complementary and Alternative Medicine, 2022: 5413985.
507. Zeng X, Lin S, Li Y. Effects of Modified Duhuo Jisheng Decoction Combined with Arthroscopic Surgery on Bone Metabolism, Oxidative Stress, and Serum TLR4 and TGF-β1 in Patients with Knee Osteoarthritis. Journal of Environmental and Public Health, 2022: 1933504.

508. Liu W, Yin W. Effect of Uterine Artery Ligation and Uterine Artery Embolization on Postpartum Hemorrhage Due to Uterine Asthenia after Cesarean Section and Its Effect on Blood Flow and Function of Uterine and Ovarian Arteries. Journal of Healthcare Engineering, 2022: 1337234.
509. Zheng J, Jing Y J, Guo A H, et al. Effect of New Nursing Team Management Mode on Self-Efficacy, Compliance, and Quality of Life of Patients with Chronic Kidney Disease and Its Chain Mediating Effect. Contrast Media & Molecular Imaging, 2022: 2071893.
510. Zhong Z, Wang X, Xu K, et al. Clinical Efficacy of Retroauricular Injection of Methylprednisolone Sodium Succinate in the Treatment of Sudden Deafness with Type 2 Diabetes. Computational and Mathematical Methods in Medicine, 2022: 3097436.
511. Xu S, Yu Y, Liu H, et al. Application of Nursing Outcome-Oriented Integrated Zero-Defect Nursing Combined with Respiratory Function Training in Long-Term Bedridden Patients Undergoing Stroke. Evidence-Based Complementary and Alternative Medicine, 2022: 4425680.
512. Xie L, Li M. Analysis of Clinical Efficacy of Clearing Heat and Dispelling Paralysis Soup in the Treatment of Osteoarthritis of the Knee Joint and Its Effect on Patients' Motor Function. Computational and Mathematical Methods in Medicine, 2022: 5104121.
513. Wang J, Zhao Y, Xie F. Study on the Nursing Effect of Diabetes Health Education Nursing Methods Applied to Diabetes Patients in the Endocrinology Department. Journal of Healthcare Engineering, 2022:3363096.
514. Wang Z, Chen M, Wang C, et al. Stereotactic Aspiration Acts as an Effective Treatment for Malignant Middle Cerebral Artery Infarction. Journal of Healthcare Engineering, 2022: 4373404.
515. Jia Y, Wang Y, Yang K, et al. Effect of Minimally Invasive Puncture Drainage and Conservative Treatment on Prognosis of Patients with Cerebral Hemorrhage. Journal of Healthcare Engineering, 2021:2401256.
516. Weng H, Li Q. Effect of Core Stability Training on Correction and Surface Electronic Signals of Paravertebral in Adolescent Idiopathic Scoliosis. BioMed Research International, 2022: 1819606.
517. Hua H, Zhang B, Wang X, et al. Diffusion Tensor Imaging Observation of Frontal Lobe Multidirectional Transcranial Direct Current Stimulation in Stroke Patients with Memory Impairment. Journal of Healthcare Engineering, 2022: 2545762.
518. Liu L, Yang Y. Nutritional Management Mode of Early Cardiac Rehabilitation in Patients with Stanford Type A Aortic Dissection. Computational and Mathematical Methods in Medicine, 2022: 2124636.
519. Liu B, Xiao C. RETRACTED: Application analysis of scenario simulation teaching method combined with progressive teaching mode in clinical teaching of orthopaedic nursing. International Journal of Electrical Engineering & Education, 2023, 60(1_suppl): 3911-3922.
520. Miao X, Tao L, Huang L, et al. Application of Laparoscopy Combined with Enhanced Recovery after Surgery (ERAS) in Acute Intestinal Obstruction and Analysis of Prognostic Factors: A Retrospective Cohort Study. BioMed Research International, 2022: 5771526.
521. Li L, Jin X, Cong W, et al. Acupuncture in the Treatment of Parkinson's Disease with Sleep Disorders and Dose Response. BioMed Research International, 2022: 7403627.
522. Wu G, Qu H. The Effect of Calisthenics on Hypoglycemic of Diabetic Patients. BioMed Research International, 2022: 7737626.
523. Xu L, Han X, Li L. Effect of Carrying out Continuous Nursing Based on Mobile Platform on the Life of Children with Leukemia after Discharge from Hospital. BioMed Research International, 2022: 7300303.
524. Yang Q, Zhang J, Li J. Clinical Effect of the Guizhi Shaoyao Zhimu Decoction in the Treatment of Hyperuricemia. BioMed Research International, 2022: 5186210.
525. Li Y, Zhao C, Xiong C, et al. Clinical Effect of Nicorandil Combined with Aspirin in the Treatment of Myocardial Ischemia. BioMed Research International, 2022: 2214411.

526. He T, Gong L. Clinical Effect of Microneedle Injection Combined with Blood Transfusion in the Treatment of Severe Anemia Complicated with Vitiligo under Regenerative Medical Technology. BioMed Research International, 2022: 7117627.
527. Meng X, Ren M, Zhuang Y, et al. Application Experience and Patient Feedback Analysis of 3D Printed AFO with Different Materials: A Random Crossover Study. BioMed Research International, 2021: 8493505.
528. He L, Chen X, Zhang Y. Clinical Effect of Hufu Copper Scraping on Shoulder-Hand Syndrome after Stroke. Emergency Medicine International, 2022: 9165141.
529. Zhu Y, Li J, Yu S, et al. Clinical Comparative Study of Intravitreal Injection of Triamcinolone Acetonide and Aflibercept in the Treatment of Diabetic Retinopathy Cystoid Macular Edema. Emergency medicine international, 2022: 1348855.
530. Liu S, Li G. Analysis of the Effect of Music Therapy Interventions on College Students with Excessive Anxiety. Occupational Therapy International, 2023: 3351918.
531. Mao L, Lu H, Lu Y. Effect of Nursing Model Based on Rosenthal Effect on Self-Efficacy and Cognition of Life Meaning in Patients with Non-Small-Cell Lung Cancer. Emergency Medicine International, 2022: 6730024.
532. Zhou Y, Yang D, Gu W. Efficacy and Mechanism of Trimebutine Maleate Combined with Lactulose in the Treatment of Constipation-Predominant Irritable Bowel Syndrome in the Elderly. Emergency Medicine International, 2022: 6125120.
533. Wu K P, Yin H A, Du A M. Effects of Modified Jianpi Qushi Heluo Decoction on Scores of TCM Syndromes, 24 h Urinary Albumin, and Plasma Albumin in IMN of Spleen-Kidney Qi Deficiency. Emergency Medicine International, 2022: 6061709.
534. Zhang Y, Lu X, Ji H, et al. Effects of Deep Hyperthermia Combined with Intraperitoneal Chemotherapy on Liver-Kidney Function, Immune Function, and Long-Term Survival in Patients with Abdominal Metastases. Emergency Medicine International, 2023: 5878402.
535. Hu J, Zhou L, Ding J. Application of Chain Nursing Process in the Nursing of Elderly Inpatients with Implantable Venous Infusion Port. Emergency Medicine International, 2022: 5496533.
536. Wang N, Wei L, Xiong R, et al. Application of Diversified Health Education Combined with Psychological Nursing in the Treatment of Patients with Infectious Bone Defects by Induction Membrane Surgery. Emergency Medicine International, 2022: 4987816.
537. Zhou Y, Qian Y, Xiong W, et al. Effect of Transcatheter Arterial Chemoembolization Combined with Radiofrequency Ablation on Liver Function and Immune Function in Patients with Hepatocellular Carcinoma. Emergency Medicine International, 2022: 4842370.
538. Xiong R, Wang N, He J X. Effects of Mind Mapping Combined with Microvideo Explanation on Disease Perception Control and Nursing Cooperation during Membrane Induction Therapy in Patients with Infectious Nonunion after Tibial Trauma. Emergency Medicine International, 2022: 4439595.
539. Xiao H, Ye J. Application of Health Education Based on Phased Transition Theory Model in Continuous Nursing for Patients with Inflammatory Bowel Disease. Emergency Medicine International, 2022: 4194178.
540. Sun N, Chen W, Wu Y, et al. Curative Effect of Yangxin Dingji Capsule Combined With Mexiletine Hydrochloride on Postoperative Arrhythmia and Its Influences on the Vascular Endothelial Function in Coronary Bifurcation Lesions. Emergency Medicine International, 2022: 4078895.
541. Farooq A, Bilgrami A, Faraz S A, et al. Comparative evaluation of Sapindus Mukorossi extract with 17% EDTA on smear layer removal in various parts of the tooth root. Journal of Applied Biomaterials & Functional Materials, 2023, 21: 22808000231166210.
542. Khokhar S, Sindhu N, Mirdha B R. Comparison of topical 0.3% ofloxacin to fortified tobramycin–cefazolin in the therapy of bacterial keratitis. Infection, 2000, 28: 149-152.

543. Zhou X, Jin T, Wang L, et al. Clinical practice of epidermal growth factor receptor-tyrosine kinase inhibitor targeted drugs combined with gadolinium oxide nanoparticles in the treatment of non-small cell lung cancer. Bioengineered. 2022;13(1):128-139.
544. Ali MK, Emam SM, Abdel-Aleem MA, et al. Misoprostol versus expectant management in women with incomplete first-trimester miscarriage after failed primary misoprostol treatment: A randomized clinical trial. Int J Gynaecol Obstet. 2021;154(3):558-564.
545. Rahmani E, Jamilian M, Samimi M, et al. The effects of coenzyme Q10 supplementation on gene expression related to insulin, lipid and inflammation in patients with polycystic ovary syndrome. Gynecol Endocrinol. 2018;34(3):217-222.
546. Knothe C, Boldt J, Hempelmann G.Influence of prophylactic nifeclipine on troponin-T levels in cardiac surgery. Journal of Cardiothoracic and Vascular Anesthesia, 1994, 8(3): 166.
547. Boldt J, Zickmann B, Ballesteros M, et al. Does the preparation of heparin influence anticoagulation during cardiopulmonary bypass?. Journal of cardiothoracic and vascular anesthesia, 1991, 5(5): 449-453.
548. Frenn M, Salzman N, Lam V, et al.Body Mass Index and Gut Microbiome: A Cluster-Randomized, Controlled, Pilot Feasibility Study. Child Obes. 2023.
549. Hasan A, Alsharawneh A, Alasamee N. Evaluation of the impact of a self-stigma reduction programme on psychosocial outcomes among people with schizophrenia spectrum disorder. Mental Health and Social Inclusion, 2024, 28(4): 358-367.
550. Boldt J, Kling D, Weidler B, et al. Acute preoperative hemodilution in cardiac surgery: volume replacement with a hypertonic saline-hydroxyethyl starch solution. Journal of cardiothoracic and vascular anesthesia, 1991, 5(1): 23-28.
551. Agarwal A, Barik S, Upadhyay A,et al. Pain behavior of children with bilateral idiopathic clubfoot undergoing Ponseti casting and the effect of non-pharmaceutical pain-relieving agents. Arch Orthop Trauma Surg. 2024;144(2):985.
552. Abdallah AA. Evaluation of the risk of postcesarean endometritis with preoperative vaginal preparation with povidone–iodine: a randomized controlled study. Middle East Fertil Soc J. 2015;20:246–50.
553. Abdallah MS, Mosalam EM, Zidan A-AA, et al. The antidiabetic metformin as an adjunct to antidepressants in patients with major depressive disorder: a proof-of-concept, randomized, double-blind, placebo-controlled trial. Neurotherapeutics. 2020;17:1897–906.
554. AbdElaal NK, Ellakwa HE, Elhalaby AF, et al. Scalpel versus diathermy skin incision in Caesarean section. J Obstet Gynaecol. 2019;39(3):340-344.
555. Abdelazim IA, Makhlouf HH. Sequential clomiphene citrate/hMG versus hMG for ovulation induction in clomiphene citrate-resistant women. Arch Gynecol Obstet. 2013;287:591–7.
556. Abdelghany AM, Kamel SB. The effect of prednisolone and/or acyclovir in relation to severity of Bell's palsy at presentation. Egyptian Journal of Ear, Nose, Throat and Allied Sciences. 2013;14(3):155-159
557. Abdelkefi A, Ladeb S, Torjman L, et al. Single autologous stem-cell transplantation followed by maintenance therapy with thalidomide is superior to double autologous transplantation in multiple myeloma: results of a multicenter randomized clinical trial. Blood. 2008;111:1805–10.
558. Abdelmageed WM, Elquesny KM, Shabana RI, et al. Analgesic properties of a dexmedetomidine infusion after uvulopalatopharyngoplasty in patients with obstructive sleep apnea. Saudi J Anaesth. 2011;5(2):150-156.
559. Abd-Elsalam S, Esmail ES, Khalaf M, et al. Hydroxychloroquine in the treatment of COVID-19: a multicenter randomized controlled study. Am J Trop Med Hyg. 2020;103:1635–9.
560. Abd-Elsalam S, Salama M, Soliman S, et al. Remdesivir efficacy in COVID-19 treatment: a randomized controlled trial. Am J Trop Med Hyg. 2021;106:886–90.

561. Aboelela MA, Kandeel AR, Elsayed U, et al. Dexmedetomidine in a surgically inserted catheter for transversus abdominis plane block in donor hepatectomy: a prospective randomized controlled study. Saudi J Anaesth. 2018;12:297–303.
562. Abou Zeid H, Al-Gahamdi A, Abdul-Hadi M. Dolasetron decreases postoperative nausea and vomiting after breast surgery. Breast J. 2002;8(4):216-221.
563. Abou-Elenain K. Study of the systemic and pulmonary oxidative stress status during exposure to propofol and sevoflurane anaesthesia during thoracic surgery. Eur J Anaesthesiol. 2010;27:566–71.
564. Aboumatar H, Naqibuddin M, Chung S, et al. Effect of a program combining transitional care and long-term self-management support on outcomes of hospitalized patients with chronic obstructive pulmonary disease: a randomized clinical trial. JAMA. 2018;320:2335–43.
565. Abou-Raya A, Abou-Raya S, HELMII M. The Effect of vitamin D supplementation on inflammatory and hemostatic markers and disease activity in patients with systemic lupus erythematosus: a randomized placebo-controlled trial. J Rheumatol. 2013;40:265–72.
566. Abou-Raya A, Abou-Raya S, Khadrawi T, et al. Effect of low-dose oral prednisolone on symptoms and systemic inflammation in older adults with moderate to severe knee osteoarthritis: a randomized placebo-controlled trial. J Rheumatol. 2014;41:53–9.
567. Abramson JS, O'Shea TM, Ratledge DL, et al. Development of a vaccine tracking system to improve the rate of age-appropriate primary immunization in children of lower socioeconomic status. J Pediatr. 1995;126(4):583-586.
568. Abu Hashim H, Mashaly AM, Badawy A. Letrozole versus laparoscopic ovarian diathermy for ovulation induction in clomiphene-resistant women with polycystic ovary syndrome: A randomized controlled trial. Arch Gynecol Obstet. 2010;282:567–71.
569. Abu Hashim H, Shokeir T, Badawy A. Letrozole versus combined metformin and clomiphene citrate for ovulation induction in clomiphene-resistant women with polycystic ovary syndrome: a randomized controlled trial. Fertil Steril. 2010;94:1405–9.
570. Aceves-Martins M, Llauradó E, Tarro L, et al. A school-based, peer-led, social marketing intervention to engage spanish adolescents in a healthy lifestyle (‘We are cool’-som la pera study): a parallel-cluster randomized controlled study. Child Obes. 2017;13:300–13.
571. Aflatoonian A, Oskouian H, Ahmadi S, et al. Can fresh embryo transfers be replaced by cryopreserved-thawed embryo transfers in assisted reproductive cycles? A randomized controlled trial. J Assist Reprod Genet. 2010;27:357–63.
572. Ahimastos AA, Aggarwal A, D’Orsa KM, et al. Effect of perindopril on large artery stiffness and aortic root diameter in patients with Marfan syndrome: a randomized controlled trial. Jama. 2007;298:1539–47.
573. Ahimastos AA, Aggarwal A, Savarirayan R, et al. A role for plasma transforming growth factor-beta and matrix metalloproteinases in aortic aneurysm surveillance in Marfan syndrome? . Atherosclerosis. 2010;209(1):211-214.
574. Ahimastos AA, Dart AM, Lawler A, et al. Reduced arterial stiffness may contribute to angiotensin-converting enzyme inhibitor induced improvements in walking time in peripheral arterial disease patients. J Hypertens. 2008;26:1037–42.
575. Ahimastos AA, Latouche C, Natoli AK, et al. Potential vascular mechanisms of ramipril induced increases in walking ability in patients with intermittent claudication. Circ Res. 2014;114:1144–55.
576. Ahimastos AA, Lawler A, Reid CM, et al. Brief communication: ramipril markedly improves walking ability in patients with peripheral arterial disease: a randomized trial. Ann Intern Med. 2006;144(9):660-664.
577. Ahimastos AA, Natoli AK, Lawler A, et al. Ramipril reduces large-artery stiffness in peripheral arterial disease and promotes elastogenic remodeling in cell culture. Hypertension. 2005;45:1194–9.

578. Ahimastos AA, Walker PJ, Askew C, et al. Effect of ramipril on walking times and quality of life among patients with peripheral artery disease and intermittent claudication. JAMA. 2013;309:453-60.
579. Ahmad J, Lynch MK, Maltenfort M. Comparison of screws to plate-and-screw constructs for midfoot arthrodesis. Foot Ankle Int. 2018;39:922–9.
580. Albada A, van Dulmen S, Bensing JM, et al. Effects of a pre-visit educational website on information recall and needs fulfilment in breast cancer genetic counselling, a randomized controlled trial. Breast Cancer Res. 2012;14(2):R37
581. Alessi J, Becker AS, Amaral B, et al. Type 1 diabetes and the challenges of emotional support in crisis situations: results from a randomized clinical trial of a multidisciplinary teleintervention. Sci Rep. 2022;12:3086
582. Ali IM, Netongo PM, Atogho-Tiedeu B, et al. Amodiaquine-Artesunate versus Artemether-Lumefantrine against Uncomplicated Malaria in Children Less Than 14 Years in Ngaoundere, North Cameroon: Efficacy, Safety, and Baseline Drug Resistant Mutations in pfcrt, pfmdr1, and pfdhfr Genes. Malar Res Treat. 2013;2013:234683.
583. Ali Z, Anjum A, Khurshid L, et al. Evaluation of low-cost custom made VAC therapy compared with conventional wound dressings in the treatment of non-healing lower limb ulcers in lower socio-economic group patients of Kashmir valley. J Orthop Surg Res. 2015;10:183.
584. Aliyev NA, Aliyev ZN. Lamotrigine in the immediate treatment of outpatients with depersonalization disorder without psychiatric comorbidity: randomized, double-blind, placebo-controlled study. J Clin Psychopharmacol. 2011;31:61–5.
585. Alshehri FM. Comparative study of pineapple juice as a negative oral contrast agent in magnetic resonance cholangiopancreatography. J Clin Diagn Res. 2015;9(1):TC13-TC16.
586. Altay Y, Demirok G, Balta O, et al. Azithromycin 1.5% Ophthalmic Solution for Blepharitis Treatment: Comparison of 14- Versus 30-Day Treatment. J Ocul Pharmacol Ther. 2017;33(6):498.
587. Amin SM. Evaluation of gabapentin and dexamethasone alone or in combination for pain control after adenotonsillectomy in children. Saudi J Anaesth. 2014;8(3):317-322.
588. An J, Lee J-H, Kim E, et al. Comparison of sugammadex and pyridostigmine bromide for reversal of rocuronium-induced neuromuscular blockade in short-term pediatric surgery: a prospective randomized study. Medicine (Baltimore). 2020;99:e19130.
589. Andruchshishin I, Sapiev S, Denisenko Y, et al. Improving the physical qualities of professional boxers by considering their personality traits. Humanit Soc Sci Commun. 2022;9:321
590. Aref NK. Does timing of urinary catheter removal after elective cesarean section affects postoperative morbidity?: a prospective randomized trial. J Matern Neonatal Med. 2020;33:3141–6.
591. Arora A, Bansal A, Purkait B, et al. Transurethral resection of prostate and bleeding: a prospective randomized, double-blind, placebo-controlled trial to see efficacy of short-term use of finasteride and dutasteride on operative blood loss and prostatic microvessel density. J Endourol. 2017 Sep;31(9):910-917.
592. Asemi Z, Hashemi T, Karamali M, et al. Effects of vitamin D supplementation on glucose metabolism, lipid concentrations, inflammation, and oxidative stress in gestational diabetes: a double-blind randomized controlled clinical trial. Am J Clin Nutr. 2013;98:1425–32.
593. Asemi Z, Karamali M, Jamilian M, et al. Magnesium supplementation affects metabolic status and pregnancy outcomes in gestational diabetes: a randomized, double-blind, placebo-controlled trial. Am J Clin Nutr. 2015;102:222–9.
594. Asemi Z, Samimi M, Tabassi Z, et al. Vitamin D supplementation affects serum high-sensitivity C-reactive protein, insulin resistance, and biomarkers of oxidative stress in pregnant women. J Nutr. 2013;143:1432–8.

595. Asgari SA, Safarinejad MR, Poorreza F, et al. The effect of parenteral testosterone administration prior to hypospadias surgery: a prospective, randomized and controlled study. J Pediatr Urol. 2015;11:143.e1-143.e6.
596. Aviles A, Cleto S. Low-dose radiotherapy (2×2 g) versus low doses and rituximab in the treatment of marginal zone b-cell lymphoma previously untreated. Leuk Res. 2020;98:106443.
597. Avilés A, Fernández RA, Perez FR, et al. Adjuvant radiotherapy in stage IV diffuse large cell lymphoma improves outcome. Leuk Lymphoma. 2004;45:1385–9.
598. Avilès A, Nambo MJ, Calva A, et al. Adjuvant radiotherapy in patients with diffuse large B-cell lymphoma in advanced stage (III/IV) improves the outcome in the rituximab era. Hematology. 2019;24(1):521-525.
599. Avilés A, Neri N, Fernández RA, et al. Randomized clinical trial to assess the efficacy of radiotherapy in primary mediastinal large B-Lymphoma. Int J Radiat Oncol Biol Phys. 2012;83:1227–31.
600. Ba MC, Long H, Zhang XL, et al. Laparoscopic hyperthermic intraperitoneal perfusion chemotherapy for patients with malignant ascites secondary to unresectable gastric cancer. Surg Laparosc Endosc Percutaneous Tech. 2020;30:55–61.
601. Badawy A, Abdel Aal I, Abulatta M. Clomiphene citrate or letrozole for ovulation induction in women with polycystic ovarian syndrome: a prospective randomized trial. Fertil Steril. 2009;92:849–52.
602. Badawy A, Elnashar A, Totongy M. Clomiphene citrate or aromatase inhibitors for superovulation in women with unexplained infertility undergoing intrauterine insemination: a prospective randomized trial. Fertil Steril. 2009;92(4):1355-1359.
603. Badawy A, Gibreal A. Clomiphene citrate versus tamoxifen for ovulation induction in women with PCOS: a prospective randomized trial. Eur J Obstet Gynecol Reprod Biol. 2011;159:151–4.
604. Badawy A, Khiary M, Ragab A, et al. Laparoscopy--or not--for management of unexplained infertility. J Obstet Gynaecol. 2010;30(7):712-715.
605. Badawy A, Mosbah A, Shady M. Anastrozole or letrozole for ovulation induction in clomiphene-resistant women with polycystic ovarian syndrome: a prospective randomized trial. Fertil Steril. 2008 May;89(5):1209-1212.
606. Badawy AM, Khiary M, Sherif LS, et al. Low-molecular weight heparin in patients with recurrent early miscarriages of unknown aetiology. J Obstet Gynaecol (Lahore). 2008;28:280–4.
607. Bae I-L, Kim Y-S, Hur M-H. The effects of listening to healing beat music on adults' recovery from exposure to stressful stimuli: a randomized controlled trial. Integr Med Res. 2022;11:100753.
608. Baek J, Tae KS, Lee A, et al. Effective lens position according to incision width in cataract surgery using phacoemultification and posterior chamber lens implantation. Semin Ophthalmol. 2018;33:846–51.
609. Banerjee D, Das A, Majumdar S, et al. PONV in Ambulatory surgery: a comparison between ramosetron and ondansetron: a prospective, double-blinded, and randomized controlled study. Saudi J Anaesth. 2014;8:25–9.
610. Bansal P, Gupta A, Mongha R, et al. Laparoscopic versus open pyeloplasty: comparison of two surgical approaches- a single centre experience of three years. J Minim Access Surg. 2008;4:76-9.
611. Bao L. Intervention Value of Path-Type Health Education on Cognition and Renal Function of Patients with Diabetic Nephropathy. Comput Math Methods Med. 2021;2021:3665460.
612. Barausse C, Pistilli R, Canullo L,et al. A 5-year randomized controlled clinical trial comparing 4-mm ultrashort to longer implants placed in regenerated bone in the posterior atrophic jaw. Clin Implant Dent Relat Res. 2022;24(1):4-12.

613. Barbalho M, Coswig V, Raiol R, et al. Single joint exercises do not provide benefits in performance and anthropometric changes in recreational bodybuilders. Eur J Sport Sci. 2020;20(1):72-79.
614. Barbalho M, Coswig VS, Steele J, et al. Evidence for an Upper Threshold for Resistance Training Volume in Trained Women. Med Sci Sports Exerc. 2019;51(3):515-522.
615. Barch DM, Whalen D, Gilbert K, et al. Neural Indicators of Anhedonia: Predictors and mechanisms of treatment change in a randomized clinical trial in early childhood depression. Biol Psychiatry. 2019;85:863–71.
616. Bartoc C, Frumento RJ, Jalbout MI, et al. A randomized, double-blind, placebo-controlled study assessing the anti-inflammatory effects of ketamine in cardiac surgical patients. J Cardiothorac Vasc Anesth. 2006;20:217–22.
617. Behtash N, Ghaemaghami F, Gilani MM, et al. To peritonealise or not to peritonealise? A randomised trial at abdominal hysterectomy in Iran. J Obstet Gynaecol (Lahore). 2001;21:520–4.
618. Bernstein AM, Roizen MF, Martinez L. Purified palmitoleic acid for the reduction of high-sensitivity C-reactive protein and serum lipids: a double-blinded, randomized, placebo controlled study. J Clin Lipidol. 2014;8:612–7.
619. Bezwoda WR, Seymour L, Dansey RD. High-dose chemotherapy with hematopoietic rescue as primary treatment for metastatic breast cancer: a randomized trial. J Clin Oncol. 1995;13:2483-9
620. Bhar D, Roybasunia S, Das A, et al. A comparison between intrathecal clonidine and neostigmine as an adjuvant to bupivacaine in the subarachnoid block for elective abdominal hysterectomy operations: a prospective, double-blind and randomized controlled study. Saudi J Anaesth. 2016;10:121–6.
621. Bhardwaj P, Godatwar PK, Charan J, et al. Efficacy and safety of Ayurveda intervention AYUSH 64 as add-on therapy for patients with COVID 19 infections: An open labelled, parallel group, randomized controlled clinical trial. medRxiv preprint, 2021: 2021.08.
622. Bhatia T, Bhatia J, Attri JP, et al. Intrathecal dextmedetomidine to reduce shoulder tip pain in laparoscopic cholecystectomies under spinal anesthesia. Anesth Essays Res. 2015;9(3):320-325.
623. Bhimani R, Bhimani F, Singh RB, et al. Enhanced conventional method is as precise as navigation for distal femur resection during total knee replacement: a randomized controlled trial. F1000Research. 2019;8:717.
624. Bilgen H, Özek E, Cebeci D, et al. Comparison of sucrose, expressed breast milk, and breast-feeding on the neonatal response to heel prick. J Pain. 2001;2:301–5.
625. Bilgili D, Yilmaz S, Dumani A, et al. Postoperative pain after irrigation with Vibringe versus a conventional needle: a randomized controlled trial. Int Endod J. 2016;49:813.
626. Biltagi M Al, Baset AA, Bassiouny M, et al. Omega-3 fatty acids, vitamin C and Zn supplementation in asthmatic children: a randomized self-controlled study. Acta Paediatr Int J Paediatr. 2009;98:737–42.
627. Biswas S, Verma R, Bhatia VK, et al. Comparison between Thoracic Epidural Block and Thoracic Paravertebral Block for Post Thoracotomy Pain Relief. J Clin Diagn Res. 2016;10(9):UC08-UC12.
628. Bittner V, Wenger NK, Waters DD, et al. Vitamin D levels do not predict cardiovascular events in statin-treated patients with stable coronary disease. Am Heart J. 2012;164:387–93.
629. Boér K, Láng I, Juhos É, et al. Adjuvant therapy of breast cancer with docetaxel-containing combination (TAC) — a Hungarian experience in the BCIRG 001 trial. Pathol Oncol Res. 2003;9:166–9.

630. Boldt J, Brenner T, Lehmann A, et al. Influence of two different volume replacement regimens on renal function in elderly patients undergoing cardiac surgery: comparison of a new starch preparation with gelatin. Intensive Care Med. 2003;29:763–9.
631. Boldt J, Brosch C, Ducke M, et al. Influence of volume therapy with a modern hydroxyethylstarch preparation on kidney function in cardiac surgery patients with compromised renal function: a comparison with human albumin. Crit Care Med. 2007;35:2740–6.
632. Boldt J, Brosch C, Lehmann A, et al. Prophylactic use of pentoxifylline on inflammation in elderly cardiac surgery patients. Ann Thorac Surg. 2001;71:1524–9.
633. Boldt J, Brosch C, Lehmann A, et al. The prophylactic use of the beta-blocker esmolol in combination with phosphodiesterase III inhibitor enoximone in elderly cardiac surgery patients. Anesth Analg. 2004;99:1009–17.
634. Boldt J, Brosch C, Piper SN, et al. Influence of prophylactic use of pentoxifylline on postoperative organ function in elderly cardiac surgery patients. Crit Care Med. 2001;29:952–8.
635. Boldt J, Brosch C, Röhm K, et al. Is albumin administration in hypoalbuminemic elderly cardiac surgery patients of benefit with regard to inflammation, endothelial activation, and long-term kidney function? Anesth Analg. 2008;107:1496–503.
636. Boldt J, Brosch C, Suttner S, et al. Prophylactic use of the phospodiesterase III inhibitor enoximone in elderly cardiac surgery patients: effect on hemodynamics, inflammation, and markers of organ function. Intensive Care Med. 2002;28:1462–9.
637. Boldt J, Ducke M, Kumle B, et al. Influence of different volume replacement strategies on inflammation and endothelial activation in the elderly undergoing major abdominal surgery. Intensive Care Med. 2004;30:416–22.
638. Boldt J, Haisch G, Suttner S, et al. Are lactated ringer's solution and normal saline solution equal with regard to coagulation? Anesth Analg. 2002;94:378–84.
639. Boldt J, Haisch G, Suttner S, et al. Effects of a new modified, balanced hydroxyethyl starch preparation (Hextend®) on measures of coagulation. Br J Anaesth. 2002;89:722–8.
640. Boldt J, Heesen M, Müller M, et al. The effects of albumin versus hydroxyethyl starch solution on cardiorespiratory and circulatory variables in critically ill patients. Anesth Analg. 1996;83:254–61.
641. Boldt J, Heesen M, Padberg W, et al. The influence of volume therapy and pentoxifylline infusion on circulating adhesion molecules in trauma patients. Anaesthesia. 1996;51:529–35.
642. Boldt J, Heesen M, Welters I, et al. Does the type of volume therapy influence endothelial-related coagulation in the critically ill? Br J Anaesth. 1995;75:740–6.
643. Boldt J, Jaun N, Kumle B, et al. Economic considerations of the use of new anesthetics: a comparison of propofol, sevoflurane, desflurane, and isoflurane. Anesth Analg. 1998;86:504–9.
644. Boldt J, Kling D, Dietevich HA, et al. Drug interactions: the new phosphodiesterase inhibitor enoximone and the calcium channel blocker nifedipine in coronary surgery patients-influence on hemodynamics and plasma concentrations. J Cardiovasc Pharmacol. 1990;15:37–43.
645. Boldt J, Kling D, Herold C, et al. Volume therapy with hypertonic saline hydroxyethyl starch solution in cardiac surgery. Anaesthesia. 1990;45:928–34.
646. Boldt J, Kling D, von Bormann B, et al. Blood conservation in cardiac operations. Cell separation versus hemofiltration. J Thorac Cardiovasc Surg. 1989;97(6):832-840.
647. Boldt J, Kling D, Zickmann B, et al. Acute preoperative plasmapheresis and established blood conservation techniques. Ann Thorac Surg. 1990;50(1):62-68.
648. Boldt J, Kling D, Zickmann B, et al. Haemodynamic effects of the phosphodiesterase inhibitor enoximone in comparison with dobutamine in esmolol-treated cardiac surgery patients. Br J Anaesth. 1990;64:611–6.
649. Boldt J, Knothe C, Schindler E, et al. Volume replacement with hydroxyethyl starch solution in children. Br J Anaesth. 1993;70:661–5.

650. Boldt J, Knothe C, Welters I, et al. Normothermic versus hypothermic cardiopulmonary bypass: do changes in coagulation differ? Ann Thorac Surg. 1996;62:130–5.
651. Boldt J, Knothe C, Zickmann B, et al. Aprotinin in pediatric cardiac operations: platelet function, blood loss, and use of homologous blood. Ann Thorac Surg. 1993;55:1460–6.
652. Boldt J, Knothe C, Zickmann B, et al. Comparison of two aprotinin dosage regimens in pediatric patients having cardiac operations: influence on platelet function and blood loss. J Thorac Cardiovasc Surg. 1993;105:705–11.
653. Boldt J, Knothe C, Zickmann B, et al. Influence of different glucose-insulin-potassium regimes on glucose homeostasis and hormonal response in cardiac surgery patients. Anesth Analg. 1993;76:233–8.
654. Boldt J, Knothe C, Zickmann B, et al. Influence of different intravascular volume therapies on platelet function in patients undergoing cardiopulmonary bypass. Anesth Analg. 1993;76:1185–90.
655. Boldt J, Knothe C, Zickmann B, et al. Platelet function in cardiac surgery: influence of temperature and aprotinin. Ann Thorac Surg. 1993;55:652–8.
656. Boldt J, Knothe C, Zickmann B, et al. The role of enoximone in cardiac surgery. Br J Anaesth. 1992;69:45–50.
657. Boldt J, Mayer J, Brosch C, et al. Volume replacement with a balanced hydroxyethyl starch (HES) preparation in cardiac surgery patients. J Cardiothorac Vasc Anesth. 2010;24:399–407.
658. Boldt J, Menges T, Wollbrück M, et al. Continuous I.V. Administration of the angiotensin-converting enzyme inhibitor enalaprilat in the critically ill : effects on regulators of circulatory homeostasis. J Cardiovasc Pharmacol. 1995;25:416–23.
659. Boldt J, Mueller M, Menges T, et al. Influence of different volume therapy regimens on regulators of the circulation in the critically ill. Br J Anaesth. 1996;77:480–7.
660. Boldt J, Müller M, Heesen M, et al. Influence of different volume therapies on platelet function in the critically ill. Intensive Care Med. 1996;22:1075–81.
661. Boldt J, Müller M, Mentges D, et al. Volume therapy in the critically ill: is there a difference? Intensive Care Med. 1998;24:28–36.
662. Boldt J, Osmer C, Schindler E, et al. Circulating adhesion molecules in cardiac operations: influence of high-dose aprotinin. Ann Thorac Surg. 1995;59:100–5.
663. Boldt J, Papsdorf M, Piper SN, et al. Continuous heparinization and circulating adhesion molecules in the critically ill. Shock. 1999;11:13–8.
664. Boldt J, Papsdorf M, Piper SN, et al. Influence of dopexamine hydrochloride on haemodynamics and regulators of circulation in patients undergoing major abdominal surgery. Acta Anaesthesiol Scand. 1998;42:941–7.
665. Boldt J, Papsdorf M, Uphus D, et al. Changes in regulators of the circulation in patients undergoing lung surgery. Br J Anaesth. 1997;79:733–9.
666. Boldt J, Piper S, Uphus D, et al. Preoperative microbiologic screening and antibiotic prophylaxis in pulmonary resection operations. Ann Thorac Surg. 1999;68:208–11.
667. Boldt J, Schindler E, Härter K, et al. Influence of intravenous administration of angiotensin-converting enzyme inhibitor enalaprilat on cardiovascular mediators in cardiac surgery patients. Anesth Analg. 1995;80:480–5.
668. Boldt J, Schindler E, Osmer CH, et al. Influence of different anticoagulation regimens on platelet function during cardiac surgery. Br J Anaesth. 1994;73:639–44.
669. Boldt J, Schindler E, Welters I, et al. The effect of the anticoagulation regimen on endothelial-related coagulation in cardiac surgery patients. Anaesthesia. 1995;50:954–60.
670. Boldt J, Schölhorn T, Mayer J, et al. The value of an albumin-based intravascular volume replacement strategy in elderly patients undergoing major abdominal surgery. Anesth Analg. 2006;103:191–9.

671. Boldt J, Suttner S, Brosch C, et al. Cardiopulmonary bypass priming using a high dose of a balanced hydroxyethyl starch versus an albumin-based priming strategy. Anesth Analg. 2009;109:1752–62.
672. Boldt J, Suttner S, Brosch C, et al. Influence on coagulation of a potato-derived hydroxethylstarch (HES 130/0.42) and a maize-derived hydroxethylstarch (HES 130/0.4) in patients undergoing cardiac surgery. Br J Anaesth. 2009;102:191–7.
673. Boldt J, Suttner S, Brosch C, et al. The influence of a balanced volume replacement concept on inflammation, endothelial activation, and kidney integrity in elderly cardiac surgery patients. Intensive Care Med. 2009;35:462–70.
674. Boldt J, von Bormann B, Kling D, et al. Preoperative plasmapheresis in patients undergoing cardiac surgery procedures. Anesthesiology. 1990;72:282–8.
675. Boldt J, Zickmann B, Ballesteros M, et al. Cardiorespiratory responses to hypertonic saline solution in cardiac operations. Ann Thorac Surg. 1991;51:610–5.
676. Boldt J, Zickmann B, Czeke A, Het al. Blood conservation techniques and platelet function in cardiac surgery. Anesthesiology. 1991;75(3):426-432.
677. Boldt J, Zickmann B, Dapper F, Hempelmann G. Does the technique of cardiopulmonary bypass affect lung water content?. Eur J Cardiothorac Surg. 1991;5(1):22-26.
678. Boldt J, Zickmann B, Fedderson B, et al. Six different hemofiltration devices for blood conservation in cardiac surgery. Ann Thorac Surg. 1991;51:747–53.
679. Boldt J, Zickmann B, Herold C, et al. Influence of hypertonic volume replacement on the microcirculation in cardiac surgery. Br J Anaesth. 1991;67:595–602.
680. Boldt J, Zickmann B, M BMB, et al. Influence of five different priming solutions on platelet function in patients undergoing cardiac surgery. Anesth Analg. 1992;74:219–25.
681. Boldt J, Zickmann B, Rapin J, et al. Influence of volume replacement with different HES-solutions on microcirculatory blood flow in cardiac surgery. Acta Anaesthesiol Scand. 1994;38(5):432-438.
682. Boldt J, Zickmann B, Schindler E, et al. Influence of aprotinin on the thrombomodulin/protein C system in pediatric cardiac operations. J Thorac Cardiovasc Surg. 1994;107:1215–21.
683. Boldt J. Endothelial-related coagulation in pediatric surgery. Ann Thorac Surg. 1998;65:S56–9.
684. Bolli R, Chugh AR, D'Amario D, et al. Cardiac stem cells in patients with ischaemic cardiomyopathy (SCIPIO): initial results of a randomised phase 1 trial. Lancet. 2011;378:1847–57.
685. Bonnefoi H, Potti A, Delorenzi M, et al. Validation of gene signatures that predict the response of breast cancer to neoadjuvant chemotherapy: a substudy of the EORTC 10994/BIG 00-01 clinical trial. Lancet Oncol. 2007;8(12):1071-1078.
686. Boonsiriseth K, Latt MM, Kiattavorncharoen S, et al. Dexamethasone injection into the pterygomandibular space in lower third molar surgery. Int J Oral Maxillofac Surg. 2017;46(7):899-904.
687. Bottini LP, Ricci L, Piattelli A, et al. Bucco-lingual crestal bone changes around implants immediately placed in fresh extraction sockets in association or not with porcine bone: a non-blinded randomized controlled trial in humans. J Periodontol. 2012;1–8.
688. Braun Janzen T, Paneduro D, Picard L, et al. A parallel randomized controlled trial examining the effects of rhythmic sensory stimulation on fibromyalgia symptoms. PLoS One. 2019;14:e0212021.
689. Breuning SE, Ferguson DG, Davidson NA, et al. Effects of thioridazine on the intellectual performance of mentally retarded drug responders and nonresponders. Arch Gen Psychiatry. 1983;40(3):309-313.
690. Brownstone S, Connor A, Stein D. Improving measles vaccine uptake rates in Nigeria: an RCT evaluating the impact of incentive sizes and reminder calls on vaccine uptake. PLoS One. 2020;15:e0233149.

691. Burns T, White SJ, Catty J. Individual Placement and Support in Europe: the EQOLISE trial. Int Rev Psychiatry. 2008;20(6):498-502.
692. Cai R, Fei Y, Li X, et al. Research on the Effect of 5Why-Based Nursing Intervention in Blood Purification Nursing. J Healthc Eng. 2021;2021:6535238.
693. Cao J, Shi X, Xu H, et al. Effects of premedication with clonidine on pre-operative anxiety and post-operative pain in children: a prospective, randomised, controlled trial. Eur J Anaesthesiol. 2010; 27: 000-000.
694. Caro-Tarragó A, Olona-Casas C, Olona-Cabases M, et al. Impact on quality of life of using an onlay mesh to prevent incisional hernia in midline laparotomy: a randomized clinical trial. J Am Coll Surg. 2014;219:470–9.
695. Chan MF, Ko CY. Osteoporosis prevention education programme for women. J Adv Nurs. 2006;54(2):159-170.
696. Chan MF. A randomised controlled study of the effects of music on sleep quality in older people. J Clin Nurs. 2011;20:979–87.
697. Chandra RK, Puri S, Hamed A. Influence of maternal diet during lactation and use of formula feeds on development of atopic eczema in high risk infants. Br Med J. 1989;298:228–30.
698. Chandra RK. Effect of vitamin and trace-element supplementation on cognitive function in elderly subjects. Nutrition. 2001;17:709–12.
699. Chandra RK. Effect of vitamin and trace-element supplementation on immune responses and infection in elderly subjects. Lancet. 1992;340:1124–7.
700. Chandrasekar S, Jeyakumar S, Ganapathy T. Glue versus suture for mesh fixation in inguinal hernia repair. Int J Surg. 2018;S1743-9191(18)30636-8.
701. Chanimov M, Evron S, Haitov Z, et al. Accidental venous and dural puncture during epidural analgesia in obese parturients (BMI > 40 kg/m(2)): three different body positions during insertion. J Clin Anesth. 2010;22:614–8.
702. Chen X, Zhang H, Zhong J, et al. Comparison of indirect pulp treatment and iRoot BP Plus pulpotomy in primary teeth with extremely deep caries: a prospective randomized trial. Clin Oral Investig. 2021;25:3067–76.
703. Chen Y, Zhang Y, Zhu Y-L, et al. Efficacy and safety of an intra-operative intra-articular magnesium/ropivacaine injection for pain control following total knee arthroplasty. J Int Med Res. 2009;37:1733–41.
704. Cheng B-Q, Jia C-Q, Liu C-T, et al. Chemoembolization combined with radiofrequency ablation for patients with hepatocellular carcinoma larger than 3 cm. JAMA. 2008;299:1669-77.
705. Cheng X, Zhang M, Fang Q, et al. Opioid-sparing effect of modified intercostal nerve block during single-port thoracoscopic lobectomy: Retraction: A randomised controlled trial. Eur J Anaesthesiol. 2021 Sep 9.
706. Cheng XQ, Mei B, Zuo YM, et al. A multicentre randomised controlled trial of the effect of intra-operative dexmedetomidine on cognitive decline after surgery. Anaesthesia. 2019;74(6):741-750.
707. Cho DR, Lee SH. Effects of virtual reality immersive training with computerized cognitive training on cognitive function and activities of daily living performance in patients with acute stage stroke: A preliminary randomized controlled trial. Medicine (Baltimore). 2019;98(11):e14752.
708. Cho YS, Seo E, Han J-H, et al. Comparison of midazolam alone versus midazolam plus propofol during endoscopic submucosal dissection. Clin Endosc. 2011;44:22-6.
709. Ci R, Qin Y, Ci C, et al. Application Evaluation of High-Flow Humidified Oxygen in Patients with Respiratory Failure after General Anesthesia Extubation for Multiple Injuries. J Healthc Eng. 2021;2021:1387129.
710. Colzato LS, Steenbergen L, Sellaro R. The effect of gamma-enhancing binaural beats on the control of feature bindings. Exp Brain Res. 2017;235(7):2125-2131.
711. Compagna R, Aprea G, De Rosa D, et al. Fast track for elderly patients: is it feasible for colorectal surgery? Int J Surg. 2014;12:S20–2.

712. Conte A, Ghiraldini B, Denófrio PHF, et al. Could implant position influence the peri-implant parameters in edentulous mandibles of diabetics rehabilitated with overdentures? A split-mouth randomized study. Int J Oral Maxillofac Surg. 2021 Mar 12:S0901-5027(21)00088-6.
713. Cui M, Zhao D, Wang H, et al. Effects of Incontro, Alleanza, Responsabilita, Autonomia Intervention Model Combined with Orem Self-Care Model and the Use of Smart Wearable Devices on Perceived Stress and Self-Efficacy in Patients after Total Hip Arthroplasty. Comput Intell Neurosci. 2022;2022:5780084.
714. Cui SL, Yu CY, Tee YW, et al. Acupuncture compared to conscious sedation for pain relief during in-vitro fertilization oocyte retrieval. Med Acupunct. 2020;32:e411–8.
715. Cunningham S, Kinsey JD. Pharmacist-Led Discharge Medication Counseling and its Corresponding Impact on Medication Adherence and Hospital Readmission Rates. Innov Pharm. 2020;11(3):10.24926/iip.v11i3.3352.
716. Dabbous HM, Abd-Elsalam S, El-Sayed MH, et al. Efficacy of favipiravir in COVID-19 treatment: a multi-center randomized study. Arch Virol. 2021;166:949–54.
717. Dabbous HM, El-Sayed MH, El Assal G, et al. Safety and efficacy of favipiravir versus hydroxychloroquine in management of COVID-19: a randomised controlled trial. Sci Rep. 2021;11:7282.
718. Dai Y, Liu S-X, Ye L, et al. Clinical efficacy of ultrasound-mediated transdermal lidocaine and capsaicin delivery for the treatment of allodynia caused by herpes zoster. Pain Med. 2020;21:3739–46.
719. Dammerman R, Kim S, Adera M, et al. A Phase 1, Open-Label, Single Dose Pharmacokinetic Study in Stabilized Patients with Schizophrenia Following Risperidone Implant. Psychopharmacol Bull. 2017;47(4):36-40.
720. Dammerman R, Kim S, Adera M, et al. A Phase-1, 6-Month Open-Label, Dose-Ranging Pharmacokinetic Study in Stabilized Patients with Schizophrenia Following Risperidone Implant. Psychopharmacol Bull. 2017;47(4):29-35.
721. Darabos N, Trsek D, Miklic D, et al. Comparison of double-bundle anterior cruciate ligament reconstruction with and without autologous conditioned serum application. Knee Surg Sports Traumatol Arthrosc. 2016;24(10):3377.
722. Darwish A, Kamel M, Zahran K, et al. Office cervicoscopy versus stationary colposcopy in suspicious cervix: a randomized controlled trial. J Midlife Health. 2019;10:115–22.
723. Darwish AM, Fouly HA, Saied WH, et al. Lactoferrin plus health education versus total dose infusion (TDI) of low-molecular weight (LMW) iron dextran for treating iron deficiency anemia (IDA) in pregnancy: a randomized controlled trial. J Matern Neonatal Med. 2019;32:2214–20.
724. Darwish AM, Khalifa EE, Rashad E, et al. Total dose iron dextran infusion versus oral iron for treating iron deficiency anemia in pregnant women: a randomized controlled trial. J Matern Neonatal Med. 2017;32:398–403.
725. Darwish AM, Metwally A, Shaaban MM, et al. Monopolar versus bipolar laparoscopic ovarian drilling in clomiphene-resistant polycystic ovaries (PCO): a preliminary study. Gynecological Surgery.2016;13:179-185.
726. Das A, Chattopadhyay S, Mandal D, et al. Does the preoperative administration of tranexamic acid reduce perioperative blood loss and transfusion requirements after head neck cancer surgery? A randomized, controlled trial. Anesth Essays Res. 2015;9:384-90.
727. Das A, Chhaule S, Bhattacharya S, et al. Controlled hypotension in day care functional endoscopic sinus surgery: a comparison between esmolol and dexmedetomidine: a prospective, double-blind, and randomized study. Saudi J Anaesth. 2016;10:276–82.
728. Das A, Dutta S, Chattopadhyay S, et al. Pain relief after ambulatory hand surgery: a comparison between dexmedetomidine and clonidine as adjuvant in axillary brachial plexus block: a prospective, double-blinded, randomized controlled study. Saudi J Anaesth. 2016;10:6–12.

729. Das A, Halder S, Chattopadhyay S, et al. Effect of two different doses of dexmedetomidine as adjuvant in bupivacaine induced subarachnoid block for elective abdominal hysterectomy operations: a prospective, double-blind, randomized controlled study. Oman Med J. 2015;30:257–63.
730. Das A, Majumdar S, Halder S, et al. Effect of dexmedetomidine as adjuvant in ropivacaine-induced supraclavicular brachial plexus block: a prospective, double-blinded and randomized controlled study. Saudi J Anaesth. 2014;8:S72–7.
731. Das A, Majumdar S, Kundu R, et al. Pain relief in day care arthroscopic knee surgery: a comparison between intra-articular ropivacaine and levobupivacaine: a prospective, double-blinded, randomized controlled study. Saudi J Anaesth. 2014;8:368–73.
732. Das A, Mukherjee A, Chhaule S, et al. Induced hypotension in ambulatory functional endoscopic sinus surgery: a comparison between dexmedetomidine and clonidine as premedication. A prospective, double-blind, and randomized study. Saudi J Anaesth. 2016;10:74–80.
733. De Hert SG, Lorsomradee S, vanden Eede H, et al. A randomized trial evaluating different modalities of levosimendan administration in cardiac surgery patients with myocardial dysfunction. J Cardiothorac Vasc Anesth. 2008;22:699–705.
734. Deekshith SRK, Reddy KJ, Raviteja R. Patelloplasty in total knee arthroplasty with circumpatellar denervation versus without denervation – a randomized prospective study. Arthroplasty. 2020;2:26
735. Dehkordi AH, Heydarnejad MS. Effect of booklet and combined method on parents' awareness of children with beta-thalassemia major disorder. J Pak Med Assoc. 2008;58(11):659.
736. Demiroglu H, Özcebe OI, Barista I, et al. Interferon alfa-2b, colchicine, and benzathine penicillin versus colchicine and benzathine penicillin in Behçet’s disease: a randomised trial. Lancet. 2000;355:605–9.
737. Derosa G, Bonaventura A, Bianchi L, et al. Comparison of vildagliptin and glimepiride: effects on glycaemic control, fat tolerance and inflammatory markers in people with type 2 diabetes. Diabet Med. 2014;31:1515–23.
738. Derosa G, Bonaventura A, Romano D, et al. Enalapril/lercanidipine combination on markers of cardiovascular risk: a randomized study. J Am Soc Hypertens. 2014;8:422–8.
739. Derosa G, Cicero AF, Carbone A, et al. Results from a 12 months, randomized, clinical trial comparing an olmesartan/amlodipine single pill combination to olmesartan and amlodipine monotherapies on blood pressure and inflammation. Eur J Pharm Sci. 2014;51:26-33.
740. Derosa G, Cicero AFG, Carbone A, et al. Different aspects of sartan + calcium antagonist association compared to the single therapy on inflammation and metabolic parameters in hypertensive patients. Inflammation. 2014;37:154–62.
741. Derosa G, Cicero AFG, Carbone A, et al. Effects of an olmesartan/amlodipine fixed dose on blood pressure control, some adipocytokines and interleukins levels compared with olmesartan or amlodipine monotherapies. J Clin Pharm Ther. 2013;38:48–55.
742. Derosa G, Cicero AFG, Carbone A, et al. Evaluation of safety and efficacy of a fixed olmesartan/amlodipine combination therapy compared to single monotherapies. Expert Opin Drug Saf. 2013;12:621–9.
743. Derosa G, Cicero AFG, Carbone A, et al. Variation of some inflammatory markers in hypertensive patients after 1 year of olmesartan/amlodipine single-pill combination compared with olmesartan or amlodipine monotherapies. J Am Soc Hypertens. 2013;7:32–9.
744. Derosa G, Maffioli P, D’Angelo A, et al. Acarbose on insulin resistance after an oral fat load: a double-blind, placebo controlled study. J Diabetes Complications. 2011;25:258–66.
745. Derosa G, Maffioli P, Ferrari I, et al. Effects of one year treatment of sibutramine on insulin resistance parameters in type 2 diabetic patients. J Pharm Pharm Sci. 2010;13:378–90.

746. Derosa G, Ragonesi PD, Carbone A, et al. Evaluation of the positive effects on insulin-resistance and β-cell measurements of vildagliptin in addition to metformin in type 2 diabetic patients. Pharmacol Res. 2013;73:20–6.
747. DeTayrac R, Deffieux X, Droupy S, et al. A prospective randomized trial comparing tension-free vaginal tape and transobturator suburethral tape for surgical treatment of stress urinary incontinence. Am J Obstet Gynecol. 2004;190:602–8.
748. Doi MY, Tano SS, Schultz AR, et al. Effectiveness of acupuncture in individuals with tinnitus: randomized controlled trial. Braz J Otorhinolaryngol. 2015 Nov 14:S1808-8694(15)00206-2.
749. Dong P, Zhong CB. Visual Darkness Reduces Perceived Risk of Contagious-Disease Transmission From Interpersonal Interaction. Psychol Sci. 2018;29(7):1049-1061.
750. Dong W, Zhang G, Qu F. Effects of Ringer's sodium pyruvate solution on serum tumor necrosis factor- and interleukin-6 upon septic shock. Pak J Med Sci. 2015;31(3):672-677.
751. Dong Y, Zhang P, Fan L. Recognition of Factors of Postoperative Complications of Knee Osteoarthritis Patients and Comprehensive Nursing Intervention. Comput Math Methods Med. 2021;2021:1840613.
752. Döra Ö, Büyük ET. Effect of white noise and lullabies on pain and vital signs in invasive interventions applied to premature babies. Pain Manag Nurs. 2021;22:724–9.
753. Egenti NT, Ede MO, Nwokenna EN, et al. Randomized controlled evaluation of the effect of music therapy with cognitive-behavioral therapy on social anxiety symptoms. Medicine (Baltimore). 2019;98(32):e16495.
754. El-Bendary M, Abd-Elsalam S, Elbaz T, et al. Efficacy of combined sofosbuvir and daclatasvir in the treatment of COVID-19 patients with pneumonia: a multicenter Egyptian study. Expert Rev Anti Infect Ther. 2022;20:291–5.
755. Elgazzar A, Eltaweel A, Youssef SA, et al. Efficacy and safety of ivermectin for treatment and prophylaxis of COVID-19 pandemic. Res Sq. 2020;3:1–9.
756. El-Haggar SM, Hegazy SK, Abd-Elsalam SM, et al. A potential role of ethosuximide and pentoxifylline in relieving abdominal pain in irritable bowel syndrome patients treated with mebeverine: a randomized, double-blind, placebo-controlled trial. J Inflamm Res. 2022;15:1159–72.
757. El-Khayat W, Elsadek M, Saber W. Comparing the effect of office hysteroscopy with endometrial scratch versus office hysteroscopy on intrauterine insemination outcome: a randomized controlled trial. Eur J Obstet Gynecol Reprod Biol. 2015;194:96–100.
758. El-refaie W, Abdelhafez MS, Badawy A. Vaginal progesterone for prevention of preterm labor in asymptomatic twin pregnancies with sonographic short cervix: a randomized clinical trial of efficacy and safety. Arch Gynecol Obstet. 2016;293:61–7.
759. Erdur B, Ozturk Y, Gurbuz ED, et al. Comparison of sequential and standard therapy for Helicobacter pylori eradication in children and investigation of clarithromycin resistance. J Pediatr Gastroenterol Nutr. 2012;55:530–3.
760. Estruch R, Martínez-González MA, Corella D, et al. Effect of a high-fat Mediterranean diet on bodyweight and waist circumference: a prespecified secondary outcomes analysis of the PREDIMED randomised controlled trial. Lancet Diabetes Endocrinol. 2016;4:666–76.
761. Estruch R, Ros E, Salas-Salvadó J, et al. Primary prevention of cardiovascular disease with a Mediterranean diet. N Engl J Med. 2013;368:1279–90.
762. Fang J, Keeler CL, Chen L. Effect of acupuncture and Chinese herbal medicine on subacute stroke outcomes: a single center randomized controlled trial. Acupunct Med. 2018;36:345.
763. Fang X, Zhang J, Zhao J, et al. Effect of Resveratrol Combined with Donepezil Hydrochloride on Inflammatory Factor Level and Cognitive Function Level of Patients with Alzheimer's Disease. J Healthc Eng. 2022;2022:9148650.

764. Farrokhian A, Raygan F, Bahmani F, et al. Long-term vitamin D supplementation affects metabolic status in vitamin D–deficient type 2 diabetic patients with coronary artery disease. J Nutr. 2017;147:384–9.
765. Farsi F, Ebrahimi-Daryani N, Golab F, et al. A randomized controlled trial on the coloprotective effect of coenzyme Q10 on immune-inflammatory cytokines, oxidative status, antimicrobial peptides, and microRNA-146a expression in patients with mild-to-moderate ulcerative colitis. Eur J Nutr. 2021;60:3397–410.
766. Finfer S, Norton R, Bellomo R, et al. The SAFE study: saline vs. albumin for fluid resuscitation in the critically ill. Vox Sang. 2004;87:123–31.
767. Fleming RM, Fleming MR, Harrington GM, et al. Long-term health effects of the three major diets under self-management with advice, yields high adherence and equal weight loss, but very different long-term cardiovascular health effects as measured by myocardial perfusion imaging and specific markers of inflammatory coronary artery disease. Clin Cardiol. 2018;41(12):1620.
768. Fougère B, Goisser S, Cantet C, et al. Omega-3 fatty acid levels in red blood cell membranes and physical decline over 3 years: longitudinal data from the MAPT study. Geroscience. 2017;39(4):429-437.
769. Fujii Y, Itakura M. A comparison of pretreatment with fentanyl and lidocaine preceded by venous occlusion for reducing pain on injection of propofol: a prospective, randomized, double-blind, placebo-controlled study in adult Japanese surgical patients. Clin Ther. 2009;31:2107–12.
770. Fujii Y, Itakura M. A prospective, randomized, double-blind, placebo-controlled study to assess the antiemetic effects of midazolam on postoperative nausea and vomiting in women undergoing laparoscopic gynecologic surgery. Clin Ther. 2010;32:1633–7.
771. Fujii Y, Itakura M. Antiemetic efficacy of low-dose midazolam in patients undergoing thyroidectomy. Otolaryngol - Head Neck Surg. 2011;144:206–9.
772. Fujii Y, Itakura M. Comparison of lidocaine, metoclopramide, and flurbiprofen axetil for reducing pain on injection of propofol in Japanese adult surgical patients: a prospective, randomized, double-blind, parallel-group, placebo-controlled study. Clin Ther. 2008;30:280–6.
773. Fujii Y, Itakura M. Comparison of propofol, droperidol, and metoclopramide for prophylaxis of postoperative nausea and vomiting after breast cancer surgery: a prospective, randomized, double-blind, placebo-controlled study in Japanese patients. Clin Ther. 2008;30:2024–9.
774. Fujii Y, Itakura M. Efficacy of the lidocaine/flurbiprofen axetil combination for reducing pain during the injection of propofol. Minerva Anestesiol. 2011;77:693–7.
775. Fujii Y, Itakura M. Low-dose propofol to prevent nausea and vomiting after laparoscopic surgery. Int J Gynecol Obstet. 2009;106:50–2.
776. Fujii Y, Itakura M. Pretreatment with flurbiprofen axetil, flurbiprofen axetil preceded by venous occlusion, and a mixture of flurbiprofen axetil and propofol in reducing pain on injection of propofol in adult Japanese surgical patients: a prospective, randomized, double-blind, placebo-controlled study. Clin Ther. 2009;31:721–7.
777. Fujii Y, Itakura M. Reduction of postoperative nausea, vomiting, and analgesic requirement with dexamethasone for patients undergoing laparoscopic cholecystectomy. Surg Endosc. 2010;24:692–6.
778. Fujii Y, Kihara S, Takahashi S, et al. Calcium channel blockers attenuate cardiovascular responses to tracheal extubation in hypertensive patients. Can J Anesth Can d'anesthésie. 1998;45:655-9.
779. Fujii Y, Nakayama M, Nakano M. Propofol alone and combined with dexamethasone for the prevention of postoperative nausea and vomiting in adult Japanese patients having third molars extracted. Br J Oral Maxillofac Surg. 2008;46:207–10.
780. Fujii Y, Nakayama M. A lidocaine/metoclopramide combination decreases pain on injection of propofol. Can J Anesth Can d'anesthésie. 2005;52:474–7.
781. Fujii Y, Nakayama M. Dexamethasone for reduction of nausea, vomiting and analgesic use after gynecological laparoscopic surgery. Int J Gynaecol Obstet. 2008,100(1):27-30.

782. Fujii Y, Nakayama M. Effects of dexamethasone in preventing postoperative emetic symptoms after total knee replacement surgery: a prospective, randomized, double-blind, vehicle-controlled trial in adult Japanese patients. Clin Ther. 2005;27:740–5.
783. Fujii Y, Nakayama M. Efficacy of lignocaine plus ketamine at different doses in the prevention of pain due to propofol injection. Clin Drug Investig. 2005;25:537–42.
784. Fujii Y, Nakayama M. Influence of age on flurbiprofen axetil requirements for preventing pain on injection of propofol in japanese adult surgical patients: a prospective, randomized, double-blind, vehicle-controlled, parallel-group, dose-ranging study. Clin Ther. 2006;28:1116–22.
785. Fujii Y, Nakayama M. Prevention of pain due to injection of propofol with IV administration of lidocaine 40 mg + metoclopramide 2.5, 5, or 10 mg or saline: a randomized, double-blind study in Japanese adult surgical patients. Clin Ther. 2007;29:856–61.
786. Fujii Y, Nakayama M. Prevention of postoperative nausea and vomiting with a small dose of propofol alone and combined with dexamethasone in patients undergoing laparoscopic cholecystectomy: a prospective, randomized, double-blind study. Surg Endosc. 2008;22:1268–71.
787. Fujii Y, Nakayama M. Reduction of postoperative nausea and vomiting and analgesic requirement with dexamethasone in women undergoing general anesthesia for mastectomy. Breast J. 2007;13:564–7.
788. Fujii Y, Nakayama M. Reduction of propofol-induced pain through pretreatment with lidocaine and/or flurbiprofen. Clin Drug Investig. 2004;24:749–53.
789. Fujii Y, Numazaki M. Dose-range effects of propofol for reducing emetic symptoms during cesarean delivery. Obstet Gynecol. 2002;99:75-9.
790. Fujii Y, Numazaki M. Randomized, double-blind comparison of subhypnotic-dose propofol alone and combined with dexamethasone for emesis in parturients undergoing cesarean delivery. Clin Ther. 2004;26:1286–91.
791. Fujii Y, Saitoh Y, Kobayashi N. Prevention of vomiting after tonsillectomy in children: granisetron versus ramosetron. Laryngoscope. 2001;111:255–8.
792. Fujii Y, Saitoh Y, Takahashi S, et al. Combined diltiazem and lidocaine reduces cardiovascular responses to tracheal extubation and anesthesia emergence in hypertensive patients. Can J Anesth Can d'anesthésie. 1999;46:952–6.
793. Fujii Y, Saitoh Y, Takahashi S, et al. Diltiazem-lidocaine combination for the attenuation of cardiovascular responses to tracheal intubation in hypertensive patients. Can J Anesth Can d'anesthésie. 1998;45:933–7.
794. Fujii Y, Saitoh Y, Tanaka H, et al. Anti-emetic efficacy of prophylactic granisetron compared with perphenazine for the prevention of post-operative vomiting in children. Eur J Anaesthesiol. 1999;16:304–7.
795. Fujii Y, Saitoh Y, Tanaka H, et al. Anti-emetic efficacy of prophylactic granisetron, droperidol and metoclopramide in the prevention of nausea and vomiting after laparoscopic cholecystectomy: a randomized, double-blind, placebo-controlled trial. Eur J Anaesthesiol. 1998;15:166–71.
796. Fujii Y, Saitoh Y, Tanaka H, et al. Cardiovascular responses to tracheal extubation or LMA removal in children. Can J Anaesth. 1998;45(2):178-181.
797. Fujii Y, Saitoh Y, Tanaka H, et al. Combination of granisetron and droperidol for the prevention of vomiting after paediatric strabismus surgery. Pediatr Anesth. 1999;9:329–33.
798. Fujii Y, Saitoh Y, Tanaka H, et al. Combination of granisetron and droperidol in the prevention of nausea and vomiting after middle ear surgery. J Clin Anesth. 1999;11(2):108-112.
799. Fujii Y, Saitoh Y, Tanaka H, et al. Comparison of granisetron and droperidol in the prevention of vomiting after strabismus surgery or tonsillectomy in children. Pediatr Anesth. 1998;8:241–4.
800. Fujii Y, Saitoh Y, Tanaka H, et al. Comparison of ramosetron and granisetron for preventing postoperative nausea and vomiting after gynecologic surgery. Anesth Analg. 1999;89:476–9.

801. Fujii Y, Saitoh Y, Tanaka H, et al. Effective dose of granisetron for the prevention of post-operative nausea and vomiting in patients undergoing laparoscopic cholecystectomy. Eur J Anaesthesiol. 1998;15:287–91.
802. Fujii Y, Saitoh Y, Tanaka H, et al. Granisetron/dexamethasone combination for reducing nausea and vomiting during and after spinal anesthesia for cesarean section. Anesth Analg. 1999;88:1346–50.
803. Fujii Y, Saitoh Y, Tanaka H, et al. Granisetron/dexamethasone combination for the prevention of postoperative nausea and vomiting after laparoscopic cholecystectomy. Eur J Anaesthesiol. 2000;17:64–8.
804. Fujii Y, Saitoh Y, Tanaka H, et al. Preoperative oral antiemetics for reducing postoperative vomiting after tonsillectomy in children: granisetron vs perphenazine. Anesth Analg. 1999;88:1298–301.
805. Fujii Y, Saitoh Y, Tanaka H, et al. Pretreatment with oral clonidine attenuates cardiovascular responses to tracheal extubation in children. Pediatr Anesth. 2000;10:65–7.
806. Fujii Y, Saitoh Y, Tanaka H, et al. Prevention of PONV with granisetron, droperidol or metoclopramide in patients with postoperative emesis. Can J Anaesth. 1998;45:153–6.
807. Fujii Y, Saitoh Y, Tanaka H, et al. Prevention of post-operative nausea and vomiting with combined granisetron and droperidol in women undergoing thyroidectomy. Eur J Anaesthesiol. 1999;16:688-91.
808. Fujii Y, Saitoh Y, Tanaka H, et al. Prevention of postoperative vomiting with granisetron in paediatric patients with and without a history of motion sickness. Pediatr Anesth. 1999;9:527–30.
809. Fujii Y, Saitoh Y, Tanaka H, et al. Prophylactic antiemetic therapy with granisetron in women undergoing thyroidectomy. Br J Anaesth. 1998;81(4):526-528.
810. Fujii Y, Saitoh Y, Tanaka H, et al. Prophylactic antiemetic therapy with granisetron-droperidol combination in patients undergoing laparoscopic cholecystectomy. Can J Anesth Can d'anesthésie. 1998;45:541–4.
811. Fujii Y, Saitoh Y, Tanaka H, et al. Prophylactic oral antiemetics for preventing postoperative nausea and vomiting: granisetron vs domperidone. Anesth Analg. 1998;87:1404–7.
812. Fujii Y, Saitoh Y, Tanaka H, et al. Prophylactic therapy with combined granisetron and dexamethasone for the prevention of post-operative vomiting in children. Eur J Anaesthesiol. 1999;16:376–9.
813. Fujii Y, Saitoh Y, Tanaka H, et al. Ramosetron for preventing postoperative nausea and vomiting in women undergoing gynecological surgery. Anesth Analg. 2000;90:472–5.
814. Fujii Y, Saitoh Y, Tanaka H, et al. Ramosetronvs granisetron for the prevention of postoperative nausea and vomiting after laparoscopic cholecystectomy. Can J Anesth Can d'anesthésie. 1999;46:991–3.
815. Fujii Y, Shiga Y. Age-related differences in metoclopramide requirement for pain on injection of propofol. Clin Drug Investig. 2006;26:639–44.
816. Fujii Y, Shiga Y. Flurbiprofen axetil preceded by venous occlusion in the prevention of pain on propofol injection in the hand: a prospective, randomized, double-blind, vehicle-controlled, dose-finding study in Japanese adult surgical patients. Clin Ther. 2005;27:588–93.
817. Fujii Y, Shiga Y. Influence of aging on lidocaine requirements for pain on injection of propofol. J Clin Anesth. 2006;18:526–9.
818. Fujii Y, Tanaka H, Ito M. A randomized clinical trial of a single dose of ramosetron for the prevention of vomiting after strabismus surgery in children: A Dose-Ranging Study. Arch Ophthalmol. 2005;123:25-8.

819. Fujii Y, Tanaka H, Ito M. Ramosetron compared with granisetron for the prevention of vomiting following strabismus surgery in children. Br J Ophthalmol. 2001;85(6):670-672.
820. Fujii Y, Tanaka H, Kawasaki T. A comparison of granisetron, droperidol, and metoclopramide in the treatment of established nausea and vomiting after breast surgery: a double-blind, randomized, controlled trial. Clin Ther. 2003;25:1142–9.
821. Fujii Y, Tanaka H, Kawasaki T. A randomized, double-blind comparison of granisetron alone and combined with dexamethasone for post-laparoscopic cholecystectomy emetic symptoms. Curr Ther Res - Clin Exp. 2003;64:514–21.
822. Fujii Y, Tanaka H, Kawasaki T. Effects of granisetron in the treatment of nausea and vomiting after laparoscopic cholecystectomy: a dose-ranging study. Clin Ther. 2004;26:1055–60.
823. Fujii Y, Tanaka H, Kawasaki T. Preoperative oral granisetron for the prevention of postoperative nausea and vomiting after breast surgery. Eur J Surg. 2001;167:184–7.
824. Fujii Y, Tanaka H, Kawasaki T. Prophylaxis with oral granisetron for the prevention of nausea and vomiting after laparoscopic cholecystectomy: a prospective randomized study. Arch Surg. 2001;136:101–4.
825. Fujii Y, Tanaka H, Kawasaki T. Randomized clinical trial of granisetron, droperidol and metoclopramide for the treatment of nausea and vomiting after laparoscopic cholecystectomy. Br J Surg. 2000;87:285–8.
826. Fujii Y, Tanaka H, Kimura N. Prevention of nausea and vomiting after middle ear surgery: granisetron versus ramosetron. Laryngoscope. 1999;109:1988–90.
827. Fujii Y, Tanaka H, Kobayashi N. Granisetron, droperidol, and metoclopramide for preventing postoperative nausea and vomiting after thyroidectomy. Laryngoscope. 1999;109:664–7.
828. Fujii Y, Tanaka H, Kobayashi N. Granisetron/dexamethasone combination for the prevention of postoperative nausea and vomiting after thyroidectomy. Anaesth Intensive Care. 2000;28:266–9.
829. Fujii Y, Tanaka H, Kobayashi N. Prevention of postoperative nausea and vomiting with antiemetics in patients undergoing middle ear surgery: comparison of a small dose of propofol with droperidol or metoclopramide. Arch Otolaryngol - Head Neck Surg. 2001;127:25–8.
830. Fujii Y, Tanaka H, Somekawa Y. A randomized, double-blind, placebo-controlled trial of ramosetron for preventing nausea and vomiting during termination of pregnancy. Int J Obstet Anesth. 2004;13:15–8.
831. Fujii Y, Tanaka H, Somekawa Y. Granisetron, droperidol, and metoclopramide for the treatment of established postoperative nausea and vomiting in women undergoing gynecologic surgery. Am J Obstet Gynecol. 2000;182:13–6.
832. Fujii Y, Tanaka H, Somekawa Y. Treatment of postoperative emetic symptoms with granisetron in women undergoing abdominal hysterectomy: a randomized, double-blind, placebo-controlled, dose-ranging study. Curr Ther Res - Clin Exp. 2004;65:321–9.
833. Fujii Y, Tanaka H, Toyooka H. Circulatory responses to laryngeal mask airway insertion or tracheal intubation in normotensive and hypertensive patients. Can J Anaesth. 1995;42:32–6.
834. Fujii Y, Tanaka H, Toyooka H. Effective dose of granisetron in the reduction of nausea and vomiting after breast surgery. Acta Anaesthesiol Scand. 1997;41:1167–70.
835. Fujii Y, Tanaka H, Toyooka H. Granisetron and dexamethasone provide more improved prevention of postoperative emesis than granisetron alone in children. Can J Anaesth. 1996;43:1229–32.

836. Fujii Y, Tanaka H, Toyooka H. Granisetron prevents nausea and vomiting during spinal anaesthesia for caesarean section. Acta Anaesthesiol Scand. 1998;42:312–5.
837. Fujii Y, Tanaka H, Toyooka H. Granisetron reduces incidence of nausea and vomiting after breast surgery. Acta Anaesthesiol Scand. 1997;41:746–9.
838. Fujii Y, Tanaka H, Toyooka H. Granisetron reduces postoperative nausea and vomiting throughout menstrual cycle. Can J Anaesth. 1997;44:489–93.
839. Fujii Y, Tanaka H, Toyooka H. Granisetron reduces the incidence and severity of nausea and vomiting after laparoscopic cholecystectomy. Can J Anaesth. 1997;44:396-400
840. Fujii Y, Tanaka H, Toyooka H. Granisetron reduces vomiting after strabismus surgery and tonsillectomy in children. Can J Anaesth. 1996;43:35–8.
841. Fujii Y, Tanaka H, Toyooka H. Granisetron-dexamethasone combination reduces postoperative nausea and vomiting. Can J Anaesth. 1995;42:387–90.
842. Fujii Y, Tanaka H, Toyooka H. Optimal anti-emetic dose of granisetron for preventing post-operative nausea and vomiting. Can J Anaesth. 1994;41:794–7.
843. Fujii Y, Tanaka H, Toyooka H. Preoperative oral granisetron prevents postoperative nausea and vomiting. Acta Anaesthesiol Scand. 1998;42:653–7.
844. Fujii Y, Tanaka H, Toyooka H. Prevention of nausea and vomiting in female patients undergoing breast surgery: a comparison with granisetron, droperidol, metoclopramide and placebo. Acta Anaesthesiol Scand. 1998;42:220–4.
845. Fujii Y, Tanaka H, Toyooka H. Prevention of nausea and vomiting with granisetron, droperidol and metoclopramide during and after spinal anaesthesia for caesarean section: a randomized, double-blind, placebo-controlled trial. Acta Anaesthesiol Scand. 1998;42:921–5.
846. Fujii Y, Tanaka H, Toyooka H. Prevention of postoperative nausea and vomiting with granisetron: a randomized, double-blind comparison with droperidol. Can J Anaesth. 1995;42(10):852-856.
847. Fujii Y, Tanaka H, Toyooka H. Prophylactic antiemetic efficacy of granisetron in patients with and without previous postoperative emesis. Can J Anaesth. 1997;44:273–7.
848. Fujii Y, Tanaka H, Toyooka H. Prophylactic antiemetic therapy with droperidol in patients undergoing laparoscopic cholecystectomy. J Anesth. 1999;13:140–3.
849. Fujii Y, Tanaka H, Toyooka H. Prophylactic antiemetic therapy with granisetron-dexamethasone combination in women undergoing breast surgery. Acta Anaesthesiol Scand. 1998;42:1038–42.
850. Fujii Y, Tanaka H, Toyooka H. Reduction of postoperative nausea and vomiting with granisetron. Can J Anaesth. 1994;41(4):291-294.
851. Fujii Y, Tanaka H, Toyooka H. The effects of dexamethasone on antiemetics in female patients undergoing gynecologic surgery. Anesth Analg. 1997;85:913–7.
852. Fujii Y, Tanaka H. Comparison of granisetron and ramosetron for the prevention of nausea and vomiting after thyroidectomy. Clin Ther. 2002;24:766–72.
853. Fujii Y, Tanaka H. Double-blind, placebo-controlled, dose-ranging study of ramosetron for the prevention of nausea and vomiting after thyroidectomy. Clin Ther. 2002;24:1148–53.
854. Fujii Y, Tanaka H. Efficacy of granisetron for the treatment of postoperative nausea and vomiting in women undergoing breast surgery. Clin Drug Investig. 2006;26:203–8.
855. Fujii Y, Tanaka H. Granisetron reduces post-operative vomiting in children: a dose-ranging study. Eur J Anaesthesiol. 1999;16:62–5.
856. Fujii Y, Tanaka H. Granisetron versus granisetron/dexamethasone combination for the treatment of nausea, retching, and vomiting after major gynecologic surgery: a randomized, double-blind study. Clin Ther. 2003;25:507–14.
857. Fujii Y, Tanaka H. Preoperative oral granisetron for the prevention of vomiting following paediatric surgery. Pediatr Anesth. 2002;12:267–71.
858. Fujii Y, Tanaka H. Prevention of nausea and vomiting with ramosetron after total hip replacement. Clin Drug Investig. 2003;23:405–9.

859. Fujii Y, Tanaka H. Prophylactic therapy with granisetron in the prevention of vomiting after paediatric surgery. A randomized, double-blind comparison with droperidol and metoclopramide. Pediatr Anesth. 1998;8:149–53.
860. Fujii Y, Tanaka H. Randomized, double-blind, placebo-controlled, dosed-finding study of the antiemetic effects and tolerability of ramosetron in adults undergoing middle ear surgery. Clin Ther. 2003;25(12):3100-3108.
861. Fujii Y, Tanaka H. Results of a Prospective, Randomized, double-blind, placebo-controlled, dose-ranging trial to determine the effective dose of ramosetron for the prevention of vomiting after tonsillectomy in children. Clin Ther. 2003;25:3135–42.
862. Fujii Y, Toyooka H, Tanaka H. A granisetron-droperidol combination prevents postoperative vomiting in children. Anesth Analg. 1998;87:761–5.
863. Fujii Y, Toyooka H, Tanaka H. Antiemetic effects of granisetron on postoperative nausea and vomiting in patients with and without motion sickness. Can J Anaesth. 1996;43:110–4.
864. Fujii Y, Toyooka H, Tanaka H. Antiemetic efficacy of granisetron and metoclopramide in children undergoing ophthalmic or ENT surgery. Can J Anaesth. 1996;43:1095–9.
865. Fujii Y, Toyooka H, Tanaka H. Cardiovascular responses to tracheal extubation or LMA removal in normotensive and hypertensive patients. Can J Anaesth. 1997;44(10):1082-1086.
866. Fujii Y, Toyooka H, Tanaka H. Effective dose of granisetron for preventing postoperative emesis in children. Can J Anaesth. 1996;43:660–4.
867. Fujii Y, Toyooka H, Tanaka H. Efficacy of thoracic epidural analgesia following laparoscopic cholecystectomy. Eur J Anaesthesiol. 1998;15:342-4.
868. Fujii Y, Toyooka H, Tanaka H. Granisetron in the prevention of nausea and vomiting after middle-ear surgery: a dose-ranging study. Br J Anaesth. 1998;80:764–6.
869. Fujii Y, Toyooka H, Tanaka H. Granisetron reduces the incidence of nausea and vomiting after middle ear surgery. Br J Anaesth. 1997;79:539–40.
870. Fujii Y, Toyooka H, Tanaka H. Granisetron-droperidol combination for the prevention of postoperative nausea and vomiting in female patients undergoing breast surgery. Br J Anaesth. 1998;81:387–9.
871. Fujii Y, Toyooka H, Tanaka H. Oral granisetron prevents postoperative vomiting in children. Br J Anaesth. 1998;81:390–2.
872. Fujii Y, Toyooka H, Tanaka H. Prevention of PONV with granisetron, droperidol and metoclopramide in female patients with history of motion sickness. Can J Anesth Can d'anesthésie. 1997;44:820–4.
873. Fujii Y, Toyooka H, Tanaka H. Prevention of postoperative nausea and vomiting in female patients during menstruation: comparison of droperidol, metoclopramide and granisetron. Br J Anaesth. 1998;80:248-9
874. Fujii Y, Toyooka H, Tanaka H. Prevention of postoperative nausea and vomiting with a combination of granisetron and droperidol. Anesth Analg. 1998;86:613–6.
875. Fujii Y, Toyooka H, Tanaka H. Prophylactic antiemetic therapy with a combination of granisetron and dexamethasone in patients undergoing middle ear surgery. Br J Anaesth. 1998;81:754–6.
876. Fujii Y, Toyooka H, Tanaka H. Prophylactic anti-emetic therapy with granisetron, droperidol and metoclopramide in female patients undergoing middle ear surgery. Anaesthesia. 1998;53:1165–8.
877. Fujii Y, Uemura A, Tanaka H. Prophylaxis of nausea and vomiting after laparoscopic cholecystectomy with ramosetron: randomised controlled trial. Eur J Surg. 2002;168:583–6.
878. Fujii Y, Uemura A. Dexamethasone for the prevention of nausea and vomiting after dilatation and curettage: a randomized controlled trial. Obstet Gynecol. 2002;99:58–62.

879. Fujii Y, Uemura A. Effect of metoclopramide on pain on injection of propofol. Anaesth Intensive Care. 2004;32:653–6.
880. Garg B, Marimuthu K, Kumar V, et al. Outcome of short proximal femoral nail antirotation and dynamic hip screw for fixation of unstable trochanteric fractures. A randomised prospective comparative trial. HIP Int. 2011;21:531–6.
881. Gatto NM, Martinez LC, Spruijt-Metz D, et al. LA sprouts randomized controlled nutrition and gardening program reduces obesity and metabolic risk in Latino youth. Obesity (Silver Spring). 2015;23(6):1244-1251.
882. Gerli S, Di C. Establishing a combined stimulation protocol hFSH followed by rFSH might represent a breakthrough in the IVF practice. Eur Rev Med Pharmacol Sci. 2013;17:2091–6.
883. Gethin G, Cowman S. Manuka honey vs. hydrogel - a prospective, open label, multicentre, randomised controlled trial to compare desloughing efficacy and healing outcomes in venous ulcers. J Clin Nurs. 2009;18:466–74.
884. Gibiansky E, Struys MM, Gibiansky L, et al. AQUAVAN injection, a water-soluble prodrug of propofol, as a bolus injection: a phase I dose-escalation comparison with DIPRIVAN (part 1): pharmacokinetics. Anesthesiology. 2005;103(4):718-729.
885. Grossarth-Maticek R, Eysenck HJ. Length of survival and lymphocyte percentage in women with mammary cancer as a function of psychotherapy. Psychol Rep. 1989;65(1):315-321.
886. Guilleminault C, Quo S, Huynk N, et al. Orthodontic expansion treatment and adenotonsillectomy in the treatment of obstructive sleep apnea in prepubertal children. Sleep. 2008;31:953–7.
887. Guo J, Jin Z, Cheng Y, et al. Effect of Early Nutritional Assessment and Nutritional Support on Immune Function and Clinical Prognosis of Critically Ill Children. J Healthc Eng. 2022;2022:7100238.
888. Guo ZH, Li ZJ, Ma Y, et al. Brief cognitive-behavioural therapy for patients in the community with schizophrenia: randomised controlled trial in Beijing, China. Br J Psychiatry. 2017;210:223–9.
889. Haisch G, Boldt J, Krebs C, et al. Influence of a new hydroxyethylstarch preparation (HES 130/0.4) on coagulation in cardiac surgical patients. J Cardiothorac Vasc Anesth. 2001;15:316–21.
890. Haisch G, Boldt J, Krebs C, et al. The influence of intravascular volume therapy with a new hydroxyethyl starch preparation (6% HES 130/0.4) on coagulation in patients undergoing major abdominal surgery. Anesth Analg. 2001;92:565–71.
891. Hajizadeh Maleki B, Tartibian B, Chehrazi M. Effects of aerobic, resistance, and combined exercise on markers of male reproduction in healthy human subjects. J Strength Cond Res. 2019;33:1130–45.
892. Hak Roh Y, Kim S, Sik Gong H, et al. A randomized comparison of ultrasound-guided versus landmark-based corticosteroid injection for trigger finger. J Hand Surg Eur Vol. 2020, 45(7):NP1-NP6.
893. Hak Roh Y, Kim S, Sik Gong H, et al. Internal fixation of unstable radial head fracture: A comparison of metallic and biodegradable implants. Injury. 2019;S0020-1383(19)30460-7.
894. Hamid R, Robson M, Pearce JM. Low dose aspirin in women with raised maternal serum alpha-fetoprotein and abnormal Doppler waveform patterns from the uteroplacental circulation. Br J Obstet Gynaecol. 1994;101(6):481-484.
895. Handa F, Fujii Y. The efficacy of oral clonidine premedication in the prevention of postoperative vomiting in children following strabismus surgery. Pediatr Anesth. 2001;11:71–4.

896. Hansen PB, Penkowa M. Bismuth adjuvant ameliorates adverse effects of high-dose chemotherapy in patients with multiple myeloma and malignant lymphoma undergoing autologous stem cell transplantation: a randomised, double-blind, prospective pilot study. Support Care Cancer. 2016;25:1279–89.
897. Hao X, Zhao S, Cheng J, et al. The Clinical Effect of High-Flow Oxygen Therapy through the Nose on Patients with Acute Left Heart Failure and Hypoxemia. J Healthc Eng. 2022;2022:7117508.
898. Harik JM, Grubbs KM, Hamblen JL. The impact of treatment description format on patient preferences for posttraumatic stress disorder treatment. J Trauma Stress. 2020;33:455–64.
899. Hattori H, Saitoh Y, Nakajima H, et al. Visual evaluation of fade in response to facial nerve stimulation at the eyelid. J Clin Anesth. 2005;17(4):276-280.
900. He X. Effect of Core Strength Training on Rehabilitation of Chronic Low Back Pain in Aerobics Athletes. Comput Intell Neurosci. 2022;2022:8740665.
901. He XZ, Zhou SH, Wan XH, et al. The effect of early and intensive statin therapy on ventricular premature beat or nonsustained ventricular tachycardia in patients with acute coronary syndrome. Clin Cardiol. 2011;34:59–63.
902. Heck M, Kumle B, Boldt J, et al. Electroencephalogram bispectral index predicts hemodynamic and arousal reactions during induction of anesthesia in patients undergoing cardiac surgery. J Cardiothorac Vasc Anesth. 2000;14:693–7.
903. Heesen M, Dietrich G V, Boldt J, et al. Beta2-adrenoceptor density of human lymphocytes after nitroprusside-induced hypotension. Anesth Analg. 1995;81:1250–4.
904. Heidarzadeh Z, Samimi M, Seifati SM, et al. The effect of zinc supplementation on expressed levels of peroxisome proliferator-activated receptor gamma and glucose transporter type 1 Genes in newborns of women with gestational diabetes mellitus. Biol Trace Elem Res. 2017;175:271–7.
905. Hemilä H, Al-Biltagi M, Baset AA. Vitamin C and asthma in children: modification of the effect by age, exposure to dampness and the severity of asthma. Clin Transl Allergy. 2011;1(1):9.
906. Hengge UR, Stocks K, Faulkner S, et al. Oxymetholone for the treatment of HIV-wasting: a double-blind, randomized, placebo-controlled phase III trial in eugonadal men and women. HIV Clin Trials. 2003;4:150–63.
907. Herr HW. Randomized trial of narrow-band versus white-light cystoscopy for restaging (Second-look) transurethral resection of bladder tumors. Eur Urol. 2015;67:605–8.
908. Hiremath VP, Rao CB, Naiak V, et al. Anti-inflammatory effect of vitamin D on gingivitis: a dose response randomised controlled trial. Indian J Public Health. 2013;57:29–32.
909. Hızlı F, Argun G, Güney İ, et al. Obturator nerve block transurethral surgery for bladder cancer: comparison of inguinal and intravesical approaches: prospective randomized trial. Ir J Med Sci. 2015;185:555–60.
910. Höcker J, Raitschew B, Meybohm P, et al. Differences between bispectral index and spectral entropy during xenon anaesthesia: a comparison with propofol anaesthesia. Anaesthesia. 2010;65:595–600.
911. Höcker J, Stapelfeldt C, Leiendecker J, et al. Postoperative neurocognitive dysfunction in elderly patients after xenon versus propofol anesthesia for major noncardiac surgery. Anesthesiology. 2009;110:1068–76.
912. Homagk L, Jarmuzek T, Homagk N, et al. Advantages of clinical pathways in severity-basedtreatment of spondylodiscitis, Neurosurgical Review. 2019;43(1) .
913. Hong SY, Jun SY. Community capacity building exercise maintenance program for frail elderly women. Asian Nurs Res (Korean Soc Nurs Sci). 2017;11:166–73.
914. Hoskins W, Pollard H. The effect of a sports chiropractic manual therapy intervention on the prevention of back pain, hamstring and lower limb injuries in semi-elite Australian Rules footballers: a randomized controlled trial. BMC Musculoskelet Disord. 2010;11:64.

915. Hosseini R, Mirghotbi M, Pourvali K, et al. The effect of food service system modifications on staff body mass index in an industrial organization. J Paramed Sci. 2015;6:2008–4978.
916. Hsieh CH, Tseng CC, Shen JY, et al. Randomized controlled trial testing weight loss and abdominal obesity outcomes of moxibustion. Biomed Eng Online. 2018;17(Suppl 2):149.
917. Hu C, Zhang S, Chen Q, et al. Effects of Different Anesthetic and Analgesic Methods on Cellular Immune Function and Stress Hormone Levels in Patients Undergoing Esophageal Cancer Surgery. J Healthc Eng. 2022;2022:4752609.
918. Huang H, Xu X, Xiao Y, et al. The Influence of Different Dexmedetomidine Doses on Cognitive Function at Early Period of Patients Undergoing Laparoscopic Extensive Total Hysterectomy. J Healthc Eng. 2021;2021:3531199.
919. Huang J wen, Liu B, Hu B shan, et al. Clinical value of circulating tumor cells for the prognosis of postoperative transarterial chemoembolization therapy. Med Oncol. 2014;31(9): 175
920. Huang P, He XY, Xu M. Effect of argatroban injection on clinical efficacy in patients with acute cerebral infarction: preliminary findings. Eur Neurol. 2021;84:38–42.
921. Hughes LJ, Peiffer JJ, Scott BR. Load-velocity relationship 1RM predictions: A comparison of Smith machine and free-weight exercise. J Sports Sci. 2020;38(22):2562-2568.
922. Hui X, Yinghua Z, Shengxiong X, et al. The effectiveness of daily humanistic care in pharmaceutical care of patients with type 2 diabetes. Medicine (Baltimore). 2022;101(41):e30136.
923. Husain S, Husain S, Izhar R. Oral misoprostol alone versus oral misoprostol and Foley's catheter for induction of labor: a randomized controlled trial. J Obstet Gynaecol Res. 2017;43:1270–7.
924. Ionson E, Limbachia J, Rej S, et al. Effects of Sahaj Samadhi meditation on heart rate variability and depressive symptoms in patients with late-life depression. Br J Psychiatry. 2019;214:218–24.
925. Ishida Y, Mine T, Taguchi T. Effect of progestins with different glucocorticoid activity on bone metabolism. Clin Endocrinol (Oxf). 2008;68:423–8.
926. Ismail AM, Abbas AM, Ali MK, et al. Peri-conceptional progesterone treatment in women with unexplained recurrent miscarriage: a randomized double-blind placebo-controlled trial. J Matern Neonatal Med. 2018;31:388–94.
927. Ismail AM, Hamed AH, Saso S, et al. Randomized controlled study of pre-conception thromboprophylaxis among patients with recurrent spontaneous abortion related to antiphospholipid syndrome. Int J Gynecol Obstet. 2016;132:219–23.
928. Iwamoto J, Sato Y, Takeda T, et al. Whole body vibration exercise improves body balance and walking velocity in postmenopausal osteoporotic women treated with alendronate: Galileo and alendronate intervention trail (GAIT). J Musculoskelet Neuronal Interact. 2012;12:136–43.
929. Iwamoto J, Sato Y, Uzawa M, et al. Comparison of effects of alendronate and raloxifene on lumbar bone mineral density, bone turnover, and lipid metabolism in elderly women with osteoporosis. Yonsei Med J. 2008;49:119-28.
930. Iwamoto J, Sato Y, Uzawa M, et al. Comparison of the effects of alendronate and alfacalcidol on hip bone mineral density and bone turnover in Japanese men having osteoporosis or osteopenia with clinical risk factors for fractures. Yonsei Med J. 2009;50:474–81.
931. Iwamoto J, Takeda T, Ichimura S. Beneficial effect of etidronate on bone loss after cessation of exercise in postmenopausal osteoporotic women. Am J Phys Med Rehabil. 2002;81(6):452-457.

932. Iwamoto J, Takeda T, Sato Y, et al. Effect of whole-body vibration exercise on lumbar bone mineral density, bone turnover, and chronic back pain in post-menopausal osteoporotic women treated with alendronate. Aging Clin Exp Res. 2005;17:157–63.
933. Jain R, Grewal A. A randomized comparative study assessing efficacy of pain versus comfort scores. Saudi J Anaesth. 2017;11(4):396-401.
934. Jamal SA, Hamilton CJ, Eastell R, et al. Effect of nitroglycerin ointment on bone density and strength in postmenopausal women a randomized trial. Jama. 2011;305:800–7.
935. Jenkins DJA, Kendall CWC, Banach MS, et al. Nuts as a replacement for carbohydrates in the diabetic diet. Diabetes Care. 2011;34:1706–11.
936. Ji J, Yuan H, Wang L, et al. Is the impact of the extent of lymphadenectomy in radical prostatectomy related to the disease risk? A single center prospective study. J Surg Res. 2012;178(2):779-784.
937. Jin EZ, Yu LH, Li XQ. Loading effect of 200 mg cilostazol on platelet inhibition in patients undergoing percutaneous coronary intervention. Int Heart J. 2012;53(1):1-4.
938. Jin F, Li Z, Tan W, et al. Preoperative versus postoperative ultrasound-guided rectus sheath block for improving pain, sleep quality and cytokine levels in patients with open midline incisions undergoing transabdominal gynecological surgery: a randomized-controlled trial. BMC Anesthesiol. 2018;18:19
939. Johannigman JA, Branson RD, Edwards MG. Closed loop control of inspired oxygen concentration in trauma patients. J Am Coll Surg. 2009;208:763–8.
940. Johnson P, Pearce JM. Recurrent spontaneous abortion and polycystic ovarian disease: comparison of two regimens to induce ovulation. BMJ. 1990;300(6718):154-156.
941. Johnston SRD, Hegg R, Im SA, et al. Phase III, Randomized Study of Dual Human Epidermal Growth Factor Receptor 2 (HER2) Blockade With Lapatinib Plus Trastuzumab in Combination With an Aromatase Inhibitor in Postmenopausal Women With HER2-Positive, Hormone Receptor-Positive Metastatic Breast Cancer: ALTERNATIVE. J Clin Oncol. 2018 Mar 10;36(8):741-748.
942. Kandeel A, Elmorhedi M, Abdalla U. Digital assistance of nasogastric tube insertion in intubated patients under general anesthesia: a single-blinded prospective randomized study. Saudi J Anaesth. 2017;11:283–6.
943. Karakisi SO, Kunt AG, Çankaya İ, et al. Do phosphorylcholine-coated and uncoated oxygenators differ in terms of elicitation of cellular immune response during cardiopulmonary bypass surgery? Perfusion. 2015;32:NP2–9.
944. Karim R, Hameed R, Ali K, et al. Comparison of oral versus intravenous proton pump inhibitors in preventing re-bleeding from peptic ulcer after successful endoscopic therapy. Cureus. 2020: 12(1):e6741.
945. Karp DD, Paz-Ares LG, Novello S, et al. Phase II study of the anti-insulin-like growth factor type 1 receptor antibody CP-751,871 in combination with paclitaxel and carboplatin in previously untreated, locally advanced, or metastatic non-small-cell lung cancer. J Clin Oncol. 2009;27:2516–22.
946. Katayama T, Saitoh Y, Nemoto C, et al. Effects of olprinone on neuromuscular blockade caused by vecuronium. Fukushima J Med Sci. 2007;53:61–9.
947. Kaur A, Garg R, Mittal RK, et al. Comparative Efficacy of Intralesional Triamcinolone Acetonide and 5-Fluorouracil for Keloid Scars. Plast Aesthet Nurs (Phila). 2022;42(4):184-189.
948. Kessler RC, Duncan GJ, Gennetian LA, et al. Associations of housing mobility interventions for children in high-poverty neighborhoods with subsequent mental disorders during adolescence. JAMA. 2014;311(9):937-948.
949. Khani Jeihooni A, Hidarnia A, Kaveh MH, et al. The effect of an educational program based on health belief model and social cognitive theory in prevention of osteoporosis in women. J Health Psychol. 2017;22:NP1–11.

950. Khattab AM, El-Seify ZA, Shaaban A, et al. Sevoflurane-emergence agitation: effect of supplementary low-dose oral ketamine premedication in preschool children undergoing dental surgery. Eur J Anaesthesiol. 2010;27(4):353-358.
951. Khouri R. Impact of an educational program on nursing students' caring and self-perception in intensive clinical training in Jordan. Adv Med Educ Pract. 2011;2:173–85.
952. Kimura S, Sawada T, Shiraishi J, et al. Effects of valsartan on cardiovascular morbidity and mortality in high-risk hypertensive patients with new-onset diabetes mellitus. Circ J. 2012;DN/JST.JSTAGE/circj/CJ-12-0387.
953. Kiran M, Makridis KG, Armstrong C, et al. A Randomized Controlled Trial Comparing Modular and Nonmodular Neck Versions of a Titanium Stem. J Arthroplasty. 2018:S0883-5403(18)30820-9.
954. Kocak I, Ustün C. Effects of metformin on insulin resistance, androgen concentration, ovulation and pregnancy rates in women with polycystic ovary syndrome following laparoscopic ovarian drilling. J Obstet Gynaecol Res. 2006;32(3):292-298.
955. Kouchaki E, Afarini M, Abolhassani J, et al. High-dose ω-3 fatty acid plus vitamin D3 supplementation affects clinical symptoms and metabolic status of patients with multiple sclerosis: a randomized controlled clinical trial. J Nutr. 2018;148:1380–6.
956. Kudoh O, Satoh D, Hori N, et al. The effects of a recruitment manoeuvre with positive end-expiratory pressure on lung compliance in patients undergoing robot-assisted laparoscopic radical prostatectomy. J Clin Monit Comput. 2020;34(2):303-310.
957. Kumar A, Begum N, Prasad S, et al. Oral dydrogesterone treatment during early pregnancy to prevent recurrent pregnancy loss and its role in modulation of cytokine production: a double-blind, randomized, parallel, placebo-controlled trial. Fertil Steril. 2014;102:1357-63.
958. Kumle B, Boldt J, Piper S, et al. The influence of different intravascular volume replacement regimens on renal function in the elderly. Anesth Analg. 1999;89:1124–30.
959. Kumral E, Sirin H, Sağduyu A, et al. Decompressive surgery in patients with malignant middle cerebral artery infarction: a randomized, controlled trial in a Turkish population (Demitur trial). Int J Stroke. 2021;17474930211007671
960. La Sala GB, Nicoli A, Fornaciari E, et al. Intracytoplasmic morphologically selected sperm injection versus conventional intracytoplasmic sperm injection: a randomized controlled trial. Reprod Biol Endocrinol. 2015;13:97.
961. Lai Z, Shi S, Fei J, et al. Total knee arthroplasty performed with either a mini-subvastus or a standard approach: a prospective randomized controlled study with a minimum follow-up of 2 years. Arch Orthop Trauma Surg. 2014;134:1155–62.
962. Lakkireddy M, Gadiga SG, Malathi RD, et al. Impact of daily high dose oral vitamin D therapy on the inflammatory markers in patients with COVID 19 disease. Sci Rep. 2021;11:10641.
963. Lang K, Boldt J, Suttner S, et al. Colloids versus crystalloids and tissue oxygen tension in patients undergoing major abdominal surgery. Anesth Analg. 2001;93:405–9.
964. Lang K, Suttner S, Boldt J, et al. Volume replacement with HES 130/0.4 may reduce the inflammatory response in patients undergoing major abdominal surgery. Can J Anesth. 2003;50:1009–16.
965. Lee DB, Suh HS, Choi YS. A comparative study of low-fluence 1,064nm Q-Switched Nd:YAG laser with or without chemical peeling using Jessner's solution in melasma patients. J Cosmet Laser Ther. 2014;16:264–70.
966. Lee HJ, Kim KS, Jeong JS, et al. Comparison of four facial muscles, orbicularis oculi, corrugator supercilii, masseter or mylohyoid, as best predictor of good conditions for intubation: a randomised blinded trial. Eur J Anaesthesiol. 2013;30:556–62.

967. Lee I. The effect of postural control intervention for congenital muscular torticollis: a randomized controlled trial. Clin Rehabil. 2015;29:795–802.
968. Lehmann A, Boldt J, Römpert R, et al. Target-controlled infusion or manually controlled infusion of propofol in high-risk patients with severely reduced left ventricular function. J Cardiothorac Vasc Anesth. 2001;15:445–50.
969. Lehmann A, Boldt J, Thaler E, et al. Bispectral index in patients with target-controlled or manually-controlled infusion of propofol. Anesth Analg. 2002;95:639–44.
970. Lehmann A, Karzau J, Boldt J, et al. Bispectral index-guided anesthesia in patients undergoing aortocoronary bypass grafting. Anesth Analg. 2003;96:336–43.
971. Lehmann A, Zeitler C, Thaler E, et al. Comparison of two different anesthesia regimens in patients undergoing aortocoronary bypass grafting surgery: sufentanil-midazolam versus remifentanil-propofol. J Cardiothorac Vasc Anesth. 2000;14:416–20.
972. Lewis JE, Atlas SE, Abbas MH, et al. The novel effects of a hydrolyzed polysaccharide dietary supplement on immune, hepatic, and renal function in adults with HIV in a randomized, double-blind placebo-control trial. J Diet Suppl. 2021; 18:I-XIII
973. Li D. The Research on the Effect of the Food with Different Glycaemic Index and Glycaemic Load on the Immunity of Endurance Athletes. Open Biomed Eng J. 2015;9:305-309.
974. Li G, Li S, Sun L, et al. A comparison study of immune-inflammatory response in electroacupuncture and transcutaneous electrical nerve stimulation for patients undergoing supratentorial craniotomy. Int J Clin Exp Med. 2015;8(2):2662-7.
975. Li G, Lv C, Tian L, et al. A randomized controlled trial of botulinum toxin A for treating neuropathic pain in patients with spinal cord injury. Medicine (Baltimore). 2017;96(20):e6919.
976. Li H. Effect of EPOC on Serum MicroRNA Expression in Patients with Hypertension. J Healthc Eng. 2022;2022:1998445.
977. Li J, Nie Q, Chen H. Clinical Effect of acupuncture combined with traditional Chinese medicine application on the treatment of functional dyspepsia in children and the influence on serum 5-HT and NO Levels. Comput Intell Neurosci. 2022;2022:6800662.
978. Li JK, Wang J, Li TF. Interposed abdominal compression-cardiopulmonary resuscitation after cardiac surgery. Interact Cardiovasc Thorac Surg. 2014;19(6):985-989.
979. Li L, Li Y, Huang R, et al. The value of adding transcutaneous neuromuscular electrical stimulation (VitalStim) to traditional therapy for post-stroke dysphagia: a randomized controlled trial. Eur J Phys Rehabil Med. 2015;51(1):71-78.
980. Li S, Chen P, Yang Q. Denosumab versus zoledronic acid in cases of surgically unsalvageable giant cell tumor of bone: a randomized clinical trial. J Bone Oncol. 2019;15:100217.
981. Li W, Wang S, Zhang Y. The effectiveness of the invisalign appliance in extraction cases using the the ABO model grading system: a multicenter randomized controlled trial. Int J Clin Exp Med. 2015;8:8276-82.
982. Li X-G, Tang T, Qian Z-L, et al. Comparison of the mini-midvastus with the mini-medial parapatellar approach in primary TKA. Orthopedics. 2010;33(10):723.
983. Liang H, Huang J, Tong J, et al. Application of Rapid Rehabilitation Nursing in Thoracic Surgery Nursing. J Healthc Eng. 2021;2021:6351170.
984. Liao C-J, Song S-H, Li T, et al. Drug coated balloon vs. standard percutaneous transluminal angioplasty for the treatment of femoropopliteal artery disease: 12 -month results of the LEGFLOW OTW China randomised controlled trial. Eur J Vasc Endovasc Surg. 2019;S1078-5884(18)30793-7
985. Liedl A, Müller J, Morina N, et al. Physical activity within a CBT intervention improves coping with pain in traumatized refugees: results of a randomized controlled design. Pain Med. 2011;12:234–45.

986. Lin F, Yao L, Bhikoo C, et al. Impact of fixed orthodontic appliance or clear-aligner on daily performance, in adult patients with moderate need for treatment. Patient Prefer Adherence. 2016;10:1639–45.
987. Liu H, Liu Z, Liu Y, et al. Effect of atorvastatin on resolution of chronic subdural hematoma: a prospective observational study. J Neurosurg. 2017;126(2):651.
988. Liu H, Luo Z, Liu Z, et al. Atorvastatin may attenuate recurrence of chronic subdural hematoma. Front Neurosci. 2016;10:303.
989. Liu JN, Wang MY, Wan K, et al. A randomized controlled trial comparing between titanium osteosynthesis and biodegradable osteosynthesis in access mandibulotomy for oral malignancy. J Oral Maxillofac Surg. 2018.
990. Liu P, You J, Loo WTY, et al. The efficacy of Guolin-Qigong on the body-mind health of Chinese women with breast cancer: a randomized controlled trial. Qual Life Res. 2017;26(9):2321-2331.
991. Liu X, Wei C, Wang Z, et al. Different anesthesia methods for laparoscopic cholecystectomy. Anaesthesist. 2011;60:723–8.
992. Liu XH, Fu PL, Wang SY, et al. The effect of drainage tube on bleeding and prognosis after total knee arthroplasty: a prospective cohort study. J Orthop Surg Res. 2014;9:27.
993. Lu D, Wang Y, Zhao T, et al. Successful implementation of an enhanced recovery after surgery (ERAS) protocol reduces nausea and vomiting after infratentorial craniotomy for tumour resection: a randomized controlled trial. BMC Neurol. 2020;20:150.
994. Lucarelli R, Picchio M, Caporossi M, et al. Transanal haemorrhoidal dearterialisation with mucopexy versus stapler haemorrhoidopexy: a randomised trial with long-term follow-up. Ann R Coll Surg Engl. 2013;95:246–51.
995. Ludbrook GL, Li F, Sleigh J, et al. Assessments of onset and duration of drug effects and pharmacokinetics by dose level of HSK3486, a new sedative-hypnotic agent, in healthy female/male subjects: a phase I multiarm randomized controlled clinical trial. Anesth Analg. 2021.
996. Luo D, Wu G, Ji Y, et al. The comparative study of clinical efficacy and safety of baclofen vs tolperisone in spasticity caused by spinal cord injury. Saudi Pharm J. 2017;25:655–9.
997. Luo Y, Li Z, Cui S, et al. Joint detection of ERCC1, TUBB3, and TYMS guidance selection of docetaxel, 5-fluorouracil and cisplatin (DDP) individual chemotherapy in advanced gastric cancer patients. Eur J Med Res. 2014;19:50
998. Luthra P, Rao JKD, Arya V, et al. 6-0 nylon versus 6-0 vicryl rapide in chieloplasty. Ann Maxillofac Surg. 2016;6:272-277.
999. Lv SJ, Zhang GH, Xia JM, et al. Early use of high-dose vitamin C is beneficial in treatment of sepsis. Ir J Med Sci. 2021;190(3):1183-1188.
1000. Ma Z, Wang Y, Sun Y, et al. Efficacy of Crizotinib Combined with Chemotherapy in Treating Advanced Non-Small-Cell Lung Cancer and Effect on Patients' Quality of Life and Adverse Reaction Rate. J Healthc Eng. 2022;2022:7898737.
1001. Macías-Cortés E del C, Llanes-González L, Aguilar-Faisal L, et al. Individualized homeopathic treatment and fluoxetine for moderate to severe depression in peri- and postmenopausal women (HOMDEP-MENOP Study): a randomized, double-dummy, double-blind, placebo-controlled trial. PLoS One. 2015;10:e0118440.
1002. Madbouly KM, Hussein A, Omar W, et al. Regenerated oxidized cellulose reinforcement of low rectal anastomosis: do we still need diversion? Dis Colon Rectum. 2010;53:889–95.
1003. Magalhães MC, Soares CJ, Araújo E, et al. The effect of adenotonsillectomy and rapid maxillary expansion on the upper airway in pediatric obstructive sleep apnea: a randomized crossover-controlled trial. Sleep. 2021:zsab304.
1004. Maged AM, Youssef G, Eldaly A, et al. Benefits of vaginal misoprostol prior to IUD insertion in women with previous caesarean delivery: a randomised controlled trial. Eur J Contracept Reprod Heal Care. 2018;23:32–7.

1005. Maher M, Sayyed T, Elkhadry SW. Nifedipine alone or combined with sildenafil citrate for management of threatened preterm labour: a randomised trial. Bjog An Int J Obstet Gynaecol. 2019;126:729–35.
1006. Maher MA, Sayyed TM, Elkhouly N. Cervical mucus removal prior to intrauterine insemination: a randomized trial. BJOG An Int J Obstet Gynaecol. 2018;125:841–7.
1007. Maher MA, Sayyed TM, Elkhouly N. Sildenafil citrate therapy for oligohydramnios: a randomized controlled trial. Obstet Gynecol. 2017;129:615–20.
1008. Mahran A, Ibrahim M, Bahaa H. The effect of endometrial injury on first cycle IVF/ICSI outcome: a randomized controlled trial. Int J Reprod Biomed. 2016;14:193–8.
1009. Maktabi M, Jamilian M, Asemi Z. Magnesium-zinc-calcium-vitamin D co-supplementation improves hormonal profiles, biomarkers of inflammation and oxidative stress in women with polycystic ovary syndrome: a randomized, double-blind, placebo-controlled Trial. Biol Trace Elem Res. 2018;182:21–8.
1010. Malik S, Asprusten TT, Pedersen M, et al. Cognitive-behavioural therapy combined with music therapy for chronic fatigue following Epstein-Barr virus infection in adolescents: a feasibility study. BMJ Paediatr Open. 2020;4(1):e000620.
1011. Manuar MB, Majumdar S, Das A, et al. Pain relief after Arthroscopic Knee Surgery: a comparison of intra-articular ropivacaine, fentanyl, and dexmedetomidine: a prospective, double-blinded, randomized controlled study. Saudi J Anaesth. 2014;8:233–7.
1012. Maroli S, Srinath HP, Goinka C, et al. Sniffing out pain: an in vivo intranasal study of analgesic efficacy. J Int Oral Heal JIOH. 2014;6:66–71.
1013. Marzouk T, Barakat R, Ragab A, et al. Lavender-thymol as a new topical aromatherapy preparation for episiotomy: a randomised clinical trial. J Obstet Gynaecol (Lahore). 2015;35:472–5.
1014. Matsuoka K, Sakamoto N, Akanuma Y, et al. A long-term effect of epalrestat on motor conduction velocity of diabetic patients: ARI-Diabetes Complications Trial (ADCT). Diabetes Res Clin Pract. 2007;77 Suppl 1:S263-S268.
1015. Matsuyama W, Mitsuyama H, Watanabe M, et al. Effects of omega-3 polyunsaturated fatty acids on inflammatory markers in COPD. Chest. 2005;128:3817–27.
1016. Mayer J, Boldt J, Röhm KD, et al. Desflurane anesthesia after sevoflurane inhaled induction reduces severity of emergence agitation in children undergoing minor ear-nose-throat surgery compared with sevoflurane induction and maintenance. Anesth Analg. 2006;102:400–4.
1017. Mayer J, Boldt J, Schellhaa A, et al. Bispectral index-guided general anesthesia in combination with thoracic epidural analgesia reduces recovery time in fast-track colon surgery. Anesth Analg. 2007;104:1145–9.
1018. Mayer J, Boldt J, Triem JG, et al. Individual titration of propofol plasma target improves anaesthetic stability in patients undergoing major abdominal surgery: a comparison with manually controlled infusion. Eur J Anaesthesiol. 2008;25:741–7.
1019. McCoy J, Goren A, Cadegiani FA, et al. Proxalutamide reduces the rate of hospitalization for COVID-19 male outpatients: a randomized double-blinded placebo-controlled trial. Front Med. 2021;8:668698.
1020. Meftah M, Wong AC, Nawabi DH, et al. Pain management after total knee arthroplasty using a multimodal approach. Orthopedics. 2012;35: e660-4.
1021. Melis MH, Elagwany AMS. Adjuvant chemotherapy followed by interval debulking surgery versus upfront surgery followed by chemotherapy in advanced epithelial ovarian carcinoma. Hematol Oncol Stem Cell Ther. 2016;S1658-3876(16)30004-8.
1022. Memis D, Inal MT, Temizoz O, et al. The effect of celiac plexus block in critically Ill patients intolerant of enteral nutrition: a randomized, placebo-controlled study. Anesth Analg. 2010;110:1071–5.

1023. Menges T, Welters I, Wagner RM, et al. The influence of acute preoperative plasmapheresis on coagulation tests, fibrinolysis, blood loss and transfusion requirements in cardiac surgery. Eur J Cardio-thoracic Surg. 1997;11:557–63.
1024. Mengistu A, Röhm KD, Boldt J, et al. The influence of aprotinin and tranexamic acid on platelet function and postoperative blood loss in cardiac surgery. Anesth Analg. 2008;107:391–7.
1025. Mengistu AM, Wolf MW, Boldt J, et al. Influence of controlled hypotension using esmolol and sodium nitroprusside on natriuretic peptides in patients undergoing endonasal sinus surgery. Eur J Anaesthesiol. 2007;24:529–34.
1026. Messer LH, Buckingham BA, Cogen FR, et al. Positive impact of the bionic pancreas on diabetes control in youth 6-17 years old with type 1 diabetes: a multicenter randomized trial. Diabetes Technol Ther. 2022;24:712–25.
1027. Michail S. Fecal microbial transplant in children with ulcerative colitis. J Pediatr Gastroenterol Nutr. 2018;1.
1028. Mira TAA, Giraldo PC, Yela DA, et al. Effectiveness of complementary pain treatment for women with deep endometriosis through transcutaneous electrical nerve stimulation (TENS): randomized controlled trial. Eur J Obstet Gynecol Reprod Biol. 2015;194:1–6.
1029. Mitra T, Das A, Majumdar S, et al. Prevention of altered hemodynamics after spinal anesthesia: a comparison of volume preloading with tetrastarch, succinylated gelatin and ringer lactate solution for the patients undergoing lower segment caesarean section. Saudi J Anaesth. 2014;8:456–62.
1030. Mochizuki S, Dahlöf B, Shimizu M, et al. Valsartan in a Japanese population with hypertension and other cardiovascular disease (Jikei Heart Study): a randomised, open-label, blinded endpoint morbidity-mortality study. Lancet. 2007;369:1431–9.
1031. Mohammadi Pelarti A, Eidani E, Hatefnia E, et al. The effects of family-centered education based on the health belief model on knowledge and attitude among the parents of children with asthma: a randomized controlled clinical trial. Jundishapur J Chronic Dis Care. 2019;8: e95909.
1032. Mohanty S, Gianni C, Mohanty P, et al. Impact of rotor ablation in nonparoxysmal atrial fibrillation patients: results from the randomized OASIS trial. J Am Coll Cardiol. 2016;68:274–82.
1033. Moll D. Telescopic crown-retained removable partial dentures on teeth and implants: an 8- to 9-year prospective randomized clinical trial. Clin Oral Implants Res. 2012;23:895–895.
1034. Mondal B, Choudhury S, Banerjee R, et al. Non-invasive vagus nerve stimulation improves clinical and molecular biomarkers of Parkinson's disease in patients with freezing of gait. NPJ Park Dis. 2021;7(1):46.
1035. Monticone M, Ambrosini E, Portoghese I, et al. Multidisciplinary program based on early management of psychological factors reduces disability of patients with subacute low back pain: one-year results of a randomized controlled study. Eur J Phys Rehabil Med. 2021;57:959–67.
1036. Monticone M, Ambrosini E, Rocca B, et al. Group-based multimodal exercises integrated with cognitive-behavioural therapy improve disability, pain and quality of life of subjects with chronic neck pain: a randomized controlled trial with one-year follow-up. Clin Rehabil. 2017;31:742–52.
1037. Monticone M, Ambrosini E, Rocca B, et al. Multimodal exercises integrated with cognitive-behavioural therapy improve disability of patients with failed back surgery syndrome: a randomized controlled trial with one-year follow-up. Disabil Rehabil. 2022;44:3422–9.
1038. Motiani P, Chaudhary S, Bahl N, et al. Intrathecal sufentanil versus fentanyl for lower limb surgeries - a randomized controlled trial. J Anaesthesiol Clin Pharmacol. 2011;27:67-73.
1039. Mshimesh B. Efficacy and safety of adalimumab versus infliximab in patients suffered from moderate to severe active ulcerative colitis. Asian J Pharm Clin Res 2017;10:300-7.

1040. Mukherjee A, Das A, Mayur N, et al. Comparative evaluation of analgesic sparing efficacy between dexmedetomidine and clonidine used as adjuvant to ropivacaine in thoracic paravertebral block for patients undergoing breast cancer surgery: a prospective, randomized, double-blind study. Saudi J Anaesth. 2018;12:548–54.

1041. Mukhtar S, Ishag Adam M, Martinez-Jimenez E, et al. Transversus abdominis plane block versus local anesthetic wound infiltration for postoperative analgesia in adult patients undergoing hernia repair in daycare procedure: a randomized control trial. Cureus. 2022;14: e21311

1042. Munyangi J, Cornet-Vernet L, Idumbo M, et al. Artemisia annua and Artemisia afra tea infusions vs. artesunate-amodiaquine (ASAQ) in treating Plasmodium falciparum malaria in a large scale, double blind, randomized clinical trial. Phytomedicine. 2019;57:49–56.

1043. Munyangi J, Cornet-Vernet L, Idumbo M, et al. Effect of Artemisia annua and Artemisia afra tea infusions on schistosomiasis in a large clinical trial. Phytomedicine. 2018;51:233–40.

1044. Muramatsu T, Matsushita K, Yamashita K, et al. Comparison between valsartan and amlodipine regarding cardiovascular morbidity and mortality in hypertensive patients with glucose intolerance: NAGOYA HEART study. Hypertension. 2012;59:580–6.

1045. Murdoch BE, Ng ML, Barwood CHS. Treatment of articulatory dysfunction in Parkinson's disease using repetitive transcranial magnetic stimulation. Eur J Neurol. 2012;19:340–7.

1046. Murthy Tk K, Kumar Pv V. Effect of perioperative intravenous lignocaine infusion on haemodynamic responses and postoperative analgesia in laparoscopic cholecystectomy surgeries. Anesthesiol Pain Med. 2018;8:e63490.

1047. Nabhan AFI. A randomized clinical trial of the effects of isosorbide mononitrate on bone formation and resorption in post-menopausal women: a pilot study. Hum Reprod. 2006;21:1320–4.

1048. Nada AM, Shafeek MM, El Maraghy MA, et al. Antenatal corticosteroid administration before elective caesarean section at term to prevent neonatal respiratory morbidity: a randomized controlled trial. Eur J Obstet Gynecol Reprod Biol. 2016;199:88–91.

1049. Nakao N, Seno H, Kasuga H, et al. Effects of combination treatment with losartan and trandolapril on office and ambulatory blood pressures in non-diabetic renal disease: a COOPERATE-ABP Substudy. Am J Nephrol. 2004;24:543–8.

1050. Nakao N, Yoshimura A, Morita H, et al. Combination treatment of angiotensin-II receptor blocker and angiotensin-converting-enzyme inhibitor in non-diabetic renal disease (COOPERATE): a randomised controlled trial. Lancet. 2003;361:117–24.

1051. Nandyala S V, Marquez-Lara A, Fineberg SJ, et al. Prospective, randomized, controlled trial of silicate-substituted calcium phosphate versus rhBMP-2 in a minimally invasive transforaminal lumbar interbody fusion. Spine (Phila Pa 1976). 2014;39:185–91.

1052. Narumi H, Takano H, Shindo S, et al. Effects of valsartan and amlodipine on cardiorenal protection in Japanese hypertensive patients: the valsartan amlodipine randomized trial. Hypertens Res. 2011;34:62–9.

1053. Neligan PJ, Malhotra G, Fraser M, et al. Noninvasive ventilation immediately after extubation improves lung function in morbidly obese patients with obstructive sleep apnea undergoing laparoscopic bariatric surgery. Anesth Analg. 2010;110:1360–5.

1054. Ni G-X, Song L, Yu B, et al. Tai chi improves physical function in older Chinese women with knee osteoarthritis. JCR J Clin Rheumatol. 2010;16:64–7.

1055. Niederhofer H, Staffen W, Mair A, et al. Brief report: melatonin facilitates sleep in individuals with mental retardation and insomnia. J Autism Dev Disord. 2003;33(4):469-472.

1056. Niederhofer H, Staffen W, Mair A. A placebo-controlled study of lofexidine in the treatment of children with tic disorders and attention deficit hyperactivity disorder. J Psychopharmacol. 2003;17:113–9.

1057. Niederhofer H, Staffen W. Acamprosate and its efficacy in treating alcohol dependent adolescents. 2003, 12(3):144-8.
1058. Nischal K, Mishra SK, Chowdhary R. Crestal bone changes and patient satisfaction with single implant-retained mandibular overdentures with dalla bona and locator attachments with immediate loading protocols. A randomized controlled clinical study. J Prosthodont. 2020;29:756–65.
1059. Niu L, Chen L, Luo Y, et al. Oxycodone versus morphine for analgesia after laparoscopic endometriosis resection. BMC Anesthesiol. 2021;21:194
1060. Numazaki M, Fujii Y. Reduction of emetic symptoms during cesarean delivery with antiemetics: propofol at subhypnotic dose versus traditional antiemetics. J Clin Anesth. 2003;15:423–7.
1061. Numazaki M, Fujii Y. Reduction of postoperative emetic episodes and analgesic requirements with dexamethasone in patients scheduled for dental surgery. J Clin Anesth. 2005;17:182–6.
1062. Nyberg J, Li H, Wessmark P, et al. Population kinetics of 0.9% saline distribution in hemorrhaged awake and isoflurane-anesthetized volunteers. Anesthesiology. 2019;131:501–11.
1063. Obaid H, Clarke A, Rosenfeld P, et al. Skin-derived fibroblasts for the treatment of refractory Achilles tendinosis: preliminary short-term results. J Bone Jt Surg. 2012;94:193–200.
1064. Ochmann C, Tuschy B, Beschmann R, et al. Supplemental oxygen reduces serotonin levels in plasma and platelets during colorectal surgery and reduces postoperative nausea and vomiting. Eur J Anaesthesiol. 2010;27:1036–43.
1065. Ogba FN, Ede MO, Onyishi CN, et al. Effectiveness of music therapy with relaxation technique on stress management as measured by perceived stress scale. Medicine (Baltimore). 2019;98(15):e15107.
1066. Ohashi J, Katsura T. The effects of coaching on salivary cortisol stress marker in mothers with young children, a randomized controlled trial. J Rural Med. 2015;10:20–8.
1067. Okhovatian F, Naimi SS, Mehdikhani R. Comparison between the Immediate Effect of Manual Pressure Release and Strain/counterstrain Techniques on Latent Trigger Point of Upper Trapezius Muscle. Clinical Chiropractic. 2012;15(2):51-55.
1068. Ong KS, Tan JM. Preoperative intravenous tramadol versus ketorolac for preventing postoperative pain after third molar surgery. Int J Oral Maxillofac Surg. 2004;33(3):274-278.
1069. Oostdijk EAN, Kesecioglu J, Schultz MJ, et al. Effects of decontamination of the oropharynx and intestinal tract on antibiotic resistance in ICUs: a randomized clinical trial. JAMA. 2014;312:1429–37.
1070. Oshaghi S, Ghadimi K, Rezaeian A, et al. Effect of short term use of repetitive transcranial stimulation as an adjuvant therapy for Bell's Palsy. Arch Neurosci. 2019;6(1):e81557.
1071. Pak CS, Lee J, Lee H, et al. A phase III, randomized, double-blind, matched-pairs, active-controlled clinical trial and preclinical animal study to compare the durability, efficacy and safety between polynucleotide filler and hyaluronic acid filler in the correction of crow's feet: a new concept of regenerative filler. J Korean Med Sci. 2014;29:S201–9.
1072. Parfenov VA, Kamchatnov PR, Khasanova DR, et al. The randomized clinical trial results of the anxiety treatment in patients with somatoform dysfunction and neurotic disorders. Sci Rep. 2021;11:24282.
1073. Park CH, Lee HK, Kim MK, et al. Comparison of 0.05% cyclosporine and 3% diquafosol solution for dry eye patients: a randomized, blinded, multicenter clinical trial. BMC Ophthalmol. 2019;19(1):131.

1074. Park J, Shin JM, Lee DK, et al. The effect of synthetic osteoconductive bone graft material for augmentation of internally fixed unstable trochanteric fractures. Biomed Res Int. 2019;2019: 5014928

1075. Paul S, Choudhury R, Kumari N, et al. Is treatment with platelet-rich fibrin better than zinc oxide eugenol in cases of established dry socket for controlling pain, reducing inflammation, and improving wound healing?. J Korean Assoc Oral Maxillofac Surg. 2019;45(2):76-82.

1076. Pazarcikci F, Efe E. Effect of care programme based on Comfort Theory on reducing parental anxiety in the paediatric day surgery: randomised controlled trial. J Clin Nurs. 2022; 31(7-8): 922-934.

1077. Pearce JM, Hamid RI. Randomised controlled trial of the use of human chorionic gonadotrophin in recurrent miscarriage associated with polycystic ovaries. BJOG An Int J Obstet Gynaecol. 1994;101:685–8.

1078. Perdanakusuma DS, Hariani L, Nasser NF, et al. The effect of a single-strain probiotic administration in the treatment of thermal burns patients. Iran J Microbiol. 2019;11(6):541.

1079. Pérez-Legaz J, Arroyo A, Moya P, et al. Perianal versus endoanal application of glyceryl trinitrate 0.4% ointment in the treatment of chronic anal fissure: results of a randomized controlled trial. Is this the solution to the headaches? Dis Colon Rectum. 2012;55:893–9.

1080. Picaud JC, Lapillonne A, Rigo J, et al. Nitrogen utilization and bone mineralization in very low birth weight infants fed partially hydrolyzed preterm formula. Semin Perinatol. 2002;26(6):439-446.

1081. Pietrzkowski Z, Roldán Mercado-Sesma A, Argumedo R, et al. Effects of once-daily versus twice daily dosing of calcium fructoborate on knee discomfort. a 90 Day, double-blind, placebo controlled randomized clinical study. J Aging Res Lifestyle. 2018; 7: 31-36

1082. Pins JJ, Keenan JM. Effects of whey peptides on cardiovascular disease risk factors. J Clin Hypertens (Greenwich). 2006;8:775–82.

1083. Piper SN, Boldt J, Schmidt C, et al. Hemodynamics, intra-mucosal pH and regulators of circulation during perioperative epidural analgesia. Can J Anesth Can d'anesthésie. 2000;47:631–7.

1084. Piper SN, Boldt J, Schmidt CC, et al. Influence of dopexamine on hemodynamics, intramucosal pH, and regulators of the macrocirculation and microcirculation in patients undergoing abdominal aortic surgery. J Cardiothorac Vasc Anesth. 2000;14:281–7.

1085. Piper SN, Fent MT, Röhm KD, et al. Urapidil does not prevent postanesthetic shivering: a dose-ranging study. Can J Anesth. 2001;48:742–7.

1086. Piper SN, Kumle B, Maleck WH, et al. Diltiazem may preserve renal tubular integrity after cardiac surgery. Can J Anesth Can D'anesthésie. 2003;50:285–92.

1087. Piper SN, Kumle B, Maleck WH, et al. Effects of postoperative sedation with propofol and midazolam on pancreatic function assessed by pancreatitis-associated protein. Anaesthesia. 2001;56:836–40.

1088. Piper SN, Maleck WH, Boldt J, et al. A comparison of urapidil, clonidine, meperidine and placebo in preventing postanesthetic shivering. Anesth Analg. 2000;90:954–7.

1089. Piper SN, Röhm KD, Boldt J, et al. Hepatocellular integrity in patients requiring parenteral nutrition: comparison of structured MCT/LCT vs. a standard MCT/LCT emulsion and a LCT emulsion. Eur J Anaesthesiol. 2008;25:557–65.

1090. Piper SN, Röhm KD, Maleck WH, et al. Dolasetron for preventing postanesthetic shivering. Anesth Analg. 2002;94:106–11.

1091. Piper SN, Röhm KD, Suttner SW, et al. A comparison of nefopam and clonidine for the prevention of postanaesthetic shivering: a comparative, double-blind and placebo-controlled dose-ranging study. Anaesthesia. 2004;59:559–64.

1092. Piper SN, Suttner S, Maleck WH, et al. Effects of sodium nitroprusside-induced controlled hypotension on pancreatic function assessed by pancreatitis-associated protein in patients undergoing radical prostatectomy. Eur J Anaesthesiol. 2002;19:609-13.
1093. Piper SN, Suttner S, Röhm KD, et al. Dolasetron, but not metoclopramide prevents nausea and vomiting in patients undergoing laparoscopic cholecystectomy. Can J Anesth Can d'anesthésie. 2002;49:1021–8.
1094. Piper SN, Suttner SW, Schmidt CC, et al. Acute phase response to nitroprusside-induced controlled hypotension in patients undergoing radical prostatectomy. Anaesthesia. 2000;55:131–6.
1095. Piper SN, Suttner SW, Schmidt CC, et al. Nefopam and clonidine in the prevention of postanaesthetic shivering. Anaesthesia. 1999;54:695–9.
1096. Piper SN, Triem JG, Maleck WH, et al. Placebo-controlled comparison of dolasetron and metoclopramide in preventing postoperative nausea and vomiting in patients undergoing hysterectomy. Eur J Anaesthesiol. 2001;18:251–6.
1097. Polizzi di Sorrentino E, Herrmann B, Villeval MC. Dishonesty is more affected by BMI status than by short-term changes in glucose. Sci Rep. 2020;10(1):12170.
1098. Poppe T, Thompson B, Boardman JP, et al. Effect of antenatal magnesium sulphate on MRI biomarkers of white matter development at term equivalent age: The magnum study. EBioMedicine. 2020;59:102957.
1099. Pott-Junior H, Bastos Paoliello MM, Miguel A de QC, et al. Use of ivermectin in the treatment of Covid-19: a pilot trial. Toxicol Reports. 2021;8:505–10.
1100. Prezioso D, Iacono F, Di Lauro G, et al. Stress urinary incontinence: long-term results of laparoscopic Burch colposuspension. BMC Surg. 2013;13(Suppl 2):S38.
1101. Qi L, Guo X, Nie C, et al. Research on Effects of Oropharyngeal Aspiration on Incidence of Ventilator-Associated Pneumonia in Patients with Cerebral Hemorrhage in ICU. J Healthc Eng. 2022;2022:6433666.
1102. Qi T, Ye L, Wang B, et al. Comparison of the effects of extracorporeal shock wave therapy and a vacuum erectile device on penile erectile dysfunction: a randomized clinical trial. Medicine (Baltimore). 2017;96:e8414.
1103. Qiu Y, Wu J, Huang Q, et al. Acute pain after serratus anterior plane or thoracic paravertebral blocks for video-assisted thoracoscopic surgery. Eur J Anaesthesiol. 2020.
1104. Rachmani R, Slavachevsky I, Amit M, et al. The effect of spironolactone, cilazapril and their combination on albuminuria in patients with hypertension and diabetic nephropathy is independent of blood pressure reduction: a randomized controlled study. Diabet Med. 2004;21:471–5.
1105. Radwan T, Fahmy R, Emady M El, et al. Comparative study between dexmedetomidine, magnesium sulphate and fentanyl as sedatives throughout awake fiberoptic intubation for patients undergoing cervical spine surgeries. Egypt J Anaesth. 2017;33:345–349.
1106. Ragab A, Goda H, Raghib M, et al. Does immediate postpartum curettage of the endometrium accelerate recovery from preeclampsia-eclampsia? a randomized controlled trial. Arch Gynecol Obstet. 2013;288:1035–8.
1107. Rahi R, Vijyendra K, Sharma SP, et al. A comparative study of intratumoral chemotherapy in advanced childhood common solid tumors. Indian J Urol. 2007;23:358–65.
1108. Ramesh J, Bang JY, Hébert-Magee S, et al. Randomized trial comparing the flexible 19G and 25G needles for endoscopic ultrasound-guided fine needle aspiration of solid pancreatic mass lesions. Pancreas. 2015;44:128–33.
1109. Rasheedy R, Tamara TF, Allam IS, et al. Vaginal misoprostol before copper IUD insertion after previous insertion failure: a double-blind, placebo-controlled, parallel-group, randomised clinical trial. Eur J Contracept Reprod Heal Care. 2019;24:222–6.

1110. Raz I, Ziegler AG, Linn T, et al. Treatment of recent-onset type 1 diabetic patients with DiaPep277: results of a double-blind, placebo-controlled, randomized phase 3 trial. Diabetes Care. 2014;37:1392–400.
1111. Reductio TSM. Impact of renin-angiotensin system inhibition on microalbuminuria in type 2 diabetes: a post hoc analysis of the shiga microalbuminuria reduction trial (SMART). Hypertens Res Off J Japanese Soc Hypertens. 2008;31:1171–6.
1112. Reuben SS, Buvanendran A, Kroin JS, et al. Postoperative modulation of central nervous system prostaglandin E2 by cyclooxygenase inhibitors after vascular surgery. Anesthesiology. 2006;104:411–6.
1113. Reuben SS, Buvenandran A, Katz B, et al. A prospective randomized trial on the role of perioperative celecoxib administration for total knee arthroplasty: improving clinical outcomes. Anesth Analg. 2008;106:1258–64.
1114. Reuben SS, Connelly NR, Maciolek H. Postoperative analgesia with controlled-release oxycodone for outpatient anterior cruciate ligament surgery. Anesth Analg. 1999;88(6):1286-1291.
1115. Reuben SS, Connelly NR. Postarthroscopic meniscus repair analgesia with intraarticular ketorolac or morphine. Anesth Analg. 1996;82(5):1036-1039.
1116. Reuben SS, Connelly NR. Postoperative analgesic effects of celecoxib or rofecoxib after spinal fusion surgery. Anesth Analg. 2000;91:1221–5.
1117. Reuben SS, Ekman EF, Charron D. Evaluating the analgesic efficacy of administering celecoxib as a component of multimodal analgesia for outpatient anterior cruciate ligament reconstruction surgery. Anesth Analg. 2007;105(1):222-227.
1118. Reuben SS, Ekman EF, Raghunathan K, et al. The effect of cyclooxygenase-2 inhibition on acute and chronic donor-site pain after spinal-fusion surgery. Reg Anesth Pain Med. 2006;31:6–13.
1119. Reuben SS, Ekman EF. The effect of initiating a preventive multimodal analgesic regimen on long-term patient outcomes for outpatient anterior cruciate ligament reconstruction surgery. Anesth Analg. 2007;105:228–32.
1120. Reuben SS, Fingeroth R, Krushell R, et al. Evaluation of the safety and efficacy of the perioperative administration of rofecoxib for total knee arthroplasty. J Arthroplasty. 2002;17:26–31.
1121. Reuben SS, Makari-Judson G, Lurie SD. Evaluation of efficacy of the perioperative administration of venlafaxine XR in the prevention of postmastectomy pain syndrome. J Pain Symptom Manage. 2004;27(2):133-139.
1122. Reuben SS, Reuben JP. Brachial plexus anesthesia with verapamil and/or morphine. Anesth Analg. 2000;91(2):379-383.
1123. Reuben SS, Rosenthal EA, Steinberg RB, et al. Surgery on the affected upper extremity of patients with a history of complex regional pain syndrome: the use of intravenous regional anesthesia with clonidine. J Clin Anesth. 2004;16:517–22.
1124. Reuben SS, Steinberg RB, Maciolek H, et al. An evaluation of the analgesic efficacy of intravenous regional anesthesia with lidocaine and ketorolac using a forearm versus upper arm tourniquet. Anesth Analg. 2002;95(2):457-60.
1125. Reuben SS, Vieira P, Faruqi S, et al. Local administration of morphine for analgesia after iliac bone graft harvest. Anesthesiology. 2001;95:390–4.
1126. Reyes-Izquierdo T, Phelan MJ, Keller R, et al. Short-term efficacy of a combination of glucosamine and chondroitin sulfate compared to a combination of glucosamine, chondroitin sulfate and calcium fructoborate (CFB) on improvement of knee discomfort conditions in healthy subjects. A comparative, double-blind, placebo controlled acute clinical study. J Aging Res Clin Pract. 2014;3(4):223–228.
1127. Rezk M, Sayyed T, Masood A, et al. Nicorandil vs nifedipine for the treatment of preterm labour: a randomized clinical trial. Eur J Obstet Gynecol Reprod Biol. 2015;195:27–30.

1128. Riesmeier A, Schellhaass A, Boldt J, et al. Crystalloid/colloid versus crystalloid intravascular volume administration before spinal anesthesia in elderly patients: the influence on cardiac output and stroke volume. Anesth Analg. 2009;108:650–4.
1129. Rigby JH, Draper DO. Effects of long duration low intensity ultrasound for active trapezius trigger points: a randomized clinical trial. J Sport Rehabil. 2017;1–17.
1130. Roberts JT, von der Maase H, Sengeløv L, et al. Long-term survival results of a randomized trial comparing gemcitabine/cisplatin and methotrexate/vinblastine/doxorubicin/cisplatin in patients with locally advanced and metastatic bladder cancer. Ann Oncol. 2006;17:v118–22.
1131. Roehm KD, Piper SN, Maleck WH, et al. Prevention of propofol-induced injection pain by remifentanil: a placebo-controlled comparison with lidocaine. Anaesthesia. 2003;58(2):165-170.
1132. Roh YH, Hong SW, Gong HS, et al. Ultrasound-guided versus blind corticosteroid injections for De Quervain tendinopathy: a prospective randomized trial. J Hand Surg Eur Vol. 2018;43:820–4.
1133. Roh YH, Song JH, Gong HS, et al. Comparison of clinical outcomes after ulnar shortening osteotomy for ulnar impaction syndrome with or without arthroscopic debridement. J Hand Surg Eur Vol. 2019; 44(6):589-593.
1134. Röhm KD, Piper SN, Suttner S, et al. Early recovery, cognitive function and costs of a desflurane inhalational vs. a total intravenous anaesthesia regimen in long-term surgery. Acta Anaesthesiol Scand. 2006;50:14–8.
1135. Röhm KD, Riechmann J, Boldt J, et al. Do patients profit from physostigmine in recovery from desflurane anaesthesia? Acta Anaesthesiol Scand. 2007;51:278–83.
1136. Röhm KD, Riechmann J, Boldt J, et al. Physostigmine for the prevention of postanaesthetic shivering following general anaesthesia - a placebo-controlled comparison with nefopam. Anaesthesia. 2005;60:433–8.
1137. Röhm KD, Schöllhorn TA, Gwosdek MJ, et al. Do we necessarily need local anaesthetics for venous cannulation? A comparison of different cannula sizes. Eur J Anaesthesiol. 2004;21(3):214-216.
1138. Röhm KD, Suttner SW, Boldt J, et al. Insignificant effect of desflurane-fentanyl-thiopental on hepatocellular integrity - a comparison with total intravenous anaesthesia using propofol-remifentanil. Eur J Anaesthesiol. 2005;22:209–14.
1139. Ronghui S. The Reasearch on the Anti-Fatigue Effect of Whey Protein Powder in Basketball Training. Open Biomed Eng J. 2015;9:330-334.
1140. Rosén A, Lekander M, Jensen K, et al. The effects of positive or neutral communication during acupuncture for relaxing effects: a sham-controlled randomized trial. Evidence-based Complement Altern Med. 2016;2016: 3925878.
1141. Rudner M, Foo C, Rönnberg J, et al. Phonological mismatch makes aided speech recognition in noise cognitively taxing. Ear Hear. 2007;28:879–92.
1142. Ruiz-Tovar J, Carbajo MA, Jimenez JM, et al. Long-term follow-up after sleeve gastrectomy versus Roux-en-Y gastric bypass versus one-anastomosis gastric bypass: a prospective randomized comparative study of weight loss and remission of comorbidities. Surg Endosc. 2019;33:401–10.
1143. Rupasinghe CD, Kantas T, Sani R, et al. Comparison of high-statin therapy vs moderate-statin therapy in achieving positive low-density lipoprotein change in patients after acute coronary syndrome: a randomized-control trial. Cureus. 2021;13: e20710
1144. Saad K, Abdel-Rahman AA, Elserogy YM, et al. Randomized controlled trial of vitamin D supplementation in children with autism spectrum disorder. J Child Psychol Psychiatry. 2018;59:20–9.
1145. Safan TF, Mohamed AA, Ragab AS. Priming with different doses of metoclopramide preceded by tourniquet alleviates propofol induced pain: a comparative study with lidocaine. Egypt J Anaesth. 2018;34:107–11.

1146. Safarinejad MR, Asgari MA, Hosseini SY, et al. A double-blind placebo-controlled study of the efficacy and safety of pentoxifylline in early chronic Peyronie's disease. BJU Int. 2010;106:240–8.
1147. Safarinejad MR, Azma K, Kolahi AA. The effects of intensive, long-term treadmill running on reproductive hormones, hypothalamus-pituitary-testis axis, and semen quality: a randomized controlled study. J Endocrinol. 2009;200:259–71.
1148. Safarinejad MR, Hosseini SY. Safety and efficacy of tramadol in the treatment of idiopathic detrusor overactivity: a double-blind, placebo-controlled, randomized study. Br J Clin Pharmacol. 2006;61:456–63.
1149. Safarinejad MR, Hosseini SY. Safety and efficacy of tramadol in the treatment of premature ejaculation: a double-blind, placebo-controlled, fixed-dose, randomized study. J Clin Psychopharmacol. 2006;26:27–31.
1150. Safarinejad MR, Taghva A, Shekarchi B, et al. Safety and efficacy of sildenafil citrate in the treatment of Parkinson-emergent erectile dysfunction: a double-blind, placebo-controlled, randomized study. Int J Impot Res. 2010;22:325–35.
1151. Safarinejad MR. Efficacy and safety of omega-3 for treatment of eary-stage Peyronie's disease: a prospective, randomized, double-blind placebo-controlled study. J Sex Med. 2009;6:1743–54.
1152. Safarinejad MR. Evaluation of the safety and efficacy of bremelanotide, a melanocortin receptor agonist, in female subjects with arousal disorder: a double-blind placebo-controlled, fixed dose, randomized study. J Sex Med. 2008;5:887–97.
1153. Safarinejad MR. Once-daily high-dose pindolol for paroxetine-refractory premature ejaculation: a double-blind, placebo-controlled and randomized Study. J Clin Psychopharmacol. 2008;28:39–44.
1154. Safarinejad MR. Safety and efficacy of escitalopram in the treatment of premature ejaculation: a double-blind, placebo-controlled, fixed-dose, randomized study. J Clin Psychopharmacol. 2007;27:444–50.
1155. Safarinejad MR. The effects of the adjunctive bupropion on male sexual dysfunction induced by a selective serotonin reuptake inhibitor: a double-blind placebo-controlled and randomized study. BJU Int. 2010;106:840–7.
1156. Saitoh Y, Fujii Y, Makita K, et al. Modified double burst stimulation of varying stimulating currents. Acta Anaesthesiol Scand. 1998;42(7):851-857.
1157. Saitoh Y, Fujii Y, Oshima T. The ulinastatin-induced effect on neuromuscular block caused by vecuronium. Anesth Analg. 1999;89:1565-9.
1158. Saitoh Y, Fujii Y, Takahashi K, et al. Recovery of post-tetanic count and train-of-four responses at the great toe and thumb. Anaesthesia. 1998;53:244–8.
1159. Saitoh Y, Hattori H, Sanbe N, et al. Delayed recovery of vecuronium neuromuscular block in diabetic patients during sevoflurane anesthesia. Can J Anesth Can d'anesthésie. 2005;52:467–73.
1160. Saitoh Y, Kaneda K, Fujii Y, et al. Nicorandil accelerates recovery of neuromuscular block caused by vecuronium. Can J Anesth. 2001;48:28-33.
1161. Saitoh Y, Kaneda K, Hattori H, et al. Monitoring of neuromuscular block after administration of vecuronium in patients with diabetes mellitus. Br J Anaesth. 2003;90:480–6.
1162. Saitoh Y, Kaneda K, Tokunaga Y, et al. Infusion of amino acid enriched solution hastens recovery from neuromuscular block caused by vecuronium. Br J Anaesth. 2001;86:814–21.
1163. Saitoh Y, Kaneda K, Toyooka H, et al. Post-tetanic count and single twitch height at the onset of reflex movement after administration of vecuronium under different types of anaesthesia. Br J Anaesth. 1994,72(6):688-90.
1164. Saitoh Y, Masuda A, Toyooka H, et al. Effect of tetanic stimulation on subsequent train-of-four responses at various levels of vecuronium-induced neuromuscular block. Br J Anaesth. 1994;73(3):416-417.

1165. Saitoh Y, Nakajima H, Hattori H, et al. Neuromuscular blockade can be assessed accelerographically over the vastus medialis muscle in patients positioned prone. Can J Anaesth. 2003;50(4):342-347.
1166. Saitoh Y, Nakata Y, Sashiyama H, et al. Assessment of neuromuscular block at the orbicularis oris, corrugator supercilii, and adductor pollicis muscles. J Anesth. 2012;26:28–33.
1167. Saitoh Y, Nakazawa K, Makita K, et al. Visual evaluation of train-of-four and double burst stimulation, fade at various currents, using a rubber band. Eur J Anaesthesiol. 1997;14:327–32.
1168. Saitoh Y, Narumi Y, Fujii Y, et al. Tactile evaluation of fade of the train-of-four and double-burst stimulation using the anaesthetist's non-dominant hand. Br J Anaesth. 1999;83(2):275-278.
1169. Saitoh Y, Narumi Y, Fujii Y. Post-tetanic count and train-of-four responses during neuromuscular block produced by vecuronium and infusion of nicardipine. Br J Anaesth. 1999;83:340-2.
1170. Saitoh Y, Tanaka H, Fujii Y, et al. Post-tetanic burst count and train-of-four during recovery from vecuronium-induced intense neuromuscular block under different types of anaesthesia. Eur J Anaesthesiol. 1998;15:524-8.
1171. Saitoh Y, Tanaka H, Toyooka H, et al. Recovery of post-tetanic and train-of-four responses at the first dorsal interosseous and adductor pollicis muscles in patients receiving vecuronium. Can J Anesth. 1996;43:362–7.
1172. Saitoh Y, Toyooka H, Amaha K. Post-tetanic burst: a new monitoring method for intense neuromuscular block. Br J Anaesth. 1995, 74(3):293-5.
1173. SAITOH Y, TOYOOKA H, AMAHA K. Recoveries of post-tetanic twitch and train-of-four responses after administration of vecuronium with different inhalation anaesthetics and neuroleptanaesthesia. Br J Anaesth. 1993;70:402–4.
1174. Saitoh Y, Toyooka H, Amaha K. Relationship between post-tetanic twitch and single twitch response after administration of vecuronium. Br J Anaesth. 1993;71(3):443-444.
1175. Saiton Y, Nakazawa K, Makita K, et al. Evaluation of residual neuromuscular blockade using modified double burst stimulation. Acta Anaesthesiol Scand. 1997;41:741–5.
1176. Sakr MF, Moussa MM. LigaSure hemorrhoidectomy versus stapled hemorrhoidopexy: a prospective, randomized clinical trial. Dis Colon Rectum. 2010;53:1161–7.
1177. Sakr SA, Gomaa GF, Osama M. A novel technique of multi-track percutaneous balloon mitral commissurotomy (PBMC). Egypt J Chest Dis Tuberc. 2013; 61:223-228.
1178. Salehi I, Hosseini SM, Haghighi M, et al. Electroconvulsive therapy and aerobic exercise training increased BDNF and ameliorated depressive symptoms in patients suffering from treatment-resistant major depressive disorder. J Psychiatr Res. 2014;57:117–24.
1179. Salek M, Nasiri SJ, Amoli HA, et al. Promising results for hypospadias repair using alloderm® (Regen): a randomized controlled trial. J Pediatr Surg. 2021;56:1623–7.
1180. Samaha AA, Mouawia H, Fawaz M, et al. Effects of a single dose of ivermectin on viral and clinical outcomes in asymptomatic SARS-CoV-2 infected subjects: a pilot clinical trial in Lebanon. Viruses. 2021;13:989.
1181. Sanad AS, Mahran AE, Aboulfotouh ME, et al. The effect of uterine artery ligation in patients with central placenta pevia: a randomized controlled trial. BMC Pregnancy Childbirth. 2018;18(1):351.

1182. Sannino G, De Falco I, De Pietro G, et al. The Effects of Physical Exercise on Cognition: How Heart Rate Variability Can Predict Cognitive Performances. Front Hum Neurosci. 2020;14:312.
1183. Sato Y, Asoh T, Kaji M, et al. Beneficial effect of intermittent cyclical etidronate therapy in hemiplegic patients following an acute stroke. J Bone Miner Res. 2000;15:2487–94.
1184. Sato Y, Honda Y, Iwamoto J, et al. Comparison of non-vertebral fracture between minodronate and risedronate therapy in elderly female patients with Alzheimer disease. J Musculoskelet Neuronal Interact. 2013;13(3):346-352.
1185. Sato Y, Honda Y, Iwamoto J, et al. Effect of folate and mecobalamin on hip fractures in patients with stroke: a randomized controlled trial. Jama. 2005;293:1082–8.
1186. Sato Y, Honda Y, Iwamoto J. Etidronate for fracture prevention in amyotrophic lateral sclerosis: a randomized controlled trial. Bone. 2006;39:1080–6.
1187. Sato Y, Honda Y, Iwamoto J. Risedronate and ergocalciferol prevent hip fracture in elderly men with Parkinson disease. Neurology. 2007;68:911–5.
1188. Sato Y, Honda Y, Kaji M, et al. Amelioration of osteoporosis by menatetrenone in elderly female Parkinson’s disease patients with vitamin D deficiency. Bone. 2002;31:114–8.
1189. Sato Y, Honda Y, Kuno H, et al. Menatetrenone ameliorates osteopenia in disuse-affected limbs of vitamin D- and K-deficient stroke patients. Bone. 1998;23:291–6.
1190. Sato Y, Honda Y, Umeno K, et al. The prevention of hip fracture with menatetrenone and risedronate plus calcium supplementation in elderly patients with Alzheimer disease: a randomized controlled trial. Kurume Med J. 2011;57:117–24.
1191. Sato Y, Iwamoto J, Honda Y. Amelioration of osteoporosis and hypovitaminosis D by sunlight exposure in Parkinson's disease. Parkinsonism Relat Disord. 2011;17(1):22-26.
1192. Sato Y, Iwamoto J, Honda Y. An open-label trial comparing alendronate and alphacalcidol in reducing falls and hip fractures in disabled stroke patients. J Stroke Cerebrovasc Dis. 2011;20:41–6.
1193. Sato Y, Iwamoto J, Honda Y. Beneficial effect of etidronate therapy in chronically hospitalized, disabled patients with stroke. J Stroke Cerebrovasc Dis. 2010;19:198–203.
1194. Satō Y, Iwamoto J, Honda Y. Once-weekly risedronate for prevention of hip fracture in women with Parkinson’s disease: a randomised controlled trial. J Neurol Neurosurg Psychiatry. 2011;82:1390–3.
1195. Sato Y, Iwamoto J, Kanoko T, et al. Amelioration of osteoporosis and hypovitaminosis D by sunlight exposure in hospitalized, elderly women with Alzheimer’s disease: a randomized controlled trial. J Bone Miner Res. 2005;20:1327–33.
1196. Sato Y, Iwamoto J, Kanoko T, et al. Low-dose vitamin D prevents muscular atrophy and reduces falls and hip fractures in women after stroke: a randomized controlled trial. Cerebrovasc Dis. 2005;20:187–92.
1197. Sato Y, Iwamoto J, Kanoko T, et al. Risedronate sodium therapy for prevention of hip fracture in men 65 years or older after stroke. Arch Intern Med. 2005;165:1743-8.
1198. Sato Y, Iwamoto J, Kanoko T, Satoh K. Alendronate and vitamin D2 for prevention of hip fracture in Parkinson's disease: a randomized controlled trial. Mov Disord. 2006;21(7):924-929.
1199. Sato Y, Iwamoto J, Kanoko T, Satoh K. Risedronate therapy for prevention of hip fracture after stroke in elderly women. Neurology. 2005 Mar 8;64(5):811-6.

1200. Sato Y, Kanoko T, Satoh K, et al. Menatetrenone and vitamin D2 with calcium supplements prevent nonvertebral fracture in elderly women with Alzheimer's disease. Bone. 2005;36:61–8.
1201. Sato Y, Kanoko T, Satoh K, et al. The prevention of hip fracture with risedronate and ergocalciferol plus calcium supplementation in elderly women with Alzheimer disease: a randomized controlled trial. Arch Intern Med. 2005;165:1737-42.
1202. Sato Y, Kanoko T, Yasuda H, et al. Beneficial effect of etidronate therapy in immobilized hip fracture patients. Am J Phys Med Rehabil. 2004;83:298–303.
1203. Sato Y, Manabe S, Kuno H, et al. Amelioration of osteopenia and hypovitaminosis D by 1α-hydroxyvitamin D3 in elderly patients with Parkinson's disease. J Neurol Neurosurg Psychiatry. 1999;66:64–8.
1204. Sato Y, Maruoka H, Oizumi K. Amelioration of hemiplegia-associated osteopenia more than 4 years after stroke by 1α-hydroxyvitamin D 3 and calcium supplementation. Stroke. 1997;28:736–9.
1205. Sato Y, Metoki N, Iwamoto J, et al. Amelioration of osteoporosis and hypovitaminosis D by sunlight exposure in stroke patients. Neurology. 2003;61:338–42.
1206. Sato Y. Efficacy of methylprednisolone pulse therapy on neuroleptic malignant syndrome in Parkinson's disease. J Neurol Neurosurg Psychiatry. 2003;74:574–6.
1207. Satouchi M, Nosaki K, Takahashi T, et al. First-line pembrolizumab vs chemotherapy in metastatic non-small-cell lung cancer: KEYNOTE-024 Japan subset. Cancer Sci. 2020;111:4480–9.
1208. Sawada T, Yamada H, Dahlöf B, et al. Effects of valsartan on morbidity and mortality in uncontrolled hypertensive patients with high cardiovascular risks: KYOTO heart study. Eur Heart J. 2009;30:2461–9.
1209. Scabini S, Rimini E, Romairone E, et al. Colon and rectal surgery for cancer without mechanical bowel preparation: one-center randomized prospective trial. World J Surg Oncol. 2010, 30:8:35.
1210. Schietroma M, Carlei F, Cecilia EM, et al. Colorectal infraperitoneal anastomosis: the effects of perioperative supplemental oxygen administration on the anastomotic dehiscence. J Gastrointest Surg. 2012;16:427–34.
1211. Schietroma M, Cecilia EM, Carlei F, et al. Dexamethasone for the prevention of recurrent laryngeal nerve palsy and other complications after thyroid surgery: a randomized double-blind placebo-controlled trial. JAMA Otolaryngol - Head Neck Surg. 2013;139:471–8.
1212. Schietroma M, Cecilia EM, Sista F, et al. High-concentration supplemental perioperative oxygen and surgical site infection following elective colorectal surgery for rectal cancer: a prospective, randomized, double-blind, controlled, single-site trial. Am J Surg. 2014;208:719–26.
1213. Schietroma M, Piccione F, Carlei F, et al. Peritonitis from perforated peptic ulcer and immune response. J Invest Surg. 2013;26(5):294-304.
1214. Schietroma M, Piccione F, Cecilia EM, et al. How does high-concentration supplemental perioperative oxygen influence surgical outcomes after thyroid surgery? A prospective, randomized, double-blind, controlled, monocentric trial. J Am Coll Surg. 2015;220:921–33.
1215. Schmidt C, Suttner S, Piper SN, et al. Comparison of the effects of desflurane and isoflurane anaesthesia on hepatocellular function assessed by alpha glutathione S-transferase. Anaesthesia. 1999;54:1207–11.
1216. Schulz-Stübner S, Henszel A, Hata JS. A new rule for femoral nerve blocks. Reg Anesth Pain Med. 2005;30:473–7.
1217. Sedghipour MR, Lotfi A, Sadeghilar A, et al. Efficacy and safety of cross-cylinder photorefractive keratectomy versus single method in medium-high astigmatism: a randomized clinical trial. Clin Exp Ophthalmol. 2012.

1218. Seif NE, ELbadawy AM. Comparative study of mid-thoracic spinal versus epidural anesthesia for open nephrectomy in patients with obstructive/restrictive lung disease: A randomized controlled study. Saudi J Anaesth. 2019;13(1):52-59.
1219. Sell A, Tein T, Pitkänen M. Spinal 2-chloroprocaine: effective dose for ambulatory surgery. Acta Anaesthesiol Scand. 2008;52:695–9.
1220. Seo KY, Kim DH, Lee SE, et al. Skin rejuvenation by microneedle fractional radiofrequency and a human stem cell conditioned medium in Asian skin: a randomized controlled investigator blinded split-face study. J Cosmet Laser Ther. 2013;15:25–33.
1221. Sepehrmanesh Z, Kolahdooz F, Abedi F, et al. Vitamin D supplementation affects the beck depression inventory, insulin resistance, and biomarkers of oxidative stress in patients with major depressive disorder: a randomized, controlled clinical trial. J Nutr. 2016;146:243–8.
1222. Seto H, Ikeda H, Hisaoka H, et al. Effect of heat- and steam-generating sheet on daily activities of living in patients with osteoarthritis of the knee: randomized prospective study. J Orthop Sci. 2008;13:187–91.
1223. Shahamfar M, Azima N, Erfanparast L. A randomized split mouth clinical trial comparing mineral trioxide aggregate with a new fast-setting calcium silicate cement in direct pulp capping of primary molars: a preliminary report from a long-term follow-up. Int J Clin Pediatr Dent. 2020;13:390–4.
1224. Shahar S, Aziz AF, Ismail SNA, et al. The effect of Polygonum minus extract on cognitive and psychosocial parameters according to mood status among middle-aged women: a randomized, double-blind, placebo-controlled study. Clin Interv Aging. 2015;10:1505-20.
1225. Shang E, Geiger N, Sturm JW, et al. Pump-assisted enteral nutrition can prevent aspiration in bedridden percutaneous endoscopic gastrostomy patients. J Parenter Enter Nutr. 2004;28:180–3.
1226. Shang E, Geiger N, Sturm JW, et al. Pump-assisted versus gravity-controlled enteral nutrition in long-term percutaneous endoscopic gastrostomy patients: a prospective controlled trial. JPEN J Parenter Enteral Nutr. 2003;27(3):216-9.
1227. Shang E, Hasenberg T, Magdeburg R, et al. First experiences with a circular stapled gastro-jejunostomy by a new transorally introducible stapler system in laparoscopic roux-en-y gastric bypass. Obes Surg. 2009;19:230–6.
1228. Shang E, Hasenberg T. Aerobic endurance training improves weight loss, body composition, and co-morbidities in patients after laparoscopic Roux-en-Y gastric bypass. Surg Obes Relat Dis. 2010;6(3):260-266.
1229. Sharma SK, Agrawal S, Damodaran D, et al. CPAP for the metabolic syndrome in patients with obstructive sleep apnea. N Engl J Med. 2011;365:2277–86.
1230. Shehata NAA. Calcium versus oral contraceptive pills containing drospirenone for the treatment of mild to moderate premenstrual syndrome: a double blind randomized placebo controlled trial. Eur J Obstet Gynecol Reprod Biol. 2016;198:100–4.
1231. Sheikh M, Hantoushzadeh S, Shariat M, et al. The efficacy of early iron supplementation on postpartum depression, a randomized double-blind placebo-controlled trial. Eur J Nutr. 2017;56:901–8.
1232. Shi C, Jin J, Qiao L, et al. Effect of perioperative administration of dexmedetomidine on delirium after cardiac surgery in elderly patients: a double-blinded, multi-center, randomized study. Clin Interv Aging. 2019; 14:571–5.
1233. Shiga Microalbuminuria Reduction Trial (SMART) Group, Uzu T, Sawaguchi M, Maegawa H, Kashiwagi A. Reduction of microalbuminuria in patients with type 2 diabetes: the Shiga Microalbuminuria Reduction Trial (SMART). Diabetes Care. 2007;30(6):1581-1583.
1234. Shimazaki A, Ueshima H, Otake H. Endotrol tracheal tube and McGrath Mac are an effective combination for oral tracheal intubation. Saudi J Anaesth. 2018;12:72–6.
1235. Shin KR, Kang Y, Park HJ, et al. Effects of exercise program on physical fitness, depression, and self-efficacy of low-income elderly women in South Korea. Public Health Nurs. 2009;26(6):523-531.

1236. Shokeir T, El-Shafei M, Yousef H, et al. Submucous myomas and their implications in the pregnancy rates of patients with otherwise unexplained primary infertility undergoing hysteroscopic myomectomy: a randomized matched control study. Fertil Steril. 2010;94:724–9.
1237. Shokeir T, Shalaby H, Nabil H, et al. Reducing blood loss at abdominal myomectomy with preoperative use of dinoprostone intravaginal suppository: a randomized placebo-controlled pilot study. Eur J Obstet Gynecol Reprod Biol. 2013;166:61–4.
1238. Singh R, Ojha S, Choubey S. A comparative study of dexmedetomidine and diltiazem for attenuating pressor responses to laryngoscopy and endotracheal intubation: a double-blind, randomized study. Anesth Essays Res. 2017;11:921-929.
1239. Song F, Ye C, Feng Q, et al. Effect of perioperative infusion of Dexmedetomidine combined with Sufentanil on quality of postoperative analgesia in patients undergoing laparoscopic nephrectomy: a CONSORT-prospective, randomized, controlled trial. BMC Anesthesiol. 2018;18(1):145.
1240. Stang J, Couto M, Carlsen KH, et al. Increased bronchial parasympathetic tone in elite cross-country and biathlon skiers: a randomised crossover studyBr J Sports Med.2014.
1241. Steenbergen L, Sellaro R, Stock AK, et al. Transcutaneous vagus nerve stimulation (tVNS) enhances response selection during action cascading processes. Eur Neuropsychopharmacol. 2015;25(6):773-778.
1242. Steenbergen L, Sellaro R, Stock A-K, et al. γ-Aminobutyric acid (GABA) administration improves action selection processes: a randomised controlled trial. Sci Rep. 2015;5:12770
1243. Stein JA, Ramirez M, Heinrich KM. The Effects of Acute Caffeine Supplementation on Performance in Trained CrossFit Athletes. Sports (Basel). 2019;7(4):95.
1244. Stein-Gold L, Kircik LH, Draelos ZD, et al. Efficacy and safety of topical oxymetazoline cream 1.0% for treatment of persistent facial erythema associated with rosacea: findings from the 2 phase 3, 29-day, randomized, controlled REVEAL trials. J Am Acad Dermatol. 2018:S0190-9622(18)30147-6.
1245. Stephens RC, O'Malley CM, Frumento RJ, et al. Low-dose endotoxin elicits variability in the inflammatory response in healthy volunteers. J Endotoxin Res. 2005;11(4):207-212.
1246. Strasser H, Marksteiner R, Margreiter E, et al. Autologous myoblasts and fibroblasts versus collagen for treatment of stress urinary incontinence in women: a randomised controlled trial. Lancet. 2007;369(9580):2179-2186.
1247. Strasser H, Marksteiner R, Margreiter E, et al. Transurethral ultrasonography-guided injection of adult autologous stem cells versus transurethral endoscopic injection of collagen in treatment of urinary incontinence. World J Urol. 2007;25:385–92.
1248. Struys MM, Vanluchene AL, Gibiansky E, et al. AQUAVAN injection, a water-soluble prodrug of propofol, as a bolus injection: a phase I dose-escalation comparison with DIPRIVAN (part 2): pharmacodynamics and safety. Anesthesiology. 2005;103(4):730-743.
1249. Suherman SK, Affandi B, Korver T. The effects of Implanon on lipid metabolism in comparison with Norplant. Contraception. 1999;60:281–7.
1250. Sun J, Yang C, Zhao H, et al. Randomised clinical trial: the clinical efficacy and safety of an alginate-antacid (Gaviscon Double Action) versus placebo, for decreasing upper gastrointestinal symptoms in symptomatic gastroesophageal reflux disease (GERD) in China. Aliment Pharmacol Ther. 2015;42:845–54.
1251. Sun LJ, Yu XB, Dai CQ, et al. A randomised prospective study of two different combined internal and external fixation techniques for distal tibia shaft fractures. Injury. 2014 Dec;45(12):1990-5.
1252. Sun QH, Wang HY, Sun SD, et al. Beneficial effect of probiotics supplements in reflux esophagitis treated with esomeprazole: a randomized controlled trial. World J Gastroenterol. 2019;25:2110–21.

1253. Sun Z, Su W, Wang L, et al. Clinical Effect of Bushen Huoxue Method Combined with Platelet-Rich Plasma in the Treatment of Knee Osteoarthritis and Its Effect on IL-1, IL-6, VEGF, and PGE-2. J Healthc Eng. 2022;2022:9491439.
1254. Sun Z, Zhu Z, Yang G, et al. The 95% effective dose of nalbuphine in patient-controlled intravenous analgesia for patients undergoing laparoscopic total hysterectomy compared to equivalent sufentanil. Medicine (Baltimore). 2020;99:e20424.
1255. Sung VW, Richter HE, Moalli P, et al. Characteristics associated with treatment failure 1 year after midurethral sling in women with mixed urinary incontinence. Obstet Gynecol. 2020;136:482–91.
1256. Suttner S, Boldt J, Schmidt C, et al. Cost analysis of target-controlled infusion-based anesthesia compared with standard anesthesia regimens. Anesth Analg. 1999;88:77–82.
1257. Suttner S, Lang K, Boldt J, et al. The influence of hyperoxic ventilation during sodium nitroprusside-induced hypotension on skeletal muscle tissue oxygen tension. Anesthesiology. 2002;96:1103–8.
1258. Suttner S, Piper SN, Kumle B, et al. The influence of allogeneic red blood cell transfusion compared with 100% oxygen ventilation on systemic oxygen transport and skeletal muscle oxygen tension after cardiac surgery. Anesth Analg. 2004;99:2–11.
1259. Suttner SW, Boldt J, Schmidt CC, et al. The effects of sodium nitroprusside-induced hypotension on splanchnic perfusion and hepatocellular integrity. Anesth Analg. 1999;89:1371.
1260. Suttner SW, Schmidt CC, Boldt J, et al. Low-flow desflurane and sevoflurane anesthesia minimally affect hepatic integrity and function in elderly patients. Anesth Analg. 2000;91:206–12.
1261. Tahir M, Chaudhry EA, Zimri FK, et al. Negative pressure wound therapy versus conventional dressing for open fractures in lower extremity trauma: a multicentre randomized controlled trial. Bone Jt J. 2020;102:912–7.
1262. Tahir MM, Khan DN, Chaudhry MEA, et al. A multicentre randomized controlled trial comparing plating with intramedullary nailing for extra-articular distal tibial fractures. Injury. 2021;52: 19-25
1263. Tang G, Hu Y, Yin S, et al. β-Carotene in golden rice is as good as β-carotene in oil at providing vitamin A to children. Am J Clin Nutr. 2012;96:658–64.
1264. Tejedor J, Ogallar C, Rodríguez JM. Surgery for esotropia under topical anesthesia. Ophthalmology. 2010;117:1883–8.
1265. Tildesley HD, Wright AM, Chan JH, et al. A comparison of internet monitoring with continuous glucose monitoring in insulin-requiring type 2 diabetes mellitus. Can J Diabetes. 2013 Oct;37(5):305-8.
1266. Torky H, Shata A, Ahmad A, et al. Effect of amlodipine on blood flow of preovulatory follicle in women with clomiphene resistant polycystic ovaries: a randomized controlled trial. Arch Gynecol Obstet. 2020;301:845–50.
1267. Tsay S-L, Rong J-R, Lin P-F. Acupoints massage in improving the quality of sleep and quality of life in patients with end-stage renal disease. J Adv Nurs. 2003;42:134–42.
1268. Tulgar S, Kapakli M, Kose H, et al. Evaluation of ultrasound-guided erector spinae plane block and oblique subcostal transversus abdominis plane block in laparoscopic cholecystectomy: randomized, controlled, prospective study. Anesth Essays Res. 2019;13:50-56
1269. Ueshima H, Hara E, Otake H. Thoracolumbar interfascial plane block provides effective perioperative pain relief for patients undergoing lumbar spinal surgery; a prospective, randomized and double blinded trial. J Clin Anesth. 2019;58:12–7.
1270. Ueshima H, Otake H. Addition of transversus thoracic muscle plane block to pectoral nerves block provides more effective perioperative pain relief than pectoral nerves block alone for breast cancer surgery. Br J Anaesth. 2017;118(3):439-443.

1271. Ueshima H, Tanaka N, Otake H. Greater analgesic effect with intermittent compared with continuous mode of lumbar plexus block for total hip arthroplasty: a randomized controlled trial. Reg Anesth Pain Med. 2019;44:632–6.
1272. Unlugenc H, Guler T, Gunes Y, et al. Comparative study of the antiemetic efficacy of ondansetron, propofol and midazolam in the early postoperative period. Eur J Anaesthesiol. 2004;21:60–5.
1273. Van Toan N, Hanh TT. Improved treatment of Asthma by using natural sources of antioxidants. Springerplus. 2013;2(1): 278.
1274. Vashisht R, Indira R, Ramachandran S, et al. Role of casein phosphopeptide amorphous calcium phosphate in remineralization of white spot lesions and inhibition of Streptococcus mutans? J Conserv Dent. 2013;16:342-6.
1275. Vasudev A, Arena A, Burhan AM, et al. A training programme involving automatic self-transcending meditation in late-life depression: preliminary analysis of an ongoing randomised controlled trial. BJPsych Open. 2016;2:195–8.
1276. Venkatachalapathy TS, Mohankumar S, Saliba MJ. A comparative study of thermal burns treated with topical heparin and without heparin. Indian J Surg. 2012;75:249.
1277. Vinson J, Nagendran M V, Burnham BR. Randomized, double-blind, placebo-controlled, linear dose, crossover study to evaluate the efficacy and safety of a green coffee bean extract in overweight subjects. Diabetes, Metab Syndr Obes Targets Ther. 2012;21-7.
1278. Walach H, Weikl R, Prentice J, et al. Experimental assessment of carbon dioxide content in inhaled air with or without face masks in healthy children. JAMA Pediatr. 2021:e212659.
1279. Wan Q, Ding W, Cui X, et al. CONSORT-epidural dexmedetomidine improves gastrointestinal motility after laparoscopic colonic resection compared with morphine. Medicine (Baltimore). 2018;97(25):e11218.
1280. Wang D, Li X, Zhang L, et al. Effects of motivational interviewing on lifestyle modification and diabetes prevention in adults with pre-diabetes. Diabetes Res Clin Pract. 2015.
1281. Wang J, Liu XF, Feng C, et al. Efficacy and safety of vortioxetine for the treatment of major depressive disorder: a randomised double-blind placebo-controlled study. Int J Psychiatry Clin Pract. 2019;23:245–50.
1282. Wang L, Zheng Y, Zhang X, et al. Controlled Hypotension Combined with Femoral Nerve Block for Knee Replacement without Tourniquet. J Healthc Eng. 2021;2021:3219337.
1283. Wang T, Yan X, Zhou Q. Effect of acupuncture on gut microbiota in participants with subjective cognitive decline. Medicine (Baltimore). 2022;101(18):e27743.
1284. Wang XH, Wang JQ, Xu Y, et al. Therapeutic effects of metformin and laparoscopic ovarian drilling in treatment of clomiphene and insulin-resistant polycystic ovary syndrome. Arch Gynecol Obstet. 2015;291:1089–94.
1285. Wang Y, Su C. The Effect of Humanistic Care Combined with Predictive Nursing on Negative Emotions and Incidence of Cardiovascular Events in Hemodialysis Patients. Comput Math Methods Med. 2022;2022:7562525.
1286. Wang Z, Yu J, Niu T, et al. Effect of Stellate Ganglion Block Combined with Lidocaine at Different Concentrations for Preemptive Analgesia on Postoperative Pain Relief and Adverse Reactions of Patients Undergoing Laparoscopic Cholecystectomy. Comput Math Methods Med. 2022;2022:6027093.
1287. Wei S, Li J. Efficacy and Safety of Temozolomide Combined with Radiotherapy in the Treatment of Malignant Glioma. J Healthc Eng. 2022;2022:3477918.
1288. Wei Z, Cao Y, Cong L, et al. Effect of metformin pretreatment on pregnancy outcome of in vitro matured oocytes retrieved from women with polycystic ovary syndrome. Fertil Steril. 2008;90:1149–54.

1289. Weiler JM, Sorkness CA, Hendeles L, et al. Randomized, Double-Blind Pilot Study to Examine the Use of Exhaled Nitric Oxide as a Bioassay for Bioequivalence of Inhaled Fluticasone Propionate. J Clin Pharmacol. 2018;58(4):448-456.
1290. Wen M, Liang Y, Shen Q, wt al. The Effect of Resourcefulness Training on Depression and Coping Styles of Patients With Coronary Heart Disease in China. J Cardiovasc Nurs. 2020.
1291. Wetzel L, Zadrazil M, Paternostro-Sluga T, et al. Intravenous nonopioid analgesic drugs in chronic low back pain patients on chronic opioid treatment: a crossover, randomised, double-blinded, placebo-controlled study. Eur J Anaesthesiol. 2014;31:35–40.
1292. Willemsen LM, Janssen PWA, Peper J, et al. Effect of adding ticagrelor to standard aspirin on saphenous vein graft patency in patients undergoing coronary artery bypass grafting (POPular CABG): a randomized, double-blind, placebo-controlled trial. Circulation. 2020;142:1799–807.
1293. Wong IKY, Andriessen A, Lee DTF, et al. Randomized controlled trial comparing treatment outcome of two compression bandaging systems and standard care without compression in patients with venous leg ulcers. J Vasc Surg. 2012;55:1376–85.
1294. Wu S, Sun Z, Li R, et al. Dexmedetomidine on continuous infraclavicular block after elbow arthrolysis. Signa Vitae. 2020;16:131-135.
1295. Xu BY, Huang YJ, Sun BG, et al. Skin-patch of Xin Huang Pian on relieving joint symptoms in patients with acute gouty arthritis: A Randomized, Double-Blind, Active-Controlled Trial. J Adv Nurs. 2020;76(6):1416-1424.
1296. Xu J, He S, Han Y, et al. Effects of modified pulmonary rehabilitation on patients with moderate to severe chronic obstructive pulmonary disease: a randomized controlled trail. Int J Nurs Sci. 2017;4:219–24.
1297. Xu T, Chen X, Li X, et al. Analysis of Anesthesia Effect of Dexmedetomidine in Clinical Operation of Replantation of Severed Finger. Comput Math Methods Med. 2021;2021:3822450.
1298. Xuan T, Wu B, Shang Y, et al. The Effect of Coenzyme A Capsule on Serum Triglyceride Concentration in Patients With Hypertriglyceridemia: Results of a Multicenter Clinical Trial. Journal of Cardiovascular Pharmacology and Therapeutics. 2013;18(5):NP1-NP1.
1299. Yan G, Zhang M, Liu Y, et al. Efficacy of vortioxetine combined cognitive behaviour intervention therapy on brain-derived neurotrophic factor level on depressive patients. Psychogeriatrics. 2019;19:475–81.
1300. Yang Y, Li Y, Zheng Y, et al. The Effect of Acupuncture Combined with Aerobic Exercise for Coronary Heart Disease as Cardiac Rehabilitation. J Healthc Eng. 2022;2022:4903265.
1301. Yao J, Wang J, Liu Y, et al. A randomized phase II study of everolimus for advanced pancreatic neuroendocrine tumors in Chinese patients. Med Oncol. 2014;31:251
1302. Ye X, Bo X, Hu X, et al. Efficacy and safety of mycophenolate mofetil in patients with active moderate-to-severe Graves' orbitopathy. Clin Endocrinol (Oxf). 2016;86:247–55.
1303. Ye X, Zhao H, Liu J, et al. Efficacy and safety of tripterygium glycosides for active moderate to severe Graves' ophthalmopathy: a randomised, observer-masked, single-centre trial. Eur J Endocrinol. 2021;184:277–87.
1304. Yin M, Pan Y. Observation of the Auxiliary Treatment Effect of Low-Frequency Nerve Therapy Instrument after Hysteroscopy for Moderate and Severe Intrauterine Adhesions Based on Intelligent Medical Treatment. J Healthc Eng. 2022;2022:2929800.
1305. You X, Liu W. Evaluation of Analgesia Effect after Ultrasound-Guided Laparoscopic Renal Surgery. Comput Math Methods Med. 2021;2021:6194806.
1306. Yousef GT, Lasheen AE. General anesthesia versus segmental thoracic or conventional lumbar spinal anesthesia for patients undergoing laparoscopic cholecystectomy. Anesth Essays Res. 2012;6(2):167-173.

1307. Youssef, J, Lee RM, Jonathan C, et al. Lavin and Dushyantha Jayaweera. “Effectiveness of ZYESAMI™ (Aviptadil) in Accelerating Recovery and Shortening Hospitalization in Critically-Ill Patients with COVID-19 Respiratory Failure: Interim Report from a Phase 2B/3 Multicenter Trial.” Social Science Research Network (2021): n. pag.
1308. Yue C, Ze-jun Y, Wu K, et al. A randomized clinical study of circumcision with a ring device versus conventional circumcision. J Urol. 2012;188:1849–54.
1309. Zarrin M, Saadat M, Yazdi MJS, et al. Efficacy of cervical manual therapy plus conventional physical therapy on clinical outcomes in patients with carpal tunnel syndrome: a double blind randomized controlled trial. Int J Clin Pract. 2021;e14371.
1310. Zeng K, Li Y, Liang M, et al. The influence of goal-directed fluid therapy on the prognosis of elderly patients with hypertension and gastric cancer surgery. Drug Des Devel Ther. 2014;8:2113-9.
1311. Zerbini L, Fabre N, Grainer A, et al. VO2p, HR and HHb kinetics in young and older adults during cycling 1 in acute hypoxia. Appl Physiol Nutr Metab. 2013;38(11).
1312. Zhang C, Yue J, Li M, et al. Bronchial blocker versus double-lumen endobronchial tube in minimally invasive cardiac surgery. BMC Pulm Med. 2019;19:207
1313. Zhang G, Gong H, Xu H. Analysis of the Mechanism and Safety of Bisphosphonates in Patients with Lung Cancer and Bone Metastases. Comput Math Methods Med. 2021;2021:5343104.
1314. Zhang J ping, Zhang N, Chen X, et al. Efficacy of dexmedetomidine as an adjunct to ropivacaine in bilateral dual-transversus abdominis plane blocks in patients with ovarian cancer who underwent cytoreductive surgery. BMC Anesthesiol. 2022;22:20
1315. Zhang LL, Jiang ZJ, Li YY, et al. Effectiveness of Home Care Interventions in Patients with Moderate to Severe Craniocerebral Injury Combined with Epilepsy. Comput Math Methods Med. 2022;2022:3654181.
1316. Zhang Q, Cao Y, Gao J, et al. Effects of cartoon violence on aggressive thoughts and aggressive behaviors. Aggress Behav. 2019; 45(5):489-497.
1317. Zhang Z, Sun X, Ma J, et al. Clinical Efficacy of Ulinastatin Combined with Meglumine Adenosine Cyclophosphate in the Treatment of Acute Myocardial Infarction. Comput Math Methods Med. 2022;2022:2172412.
1318. Zhang ZJ, Zheng ML, Nie Y, et al. Comparison of Arndt-endobronchial blocker plus laryngeal mask airway with left-sided double-lumen endobronchial tube in one-lung ventilation in thoracic surgery in the morbidly obese. Brazilian J Med Biol Res. 2017;51:e6825.
1319. Zhao F, Wang Z, Yang J, et al. Low-dosage adrenaline induces transient marked decrease of blood pressure during functional endoscopic sinus surgery. Am J Rhinol. 2006;20:182–5.
1320. Zhao H, Zhao H, Wang Y, et al. Randomized clinical trial of arginine-supplemented enteral nutrition versus standard enteral nutrition in patients undergoing gastric cancer surgery. J Cancer Res Clin Oncol. 2013;139:1465–70.
1321. Zhao Q, Du H, Liu J, et al. To Explore the Effects of Acupuncture and Medical Treatment at Different Times on the Gastrointestinal Reaction and White Blood Cell Count of Patients with Lung Cancer Chemotherapy. Appl Bionics Biomech. 2022;2022:5261344.
1322. Zhao X, Wang L, Hu Y, et al. Efficacy of Unilateral Ilioinguinal Transversus Abdominis Plane Block for Alleviation of Catheter-related Bladder Discomfort in Male Patients after Emergence from General Anesthesia: a Prospective, Randomized Controlled Trial. Research Square, 2020.
1323. Zheng X, Zhao J, Liu S, et al. Application of a surgical guide in the extraction of impacted mesiodentes: a randomized controlled trial. Clin Oral Investig. 2021;25:2999–3006.
1324. Zhu LX, Ho SC, Sit JW, et al. The effects of a transtheoretical model-based exercise stage-matched intervention on exercise behavior in patients with coronary heart disease: a randomized controlled trial. Patient Educ Couns. 2014;95(3):384-392.

1325. Zhu LX, Ho SC, Sit JW, He HG. Can the transtheoretical model motivate patients with coronary heart disease to exercise?. Nurs Health Sci. 2015;17(1):143.
1326. Zhu LX, Ho SC, Sit JWH, et al. Effect of a transtheoretical model–based stage-matched exercise intervention on exercise behavior and angina in patients with coronary heart disease. J Cardiovasc Nurs. 2014, 29(5):471.
1327. Zhu LX, Ho SC, Sit JWH, et al. Effects of a transtheoretical model-based exercise stage-matched intervention on exercise behaviour and quality of life in patients with coronary heart disease: a randomized controlled trial. J Adv Nurs. 2014;70:2414 PP-England.
1328. Zhu X, Cao Y, Liu W, et al. Stereotactic body radiotherapy plus pembrolizumab and trametinib versus stereotactic body radiotherapy plus gemcitabine for locally recurrent pancreatic cancer after surgical resection: an open-label, randomised, controlled, phase 2 trial. Lancet Oncol. 2022;23(3):e105-e115.
1329. Zolfaghari M, Mousavifar SA, Haghani H. Mobile phone text messaging and Telephone follow-up in type 2 diabetic patients for 3 months: a comparative study. J Diabetes Metab Disord. 2012;11(1):7.
1330. Zolfaghari M, Mousavifar SA, Pedram S, et al. The impact of nurse short message services and telephone follow-ups on diabetic adherence: which one is more effective? J Clin Nurs. 2012;21:1922–31.

**Supplementary Table S3. List of excluded retractions (with reasons)**

| Excluded retractions (N = 1,178) | Reasons for exclusion |
| --- | --- |
| 1 Wang Y, Gao Y, Yuan L, et al.Psychological needs and associated factors among perioperative patients with oral cancer. Oral Oncol. 2021;123:105615. | Non randomized controlled trials |
| 2 Moges K, Tadesse Y, Fentie F. The postoperative analgesia efficacy of dexamethasone added to bupivacaine versus bupivacaine alone in ultrasound-guided supraclavicular brachial plexus block for upper limb orthopedic surgeries, Ethiopia: An observational prospective cohort study. SAGE Open Med. 2022;10:20503121221126687. | Non randomized controlled trials |
| 3 Chen M, Ran B, Gao X, et al.Evaluation of occupational stress management for improving performance and productivity at workplaces by monitoring the health, well-being of workers. 2021. | Non randomized controlled trials |
| 4 Boldt J, Zickmann B, Benson M, et al. Does platelet size correlate with function in patients undergoing cardiac surgery?. Intensive care medicine, 1993, 19: 44-47. | Non randomized controlled trials |
| 5 Liu Q, Cheng J, Li J, et al. Clinical Study of Virtual Reality Augmented Technology Combined with Contrast-Enhanced Ultrasound in the Assessment of Thyroid Cancer. Journal of Healthcare Engineering, 2021: 8042755. | Non randomized controlled trials |
| 6 Gao C, Wu Y, Liu J, et al. Systematic Evaluation of the Effect of Rehabilitation of Lower Limb Function in Children with Cerebral Palsy Based on Virtual Reality Technology. Journal of Healthcare Engineering, 2021: 6625604. | Non randomized controlled trials |
| 7 Li Y, Lv Z, Han H, et al. The Relationship between Meniere's Disease and Acute Low-Tone Sensorineural Hearing Loss. Evidence-Based Complementary and Alternative Medicine, 2022: 4217131. | Non randomized controlled trials |
| 8 Shen X, Wei J, Zhang Y, et al. Analysis of Effect of Six Sigma Method Combined with CI Strategy on Improving of Nursing Quality in Outpatient Infusion Rooms. BioMed Research International, 2022: 8975435. | Non randomized controlled trials |
| 9 Huang H, Gu J, Su H, et al. Solitaire™ Stent Thrombectomy System in the Treatment of Acute Lower-Limb Ischemia: Comparisons in Safety and Effectiveness with Conventional Catheter-Directed Thrombolysis Therapy. BioMed Research International, 2022: 6997221. | Non randomized controlled trials |
| 10 Zhang Z. Clinical Observation of Botulinum Toxin Injection in the Treatment of Focal Dystonia and Muscle Spasm. BioMed Research International, 2022: 1495807. | Non randomized controlled trials |
| 11 Liu L, Men X, Song X, et al. Application Analysis of Multiacupoint Stimulation in Multimodal Labor Analgesia during the Whole Stage of Labor in Primipara. Evidence-Based Complementary and Alternative Medicine, 2022: 5161562. | Non randomized controlled trials |
| 12 Lv M, Yu S, Li Y, et al. Ultrasound Multiparametric Assessment of the Impact of Hypertensive Disorders of Pregnancy on Fetal Cardiac Function and Growth and Development. Evidence-Based Complementary and Alternative Medicine, 2022: 3419966. | Wrong study design |
| 13 Ma C, Ma Y, Lu S, et al. Clinical Study on Effect of Solution Focused Approach on the Complications, Pain, Sleep, and Quality of Life in Patients with Hepatocellular Carcinoma Undergoing TACE. Evidence-Based Complementary and Alternative Medicine, 2021: 5068228. | Non randomized controlled trials |

| | |
|---|---|
| 14 Yang L, Zhao Z. Somatostatin plus Ulinastatin in the Treatment of Severe Acute Pancreatitis and Its Effect on Serum Cytokine Levels. Evidence-Based Complementary and Alternative Medicine, 2022: 7223632. | Non randomized controlled trials |
| 15 Yu Y, Hu J, Xia Q, et al. Ultrasound Comparative Analysis of Coronary Arteries before and after Immune Blocking Therapy with Gamma Globulin in Children with Kawasaki Disease. Evidence-Based Complementary and Alternative Medicine, 2022: 2900378. | Non randomized controlled trials |
| 16 Xu J, Yu X, Wang Z. The Risk and Clinical Treatment of Hypertensive Diseases in Pregnant Women. BioMed Research International, 2022: 8480106. | Non randomized controlled trials |
| 17 Cai D H, Wang J, Zhong A, et al. Relationship between Acute Respiratory Tract Infection and the Serum 25 (OH) D3 Level in Chronic Kidney Disease Patients and Its Prevention and Treatment. BioMed Research International, 2022: 2550686. | Non randomized controlled trials |
| 18 Ma L, Yu X, He R, et al. Experimental Analysis of the Effect of Rehabilitation Intervention on Tennis Players by Joint Injury Treatment. BioMed Research International, 2022: 4625719. | Non randomized controlled trials |
| 19 He Y, He J, Hou X. Influence of Continuous Nursing Intervention on Treatment Compliance of Patients with Depression. Evidence-Based Complementary and Alternative Medicine, 2022: 7080678. | Non randomized controlled trials |
| 20 Wu Z, Zhu Y. Comparison of the Effects of Epidural Anesthesia and General Anesthesia on Perioperative Cognitive Function and Deep Vein Thrombosis in Patients Undergoing Total Knee Arthroplasty. Evidence-Based Complementary and Alternative Medicine, 2021: 1565067. | Non randomized controlled trials |
| 21 Yang L, Han F. A Rehabilitation Model Conducive to Postoperative Recovery of Endometrial Cancer Patients after Laparoscopy. BioMed Research International, 2022: 9910841. | Non randomized controlled trials |
| 22 Liu N, Wang B. Comprehensive Rehabilitation Therapy Plus Glucosamine Hydrochloride for Exercise-Induced Knee Injuries and the Effect on Knee Function of Patients. Evidence-Based Complementary and Alternative Medicine, 2022: 8120458. | Non randomized controlled trials |
| 23 Xia Y, Wang J, Wang P. Systematic Nursing Interventions Combined with Continuity of Care in Patients with a Spinal Fracture Complicated with a Spinal Cord Injury and Its Effect on Recovery and Satisfaction. Evidence-Based Complementary and Alternative Medicine, 2022: 3771144. | Non randomized controlled trials |
| 24 Tan N, Xu L, Wu J. Analysis of the Causes and Preventive Strategies of Urogenic Sepsis after Flexible Ureteroscopic Lithotripsy. Evidence-Based Complementary and Alternative Medicine, 2022: 5332101. | Non randomized controlled trials |
| 25 Tang Z, Tang Y, Liu T, et al. Clinical Study on the Efficacy of Laparoscopic Hepatectomy and Its Influence on the Expression of Serum VEGF, FGF, and Immune Function. Evidence-Based Complementary and Alternative Medicine, 2021: 4432022. | Wrong study design |
| 26 Yue H, Fan K, Zhang Z, et al. Clinical Analysis of Video-Assisted Thoracoscopic Surgery for Resection of Solitary Pulmonary Nodules and Influencing Factors in the Diagnosis of Benign and Malignant Nodules. Evidence-Based Complementary and Alternative Medicine, 2021: 1490709. | Wrong study design |

| | |
|---|---|
| 27 Li J, Zhang X, Xie S, et al. Analysis of the Influence of High-Dose rhGH Therapy on Serum Vitamin D and IGF-1 Levels in School-Age Children with Idiopathic Short Stature. Evidence-Based Complementary and Alternative Medicine, 2021: 5776487. | Wrong study design |
| 28 Hao W, Zhu C, Chen Y, et al. Clinical Effect of Standardized Dietary Avoidance Therapy on Children with Milk Protein Allergy and Its Effect on Intestinal Flora. Evidence-Based Complementary and Alternative Medicine, 2022: 3362374. | Non randomized controlled trials |
| 29 Huang X, Zheng X, Shen C. Antiviral Therapy with Entecavir following Antituberculosis Therapy Alleviates Liver Injury and Restores Innate Immunity in Tuberculosis Patients Coinfected with Hepatitis B Virus. Evidence-Based Complementary and Alternative Medicine, 2021: 2884151. | Non randomized controlled trials |
| 30 Jin S, Wang L, Zhou S. Clinical Study on Different Methods of Internal Fixation for Treatment of Lisfranc Joint Injury. Evidence-Based Complementary and Alternative Medicine, 2021: 1300920. | Non randomized controlled trials |
| 31 Li C, Song W, Lei Y, et al. Clinical Application of Artificial Dermis and Autologous Skin in Repairing Skin and Soft Tissue Defects of Hands and Feet with Bone Exposure Injuries. Evidence-Based Complementary and Alternative Medicine, 2021: 1202826. | Non randomized controlled trials |
| 32 Wang X, Wu C. Study on the Effects of Optimized Emergency Nursing Combined with Mild Hypothermia Nursing on Neurological Prognosis, Hemodynamics, and Cytokines in Patients with Cardiac Arrest. Evid Based Complement Alternat Med. 2022:1787312. | Non randomized controlled trials |
| 33 Liu Y, Meng R, Dong J. Effect of Chronic Heart Failure Complicated with Type 2 Diabetes Mellitus on Cognitive Function in the Elderly. Evidence-Based Complementary and Alternative Medicine, 2022: 4841205. | Wrong study design |
| 34 Yun X Y, Chen S J, Zheng Q W. Targeted Perioperative Nursing Combined with Propofol and Fentanyl for Gynecological Laparoscopic Surgery. Evidence-Based Complementary and Alternative Medicine, 2022: 1257260. | Wrong study design |
| 35 Zhang H, Yang Q, Liu T, et al. Curative Effect Observation and Prognosis Analysis of Video-Assisted Thoracic Surgery and Thoracotomy in Patients with Hemopneumothorax. Evidence-Based Complementary and Alternative Medicine, 2021: 3937420. | Wrong study design |
| 36 Tang B, Wang X, Luo Y, et al. Efficacy and Safety of Intravitreal Injection of Triamcinolone Acetonide and Conbercept for Intraocular Lens after Cataract Surgery. Evidence-Based Complementary and Alternative Medicine, 2022: 5606343. | Wrong study design |
| 37 Geng Y, Liu L. Impact of Allogeneic Leukocyte-Depleted Red Blood Cell Transfusion on Inflammatory Response and Blood Coagulation in Patients with Recurrence of Colon Cancer after Operation. Evidence-Based Complementary and Alternative Medicine, 2021: 6957569. | Non randomized controlled trials |
| 38 Wang W, Yang Q, Xun Q,et al. Study on the Management of the Health Status of Patients with Stable Chronic Obstructive Pulmonary Disease Treated with Double Bronchodilator. Evid Based Complement Alternat Med.2022:1183436. | Wrong study design |
| 39 Tan J, Hu F, Ou J, et al. Analysis of the Curative Effect and Prognostic Factors of Anterior Cervical Surgery for Spinal Cord Injury without Radiographic Abnormalities. Evidence-Based Complementary and Alternative Medicine, 2022: 6836966. | Non randomized controlled trials |

| | |
|---|---|
| 40 Xu H, Yang W, Liu Y, et al. Analysis of Nursing Effect and Impact of Narrative Nursing Model on Anxiety of Tumor Patients with PICC under Chemotherapy. Evidence-Based Complementary and Alternative Medicine, 2021: 3698845. | Non randomized controlled trials |
| 41 Liu L, Guo Q, Shen W. Clinical Effectiveness of Laparoscopic Fiberoptic Choledochoscopy versus Conventional Open Surgery for Gallbladder Stones Complicated with Common Bile Duct Stones. Evid Based Complement Alternat Med. 2022:5668482. | Non randomized controlled trials |
| 42 Yang J, Guo Y, Dai Y. Impact of Kangaroo Mother Care Intervention on Immunological and Pulmonary Functions of Preterm Infants during Breastfeeding. Evidence-Based Complementary and Alternative Medicine, 2022: 3180871. | Non randomized controlled trials |
| 43 Xue L M, Yuan X, Zhang S, et al. Investigating the Effects of Dapagliflozin on Cardiac Function, Inflammatory Response, and Cardiovascular Outcome in Patients with STEMI Complicated with T2DM after PCI. Evidence-Based Complementary and Alternative Medicine, 2021: 9388562. | Wrong study design |
| 44 Sun M, Zhou R, Wang X, et al. Influence of Different Antiepileptic Drugs on Blood Ammonia and Homocysteine Levels in Children with Epilepsy. Evidence-Based Complementary and Alternative Medicine, 2021: 5698765. | Wrong study design |
| 45 Li C, Ma X, Yang Y,et al. Thoracoscopic Lobectomy versus Segmentectomy in the Treatment of Patients with Early-Stage Lung Cancer. Evid Based Complement Alternat Med.2022:4376968. | Wrong study design |
| 46 Huang W, Zhu W, Lu W. Comparison of Iliac Bone Transplantation with Bone Transport in the Treatment of Femur Fracture and Bone Defect. Evidence-Based Complementary and Alternative Medicine, 2022: 5358923. | Wrong study design |
| 47 Xu H, Li J, Zhong G, et al. Characteristics of the Dynamic Electrocardiogram in the Elderly with Nonvalvular Atrial Fibrillation Combined with Long R-R Intervals. Evidence-Based Complementary and Alternative Medicine, 2021: 4485618. | Non randomized controlled trials |
| 48 Yang W, Yang X, Zhang S,et al. Correlation of Serum Chemokine (C-C Motif) Ligand 21 and Heat Shock Protein 90 with Preeclampsia. Evid Based Complement Alternat Med.2022:2156424. | Wrong study design |
| 49 Zhou Y. Effects of High Flux Hemodialysis Combined with L-Carnitine on Microinflammation and Arteriovenous Fistula in Maintenance Hemodialysis Patients. Evidence-Based Complementary and Alternative Medicine, 2022: 6964127. | Non randomized controlled trials |
| 50 Zhao L, Qi J, Luo F, et al. The Diagnostic Value of Combined Detection of Serum Lp-PLA2 and Hcy and Color Doppler in Elderly Patients with Acute Coronary Syndrome and Effect on Endothelial Function. Evidence-Based Complementary and Alternative Medicine, 2022: 3150670. | Wrong study design |
| 51 Wan H, Tang D. Application Value of Nursing Intervention under the Guidance of Risk Prevention Management Concept in Preventing Vascular Access Infection in Patients Undergoing Maintenance Hemodialysis. Evidence-Based Complementary and Alternative Medicine, 2022: 9676074. | Non randomized controlled trials |
| 52 Wang Y, Wang J, Chen X. Clinical Efficacy of Xueshuantong plus Urokinase in the Treatment of Sudden Deafness. Evidence-Based Complementary and Alternative Medicine, 2022: 7775556. | Non randomized controlled trials |
| 53 Zheng L, Zhu Z. Growth and Development in Preterm Infants and Maternal Parenting Stress after WeChat-Based Extended Care. Evidence-Based Complementary and Alternative Medicine, 2022: 9987891. | Wrong study design |

| | |
|---|---|
| 54 Huang B B, Niu S K. The Effectiveness and Safety of Ropivacaine and Medium-Dose Dexmedetomidine in Cesarean Section. Evidence-Based Complementary and Alternative Medicine, 2022: 4447484. | Non randomized controlled trials |
| 55 Liu B, Shi T, Tian S, et al. Efficacy and Mechanism of Roxadustat plus Oral Iron in the Treatment of Elderly Chronic Kidney Disease with Anemia. Evidence-Based Complementary and Alternative Medicine, 2022: 9192655. | Non randomized controlled trials |
| 56 Gu J, Liang Y. Clinical Nursing Paths Benefit Patient Outcomes Undergoing Transcatheter Arterial Chemoembolization for Hepatocellular Carcinoma. Evidence-Based Complementary and Alternative Medicine, 2022: 4655293. | Wrong study design |
| 57 Wu L, Sun Y, Ni G, et al. Edaravone Combined with Clopidogrel Is Beneficial to Improve Efficacy, Neurological Impairment, and Life Function in Acute Cerebral Infarction Patients. Evidence-Based Complementary and Alternative Medicine, 2021: 8030521. | Non randomized controlled trials |
| 58 Li G, Liu J. Analysis of Efficacy, Safety, and Prognostic Factors of mFOLFOX6 Regimen Combined with Cetuximab and Simvastatin in the Treatment of K-RAS Mutant Colorectal Cancer. Evidence-Based Complementary and Alternative Medicine, 2021: 2280440. | Non randomized controlled trials |
| 59 Xu W, Tang J, Chen J. Influence of Diagnostic Informing on Negative Emotions, Illness Perception, Self-Perceived Burden, and Posttraumatic Stress Disorder in Patients with Gastrointestinal Tumors. Evidence-Based Complementary and Alternative Medicine, 2021: 2568195. | Non randomized controlled trials |
| 60 Zhao C, Huang M, Wang B, et al. Influence of Dexmedetomidine on Diaphragm Function and Postoperative Outcomes in ICU Patients with Mechanical Ventilation. Evidence-Based Complementary and Alternative Medicine, 2021: 1990838. | Non randomized controlled trials |
| 61 Zhang M, Gao L, Liu X, et al. Low-Dose Apatinib Improves the Prognosis of Patients with Recurrent High-Grade Gliomas. Evidence-Based Complementary and Alternative Medicine, 2022: 3181133. | Wrong study design |
| 62 Pan P, Chen L, Zhang D, et al. Continuing Care Bundle in Elderly Patients with Rectal Cancer after Radical Resection with Permanent Stoma. Evidence-Based Complementary and Alternative Medicine, 2022: 4065886. | Non randomized controlled trials |
| 63 Chang R, Wu J, Zhang X, et al. Analysis of the Reactivity of Aspirin and Clopidogrel and Its Influencing Factors in Patients with Coronary Heart Disease at High Altitude. Evidence-Based Complementary and Alternative Medicine, 2021: 2849982. | Non randomized controlled trials |
| 64 Ma J, Zhu S, Chen Z, et al. Clinical Significance of Detection of Peripheral Blood VASP Level in Lung Cancer Patients. Evidence-Based Complementary and Alternative Medicine, 2022: 1703339. | Wrong study design |
| 65 Wang Y, Zhou Y. Observation on the Effect of Rehabilitative Physical Training on Ice and Snow Sports Injury under Ultrasound Examination. Scanning, 2022: 2931686. | Wrong study design |
| 66 Wenjie E, Yu Q. Effect of Rehabilitation Physical Training on Basketball Injury under Ultrasound Examination. Scanning,2022. | Non randomized controlled trials |
| 67 Yin Y, Wei Z. Effect of Nursing Intervention on Coronary CT Angiography in Elderly Patients. Scanning, 2022: 3663285. | Non randomized controlled trials |

| | |
|---|---|
| 68 Liu X, Tang Z, Wang B, et al. Clinical Observation of MRI Image in Floating Needle Therapy for Cervical Spondylosis of Cervical Type. Scanning, 2022: 1340192. | Wrong study design |
| 69 Qian T. Evaluation of the Effect of Refined Nursing Intervention on Coronary CT Imaging Microscopy. Scanning, 2022: 4870548. | Non randomized controlled trials |
| 70 Xu X, Guo D, Zhang Y, et al. Effect of Microscope Combined with Wechat Smart Platform on Clinical Efficacy and Gastrointestinal Function of Patients with Cholecystolithiasis Combined with Common Bile Duct Stones. Scanning, 2022: 9661506. | Wrong study design |
| 71 Lin S, Zang M. Effectiveness of Mayinglong Musk Hemorrhoid Ointment on Wound Healing and Complications after Internal Hemorrhoid Ligation and External Hemorrhoidectomy. Evidence-Based Complementary and Alternative Medicine, 2022: 5630487. | Wrong study design |
| 72 Gong X, Qian Y, Zhang L, et al. Effect of Uterine Arterial Chemoembolization Combined with Ultrasound-Guided Uterine Curettage on Cervical Pregnancy and Influencing Factors. Evidence-Based Complementary and Alternative Medicine, 2021: 4609497. | Non randomized controlled trials |
| 73 Ma T, Wang Y, Liu J, et al. Clinical Study of Influence of Continuous Nursing Intervention Combined with Comfort Nursing Intervention under Medical-Nursing Combination on Self-Care Ability and Satisfaction of Elderly Patients with Chronic Diseases. Evidence-Based Complementary and Alternative Medicine, 2021: 1464707. | Non randomized controlled trials |
| 74 Han Y. A Virtual Reality Algorithm for the Study of Clinical Efficacy of Sports Injury Rehabilitation Training. Journal of healthcare engineering, 2021: 6725625. | Non randomized controlled trials |
| 75 Lv H, Zhao X, Yu J. Analysis of the Clinical Effects of Sodium Valproate and Levetiracetam in the Treatment of Women with Epilepsy during Pregnancy. Evidence-Based Complementary and Alternative Medicine, 2021: 5962200. | Non randomized controlled trials |
| 76 Demir M, Akın M, Yücel N, et al. Role of Radiology and Laparoscopy in Childhood Peptic Ulcer Perforation. Journal of Environmental and Public Health, 2022: 1211499. | Non randomized controlled trials |
| 77 Ma T, Li G, Zhang H,et al. Epidural Anesthesia versus General Anesthesia for Total Knee Arthroplasty: Influences on Perioperative Cognitive Function and Deep Vein Thrombosis. Comput Math Methods Med.2022:4259499. | Wrong study design |
| 78 Zhu J, Wang S, Chen Z, et al. Efficacy of Rosuvastatin Combined with rt-PA Intravenous Thrombolytic Therapy for Elderly Acute Ischemic Stroke Patients. Computational and Mathematical Methods in Medicine, 2022: 9403693. | Non randomized controlled trials |
| 79 Jiang Y, Song B, Chen Z. Efficacy and Side Effects of Irinotecan Combined with Nedaplatin versus Paclitaxel Combined with Cisplatin in Neoadjuvant Chemotherapy for Locally Advanced Cervical Cancer and Tumor Marker Analysis: Based on a Retrospective Analysis. Comput Math Methods Med.2022:5936773. | Non randomized controlled trials |
| 80 Ge J, Jiao X, Qi F, et al. Neural Function Recovery and Safety of Mild Hypothermia Therapy Combined with Monosialotetrahexosylganglioside on Neonatal Asphyxia Complicated by Hypoxic Ischemic Encephalopathy. Computational and Mathematical Methods in Medicine, 2021: 6186011. | Wrong study design |

| | |
|---|---|
| 81 Chen X, Qiu X, Jin Y. Effects of Dydrogesterone Tablets Combined with Zishen Yutai Pills on Threatened Abortion in Early Pregnancy and Pregnancy Outcomes. Computational and Mathematical Methods in Medicine, 2022: 4593637. | Wrong study design |
| 82 Xu H, Fan Y, Lu J, et al. Analysis of Efficacy, Complications, and Inflammatory Reactions of Bridge Combined Internal Fixation System for Periarticular Fractures of the Shoulder. Computational and Mathematical Methods in Medicine, 2022: 5048172. | Wrong study design |
| 83 Yu H, Dong H, Ruan B, et al. Clinical Effect of Suture Anchor and Double-Pulley Technique in the Treatment of Inferior Patellar Fracture. Comput Math Methods Med.2021:4964195. | Non randomized controlled trials |
| 84 Li Y, Liu S, He Z, et al. Comparison of Long-Term Efficacy of MIS-TLIF Intraoperative Implants in Patients with Osteoporosis. Computational and Mathematical Methods in Medicine, 2022: 2565391. | Non randomized controlled trials |
| 85 Cong Y, Deng H, Lei J, et al. Comparison of the Effects of Intramedullary Nailing and Plate Fixation on Lower-Extremity Deep Vein Thrombosis after Tibial Fractures. Computational and Mathematical Methods in Medicine, 2022: 4852201. | Non randomized controlled trials |
| 86 Du X, Yu L, Wu X, et al. Ultrasonography-Guided Combination with Elbow Arthrography-Assisted Minimally Invasive Treatment of Radial Neck Fractures in Young Children. Computational and Mathematical Methods in Medicine, 2022: 6840716. | Non randomized controlled trials |
| 87 Luo H, Zhu G. Clinical Application Effect of Cluster Management in Noninvasive Ventilator Nursing Care of Patients with Severe Heart Failure. Computational and Mathematical Methods in Medicine, 2022: 9628213. | Non randomized controlled trials |
| 88 Li D, Zhang W, Wei X. Effect of Massive Transfusion Protocol on Coagulation Function in Elderly Patients with Multiple Injuries. Comput Math Methods Med.2021:2204542. | Wrong study design |
| 89 Chu L, Wu Y, Yang L, et al. Abnormal levels of cortisol and cortisone in patients with prurigo nodularis. Indian Journal of Dermatology, 2021, 66(6): 685-687. | Wrong study design |
| 90 Zhang G, Chen S, Zhang D, et al. Long-Term Outcomes of Endoscopic Intervention in the Treatment of Symptomatic Pancreas Divisum. Journal of Healthcare Engineering, 2022: 8508943. | Non randomized controlled trials |
| 91 Lan W, Xie F, Que W, et al. Efficacy of Intramedullary Nailing in the Treatment of Comminuted Proximal Humeral Fractures and Its Influence on Shoulder Joint Function Recovery. Journal of Healthcare Engineering, 2022: 7272385. | Non randomized controlled trials |
| 92 Gu Y, Liu X, Gu T, et al. Effect of Different Doses of Propofol on Pulmonary Function and Inflammatory Response in Patients with Lung Ischemia Reperfusion Injury Induced by One-Lung Ventilation Based on Big Data Analysis. Journal of Healthcare Engineering, 2022: 7677266. | Wrong study design |
| 93 Zhang W, Yao T, Dong G. Application of Sandwich Teaching Method Based on Network Platform in Rehabilitation Nursing Teaching in Postepidemic Era. BioMed Research International, 2022: 7489023. | Non randomized controlled trials |
| 94 Liu Q, Wang J, Han J, et al. Effects of Seamless Operating Room Nursing Combined with Multistyle Health Education on the Psychological State, Rehabilitation Quality, and Nursing Satisfaction in Patients with Internal Fixation of Femoral Fracture. Journal of healthcare engineering, 2022: 5196363. | Wrong study design |

| | |
|---|---|
| 95 Kong G, Liu J, Jiang J. Effect of Comprehensive Nursing Intervention Under Internet-Based WeChat Platform Education on Postoperative Recovery of Puerperae Undergoing Cesarean Section. Journal of Healthcare Engineering, 2022: 5040461. | Non randomized controlled trials |
| 96 Liu Z, Liu L, Zhang H, et al. Preventive Effect Observation of Dapagliflozin on Middle and Later Ventricular Remodeling in Patients with Acute ST Segment Elevation Anterior Wall Myocardial Infarction: A Single-Center, Retrospective Cohort Study. Journal of Healthcare Engineering, 2022: 3955914. | Non randomized controlled trials |
| 97 Wang X J, He N N, Ji W B, et al. Effect of Penetration Electroacupuncture Combined with Intermediate Frequency Electrotherapy, Facial Acupoint Massage, and Cervical Reduction on Facial Nerve Function and Curative Effect of Senile Refractory Facial Paralysis. Journal of Healthcare Engineering, 2021: 3776006. | Non randomized controlled trials |
| 98 Jiang W, Zhang Y, Huang Y, et al. Effects of Different Nonsteroidal Anti-Inflammatory Drugs Combined with Platelet-Rich Plasma on Inflammatory Factor Levels in Patients with Osteoarthritis. Journal of Healthcare Engineering, 2022: 1979892. | Non randomized controlled trials |
| 99 Du W, Liu Z, Wang D. Clinical Efficacy of LSC and TVT-O for Stress Urinary Incontinence Complicated with Pelvic Organ Prolapse and Factors Influencing Postoperative Urinary Function Recovery. Journal of Healthcare Engineering, 2022: 1557256. | Non randomized controlled trials |
| 100 Li J, Ma J, Liu S, et al. The Effects of Rso2 and PI Monitoring Images on the Treatment of Premature Infants Based on Deep Learning. Computational and Mathematical Methods in Medicine, 2022: 5671713. | Non randomized controlled trials |
| 101 Wu C, Duan Z, Shu J, et al.Cardiac Magnetic Resonance Assessment of the Protective Effect of Remote Ischemic Postconditioning on Coronary Microcirculation after Reperfusion Therapy for Acute ST-Segment Elevation Myocardial Infarction. Computational and Mathematical Methods in Medicine, 2022: 5629763. | Wrong study design |
| 102 Zhang W, Zhang B, Zhang H, et al.Analysis of the Influence of Network Continuous Care on the Quality of Life of Patients with Coronary Artery Disease (CAD) after PIC. BioMed Research International, 2022: 3046554. | Wrong study design |
| 103 Jing W, Dai Y, Zhu J, et al.Clinical Efficacy and Safety Evaluation of Calcitriol Combined with Bisphosphonates in the Therapy of Postmenopausal Osteoporosis: Based on a Retrospective Cohort Study. BioMed Research International, 2022: 2711938. | Wrong study design |
| 104 Dummer R, Queirolo P, Duhard P G, et al. Atezolizumab, vemurafenib, and cobimetinib in patients with melanoma with CNS metastases (TRICOTEL): a multicentre, open-label, single-arm, phase 2 study. The Lancet Oncology, 2023, 24(12): e461-e471. | Wrong study design |
| 105 Zheng J, Xue Y, Li C.Short-Term Efficacy and Quality of Life of Gastric Cancer Patients Undergoing Radical Gastrectomy Assisted by External Vision. Computational and Mathematical Methods in Medicine, 2021: 4256347. | Non randomized controlled trials |
| 106 Zhang T, Zhang L.Focus on the Analysis of the Effect of Solving the Nursing Mode on the Time of the Production Time of the First Maternity and the Subjective Happiness of the Postpartum. Contrast Media & Molecular Imaging, 2022: 2952949. | Wrong study design |
| 107 Zhang Y, Liu P, Su W, et al.Fibroblast Growth Factor 3 Is Associated with Tongue Squamous Cell Carcinoma: A Controlled Study. Computational and Mathematical Methods in Medicine, 2022: 3331119. | Wrong study design |

| | |
|---|---|
| 108 Li S, Luo J, Wang Z, et al.Efficacy and Prognosis of Ultrasound-Guided Percutaneous Catheter Drainage in Patients with Liver Abscess Complicated with Septic Shock. Computational and Mathematical Methods in Medicine, 2022: 4688356. | Wrong study design |
| 109 Zhang B, Chen C.Comparison of Ventilator-Associated Pneumonia and Surgical Site Infection between Two Methods of Tracheostomy. Computational and Mathematical Methods in Medicine, 2022: 3186634. | Wrong study design |
| 110 Xia Y, Chen Y, Xia R, et al.Nursing Process Design of Intravenous Thrombolysis in Elderly Patients with Acute Cerebral Infarction. Computational Intelligence and Neuroscience, 2022: 2047948. | Wrong study design |
| 111 Men B, Jiang S, Li Y.Investigation of the Effect of Oral Implant Surgery on Clinical Treatment and Oral Function of Patients with Dentition Loss. Contrast Media & Molecular Imaging, 2022: 1698842. | Wrong study design |
| 112 Cong S, Dong C, Hu Y, et al.Effect of Salvia Miltiorrhiza Polyphenolic Acid Injection on Improving Limb Use and Cognitive Impairment in Patients with Acute Stroke. Computational and Mathematical Methods in Medicine, 2022: 1481294. | Wrong study design |
| 113 Wu J, Mao B, Jin T, et al.Comparative Study on the Clinical Effects of Different Surgical Methods in the Treatment of Gastrointestinal Stromal Tumors. Evidence-Based Complementary and Alternative Medicine, 2022: 1280756. | Non randomized controlled trials |
| 114 Zhang J, Xing Z.Application Effect Analysis of a Nanotube Combined with Orthopedic Exercise Rehabilitation Therapy in the Treatment of Patients with Knee Arthritis. Journal of Nanomaterials, 2022: 1275250. | Wrong study design |
| 115 Zhang Q, Zhou S, Yin H, et al.Analysis of Immunotherapy Combined with Radiotherapy in Patients with Brain Metastasis of Driver Gene-Negative Non-Small-Cell Lung Cancer. Evidence-Based Complementary and Alternative Medicine, 2022: 1193075. | Non randomized controlled trials |
| 116 Xiang M, Cao F, Peng J, et al.Application of Nanoscaffold Material Combined with Exercise Rehabilitation Therapy in the Treatment of Athletes with Hip Injuries. Journal of Nanomaterials, 2022: 6582511. | Wrong study design |
| 117 Yu S, Lu C, Qin L.A Retrospective Study of Diaphragmatic Breathing Training Combined with Discharge Care Bundles in Patients with Chronic Obstructive Pulmonary Disease. Evidence-Based Complementary and Alternative Medicine, 2022: 9649986. | Wrong study design |
| 118 Lu S X, Xu J, Lu H Y, et al.Balloon Eustachian Tuboplasty and Grommet Insertion: A Combined Surgical Treatment for Chronic Suppurative Otitis Media with Eustachian Tube Dysfunction. Evidence-Based Complementary and Alternative Medicine, 2022: 9516029. | Non randomized controlled trials |
| 119 Cai X, Li J, Li W.Application of PDCA Circulation Regulation Combined with Nursing Mark in Nursing Safety and Quality Regulation of Disinfection Supply Center. Evidence-Based Complementary and Alternative Medicine, 2022: 9253777. | Non randomized controlled trials |
| 120 Li C, Guo Z.Outcome of Percutaneous Transforaminal Endoscopic Lumbar Decompression for Multisegment Lumbar Spinal Stenosis and the Effect on VAS Scores. Evidence-Based Complementary and Alternative Medicine, 2022: 9040402. | Wrong study design |
| 121 Zhao Y, Liang J, Ou H, et al.Effects of Swallowing Rehabilitation Training with a Balloon Dilation Therapy on the Deglutition Function and Quality of Life of Patients with Dysphagia after Radiotherapy for Nasopharyngeal Carcinoma. Evidence-Based Complementary and Alternative Medicine, 2022: 7496753. | Wrong study design |

| | |
|---|---|
| 122 Wang P, Jin X, Zhang Y, et al.Effect of Vitamin D Combined with Recombinant Human Growth Hormone in Children with Growth Hormone Deficiency. Disease Markers, 2022: 7461958. | Non randomized controlled trials |
| 123 Yu J, Xu D.Efficacy and Safety of Peginterferon α-2a and Entecavir Tenofovir in the Treatment of Chronic Hepatitis B Genotype C. Contrast Media & Molecular Imaging, 2022: 7623832. | Non randomized controlled trials |
| 124 Tian N, Wang D, Li X, et al.Effect of Paclitaxel Combined with Doxorubicin Hydrochloride Liposome Injection in the Treatment of Osteosarcoma and MRI Changes before and after Treatment. Evidence-Based Complementary and Alternative Medicine, 2022: 5651793. | Wrong study design |
| 125 Peng T, Lou Z, Wang X, et al.Clinical Comparison of Endoscopic Ultrasonography and CT in Preoperative TN Staging of Esophagogastric Junction Cancer. Contrast Media & Molecular Imaging, 2022: 5810405. | Non randomized controlled trials |
| 126 Wang H, Zhou D, Sun Z, et al.Effects of Calf Blood-Deproteinized Extract Ophthalmic Gel Combined with Sodium Hyaluronate Eye Drops on Conjunctival Hyperemia Score and Tear Film Stability in Patients with Dry Eye. Computational Intelligence and Neuroscience, 2022: 6732914. | Non randomized controlled trials |
| 127 Wu S F, Fang J, Yu F, et al.Clinical Study of Intravitreal Injection of Anti-VEGF Drugs Combined with Triamcinolone Acetonide in the Treatment of Coats Disease. Computational and Mathematical Methods in Medicine, 2022: 9911549. | Wrong study design |
| 128 Mei Y.Application of Clinical Case Teaching Mode in Gynecological Nursing Teaching. Computational and Mathematical Methods in Medicine, 2022: 9739313. | Non randomized controlled trials |
| 129 Zhao Z, Li J, Yu X, et al.Effect of Mosaic Allograft Osteochondral Transplantation Combined with Corrective Osteotomy in Treating Osteochondral Lesions of the Talus on Ankle and Knee Joint Function and Lower Limb Alignment. Computational and Mathematical Methods in Medicine, 2022: 9688098. | Wrong study design |
| 130 Shang P P, Yu Y, Yan P N, et al.Therapeutic Effect and Prognosis of Biliary Tract Tumor Transformation. Computational and Mathematical Methods in Medicine, 2022: 9489003. | Wrong study design |
| 131 Li W, Li G, Zhou W, et al.Effect of Autoimmune Cell Therapy on Immune Cell Content in Patients with COPD: A Randomized Controlled Trial. Computational and Mathematical Methods in Medicine, 2022: 8361665. | Non randomized controlled trials |
| 132 Ma H, Cao J, Li M. Application of PDCA Process Management in Day Operation Ward and the Influence of Nursing Quality and Safety. Comput Math Methods Med.2022:8169963. | Wrong study design |
| 133 Chen S, Wang Y, Huang S, et al.Effects of Silicone Mattress Combined with Hydrocolloid Dressing on Pressure Ulcers and Phlebitis in ICU Patients with Liver Failure. Computational and Mathematical Methods in Medicine, 2022: 7828650. | Wrong study design |
| 134 Yu X, Chen L, Chen S, et al.Application of Care Bundles in Postanesthesia Recovery for Elderly Patients with Colorectal Cancer. Computational and Mathematical Methods in Medicine, 2022: 7669889. | Wrong study design |
| 135 Hu R, Zhang F, Guo X, et al.Effect of Vitapex Combined with AH-Plus Paste on Inflammation in Middle-Aged and Elderly Patients with Periodontal-Endodontic Disease. Computational and Mathematical Methods in Medicine, 2022: 7540961. | Wrong study design |

| | |
|---|---|
| 136 Zhang J, Han Z, Jia M, et al.Application Effect of Doctor-Nurse-Patient Integration Model Based on Heart Rate Management Strategies in Middle-Aged and Young Outpatients with Hypertension. Computational and Mathematical Methods in Medicine, 2022: 7459518. | Non randomized controlled trials |
| 137 Jiang Q, Zhao G, Song S, et al.Analyzing the Treatment of Patients with Acute Exacerbation of COPD with the Aid of Intelligent Diagnosis Method. Journal of Healthcare Engineering, 2022: 3962074. | Wrong study design |
| 138 Zhang M, Lai W, Zhang J, et al.Efficacy Investigation of TACE Combined with Lenvatinib and Sintilimab in Intermediate-Stage Hepatocellular Carcinoma. Disease Markers, 2022: 6957580. | Non randomized controlled trials |
| 139 Chen J, Zheng Y, Zhang W, et al. Efficacy and Safety of Amniotic Membrane Transplantation Combined with Closure of Tenon Capsule and Bulbar Conjunctival Space in the Treatment of Primary Pterygium. Contrast Media Mol Imaging.2022:5844973. | Wrong study design |
| 140 Han T, Deng Y, Quan D, et al.WeChat-Based Comprehensive Education on Egg White Protein Intake for Patients Undergoing Peritoneal Dialysis: A Combined Prospective and Retrospective Study. Contrast Media & Molecular Imaging, 2022: 5651992. | Non randomized controlled trials |
| 141 Ding X, Pan Z, Ma Z, et al.Clinical Application of Evoked Potentials in the Operation of Cervical Spondylotic Myelopathy with Different Imaging. Contrast Media & Molecular Imaging, 2022: 4154278. | Non randomized controlled trials |
| 142 Xi C, Jiang H, Xue Y, et al.Effects of Bevacizumab Combined with Chemotherapy on CT, CyFRA21-1, and ProGRP and Prognosis of Lung Cancer Patients under Nursing Intervention. Computational and Mathematical Methods in Medicine, 2022: 9422902. | Non randomized controlled trials |
| 143 Lin L, Yang S, Bai Q, et al.TCM Treatment and Drug Co-Occurrence Analysis of Psoriasis. Computational Intelligence and Neuroscience, 2022: 4268681. | Non randomized controlled trials |
| 144 Zhang L, Tao Z, Wang X.Comparison of Short-Term Restorative Effects and Periodontal Health Status of Restorations Made of Different Materials in Full-Crown Restoration of Mandibular Premolar Tooth Defects. Disease Markers, 2022: 3682741. | Non randomized controlled trials |
| 145 Yuan Y, Pan S, Zou C, et al.Analysis of the Relationship between Scleritis and T Cell Activation in Patients with Hepatocellular Carcinoma Treated with PD-1 Carrelizumab. Contrast Media & Molecular Imaging, 2022: 4853481. | Non randomized controlled trials |
| 146 Bian L, Li J, Li W, et al.Analysis of the Effect of Holistic Nursing in the Operating Room Based on PDCA and Evidence-Based Nursing in the Otorhinolaryngology Operating Room: Based on a Retrospective Case-Control Study. Contrast Media & Molecular Imaging, 2022: 4514669. | Non randomized controlled trials |
| 147 Handa A, Bhullar K K, Batra D, et al. Clinical performance of various bonding agents in noncarious cervical defects. Journal of Conservative Dentistry and Endodontics, 2023, 26(3): 271-274. | Wrong study design |
| 148 Lin G S, Wang W W, Lin H, et al.Bevacizumab Combined with Intensity-Modulated Radiation Therapy on Cognitive and Coagulation Function in Postoperative Glioma Patients. Journal of Healthcare Engineering, 2022: 9367919. | Non randomized controlled trials |

| | |
|---|---|
| 149 Li J, Huang Z, Li K, et al. Study on the Effect of Self-Made Lifei Dingchuan Decoction Combined with Western Medicine on Cough Variant Asthma. Comput Math Methods Med.2022:9803552. | Wrong study design |
| 150 Jia N, Gao W, Fan X, et al.Clinical Efficacy of PEG-IFNα-2a and PEG-IFNα-2b in the Treatment of Hepatitis B e Antigen-Positive Hepatitis B and Their Value in Improving Inflammatory Factors and Hemodynamics in Patients: A Comparative Study. Oxidative Medicine and Cellular Longevity, 2022: 3185320. | Non randomized controlled trials |
| 151 Gao H, You W, Lv J, Li Y. Hemodynamic Analysis of Pipeline Embolization Device Stent for Treatment of Giant Intracranial Aneurysm under Unsupervised Learning Algorithm. J Healthc Eng. 2022:8509195. | Non randomized controlled trials |
| 152 Qiu H, Du W. Evaluation of the Effect of PDCA in Hospital Health Management. J Healthc Eng. 2021:6778045. | Non randomized controlled trials |
| 153 Miao S, Li S, Wu Z, et al. The Clinical Efficacy and Risk Factors after Revision and Reconstruction of Anterior Cruciate Ligament. J Healthc Eng. 2021:6606492. | Wrong study design |
| 154 Yuan W, Li Y, Dai Y, et al. Efficacy of Super-Mini-PCNL and Ureteroscopy in Kidney Stone Sufferers and Risk Factors of Postoperative Infection. J Healthc Eng. 2022:4733329. | Wrong study design |
| 155 Tang H, Gao L, Li Y. Influence of the Clinical Nursing Pathway on Nursing Outcomes and Complications of Cervical Carcinoma Patients Undergoing Chemotherapy via PICC. Evid Based Complement Alternat Med. 2022:4040033. | Non randomized controlled trials |
| 156 Chen Y, Wang F, Cui L, et al. Short- and Long-Term Influences of Benzodiazepine and Z-Drug Use in Patients with Bipolar Disorder Combined Sleep Disturbance during Affective Period: A Nine-Month Follow-Up Analysis. Dis Markers.2022:6799898. | Wrong study design |
| 157 Wang R, Duan G, Wu Y, et al. Effectiveness of Chinese Native Culture Education for Improving Undergraduate Nursing Students' Transcultural Self-Efficacy. Dis Markers.2022:5813946. | Non randomized controlled trials |
| 158 He L, Yang Z, Xu J, Wang Q. Evaluation of the Effectiveness of a Combination of Chinese Herbal Fumigation Sitz-Bath and Red Ointment in Managing Postoperative Wound Healing and Pain Control in Anal Fistula Patients. Contrast Media Mol Imaging.2022:1905279. | Non randomized controlled trials |
| 159 Gong F, Li X, Zhang H,et al. Comparison of the Effects of Open Surgery and Minimally Invasive Surgery on the Achilles Tendon Rupture Healing Based on Angiogenesis. Comput Intell Neurosci.2022:1447129. | Wrong population |
| 160 Zhou Q, Han Y, Chen J. The Efficacy of Remifentanil Combined with Propofol in Craniotomy for Tumor Was Evaluated by Wake Quality, Hemodynamics, and Adverse Reactions. Biomed Res Int. 2022:4861043. | Non randomized controlled trials |
| 161 Wang X, Liu J, Zhou P, et al. Effects of Angelica Fritillaria Kushen Pill on Renal Function and Immune Function after Laparoscopic Radical Nephrectomy for Patients with Renal Carcinoma. J Healthc Eng. 2022:4082121. | Non randomized controlled trials |
| 162 Hu B, Wang H, Ma T, et al. Effect Analysis of Epidural Anesthesia with 0.4% Ropivacaine in Transforaminal Endoscopic Surgery. J Healthc Eng.2021:2929843. | Non randomized controlled trials |
| 163 Li J, He Q. Evaluation of Tresiba Combined with Six Ingredient Rehmannia Pill in the Treatment of Type 2 Diabetes. J Healthc Eng. 2022:2177176. | Wrong study design |

| | |
|---|---|
| 164 Li Q. The Effects of Yoga Exercise on Pelvic Floor Rehabilitation of Postpartum Women. J Healthc Eng. 2022:1924232. | Non randomized controlled trials |
| 165 Li M, Liu Y. The Impact of Cultural Creative Product Design for Sport Events on the Residents' Fitness. J Environ Public Health. 2022:3150099. | Non randomized controlled trials |
| 166 Wu Y, Gu Y, Rao X,et al. Clinical Effects of Outpatient Health Education on Fall Prevention and Self-health Management of Elderly Patients with Chronic Diseases. Evid Based Complement Alternat Med. 2022:6265388. | Wrong study design |
| 167 Shi Y. Effect of Atropine Eye Drops Combined with VR-Based Binocular Visual Function Balance Training for Prevention and Control of Juvenile Myopia. Evid Based Complement Alternat Med.2022:4159996. | Non randomized controlled trials |
| 168 Pang Z, Li Y, Huang A, et al. Effect of HCH Nutrition Management Combined with Early Exercise Nursing on Nutrition Status and Postoperative Rehabilitation of Patients after Gastric Cancer Surgery. Evid Based Complement Alternat Med.2022:2813050. | Wrong study design |
| 169 Shan Z. Electron Microscope Observation of Acupuncture and Nerve Repair in the Treatment of Peripheral Facial Paralysis. Emerg Med Int.2022:5432223. | Non randomized controlled trials |
| 170 Wang H, Lei F, Bai L, Zhang A. Effects of Amiodarone and Esmolol for Heart Rate and Cardiovascular Changes. Emerg Med Int. 2022:9197369. | Wrong study design |
| 171 Zhang J, Mu Y, Zhang Y. Effects of Acupuncture and Rehabilitation Training on Limb Movement and Living Ability of Patients with Hemiplegia after Stroke. Behav Neurol. 2022:2032093. | Non randomized controlled trials |
| 172 Lin Y, Wu D, Shen Y, He Y, Ye J. Comparison of Outcomes between Two Surgical Techniques for Patients with Intestinal Neuronal Dysplasia. J Immunol Res.2022:9165651. | Wrong study design |
| 173 Lei L. Observation on the Effect of Intelligent Machine-Assisted Surgery and Perioperative Nursing. J Healthc Eng.2022:6264441. | No full-text |
| 174 Tang Y, Liu T, Cai Q, Zhao M. The Effects of Febuxostat on Urine NGAL and Urine KIM-1 in Patients with Hyperuricemia. J Healthc Eng. 2022:6028611. | Wrong study design |
| 175 Tang Y, Liu T, Cai Q, Zhao M. The Effects of Febuxostat on Urine NGAL and Urine KIM-1 in Patients with Hyperuricemia. J Healthc Eng. 2022:6028611. | Wrong study design |
| 176 Hou Y, Liu B. Analysis of Systolic Blood Pressure Level and Short-Term Variability in Masked Hypertension. J Healthc Eng.2022:8016893. | Wrong study design |
| 177 Li J G, Wang X S, Wang L J, et al.Effects of Artificial Intelligence and Virtual Reality in Martial Arts Sports on Students' Physical and Mental Health. International Transactions on Electrical Energy Systems, 2022: 1359243. | Wrong study design |
| 178 Zhang H, Yin Y, Tao W, et al.Clinical Observation of MRI Scanning Combined with Clinical Nursing for Surgical Breast Cancer Patients. International Journal of Analytical Chemistry, 2022: 6863281. | Wrong study design |
| 179 Zhang S, Yang K, Wang B.Efficacy Evaluation of the VFQ-25 Scale in Patients with Different Degrees of Vitreous Opacity After Nd: YAG Laser Ablation. Evidence-Based Complementary and Alternative Medicine, 2022: 5075447. | Wrong study design |

| | |
|---|---|
| 180 Huang S, Xie Y, Huang Z, et al.Preoperative Predictors of Prolonged Hospital Stay in Accelerated Rehabilitation for Patients Undergoing Orthopedic Surgery. Bioinorganic Chemistry and Applications, 2021: 7832216. | Non randomized controlled trials |
| 181 Lv F, Wang H, Zhang D, et al.Clinical Characteristics, Treatment, and Visual Prognosis in Pediatric Endophthalmitis: A 232-Case Retrospective Study. Applied Bionics and Biomechanics, 2022: 8523747. | Non randomized controlled trials |
| 182 He R.The Intervention of Music Therapy on Behavioral Training of High-Functioning Autistic Children under Intelligent Health Monitoring. Applied bionics and biomechanics, 2022: 5766617. | Non randomized controlled trials |
| 183 Tang L, Xing Y, Li H.Construction of Nursing Practice Model in Case Management of Concurrent Chemotherapy and Radiochemotherapy Treatment in Cervical Cancer. Applied Bionics and Biomechanics, 2022: 5450575. | Wrong study design |
| 184 Chen Y, Huang X, Lu Q, et al.Clinical Study of Mobile Application-(App-) Based Family-Centered Care (FCC) Model Combined with Comprehensive Iron Removal Treatment in Children with Severe Beta Thalassemia. Applied Bionics and Biomechanics, 2022: 4658709. | Wrong study design |
| 185 Zhangyanhui, Cui H, Genglei.Combination of Traditional Chinese and Western Medicine in Sports in Pharmaceutical Health. Applied Bionics and Biomechanics, 2021: 1840915. | Wrong study design |
| 186 Morisky DE, Ang A, Krousel-Wood M, et al. Predictive validity of a medication adherence measure in an outpatient setting. J Clin Hypertens (Greenwich). 2008;10(5):348-54. | Non randomized controlled trials |
| 187 Chu L, Qian G.Cohort Study on the Effect of Psychological Education for Nurses in Psychiatric Department. Emergency Medicine International, 2022: 7394710. | Wrong study design |
| 188 Yu Q, Shan L, Du L, et al.Effects of Sports Drinks on Weight Loss Control and Lipid Metabolism in Overweight Students. Journal of Food Quality, 2022: 2183088. | Wrong study design |
| 189 Wenqin L.Analysis of the Effectiveness of Music Therapy on Mental Health of College Students. Journal of Healthcare Engineering, 2022: 7288788. | Wrong study design |
| 190 Zhang X.Clinical Observation of the Effect of Nasal Breathing on Nonanalgesic and Sedative Gastroscopy. Contrast Media & Molecular Imaging, 2022: 8603625. | Non randomized controlled trials |
| 191 Zhao Z, Lv D, Zhang B, et al.Efficacy of Human-Recombinant Epidermal Growth Factor Combined with Povidone-Iodine for Pressure Ulcers and Its Influence on Inflammatory Cytokines. Mediators of Inflammation, 2022: 3878320. | Wrong study design |
| 192 Li Y, Xiao F.Preventive Effect of Intensive Nursing Intervention of Deep Vein Thrombosis of Lower Extremities in Elderly Patients with Gastrointestinal Tumors after Surgery. Journal of Oncology, 2022: 2967981. | Non randomized controlled trials |
| 193 Qi F, Xiang M, Deng Y, et al.Application of Da Vinci Robot and Thoracoscopy in Radical Lung Cancer Surgery. Journal of Healthcare Engineering, 2022: 2011062. | Non randomized controlled trials |
| 194 Healthcare Engineering JO.Study on the Application and Efficacy of Responsibility Nursing in Dialysis Care. J Healthc Eng. 2023:9828701. | Wrong study design |
| 195 Abdellaoui S, Gregoire M, Dubert M,et al. Suboptimal dalbavancin dosages in an adult with sickle-cell disease and glomerular hyperfiltration. J Antimicrob Chemother. 2023;78(3):851-852. | Wrong study design |

| | |
|---|---|
| 196 De Vecchis R, Paccone A, Di Maio M. Sacubitril/valsartan improves left ventricular longitudinal deformation in heart failure patients with reduced ejection fraction. Minerva Cardioangiologica, 2019, 67(6): 456-463. | Wrong study design |
| 197 De Vecchis R, Soreca S, Ariano C. Ablation, rate or rhythm control strategies for patients with atrial fibrillation: how do they affect mid-term clinical outcomes?. Minerva Cardioangiologica, 2019, 67(4): 272-279. | Wrong study design |
| 198 Li N, Zhang J, Meng X, et al.Clinical Application Value of High-Frequency Ultrasound Combined with Detection of Serum High Mobility Group Box 1, Soluble IL-2 Receptor, and Thyroglobulin Antibody in Diagnosing Thyroid Cancer. Journal of Healthcare Engineering, 2022: 7851436. | Wrong study design |
| 199 Yang R, Wang Z, Jia Y, et al.Comparison of Clinical Efficacy of Sodium Nitroprusside and Urapidil in the Treatment of Acute Hypertensive Cerebral Hemorrhage. Journal of Healthcare Engineering, 2022: 2209070. | Wrong study design |
| 200 Zhu W, Mao S, Chen Y, et al.Analysis of Clinical Efficacy of Laparoendoscopic Single-Site Surgery for Uterine Fibroids. Journal of Healthcare Engineering, 2022: 5606998. | Wrong study design |
| 201 Cai F, Wang X, Deng Z, et al.Exploration of the Visual Function after the Implantation of Continuous Visual Range Human Cocrystal Micromonocular Vision in Both Eyes of the Patient. Journal of Healthcare Engineering, 2022: 6000977. | Non randomized controlled trials |
| 202 Fang X, Liao T, Chen J, et al.The Feasibility Mechanism of Nerve Interventional Thrombectomy for Occlusion of Cranial Artery M1 and M2 Segments. Computational and Mathematical Methods in Medicine, 2022: 6350033. | Non randomized controlled trials |
| 203 Liu X, Yang Y, Ma X, et al.The Effect of CT-Guided Artificial Pneumothorax plus Thoracoscopy and Central Venous Catheterization on the Drainage Effect of Pediatric Empyema and Pulmonary Function. Contrast Media & Molecular Imaging, 2022: 8230212. | Wrong study design |
| 204 Huang S, Xiao Y, Li H, Li D. Research on Improving Radiotherapy Accuracy Based on Image-Guided Radiotherapy. Contrast Media Mol Imaging. 2022:9696403. | Non randomized controlled trials |
| 205 Wu C, Sang-Yeol L.The Application of Art Therapy Based on Particle Swarm Optimization Method for Preschool Children’s Mental Health. Wireless Communications and Mobile Computing, 2022: 9261191. | Wrong study design |
| 206 Ma Y, Zhang J, Fan R.Efficacy of Glucocorticoid plus Intravenous Immunoglobulin in Children with Immunoglobulin-Insensitive Kawasaki Disease. Journal of Healthcare Engineering, 2022: 9011259. | Non randomized controlled trials |
| 207 Zhang Y, Zhang Y, Pan J.Efficacy Evaluation of High-Volume Hemofiltration in Patients with Severe Acute Respiratory Distress Syndrome. Evidence-Based Complementary and Alternative Medicine, 2022: 9488047. | Wrong study design |
| 208 Wang M, Pang W, Zhou L, et al.Effect of Transumbilical Single-Port Laparoscopic-Assisted Duhamel Operation on Serum CRP and IL-6 Levels in Children with Hirschsprung’s Disease. Journal of Healthcare Engineering, 2022: 8349851. | Wrong study design |
| 209 Wang T, Dong S, Zhang M, et al.Effect of Solitaire FR Stent Thrombectomy Combined with the Suction Thrombus on the Clinical Effect and Prognosis of Acute Middle Cerebral Artery Occlusion. Evidence-Based Complementary and Alternative Medicine, 2022: 9227790. | Non randomized controlled trials |

| | |
|---|---|
| 210 Ji Q, Wang F, He Q, et al.Effect of Low-Dose Dexmedetomidine Combined with Lumbosacral Plexus Block Guided by Ultrasound Imaging Based on Image Segmentation Algorithm in Fracture Surgery. Computational Intelligence and Neuroscience, 2022: 8063874. | Non randomized controlled trials |
| 211 Yang M, Yang L, Wan L, et al.Clinical Effect of Ear Endoscopic Intervention on CMEC Patients and Analysis of the Relationship between ROS, P-Akt, and HIF-1α Expression and the Degree of Bone Destruction. Contrast Media & Molecular Imaging, 2022: 9931388. | Non randomized controlled trials |
| 212 Pan Y, Han P, Fang F, et al.Analysis of the Correlation of Basic Fibroblast Growth Factor in Serum of Patients with Diffuse Large B-Cell Lymphoma with Clinicopathological Efficacy and International Prognostic Index. Contrast Media & Molecular Imaging, 2022: 8311535. | Wrong study design |
| 213 Deng K, Luo L.Analysis of the Application Value of Different Esophagography Techniques in the Diagnosis of H-Type Tracheoesophageal Fistula in Neonates. Evidence-Based Complementary and Alternative Medicine, 2022: 7264343. | Wrong study design |
| 214 Ren X, Jin J, Chen Y, Jin J. Research on the Effect of Nursing Methods for Gestational Diabetes Mellitus Based on Comprehensive Nursing Intervention. Comput Math Methods Med.2022:2396658. | Non randomized controlled trials |
| 215 Li J N, Zhang X L, Su H, et al.Investigation of the Effects of Large Bone Flap Craniotomy on Cerebral Hemodynamics, Intracranial Infection Rate, and Nerve Function in Patients with Severe Craniocerebral Trauma. Contrast Media & Molecular Imaging, 2022: 2681278. | Non randomized controlled trials |
| 216 Zhang L, Liu H.Influence of Adenoid Hypertrophy on Malocclusion and Maxillofacial Development in Children. Evidence-Based Complementary and Alternative Medicine, 2022: 2052359. | Non randomized controlled trials |
| 217 Yang X, Bao L, Gong X, et al.Impacts of Ultrasound-Guided Nerve Block Combined with General Anesthesia with Laryngeal Mask on the Patients with Lower Extremity Fractures. Journal of Environmental and Public Health, 2022: 3603949. | Wrong study design |
| 218 Li S, Lu H.Functions of Heparin Sodium Injection in the Prevention of Peripherally Inserted Central Catheter-Related Venous Thrombosis in NSCLC Patients during Postoperative Chemotherapy. Computational and mathematical methods in medicine, 2022: 1239058. | Non randomized controlled trials |
| 219 Yang Z, Shao L, Teng Y.Evaluation of the Efficacy and Adverse Reactions of Mirena Combined with Hysteroscopic Surgery When Treating AUB: Based on a Retrospective Cohort Study. Computational and Mathematical Methods in Medicine, 2022: 4082266. | Wrong study design |
| 220 Lu X, Qian C. Efficacy, Safety, and Prognosis of Sequential Therapy with Tamoxifen and Letrozole versus Letrozole Monotherapy for Breast Carcinoma. Comput Math Methods Med.2022:1979254. | Non randomized controlled trials |
| 221 Yang J, Huang Y, Li Y, et al.Efficacy of Flexible Ureteroscopic Lithotripsy and Percutaneous Nephrolithotomy in the Treatment of Complex Upper Urinary Tract Nephrolithiasis. Computational and Mathematical Methods in Medicine, 2022: 2378113. | Wrong study design |

| | |
|---|---|
| 222 Li Z.Efficacy of Fenestration Decompression for the Treatment of Oral and Maxillofacial Cysts. Computational and Mathematical Methods in Medicine, 2022: 2262547. | Wrong study design |
| 223 Cao H, Jin S, Bai B.Efficacy Analysis of DSA-Guided Bronchial Arterial Chemoembolization Interventional Therapy in Patients with Middle-Advanced Primary Bronchial Lung Cancer. Computational and Mathematical Methods in Medicine, 2022: 3722703. | Non randomized controlled trials |
| 224 Wang H, Zhang D, Qian H, et al.Effects of Ulinastatin on Myocardial Ischemia-Reperfusion Injury, Cardiac Function, and Serum TNF-α and IL-10 Levels in Patients Undergoing Cardiac Valve Replacement under Cardiopulmonary Bypass. Computational and Mathematical Methods in Medicine, 2022: 1823398. | Non randomized controlled trials |
| 225 Zhang J, Wang Y, Liu Y, et al.Effects of Modified Sang ju-Yin Decoction Combined with IFNαlb Nebulization on IL-1β and HBD2 in Children with Asthmatic Bronchitis. Computational and Mathematical Methods in Medicine, 2022: 2802636. | Wrong study design |
| 226 Bi M, Meng L, Bai L.Effects of Comprehensive Nursing Based on Orem's Self-Care Theory on Symptom Improvement and Pregnancy Outcome in Patients with Antiphospholipid Syndrome: A Retrospective Cohort Study. Computational and Mathematical Methods in Medicine, 2022: 4133812. | Non randomized controlled trials |
| 227 Liu B, Li Q H, Xie H, et al.Evaluation of Vertebral Function and Long-Term Quality of Life after Percutaneous Minimally Invasive Surgery in Patients with Thoracolumbar Spine Fractures. Computational and Mathematical Methods in Medicine, 2022: 2723542. | Wrong study design |
| 228 Yang M.Effect of Reading Activities on Children's Mental Health under the Environment of Artificial Intelligence and Deep Learning. Journal of Environmental and Public Health, 2022: 1762767. | Non randomized controlled trials |
| 229 Fan L, Yang Y, Zhang F, et al.Clinical Efficacy of Immunoglobulin Combined with Glucocorticoids in the Treatment of Oculomotor Myasthenia Gravis in Children and the Effect on Serum Immunity. Computational and Mathematical Methods in Medicine, 2022: 1772881. | Wrong study design |
| 230 Li L, Chen H, Peng C, et al.Analysis on Value of Continuous Nursing Based on WeChat in Improving Healthy Quality of Life and Self-Management Behavior of Patients with Diabetic Nephropathy. Evidence-Based Complementary and Alternative Medicine, 2022: 5131830. | Wrong study design |
| 231 Ke J, Cai Q, Zhang C, et al.Analysis of the Efficacy and Safety of Pulpitis Treated with Different Root Canal Flushing Fluids Based on VAS and Temporomandibular Joint Function. Contrast Media & Molecular Imaging, 2022: 1470389. | Wrong study design |
| 232 Sun X.The Practical Application of Oil Painting in the Treatment of Mental Illness. Occupational Therapy International, 2022: 1727507. | Wrong study design |
| 233 Feng C, Lv C, Zhang X, et al.Effect of 1+ N Extended Nursing Service on Functional Recovery of Colostomy Patients. Computational Intelligence and Neuroscience, 2022: 2645528. | Non randomized controlled trials |
| 234 Jia H, Wang B, Yu F, et al. RETRACTED: Effect of comprehensive nursing education on self-care ability and psychological state of patients with coronary heart disease after percutaneous coronary intervention. International Journal of Electrical Engineering & Education, 2023, 60(1_suppl): 3836-3848. | No full-text |

| | |
|---|---|
| 235 Wang B, Sun Y, Shi D, et al.Impact of Alendronate Sodium plus Elcatonin on Postoperative Bone Pain in Patients with Osteoporotic Fractures. BioMed Research International, 2022: 1213278. | Wrong study design |
| 236 Lei L, Du T, Yang J, et al.Analysis of the Diagnostic Efficacy of DOTATATE Imaging Combined with CGA and BSP Detection Mode for NEN Patients with Bone Metastasis. BioMed Research International, 2022: 6279826. | Wrong study design |
| 237 Tu M, Zhang A, Hu L, Wang F. A Retrospective Cohort Study of the Efficacy, Safety, and Clinical Value of 6-TG versus 6-MP Maintenance Therapy in Children with Acute Lymphoblastic Leukemia. Biomed Res Int.2022:7580642. | Wrong study design |
| 238 Sun P.Nanotube Combined with Roller Skater Rehabilitation Therapy in the Treatment of Knee Arthritis. Advances in Materials Science and Engineering, 2022: 8286306. | Wrong study design |
| 239 Tang Y, Fu J.Application of Composite Nano-Bone Transplantation and Massage Exercise Rehabilitation Training in Patients Undergoing Limb Replantation. Advances in Materials Science and Engineering, 2022: 2065744. | Wrong study design |
| 240 Angelakis E, Million M, Kankoe S, et al. Abnormal weight gain and gut microbiota modifications are side effects of long-term doxycycline and hydroxychloroquine treatment. Antimicrobial agents and chemotherapy, 2014, 58(6): 3342-3347. | Wrong study design |
| 241 Angelakis E, Oddoze C, Raoult D. Vitamin D and prolonged treatment with photosensitivity-associated antibiotics. Antimicrobial agents and chemotherapy, 2013, 57(12): 6409-6410. | Wrong study design |
| 242 Zhu H, Xiao H, Lu G, et al.Effect of Transdermal Fentanyl Patch Combined with Enhanced Recovery after Surgery on the Curative Effect and Analgesic Effect of Liver Cancer. BioMed Research International, 2022: 9722458. | Wrong study design |
| 243 Wang C, Sun J, Shao J, et al.Clinical Observation and Value Analysis of Endovascular Interventional Therapy for Intracranial Venous Sinus Thrombosis. BioMed Research International, 2022: 4931210. | Wrong study design |
| 244 Wang X, Zhang X, Guan Q, et al.Clinical Effect of Digital Subtraction Angiography Combined with Neurointerventional Thrombolysis for Acute Ischemic Cerebrovascular Disease and Its Influence on Vascular Endothelial Function and Oxidative Stress. Oxidative Medicine and Cellular Longevity, 2022: 2777865. | Wrong study design |
| 245 Zhao Y, Wang X, Zhang D.A Retrospective Study on the Efficacy of Two Different Rehabilitation Interventions on KOA: Shock Wave Therapy vs. Electroacupuncture Therapy. BioMed Research International, 2021: 2099653. | Wrong study design |
| 246 Xu J, Shao H, Yang Y, et al. RETRACTED: Improvement and effect of stress responses and ovarian reserve function in patients with ovarian cysts after laparoscopic surgery. Journal of International Medical Research, 2019, 47(7): 3212-3222. | Wrong study design |
| 247 Zhang M, Zhang X, Wang C, et al.The Role of Jinhuang Powder to Prevent Adverse Effects of Subcutaneous Injection of Enoxaparin Sodium. Emergency Medicine International, 2022: 7806659. | Wrong study design |
| 248 Qie D.The Relevance of Virtual-Assisted Early Childhood Education and Occupational Psychotherapy Based on Emotional Interaction. Occupational Therapy International, 2022: 2785987. | Wrong study design |
| 249 Zhang L, Li Z, Li N.Serum IMA and LP-PLA2 Levels in Patients with Coronary Heart Disease and Their Correlation with the Degree of Myocardial Ischaemia and Their Diagnostic Value. Emergency Medicine International, 2022: 1698315. | Wrong study design |
| 250 Zou H.Music Therapy Methods Based on SVM and MLP. Journal of Mathematics, 2022: 3377809. | Wrong study design |

| | |
|---|---|
| 251 Wang J, Li W.Improvement Effect of PERMA Model-Based Nursing Intervention plus Music Therapy on Patients with Acute Liver Failure Undergoing Plasma Exchange Therapy. Emergency Medicine International, 2022: 2485056. | Non randomized controlled trials |
| 252 Yuan X, Wei H, Liu X, et al.Effects of Stone Removal via Different Approaches in the Treatment of Incarcerated Upper Ureteral Calculi: A Comparative Study. Emergency Medicine International, 2022: 7651215. | Wrong study design |
| 253 Sun M, Zhuang L.Effect of Cognitive Behavioral Therapy on Stress Disorder, Cognitive Function, Motor Function, and Daily Living Ability of Patients with a Traumatic Brain Injury. Emergency medicine international, 2022: 2375344. | Non randomized controlled trials |
| 254 Su L, Li S, Sun B.Curative Effect of Prebiotics/Probiotics-Assisted Ketogenic Diet on Children with Refractory Epilepsy. Emergency Medicine International, 2022: 1076053. | Wrong study design |
| 255 Fei G, Yan H.Clinical Effect of Minimally Invasive Percutaneous Pedicle Screw Internal Fixation Combined with Injured Vertebrae Bone Grafting in the Treatment of Thoracolumbar Fractures in Orthopedic Surgery. Emergency Medicine International, 2022: 3081380. | Wrong study design |
| 256 Fan B, Xiao H, Wu P, et al.Comparison of Curative Effect between PFNA and PCCP in the Treatment of Femoral Intertrochanteric Fractures. Emergency Medicine International, 2022: 5957025. | Wrong study design |
| 257 Shao H, Luo R, You C, et al.Clinical Effect of Emergency Dermabrasion Combined with Biological Dressing A on Wound Microcirculation and Preventing Sepsis in Deep Degree-II Burns. Emergency Medicine International, 2022: 4730905. | Wrong study design |
| 258 He S, Xu H, Liu S.Effect of Arthroscopic Acromioplasty Combined with Rotator Cuff Repair in the Treatment of Aged Patients with Full-Thickness Rotator Cuff Tear and Rotator Cuff Injury. Emergency Medicine International, 2022: 4475087. | Wrong study design |
| 259 Peng D, Sun W. Retracted: Clinical effect of surgical treatment for lung metastasis and prognostic risk factor analysis: a single-centred cohort study. ANZ Journal of Surgery, 2023, 93(6): 1551-1558. | Wrong study design |
| 260 Sen Ö, Yilmaz S, Sen F, et al. Retracted: T-peak to T-end Interval Predicts Appropriate Shocks in Patients with Heart Failure Undergoing Implantable Cardioverter Defibrillator Implantation for Primary Prophylaxis. Annals of Noninvasive Electrocardiology, 2024, 29(1). | Non randomized controlled trials |
| 261 Zhang X, Xing T J. RETRACTED: Evaluation of the safety of retroperitoneal laparoscopic partial nephrectomy by investigating the perioperative indicators. Frontiers in Oncology, 2023, 13: 1138210. | Wrong study design |
| 262 Lukyanenko N, Lenha E, Spaska A, et al. Tactics for treating young children with pyelonephritis and vesicoureteral reflux associated with impaired fibrillogenesis. Mol Cell Biochem. 2023;478(3):531-538. | Non randomized controlled trials |
| 263 Shen F, Li J, Liu F, et al. RETRACTED: The efficacy and adverse effects of anlotinib in the treatment of high-grade glioma: A retrospective analysis. Frontiers in Oncology, 2023, 13: 1095362. | Wrong study design |
| 264 Qiu J, Shi P, Mao W, et al. Effect of apoptosis in neural stem cells treated with sevoflurane. BMC anesthesiology, 2015, 15: 1-8. | Wrong population |
| 265 Zhumalina AK, Tusupkaliev BT, Zharlykasinova MB, et al. The levels of pro- and anti-inflammatory cytokines in premature infants with perinatal infections. Mol Cell Biochem. 2022;477(2):621-625. | Wrong study design |

| | |
|---|---|
| 266 Chi CT, Minh TL, Baxter B, et al. Rescue intracranial stenting in acute ischemic stroke (study). Interv Neuroradiol. 2023:15910199231171272. | Wrong study design |
| 267 Yang T, Niu S L, Wang G D, et al. Two mixed ligand Co (II)-coordination polymers: treatment ability on skin necrosis after fracture and internal fixation by reducing MCSF and TNF-alpha.2021;51:352. | No full-text |
| 268 Wang Y, Jin C, Liu S, et al. Clinical Analysis of Immune-Related Adverse Events in 78 Patients with Advanced Metastatic Lung Cancer Treated with Immune Checkpoint Inhibitors. Journal of Clinical Pharmacology, 2023. | No full-text |
| 269 Boldt J, Zickmann B, Ballesteros M, et al. Right ventricular function in patients with aortic stenosis undergoing aortic valve replacement. Journal of cardiothoracic and vascular anesthesia, 1992, 6(3): 287-291. | Non randomized controlled trials |
| 270 Riedinger C J, Barrington D A, Nagel C I, et al. Cost-effectiveness of chemotherapy and dostarlimab for advanced or recurrent endometrial cancer. Gynecologic oncology, 2024, 183: 78-84. | Non randomized controlled trials |
| 271 Peng Y, Tang S.The Factors Affecting Orthodontic Pain with Periodontitis. Journal of Healthcare Engineering, 2021: 8942979. | Presudo randomization |
| 272 Wang L, Xu Z.Comparison of the Early Results of Lateral Direct Anterior Approach (L-DAA) and Traditional Posterolateral Approach (PLA) in Hip Arthroplasty. Computational and Mathematical Methods in Medicine, 2021: 1187011. | Non randomized controlled trials |
| 273 Liu M, Xu Z, Li H.Effect of Orthodontic Combined with Implant Repair on Aesthetic Effect and Gingival Crevicular Fluid Factor in Patients with Dentition Defect and Periodontitis. BioMed Research International, 2022: 8065313. | Non randomized controlled trials |
| 274 Chang S, Qi Y, Zhou Y, et al.Analysis of Rapid Rehabilitation Effect of Children with Severe Viral Encephalitis Based on Continuous Nursing of Omaha System. Journal of Healthcare Engineering, 2022: 6481697. | Non randomized controlled trials |
| 275 Zhu X, Yang H, Qu M, et al.Application of Intravenous Anesthesia in Laparoscopic Hiatal Hernia Repair of Children. Contrast Media & Molecular Imaging, 2022: 5290813. | Non randomized controlled trials |
| 276 Zhou H, Li M.The Value of Gastric Cancer Staging by Endoscopic Ultrasonography Features in the Diagnosis of Gastroenterology. Computational and Mathematical Methods in Medicine, 2022: 6192190. | Non randomized controlled trials |
| 277 Lu Q, Wu C, Wu Z, et al.Effects of Different Doses of Dex Anesthesia on Inflammatory Factors and Hemodynamics in Patients Undergoing Neurosurgery and Its Relationship with RSS Score. BioMed Research International, 2022: 6447407. | Presudo randomization |
| 278 Hou Y, Yang L.Effective Analysis of Multichannel Functional Electrical Stimulation plus Early Rehabilitation Training for Hemiplegic Patients after Stroke. Evidence-Based Complementary and Alternative Medicine, 2022: 6061652. | Non randomized controlled trials |
| 279 Zhao J, Wang WB, Ding H,et al. Prevention of Dexmedetomidine on Postoperative Delirium and Early Postoperative Cognitive Dysfunction in Elderly Patients Undergoing Thoracoscopic Lobectomy. Evid Based Complement Alternat Med. 2022:5263021. | Non randomized controlled trials |
| 280 Liu C, Luo H, Wang Z, et al.Observation on the Efficacy of Moxibustion Combined with Ear Acupoint Pressing Beans in Treating Patients with Phlegm Stasis Syndrome Vertigo. Emergency Medicine International, 2022: 4295423. | Presudo randomization |
| 281 Yang Y, Xiao Y, Zhang L, et al.Effects of Different Intervention Methods on Intestinal Cleanliness in Children Undergoing Colonoscopy. Journal of Healthcare Engineering, 2022: 1898610. | Presudo randomization |

| | |
|---|---|
| 282 Tang R, Yang J, Wan L, et al.Clinical Effect of Flexible Ureteroscope and Laparoscope in the Treatment of Parapelvic Cyst. BioMed Research International, 2022: 5718923. | Non randomized controlled trials |
| 283 Palomba S, Russo T, Iuzzolino D, et al. Comparison between two laparoscopic retropubic urethropexy. Minerva chirurgica, 2002, 57(3): 323-329. | No full-text |
| 284 Liu H, Zhang H, Wang C, et al. Efficacy and safety of laparoscopic vaginoplasty using the peritoneal flap and cervicoplasty in patients with congenital cervical and complete vaginal atresia: a pilot study. Annals of translational medicine, 2023, 11(6):257. | Non randomized controlled trials |
| 285 Bai C, Ye Q, Zhao Y, et al.MIP-1α Level and Its Correlation with the Risk of Left Atrial Remodeling in Patients with Atrial Fibrillation. Contrast Media & Molecular Imaging, 2022: 1756268. | Non randomized controlled trials |
| 286 Liang Y, Zhu Y, Zhang Y, et al.Clinical Observation and Pharmacoeconomic Evaluations of Original Research Drug and Generic Drug Bortezomib in the Treatment of Multiple Myeloma. Journal of Healthcare Engineering, 2022: 5201354. | Non randomized controlled trials |
| 287 Boldt J, Kling D, Bormann B, et al. Influence of cardiac output on thermal-dye extravascular lung water (EVLW) in cardiac patients. Intensive care medicine, 1987, 13: 310-314. | Non randomized controlled trials |
| 288 Gong X, Wu W, Xing D, et al.Effect of High-Quality Nursing Based on Comprehensive Nursing on the Postoperative Quality of Life and Satisfaction of Patients with Malignant Glioma. Evidence-Based Complementary and Alternative Medicine, 2022: 9345099. | Non randomized controlled trials |
| 289 Wang Y, Xu Z, Chen S, et al.Effects of Medium-Term Soft Contact Lens Fitting on Dry Eye: Analyses Using Ultra-High Resolution Optical Coherence Tomography and Digital Slit-Lamp Biomicroscopy. Disease Markers, 2022: 7220706. | Non randomized controlled trials |
| 290 Song Z, Jiang R, Li C, et al.Menopausal Symptoms and Sleep Quality in Women Aged 40–65 Years. BioMed Research International, 2022: 2560053. | Non randomized controlled trials |
| 291 Wang J, Lin Y, Wei Y, et al.Intervention of WeChat Group Guidance in Rapid Rehabilitation after Gynecological Laparoscopic Surgery. Journal of Healthcare Engineering, 2021: 8914997. | Non randomized controlled trials |
| 292 Nie X.Construction and Application of Comprehensive Nursing Information Service Platform Based on Internet of Things Technology. Journal of healthcare engineering, 2022: 7178531. | Non randomized controlled trials |
| 293 Yang Y, Chen P, Jiao C.Influence of Nursing Intervention Based on Risk Assessment Model on Self-Efficacy and Postoperative Rehabilitation of Surgical Patients. Journal of Healthcare Engineering, 2022: 6750320. | Non randomized controlled trials |
| 294 Guo W, Li W, Mei X, et al.Application of the Concept of Enhanced Recovery after Surgery in Total Laparoscopic Radical Gastrectomy. Journal of Healthcare Engineering, 2022: 5390182. | Non randomized controlled trials |
| 295 Jiang J, Gao S, Han T. Study on the Influencing Mechanism of Human Chorionic Gonadotropin (hCG) on Oocyte Maturation in Patients with Polycystic Ovary Syndrome. Comput Math Methods Med. 2022:7933166. | Non randomized controlled trials |
| 296 Han Y, Jin J, Wu F, et al.Impacts of Low-Dose Total Glycosides of Tripterygium wilfordii plus Methotrexate on Immunological Function and Inflammation Level in Patients with Rheumatoid Arthritis. Computational and Mathematical Methods in Medicine, 2022: 7523673. | Non randomized controlled trials |

| | |
|---|---|
| 297 Wang J, Wang M, Li W.Application of Precise Positioning for Sputum Expectoration in ICU Patients with Pulmonary Infection. Computational and Mathematical Methods in Medicine, 2022: 1395958. | Non randomized controlled trials |
| 298 Song B, Zhang B, An A, et al.Nanocarbon Tracer and Areola Injection Site Are Superior in the Sentinel Lymph Node Biopsy Procedure for Breast Cancer. Computational and Mathematical Methods in Medicine, 2022: 4066179. | Non randomized controlled trials |
| 299 Han X, Li J, Zeng P, et al.Effect of the Kanghuier Transparent Hydrocolloid Dressing in Preventing Central Venous Catheter Infection and Phlebitis after Cardiac Surgery. Computational and Mathematical Methods in Medicine, 2022: 4700257. | Non randomized controlled trials |
| 300 Guo W, Yang S, Gong Y, et al.Application of Absorbable Suture in Strabismus Correction and Nursing Management Advantage of Watson Care Theory in Perioperative Period. Computational and Mathematical Methods in Medicine, 2022: 8739551. | Non randomized controlled trials |
| 301 Liu W, Zhang Y, Liu H, et al.Influence of Health Education Based on IMB on Prognosis and Self-Management Behavior of Patients with Chronic Heart Failure. Computational and Mathematical Methods in Medicine, 2022: 8517802. | Non randomized controlled trials |
| 302 Lu L, Yang J, Zheng J, et al.The Effect of MDT Collaborative Nursing Combined with Hierarchical Nursing Management Model on the Quality of Life and Comfort of Patients with Gallbladder Stones Combined with Acute Cholecystitis after Surgery. Computational and Mathematical Methods in Medicine, 2022: 8696084. | Non randomized controlled trials |
| 303 Cai J, Yuan L, Gao H, et al.Clinical Characteristics and Empirical Research Model of Infectious Mononucleosis Complicated with Mycoplasma pneumoniae or/and Cytomegalovirus Infection. Computational and Mathematical Methods in Medicine, 2021: 2867913. | Non randomized controlled trials |
| 304 Song X, Shao X.Effect of Annular External Fixator-Assisted Bone Transport on Clinical Healing, Pain Stress and Joint Function of Traumatic Massive Bone Defect of Tibia. Computational and Mathematical Methods in Medicine, 2022: 9052770. | Non randomized controlled trials |
| 305 Du K, Hao S, Luan H. Expression of Peripheral Blood DCs CD86, CD80, and Th1/Th2 in Sepsis Patients and Their Value on Survival Prediction. Comput Math Methods Med. 2022:4672535. | Non randomized controlled trials |
| 306 Peng H, Yuan B, Mao J, Wang J, Luo S. Efficacy of Letrozole Combined with Urinary Gonadotropin for Ovulation Induction in Endocrine Abnormal Infertility Patients: A Retrospective Single-Center, Case-Control Study. Comput Math Methods Med.2022:5363754. | Non randomized controlled trials |
| 307 You Z, Zhang H, Zhang X, et al.Therapeutic Effect Analysis of Plasma Bipolar Intelligent Electrotonic for Cystostomy in the Treatment of Senile Prostatic Hyperplasia. Journal of Healthcare Engineering, 2021: 9332848. | Non randomized controlled trials |
| 308 Jiang H, Li H, Wang Z, et al.Effect of Early Cognitive Training Combined with Aerobic Exercise on Quality of Life and Cognitive Function Recovery of Patients with Poststroke Cognitive Impairment. Journal of Healthcare Engineering, 2022: 9891192. | Presudo randomization |
| 309 Yin J.Effect of Acceptance and Commitment Therapy Combined with Music Relaxation Therapy on the Self-Identity of College Students. Journal of healthcare engineering, 2022: 8422903. | Non randomized controlled trials |

| | |
|---|---|
| 310 Liao L, Feng J, Fu X, et al.Comparison between Traditional Chinese Medicine Constitution and Blood Biochemical Markers Associated with Left and Right Mammary Hyperplasia in Rural Areas of Southwest China. Journal of Healthcare Engineering, 2022: 9274060. | Non randomized controlled trials |
| 311 Wang N, Xiao F, Shao H, et al.Clinical Efficacy of Yiqi Yangyin Decoction Combined with Docetaxel on Advanced Ovarian Cancer and the Effect on the Levels of Serum Markers VEGF, HE4, and CA125. Journal of healthcare engineering, 2022: 8401202. | Non randomized controlled trials |
| 312 Dai S, Yao Y, Xia D.Clinical Application of Spiral CT Reconstruction Imaging in Patients with Tracheal Stenosis before Anesthesia. Contrast Media & Molecular Imaging, 2022: 9633527. | Non randomized controlled trials |
| 313 Liu L W, Cai Y C, Tao X L, et al.Changes of Tumor Markers in Patients with Lung Cancer after Immunotherapy and Their Link with Inflammation in the Body. Computational and Mathematical Methods in Medicine, 2022: 7781686. | Non randomized controlled trials |
| 314 Cai J, Jiang D, Zang W.Analysis of the Curative Effect and Influencing Factors of Collagen Sponge Combined with Autologous Skin Graft in the Treatment of Deep Burn Patients. Evidence-Based Complementary and Alternative Medicine, 2021: 6963401. | Non randomized controlled trials |
| 315 Duan Z, Li C, Leung W T, et al.Alterations of Several Serum Parameters Are Associated with Preeclampsia and May Be Potential Markers for the Assessment of PE Severity. Disease markers, 2020: 7815214. | Non randomized controlled trials |
| 316 Zhou Y, Yang B, Yao R, et al.Effects of Insulin Combined with Traditional Chinese Medicine Assisted Comprehensive Nursing Intervention on Oxidative Stress State, Cell Adhesion Factor, and Pregnancy Outcome of Patients with Gestational Diabetes Mellitus. Journal of Healthcare Engineering, 2022: 5330134. | Non randomized controlled trials |
| 317 Chen S, Yang Y, Jiao Y, et al.Efficacy of Drug-Eluting Bead Transarterial Chemoembolization in the Treatment of Colorectal Cancer Liver Metastasis. Journal of Healthcare Engineering, 2022: 4930047. | Non randomized controlled trials |
| 318 Zhao Y, Jiang C, Wu Q, et al.Effects of Endoscopic Sinus Surgery Combined with Budesonide Treatment on Nasal Cavity Function and Serum Inflammatory Factors in Patients with Chronic Sinusitis. Journal of Healthcare Engineering, 2022: 4140682. | Non randomized controlled trials |
| 319 Sun S, Xi Y, Shi X, et al. Study on the Application Value of CT Thin-Layer Scan Data Assisted 3D Printing Technology in Hip and Knee Replacement. J Healthc Eng.2021:3491509. | Non randomized controlled trials |
| 320 Wang L, Wu H, Wang R, et al.Correlation of Presacral Tumour Recurrence with Tumour Metastasis and Long-Term Tumour Recurrence Risk in Patients with Rectal Cancer. Evidence-Based Complementary and Alternative Medicine, 2022: 6202457. | Non randomized controlled trials |
| 321 Mi H, Sun N.Effect of Oxytocin Combined with Different Volume of Water Sac in High-Risk Term Pregnancies. Evidence-Based Complementary and Alternative Medicine, 2022: 1004816. | Non randomized controlled trials |
| 322 Chen S, Zhou S, Lin Y, et al.Self-Expandable Metal Stent as a Bridge to Surgery for Left-Sided Acute Malignant Colorectal Obstruction: Optimal Timing for Elective Surgery. Computational and Mathematical Methods in Medicine, 2022: 6015729. | Non randomized controlled trials |

| | |
|---|---|
| 323 Cao J, Wang H.Effect of Blood Homocysteine on the Outcome of Artificial Insemination in Women with Polycystic Ovary Syndrome. BioMed research international, 2022: 6311419. | Non randomized controlled trials |
| 324 Baimbetov A K, Abzaliev K B, Jukenova A M, et al. The efficacy and safety of cryoballoon catheter ablation in patients with paroxysmal atrial fibrillation. Irish Journal of Medical Science, 2021: 1-7. | Non randomized controlled trials |
| 325 Liu Z, Qiu X, Yang H, et al.The Value of Rivaroxaban Combined with Ticagrelor in Antithrombotic Therapy after PCI in Patients with Nonvalvular Atrial Fibrillation with Acute Coronary Syndrome. Evidence-Based Complementary and Alternative Medicine, 2022: 4807175. | Non randomized controlled trials |
| 326 Ma Y, Liang H, Jin Y.Data Analysis of Nursing Effects in Pediatric Gastroenterology Department under High Content Image Analysis Technology. Contrast Media & Molecular Imaging, 2022: 4302331. | Non randomized controlled trials |
| 327 Yu J, Fan D, Bao L, et al.Analysis of the Effect of Percutaneous Cone Shaping in Patients with Osteoporotic Vertebral Fractures. Contrast Media & Molecular Imaging, 2022: 5188703. | Non randomized controlled trials |
| 328 Zhang C, Yang S, Zhang L, et al.A Cohort Study to Evaluate the Efficacy and Value of CT Perfusion Imaging in Patients with Metastatic Osteosarcoma after Chemotherapy. Computational and Mathematical Methods in Medicine, 2022: 5417753. | Non randomized controlled trials |
| 329 Guo Y, Song Q, Cui Y, et al.Clinical Effects of Primary Nursing on Diabetic Nephropathy Patients Undergoing Hemodialysis and Its Impact on the Inflammatory Responses. Evidence-Based Complementary and Alternative Medicine, 2022: 1011415. | Non randomized controlled trials |
| 330 Shi L, Yang D, Wang X.Clinical Effect of Metronidazole Vaginal Effervescent Tablet Combined with Flavescentis Sophora Suppository in the Treatment of Trichomonas Vaginitis. Contrast Media & Molecular Imaging, 2022: 1250755. | Non randomized controlled trials |
| 331 Zhang T, Ma F.Changes of Entropy Index and Cerebral Oxygen Metabolism in the Maintenance of Remifentanil Anesthesia and Their Predictive Value for Postoperative Hyperalgesia. Computational and Mathematical Methods in Medicine, 2022: 1080858. | Non randomized controlled trials |
| 332 Zhao M, Han J, Li L.IGST Combined with Conventional Drug Therapy and TCM Therapy for Treatment of Bilateral Vestibular Hypofunction. Evidence-Based Complementary and Alternative Medicine, 2022: 5689454. | Presudo randomization |
| 333 Zhang X, Wang H.Application Effect Analysis of Clinical Nursing Pathway in the Care of Neonatal Hypoxic-Ischemic Encephalopathy. Computational and Mathematical Methods in Medicine, 2022: 9379361. | Non randomized controlled trials |
| 334 Zhu L, Liu J, Li Y, et al.Effect of Stereotactic Body Radiation Therapy Combined with Thermoplastic Fixation on Set-Up Errors in Breast Cancer Patients Undergoing Radiotherapy. Computational and Mathematical Methods in Medicine, 2022: 8370842. | Presudo randomization |
| 335 Cao X.Monitoring Mycoplasma pneumoniae-Specific Antibody, C-Reactive Protein, and Procalcitonin Levels in Children with Mycoplasma Pneumonia Is Important. Computational and Mathematical Methods in Medicine, 2022: 7976858. | Non randomized controlled trials |
| 336 Cavallini M, De Luca C, Prussia G, et al. Retracted: PN-HPT®(Polynucleotides Highly Purified Technology) in facial middle third rejuvenation. Exploring the potential. Journal of Cosmetic Dermatology, 2022, 21(2): 615-624. | Non randomized controlled trials |

| | |
|---|---|
| 337 Boldt J, Menges T, Kuhn D, et al. Alterations in circulating vasoactive substances in the critically ill—a comparison between survivors and non-survivors. Intensive care medicine, 1995, 21: 218-225. | Non randomized controlled trials |
| 338 Izmalkov S N, Semenkin O M, Bratiichuk A N. RETRACTED: Corrective Osteotomy of Malunited Fractures of the Distal Radius Using a Combined Surgical Approach. Hand, 2022, 17(4): 740-747. | Non randomized controlled trials |
| 339 Wu H, Liu J, Qian F,et al. Clinical Evaluation of Levetiracetam in the Treatment of Epilepsy. J Healthc Eng.2022:3789516. | Non randomized controlled trials |
| 340 Deng Q, Chen Y, Wang X, et al.Comparison of the Efficacy of Different Insulin Administration and Blood Glucose Monitoring Methods in the Treatment of Type 1 Diabetes Mellitus in Children. Evidence-Based Complementary and Alternative Medicine, 2022: 2862682. | Non randomized controlled trials |
| 341 Zhou X, Yang J, Liu L, et al.Clinical Effect of Butylphthalide Combined with Rt-PA Intravenous Thrombolysis in the Treatment of Acute Cerebral Infarction. Applied Bionics and Biomechanics, 2022: 9215685. | Non randomized controlled trials |
| 342 Boldt J, Muller M, Heesen M, et al. Does age influence circulating adhesion molecules in the critically ill?. Critical care medicine, 1997, 25(1): 95-100. | Non randomized controlled trials |
| 343 Boldt J, Menges T, Wollbruck M, et al. Continuous hemofiltration and platelet function in critically ill patients. Critical care medicine, 1994, 22(7): 1155-1160. | Non randomized controlled trials |
| 344 Boldt J, Menges T, Wollbrück M, et al. Is continuous cardiac output measurement using thermodilution reliable in the critically ill patient?. Critical care medicine, 1994, 22(12): 1913-1918. | Non randomized controlled trials |
| 345 BOLDT J, KLING D, MOOSDORF R, et al. Influence of acute volume loading on right ventricular function after cardiopulmonary bypass. Critical care medicine, 1989, 17(6): 518-522. | Non randomized controlled trials |
| 346 Efficacy of a 12-month weight-loss intervention (Glb-Cva) delivered to adults with stroke: results from a randomized controlled trial | No full-text |
| 347 Li K, Zhang Q, Lu X, Yao S. Effects of Butylphthalide Sodium Chloride Injection Combined with Edaravone Dexborneol on Neurological Function and Serum Inflammatory Factor Levels in Sufferers Having Acute Ischemic Stroke. J Healthc Eng. 2022:1509407. | Non randomized controlled trials |
| 348 Nie T, Chang B, Tian L, et al. Correlation of Complex Impacted Mandibular Teeth with Pericoronitis and Effect of Minimally Invasive Tooth Extraction on Patients' Long-term Outcome of Masticatory Ability. Emerg Med Int.2022:6389900. | Non randomized controlled trials |
| 349 Yin D, Shen G.Aesthetic Effect of Autologous Fat Transplantation on Frontotemporal Depression Filling and Its Influence on SCL-90 and SES of Patients. Emergency Medicine International, 2022: 3374780. | Non randomized controlled trials |
| 350 Zhang Y, Qiu S, Orlova E. The systemic inflammatory response syndrome in acute antipsychotic poisoning. J Biochem Mol Toxicol. 2020;34(10):e22546. | Non randomized controlled trials |
| 351 Noda Y, Sato A, Fujii K, et al. A pilot study of the effect of transcranial magnetic stimulation treatment on cognitive dysfunction associated with post COVID-19 condition. Psychiatry & Clinical Neurosciences, 2023, 77(4):241-242. | No full-text |

| | |
|---|---|
| 352 Li X, Zheng X, Jin B, et al. Safety and Efficacy of Biodegradable Patent Foramen Ovale Occluder in Patients with Migraine: A Clinical Trial. Congenital Heart Disease, 2023, 18(3):373-385. | Non randomized controlled trials |
| 353 Cui L, Li P, Zhang J, et al.Exploring the Effect of Enbrel Softgels on PWI Indicators in VCIND Patients. Journal of Healthcare Engineering, 2022: 9681235. | Non randomized controlled trials |
| 354 Lu X, Tang H, Xu T, et al.The Significance of Three-Dimensional Team Management in the Medical Community Model for Patients with Hypertension and Diabetes. Journal of Healthcare Engineering, 2022: 1960030. | Non randomized controlled trials |
| 355 Zhao G, Shi J, Chen Y.Analysis of Influencing Factors of Serum Stress Index and Prognosis of HICH Patients by Different Anesthesia Methods Combined with Small Bone Window Microsurgery. Journal of Healthcare Engineering, 2022: 6971092. | Non randomized controlled trials |
| 356 Yuan B, Qin X, Xi J.The Comparison of Life Quality between Ultrasound-Guided High-Intensity Focused Ultrasound and Laparoscopic Myomectomy for the Treatment of Uterine Fibroids. Computational and Mathematical Methods in Medicine, 2022: 9604915. | Non randomized controlled trials |
| 357 Zhang Z, Zhang Y, Huang C, et al.Comparison of Clinical Efficacy and Recovery Effect between Lateral and Posterior Foraminoscopy and PELD in LDH Patients. Contrast Media & Molecular Imaging, 2022: 8135322. | Non randomized controlled trials |
| 358 Zhang H, Huang T, Shen J, et al.Clinical Effect of Renal Arterial Sympathetic Radiofrequency Ablation on Secondary Hypertension. Computational and Mathematical Methods in Medicine, 2022: 9948057. | Non randomized controlled trials |
| 359 Chen G, Wang W, Wang P, et al.Clinical Application of Restrictive Brace Combined with Psychological Intervention after Replantation of Severed Fingers in Children. Computational and Mathematical Methods in Medicine, 2022: 9631858. | Non randomized controlled trials |
| 360 Huang Y, Chen H.Analysis of the Influence of Nursing Safety Management on Nursing Quality in Hemodialysis Room. Computational Intelligence and Neuroscience, 2022: 6327425. | Non randomized controlled trials |
| 361 Zhang F, Liao L, Wei S, et al.Risk Factors of Acute Radiation-Induced Lung Injury Induced by Radiotherapy for Esophageal Cancer. Computational and Mathematical Methods in Medicine, 2022: 2416196. | Non randomized controlled trials |
| 362 Meng Q. Study on Strength and Quality Training of Youth Basketball Players. Comput Math Methods Med.2022:4676968. | Non randomized controlled trials |
| 363 Liu Y, Zhang Y, Pang L.Analysis of Related Factors of Mother-to-Child Transmission of AIDS and Evaluation of Measures to Prevent Mother-to-Child Transmission. Computational and Mathematical Methods in Medicine, 2022: 3190370. | Non randomized controlled trials |
| 364 Liu S, Zhang X, Zhou Q.Efficacy of Periodontal Endodontics Combined with Diode Laser (DL) Therapy on Severe Periodontitis. Evidence-Based Complementary and Alternative Medicine, 2022: 5689900. | Non randomized controlled trials |
| 365 Zhou F, Wu Z, Yu Y, et al.Establishment and Application of Pressure Injury Assessment Module in Operating Room Based on Information Management System. Journal of Healthcare Engineering, 2022: 1463826. | Non randomized controlled trials |
| 366 Shen L, Shi W, Cai L, et al.Discuss the Application of Data Services in Data Health Management of High-Risk Pregnant and Lying-In Women in Smart Medical Care. Scanning, 2022: 5957697. | Non randomized controlled trials |

| | |
|---|---|
| 367 Xu J, Li S, Sun Y, et al.Triplanar osteotomy combined with proximal tibial transverse transport to accelerate healing of recalcitrant diabetic foot ulcers. Journal of Orthopaedic Surgery and Research, 2022, 17(1): 528. | Non randomized controlled trials |
| 368 Cag Y, Icten S, Isik-Goren B, et al. A novel approach to managing COVID-19 patients; results of lopinavir plus doxycycline cohort. Eur J Clin Microbiol Infect Dis. 2021;40(2):407-411. | Non randomized controlled trials |
| 369 Yang Q, Mo W, Che X,et al. Clinical Efficacy of Laparoscopic-Assisted Proximal Gastrectomy with Postoperative Double-Channel Digestive Tract Reconstruction: A Case-Control Analysis. Biomed Res Int.2022:1587398. | Non randomized controlled trials |
| 370 Yan H, Xia S, Yao D, et al.The Use of BT-ESD Technology under General Intravenous Anesthesia in the Treatment of Nonmuscle Invasive Bladder Cancer and the Effect of PI3K/Akt Signaling Pathway on Tumor Recurrence. BioMed Research International, 2021: 8860745. | Non randomized controlled trials |
| 371 Asakawa K, Ooka H, Honda M, et al. Effects of chewing gum for tear production in healthy young subjects. Acta Ophthalmologica (1755375X), 2021, 99(8):e1539-e1540. | No full-text |
| 372 Xiao X, Zhou L, Zhang L, et al.Short-Term and Long-Term Curative Effect of Partial Hepatectomy on Ruptured Hemorrhage of Primary Liver Cancer after TAE. Emergency medicine international, 2022: 2484418. | Non randomized controlled trials |
| 373 Wang J, Tao Z, Zhang K, et al.Infection Control-Based Construction of a Fever Outpatient Routine Management Model. Emergency Medicine International, 2022: 2902800. | Non randomized controlled trials |
| 374 Li J, Zhang X.Effectiveness and Safety Analysis of Plasma Beam in the Treatment of Facial Depressed Scars. Emergency Medicine International, 2022: 1194355. | Non randomized controlled trials |
| 375 Lv H, Bi H, Wei J, et al.Effect of MED-TLIF Combined with Percutaneous Pedicle Screw Fixation on Function and Spinal Pelvic Parameters in Patients with Lumbar Spondylolisthesis. Emergency Medicine International, 2022: 2577920. | Non randomized controlled trials |
| 376 Zhao S, Peng L, Mo T, et al.Application and the Effect of the Triple Prerehabilitation Nursing Model in the Perioperative Period of Knee Arthroplasty in Diabetic Patients. Emergency Medicine International, 2022: 1858631. | Non randomized controlled trials |
| 377 Miyata M, Hirabayashi Y, Munakata Y, et al. Feasibility of methotrexate discontinuation following tocilizumab and methotrexate combination therapy in patients with long-standing and advanced rheumatoid arthritis: a 3-year observational cohort study. Fukushima Journal of Medical Science, 2023, 69(1): 11-20. | Non randomized controlled trials |
| 378 Narukawa S, Ishizuka K, Sugimoto K, et al. RETRACTED: Utility of phrenic nerve conduction studies for identification of patients with neuromuscular diseases requiring invasive mechanical ventilation. Muscle & Nerve, 2022, 65(2): 211-216. | Non randomized controlled trials |
| 379 Alborzi S, Keramati P, Younesi M, et al. The impact of laparoscopic cystectomy on ovarian reserve in patients with unilateral and bilateral endometriomas. Fertility and sterility, 2014, 101(2): 427-434. | Non randomized controlled trials |
| 380 Alhammadi O A, Al Hammadi A, Ganesan S, et al. Clinical characteristics of patients with mpox infection in the United Arab Emirates: a prospective cohort study. International Journal of Infectious Diseases, 2023, 134: 303-306. | Non randomized controlled trials |
| 381 B. Zickmann, J. Boldt, Ch. Knothe,et al.Anaesthesia in heart transplantations in neonates and infants.Journal of Cardiothoracic and Vascular Anesthesia.1994,8(5):24. | No full-text |

| | |
|---|---|
| 382 Radavelli Bagatini S, Bondonno C, Dalla Via J, et al. Impact of Provision of Abdominal Aortic Calcification Results on Cardiovascular Risk Reducing Behaviours: A 12-Week RCT. Heart Lung Circ. 2024;33(11):R1. | No full-text |
| 383 Qin Q, Liu H, Yang Y, et al. Probiotic Supplement Preparation Relieves Test Anxiety by Regulating Intestinal Microbiota in College Students.Dis Markers. 2021:5597401. | Non randomized controlled trials |
| 384 Xia Z, Jin H. Application of CT Ultrasonography Combined with Microscopic Intraperitoneal Hyperthermic Perfusion Chemotherapy in Postoperative Treatment of Oocyst Carcinoma. Scanning. 2022:5444552. | Non randomized controlled trials |
| 385 Song J, Chen J, Lin C.Therapeutic Effect of Laparoscopic Cholecystectomy on Patients with Cholecystolithiasis Complicated with Chronic Cholecystitis and Postoperative Quality of Life. Evidence-Based Complementary and Alternative Medicine, 2022: 6813756. | Non randomized controlled trials |
| 386 Huang X, Wu H.Effect of Predictive Nursing Combined with Emotional Therapy on Rehabilitation Effect and Psychological State of Patients with Brain Injury after the Operation. Applied Bionics and Biomechanics, 2022: 4159085. | No mention of randomisation in method |
| 387 Ramachenderam L R, Mashadi A, Wong J S W, et al. Use of proton pump inhibitors (PPI) in patients with acute coronary syndrome (ACS) receiving dual-antiplatelet therapy (DAPT). Clinical Medicine, 2024, 24: 100143. | Wrong study design |
| 388 Feduccia A A, Jerome L, Mithoefer M C, et al. Discontinuation of medications classified as reuptake inhibitors affects treatment response of MDMA-assisted psychotherapy. Psychopharmacology, 2021, 238: 581-588. | Wrong study design |
| 389 Wu J, Liu L, Hu H, et al.Functional Outcome and Inflammatory Response of Patients with Extra-Articular Distal Humeral Fractures following Implantation of Anatomically Precontoured Locking Compression Plates through a Posterior Approach. Evidence-Based Complementary and Alternative Medicine, 2021: 2426298. | Presudo randomization |
| 390 Qiu B, Wang A, Chen Y, et al.Analysis on Effects of Laparoscopic Total Hysterectomy Combined with High Hysterosacral Ligament Suspension in the Treatment for Uterine Prolapse. Evidence-Based Complementary and Alternative Medicine, 2022: 2585529. | No mention of randomisation in method |
| 391 Tang Y, Wei X, Huang Y, et al.Intervention Effect of Traditional Chinese Medicine Hot Pressing Combined with Health Education on the Adolescent's Visual Fatigue. Journal of Healthcare Engineering, 2022: 2450197. | Presudo randomization |
| 392 Huang W K, Hsu H C, Yang T S, et al. Zinc supplementation decreased incidence of grade≥ 2 hand-foot skin reaction induced by regorafenib: A phase II randomized clinical trial. European Journal of Cancer, 2023, 195: 113286. | Wrong study design |
| 393 Abdel-Meguid M E. Dexmedetomidine as anesthetic adjunct for fast tracking and pain control in off-pump coronary artery bypass. Saudi Journal of Anaesthesia, 2013, 7(1): 6. | Presudo randomization |
| 394 Tie M, Chen B, Lv R, et al.Effects of Shenling Chengqi Decoction on Gastrointestinal Function and Immune Status of Patients with Gastrointestinal Injury in Severe Sepsis. Computational and Mathematical Methods in Medicine, 2022: 2219451. | Presudo randomization |
| 395 Tian B, Tian B, Zhang Y.The Efficacy of Mannitol Combined with 6-Aminocaproic Acid in the Treatment of Patients with Cerebral Hemorrhage and Its Impact on Immune Function. Evidence-Based Complementary and Alternative Medicine, 2022: 7396310. | Presudo randomization |

| | |
|---|---|
| 396 Li C, He A.Influence of Diversity Nursing on Patients' Rehabilitation in Cardiology Treatment. Journal of Healthcare Engineering, 2021: 5606660. | No mention of randomisation in method |
| 397 Yu L, Guo Y L, Che T.The Effect of Pilates Exercise Nursing Combined with Communication Standard-Reaching Theory Nursing and Pelvic Floor Muscle Training on Bladder Function and Family Function of Patients after Cervical Cancer Surgery. Computational and Mathematical Methods in Medicine, 2022: 6444462. | No mention of randomisation in method |
| 398 Luo Q, Jiang C, Chen L, et al.Intervention Effect of Lumbar Transforaminal Epidural Block on the Treatment for Low Back Pain with Radicular Pain. Computational and Mathematical Methods in Medicine, 2022: 9146267. | No mention of randomisation in method |
| 399 Shen Y, Lu M, Xu Q, et al.Effect of Bairui Granule on Inflammatory Mediators in Induced Sputum, Leukotriene C4, and EOS in Peripheral Blood of Children with Cough Variant Asthma. Computational and Mathematical Methods in Medicine, 2022: 2657994. | Presudo randomization |
| 400 Chen S, Zhang Y, Shen P, et al.Analysis of the Mechanism of Ureproofing Technology and Postlaparoscopy on Patients with Urology and Infection. Journal of Healthcare Engineering, 2022: 4373416. | No mention of randomisation in method |
| 401 Alves J C, Dos Santos A M M P, Jorge P, et al. Effect of a single intra-articular high molecular weight hyaluronan in a naturally occurring canine osteoarthritis model: a randomized controlled trial. Journal of Orthopaedic Surgery and Research, 2021, 16: 1-14. | Wrong population |
| 402 Tang W, Mu D, Han L,et al. Screening of Clinical Factors Related to Prognosis of Breast Cancer Based on the Cox Proportional Risk Model. J Comput Biol. 2021;28(1):89-98. | Wrong study design |
| 403 Sato Y, Kaji M, Oizomi K. An alternative to vitamin D supplementation to prevent fractures in patients with MS. Neurology. 1999;53(2):437. | Wrong study design |
| 404 Earl CS, Keong TW, An SQ, et al. Haemophilus influenzae responds to glucocorticoids used in asthma therapy by modulation of biofilm formation and antibiotic resistance. EMBO Mol Med. 2015;7(8):1018-1033. | Wrong study design |
| 405 Wiese MN, Kawel-Boehm N, Moreno de la Santa P, et al. Functional results after chest wall stabilization with a new screwless fixation device. Eur J Cardiothorac Surg. 2015;47(5):868-875. | Wrong study design |
| 406 Yu L, Ke W, Wang Y, et al. Predictive and prognostic value of ER-α36 expression in breast cancer patients treated with chemotherapy. Steroids. 2014;84:11-16. | Wrong study design |
| 407 Aksekili MA, Biçici V, Işık Ç, et al. Comparison of early postoperative period electrophysiological and clinical findings following carpal tunnel syndrome: is EMG necessary?. Int J Clin Exp Med. 2015;8(6):10011-10015. | Wrong study design |
| 408 Hou W, Guan J, Lu H,et al. The effects of dexamethasone on the proliferation and apoptosis of human ovarian cancer cells induced by paclitaxel. J Ovarian Res. 2014;7:89. | Wrong study design |
| 409 Rincon N, Xu K, Li J, et al. Blockade of receptor for advanced glycation end products in a model of type 1 diabetic leukoencephalopathy. Diabetes. 2012. | Wrong study design |
| 410 Magawa S, Tanaka H, Furuhashi F, et al. Intrapartum cardiotocogram monitoring between obstetricians and computer analysis. J Matern Fetal Neonatal Med. 2021;34(20):3408-3414. | Wrong study design |

| | |
|---|---|
| 411 Bergek C, Zdolsek JH, Hahn RG. Accuracy of noninvasive haemoglobin measurement by pulse oximetry depends on the type of infusion fluid. Eur J Anaesthesiol. 2013;30(2):73-79. | Wrong study design |
| 412 Liu W, Jiao Y, Xing H, et al. Active surveillance of ventilator-associated pneumonia in the intensive care unit and establishment of the risk grading system and effect evaluation. Ann Transl Med. 2019;7(22):617. | Wrong study design |
| 413 Mohammed M A, Faisal M, Richardson D, et al. Adjusting for illness severity shows there is no difference in patient mortality at weekends or weekdays for emergency medical admissions. Qjm,.2016, 110(7):e1-e8. | Wrong study design |
| 414 Boldt J, Kling D, Scheld H, et al. Age and Cardiac Surgery: Influence on Extravascular Lung Water. Chest. 2023;163(4):997. | Wrong study design |
| 415 Kuo WS, Weng CT, Chen JH, et al. Amelioration of Experimentally Induced Arthritis by Reducing Reactive Oxygen Species Production through the Intra-Articular Injection of Water-Soluble Fullerenol . Nanomaterials (Basel). 2019;9(6):909. | Wrong study design |
| 416 Jiang B, Liu Q, Gai J, Guan J, Li Q. Analysis of Adjuvant Chemotherapy on Pathological Remission of Breast Cancer. Comput Math Methods Med.2021:5440154. | Wrong study design |
| 417 Zhao X, Zhang Y, Chen T, Jia J. Analysis of Clinical Effect after Treatment of Patients with Femoral Neck Fracture Using Total or Hemihip Arthroplasty . Comput Math Methods Med. 2022:7422229. | Wrong study design |
| 418 Luo J, Wang Y, Li W, et al. Analysis of Infection Factors after Radical Mastectomy for Breast Cancer by CT Image and AUTO-plan Intelligent Analysis under Regional Nerve Block. Neurosci Lett. 2020:135214 | Wrong study design |
| 419 Pankov I O. Analysis of the causes of deaths of patients with severe skeletal polytrauma. Prakticheskaya meditsina, 2015, 1(4): 157-160. | Wrong study design |
| 420 Yang S, Guan H, Chen Z, et al. Analysis of the Role of Comprehensive Treatment Model in the Treatment of Prostate Cance. Comput Math Methods Med. 2022:2118823. | Wrong study design |
| 421 Wang Z, Huang J, Wang M,et al. Analysis on the Effects of CT- and Ultrasound-Guided Percutaneous Transthoracic Needle Biopsy Combined with Serum CA125 and CEA on the Diagnosis of Lung Cancer. J Healthc Eng. 2022:2289432. | Wrong study design |
| 422 Ahlin E, Elshafei A, Nur M, et al. Anti-citrullinated peptide antibodies and rheumatoid factor in Sudanese patients with Leishmania donovani infection. Rev Bras Reumatol. 2011;51(6):579-586. | Wrong study design |
| 423 Bo L. AOFAS scores for curative effect analysis on arthroscopic treatment of subtalar ankle instability with osteochondral injury syndrome. Future generation computer systems.2019: 506-510. | Wrong study design |
| 424 Qi Y, Wang X.The application of three-dimensional reconstruction of mandibular neural tube in osteotomy of mandibular angle. World Neurosurg. 2020:S1878-8750(19)33206-1. | Wrong study design |
| 425 Chang CJ, Chen CY, Huang CW. Applications for medical recovery using wireless control of a bluetooth ball with a hybrid G-sensor and human-computer interface technology. Journal of Vibration and Control. 2013;19(8):1139-51. | Wrong study design |
| 426 Mills JK, Minhas JS, Robotham SL. An assessment of the dementia CQUIN–An audit of improving compliance. Dementia. 2014;13(5):697-703. | Wrong study design |

| | |
|---|---|
| 427 Maria A, Asmanova, Natalya V, et al. Assessment of the Influence of Clinical Aspects of Combined Pathology (HIV Infection and Tuberculosis) on the Incidence of Contact in the FocusModern Science: Topical Problems of Theory and Practice. Series: Natural and Technical Sciences. 2020. | Wrong study design |
| 428 Mason AL, Lau JY, Hoang N, et al. Association of diabetes mellitus and chronic hepatitis C virus infection. Hepatology. 1999;29(2):328-333. | Wrong study design |
| 429 Kang JH, Boumenna T, Stein JD, et al. Association of Statin Use and High Serum Cholesterol Levels With Risk of Primary Open-Angle Glaucoma. JAMA Ophthalmol. 2019;137(7):756-765. | Wrong study design |
| 430 Zhao X M, Zhuang P, Li Y, et al. Asymmetry of Subthalamic Neuronal Firing Rate and Oscillatory Characteristics in Parkinson's Disease. Neuropsychiatric Disease and Treatment. 2020: 313-323. | Wrong study design |
| 431 Guan Q, Du B, Teng Z, et al. Bayes clustering and structural support vector machines for segmentation of carotid artery plaques in multicontrast MRI. Comput Math Methods Med. 2012:549102. | Wrong study design |
| 432 Deroux A, Dumestre-Perard C, Khalil-Mgharbel A, et al. The Search for Biomarkers of Bradykinin-Mediated Angio-Oedema Attacks. Int Arch Allergy Immunol. 2016;170(2):108-114. | Wrong study design |
| 433 Hao D, Xu Y, Zhao M, et al. Biosynthesis of Clinacanthus nutans Lindau leaf extract mediated ag NPs, au NPs and their comparative strong muscle relaxant, analgesic activities for pain management in nursing care for using in intensive nursing care unit . J Photochem Photobiol B. 2020;202:111674. | Wrong study design |
| 434 Schulpis KH, Tsakiris S. Brain Damage in Phenylalanine, Homocysteine and Galactose Metabolic Disorders. InMetabolic Encephalopathy.2008. 393-457. | Wrong study design |
| 435 Qin HM, Zheng D, Wu J. Cardiac Rehabilitation Improves Long-Term Prognosis for People with Chronic Kidney Disease Undergoing Percutaneous Coronary Intervention: A Propensity Matching Analysis. J Healthc Eng. 2022:1196682. | Wrong study design |
| 436 Schopfer DW, Takemoto S, Allsup K, et al. Cardiac rehabilitation use among veterans with ischemic heart disease. JAMA Intern Med. 2014;174(10):1687-1689. | Wrong study design |
| 437 Wu P, Yin D, Liu J, et al. Cell membrane based biomimetic nanocomposites for targeted therapy of drug resistant EGFR-mutated lung cancer. Nanoscale. 2019;11(41):19520-19528. | Wrong study design |
| 438 Sato Y, Inose M, Higuchi I,et al. Changes in the supporting muscles of the fractured hip in elderly women. Bone. 2002;30(1):325-330. | Wrong study design |
| 439 Lu Y, Ge S, Liu Y, et al. The Changes of ADC Value, DCE-MRI Parameters and Their Influence on Neuropsychology in Prostate Cancer Patients after Endocrine Therapy Based on Magnetic Resonance Imaging. Neurosci Lett. 2020. | Wrong study design |
| 440 The Scientific World Journal . Changes of Radial Diffusivity and Fractional Anisotopy in the Optic Nerve and Optic Radiation of Glaucoma Patients. ScientificWorldJournal. 2016:5803036. | Wrong study design |
| 441 Sullivan TJ, Dairaghi DJ, Krasinski A,et al. Characterization of CCX140-B, an orally bioavailable antagonist of the CCR2 chemokine receptor, for the treatment of type 2 diabetes and associated complications. Journal of Pharmacology and Experimental Therapeutics. 2012. | Wrong study design |

| | |
|---|---|
| 442 Kupfer Y, Seneviratne C, Chawla K, et al. Chest tube drainage of transudative pleural effusions hastens liberation from mechanical ventilation . Chest. 2011;139(3):519-523. | Wrong study design |
| 443 Wang Q, Li Z, Hu Y, et al. Circ-TFCP2L1 Promotes the Proliferation and Migration of Triple Negative Breast Cancer through Sponging miR-7 by Inhibiting PAK1. J Mammary Gland Biol Neoplasia. 2019;24(4):323-331. | Wrong study design |
| 444 Zhu L W, Liu L. Circular RNA-0007874 (circMTO1) reverses chemoresistance to temozolomide by acting as a sponge of microRNA-630 in glioblastoma by Jiang Rao, Xinxin Cheng, Huimin Zhu, Lifeng Wang, Li Liu. Cell Biol Int. 2019;43(12):1525. | Wrong study design |
| 445 Sun Y, Hu C, Song X. Clinical effect of duloxetine on improving osteoporosis low back pain in older adults. Applied Nanoscience. 2023;13(2):1647-53. | Wrong study design |
| 446 Hosalkar H, Bomar JD. Clinical effectiveness of continuous passive motion (CPM) following femoroacetabular impingement surgery in adolescents . J Child Orthop. 2012;6(4):269-275. | Wrong study design |
| 447 Wang JJ, Fan SJ, Wang LL, et al. Clinical relevance of gemstone spectral CT in the diagnosis of carotid atherosclerosis. Experimental and Therapeutic Medicine. 2017;13(6):2629-36. | Wrong study design |
| 448 Nguyen PN, Nguyen VT. Combination of B-Mode Ultrasound and Doppler Ultrasound in Approaching to Uterine Intracavitary Pathologies Among Women Above 40 Years with Abnormal Uterine Bleeding: A Multicenter-Based Study from Vietnam. J Midlife Health. 2022;13(2):145-151. | Wrong study design |
| 449 Ueshima H, Kitamura A. Combination of Parker Flex-IT™ Stylet and McGRATH MAC for effective double lumen tube intubation.Saudi J Anaesth. 2014;8(4):574. | Wrong study design |
| 450 Feng Q, Liu J, Yao J. Common bile duct stones with situs inversus totalis. ANZ J Surg. 2019;89(11):1527. | Wrong study design |
| 451 Li J, Liu J, Feng G,et al.Common polymorphisms in the MDGA1 gene are associated with bipolar disorder and schizophrenia in the Chinese Han population. Progress in neuro-psychopharmacology & biological psychiatry. 2009. | Wrong study design |
| 452 Franks PW, Christophi CA, Jablonski KA,et al. Common variation in PPARGC1A/B and progression to diabetes or change in metabolic traits following preventive interventions: the Diabetes Prevention Program. Diabetologia. 2013;56:2102. | Wrong study design |
| 453 Zamani M, Masoumi B, Esmailian M, et al. A Comparative Analysis of Diagnostic Accuracy of Focused Assessment With Sonography for Trauma Performed by Emergency Medicine and Radiology Residents. Iran Red Crescent Med J. 2015;17(12):e20302. | Wrong study design |
| 454 Jentschke M, Chen K, Arbyn M, et al. Comparative evaluation of two vaginal self-sampling devices for the detection of human papillomavirus infections. J Clin Virol. 2015: S1386-6532. | Wrong study design |
| 455 Hou Y, Shi G, Shi J, et al. A Comparative Study Between Anterior Controllable Antedisplacement and Fusion Versus Laminoplasty in the Surgical Management of Multilevel Cervical Ossification of the Posterior Longitudinal Ligament. World Neurosurg. 2018:S1878-8750(18)32147-8. | Wrong study design |

| | |
|---|---|
| 456 Zou D, Wu C, Miao J, et al. A Comparative Study of ARHI Imprinted Gene Detection and Fine-Needle Aspiration Cytology in the Differential Diagnosis of Benign and Malignant Thyroid Nodules. Genet Test Mol Biomarkers. 2019;23(9):681-687. | Wrong study design |
| 457 Wang Y, Nogueira R, Fan L X. A comparison of bioelectrical impedance analysis and skinfold measurements with Medix DR Dual-energy X-ray absorptiometry for assessment of body fat percentage.2019: 173-e1. | Wrong study design |
| 458 Iwai K, Tsujita Y. A Comparison of Early Rehabilitation in the Intensive Care Units of Patients With Severe COVID-19: A Propensity Score Matching Analysis . Cureus. 2022;14(11):e31328. | Wrong study design |
| 459 Di Giacomo M. Comparison of three peripherally-inserted central catheters: pilot study. Br J Nurs. 2009;18(1):8-16. | Wrong study design |
| 460 Stenroos A, Laihinen T, Lybäck C, et al. Complementary Cast Immobilization Is Not Necessary after Intramedullary Fixation of Unstable Tibial Shaft Fractures in Pediatric Patients. Eur J Pediatr Surg. 2020. | Wrong study design |
| 461 Elahi M M, Kirke R, Lee D, et al. The complications of repeat median sternotomy in paediatrics: six-months follow-up of consecutive cases. Interact Cardiovasc Thorac Surg. 2014;19(5):886. | Wrong study design |
| 462 Singh A, Dilnawaz F, Mewar S, et al. Composite polymeric magnetic nanoparticles for co-delivery of hydrophobic and hydrophilic anticancer drugs and MRI imaging for cancer therapy . ACS Appl Mater Interfaces. 2011;3(3):842-856. | Wrong study design |
| 463 Lambert PW, DeOreo PB, Hollis BW, et al. Concurrent measurement of plasma levels of vitamin D3 and five of its metabolites in normal humans, chronic renal failure patients, and anephric subjects. J Lab Clin Med. 1981,98(4):536-548. | Wrong study design |
| 464 Pang Z, Wang X, Wu Y, et al. Construction and Empirical Study of Nursing Quality Evaluation Index System of Hepatobiliary Surgery Based on ERAS Concept. Comput Math Methods Med. 2022:1117880. | Wrong study design |
| 465 Huajun L, Zhao L, Xuemei M, et al. Correlation analysis of biochemical indicators in common bile duct stone patients with negative magnetic resonance cholangiopancreatography. 2019: 530-535. | Wrong study design |
| 466 Malin SR, Srivastava BJ. Correlation between heart attacks and magnetic activity.Nature. 1979;277(5698):646-648. | Wrong study design |
| 467 Stępień E, Konkolewska M, Kapusta M, et al. Correlation between the number and origin of circulating microparticles and fibrin clot properties in patients with coronary artery disease. Int J Cardiol. 2015;181:147-148. | Wrong study design |
| 468 Mancevski B, Gogate J, Ashcroft K, et al. Correlation of Caregiver Burden and Baseline Characteristics of Caregivers and Patients in Their Care with Recent-Onset Psychosis: The FIRST Study.Psychopharmacol Bull. 2017;47(3):17-25. | Wrong study design |
| 469 Watson DA, Ross SA. Corticosteroids for the complications of Ross River virus infection. Med J Aust. 1998;168(2):92. | Wrong study design |
| 470 Schwentner C, Todenhöfer T, Seibold J, et al. Cost effective laparoendoscopic single-site surgery with a reusable platform. JSLS. 2013;17(2):285-291. | Wrong study design |
| 471 Sato Y, Kondo I, Ishida S, et al. Decreased bone mass and increased bone turnover with valproate therapy in adults with epilepsy.Neurology. 2001;57(3):445-449. | Wrong study design |
| 472 Zong H, Zhang Y, You Y, et al. Decreased Warburg effect induced by ATP citrate lyase suppression inhibits tumor growth in pancreatic cancer.Med Oncol. 2015;32(3):85. | Wrong study design |

| | |
|---|---|
| 473 Hurlstone DP, Kiesslich R, Hunter MD, et al. Defining In Vivo Mucosal Inflammatory Activity Using Laser Scanning Confocal Endomicroscopy in Human Ulcerative Colitis. Gastroenterology.2008. | Wrong study design |
| 474 Miller DL, Watkins KT, Helms GA, et al.Dehydrated Human Amnion/Chorion Membrane (Placenta) Reduces Anastomotic Leaks after Esophagectomy.Ann Thorac Surg. 2020. | Wrong study design |
| 475 Hoskins W, Pollard H. A descriptive study of a manual therapy intervention within a randomised controlled trial for hamstring and lower limb injury prevention.Chiropr Osteopat. 2010;18:23. | Wrong study design |
| 476 Fathi M, Hasani S A, Zare M A, et al. Diagnostic accuracy of emergency physician performed graded compression ultrasound study in acute appendicitis: a prospective study. Journal of ultrasound, 2015, 18: 57-62 | Wrong study design |
| 477 Olalla S, Monleon J, Cristóbal I, Cañete ML. Diagnostic evaluation of uterine myomas. Eur J Obstet Gynecol Reprod Biol. 2020. | Wrong study design |
| 478 Wu G, Jin T, Li T, et al. The diagnostic value of time-resolved MR angiography with Gadobutrol at 3T for preoperative evaluation of lower extremity tumors: Comparison with computed tomography angiography.Eur J Radiol Open. 2020;7:100224. | Wrong study design |
| 479 Vladimir Vladimirovich, Maslyakov, Vitaly Gennadievic, Barsukov, Alexander Vyacheslavovich, Uskov,Diagnostics of Neck Gunshot Injuries in the Civil Population in the Conditions of Local Military Conflict by the Forces of Civil HealthcareIn the World of Scientific Discoveries. 2016. | Wrong study design |
| 480 Tan D, Zhang Y. Differences in Outcome and Comparison of Stress and Immune Status in Patients with Recurrent Common Bile Duct Stones after Biliary Tract Surgery Choosing Three Procedures (ERCP, OCBDE, and LCBDE) for Treatment. Comput Math Methods Med. 2022:9197990. | Wrong study design |
| 481 Ali M, Lange SA, Wittlinger T, et al. Direct transfer of STEMI patients to cardiac catheterization laboratory : Prognostic relevance for in-hospital mortality. Herz. 2019;44(5):460. | Wrong study design |
| 482 Kwon JH, Denlinger CE. Does dehydrated human amnion/chorion membrane enhance esophageal anastomotic healing? Ann Thorac Surg. 2020. | Wrong study design |
| 483 Doolub G, Forfar C. DOES INTRACORONARY ADENOSINE INJECTION DURING PRIMARY PCI REDUCE MICROVASCULAR OBSTRUCTION IN PATIENTS ADMITTED WITH STEMI?. 2013: E134-E134. | Wrong study design |
| 484 Ouyang T, Liu J, Shi C, et al. Drug-Eluting Bead Transarterial Chemoembolization versus Conventional Transarterial Chemoembolization Both Combined Apatinib for Hepatocellular Carcinoma: A Retrospective, Propensity-Score Matched Study.J Hepatocell Carcinoma. 2021;8:1459-1471. | Wrong study design |
| 485 Wei J, Zhu R, Zhang H,et al.Dynamic Observation of Postoperative Infection and Neuron-specific Enolase Levels in Serum in Patients with Non-small Cell Lung Cancer According to Positron Emission Tomography–Computed Tomography.2020: 135226. | Wrong study design |
| 486 R NicolescuEarly nutrition, growth trajectories and later metabolic risk - forward more positive correlationsRomanian Journal of Diabetes Nutrition and Metabolic Diseases. 2008. | Wrong study design |

| | |
|---|---|
| 487 Zheng X, Li X, Xu J, et al. Effect of abdominal aortic balloon occlusion timing on femoral nerve injury in patients with dangerous placenta preview based on color dropper ultrasound images. World Neurosurg. 2020 | Wrong study design |
| 488 El Askary A, Shafie A, Almehmadi M, et al. Effect of Application of Treadmill Training on Metabolic Control and Vitamin D Level in Saudi Patients with Type 2 Diabetes Mellitus.Comput Math Methods Med. 2022:3059629. | Wrong study design |
| 489 Tenjin T, Miyamoto S, Miyake N, et al. Effect of blonanserin on cognitive function in antipsychotic-naïve first-episode schizophrenia. Human Psychopharmacology: Clinical and Experimental. 2012 ;27(1):90-100. | Wrong study design |
| 490 Feng D, Hu L, Hao J, et al. Effect of dexmedetomidine on comfort and satisfaction of patients . 2020: 1151-1151. | Wrong study design |
| 491 Shah M, Shah S, Gandhi B. Effect of DICOM workflow on electronic data management in ophthalmology. Astrocyte, 2015, 2(1): 31-31. | Wrong study design |
| 492 Nanba Y, Miyamoto S, Takemasa S, et al. Effect of exercise at light loads with manipulative resistance on infraspinatus, trapezius (upper fiber) and deltoid (middle fiber) muscle activities in shoulder joint elevation . J Phys Ther Sci. 2015;27(3):627-629. | Wrong study design |
| 493 Lee JM, Lee JH, Tong SY, et al. The effect of HER-2 polymorphism according to age on the risk and pathologic feature of endometrial cancer .Obstet Gynecol Sci. 2014;57(5):425. | Wrong study design |
| 494 Dai L. Effect of Hierarchical Nursing Management in Patients with Hypertension Complicated with Cardiovascular and Cerebrovascular Risk Factors. Comput Math Methods Med.2021:1246566. | Wrong study design |
| 495 Cheng W, Ju J, Sun Y, et al. The effect of LED lighting on color discrimination and preference of elderly people. Human Factors and Ergonomics in Manufacturing & Service Industries, 2016, 26(4): 483-490. | Wrong study design |
| 496 Pirat B, Yildirir A, Simsek V, et al.The effect of leg lifting on tissue doppler parameters in severe coronary artery disease. SCANDINAVIAN CARDIOVASCULAR JOURNAL. 2009;43(1):80. | Wrong study design |
| 497 Chen J, Gu Z, Pan Y, et al. Effect of Radical Laparoscopic Surgery and Conventional Open Surgery on Surgical Outcomes, Complications, and Prognosis in Elderly Patients with Bladder Cancer.Evid Based Complement Alternat Med.2022:1681038. | Wrong study design |
| 498 Li R, Li L, Chen Q. Effect of Respiratory Training Combined with Core Muscle Training on the Overall Motor Function and Activities of Daily Living of Patients with Early and Midterm Stroke.J Healthc Eng. 2022:2830711. | Wrong study design |
| 499 Zhou MH, Kansagra AP. Effect of routing paradigm on patient-centered outcomes in acute ischemic stroke.J Neurointerv Surg. 2019;11(3):251-256. | Wrong study design |
| 500 Koh YG, Lee JA, Lee HY, et al. Effect of sagittal femoral component alignment on biomechanics after mobile-bearing total knee arthroplasty . J Orthop Surg Res. 2019;14(1):400. | Wrong study design |
| 501 Mei ML, Yan Z, Duangthip D, et al. Effect of silver diamine fluoride on plaque microbiome in children. J Dent. 2020;102:103479. | Wrong study design |

| | |
|---|---|
| 502 Sheng Y, Li Y, Feng T. Effect of Yifei-Huoxue Decoction Combined with Tiotropium on Inflammatory Cytokine Levels, Pulmonary Function, and Quality of Life in Patients with Chronic Obstructive Pulmonary Disease .Comput Intell Neurosci. 2022:5740181. | Wrong study design |
| 503 Ngemu EK, Khayeka-Wandabwa C, Kweka EJ, et al. Effectiveness of option B highly active antiretroviral therapy (HAART) prevention of mother-to-child transmission (PMTCT) in pregnant HIV women. BMC Res Notes. 2014;7:52. | Wrong study design |
| 504 Bae S, Kim MC, Kim JY, et al. Effectiveness of Surgical and Cotton Masks in Blocking SARS-CoV-2: A Controlled Comparison in 4 Patients. Ann Intern Med. 2020;173(1):W22-W23. | Wrong study design |
| 505 Yener AU, Ozcan S, Budak AB, et al. The effects of 21 and 23 milimeter aortic valve prosthesis on hemodynamic performance and functional capacity in young adults.Pak J Med Sci. 2014;30(2):356-360. | Wrong study design |
| 506 Qu L, Zhou M, Yu Y, et al. Effects of Nutritious Meal Combined with Online Publicity and Education on Postoperative Nutrition and Psychological State in Patients with Low Rectal Cancer After Colostomy.Comput Math Methods Med.2022:1541385. | Wrong study design |
| 507 Stubgen J P. Effects of recombinant type 1 interferon therapy on human muscle diseases. Muscle Nerve. 2010;42(1):150. | Wrong study design |
| 508 London RE, Slagter HA. Effects of Transcranial Direct Current Stimulation over Left Dorsolateral pFC on the Attentional Blink Depend on Individual Baseline Performance. J Cogn Neurosci. 2015;27(12):2382-2393. | Wrong study design |
| 509 Kuroki K, Nogami A, Yoshida K, et al. Efficacy of Intensive Radiofrequency Energy Delivery to the Localized Dense Scar Area in Post-Infarction Ventricular Tachycardia Ablation- A Comparative Study With Standard Strategy Targeting the Infarcted Border Zone. Circ J. 2017;81(11):1603-1610. | Wrong study design |
| 510 Feng W,Yong Y.Enhancement of doxorubicin efficacy through suppression of serine synthesis in triple-negative breast cancer. Breast Cancer Res Treat. 2015;151(2):477. | Wrong study design |
| 511 Ye T, Ouyang Y, Chen A. Evaluation of coracoclavicular stabilization of acute acromioclavicular joint dislocation with multistrand titanium cables.Eur J Orthop Surg Traumatol. 2014;24(7):1061-1066. | Wrong study design |
| 512 Yao G, Zhang G, Ling L.Evaluation of renal vascular lesions using thrombomodulin and vascular cell adhesion molecule-1 in patients with biopsy-proven lupus nephritis .Clin Rheumatol. 2023;42(1):1. | Wrong study design |
| 513 Dayem RN. Evaluation of the ablation efficacy and morphology of some hard tissues irradiated with different types and modes of laser. Lasers in Medical Science. 2022;37:1. | Wrong study design |
| 514 Saha SG, Vijaywargiya N, Saxena D, et al. Evaluation of the incidence of microcracks caused by Mtwo and ProTaper Next rotary file systems versus the self-adjusting file: A scanning electron microscopic study. J Conserv Dent. 2017;20(5):355-359. | Wrong study design |
| 515 Näher H, Schüle T, Petzoldt D. Evidence for genetic HIV variants from detection of HIV-DNA . Lancet. 1991;338(8765):519-520. | Wrong study design |

| | |
|---|---|
| 516 Lu X, Chen F, Yuan D, et al. Exosome-derived PTENP1 suppresses cisplatin resistance of bladder cancer (BC) by suppressing cell proliferation, migration and inducing apoptosis via the miR-103a/PDCD4 axis.RSC Adv. 2019;9(64):37642-37651. | Wrong study design |
| 517 Papp K, Gottlieb A B, Naldi L, et al. Experience with ustekinumab in patients with psoriasis enrolled in a large, multicenter, prospective, disease-based registry (Psoriasis Longitudinal Assessment and Registry [PSOLAR]). 2015. | Wrong study design |
| 518 Zhang L, Cui Y, Wang YC, et al. Exploring the mechanism by which accumbal deep brain stimulation attenuates morphine-induced reinstatement through manganese-enhanced MRI and pharmacological intervention.2017: 29-40. | Wrong study design |
| 519 Martin MA, Meyricke R, O'Neill T, et al. Factors affecting hospital readmission rates for breast cancer patients in Western Australia.J Surg Oncol. 2007. | Wrong study design |
| 520 Chapman J, Asherov A, Wang N, et al. Familial Alzheimer's disease associated with S182 codon 286 mutation. Lancet. 1995;346(8981):1040. | Wrong study design |
| 521 Jian S, Yongming Q, Zhihua C, et al. Feasibility and safety of moderate hypothermia after acute ischemic stroke. Int J Dev Neurosci. 2003;21(6):353-356. | Wrong study design |
| 522 Çatma MF, Ünlü S, Öztürk A, et al. Femoral shortening osteotomy in total hip arthroplasty for severe dysplasia: a comparison of two fixation techniques.Int Orthop. 2016;40(11):2271-2276. | Wrong study design |
| 523 Koren G, Gilboa D, Katz R. Fetal Safety of Dydrogesterone Exposure in the First Trimester of Pregnancy. Clin Drug Investig. 2020;40(7):679. | Wrong study design |
| 524 Novolodskiy, Boris V, Taevsky, et al. Flow charts as a tool in assessing the performance of the ophthalmological servicePractical Medicine. 2015. | Wrong study design |
| 525 Noda Y, Yasuda T, Kanzaki R, et al. Functional analysis of newly identified RYR1 variants in patients susceptible to malignant hyperthermia . J Anesth. 2020;34(5):658-665. | Wrong study design |
| 526 Huang GF, Chou YL, Su FC. Gait analysis and energy consumption of below-knee amputees wearing three different prosthetic feet. Gait Posture. 2000;12(2):162-168. | Wrong study design |
| 527 Vilar KM, Pereira MC, Dantas AT, et al. Galectin-1, -4, and -7 Were Associated with High Activity of Disease in Patients with Rheumatoid Arthritis.Autoimmune Dis. 2019:3081621. | Wrong study design |
| 528 Nuttall FQ, Gannon MC, Hoover H. Glycemic response to ingested dreamfields pasta compared with traditional pasta. Diabetes Care. 2011;34(2):e17-e18. | Wrong study design |
| 529 Wang J, Wang J, Dai J, et al. A Glycolytic Mechanism Regulating an Angiogenic Switch in Prostate Cancer . Cancer Res. 2021;81(6):1623. | Wrong study design |
| 530 Ninis VN, Kýlýnç MO, Kandemir M, et al. High frequency of T9 and CFTR mutations in children with idiopathic bronchiectasis. J Med Genet. 2003;40(7):530-535. | Wrong study design |
| 531 Yao Q, Tian W, Qiu L. High-resolution ultrasound Images in gouty arthritis to Evaluate Relationship between tophi and bone erosion. 2019. | Wrong study design |

| | |
|---|---|
| 532 BRADU A, PODOLEANU A, ROSEN R B. High-speed en-face optical coherence tomography system for closed loop adaptive optics for the retina. Rom. J. Biophys, 2005, 15(1-4): 113-119. | Wrong study design |
| 533 Van Le TS, Miller R, Barder T, et al. Highly specific urine-based marker of bladder cancer.Urology. 2005;66(6):1256-1260. | Wrong study design |
| 534 Ruhullah M, Singh HR, Shah S, et al. Hip spica versus Rush pins for management of femoral diaphyseal fractures in children. Indian J Orthop. 2014;48(5):488-494. | Wrong study design |
| 535 Kalliantas D, Kallianta M, Karagianni CS. Homeopathy combat against coronavirus disease (Covid-19). Z Gesundh Wiss. 2021;29(1):253. | Wrong study design |
| 536 Irshad M, Khattak S A, Hassan M M, et al. How perceived threat of Covid-19 causes turnover intention among Pakistani nurses: A moderation and mediation analysis. Int J Ment Health Nurs. 2021;30(1):350. | Wrong study design |
| 537 Silva A M T C, Vilanova-Costa C A S T, de Oliveira S F, et al. Human papillomavirus detection and genotyping in squamous cell carcinomas of the larynx.J Virol Methods. 2009;157(2):231. | Wrong study design |
| 538 Qian L, Ji AH, Zhang WJ, et al. HuR, TTP, and miR-133b expression in NSCLC and their association with prognosis. Eur Rev Med Pharmacol Sci. 2018;22(2):430-442. | Wrong study design |
| 539 Piao X, Zou Y, Sui X, et al. Hydrostatin-SN10 Ameliorates Pancreatitis-Induced Lung Injury by Affecting IL-6-Induced JAK2/STAT3-Associated Inflammation and Oxidative Stress.Oxid Med Cell Longev. 2019:9659757. | Wrong study design |
| 540 Davido B, Lansaman T, Bessis S, et al. Hydroxychloroquine plus azithromycin: a potential interest in reducing in-hospital morbidity due to COVID-19 pneumonia (HI-ZY-COVID)?.MedRxiv, 2020. | Wrong study design |
| 541 Chino F, Suneja G, Chino J. Image Guided HDR Brachytherapy for Pelvic Sidewall Recurrence of Endometrial Cancer. 2018. | Wrong study design |
| 542 Liu Y, Zhou J, Yu Z,et al.Immunological properties reveal the monovalent and bivalent recombinant dengue virus-like particles as candidate vaccine for dengue.Microbiol Immunol. 2015;59(3):181. | Wrong study design |
| 543 Sepiashvili R.Immunorehabilitology: From Immunotherapy to Personalized Targeted Immunorehabilitation. International Journal on Immunorehabilitation, 2016, 18(2): 65-71. | Wrong study design |
| 544 Sepiashvili R. Immunotherapy of HIV-infected patients with Gc protein-derived macrophage activating factor.J Med Virol. 2014;86(11):1998. | Wrong study design |
| 545 Torregiani G, Claroni C, Covotta M, et al. Impact of a goal-directed fluid therapy on length of hospital stay and costs of hepatobiliarypancreatic surgery: a prospective observational study. J Comp Eff Res. 2018;7(12):1171-1179. | Wrong study design |
| 546 Okuyama H, Hirono O, Tamura H, et al. Impact of aortic arch stiffness on recurrence of stroke in patients with acute ischemic stroke. Circ J. 2008;72(8):1296-1302. | Wrong study design |
| 547 Alyaa SN, Ban SD. The Impact of Feeding Pattern on Gingival Health Condition and Salivary Adiponectin Level in a Relation to Body Composition Among Primary School Children in Baghdad City IraqJournal of Research in Medical and Dental Science. 2020. | Wrong study design |

| | |
|---|---|
| 548 Yang Z, Hu Q, Liu D, et al. Implementation of specially designed enhanced recovery after surgery protocols versus conventional protocol in total pelvic floor reconstructive surgery. Int J Gynaecol Obstet. 2020;149(2):255. | Wrong study design |
| 549 Etemadifar M, Aghababaee A, Sedaghat N, et al. Incidence and mortality of COVID-19 in Iranian multiple sclerosis patients treated with disease-modifying therapies. Rev Neurol (Paris). 2020. | Wrong study design |
| 550 Song S, Song Y, Zhang H, et al. Increased counts and degranulation of duodenal mast cells and eosinophils in functional dyspepsia- a clinical study . Med Glas (Zenica). 2014;11(2):276-282. | Wrong study design |
| 551 Dong Z, Yang X, Chang L, et al.Influence of MRI on Diagnostic Efficacy and Satisfaction of Patients with Alzheimer's Disease. Computational and Mathematical Methods in Medicine, 2021, 2021(1): 9038784. | Wrong study design |
| 552 Alexander N, Legotkin, Anna B, et al. Influence of Physical Loading on Biological AgeInternational Research Journal. 2016. | Wrong study design |
| 553 Abou-Madina MM, Özcan M, Abdelaziz KM. Influence of resin cements and aging on the fracture resistance of IPS e.max press posterior crowns. Int J Prosthodont. 2012;25(1):33-35. | Wrong study design |
| 554 Jain AL. Influence of vitamins and trace-elements on the incidence of respiratory infection in the elderly. Nutr Res. 2016;36(7):758. | Wrong study design |
| 555 Zhan W J, Zhu J F, Zhang Y. Inhibition of Corneal Neovascularization by Hydrazinocurcumin. Tropical Journal of Pharmaceutical Research, 2016, 15(2): 349-354. | Wrong study design |
| 556 Wu W, Yu LH, Ma B, et al. The Inhibitory Effect of Doxycycline on Cisplatin-Sensitive and -Resistant Epithelial Ovarian Cancer. PLoS One. 2020;15(4):e0231890. | Wrong study design |
| 557 Cole WR, Gregory E, Arrieux JP, et al. Intraindividual Cognitive Variability: An Examination of ANAM4 TBI-MIL Simple Reaction Time Data from Service Members with and without Mild Traumatic Brain Injury. J Int Neuropsychol Soc. 2018;24(2):156-162. | Wrong study design |
| 558 Jiang Z, Chen Z, Chen Y, et al. Involvement of pro-inflammatory cytokines in diabetic neuropathic pain via central PI3K/Akt/mTOR signal pathway.Arch Physiol Biochem. 2021;127(6):I-IX. | Wrong study design |
| 559 Lin JC, Lin SC, Mar EC, et al. Is Kaposi's-sarcoma-associated herpesvirus detectable in semen of HIV-infected homosexual men?.Lancet. 1995;346(8990):1601-1602. | Wrong study design |
| 560 Patel A, Desai S, Grainger D W, et al. Ivermectin in COVID-19 related critical illness. Available at SSRN. 2020: 3570270. | Wrong study design |
| 561 Morga E, Mouad-Amazzal L, Felten P, et al. Jagged1 regulates the activation of astrocytes via modulation of NFkappaB and JAK/STAT/SOCS pathways.Glia. 2009;57(16):1741-1753. | Wrong study design |
| 562 Huschak G, Holzhausen HJ, Beier A, et al. Lack of Relationship Between Occupational Workload and Microscopic Alterations in Lumbar Intervertebral Disc Disease. Open Orthop J. 2017;11:389. | Wrong study design |
| 563 Ueshima H, Otake H. The lateral transversus thoracic muscle plane block is effective for the pericardial drainag. J Clin Anesth. 2017;42:12. | Wrong study design |

| | |
|---|---|
| 564 Liu Y, Li M, Yu H, et al. LncRNA SRA1 is down-regulated in HPV-negative cervical squamous cell carcinoma and regulates cancer cell behaviors. Biosci Rep. 2019;39(8):BSR20191226. | Wrong study design |
| 565 Simoglou C, Tsolakis N. Lobectomy after three-dimensional computed tomography of the pulmonary artery .Asian Cardiovasc Thorac Ann. 2014;22(9):1080-1083. | Wrong study design |
| 566 Zhang C, Du S, Cao L. Long non-coding RNA KCNQ1OT1 promotes osteosarcoma progression by increasing β-catenin activity. RSC Adv. 2018;8(66):37581-37589. | Wrong study design |
| 567 Zhang N, Li S, Hua H, et al. Low density lipoprotein receptor targeted doxorubicin/DNA-Gold Nanorods as a chemo- and thermo-dual therapy for prostate cancer. Int J Pharm. 2016;513(1-2):376-386. | Wrong study design |
| 568 Zalazar F, De Luca P, Gardner K, et al. Low doses of CPS49 and flavopiridol combination as potential treatment for advanced prostate cancer. Curr Pharm Biotechnol. 2015;16(6):553-563. | Wrong study design |
| 569 Falci DR, Pasqualotto AC, Nucci M, et al. Low Sensitivity of Lateral-Flow Device-Aspergillus in Patients with Probable and Proven Aspergillosis: Results from a Multicentre Evaluation. J Clin Microbiol. 2018;56(5):e01864-17. | Wrong study design |
| 570 Ueshima H, Hiroshi O. Lumbar vertebra surgery performed with a bilateral posterior quadratus lumborum block.J Clin Anesth. 2017;41:61. | Wrong study design |
| 571 Rzymowska J. Magnesium and iron contents of leukemic lymphocytes in acute leukemias and hemolytic anemia.Biol Trace Elem Res. 2014;162(1-3):360. | Wrong study design |
| 572 Kim J, Ebertowski J, Janiga M, et al. Many young men with prostate-specific antigen (PSA) screen-detected prostate cancers may be candidates for active surveillance. BJU Int. 2013;111(6):934-940. | Wrong study design |
| 573 Wansink B, Chandon P. Meal size, not body size, explains errors in estimating the calorie content of meals. Annals of internal medicine, 2006, 145(5): 326-332. | Wrong study design |
| 574 Liu Y, Wang L, Chen L, et al. Mental Health Status of Paediatric Medical Workers in China During the COVID-19 Outbreak. Front Psychiatry. 2020;11:702. | Wrong study design |
| 575 Cuoghi OA, Sella RC, de Mendonça MR. Mesiodistal angulations of the mandibular canines, premolars and molars with or without the presence of third molars. Eur J Orthod. 2010;32(4):472-476. | Wrong study design |
| 576 Chai C, Song LJ, Han SY, et al. MicroRNA-21 promotes glioma cell proliferation and inhibits senescence and apoptosis by targeting SPRY1 via the PTEN/PI3K/AKT signaling pathway. CNS Neurosci Ther. 2018;24(5):369-380. | Wrong study design |
| 577 Gao J, Feng ST, Wu B, et al. Microstructural brain abnormalities of children of idiopathic generalized epilepsy with generalized tonic-clonic seizure: a voxel-based diffusional kurtosis imaging study. J Magn Reson Imaging. 2015;41(4):1088-1095. | Wrong study design |
| 578 Liu X, Li G, Wang J, et al. Minimally Invasive Unilateral vs. Bilateral Pedicle Screw Fixation and Lumbar Interbody Fusion in Treatment of Multi-Segment Lumbar Degenerative Disorders.Med Sci Monit. 2015;21:3652-3657. | Wrong study design |
| 579 Jendryczko A, Drózdz M. Mutacje mitochondrialnego DNA czynnikiem procesu starzenia oraz zmian degeneracyjnych. Przegl Lek. 1990;47(9):645-647. | Wrong study design |

| | |
|---|---|
| 580 Xiong W, Xu M, Zhao Y, et al. EXPRESS: A Modified Risk Assessment Score in the Prognostic Evaluation of One-year Survival Rate of Pulmonary Arterial Hypertension. Pulm Circ. 2018. | Wrong study design |
| 581 Cao L, Wang S, Zhang L, Li J. mPEG-b-P(Glu-co-Phe) nanoparticles increase gastric retention time and gastric ulcer treatment efficacy of 20(S)-ginsenoside Rg3.Biomed Pharmacother. 2022;146:112608. | Wrong study design |
| 582 Sama MT, Homeida M, Ngang P, et al. A multi-centre study of community-directed ivermectin distributors' (CDDs') involvement in other healthcare and development programme activities in Cameroon, Togo, Sudan, Nigeria and Uganda. Trop Doct. 2003;33(4):237-241. | Wrong study design |
| 583 Youssef M A, Elahwal H M, Elnashartawy H S, et al. Multi-detector computed tomography in evaluation of post-operative complications in hepatic transplantation recipients. The Egyptian Journal of Radiology and Nuclear Medicine. 2015: 823-832. | Wrong study design |
| 584 Oleg D, Starodubov, Olga A, et al. Myocardial Muscle Bridges: Pathophysiological Features and Clinical and Morphological SignsScientific News of the Belgorod State University. Series: Medicine. Pharmacy. 2016. | Wrong study design |
| 585 Li W, Ping Z, Xuemei G, et al. Naturally Occurring Sclareol Diterpene Augments the Chemosensitivity of Human Hela Cervical Cancer Cells by Inducing Mitochondrial Mediated Programmed Cell Death, S-Phase Cell Cycle Arrest and Targeting Mitogen-Activated Protein Kinase (MAPK)/Extracellular-Signal-Regulated Kinase (ERK) Signaling Pathway. Med Sci Monit. 2020;26:e920248. | Wrong study design |
| 586 Štěchovský C, Hájek P, Horváth M, et al. Near-infrared spectroscopy characterization of internal carotid artery restenosis after endarterectomy. International journal of cardiology. Heart & vasculature. 2015. | Wrong study design |
| 587 Jose TP, Oliveira S, Tavares I, et al. Neuroinflammation and neurodegeneration in the prefrontal cortex during diabetic neuropathic painDiabetologia. 2015. | Wrong study design |
| 588 Sun H, Wang C, Wang G, et al. Neuromyelitis optica spectrum disorder with common non-organ-specific autoantibodies. Radiology of infectious diseases. 2016. | Wrong study design |
| 589 Botescu SNew data demonstrate comparable glycaemic control for Levemir? and Glargine, with a unique weight benefit for Levemir?Romanian Journal of Diabetes Nutrition and Metabolic Diseases. 2008. | Wrong study design |
| 590 Hao Y, Yu HZ, Yuan HT. A New Zn(II)-containing Coordination Polymer for Photocatalytic Degradation of Organic Dyes and Treatment Activity on Atherosclerosis via Reducing the Vcam-1 Expression. J Oleo Sci. 2020;69(1):55-63. | Wrong study design |
| 591 Natalya V, Efimenko, Tatiana I, et al. Non-alcoholic fatty liver disease: modern aspects of etiopathogenesis and treatmentBulletin of Avicenna. 2016. | Wrong study design |
| 592 Y W, L X, N Z, et al. A novel DNA sensor based on C60NPs-PAMAM-PtPNPs to detect VKORC1 gene for guiding rational clinical therapy with Warfarin. Anal Chim Acta. 2019;1078:232. | Wrong study design |
| 593 Taimeh Z, Cogswell R, Duval S, et al. Novel Method of Matching Size of Donors and Heart Transplant Recipients Using Predicted Total Ventricular Mass Is Associated with Improved Survival After Cardiac Transplantation. The Journal of Heart and Lung Transplantation, 2016. | Wrong study design |

| | |
|---|---|
| 594 Jiang XF, Tian Z, Zhu SX, et al. A novel small-molecule inhibitor suppresses colon cancer metastasis through inhibition of metastasis-associated in colon cancer-1 transcription. Invest New Drugs. 2021;39(1):293. | Wrong study design |
| 595 Li Y, Yang G, Li M, et al. Nursing Observation on the Clinical Efficacy and Toxicity of Lobaplatin Compared with Cisplatin in the Treatment of Locally Advanced Hypopharyngeal Carcinoma Based on Intelligent CT Imaging. J Healthc Eng. 2021;2021:9982888. | Wrong study design |
| 596 Iacono F, Prezioso D, Illiano E, et al. Observational study: daily treatment with a new compound "tradamixina" plus serenoa repens for two months improved the lower urinary tract symptoms. BMC Surg. 2016;16(1):23. | Wrong study design |
| 597 Annear MJ. Online Education Improves Dementia Knowledge: Evidence From an International Intervention. J Appl Gerontol. 2019;38(4):NP2-NP18. | Wrong study design |
| 598 Wetzel S L, Wollenberg J. Oral Potentially Malignant Disorders. Dental Clinics of North America, 2020, 64(1): 25-37. | Wrong study design |
| 599 Ueshima H, Otake H. Pectoral nerves block for a contraction of the latissimus dorsi muscle. J Clin Anesth. 2016;31:200. | Wrong study design |
| 600 Eysenck HJ, Grossarth-Maticek R, Everitt B.Personality, Stress, Smoking, and Genetic Predisposition as Synergistic Risk Factors for Cancer and Coronary Heart Disease. Integr Psychol Behav Sci. 2021 | Wrong study design |
| 601 Wolfort RM, Manriquez R, Stokes KY, et al. Platelet-derived RANTES mediates hypercholesterolemia-induced superoxide production and endothelial dysfunction. Arterioscler Thromb Vasc Biol. 2011;31(4):e7. | Wrong study design |
| 602 Moghe S, Saini N, Moghe A. Platelet-rich plasma in periodontal defect treatment after extraction of impacted mandibular third molars. Natl J Maxillofac Surg. 2012;3(2):139-143. | Wrong study design |
| 603 Campbell DA, Field M, McArdle CS, et al. Polymorphism at the tumour necrosis factor locus: a marker of genetic predisposition to colorectal cancer?. Lancet. 1994;343(8892):293-294. | Wrong study design |
| 604 Southall M. A polyurethane–chitosan brush as an injectable hydrogel for controlled drug delivery and tissue engineering. Polymer Chemistry, 2021, 12(15): 2346-2346. | Wrong study design |
| 605 Kristina E, Fedorova, Yulia B, Khusainova. Possibilities of Application of Local-Regional Muscle Endurance and Its Efficiency in Training High Qualification SkiersInternational Scientific Research Journal. 2015. | Wrong study design |
| 606 Brinsden MD, Carr AJ, Rees JL. Post-traumatic flexion contractures of the elbow: Operative treatment via the limited lateral approach. J Orthop Surg Res. 2008;3:39. | Wrong study design |
| 607 Hou S, Bin C. Postoperative Effect Observation and Clinical Study of Dahuang Zhechong Pills from Jingui Yaolue in Treating Patients with Early-to-Mid Prostate Cancer Undergoing Radical Resection. Comput Intell Neurosci. 2022;2022(1):2998825. | Wrong study design |
| 608 Carabineanu R. The potential use of glycated hemoglobin for diabetes screening/diagnosisRomanian Journal of Diabetes Nutrition and Metabolic Diseases. 2008. | Wrong study design |
| 609 Qu S, Shan L, Zhang Z, et al. Prediction Effect of Amplitude-Integrated EEG on the Brain Damage and Long-Term Nervous System Development of Late Preterm Infants. J Healthc Eng. 2021;2021:4041082. | Wrong study design |

| | |
|---|---|
| 610 Sanada K, Miyachi M, Yamamoto K, et al. Prediction models of sarcopenia in Japanese adult men and women. Japanese Journal of Physical Fitness and Sports Medicine, 2010, 59(3): 291-302. | Wrong study design |
| 611 Vandeputte C, Krygier J, Ameye L, et al. Predictive role of body composition on survival and treatment toxicity for metastatic colorectal cancer patients. Nutrition Clinique et Métabolisme, 2016, 30(1): 62. | Wrong study design |
| 612 Xin Liu, Yvette D. Miller, Nicola W. Burton. Preliminary study of the effects of Tai Chi and Qigong medical exercise on indicators of metabolic syndrome and glycaemic control in adults with raised blood glucose levels. Br J Sports Med. 2010;44(8):608. | Wrong study design |
| 613 Roh YH, Koh YD, Kim JO, et al. Preoperative Pain Sensitization Is Associated With Postoperative Pillar Pain After Open Carpal Tunnel Release. Clin Orthop Relat Res. 2018;476(4):734-740. | Wrong study design |
| 614 Opie GM, Vosnakis E, Ridding MC, et al. Priming theta burst stimulation enhances motor cortex plasticity in young but not old adults. Brain Stimul. 2017;10(2):298-304. | Wrong study design |
| 615 Larson WL. Prism adaptation without binocular vision. Optom Vis Sci. 1990;67(3):196-200. | Wrong study design |
| 616 Al Qteishat A, Kirov K, Bokov D. The profile of the key pro-inflammatory cytokines in the serum of patients with CD and their association with the disease severity and activity. BMC Gastroenterol. 2022;22(1):477. | Wrong study design |
| 617 Yu Y, Lang QB, Chen Z, et al. Prognostic analysis of transarterial chemoembolization combined with a traditional Chinese herbal medicine formula for treatment of unresectable hepatocellular carcinoma. Chin Med J (Engl). 2009;122(17):1990-1995. | Wrong study design |
| 618 Yurttaş V, Ural A, Kutluhan A, et al. Prognostic factors for graft success in tympanoplasty with mastoidectomy. ENT Updates, 2015, 5(2): 72-75. | Wrong study design |
| 619 Grossarth-Maticek R, Eysenck HJ. Prophylactic effects of psychoanalysis on cancer-prone and coronary heart disease-prone probands, as compared with control groups and behaviour therapy groups. J Behav Ther Exp Psychiatry. 2021;73:101669. | Wrong study design |
| 620 Zhu F, Li Q, Yang Y, et al. Propofol Suppresses Proliferation, Migration, Invasion And Promotes Apoptosis By Upregulating microRNA-140-5p In Gastric Cancer Cells. Onco Targets Ther. 2019;12:10129-10138. | Wrong study design |
| 621 Tanasescu R, Ticmeanu M, Cojocaru I, et al. A prospective study of some inflammatory markers in acute stroke: correlations with short-term occurrence of new cardiovascular events. Romanian journal of neurology, 2008, 7(2). | Wrong study design |
| 622 Aradeep Chatterjee, Jaydip Biswas, Ashim Kumar Chatterjee. Psorinum Therapy in Treating Stomach, Gall Bladder, Pancreatic, and Liver Cancers: A Prospective Clinical Study. Evid Based Complement Alternat Med. 2018;2018:6803672. | Wrong study design |
| 623 Xu Y, Xu S, Cai Y, et al. Qingyihuaji Formula Inhibits Pancreatic Cancer and Prolongs Survival by Downregulating Hes-1 and Hey-1. Evid Based Complement Alternat Med. 2015;2015:145016. | Wrong study design |
| 624 Rejinold NS, Thomas RG, Muthiah M, et al. Radio frequency triggered curcumin delivery from thermo and pH responsive nanoparticles containing gold nanoparticles and its in vivo localization studies in an orthotopic breast tumor model. RSC Adv. 2020;10(48):28483. | Wrong study design |

| | |
|---|---|
| 625 Slutsky R, Watkins J, Costello D. Radionuclide evaluation of the systolic blood pressure/end-systolic volume relationship: response to pharmacologic agents in patients with coronary artery disease. Am Heart J. 1983;105(1):53-59. | Wrong study design |
| 626 Wen P, Chen S, Wang J, et al. Receiver operating characteristics (ROC) analysis for decreased disease risk and elevated treatment response to pegylated-interferon in chronic hepatitis B patients. Future generation computer systems. 2019. | Wrong study design |
| 627 Wang J, Zuo J, Wang MD, et al. Receptor tyrosine kinase AXL is correlated with poor prognosis and induces temozolomide resistance in glioblastoma. CNS Neurosci Ther. 2020;26(7):777. | Wrong study design |
| 628 Green O, Young EM, Oberman J, et al. Recruitment of pregnant women to randomised trials of COVID 19 treatments, and pharmaceutical treatments received outside such trials: A research article. Eur J Obstet Gynecol Reprod Biol. 2022;275:12-16. | Wrong study design |
| 629 Andersson L, Myhre S, Bostrom P, et al. Reduced syntaxin-5 in skeletal muscle of patients with type 2 diabetes. A link between lipid storage and insulin resistance. Atherosclerosis. 2015;239(1):288. | Wrong study design |
| 630 Yang YM, Li YH, Ding LL, et al. Regulatory effect of lncRNA NKILA on autophagy induced by sepsis kidney injury. Eur Rev Med Pharmacol Sci. 2019;23(18):8011-8017. | Wrong study design |
| 631 SĂVOIU G, NOVEANU L, Fira-Mladinescu O, et al. Relationship between brachial artery flow-mediated dilation and carotid artery intima-media thickness in the middle-aged subjects with low cardiovascular risk. Rom. J. Biophys. 2008, 18(3): 209-216. | Wrong study design |
| 632 Relationship between brachial artery flow-mediated dilation and carotid artery intima-media thickness in the middle-aged subjects with low cardiovascular risk | Wrong study design |
| 633 Camelia C, Doffoel M, Habersetzer F, et al. Relationship Between Genotypes of Hepatitis C Virus and the Progression to Cirrhosis in Chronic Hepatitis C Patients. Acta Medica Marisiensis, 2012, 58(3). | Wrong study design |
| 634 Najjarpour-Jabbari H, Nouri M, Pezeshkian M, et al. Relationship of epicardial and subcutaneous fatty acids with serum lipids and vascular cramps in patients undergoing coronary artery bypass graft. Crescent Journal of Medical and Biological Sciences. 2019, 6(2): 201-208. | Wrong study design |
| 635 GÖÇEN U, Atalay A. Repair of congenital heart defects by using the minimal right vertical infra-axillary thoracotomy in children under one year of age. International Journal of Clinical amd Experimental Medicine. 2016, 9(8). | Wrong study design |
| 636 Yin T. Research on computer technical assistance tools for special children for music therapy. Cluster Computing. 2019, 22(Suppl 2): 3605-3612. | Wrong study design |
| 637 Song ZZ. Resynchronization of the infarcted myocardium. Int J Cardiol. 2009. | Wrong study design |
| 638 Tseng C P, Chen C W, Liu K F R. Risk control allocation model for pressure vessels and piping project. Journal of Vibration and Control. 2014, 20(10): 1604-1604. | Wrong study design |
| 639 Shen H, Hu Y, Liu X, et al. Role of Cardiovascular Color Doppler Imaging Information Technology Under Artificial Intelligence Neural Network in the Diagnosis of Septic Shock Patients. Neurosci Lett. 2020. | Wrong study design |

| | |
|---|---|
| 640 Ke L, Li J, Chen X, et al. Role of Color Doppler Imaging in Diagnosis of Carotid Atherosclerosis and Nerve Damage in Patients with Bacterial Infection. Neurosci Lett. 2020. | Wrong study design |
| 641 Khang G, Kim S H, Kim M S, et al. Role of cytokines for the treatment of intervertebral disc using regenerative medicine. Tissue Eng Regen Med. 2007, 4(4): 490. | Wrong study design |
| 642 Zhang A, Wang J, Jing Q. Role of Folic Acid Drugs in the Treatment with Antithrombotic and Anticoagulant Drugs for Patients with Cardiovascular Diseases Based on the Analysis of Virtual Reality Medical Data. J Healthc Eng. 2021;2021:9914787. | Wrong study design |
| 643 Paventi S, Parafati MA, Di Luzio E, et al. Safety and feasibility of two-dimensional echocardiography and myocardial perfusion imaging in patients with chest pain. Angiology. 2001;52(5):305-309. | Wrong study design |
| 644 Acharya S, Mandal PK. Salivary IgA and dental caries in HIV patients: A pilot study. J Indian Soc Pedod Prev Dent. 2016;34(4):341-347. | Wrong study design |
| 645 Wu L, Hu A, Tam N, et al. Salvage liver transplantation for patients with recurrent hepatocellular carcinoma after curative resection. PLoS One. 2012;7(7):e41820. | Wrong study design |
| 646 Sacchidanand S, Purohit V. Sclerotherapy for the treatment of pyogenic granuloma. Indian J Dermatol. 2013;58(1):77-78. | Wrong study design |
| 647 Xiang Q, Liang Y, Chen B. Serum Fluoride Level and Children's Intelligence Quotient in Two Villages in China. Environ Health Perspect. 2010. | Wrong study design |
| 648 Wu Y, Wan X, Ji F, et al. Serum miR-658 induces metastasis of gastric cancer by activating PAX3-MET pathway: A population-based study. Cancer Biomark. 2018;22(1):111-118. | Wrong study design |
| 649 Dogan S. Severe contact dermatitis due to camomile: a common complementary remedy with potential sensitization risks. Allergy, asthma, and clinical immunology: official journal of the Canadian Society of Allergy and Clinical Immunology, 2013, 9(1): 28. | Wrong study design |
| 650 Rogers RG, Kammerer-Doak D, Darrow A, et al. Sexual function after surgery for stress urinary incontinence and/or pelvic organ prolapse: a multicenter prospective study. Am J Obstet Gynecol. 2004;191(1):206-210. | Wrong study design |
| 651 Schultz W C M W, Van de Wiel H B M, Hahn D E E, et al. Sexuality and cancer in women. Annual Review of Sex Research, 1992, 3(1): 151-200.doi:10.1080/10532528.1992.10559878 | Wrong study design |
| 652 Vuorinen AL, Strahilevitz MA, Wansink B, et al. Shifts in the Enjoyment of Healthy and Unhealthy Behaviors Affect Short- and Long-Term Postbariatric Weight Loss†. Bariatr Surg Pract Patient Care. 2017;12(1):35-42. | Wrong study design |
| 653 Dobreanu M, Dobreanu D, Gălăţeanu C, et al. Short term effects of atorvastatin on leucocytes integrin expression in unstable angina. Revista Română de Medicină de Laborator Vol, 2006, 2(1). | Wrong study design |
| 654 Zhou D, Jiang X, Ding W, et al. siRNA-participated chemotherapy: an efficient and specific therapeutic against gastric cance. J Cancer Res Clin Oncol. 2015;141(11):2069. | Wrong study design |
| 655 Ajit Vigg, Ajit Vigg. Sleep in Type 2 diabetes. 2003 | Wrong study design |

| | |
|---|---|
| 656 Spiroski M. Sleep Organisation in Depression and Schizophrenia: Index of Endogenous Periodicity of Sleep as a State Marker. Open Access Macedonian Journal of Medical Sciences. 2014, 2(2): 319-319. | Wrong study design |
| 657 Kufner A, Ali HF, Ebinger M, et al. The smoking paradox in ischemic stroke patients treated with intra-arterial thrombolysis in combination with mechanical thrombectomy-VISTA-Endovascular. PLoS One. 2021;16(5):e0251888. | Wrong study design |
| 658 Han W, Du X, Wang J, et al. SNHG16 indicates a poor prognosis and affects cell proliferation, migration and invasion in non-small cell lung cancer. Exp Cell Res. | Wrong study design |
| 659 Barbosa G M, Dantas G A F, Souza B R, et al. Static stretching program decrease the eccentric peak torque without changing the functional perfomance in healthy adults: A Randomized controlled trial. Physical Therapy in Sport. 2020, 45: e2. | Wrong study design |
| 660 Alruwaili NK, Zafar A, Imam SS, et al. Stimulus Responsive Ocular Gentamycin-Ferrying Chitosan Nanoparticles Hydrogel: Formulation Optimization, Ocular Safety and Antibacterial Assessment. Int J Nanomedicine. 2020;15:4717-4737. | Wrong study design |
| 661 Shukla A, Kumar B. A Study of Otorhinolaryngological Diseases with Ophthalmic Complications: At a Tertiary Care Center. Indian J Otolaryngol Head Neck Surg. 2022;74(Suppl 2):1820-1825. | Wrong study design |
| 662 Meng X, Liu W, Zhang H. A Study of Rock Burst Hazard Prevention Method. Geotechnical and Geological Engineering. 2018, 36: 2237-2246. | Wrong study design |
| 663 Zukauskas G, Ruksenas O, Burba B, et al. A study of stress affecting police officers in Lithuania. Int J Emerg Ment Health. 2009;11(4):205-214. | Wrong study design |
| 664 Wang P, Liu C, Wei L, et al. A Study on Risk Factors Associated with Reflux Esophagitis in Patients Undergoing Esophageal Cancer Surgery. J Healthc Eng. 2022;2022:3409693. | Wrong study design |
| 665 Zhou G, Kong X. Study on the Effect of Combination of Prednisone and Vitamin D in the Treatment of Primary Nephrotic Syndrome in Children. J Healthc Eng. 2021;2021:7932721. | Wrong study design |
| 666 Liu J, Li C, Wang Q, et al. Study on Toll-Like Receptor 2-Mediated Inflammation-Induced Familial Hypertension Combined with Hyperlipemia and Its Mechanism. J Healthc Eng. 2022;2022:1473597. | Wrong study design |
| 667 Zhu Y, Sheng Y. Sustained delivery of epalrestat to the retina using PEGylated solid lipid nanoparticles laden contact lens. Int J Pharm. 2020;587:119688. | Wrong study design |
| 668 Alhelih E, Ghazi Baker O, Aboshaiqah AE. Symptom trajectories and occurrence in older Saudi children with cancer during a course of chemotherapy. Eur J Cancer Care (Engl). 2017;26(4):e12555. | Wrong study design |
| 669 Verma A. Synthesis and antibacterial activity of chitosan–ciprofloxacin prodrug conjugates. Medicinal Chemistry Research, 2015, 24: 901-901. | Wrong study design |
| 670 Keri R S, Hosamani K M, Shingalapur R V. Shingalapur. Synthesis, anti-bacterial, anti-fungal and cytotoxic properties of novel pyrimidine derivatives from chromen-2-one moiety. Medicinal chemistry research. 2010;23: 3927-3927. | Wrong study design |
| 671 Xu B, Dong F Q, Yan H J. Synthesis, crystal structure, and antigastric cancer effect of a novel molybdoarsenate compound. Inorganic and Nano-Metal Chemistry. 2017, 47(12): 1686-1690. | Wrong study design |

| | |
|---|---|
| 672 Napoleão P, Selas M, Freixo C, et al. T lymphocytes alterations are associated with oxidized LDL, troponin T, white blood cells and C-reactive protein during acute myocardial infarction. Clin Hemorheol Microcirc. 2014;56(1):57-66. | Wrong study design |
| 673 Gualtieri CT, Breuning SE, Schroeder SR, et al. Tardive dyskinesia in mentally retarded children, adolescents, and young adults: North Carolina and Michigan studies. Psychopharmacol Bull. 1982;18(1):62-65. | Wrong study design |
| 674 Noah C, Michelle G, Nicole W, et al. Teaching electronic medical record (EMR) data discipline to clinical trainees: A Canadian pilot study. Int J Med Inform. 2022;159:104664. | Wrong study design |
| 675 Becker-Weidman A. Treatment for Children with Reactive Attachment Disorder: Dyadic Developmental Psychotherapy. Child Adolesc Ment Health. 2008;13(1):52. | Wrong study design |
| 676 Gaffo A, Saag K G, Curtis J R. Treatment of rheumatoid arthritis. American journal of health-system pharmacy, 2006, 63(24): 2451-2465.doi:10.1002/anr.1790020311 | Wrong study design |
| 677 Yu H B, Chen F J, Li J, et al. Two new Cd (II)/Co (II) compounds: Luminescent and photocatalytic property, and treatment activity on coronary artery atherosclerosis. Arabian journal of chemistry. 2022. | Wrong study design |
| 678 Zhang J M, Zeng M L, Liang X X, et al. Two transition metal coordination polymers: Selective gas sorption and treatment activity in combination with Latanoprost on primary open-angle glaucoma. Journal of Solid State Chemistry. 2021. | Wrong study design |
| 679 Suba EJ, Ortega RE, Mutch DG. Unethical randomised controlled trial of cervical screening in India: US Freedom of Information Act disclosures. BMJ Glob Health. 2017;2(2):e000177. | Wrong study design |
| 680 Ishikawa Y, Ueshima H. Use of a cardiopulmonary resuscitation simulator for objective evaluation of the quality of cardiopulmonary resuscitation objectively. J Clin Anesth. 2020;60:19-20. | Wrong study design |
| 681 Šín P, Holoubek J, Hokynková A,et al. The use of ante-and retrograde flow in microsurgical mandibular reconstruction with use of the 3D surgical modeling technique and cutting guide application. Microsurgery. 2020 Feb 14;40(3):414-6. | Wrong study design |
| 682 Elisabet Esquivel-Prados, Elisa Pareja-Martinez, Joanna C Muollin, et al. Validity and reliability of a Spanish version of the Morisky Medication Adherence Scale (MMAS-8) questionnaire in patients with type 2 diabetes mellitus treated with oral antidiabeticsAtencion Primaria. 2018. | Wrong study design |
| 683 Lin S, Guo Y. Value Analysis of Using Urinary Microalbumin in Artificial Intelligence Medical Institutions to Detect Early Renal Damage in Diabete. J Healthc Eng. 2021;2021:6678454. | Wrong study design |
| 684 Alipio, M.M. Vitamin D Supplementation Could Possibly Improve Clinical Outcomes of Patients Infected with Coronavirus-2019 (COVID-2019).Social Science Research Network. 2020. | Wrong study design |
| 685 Gurkan N. Vitamin D supplementation during pregnancy inhibits the activation of fetal membrane NF-κB pathway. Eur Rev Med Pharmacol Sci. 2022;26(16):5926-5931. | Wrong study design |
| 686 Tanaka K, Yamada M, Hatoh T, et al. "Whirl sign": small bowel volvulus in patients after gastrectomy. Am Surg. 2012;78(11):E452-E453. | Wrong study design |
| 687 Liu X, Li Y, Ma Q, et al. Withaferin-A Inhibits Growth of Drug-Resistant Breast Carcinoma by Inducing Apoptosis and Autophagy, Endogenous Reactive Oxygen Species (ROS) Production, and Inhibition of Cell Migration and Nuclear Factor | Wrong study design |

| | |
|---|---|
| kappa B (Nf-κB)/Mammalian Target of Rapamycin (m-TOR) Signalling Pathway. Medical Science Monitor: International Medical Journal of Experimental and Clinical Research. 2021;27:e932348-1 | |
| 688 Hussain A, Gopalakrishnan A, Muthuvel B, et al. Young adults with myopia have lower concentrations of neuromodulators-dopamine and melatonin in serum and tear. Exp Eye Res. 2021;209:108684. | Wrong study design |
| 689 Hammad M, Kestutis R, Virgilius T, et al. Advantages and disadvantages of artificial heart valvesScience Technology and Education. 2015. | Wrong publication type |
| 690 Pesenti F B, da Silva R A, da Silva L A, et al. Cold water immersion effects on doms, muscle recruitment, dynamic postural control and sleep quality in soccer players: A randomized and blinded study. Physical Therapy in Sport.2020, 45: e4. | Wrong publication type |
| 691 Delye H. Craniosynostosis: Endoscopically Versus Open Treatment. Evidence for Neurosurgery: Effective Procedures and Treatment. 2019:127-43. | Wrong publication type |
| 692 Jia F, Xue Y, Liu K, et al. Effects of total parathyroidectomy treatment on parathyroid hormone levels, recurrent laryngeal nerve function, and the rate of infection complications of secondary hyperparathyroidism patients under image information health monitoring by magnetic resonance imaging. Neurosci Lett. 2020;735:135195. | Wrong publication type |
| 693 Elias D, Avron A, Tamir M, et al. Evaluation of DiaPep277 (R) treatment in type 1 diabetes by integrated analysis.DIABETOLOGIA. 2014, 57: S190-S191. | Wrong publication type |
| 694 Larisa A, Knyazeva, Larisa I, et al. Indicators of Local and Regional Arterial Rigidity in Patients with Rheumatoid ArthritisInternational Journal of Applied and Fundamental Research. 2016. | Wrong publication type |
| 695 Chaves S F, de Menezes F S, Pereira A L A, et al. Instrument assisted soft tissue mobilization decreases pain intensity and fatigue perception after long-distance ironman triathlon. Physical Therapy in Sport, 2020, 45: e5. | Wrong publication type |
| 696 Bansal P, Gupta A, Mongha R, et al. Laparoscopic versus open pyeloplasty: Comparison of two surgical approaches -- a single centre experience of three years. J Minim Access Surg. 2008;4(3):76-79. | Wrong publication type |
| 697 de Souza F A, de Sousa N T A, de Oliveira V A V, et al. Neuromuscular electrical stimulation during blood flow restriction promotes altered muscle electrical activity and improves balance in high-performance basketball athletes. Blind randomized clinical trial. Physical Therapy in Sport, 2020, 45: e3-e4. | Wrong publication type |
| 698 Feng Y, Han Z, Gu B, et al. A Novel Method for the Prevention and Treatment of Small-for-Size Syndrome in Liver Transplantation. Dig Dis Sci. 2020;65(9):2619-2629. | Wrong publication type |
| 699 Andrew Chua, Vishal Patel, Allison Perrin, et al. Number needed to treat with 4-factor prothrombin complex concentrate for urgent warfarin reversal. Am J Hematol. 2017;92(4):E60. | Wrong publication type |
| 700 Zhang X, Mo R, Zhao H, et al. A comparative effectiveness meta-analysis of photodynamic therapy and stent drainage for unresectable cholangiocarcinoma. Photodiagnosis Photodyn Ther. 2018. | Wrong publication type |
| 701 Rini BI. VEGF-targeted therapy in metastatic renal cell carcinoma. The oncologist. 2005 ;10(3):191-7. | Wrong publication type |
| 702 Lee J, Kim EH, Shin D, et al. Accelerated oral wound healing using a pre-vascularized mucosal cell sheet. Sci Rep. 2017;7(1):10667. | Wrong population |

| | |
|---|---|
| 703 Gadde UD, Oh S, Lillehoj HS, et al. Antibiotic growth promoters virginiamycin and bacitracin methylene disalicylate alter the chicken intestinal metabolome. Sci Rep. 2018;8(1):3592. | Wrong population |
| 704 Kulp SK, Chen CS, Wang DS, et al. Antitumor Effects of a Novel Phenylbutyrate-based Histone Deacetylase Inhibitor, (S)-HDAC-42, in Prostate Cancer. Clin Cancer Res. 2019;25(9):2940. | Wrong population |
| 705 Badr MO, Hashem MA, Gado NN. Comparative biochemical studies on steroidogenic compounds in chickens. Res Vet Sci. 2010;89(2):168-173. | Wrong population |
| 706 Meghashri S, Chauhan J B, Zameer F. Contribution of herbal principles towards cytoprotective, antioxidant and anti-Rhizopus activities. 2012: 29-33. | Wrong population |
| 707 Kaya A, Yörük M A, Esenbuga N, et al.The effect of raw and processed common vetch seed (Vicia sativa) added to diets of laying hens on performance, egg quality, blood parameters and liver histopathology. The Journal of Poultry Science, 2013, 50(3): 228-236. | Wrong population |
| 708 Qian W, Xiaoyi W, Zi Y, et al. Effects of Yin-nourishing and blood-cooling decoction on proteinuria and Renal Tubular damage in IgA Nephropathy. Future generation computer systems.2019:682-687. | Wrong population |
| 709 Aisilahong G, Maimaiti P, Abulimiti A, et al. Establishment and evaluation of contracture model of knee joint. 2020: 1152-1152. | Wrong population |
| 710 Hattori S, Hattori Y, Kasai K. Hypoadiponectinemia is caused by chronic blockade of nitric oxide synthesis in rats.Metabolism. 2005;54(4):482-487. | Wrong population |
| 711 Lin Z, Zhang C, Zhang X, et al. Improving Small Intestinal Motility in Experimental Acute Necrotising Pancreatitis by Modulating the CPI-17/MLCP Pathway Using Chaiqin Chengqi Decoction.Evid Based Complement Alternat Med. 2020:9189457. | Wrong population |
| 712 Mao SC, Chang CH, Wu CC, et al. Inhibition of spontaneous recovery of fear by mGluR5 after prolonged extinction training. PloS one. 2013;8(3):e59580. | Wrong population |
| 713 Foreman TW, Bucşan AN, Mehra S, et al. Isoniazid and Rifapentine Treatment Eradicates Persistent Mycobacterium tuberculosis in Macaques . Am J Respir Crit Care Med. 201(4):469-477. | Wrong population |
| 714 Saraf SK, Singh RP, Singh V, et al. Pullout strength of misplaced pedicle screws in the thoracic and lumbar vertebrae - A cadaveric study. Indian J Orthop. 2013;47(3):238-243. | Wrong population |
| 715 Wei Z. Research on the Effect of Puerarin on Alleviating Sports Fatigue. Open Biomed Eng J. 2015;9:288-291. | Wrong population |
| 716 Lim C H, Sun K, Son H S, et al. A study of optimal model for the circuit configuration of Korean pulsatile extracorporeal life support system (T-PLS). The Korean Journal of Thoracic and Cardiovascular Surgery. 2005: 661-668. | Wrong population |
| 717 Compare A, Kouloulias V, Apostolos V, et al. WELL. ME-Wellbeing therapy based on real-time personalized mobile architecture, vs. cognitive therapy, to reduce psychological distress and promote healthy lifestyle in cardiovascular disease patients: study protocol for a randomized controlled trial . Trials. 2018. | Wrong publication type |

| | |
|---|---|
| 718 Mao Y, Qin ZH. Association of apneic oxygenation with decreased desaturation rates during rapid sequence intubation by a Chinese emergency medicine service. Int J Clin Exp Med. 2015;8(7):11428-11434. | Wrong study design |
| 719 Saccomanni B. Early outcome of arthroscopic Bankart's repair for recurrent traumatic anterior shoulder instability. J Clin Orthop Trauma. 2013;4(3):129-134. | Wrong study design |
| 720 Bhattacharyya T, Sharma SC, Yadav BS,et al. Outcome of neoadjuvant chemotherapy in locally advanced breast cancer: A tertiary care centre experience. Indian J Med Paediatr Oncol. 2014;35(3):215-220. | Wrong study design |
| 721 Yang L, Yu C, Lan Z. Autogenous regulation effect of long-time different load exercise on female endocrine hormone. The International Journal of Electrical Engineering & Education. 2019, 56(4): 315-326. | Wrong population |
| 722 Irie H, Shiraishi J, Sawada T, et al. Cardio-cerebrovascular protective effects of valsartan in high-risk hypertensive patients with overweight/obesity: A post-hoc analysis of the KYOTO HEART Study. Int J Cardiol. 2012. | Presudo randomization |
| 723 Arsenault BJ, Barter P, DeMicco DA, et al. Prediction of cardiovascular events in statin-treated stable coronary patients of the treating to new targets randomized controlled trial by lipid and non-lipid biomarkers. PLoS One. 2014;9(12):e114519. | Presudo randomization |
| 724 Yoshida H, Shimizu M, Ikewaki K, et al. Sex differences in effects of valsartan administration on cardiovascular outcomes in hypertensive patients: findings from the Jikei Heart Study. Journal of hypertension, 2010, 28(6): 1150-1157. | Presudo randomization |
| 725 Iwamoto J, Takeda T, Sato Y, et al. Effects of alendronate on metacarpal and lumbar bone mineral density, bone resorption, and chronic back pain in postmenopausal women with osteoporosis. Clin Rheumatol. 2004;23(5):383-389. | Presudo randomization |
| 726 Shah SR, Shah SA, Jangda MA, et al. Topical vasodilator response in skeletonized internal mammary artery: Is there really a difference?. Avicenna J Med. 2017;7(1):23-27. | Presudo randomization |
| 727 Ivandic BT, Ivandic T. Effects of Photobiomodulation Therapy on Patients with Primary Open Angle Glaucoma: A Pilot Study. Photomed Laser Surg. 2015;10.1089/pho.2015.3944. | Presudo randomization |
| 728 Yang J, Wang N, Tong X, et al. Impact of guidewire selection and operator expertise on radiation exposure in transradial angiography. J Cardiothorac Surg. 2014;9(1):194. | Presudo randomization |
| 729 Vinod A, St B, Nanda NS,et al. Comparison of Two Stump Closure Techniques in Laparoscopic Appendicectomy: A Single-Centre Prospective Cohort Study. Cureus. 2022;14(1):e21796. | Presudo randomization |
| 730 Xu Q, Wei YT, Fan SB, et al. Early hyperbaric oxygen therapy may improve the long term neurological consequences of diabetic patients suffering from hemorrhagic stroke. Neurosci Lett. 2017;644:83-86. | Presudo randomization |
| 731 Sajovic M, Strahovnik A, Komadina R, et al. The effect of graft choice on functional outcome in anterior cruciate ligament reconstruction.Int Orthop. 2008;32(4):473-478. | Presudo randomization |
| 732 Hu B, Ren G, Zhao L. Effect of Health Education Combined with Dietary Guidance on Nutritional Indicator, Immune Level, and Quality of Life of Patients with Pulmonary Tuberculosis. Comput Math Methods Med. 2021:9463577. | Presudo randomization |
| 733 Ohtake M, Morikagi Y, Suzuki I, et al. Effects of exercise on the prevention of conditions leading to the need for long-term care. Aging Clin Exp Res. 2013;25(1):49-57. | Presudo randomization |

| | |
|---|---|
| 734 Suttner SW, Boldt J, Schmidt CC, et al. The effects of sodium nitroprusside-induced hypotension on splanchnic perfusion and hepatocellular integrit.Anesth Analg. 1999;89(6):1371-1377. | Presudo randomization |
| 735 Finelli C, Crispino P, Gioia S, et al. The improvement of large High-Density Lipoprotein (HDL) particle levels, and presumably HDL metabolism, depend on effects of low-carbohydrate diet and weight loss. EXCLI J. 2016;15:166-176. | Presudo randomization |
| 736 Zhang CJ, Deng YZ, Lei YH, et al. The mechanism of exogenous adiponectin in the prevention of no-reflow phenomenon in type 2 diabetic patients with acute myocardial infarction during PCI treatment. Eur Rev Med Pharmacol Sci. 2018;22(7):2169-2174. | Presudo randomization |
| 737 Suzuki T, Kurazumi T, Ueda T, et al. Desflurane anesthesia worsens emergence agitation in adult patients undergoing thyroid surgery compared to sevoflurane anesthesia. JA Clin Rep. 2017;3(1):36. | Presudo randomization |
| 738 Boyle R, Brown N, Chiang WC, et al. Partially hydrolysed, prebiotic supplemented whey formula for the prevention of allergic manifestations in high risk infants: a multicentre double-blind randomised controlled trial. Clin Transl Allergy. 2015; 5(Suppl 3): P30. | Wrong publication type |
| 739 Bernard GC, Eric M, Jean ML, et al. First Double-Blind Placebo Controlled, Multi-Centre, Randomized Trial Of Sulforaphane In Men With Rising PSA Following Radical Prostatectomy. the journal of urology, 191(4s), p. e809 | Wrong publication type |
| 740 Velásquez-Rimachi, Victor, et al. Greater occipital nerve block for chronic migraine patients: A meta-analysis.Acta Neurologica Scandinavica 146.2 (2022): 101-114. | Wrong study design |
| 741 Nagy HI, Elkadi HW. Can sugammadex improve the reversal profile of Atracurium under Sevoflurane anesthesia?. Egyptian Journal of Anaesthesia. 2014;30(1):95-9. | No mention of randomisation in method |
| 742 Yuan B, Yuan M. Changes of Mental State and Serum Prolactin Levels in Patients with Schizophrenia and Depression after Receiving the Combination Therapy of Amisulpride and Chloroprothixol Tablets . Comput Math Methods Med. 2022:6580030. | No mention of randomisation in method |
| 743 Wang X, Wang C, Qi R. Effectiveness and Prognosis: Drainage Skin-Bridge Sparing Surgery Combined with Fistulotomy versus Fistulotomy Only in the Treatment of Anal Fistula. J Healthc Eng. 2021:6940072. | No mention of randomisation in method |
| 744 Chen X, Sheng D, Kong X. The Efficacy of Hydroxychloroquine Combined with Huangqi Tablets in the Treatment of Diabetic Nephropathy.J Healthc Eng. 2021:7988924. | No mention of randomisation in method |
| 745 Li Z, Chen H, Zhao S, et al. Impact of Cluster Nursing on Nursing on VAS Score and Urinary Function of Patients after Percutaneous Nephrolithotomy with Pneumatic Lithotripsy (PCNL).Appl Bionics Biomech. 2022:3743640. | No mention of randomisation in method |
| 746 Wang Z, Xu X. The Value of Combined Application of Oxycodone Hydrochloride Injection and Dexmedetomidine in Anesthesia for LC for Patients with Gallbladder Lesions. Journal of Healthcare Engineering, 2021, : 1290650. | No mention of randomisation in method |
| 747 Lin X, Zhou N, Huang X, et al. Anterior maxillary segmental distraction osteogenesis for treatment of maxillary hypoplasia in patients with repaired cleft palate. Journal of Craniofacial Surgery. 2018;29(5):e480-4. | Not retracted |
| 748 Shamas IU, Beigh Z, Ahmad S,et al. A short-term evaluation between the result of palisade cartilage tympanoplasty and temporalis fascia technique. Indian Journal of Otology. 2014 ;20(2):63-6. | Not retracted |

| | |
|---|---|
| 749 Jafari F, Mobasheri M, Mirzaeian R. Effect of diet education on blood pressure changes and interdialytic weight in hemodialysis patients admitted in hajar hospital in shahrekord. Mater Sociomed. 2014;26(4):228-230. | Not retracted |
| 750 Yang Y, Gao SG, Zhang Fjet al. Effects of osteopontin on the expression of IL-6 and IL-8 inflammatory factors in human knee osteoarthritis chondrocytes. Eur Rev Med Pharmacol Sci. 2014;18(23):3580-3586. | Not retracted |
| 751 Baker SG, Sargent DJ. Designing a randomized clinical trial to evaluate personalized medicine: a new approach based on risk prediction. J Natl Cancer Inst. 2010;102(23):1756-1759. | Not retracted |
| 752 Chawda R, Soni R, Kantesaria M, et al. Fixation of basicervical and related fractures using DHS with DRS. 2014. | Not retracted |
| 753 Cojocaru M, Chicoş B. Genetic differences between patients with rheumatoid arthritis. Rom J Intern Med. 2013;51(2):89-91. | Not retracted |
| 754 Bour F, Grezard C, Cheminon M,et al. Medication adherence of hospitalized Parkinson's patients included in an intensive rehabilitation program.2019: E1-E1. | Not retracted |
| 755 Boldt J, Menges T, Wollbrück M, et al. Platelet function in critically ill patients. Chest. 1994;106(3):899-903. | Non randomized controlled trials |
| 756 El Refaeey AEA, Abdelfattah H, Mosbah A, et al. Is early intervention using Mansoura-VV uterine compression sutures an effective procedure in the management of primary atonic postpartum hemorrhage? : a prospective study. BMC Pregnancy Childbirth. 2017;17(1):160. | Non randomized controlled trials |
| 757 Konst, Matthew J, Johnny LM. Temporal and diagnostic influences on the expression of comorbid psychopathology symptoms in infants and toddlers with Autism Spectrum Disorder. Research in Autism Spectrum Disorders 3.8 (2014): 200-208. | Non randomized controlled trials |
| 758 Chockalingam A, Chan A, Kanaley JA, et al. Home-Based Arm Cardiac Rehabilitation in Disabled Veterans: A Pilot Study. Mo Med. 2021;118(4):387-392. | Non randomized controlled trials |
| 759 Poling A, Breuning SE. Effects of methylphenidate on the fixed-ratio performance of mentally retarded children. Pharmacol Biochem Behav. 1983;18(4):541-544. | Non randomized controlled trials |
| 760 Abd-Elsalam S, Sharaf-Eldin M, Soliman S,et al. Efficacy and safety of sofosbuvir plus ribavirin for treatment of cirrhotic patients with genotype 4 hepatitis C virus in real-life clinical practice. Arch Virol. 2018;163(1):51-56. | Non randomized controlled trials |
| 761 Rezk M, Sayyed T, Masood A,et al. Risk of bacterial vaginosis, Trichomonas vaginalis and Candida albicans infection among new users of combined hormonal contraception vs LNG-IUS. Eur J Contracept Reprod Health Care. 2017;22(5):344-348. | Non randomized controlled trials |
| 762 Rezk M, Al-Halaby A, Emarh M, et al. Correlation between uterine artery Doppler indices and menstrual irregularities among levonorgestrel releasing intrauterine system and depot medroxyprogesterone acetate users: a prospective observational study. Eur J Contracept Reprod Health Care. 2017;22(4):316-320. | Non randomized controlled trials |
| 763 Lemire CA, Seto B, Yamada K, et al. Normobaric hyperoxia rapidly reduces diabetic macular oedema. Clin Exp Ophthalmol. 2021;49(7):759-761. | Non randomized controlled trials |

| | |
|---|---|
| 764 Shokeir T, Abdelshaheed M. Sonohysterography as a first-line evaluation for uterine abnormalities in women with recurrent failed in vitro fertilization-embryo transfer. Fertil Steril. 2009;91(4 Suppl):1321-1322. | Non randomized controlled trials |
| 765 Boldt J, Knothe C, Zickmann B, et al. The effects of preoperative aspirin therapy on platelet function in cardiac surgery. Eur J Cardiothorac Surg. 1992;6(11):598-602. | Non randomized controlled trials |
| 766 Xu F, Yang J, Xu B, et al. Clinical Research on Systemic Chemotherapy Combined With Bronchoscopic Seed Implantation in the Treatment of Advanced Lung Cancer. Technol Cancer Res Treat. 2020;19:1533033820971600. | Non randomized controlled trials |
| 767 Riordan NH, Hincapié ML, Morales I, et al. Allogeneic Human Umbilical Cord Mesenchymal Stem Cells for the Treatment of Autism Spectrum Disorder in Children: Safety Profile and Effect on Cytokine Levels. Stem Cells Transl Med. 2019;8(10):1008-1016. | Non randomized controlled trials |
| 768 Jeong O, Park YK, Jung MR, et al. Compliance with Guidelines of Enhanced Recovery After Surgery in Elderly Patients Undergoing Gastrectomy. World J Surg. 2017;41(4):1040-1046. | Non randomized controlled trials |
| 769 HaraE, Ueshima H, Tanaka N , Otake H. Pectoral Nerves (PECS) Block Is Effective for Motor Function Recovery in the Early Postoperative Period after Breast Cancer Surgery. Open J Anesthesiol, 7, 351-5. | Non randomized controlled trials |
| 770 Iwamoto J, Takeda T, Sato Y, et al. Comparison of the effect of alendronate on lumbar bone mineral density and bone turnover in men and postmenopausal women with osteoporosis. Clin Rheumatol. 2007;26(2):161-167. | Non randomized controlled trials |
| 771 Connolly RM, Leal JP, Solnes L, et al. TBCRC026: Phase II Trial Correlating Standardized Uptake Value With Pathologic Complete Response to Pertuzumab and Trastuzumab in Breast Cancer. J Clin Oncol. 2019;37(9):714-722. | Non randomized controlled trials |
| 772 Bansal H, Comella K, Leon J, et al. Intra-articular injection in the knee of adipose derived stromal cells (stromal vascular fraction) and platelet rich plasma for osteoarthritis. J Transl Med. 2017;15(1):141. | Non randomized controlled trials |
| 773 Jayaweera JAAS, Reyes M. Antimicrobial misuse in pediatric urinary tract infections: recurrences and renal scarring. Ann Clin Microbiol Antimicrob. 2018;17(1):27. | Non randomized controlled trials |
| 774 Boldt J, Kling D, Dapper F, et al. Myocardial temperature during cardiac operations: influence on right ventricular function. J Thorac Cardiovasc Surg. 1990;100(4):562-568. | Non randomized controlled trials |
| 775 Boldt J, Kumle B, Papsdorf M,et al. Are circulating adhesion molecules specifically changed in cardiac surgical patients?. Ann Thorac Surg. 1998;65(3):608-614. | Non randomized controlled trials |
| 776 Boldt J, Knothe C, Schindler E, et al. Thrombomodulin in pediatric cardiac surgery. Ann Thorac Surg. 1994;57(6):1584-1589. | Non randomized controlled trials |
| 777 Iwamoto J, Uzawa M, Sato Y,et al. Effect of alendronate on bone mineral density and bone turnovermarkers in post-gastrectomy osteoporotic patients. J Bone Miner Metab. 2021;39(3):521. | Non randomized controlled trials |
| 778 Boldt J, Wollbrück M, Menges T,et al. Changes in regulators of circulation in patients undergoing continuous pump-driven veno-venous hemofiltration. Shock. 1994;2(3):157-163. | Non randomized controlled trials |
| 779 Wei XF, Srivastava A, Lin P, et al. Neoadjuvant chemotherapy as a comprehensive treatment in patients with laryngeal and hypopharyngeal carcinoma. Acta Otolaryngol. 2020;140(7):603-609. | Non randomized controlled trials |

| | |
|---|---|
| 780 Roh YH, Noh JH, Gong HS, et al. Comparative study on the effectiveness of a corticosteroid injection for carpal tunnel syndrome in patients with and without Raynaud's phenomenon. Bone Joint J. 2017;99-B(12):1637-1642. | Non randomized controlled trials |
| 781 Boldt J, Knothe C, Zickmann B, et al. Does correction of acidosis influence microcirculatory blood flow during cardiopulmonary bypass?. Br J Anaesth. 1993;71(2):277-281. | Non randomized controlled trials |
| 782 Boldt J, Schindler E, Knothe C, et al. Endothelial-related coagulation in cardiac surgery. Br J Anaesth. 1995;74(2):174-179. | Non randomized controlled trials |
| 783 Mizuno T, Sakakibara T, Kasai Y. Three-Minutes Sitting Test for evaluating lumbar foraminal stenosis: A preliminary report. J Spinal Cord Med. 2018. | Non randomized controlled trials |
| 784 Cai Q, Yang M, Liu D, et al. Experimental Treatment with Favipiravir for COVID-19: An Open-Label Control Study. Engineering (Beijing). 2020;6(10):1192-1198. | Non randomized controlled trials |
| 785 Maimaiti, Palida, et al. Statistical analysis with Kruskal Wallis test for patients with joint contracture . 2020: 1153-1153. | Non randomized controlled trials |
| 786 Ling Q, Xu X, Wei Q, et al. Downgrading MELD improves the outcomes after liver transplantation in patients with acute-on-chronic hepatitis B liver failure. PLoS One. 2012;7(1):e30322. | Non randomized controlled trials |
| 787 Huang, HT,Chong J Z. Clinical outcome of patients with Type II gastroesophageal junction adenocarcinoma (GEJA) undergoing radical resection transthoracic or transhiatal. 2019: 125-125. | Non randomized controlled trials |
| 788 Nakajima I, Noda T, Kanzaki H,et al. effects of cardiac resynchronization therapy in patients with inotrope-dependent class IV end-stage heart failure. Journal of Arrhythmia. 2013;29(6):342-6. | Non randomized controlled trials |
| 789 Lee AY, Kim YH. Comparison of Movement of the Upper Dentition According to Anchorage Method: Orthodontic Mini-Implant versus Conventional Anchorage Reinforcement in Class I Malocclusion. ISRN Dent. 2011:321206. | Non randomized controlled trials |
| 790 Saitoh Y, Aoki K, Okazaki M,et al. Reversal of vecuronium with neostigmine: a comparison between male and female patients. Fukushima J Med Sci. 2009;55(2):61-70. | Non randomized controlled trials |
| 791 Qin H, Wu H, Chen Y,et al. Early Detection of Postoperative Acute Kidney Injury in Acute Stanford Type A Aortic Dissection With Doppler Renal Resistive Index. J Ultrasound Med. 2017;36(10):2105-2111. | Non randomized controlled trials |
| 792 Zhang Y, Zhu X, Liu D, et al. Pre-treatment DWI as a predictor of overall survival in locally advanced pancreatic cancer treated with Cyberknife radiotherapy and sequential S-1 therapy. Cancer Imaging. 2018;18(1):6. | Non randomized controlled trials |
| 793 Rizzo R, Quaranta A, De Paoli M,et al. Three-Dimensional Bone Augmentation and Immediate Implant Placement via Transcrestal Sinus Lift: 8-Year Clinical Outcomes. Int J Periodontics Restorative Dent. 2018;38(3):423-429. | Non randomized controlled trials |
| 794 Sato Y, Honda Y, Asoh T, et al. Hypovitaminosis D and decreased bone mineral density in amyotrophic lateral sclerosis. Eur Neurol. 1997;37(4):225-229. | Non randomized controlled trials |
| 795 Iwamoto J, Takada T, Sato Y, et al. Effect of risedronate on speed of sound in postmenopausal women with osteoporosis. World J Orthop. 2013;4(4):316-322. | Non randomized controlled trials |
| 796 Ehrlich GD, Hu FZ, Sotereanos N, et al. What role do periodontal pathogens play in osteoarthritis and periprosthetic joint infections of the knee? 2014;12(1):13-20. | Non randomized controlled trials |

| | |
|---|---|
| 797 Le A, Wang Z, Shan L, et al. Peritoneal vaginoplasty by Luohu I and Luohu II technique: a comparative study of the outcomes. Eur J Med Res. 2015;20(1):69. | Non randomized controlled trials |
| 798 Tenjin T, Miyamoto S, Miyake N, et al. Effect of blonanserin on cognitive function in antipsychotic-naïve first-episode schizophrenia. Hum Psychopharmacol. 2012;27(1):90-100. | Non randomized controlled trials |
| 799 Goldberg RJ, Spencer FA, Okolo J,et al. Long-term trends (1986-2003) in the use of coronary reperfusion strategies in patients hospitalized with acute myocardial infarction in central Massachusetts. Int J Cardiol. 2008;131(1):83-89. | Non randomized controlled trials |
| 800 Sahin C, Aras HI. The Effect of Nasal Packing Removal on Patients Anxiety. Med Arch. 2015;69(6):393-395. | Non randomized controlled trials |
| 801 Cassani RS, Fassini PG, Silvah JH, et al. Impact of weight loss diet associated with flaxseed on inflammatory markers in men with cardiovascular risk factors: a clinical study. Nutr J. 2015;14:5. | Non randomized controlled trials |
| 802 Barakat MI, Elhady W, Gouda M, et al. Surgical management of intractable spasticity. Eur Spine J. 2016;25(3):928-935. | Non randomized controlled trials |
| 803 Popovic M, Tasic M, Grubisa M. Efficacy And Safety Of IVUS-Guided Percutaneous Coronary Interventions. Experimental and Applied Biomedical Research (EABR).;16(2):115-9. | Non randomized controlled trials |
| 804 Safarinejad MR, Kolahi AA, Hosseini L. The effect of the mode of delivery on the quality of life, sexual function, and sexual satisfaction in primiparous women and their husbands. J Sex Med. 2009;6(6):1645-1667. | Non randomized controlled trials |
| 805 He Y, Coonar A, Gelvez-Zapata S,et al. Evaluation of a robot-assisted video-assisted thoracoscopic surgery programme. Exp Ther Med. 2014;7(4):873-876. | Non randomized controlled trials |
| 806 Behr TM, Liersch T, Greiner-Bechert L, et al. Radioimmunotherapy of small-volume disease of metastatic colorectal cancer: Results of a phase II trial with the iodine-131–labeled humanized anti–carcinoembryonic antigen antibody hMN-14. Cancer: Interdisciplinary International Journal of the American Cancer Society. 2002;94(S4):1373-81. | Non randomized controlled trials |
| 807 Ran B, Chen XY, Zhang GY, et al. Comparison of the sagittal profiles among thoracic idiopathic scoliosis patients with different Cobb angles and growth potentials. J Orthop Surg Res. 2014;9:19. | Non randomized controlled trials |
| 808 Shaw LJ, Bairey Merz CN, Azziz R, et al. Postmenopausal women with a history of irregular menses and elevated androgen measurements at high risk for worsening cardiovascular event-free survival: results from the National Institutes of Health--National Heart, Lung, and Blood Institute sponsored Women's Ischemia Syndrome Evaluation. J Clin Endocrinol Metab. 2008;93(4):1276-1284. | Non randomized controlled trials |
| 809 Liu D, Jia H, Fu Y, et al. Prognostic utility of coronary computed tomographic angiography: a 5-year follow-up in type 2 diabetes patients with suspected coronary artery disease. J Diabetes Res. 2014:103459. | Non randomized controlled trials |
| 810 Yamamoto N, Suyama H, Yamamoto N,et al. Immunotherapy of metastatic breast cancer patients with vitamin D-binding protein-derived macrophage activating factor (GcMAF). Int J Cancer. 2008;122(2):461-467. | Non randomized controlled trials |
| 811 Xu Z, Dong M, Dong R, et al. Further pieces of evidence to the pulmonary origin of sevoflurane escaping to the operating room during general anaesthesia. Cell Biochem Biophys. 2014;70(1):705. | Non randomized controlled trials |
| 812 Chan MF, Wong FK, Chang K, et al. Identifying patient readmission subtypes from unplanned readmissions to hospitals in Hong Kong: a cluster analysis. Nurs Health Sci. 2009;11(1):37-44. | Non randomized controlled trials |

| | |
|---|---|
| 813 Yener AU, Ozcan S, Budak AB,et al. The effects of 21 and 23 milimeter aortic valve prosthesis on hemodynamic performance and functional capacity in young adults. Pak J Med Sci. 2014;30(2):356-360. | Non randomized controlled trials |
| 814 Ge J, Baumgart D, Haude M, et al. Role of intravascular ultrasound imaging in identifying vulnerable plaques. Herz. 1999;24(1):32-41. | Non randomized controlled trials |
| 815 Muller J, Karl A, Denke C, et al. Biofeedback for pain management in traumatised refugees. Cogn Behav Ther. 2009;38(3):184-190. | Non randomized controlled trials |
| 816 Geraerts E, McNally RJ, Jelicic M, et al. Linking thought suppression and recovered memories of childhood sexual abuse. Memory. 2008;16(1):22-28. | Non randomized controlled trials |
| 817 Kathiresan G, Jayachandran J, Veeraghavan S. Cardiac autonomie reaction to eccentric isokinetic strength training in healthy sexagenarian men. (2011): S41-S45. | Non randomized controlled trials |
| 818 Steinfeld SD, Demols P, Salmon I, et al. Infliximab in patients with primary Sjögren's syndrome: a pilot study. Arthritis & Rheumatism. 2001 Oct;44(10):2371-5. | Non randomized controlled trials |
| 819 Steinfeld SD, Demols P, Appelboom T. Infliximab in primary Sjögren's syndrome: One-year followup. Arthritis & Rheumatism. 2002 Dec;46(12):3301-3. | Non randomized controlled trials |
| 820 Fujii Y, Tanaka H, Toyooka H, et al. Airway occlusion pressure is an indicator of respiratory depression with isoflurane. J Anesth. 1994;8(3):253-255. | Non randomized controlled trials |
| 821 Shanker AS, Phanikrishna B, Reddy CB. Association between erectile dysfunction and coronary artery disease and it's severity.(2013): 180-186. | Non randomized controlled trials |
| 822 Jeong H, Lee SH, Jang EA, et al. Haemodynamics and cerebral oxygenation during arthroscopic shoulder surgery in beach chair position under general anaesthesia. Acta Anaesthesiol Scand. 2012;56(7):872-879. | Non randomized controlled trials |
| 823 Fujii Y, Toyooka H, Ishikawa E, et al. Blood flow velocity in the middle cerebral artery response to tourniquet release. Anaesth Intensive Care. 1999;27(3):253-256. | Non randomized controlled trials |
| 824 Burbaud P, Camus O, Guehl D, eet al. Influence of cognitive strategies on the pattern of cortical activation during mental subtraction. A functional imaging study in human subjects. Neurosci Lett. 2000;287(1):76-80. | Non randomized controlled trials |
| 825 Schofer MD, Hrabal SA, Timmesfeld N, et al. Cable wakeboarding, a new trendy sport: analysis of injuries with regard to injury prevention. Scandinavian journal of medicine and science in sports. 2014. | Non randomized controlled trials |
| 826 Boldt J, Mengistu A. A new plasma-adapted hydroxyethyl starch preparation: in vitro coagulation studies. J Cardiothorac Vasc Anesth. 2010;24(3):394-398. | Non randomized controlled trials |
| 827 Mengistu AM, Wolf MW, Boldt J, et al. Evaluation of a new platelet function analyzer in cardiac surgery: a comparison of modified thromboelastography and whole-blood aggregometry. J Cardiothorac Vasc Anesth. 2008;22(1):40-46. | Non randomized controlled trials |
| 828 Mengistu AM, Mayer J, Boldt J, et al. Usefulness of monitoring platelet function by multiple electrode aggregometry in primary coronary artery bypass surgery. J Cardiothorac Vasc Anesth. 2011;25(1):42-47. | Non randomized controlled trials |

| | |
|---|---|
| 829 Kumle B, Boldt J, Suttner SW,et al. Influence of prolonged cardiopulmonary bypass times on splanchnic perfusion and markers of splanchnic organ function. Ann Thorac Surg. 2003;75(5):1558-1564. | Non randomized controlled trials |
| 830 Schulte-Uebbing C, Schlett S, Craiut I, et al. Chronical cervical infections and dysplasia (CIN I, CIN II): Vaginal vitamin D (high dose) treatment: A new effective method?. Dermatoendocrinol. 2014;6(1):e27791. | Non randomized controlled trials |
| 831 Suttner S, Lang K, Piper SN, et al. Continuous intra- and postoperative thoracic epidural analgesia attenuates brain natriuretic peptide release after major abdominal surgery. Anesth Analg. 2005;101(3):896-903. | Non randomized controlled trials |
| 832 Bhui K, Warfa N. Trauma, khat and common psychotic symptoms: a quantitative study. J Ethnopharmacol. 2010;131(2):459-463. | Non randomized controlled trials |
| 833 Ozaki H, Miyachi M, Nakajima T, et al. Muscle volume and strength and arterial compliance after walk training with blood flow reduction in elderly women. J Am Geriatr Soc. 2010;58(8):1597-1598. | Non randomized controlled trials |
| 834 Fechner J, Ihmsen H, Hatterscheid D, et al. Pharmacokinetics and clinical pharmacodynamics of the new propofol prodrug GPI 15715 in volunteers. Anesthesiology. 2003;99(2):303-313. | Non randomized controlled trials |
| 835 Weber A, Reser D, Reuthebuch O, et al. right anterior minithoracotomy for minimal access aortic valve replacement. J Card Surg. 2009. | Non randomized controlled trials |
| 836 Freschi SA, Dodson N. Analysis of compression forces between varying sizes of cannulated screws versus rail external fixation for treatment of Jones type fifth metatarsal fracture. J Foot Ankle Surg. 2008;47(4):295-298. | Non randomized controlled trials |
| 837 Abou-Elela A, Reyad I, Morsy A, et al. Continence after radical prostatectomy with bladder neck preservation. (2007): 96-101. | Non randomized controlled trials |
| 838 Roblin X, Peyrin-Biroulet L, Phelip JM, et al. A 6-thioguanine nucleotide threshold level of 400 pmol/8 x 10(8) erythrocytes predicts azathioprine refractoriness in patients with inflammatory bowel disease and normal TPMT activity. Am J Gastroenterol. 2008;103(12):3115-3122. | Non randomized controlled trials |
| 839 Souglakos J, Vardakis N, Androulakis N, et al. Irinotecan plus weekly 5-fluorouracil and leucovorin as salvage treatment for patients with metastatic colorectal cancer: a phase II trial. Dig Dis. 2007;25(1):100-105. | Non randomized controlled trials |
| 840 Kwon OJ, Kwak JY, Kang CM. The impact of gender and age matching for long-term graft survival in living donor renal transplantation. Transplant Proc. 2005;37(2):726-728. | Non randomized controlled trials |
| 841 Ben-Gal Y, Mohr R, Uretzky G, et al. Drug-eluting stents versus arterial myocardial revascularization in patients with diabetes mellitus. J Thorac Cardiovasc Surg. 2006;132(4):861-866. | Non randomized controlled trials |
| 842 Behr TM, Griesinger F, Riggert J, et al. High-dose myeloablative radioimmunotherapy of mantle cell non-Hodgkin lymphoma with the iodine-131-labeled chimeric anti-CD20 antibody C2B8 and autologous stem cell support. Results of a pilot study. Cancer. 2002;94(4 Suppl):1363-1372. | Non randomized controlled trials |
| 843 Affandi B, Korver T, Geurts TB,et al. A pilot efficacy study with a single-rod contraceptive implant (Implanon) in 200 Indonesian women treated for < or = 4 years. Contraception. 1999;59(3):167-174. | Non randomized controlled trials |

| | |
|---|---|
| 844 Barbaro G, Di Lorenzo G, Grisorio B, et al. Incidence of dilated cardiomyopathy and detection of HIV in myocardial cells of HIV-positive patients. Gruppo Italiano per lo Studio Cardiologico dei Pazienti Affetti da AIDS. N Engl J Med. 1998;339(16):1093-1099. | Non randomized controlled trials |
| 845 Feifel D, Moutier CY, Perry W. Safety and tolerability of a rapidly escalating dose-loading regimen for risperidone. J Clin Psychiatry. 2000;61(12):909-911. | Non randomized controlled trials |
| 846 Friedman AJ, Thomas PP. Does low-dose combination oral contraceptive use affect uterine size or menstrual flow in premenopausal women with leiomyomas?. Obstet Gynecol. 1995;85(4):631-635. | Non randomized controlled trials |
| 847 Herman TS, Jochelson MS, Teicher BA, et al. A phase I-II trial of cisplatin, hyperthermia and radiation in patients with locally advanced malignancies. Int J Radiat Oncol Biol Phys. 1989;17(6):1273-1279. | Non randomized controlled trials |
| 848 Slutsky RA, Brown JJ. Chest radiographs in congestive heart failure: response to therapy in acute and chronic heart disease. Radiology. 1985;154(3):577-580. | Non randomized controlled trials |
| 849 Verma I, Chugh C, Sood D, et al. Perinatal Outcome in Pregnancies Associated with Hypertension: A Prospective Cohort Study in a Rural Tertiary Care Teaching Hospital of North India. Indian J Community Med. 2021;46(4):651-656. | Non randomized controlled trials |
| 850 Hua K, Zhao Y, Dong R,et al. Minimally Invasive Cardiac Surgery in China: Multi-Center Experience. Med Sci Monit. 2018;24:421-426. | Non randomized controlled trials |
| 851 Niv Y, Boltin D, Halpern M, et al. Membrane-bound mucins and mucin terminal glycans expression in idiopathic or Helicobacter pylori, NSAID associated peptic ulcers. Dig Dis Sci. 2012;57(10):2535-2544. | Non randomized controlled trials |
| 852 El-Shmaa NS, El Amrousy D, El Feky W. The efficacy of pre-emptive dexmedetomidine versus amiodarone in preventing postoperative junctional ectopic tachycardia in pediatric cardiac surgery. Ann Card Anaesth. 2016;19(4):614-620. | Non randomized controlled trials |
| 853 Iwamoto J, Takeda T, Sato Y, et al. Comparison of effect of treatment with etidronate and alendronate on lumbar bone mineral density in elderly women with osteoporosis. Yonsei Med J. 2005;46(6):750-758. | Non randomized controlled trials |
| 854 Sinha SK, Mishra V, Afdaali N, et al. Coronary Angiography Safety between Transradial and Transfemoral Access. Cardiol Res Pract. 2016;2016:4013843. | Non randomized controlled trials |
| 855 Saitoh Y, Kaneda K, Murakawa M. The effect of ulinastatin pre-treatment on vecuronium-induced neuromuscular block in patients with hepatic cirrhosis. Anaesthesia. 2002;57(3):218-222. | Non randomized controlled trials |
| 856 Dave J, Vaghela S. A comparison of the sedative, hemodynamic, and respiratory effects of dexmedetomidine and propofol in children undergoing magnetic resonance imaging. Saudi J Anaesth. 2011;5(3):295-299. | Non randomized controlled trials |
| 857 Reuben SS, Buvanendran A, Kroin JS, et al. The analgesic efficacy of celecoxib, pregabalin, and their combination for spinal fusion surgery. Anesth Analg. 2006;103(5):1271-1277. | Non randomized controlled trials |
| 858 Reuben SS, Buvanendran A, Kroin JS,et al. The analgesic efficacy of celecoxib, pregabalin, and their combination for spinal fusion surgery. Anesth Analg. 2006;103(5):1271-1277. | Duplicates |
| 859 Breuning SE, Ferguson DG, Davidson NA,et al. Effects of thioridazine on the intellectual performance of mentally retarded drug responders and nonresponders. Arch Gen Psychiatry. 1983;40(3):309-313. | Non randomized controlled trials |

| | |
|---|---|
| 860 Ozayar, E. (2016). The Effect of Bougie Size on the Incidence of Postoperative Sore Throat in Bariatric Surgery . | Non randomized controlled trials |
| 861 Blake DF, Young DA, Brown LH. Transcutaneous oximetry: normal values for the lower limb. Diving Hyperb Med. 2014;44(3):146-153. | Non randomized controlled trials |
| 862 Fujii Y, Tanaka H, Saitoh Y,et al. Effects of calcium channel blockers on circulatory response to tracheal intubation in hypertensive patients: nicardipine versus diltiazem. Can J Anaesth. 1995;42(9):785-788. | Non randomized controlled trials |
| 863 Boldt J, Dieterich HA, Kling D, et al. Hemodynamic effects of enoximone in cardiac surgery patients. J Cardiovasc Pharmacol. 1989;14 Suppl 1:S50-S56. | Non randomized controlled trials |
| 864 Krasnici S, Ertel W, Fassola I. Volar locking plate removal following distal radius fractures: indications and outcomes for 71 fractures. J Hand Surg Eur Vol. 2020;45(8):NP13-NP14. | Non randomized controlled trials |
| 865 Ji YW, Kim M, Kang DSY, et al. Effect of Lowering Laser Energy on the Surface Roughness of Human Corneal Lenticules in SMILE. J Refract Surg. 2017;33(9):617-624. | Non randomized controlled trials |
| 866 Robertson IJ, Bennani F, Ryan RS, et al. Paediatric Ewing-like sarcoma arising from the cranium - a unique diagnostic challenge. Diagn Pathol. 2016;11(1):54. | Non randomized controlled trials |
| 867 Sirpal S, Potter JD. The troponin T isoform shift may constitute a protective compensatory mechanism in hypertrophic cardiomyopathy. J Biol Chem. 2008. | Non randomized controlled trials |
| 868 Sharma, Manisha, et al.The hydrophobic rich N-and C-terminal tails of β-catenin facilitate nuclear import. Journal of Biological Chemistry 290.30 (2015): 18479. | Non randomized controlled trials |
| 869 Salt and water imbalance following pituitary surgery. Eur J Endocrinol. 2014;171(6):Z1. | Non randomized controlled trials |
| 870 La Tonya Noel, Robert LG, Jill Gromer, et al. Depression in Dementia Caregivers: An Analysis of Meaning and Symptom Severity. Journal of Gerontological Social Work. 2014. | Non randomized controlled trials |
| 871 Venkatadass K, Bittersohl B, Fornari ED, et al. Does incisional wound VAC after major hip surgery in obese pediatric patients reduce wound infection and scar formation? A pilot study. Clin Orthop Relat Res. 2013;471(8):2730. | Non randomized controlled trials |
| 872 Selective cerebro-myocardial perfusion under mild hypothermia during primary repair for aortic coarctation with ventricular septal defect. Artif Organs. 2013;37(4):418. | Non randomized controlled trials |
| 873 Presurgical nasoalveolar molding with computer-aided reverse-engineering and rapid prototyping technique in infants with unilateral cleft lip and palate. J Oral Maxillofac Surg. 2011;69(11):2936. | Non randomized controlled trials |
| 874 Xia YF, Huang S, Li X, et al. A family-based association study of megsin A23167G polymorphism with susceptibility and progression of IgA nephropathy in a Chinese population. Clin Nephrol. 2006;65(3):153-159. | Non randomized controlled trials |
| 875 Nezhat F, Nezhat C, Pennington E, et al. Laparoscopic segmental resection for infiltrating endometriosis of the rectosigmoid colon: a preliminary report. Surg Laparosc Endosc. 1992;2(3):212-216. | Non randomized controlled trials |
| 876 Van Thiel DH. Successful treatment of end stage liver disease due to hepatitis C prior to liver transplantation. Hepatology. 1995;22(5):1619. | Non randomized controlled trials |

| | |
|---|---|
| 877 Tseng E, Potter SM, Picciano MF. Dietary protein source and plasma lipid profiles of infants. Pediatrics. 1990;85(4):548-552. | Non randomized controlled trials |
| 878 Glueck CJ, Mellies MJ, Dine M,et al. Safety and efficacy of long-term diet and diet plus bile acid-binding resin cholesterol-lowering therapy in 73 children heterozygous for familial hypercholesterolemia. Pediatrics. 1986;78(2):338-348. | Non randomized controlled trials |
| 879 Salas-Salvadó J, Bulló M, Babio N, et al. Erratum. Reduction in the Incidence of Type 2 Diabetes With the Mediterranean Diet: Results of the PREDIMED-Reus nutrition intervention randomized trial. Diabetes Care 2011;34:14-19. Diabetes Care. 2018;41(10):2259-2260. | Non randomized controlled trials |
| 880 Hashizume K, Suzuki S, Komatsu A, et al. Administration of recombinant human growth hormone normalizes GH-IGF1 axis and improves malnutrition-related disorders in patients with anorexia nervosa. Endocr J. 2007;54(2):319-327. | Non randomized controlled trials |
| 881 Velikova T, Kyurkchiev D, Spassova Z, et al. Alterations in cytokine gene expression profile in colon mucosa of Inflammatory Bowel Disease patients on different therapeutic regimens. Cytokine. 2017;92:12-19. | Non randomized controlled trials |
| 882 He J, Wen J. Analysis Model of the Impact of Refined Intervention in Operating Room on Patients' Recovery Quality and Complications after Thoracic Surgery Based on Deep Neural Network. J Healthc Eng. 2021:7006120. | Non randomized controlled trials |
| 883 Safarinejad MR. Analysis of association between the 5-HTTLPR and STin2 polymorphisms in the serotonin-transporter gene and clinical response to a selective serotonin reuptake inhibitor (sertraline) in patients with premature ejaculation. BJU Int. 2015;115(3):E9. | Non randomized controlled trials |
| 884 Chen Y, Li Q. Analysis of the Effect of Nursing Intervention on Children with Respiratory Tract Infection Based on Comprehensive Nursing. J Healthc Eng. 2021:6923823. | Non randomized controlled trials |
| 885 Zhang Y, Zhang Q, Lv J, et al. Analysis on the diagnosis of infection risk factors and the incidence of ureteroscopy holmium laser lithotripsy and vagal excitation under computed tomography image information health technology. Neurosci Lett. 2020:135224. | Non randomized controlled trials |
| 886 Roth B, Marciniak B, Engelhardt T, et al. Anatomic relationship between the internal jugular vein and the carotid artery in pre-school children--an ultrasonographic study .2008;18(11):1045-1049. | Non randomized controlled trials |
| 887 Babu BS, Shetty N, C N, et al. Assessing the Contact Angle Between Dentin Treated With Irrigation and Calcium Hydroxide and Root Canal Sealers. Cureus. 2022;14(9):e29474. | Non randomized controlled trials |
| 888 Henson D, Tahhan AS, Nardo D, et al. Association Between ApoA-I (Apolipoprotein A-I) Immune Complexes and Adverse Cardiovascular Events-Brief Report. Arterioscler Thromb Vasc Biol. 2019;39(9):1884-1892. | Non randomized controlled trials |
| 889 Khaki-Khatibi F, Samadi N, Yaghoubi A. Association between inflammatory factor, lipid peroxidation and total-antioxidant in non-diabetic patients of coronary artery disease. Journal of Research in Clinical Medicine, 2016, 4(3): 182-182. | Non randomized controlled trials |
| 890 Akinyi MV, Dandara C, Gamieldien J, et al. Association of transforming growth factor β-1 (TGFB1) regulatory region polymorphisms with myasthenia gravis-related ophthalmoparesis . J Neuroimmunol. 2012;246(1-2):96-99. | Non randomized controlled trials |
| 891 Ueshima H, Ata A, Otake H. Clinical efficacy of a supraglottic airway device with a pressure gauge for robot-assisted surgery.J Clin Anesth. 2017;41:74-75. | Non randomized controlled trials |

| | |
|---|---|
| 892 Choi J, Shim JH, Shin YM, et al. Clinical significance of the best response during repeated transarterial chemoembolization in the treatment of hepatocellular carcinoma. J Hepatol. 2014;60(6):1212-1218. | Non randomized controlled trials |
| 893 Diao M, Sun J, Tian F, et al. Cochlear implant device activation after 7 days in cochlear implant recipients. Eur Arch Otorhinolaryngol. 2019;276(1):281. | Non randomized controlled trials |
| 894 Puthenparampil M, Poggiali D, Causin F, et al. Cortical relapses in multiple sclerosis. Mult Scler. 2016;22(9):1184-1191. | Non randomized controlled trials |
| 895 Jayaraj, Joshua Chadwick, Lusine Abrahamyan, et al. Comparative Effectiveness of Complete Revascularization versus Infarct Related Artery-only Percutaneous Coronary Revascularization for Multivessel Disease after ST-Segment Elevation Myocardial Infarction. International Cardiovascular Forum Journal. 2016(08). | Non randomized controlled trials |
| 896 Adesola A M, Azeez O M. Comparison of cardio-pulmonary responses to forward and backward walking and running. African Journal of Biomedical Research, 2009, 12(2): 95-100. | Non randomized controlled trials |
| 897 Chernecky C C, Denise Macklin B S N, Jarvis W R, et al. Comparison of Central Line-Associated Bloodstream Infection Rates when Changing to a Zero Fluid Displacement Intravenous Needleless Connector in Acute Care Settings. Am J Infect Control. 2016;44(1):123. | Non randomized controlled trials |
| 898 Ma Y, Zhou J, Wang H. Dosimetric Comparison between Volumetric Modulated Arc Therapy (VMAT) and Intensity-Modulated Radiotherapy (IMRT) for Dental Structures of Head and Neck Cancer Patients.J Healthc Eng.2022:4998997. | Non randomized controlled trials |
| 899 Ren D, Feng M, Zhang S, et al. Effect of Apatinib Combined with Seggio on the Expression of Serum AFP and CA724 and Long-Term Survival Rate in Patients with Advanced Gastric Cancer Undergoing Comfortable Nursing Intervention. J Healthc Eng. 2022:2004973. | Non randomized controlled trials |
| 900 Mansour, Ahmed E, Mohamed E,et al.Effect of Aspergillus Fumigatus sensitization and colonization on lung function and airways inflammation in asthma.Egyptian Journal of Chest Diseases and Tuberculosis.2012: 243-246. | Non randomized controlled trials |
| 901 Yalçın Bahat P, Yüksel Özgör B, Turan G, et al. The Effect of Neural Therapy on Long-Term Postoperative Discomforts after Bilateral Tubal Ligation. Complement Med Res. 2020. | Non randomized controlled trials |
| 902 Cheng X, Kong X, Fan Y, et al. Effects of Equine-Assistant Activity on Gross Motor Coordination in Children Aged 8 to 10 Years.J Healthc Eng. 2022:3623686. | Non randomized controlled trials |
| 903 Xiaoli S, Weiyan H, Li D. Effects of Neonatal Feeding Patterns on Infant Health.Appl Bionics Biomech. 2022:2225415. | Non randomized controlled trials |
| 904 Guo J, Liu Y, Feng Y, et al. Effects of Surgical Treatment Guided by the Three-Column Classification Method on Knee Joint Function and Postoperative Complications in Patients with Tibial Plateau Fractures. Comput Intell Neurosci. 2022:5935102. | Non randomized controlled trials |
| 905 Lv X, Zhao Y, Wu Y. Effects of the Training of Aerobic Function on Clinical Symptoms and Quality of Life in Patients with Medium and Advanced Lung Cancer.J Healthc Eng. 2022:6753959. | Non randomized controlled trials |
| 906 Breuning SE, Davis VJ, Matson JL, et al. Effects of thioridazine and withdrawal dyskinesias on workshop performance of mentally retarded young adults. Am J Psychiatry. 1982;139(11):1447-1454. | Non randomized controlled trials |

| | |
|---|---|
| 907 Allen S, Aghajanyan IG. Efficacy of thermobalancing therapy for chronic prostatitis/chronic pelvic pain syndrome, confirmed by clinical study, may suggest etiology and pathophysiology of this disease.Can Urol Assoc J.2017. | Non randomized controlled trials |
| 908 Liu J, Xun Z. Evaluation of the Effect of Comprehensive Nursing in Psychotherapy of Patients with Depression . Comput Math Methods Med. 2021;2021:2112523. | Non randomized controlled trials |
| 909 Barbalho M, Coswig VS, Steele J, et al. Evidence of a Ceiling Effect for Training Volume in Muscle Hypertrophy and Strength in Trained Men - Less is More? Int J Sports Physiol Perform. 2020;15(2):268-277. | Non randomized controlled trials |
| 910 Zheng Q, Cao L, Ma H, et al. Evidence-Based Care Can Improve Treatment Compliance and Quality of Life of Patients with Acute Pancreatitis. J Healthc Eng.2022:7621658. | Non randomized controlled trials |
| 911 Ma M, He F, Lv X, et al. Feasibility and effectiveness of thoracoscopic pulmonary segmentectomy for non-small cell lung cancer .Medicine (Baltimore). 2020;99(5):e18959. | Non randomized controlled trials |
| 912 Li Z, Krak M, Zerlin A, et al. The impact of spices on vegetable consumption: A pilot study. Food and Nutrition Sciences, 2015, 6(4): 437-444. | Non randomized controlled trials |
| 913 Barbalho M, Gentil P, Raiol R, et al Influence of Adding Single-Joint Exercise to a Multijoint Resistance Training Program in Untrained Young Women. J Strength Cond Res. 2020;34(8):2214-2219. | Non randomized controlled trials |
| 914 Chandra R K. Influence of multinutrient supplement on immune responses and infection-related illness in 50–65 year old individuals. 2002. | Non randomized controlled trials |
| 915 Xu S, Zhou B, Zhou B, et al. The Level of HbA1c Evaluates the Extent of Coronary Atherosclerosis Lesions and the Prognosis in Diabetes with Acute Coronary Syndrome.Comput Math Methods Med. 2022:7796809. | Non randomized controlled trials |
| 916 Sato Y, Honda Y, Jun I. Long-term oral anticoagulation therapy and the risk of hip fracture in patients with previous hemispheric infarction and nonrheumatic atrial fibrillation. Cerebrovasc Dis. 2010;29(1):73-78. | Non randomized controlled trials |
| 917 Haytoglu Z, Gundeslioglu OO. Mediterranean fever gene variants and colchicine therapy in periodic fever, aphthous stomatitis pharyngitis, adenitis syndrome in a Mediterranean region.Expert Rev Clin Immunol. 2019;15(5):571-575. | Non randomized controlled trials |
| 918 Van Kann D H H, de Vries S I, Schipperijn J, et al. A multicomponent schoolyard intervention targeting children's recess physical activity and sedentary behavior: effects after 1 year. Journal of Physical Activity and Health.2017, 14(11): 866-875. | Non randomized controlled trials |
| 919 Al-Omiri MK. Muscle activity and masticatory efficiency with bilateral extension base removable partial dentures with different cusp angles.J Prosthet Dent. 2018;119(3):369-376. | Non randomized controlled trials |
| 920 Li Y, Xu W, Jiang Z, et al. Neutropenia and invasive fungal infection in patients with hematological malignancies treated with chemotherapy: a multicenter, prospective, non-interventional study in China. Tumour Biol. 2014;35(11):11693. | Non randomized controlled trials |
| 921 Adogwa O, Elsamadicy AA, Han J, et al. Outcomes After Anterior Lumbar Interbody Fusion Versus Transforaminal Lumbar Interbody Fusion for the Treatment of Symptomatic L5-S1 Spondylolisthesis: A Prospective, Multi-Institutional Comparative Effectiveness Study. World Neurosurg. 2015. | Non randomized controlled trials |

| | |
|---|---|
| 922 Wei LM, Zhu YQ, Liu F, et al. Percutaneous Aspiration Thrombectomy for Arterial Thromboembolism during Infrainguinal Endovascular Recanalization. PLoS One. 2015;10(10):e0140494. | Non randomized controlled trials |
| 923 Vuijk P, van Lier PA, Huizink AC, et al. Prenatal smoking predicts non-responsiveness to an intervention targeting attention-deficit/hyperactivity symptoms in elementary schoolchildren. J Child Psychol Psychiatry. 2006;47(9):891-901. | Non randomized controlled trials |
| 924 Brady L W, Freire J E, Longton W A, et al. Radiation therapy for macular degeneration: technical considerations and preliminary results. Int J Radiat Oncol Biol Phys 1997;39:945-8. Int J Radiat Oncol Biol Phys. 2014;90(3):717-718. | Non randomized controlled trials |
| 925 Ghandehari K, Ahmadi F, Afzalnia A. Results of Surgical and Nonsurgical Treatment of Aneurysms in a Developing Country. Stroke Res Treat. 2016;2016:2308436. | Non randomized controlled trials |
| 926 Huang H, Xia C, Hu M, et al. The role of laryngeal ultrasound in diagnosis of infant laryngomalacia. Int J Pediatr Otorhinolaryngol. 2019;124:111-115. | Non randomized controlled trials |
| 927 Ziazadeh D, Mater R, Himelhoch B, et al. Single-dose del Nido Cardioplegia in Minimally Invasive Aortic Valve Surgery. Semin Thorac Cardiovasc Surg. | Non randomized controlled trials |
| 928 Xing F, Ouyang Y, Li X. Total facial nerve decompression in severe idiopathic recurrent facial palsy: its long-term follow-up results. Acta Oto-Laryngologica. 2019, 139(11): 1049-1051. | Non randomized controlled trials |
| 929 Shen M, Liu L, Lin J, et al. Treatment of Very Severe Osteoporotic Vertebral Compression Fractures with Balloon KyphoplastyNeuroradiology. 2013. | Non randomized controlled trials |
| 930 Smith M M, Trexler E T, Sommer A J, et al. Unrestricted Paleolithic diet is associated with unfavorable changes to blood lipids in healthy subjects. Int J Exerc Sci, 2014, 7(2): 128-39. | Non randomized controlled trials |
| 931 Riener EK, Arnold N, Kommoss F, et al. The prognostic and predictive value of immunohistochemically detected HER-2/neu overexpression in 361 patients with ovarian cancer: a multicenter study. Gynecol Oncol. 2004;95(1):89-94. | Non randomized controlled trials |
| 932 Iwamoto M, Sato M, Kono M, et al. Walnuts Lower Serum Cholesterol in Japanese Men and Women. The Journal of nutrition, 2000, 130(2): 171-176. | Non randomized controlled trials |
| 933 Fujii Y, Tanaka H, Toyooka H. Intraoperative ventilation with air and oxygen during laparoscopic cholecystectomy decreases the degree of postoperative hypoxaemia. Anaesth Intensive Care. 1996;24(1):42-44. | No mention of randomisation in method |
| 934 Jayaraj JC, Abrahamyan L, Demirchyan A. Clinical outcomes of complete versus lesion only primary percutaneous coronary revascularization for multivessel disease and ST-segment elevation myocardial infarction. Indian Heart Journal. 2016. | No full-text |
| 935 Marina P, Kurochka, Ekaterina I, et al. Comparative Assessment of Adaptation Capabilities of Fruits and Newborns in Pregnant Women without and Presence of Hypertensive Disorders During PregnancyInternational Research Journal. 2016. | No full-text |
| 936 Anna B, Lopatina. Effectiveness of Dr. Nona CapillarotherapyInternational Research Journal. 2016. | No full-text |
| 937 T Popescu, M Mota. Evaluation of diabetic retinopathy in a group of type 2 diabetic patientsRomanian Journal of Diabetes Nutrition and Metabolic Diseases. 2012. | No full-text |
| 938 Alexander N, Legotkin, Anna B, et al. Hormonal Rebuilding and SportInternational Research Journal. 2016. | No full-text |

| | |
|---|---|
| 939 Suzuki M, Sumiyoshi T, Miyachi H, et al. Mechanical circulatory support with optimal coronary reflow in a cardiogenic shock complicating acute myocardial infarction. Journal of cardiology. 2016. | No full-text |
| 940 Marina A, Posokhova, Nadezhda M, et al. The Use of Respiratory Techniques for the Development of Speech Breathing in Children with Speech ImpairmentJournal of Scientific Articles Health and Education in the XXI Century. 2016. | No full-text |
| 941 Amit Patel. Usefulness of Ivermectin in COVID-19 Illness. Social Science Research Network. 2020 | No full-text |
| 942 Shokhin K, Nazarov, Kh N, et al. Antibiotic prophylaxis of postoperative wound infectious and inflammatory complications in abdominal surgeryBulletin of Avicenna. 2015. | No full-text |
| 943 Anna B, Lopatina. The Application of Capillarotherapy in Patients with Type I Diabetes and its Influence on the Parametric Characteristics of the CapillarsJournal of Scientific Articles Health and Education in the XXI Century. 2016. | No full-text |
| 944 Sergey A, Emelyanov, Mikhail S, et al. The Application of Dairy Products of Healthy Food in Correction of the Functional States of Nursing MothersModern Science and Innovation. 2016. | No full-text |
| 945 Xu L, Chi C, Wang C, et al. Association between bevacizumab-related chemotherapy regimens and serum vascular endothelial growth factor-A165b level in patients with metastatic colorectal cancer. Eur Rev Med Pharmacol Sci. 2014;18(18):2687-92. | No full-text |
| 946 Oana B, Ionescu-Tirgoviste C. Atomic force microscopy. Its potential in diabetes researchRomanian Journal of Diabetes Nutrition and Metabolic Diseases. 2007. | No full-text |
| 947 Suh SH, Chung TS, Kim DJ, et al. Brainstem venous congestion after transvenous embolization in cavernous dural arteriovenous fistula: treatment with stent grafts. Neuroradiology. 2014;56:805. | No full-text |
| 948 Bevilacqua L, Liani G, Castronovo G, et al. Clinical and spectrophotometric evaluation after chlorhexidine use in periodontal flap surgery: A prospective randomized clinical trial. American Journal of Dentistry. 2016;29(2):75-80. | No full-text |
| 949 SON HS, FANG YH, Hwang Z, et al. Comparison of pulsatile and non-pulsatile extracorporeal circulation on the pattern of coronary artery blood flow. The Korean Journal of Thoracic and Cardiovascular Surgery. 2005:101-9. | No full-text |
| 950 Manoj Kumar, Rakesh Bahadur Singh, Shilpi AgrawalDexmedetomidine and Postoperative Shivering: A Randomized Placebo Control StudyJournal of Medical Science and Clinical Research. 2014. | No full-text |
| 951 Wu WC, Lee WJ, Yeh C, et al.Do different bariatric surgery procedures impact hepassocin plasma levels in patients with type 2 diabetes mellitus?. Liver research.2019. | No full-text |
| 952 Cha KY, Wirth DP. Does prayer influence the success of in vitro fertilization-embryo transfer? Report of a masked, randomized trial. The Journal of reproductive medicine. 2001;46(9):781-7. | No full-text |
| 953 Badowski M, Shultz C,Harris DT. The effect of anticoagulant choice on collection of cord blood. Transfusion. 2014. | No full-text |
| 954 Fujii Y. Effects of diltiazem compared with nicardipine on diaphragmatic fatigability in vivo. European Journal of Anaesthesiology. 2003;20(7):575-6. | No full-text |
| 955 Timar B, Serafinceanu C, Vlad A, et al. The emerging role of SGLT2 inhibitors in the treatment of type 2 diabetes. Focus on dapagliflozin. Romanian Journal of Diabetes Nutrition & Metabolic Diseases. 2016;23(1):113-20. | No full-text |

| | |
|---|---|
| 956 Miulescu RD, Danoiu R, Margina D, et al. Evaluation of serum vitamin D in patients with metabolic syndromeRomanian Journal of Diabetes Nutrition and Metabolic Diseases. 2010. | No full-text |
| 957 Wang CJ, Weng LH, Chou WY, et al. Extracorporeal shock wave therapy enhances early tendon-bone healing and reduces bone tunnel enlargement in hamstring autograft anterior cruciate ligament reconstruction. Am J Sports Med. 2011;20:1-5. | No full-text |
| 958 Randell DJ, Byars A, Williams F, et al. Glyconutrient Supplementation in Patients with Myasthenia Gravis. Journal of Alternative and Complementary Medicine. 2008 . | No full-text |
| 959 Ghali GZ, Ghali MG, Ghali EZ. Hybrid surgical endovascular theatres in the treatment of arteriovenous malformations.World neurosurgery.2019. | No full-text |
| 960 Sain M, Ljutic D, Kovacic V, et al. Individualization of nadroparin doses inhemodialyzed patients. Ther Apher Dial. 2011;15(5):511 | No full-text |
| 961 El Mokhtari N E, Arlt A, Meissner A, et al. Inotropic therapy for cardiac low output syndrome: comparison of hemodynamic effects of dopamine/dobutamine versus dopamine/dopexamine. Eur J Med Res, 2008, 13(10): 459-463. | No full-text |
| 962 Di Lorenzo E, Sauro R, Varricchio A, et al. Long-Term outcome of drug-eluting stents compared with bare metal stents in ST-segment elevation myocardial infarction: results of the paclitaxel- or sirolimus-eluting stent versus bare metal stent in Primary Angioplasty (PASEO) Randomized Trial. Circulation. 2009;120(11):964-972. | No full-text |
| 963 Ghali G Z, Ghali M G Z, Ghali E Z. Multimodal treatment and microsurgical resection of basal ganglionic and thalamic arteriovenous malformations. World Neurosurg. 2019. | No full-text |
| 964 Shebak SS, Whitham MD, Snyder AD, et al. Potential for Increase in Gabapentin Associated Complications with Its Increased Availability. Subst Abus. 2015. | No full-text |
| 965 Gilder D A, Geisler J R, Luna J A, et al. A randomized trial of motivational interviewing for the prevention of underage drinking in American Indian adolescents. Journal of substance abuse treatment. 2017: S0740-5472 (17) 30170-8. | No full-text |
| 966 Watters K, Wragg G, Van Renen J. Reconstitution of Plenaxis (Abarelix) 100 mg for Injection Is More Effective With a Vortex-Like Mixer Than When Performed Manually. Journal of Pharmacy Practice, 2009, 22(4). | No full-text |
| 967 Bishay S N G. Reconstruction of acute closed traumatic extensor hallucis longus tendon rupture in adolescents with spastic cerebral palsy. Journal of children's orthopaedics, 2011, 5(3): 239-239. | No full-text |
| 968 Altinel L, Kose K C, Ergan V. Shed blood transfusion and its effect on postoperative fever: a comparative study. Archives of Orthopaedic and Trauma Surgery, 2010, 130(6): 717-717. | No full-text |
| 969 Iwatani S, Mizobuchi M, Tanaka S, et al. Surfactant lavage therapy for respiratory deterioration in extremely premature infants. Pediatr Int. 2013;55(2):263. | No full-text |
| 970 Knobloch K, Schreibmueller L, Jagodzinski M, et al. Tendon and paratendon Achilles microcirculation in eccentric training and an Achilles wrap in insertional and mid-portion tendinopathy--a randomized trial. Br J Sports Med. 2007;41(3):184. | No full-text |

| | |
|---|---|
| 971 Xiaowei Z, Zhenhua L, Yeqing Y, et al. Testosterone therapy improves psychological distress and health-related quality of life in Chinese men with symptomatic late-onset hypogonadism patients. Aging Male. 2013;16(2):79. | No full-text |
| 972 Fujii Y, Tanaka H, Ito M. Treatment of vomiting after paediatric strabismus surgery with granisetron, droperidol, and metoclopramide. Ophthalmologica, 2002, 216(5). | No full-text |
| 973 El James Glicio. Vitamin D Level of Mild and Severe Elderly Cases of COVID-19: A Preliminary Report. Social Science Research Network. 2020. | No full-text |
| 974 Chauhan S, Saxena N, Rao BH, Singh RS, Bhan A. A comparison of esmolol and diltiazem for heart rate control during coronary revascularisation on beating heart. Ann Card Anaesth. 2000;3(1):28-31. | No full-text |
| 975 Medeiros JFP, Borges MVO, Soares AA, et al. Association of Vitamin D Supplementation in Cardiorespiratory Fitness and Muscle Strength in Adult Twins: A Randomized Controlled Trial. Int J Sport Nutr Exerc Metab. 2022;32(1):2-7. | No full-text |
| 976 Zhang GL, Shi ZJ, Hao P, et al. The clinical application of an innovative tracheotomy fixation belt in critically ill patients. Technol Health Care. 2021. | No full-text |
| 977 Legrand FD, Polidori G, Beaumont F, et al. Whole-Body Cryotherapy as an Innovative Treatment for COVID 19-Induced Anosmia-Hyposmia: A Feasibility Study. J Integr Complement Med. 2022;28(3):e284-e288. | No full-text |
| 978 Harraa S Mohammed-Salih, Hayder F Saloom. Gingival Crevicular Fluid Flow Rate as an Indicator for Optimum Orthodontic Force: A Split-Mouth, Randomized Control Trial, J Res Med Dent Sci, 2021, 9 (4):48-59. | No full-text |
| 979 Ajayi, Sunday A. Concomitant symptomatic thoracolumbar and lower lumbar disc herniations: Strategies for treatment and outcomes .(2018): 112-112. | No full-text |
| 980 Piper SN, Beschmann RB, Mengistu A, et al. Postoperative analgosedation with S(+)-ketamine decreases the incidences of postanesthetic shivering and nausea and vomiting after cardiac surgery. Med Sci Monit. 2008;14(12):PI59-PI65. | No full-text |
| 981 Röhm KD, Riechmann J, Boldt J,et al. Total intravenous anesthesia with propofol and remifentanil is associated with a nearly twofold higher incidence in postanesthetic shivering than desflurane-fentanyl anesthesia. Med Sci Monit. 2006;12(11):CR452-CR456. | No full-text |
| 982 Akther MJ, Nasar S, Khanam N. Role of LDH as Prognostic Biochemical Marker for Breast Cancer Among Poor Patients, a Study at Rural Hospital. Indian J Surg. 2013;75(3):250. | No full-text |
| 983 Saitoh Y, Fujii Y, Ueki M, et al. Accelographic and mechanical post-tetanic count and train-of-four ratio assessed at the great toe. Eur J Anaesthesiol. 1998;15(6):649-655. | No full-text |
| 984 The clinical and laboratory features in Turkish systemic sclerosis patients: a single-center experience. Rheumatol Int. 2012;32(4):1043. | No full-text |
| 985 Effects of enflurane and propofol on seizure and recovery profiles in electroconvulsive therapy. Middle East J Anaesthesiol. 2012;21(4):659. | No full-text |
| 986 Hüttner I, Boldt J, Haisch G,et al. Influence of different colloids on molecular markers of haemostasis and platelet function in patients undergoing major abdominal surgery. Br J Anaesth. 2000;85(3):417-423. | No full-text |

| | |
|---|---|
| 987 Mayer J, Boldt J, Schöllhorn T, et al. Semi-invasive monitoring of cardiac output by a new device using arterial pressure waveform analysis: a comparison with intermittent pulmonary artery thermodilution in patients undergoing cardiac surgery. Br J Anaesth. 2007;98(2):176-182. | No full-text |
| 988 Suttner SW, Piper SN, Lang K, et al. Cerebral effects and blood sparing efficiency of sodium nitroprusside-induced hypotension alone and in combination with acute normovolaemic haemodilution. Br J Anaesth. 2001;87(5):699-705. | No full-text |
| 989 Reuben SS, Ekman EF. The effect of cyclooxygenase-2 inhibition on analgesia and spinal fusion. J Bone Joint Surg Am. 2005;87(3):536-542. | No full-text |
| 990 Abu-Omar AA. Prevention of postpartum hemorrhage, safety and efficacy. Saudi Med J. 2001;22(12):1118-1121. | No full-text |
| 991 Leth H, Kroustrup JP, Larsen JF,et al. The effect of weight loss after gastric banding on the molecular distribution of serum adiponectin. Eur J Endocrinol. 2008;159(3):357. | No full-text |
| 992 Quinonez R, Stearns SC. Issues and early evidence for the economic evaluation of the effects of periodontal therapy on pregnancy outcomes. J Periodontol. 2008;79(2):203-206. | No full-text |
| 993 Wu YW, Shiau JM, Hong CC, et al. Intrathecal midazolam combined with low-dose bupivacaine improves postoperative recovery in diabetic mellitus patients undergoing foot debridement. Acta Anaesthesiol Taiwan. 2005;43(3):129-134. | No full-text |
| 994 Piper SN, Kiessling AH, Suttner SW, et al. Prevention of atrial fibrillation after coronary artery bypass graft surgery using a potassium-magnesium-aspartate solution (Inzolen®). Thorac Cardiovasc Surg. 2007;55:418–23. | No full-text |
| 995 Khamees RE, Kishk EA, Mahmoud SS, et al. Effects of the levonorgestrel-releasing intrauterine system versus the copper intrauterine device on uterine artery Doppler indices. Eur J Contracept Reprod Health Care. 2022;27(1):23-27. | No full-text |
| 996 Sato Y, Kuno H, Kaji M, et al. Effect of ipriflavone on bone in elderly hemiplegic stroke patients with hypovitaminosis D. Am J Phys Med Rehabil. 1999;78(5):457-463. | No full-text |
| 997 Bevilacqua L, Liani G, Castronovo G, Costantinides F. Clinical and spectrophotometric evaluation after chlorhexidine use in periodontal flap surgery: A prospective randomized clinical trial. Am J Dent. 2016;29(2):75-80. | No full-text |
| 998 Dexmedetomidine and Postoperative Shivering: A Randomized Placebo Control Study | No full-text |
| 999 Interaction study between finasteride and tamsulosin in healthy young male subjects. Clin Drug Investig. 2014;34(1):81. | No full-text |
| 1000 Numazaki M, Fujii Y. Subhypnotic dose of propofol for the prevention of nausea and vomiting during spinal anaesthesia for caesarean section. Anaesth Intensive Care. 2000;28(3):262-265. | No full-text |
| 1001 Shang E, Weiss C, Post S, et al. The influence of early supplementation of parenteral nutrition on quality of life and body composition in patients with advanced cancer. JPEN J Parenter Enteral Nutr. 2006;30(3):222-230. | No full-text |
| 1002 Sabapathy V. A, P. Thilaak, S. S. Gopal, et al.Endotracheal intubation without muscle relaxants in children undergoing cleft lip, palate and alveolar surgery. A comparative study of sevoflurane and propofol. J Clin Diagn Res 5 (2011): 1421-5. | No full-text |

| | |
|---|---|
| 1003 Asgary S,M. J. Eghbal. A clinical trial of pulpotomy vs. root canal therapy of mature molars. Journal of dental research 89.10 (2010): 1080-1085. | No full-text |
| 1004 Piper SN, Röhm KD, Boldt J, et al. Inspired oxygen fraction of 0.8 compared with 0.4 does not further reduce postoperative nausea and vomiting in dolasetron-treated patients undergoing laparoscopic cholecystectomy. Br J Anaesth. 2006;97(5):647-653. | No full-text |
| 1005 Boldt J, Weber A, Mailer K, et al. Acute normovolaemic haemodilution vs controlled hypotension for reducing the use of allogeneic blood in patients undergoing radical prostatectomy. Br J Anaesth. 1999;82(2):170-174. | No full-text |
| 1006 Boldt J, Brosch Ch, Röhm K,et al. Comparison of the effects of gelatin and a modern hydroxyethyl starch solution on renal function and inflammatory response in elderly cardiac surgery patients. Br J Anaesth. 2008;100(4):457-464. | No full-text |
| 1007 Suttner S, Boldt J, Mengistu A,et al. Influence of continuous perioperative beta-blockade in combination with phosphodiesterase inhibition on haemodynamics and myocardial ischaemia in high-risk vascular surgery patients. Br J Anaesth. 2009;102(5):597-607. | No full-text |
| 1008 Piper SN, Beschmann R, Mengistu A, et al. Assessment of recovery, dreaming, hemodynamics, and satisfaction in postcardiac surgery patients receiving supplementary propofol sedation with S(+)-ketamine. Minerva Anestesiol. 2009;75(6):363-373. | No full-text |
| 1009 Uflacker R, Schönholz C, Papamitisakis N,et al. Interim report of the SENTIS trial: cerebral perfusion augmentation via partial aortic occlusion in acute ischemic stroke. J Cardiovasc Surg (Torino). 2008;49(6):715-721. | No full-text |
| 1010 Hurlstone DP, Kiesslich R, Thomson M, et al. Confocal chromoscopic endomicroscopy is superior to chromoscopy alone for the detection and characterisation of intraepithelial neoplasia in chronic ulcerative colitis. Gut. 2008;57(2):196-204. | No full-text |
| 1011 Lv F, Liu X, Wang B, et al. S-1 monotherapy as second line chemotherapy in advanced gastric cancer patients previously treated with cisplatin/infusional fluorouracil. Int J Clin Exp Pathol. 2014;7(7):4274-4279. | Wrong study design |
| 1012 Pozzilli P, Raz I, Peled D, et al. Evaluation of long-term treatment effect in a type 1 diabetes intervention trial: differences after stimulation with glucagon or a mixed meal. Diabetes Care. 2014;37(5):1384-1391. | Wrong study design |
| 1013 Fujii Y. Pretreatment with flurbiprofen axetil and venous occlusion to reduce pain during injection of propofol. Can J Anaesth. 2004;51(10):1047-1048. | Wrong publication type |
| 1014 Nakano M, Fujii Y. Prevention of nausea and vomiting after dental surgery: a comparison of small doses of propofol, droperidol, and metoclopramide. Can J Anaesth. 2003;50(10):1085. | Wrong publication type |
| 1015 Numazaki M, Fujii Y. Antiemetic efficacy of propofol at small doses for reducing nausea and vomiting following thyroidectomy. Can J Anaesth. 2005;52(3):333-334. | Wrong publication type |
| 1016 Moshonov J, Peretz B, Brown T, et al. Cleaning of the root canal using Nd:YAP laser and its effect on the mineral content of the dentin. J Clin Laser Med Surg. 2004;22(2):87-89. | In vitro |
| 1017 Luo W, Ma L, Wen Q, et al. Analysis of the TCR alpha and beta chain CDR3 spectratypes in the peripheral blood of patients with Systemic Lupus Erythematosus. J Autoimmune Dis. 2008; 29;5:4. | In vitro |

| | |
|---|---|
| 1018 de Meireles DA, de Brito TC, Marques AA, et al. Micro-computed tomography evaluation of apical transportation and centring ability of Reciproc and WaveOne systems in severely curved root canals. Int Endod J. 2015;48(8):814. | In vitro |
| 1019 Boldt J, Kling D, von Bormann B,et al. Homologes Frischplasma in der Herzchirurgie. Mythos oder Notwendigkeit . Anaesthesist. 1989;38(7):353-359. | Foreign language |
| 1020 MULLER. Hidrotische ektodermale Dysplasie. Dermatol Wochenschr. 1959;139(9):211-212. | Foreign language |
| 1021 Kim SJ. Placental site trophoblastic tumour. Best Practice & Research Clinical Obstetrics & Gynaecology. 2003;17(6):969-84. | Foreign language |
| 1022 Mao Y, Zhao J, Gao Y.Right beauty holds the mi organism and propofol for elderly patients with hip fracture surgery ICU sedation effect of comparative study. Zhonghua yi xue za zhi, 2015, 95(19): 1493-1495. | Foreign language |
| 1023 Kim SH, Kim KP, Heo ST, et al. Experience of therapeutic plasma exchange in patients with severe fever with thrombocytopenia syndrome. The Korean Journal of Blood Transfusion. 2014;25(3):211-7. | Foreign language |
| 1024 Piper SN, Haisch G, Kumle B, et al. Einfluss der Esmolol- und der Natrium-Nitroprussid-induzierten kontrollierten Hypotension auf die hepatozelluläre Integrität bei endonasalen Nebenhöhleneingriffen . Anasthesiol Intensivmed Notfallmed Schmerzther. 2003;38(12):781-786. | Foreign language |
| 1025 Fujii Y, Tanaka H, Toyooka H. Effects of laryngeal mask airway on circulation and on incidence of postoperative sore throat and hoarseness. Masui. The Japanese Journal of Anesthesiology. 1993;42(11):1659-62. | Foreign language |
| 1026 Fujii Y, Tanaka H. Postoperative anti-emetic effects of low dose droperidol. Masui. The Japanese Journal of Anesthesiology. 1993;42(5):694-7. | Foreign language |
| 1027 Roca Biosca A, Anguera Saperas L, García Grau N, et al. Prevención de la neumonía asociada a la ventilación mecánica: estudio comparativo de dos métodos de higiene oral. Enferm Intensiva. 2011;22(3):104-111. | Foreign language |
| 1028 Rissoul K, Madani M, Nougier C, et al. Évaluation de l'intérêt du test de confirmation avec une forte concentration d'héparine en Elisa dans la thrombopénie induite par l'héparine . Ann Biol Clin (Paris). 2010;68(5):555-560. | Foreign language |
| 1029 Piper SN, Kumle B, Röhm KD, et al. Einfluss der extrakorporalen Zirkulation (EKZ) auf die natriuretischen Peptide ANP und BNP. Ein Vergleich zwischen langen und kurzen EKZ-Zeiten sowie Off-Pump-Eingriffen . Anasthesiol Intensivmed Notfallmed Schmerzther. 2003;38(7):463-469. | Foreign language |
| 1030 Ito T, Okubo Y, Roth A. Efficacy of mirtazapine for appetite loss and nausea of the cancer patient--from clinical experience in Memorial Sloan-Kettering Cancer Center. Gan to kagaku ryoho. Cancer & chemotherapy. 2009;36(4):623-6. | Foreign language |
| 1031 Song RH, Kim DH. The effects of foot reflexion massage on sleep disturbance, depression disorder, and the physiological index of the elderly. Journal of Korean Academy of Nursing. 2006 ;36(1):15-24. | Foreign language |
| 1032 Drózdz M, Strzelczak Z, Jendryczko A. Palenie papierosów i palenie bierne w czasie ciazy i po porodzie: wpływ na mase urodzeniowa, długość okresu laktacji oraz stezenie kotyniny w mleku matki i wydalanie kotyniny w moczu dziecka . Ginekol Pol. 1988;59(9):528-533. | Foreign language |

| | |
|---|---|
| 1033 Boldt J, Zickmann B, Thiel A, et al. Hyperosmolarer Volumenersatz in der Herzchirurgie . Anaesthesist. 1990;39(8):412-419. | Foreign language |
| 1034 Boldt J, Kling D, Zickmann B,et al. Hämodynamische Effekte verschiedener Hydroxyäthylstärke-Lösungen bei kardiochirurgischen Patienten . Anaesthesist. 1990;39(1):6-12. | Foreign language |
| 1035 Boldt J, von Bormann B, Kling D, et al. Volumenersatz mit einem neuen Hydroxyäthylstärke-Präparat (3% HAS 200/0.5) in der Herzchirurgie . Infusionsther Klin Ernahr. 1986;13(3):145-151. | Foreign language |
| 1036 Pavel L, Pavel S. Bruikbaarheid micronutriënten bij behandeling van parodontitis . Ned Tijdschr Tandheelkd. 2010;117(2):103-106. | Foreign language |
| 1037 Triem JG, Röhm KD, Boldt J,et al. Propofol-Anästhesie mittels Target-Controlled-Infusion (TCI). Ein Vergleich der Verfahren: Optimated-Target-Controlled Infusion (OTCI) und Manually-Controlled-Infusion (MCI) . Anasthesiol Intensivmed Notfallmed Schmerzther. 2006;41(3):150-155. | Foreign language |
| 1038 Piper SN, Triem JG, Röhm KD, et al. Ein Vergleich der ProSeal(R)-Larynxmaske mit der konventionellen endotrachealen Intubation bei Laparoskopien in der Gynäkologie . Anasthesiol Intensivmed Notfallmed Schmerzther. 2004;39(3):132-137. | Foreign language |
| 1039 Triem JG, Röhm KD, Boldt J, et al. Propofolapplikationssysteme: Handhabung, Kreislaufverhalten und Propofolverbrauch . Anaesthesist. 2009;58(3):231-239. | Foreign language |
| 1040 Röhm KD, Piper SN, Schöllhorn TA,et al. Injektionsschmerz unter Propofol-MCT/LCT und Propofol-LCT - Vergleich einer Prophylaxe mit Lidocain . Anasthesiol Intensivmed Notfallmed Schmerzther. 2003;38(10):643-647. | Foreign language |
| 1041 Mohamadi, Akram, et al.The Effect Of Family Centered Education Based On Health Belief Model On The Absence Of School And Hospitalization Of Children With Asthma: A Clinical Trial Study. (2020): 420-429. | Foreign language |
| 1042 CHO J W, Youn J, Choi M G, et al. Assessing nutritional status in outpatients after gastric cancer surgery: a comparative study of five nutritional screening tools. Korean Journal of Community Nutrition, 2021: 280-295. | Foreign language |
| 1043 LEE D Y, KIM D S, SOHN D S, et al. Attenuated Ceramide-induced Neuronal Apoptosis by Acanthopanax senticous. Korean Journal of Physical Anthropology.2003: 267-277. | Foreign language |
| 1044 Achkasov EE, Ul'ianov AA, Bezuglov ÉN, et al. Autoplasma enriched with platelet derived growth factor in surgery and traumatology. Khirurgiia (Mosk). 2014;(9):48-54. | Foreign language |
| 1045 Geiges B, von Falck C, Knobloch K, et al. Biodegradierbare Schraube vs. einer Press-fit-Verankerung für VKB-Rekonstruktionen. Eine prospektive randomisierte Studie. Unfallchirurg. 2014;117(8):755. | Foreign language |
| 1046 SORLEA S, COROS M F, GEORGESCU R, et al. BIOPSIA NODULULUI SANTINELA IN CANCERUL DE COLON. Clujul Medical. 2011. | Foreign language |
| 1047 Rakhmatullo A, Rakhmonov, MO, et al. Cardioembolic stroke and its course in patients with heart diseaseBulletin of Avicenna. 2015. | Foreign language |

| | |
|---|---|
| 1048 Saodat M, Azimova, AD, Dustov, Rustam Abdusamadovich, Tursunov,Chronic hepatitis C in TajikistanBulletin of Avicenna. 2015. | Foreign language |
| 1049 Lux EA, Gendolla A. Chronische Migräne richtig diagnostizieren und behandeln. Schmerzmedizin. 2018:34-38. | Foreign language |
| 1050 Rodríguez A, Santaera O, Lugones M, et al. Clinico-angiographic correlations in transmural acute myocardial infarction treated with thrombolytic agents. Medicina (B Aires). 1988;48(2):132-140. | Foreign language |
| 1051 Maria Andreevna, Asmanova, Valentinovna N, et al. Comparative Aspects of the Effectiveness of Microbiological Methods for Determining the Massity of Bacterium Exposure in Patients with Tuberculosis and Combined Pathology (HIV Infection and Tuberculosis)Modern Science: Topical Problems of Theory and Practice. Series: Natural and Technical Sciences. 2020. | Foreign language |
| 1052 Schwerthöffer D, Pajonk FG.Comparison of sublingual and intravenous administration of lorazepam in psychiatric emergencies in emergency medical services. Anaesthesist. 2019;68(2):83-89. | Foreign language |
| 1053 Faig AO, Mirzazade. Comparison of the course of the early period after Fontaine's operation in children of different age groupsBulletin of Avicenna. 2015. | Foreign language |
| 1054 Vadim A, Byvaltsev, Ivan A, et al. Comparison of the Results of Total Arthroplasty With Discover Prosthesis and Anterior Neck Spondilodesis in Surgical Treatment of Degenerative Diseases of the Neck Intervertebral Discs: Metayzdonalysis of the WaysTraumatology and Orthopedics of Russia. 2018. | Foreign language |
| 1055 Haouichat H, Benali R, Benyounes A,et al. Contrôle de l'asthme chez l'adulte en Algérie. Comparaison avec les autres pays d'Afrique du Nord et du Moyen Orient. Revue Des Maladies Respiratoires. 2020;37(1):15-25. | Foreign language |
| 1056 Jendryczko A, Drózdz M, Tomala J, et al. Copper and zinc concentrations in normal and malignant tissues of ovary, uterus and cervix. Ginekol Pol. 1985;56(11):677-681. | Foreign language |
| 1057 Anna B, Lopatina. Correction of Microcirculation Disorders with Capillarotherapy in Patients with Type I Diabetes and Assessment of its EfficiencyJournal of Scientific Articles Health and Education in the XXI Century. 2016. | Foreign language |
| 1058 Podkamenev VV, Pikalo IA. The criteria for choose of treatment method of children with spleen injuries. Khirurgiia (Mosk). 2014;(11):34-40. | Foreign language |
| 1059 Represas-Carrera F. Cumplimiento del tratamiento por vía inhalatoria en pacientes con enfermedad pulmonar obstructiva crónica.Enfermería Universitaria.2014: 128-131. | Foreign language |
| 1060 Piper SN, Röhm KD, Papsdorf M, et al. Dolasetron reduces pain on injection of propofol.Anasthesiol Intensivmed Notfallmed Schmerzther. 2002;37(9):528-531. | Foreign language |
| 1061 Oksana Aleksandrovna, Soldatova,Dynamics of Antiaggregative Activity of the Vascular Wall in Patients with Arterial Hypertension Degree 1-2 with Metabolic Syndrome Receiving Lisinopril in a Treatment ComplexBulletin of the Russian University of People's Friendship. Series: Ecology and Life Safety. 2015. | Foreign language |
| 1062 Montes LA, Valenzuela MJ. Effectiveness of low back pain treatment with acupuncture. Biomedica. 2017;38(0):54-60. | Foreign language |

| | |
|---|---|
| 1063 Kim H S, Kim S. Effects of an integrated self-management program on self-management, glycemic control, and maternal identity in women with gestational diabetes mellitus. Journal of Korean Academy of Nursing, 2013, 43(1): 69-80. | Foreign language |
| 1064 Marra M L, Valenzano A, Ruberto M, et al. The effects of overweight and obesity on cognitive functions and psychological well-being. Acta Medica Mediterranea.2017, 33: 1225-1231. | Foreign language |
| 1065 Kim Y J, Ha J Y. The effects of visiting exercise program and telecoaching for physical activity promotion on physical fitness and quality of life in the frail elderly. Korean Journal of Adult Nursing. 2011, 23(2): 198-207. | Foreign language |
| 1066 Regina R, Khalfina, Alexander V, et al. Efficiency of Application of Physical Means of Rehabilitation in Visual FatigueProblems of Modern Pedagogical Education. 2016. | Foreign language |
| 1067 Negmatova GM, Acheva GA, Sharipova HE, et al. Efficiency of Controlled Therapy of Arterial Hypertension in Middle-Aged PersonsBulletin of Avicenna. 2016. | Foreign language |
| 1068 Sukovatykh B, Sukovatykh, Nelly M, et al. Endoprosthetics with abdominal wall lifting in the treatment of umbilical and incisional ventral hernias (with commentary)Surgery. Journal them. N. I. Pirogov. 2015. | Foreign language |
| 1069 Anna B, Lopatina. Estimation of the Efficiency of Application of Different Kinds of Capillarotherapy for Correction of Microcirculation Disorders in Patients with Type I Diabetes MellitusJournal of Scientific Articles Health and Education in the XXI Century. 2016. | Foreign language |
| 1070 Arreola-Ornelasa H, Lemus-Carmonab EA, Camacho-Corderoc LM. Evaluación económica de ranibizumab en pacientes adultos con degeneración macular relacionada con la edad (DMRE) exudativa en México. Revista Mexicana de Oftalmología.2014: 5-15. | Foreign language |
| 1071 Rolando NS, Natalia AC, Alejandra MM, et al. Evaluation of Direct Antibiograma from the Bottle Blood Culture with Vitek 2 C System: Its Usefulness in ClinicalRevista Argentina de Microbiología. 2012. | Foreign language |
| 1072 Noriko Y, Misuzu N. Expert critical care nurses＊ reflection to assist patients＊ families with surrogate decision-making regarding life-sustaining treatmentJournal of Japanese Association for Emergency Nursing. 2017. | Foreign language |
| 1073 Abidin A, Tasnim T, Fatmawati F, et al. Faktor risiko wasting dalam penerapan full day school pada anak di paud pesantren ummusabri kendari. Jurnal Penelitian Kesehatan" SUARA FORIKES"(Journal of Health Research" Forikes Voice"), 2018, 9(4): 263-268. | Foreign language |
| 1074 Yulia S, Rafikova, Tatyana V, Saprina, et al. Features of Eating Behavior in Children and Adolescents Born PrematureModern Problems of Science and Education. 2015. | Foreign language |
| 1075 Umeda K, Rakhimova OI, Kosimov. Features of the clinical and immunological course of atopic dermatitis in childrenBulletin of Avicenna. 2015. | Foreign language |
| 1076 Sherali R, Sultonov AM, Sattorov. Features of the tactics of surgical treatment of congenital hydronephrosis caused by an aberrant vesselBulletin of Avicenna. 2015. | Foreign language |
| 1077 Saburov SK. Frequency of Incidence and Complications in Orthopedic Dentistry During Prosthetics with Fixed Structures of Patients with Generalized PathologyBulletin of Avicenna. 2016. | Foreign language |

| | |
|---|---|
| 1078 Sergeyev OV, Bosh'ian RE, Barinsky IF. High-Productive Sequencing in Diagnostics and Prevention of Infection of Simple Herpes (Herpesviridae, Alphaherpesvirinae, Simplexvirus, Human Alphaherpesvirus 1). Vopr Virusol. 2020;65(3):126-131. | Foreign language |
| 1079 Tardieu G, Tabary JC, Tardieu C, et al. Rétraction, hyperextensibilité et "faiblesse" de l'I.M.C. expressions apparemment opposées d'un même trouble musculaire. Conséquences thérapeutiques.Rev Chir Orthop Reparatrice Appar Mot. 1971;57(7):505-516. | Foreign language |
| 1080 Nasyr A, Rasulov, Karimkhon M, et al. Iatrogenic damage to the bile ductsBulletin of Avicenna. 2015. | Foreign language |
| 1081 Patrikeev AV, Rudman VI, Maksimkin DA, et al. Immediate and long-term results of endovascular treatment of patients with postinfarction cardiosclerosis. Khirurgiia (Mosk). 2015;(1):17-22. | Foreign language |
| 1082 Rabant M, Calvani J, Terada M, et al. Immunofluorescence multiparamétrique in situ: vers l'amélioration du phénotype de l'infiltrat cellulaire au cours du rejet d'allogreffe rénale. Néphrologie & Thérapeutique. 2019; 15: S43-S52. | Foreign language |
| 1083 Alena D, Donika, Shogik G, et al. Implementation of the Professional Role of the Therapist in the Mirror of Paradoxic MedicineEconomic and Humanitarian Research of Regions. 2015. | Foreign language |
| 1084 Azamat K, Kade, Sergey A, et al. Implementation of the Visibility Principle in Teaching Pathological PhysiologyInternational Journal of Experimental Education. 2015. | Foreign language |
| 1085 Larisa R, Legotkina, Anna B, et al. Influence of Physical Loading on the Student OrganismInternational Research Journal. 2016. | Foreign language |
| 1086 Vsevolod L, Adzhienko, Oksana V, et al. Interactive Games as One of the Forms of Productive Pedagogical Technologies in Pharmaceutical EducationJournal of Scientific Articles Health and Education in the XXI Century. 2016. | Foreign language |
| 1087 Anna B, Lopatina Interdisciplinary Approach in Training General Practice DoctorsModern Problems of Science and Education. 2016. | Foreign language |
| 1088 Liana S, Karapetyan. Interdisciplinary aspects of the diagnosis and treatment of snoring and obstructive sleep apnea syndromeRMJ . 2015. | Foreign language |
| 1089 Larisa R, Legotkina. Intoxication During Weight ReductionInternational Research Journal. 2016. | Foreign language |
| 1090 Natalia A, Malofeevskaya, Olga V, et al. Intra-Regional Differences in the Impact of Environmental and Economic Factors on Child Oncology in RussiaJournals of Higher Educational Institutions. Povolga Region. Medical Sciences. 2017. | Foreign language |
| 1091 Damjanović A, Damjanović A, Stojanović R, et al. Kliničko terapijske specifičnosti shizofrenije s prodromima i ranim početkom-model rizičnog mentalnog stanja i shizofrene vulnerabilnosti. Jahr–European Journal of Bioethics, 2013, 4(2): 747-758. | Foreign language |
| 1092 Coltescu F Klara B, Ioana H, et al. L * utilisation de la proteine p16ink4a dans le diagnostic des cinRomanian Journal of Functional & Clinical Macro- & Microscopical Anatomy & of Anthropology. 2012. | Foreign language |
| 1093 Rakhmatullaev, Karimdzhon P, Artykov, et al. Laparoscopic Simultaneous Operations for Combined Surgical Diseases of the Organs of the Abdominal CavityBulletin of Avicenna. 2016. | Foreign language |

| | |
|---|---|
| 1094 Pavel I, Lukyanenok, Valentina M, et al. Magnetic Resonance Brain Tomography in Patients with Multiple Sclerosis on the Background of Treatment with Immunomodulating DrugsSuccesses of Modern Natural Science. 2015. | Foreign language |
| 1095 Volkova NN. The main causes and principles of treatment for constipation in the elderlyRussian Medical Journal. Medical Review. 2015. | Foreign language |
| 1096 Nikolay M, Kondyrev, Sergey S, et al. Method for early diagnosis of rotator cuff injuriesBulletin of the Russian University of People's Friendship. Series: Medicine. 2015. | Foreign language |
| 1097 Saad A, Fadel. Modern problems of health preservation and preservation of moral health of students in the educational space of the Republic of IraqKazan Pedagogical Journal. 2015. | Foreign language |
| 1098 Yakupova, Lilia A, Khasanova, et al. Morphofunctional Characteristics of Thymus on the Background of Oral Administration of Nanodisperse Titanium DioxideBulletin of the Bashkir State Pedagogical University IM. M. Akmulla. 2017. | Foreign language |
| 1099 Maltese A, Gallai B, Romano P, et al. Motion sickness in childhood migraine. Acta Medica Mediterranea, 2017, 33: 1241. | Foreign language |
| 1100 Yu V, Kokorev, Svetlana V, et al. A New Method of Surgical Treatment of Severe BlepharoptosisInternational Research Journal. 2015. | Foreign language |
| 1101 Natalya V, Efimenko, Tatyana S, et al. Non-drug technologies for the correction of psychoemotional disorders in irritable bowel syndromeBulletin of Avicenna. 2015. | Foreign language |
| 1102 Musoev D S. Osteosynthesis in the treatment of diaphyseal fractures of long bones in children. Vestnik avitsenny, 2015 (3): 37. | Foreign language |
| 1103 Elmira A, Khachaturova, Alexander V, et al. Peculiarities of Anti-Coagulant Therapy in Patients with Colorectal Cancer Depending on Hemostasis DisordersColoproctology. 2015. | Foreign language |
| 1104 Namuna I, Mustafakulova, Nazirov. Peculiarities of Psychophenotypic Portrait and Separate Clinical-Pathogenetic Mechanisms of Chronic Pulmonary Heart Development in Patients with COPD Depending on the Type of Higher Nervous ActivityBulletin of Avicenna. 2016. | Foreign language |
| 1105 Alexander S, Dukhanin. Ph Fundamentals of Topic Medicinal Preparation: Optimal Value Selection and the Role of the Buffer SystemBulletin of Dermatology and Venerology. 2016. | Foreign language |
| 1106 Illarioshkin SN, Maxim A, Domashenko, et al. Possibilities of Anxiety Disorders Treatment with the Use of TenotenNervous Diseases. 2018. | Foreign language |
| 1107 Andrey D, Kaprin, Dmitry V, Sidorov,et al. Possibilities of Prediction and Surgical Prevention of Acute Liver Insufficiency after Extensive Liver Resections for Primary and Metastatic TumorsOncology. Journal Them. P A Hercena. 2016. | Foreign language |
| 1108 Solov'ev IA, Kolunov AV. Postoperative intestinal paresis: the problem of abdominal surgery. Khirurgiia (Mosk). 2013;(11):46-52. | Foreign language |

| | |
|---|---|
| 1109 Park H J, Shin H N, Shin J Y. Prescribing Patterns of Codeine among Children under Aged 12 in Korea. Korean Journal of Clinical Pharmacy. 2015: 273-279. | Foreign language |
| 1110 Nathal E, Sierra-Honigmann M R, de la Cabada Cortés F J. Pyrogenic activity in the sera of febrile patients mediated by endogenous pyrogen and activators of adenyl cyclase. Revista de investigacion clinica; organo del Hospital de Enfermedades de la Nutricion, 1984, 36(2): 125-131. | Foreign language |
| 1111 Paulo HBM, Simone CTM, Eveline TP, et al. Quality of life of elderly caregivers of link to the Family Health Program - Teixeiras, MGRevista Brasileira de Geriatria e Gerontologia. 2011. | Foreign language |
| 1112 Yulia A, Nazaryants, Stanislav I, et al. Quality of Life of Patients After Laparoscopic Hernioplasty of Postoperative Ventral HerniaModern Problems of Science and Education. 2015. | Foreign language |
| 1113 Rationale for the use of capillary therapy in patients with type I diabetes mellitus and the effect of capillary therapy on microcirculation parameters | Foreign language |
| 1114 Vera V, Kiryanova, Natalya V, et al. Research of the Dynamics of the Quality of Life of Patients with the Use of Bioresonance Therapy in the Complex Treatment of HypothyrosisBulletin of Avicenna. 2016. | Foreign language |
| 1115 Anna B, Lopatina. Restoration of Microcirculation of FightersInternational Research Journal. 2016. | Foreign language |
| 1116 Zaur K, Shugushev, Daniil A, et al. Results of Bioabsorbable Vascular Endoprosthesis Implantation in Patients with Coronary Heart Disease with Concomitant Type II Diabetes MellitusCardiology and Cardiovascular Surgery. 2016. | Foreign language |
| 1117 Daria S, Kaskaeva, Marina M, et al. Results of In-Depth Medical Examination of Students of the 1st Course of the Krasnoyarsk State Medical UniversityIn the World of Scientific Discoveries. 2016. | Foreign language |
| 1118 Nasrulla A, Shanazarov, Andrey V, et al. The risk of developing multiple primary metachronous cancer, depending on the treatment of the first tumorBulletin of Avicenna. 2015. | Foreign language |
| 1119 Valentina O, Shtumf. The role of cognitive ability of frequently ill older preschool children in the formation of ideas about orthobiosiBulletin of the Krasnoyarsk State Pedagogical University Im. V. P. Astafieva. 2015. | Foreign language |
| 1120 Nelly K, Akhkubekova, Natalya Vet al. Sanatorium rehabilitation of patients with osteopenic syndrome associated with functional hyperprolactinemiaBulletin of Avicenna. 2019. | Foreign language |
| 1121 Malokhat, Muminova, Shukhrat F, et al. Sanatorium-and-spa rehabilitation of elderly patients with coronary heart diseaseBulletin of Avicenna. 2015. | Foreign language |
| 1122 Same' Rakhmonberdievich, Rasulov DZ, Zikiryakhodzhaev, et al. Skin Cancer Developing from ScarsBulletin of Avicenna. 2016. | Foreign language |
| 1123 Sergey Nh, Gontarev, Inna S, et al. Some Aspects of Examination of Children and Adolescents with Orthodontic Diseases in the Background of the General Somatic StatusScientific News of the Belgorod State University. Series: Medicine. Pharmacy. 2015. | Foreign language |
| 1124 Mizhgona I, Sharipova, Munira Det al. The state of the autonomic nervous system in pregnant women with neurocirculatory dystonia during physical rehabilitationBulletin of Avicenna. 2015. | Foreign language |

| | |
|---|---|
| 1125 Matkassymova AT, Abdizhalil A, Bolotbekova. State of the Immune System in Children with Non-Rheumatic MyocarditisModern Problems of Science and Education. 2016. | Foreign language |
| 1126 Komildjon I, Ismoilov, Davlatov, et al. The State of the Plate Line of Hemostasis in Children of the First Year of Life with Bacterial PneumoniaBulletin of Avicenna. 2016. | Foreign language |
| 1127 Komildjon I, Ismoilov, Mavluda Met al. Structural and Functional State of the Cardiovascular System in Children with Bronchial AsthmaBulletin of Avicenna. 2016. | Foreign language |
| 1128 Alexey S, Kotov. Surgical interventions and pain relief in patients with epilepsy. Clinical lectureRMJ . 2015. | Foreign language |
| 1129 Boris Y, Alekseev, Kirill M, et al. Target Therapy in Patients with Metastatic Kidney Cancer in Real Clinical PracticeMedical Board. 2016. | Foreign language |
| 1130 Sharafidin Z, Khabibulaev, Jumaboy R, et al. To the Question About the Volume of Surgical Interventions on the Ways of the Regional Lymphotous for Malignant Tumors of the Head and NeckBulletin of Avicenna. 2016. | Foreign language |
| 1131 Röck T, Naycheva L, Willmann G, et al. Transkorneale Elektrostimulation bei Patienten mit primärem Offenwinkelglaukom. Ophthalmologe. Der Ophthalmologe. 2015;112(8):694. | Foreign language |
| 1132 Yo B, Yorov, Nuriddin D, et al. Treatment of acute cholecystopancreatitis by conjulation of the round ligament of the liverBulletin of Avicenna. 2015. | Foreign language |
| 1133 Anton V, Molochkov, Andrey D, et al. Treatment of basal cell carcinoma using photodynamic therapy and local application of the photosensitizer photolonRadiation and Risk . 2015. | Foreign language |
| 1134 Tatiana M, Tregubova, Nadezhda V, et al. Updating the Formation of a Healthy Lifestyle of a Medical College Student in Modern ConditionsKazan Pedagogical Journal. 2016. | Foreign language |
| 1135 Carrera FJ. Utilizan correctamente los inhaladores los pacientes con enfermedad pulmonar obstructiva crónica del centro de Atención Primaria Antón de Borja?. Enfermería clínica. 2015;25(1):3-8. | Foreign language |
| 1136 Sergey Valentinovich, Shkodkin, Fironov, et al. Ways of Reducing Intraoperative Blood Loss in Renal Cell Cancer Surgery Complicated by Invasion in the Lower Cave VinExperimental and Clinical Urology. 2016. | Foreign language |
| 1137 Eduard S, Temkin, Viktor I, et al. Application of Dental Prostheses from PEEK (Polyester-Etherketone) Material with Support on Implants and Teeth in Patients with Chronic Generalized PeriodontitisBulletin of Avicenna. 2016. | Duplicates |
| 1138 Liu H, Luo Z, Liu Z, et al. Atorvastatin may attenuate recurrence of chronic subdural hematoma. Frontiers in neuroscience. 2016;10:177917. | Duplicates |
| 1139 Rupasinghe CD, Kantas T, Sani R, et al. Comparison of High-Statin Therapy vs Moderate-Statin Therapy in Achieving Positive Low-Density Lipoprotein Change in Patients After Acute Coronary Syndrome: A Randomized-Control Trial. Cureus. 2021. | Duplicates |
| 1140 Ahimastos AA, Aggarwal A, Kellie MD, et al. Effect of perindopril on large artery stiffness and aortic root diameter in patients with Marfan syndrome: a randomized controlled trial. Jama. 2007 Oct 3;298(13):1539-47. | Duplicates |

| | |
|---|---|
| 1141 Omilaenko, Vorobiev, et al. The effect of therapy based on the use of ultra-low doses of antibodies to the C-terminal fragment of the beta subunit of the insulin receptor and antibodies to endothelial NO-synthase on the functional state of the liver in patients with type 2 diabetes mellitus and non-alcoholic fatty liver diseasePractical Medicine. 2015. | Duplicates |
| 1142 Mira TA, Giraldo PC, Yela DA, et al. Effectiveness of complementary pain treatment for women with deep endometriosis through Transcutaneous Electrical Nerve Stimulation (TENS): randomized controlled trial. European Journal of Obstetrics & Gynecology and Reproductive Biology. 20151;194:1-6. | Duplicates |
| 1143 Zhu LX, Ho SC, Sit JW, et al. Effects of a transtheoretical model–based exercise stage–matched intervention on exercise behaviour and quality of life in patients with coronary heart disease: a randomized controlled trial. Journal of Advanced Nursing. 2014 ;70(10):2414. | Duplicates |
| 1144 Daneshparvar N, Chu TM, Blanchard S,et al. The Effects of Clockwise and Counterclockwise Conventional and Osseodensification Drilling on the Dimensions, Density, and Biomechanical Properties of Bone. International Journal of Oral & Maxillofacial Implants. 2023;38(1). | Duplicates |
| 1145 Oostdijk EA, Kesecioglu J, Schultz MJ, et al. Effects of decontamination of the oropharynx and intestinal tract on antibiotic resistance in ICUs: a randomized clinical trial. Jama. 2014;312(14):1429-37. | Duplicates |
| 1146 Derosa G, Maffioli P, Ferrari I, et al. Effects of one year treatment of sibutramine on insulin resistance parameters in type 2 diabetic patients. Journal of Pharmacy & Pharmaceutical Sciences. 2010;13(3):378-90. | Duplicates |
| 1147 Safarinejad MR. The effects of the adjunctive bupropion on male sexual dysfunction induced by a selective serotonin reuptake inhibitor: a double-blind placebo-controlled and randomized study. BJU international. 2010;106(6):840-7. | Duplicates |
| 1148 Mengistu AM, Röhm KD, Boldt J, et al. The influence of aprotinin and tranexamic acid on platelet function and postoperative blood loss in cardiac surgery. Anesth Analg. 2008;107(2):391-397. | Duplicates |
| 1149 Zhao F, Wang Z, Yang J, et al. Low-dosage adrenaline induces transient marked decrease of blood pressure during functional endoscopic sinus surgery. Am J Rhinol. 2006;20(2):182-185. | Duplicates |
| 1150 Qian RX, Lu K. A Multicentre Randomized Controlled Trial Comparing Plating with Intramedullary Nailing for Extra-articular Distal Tibial Fractures. Injury. 2021;52(11):3548. | Duplicates |
| 1151 Liedl A, Müller J, Morina N, et al. Physical activity within a CBT intervention improves coping with pain in traumatized refugees: results of a randomized controlled design. Pain Med. 2011;12(2):234-245. | Duplicates |
| 1152 Niederhofer H, Staffen W, Mair A. A placebo-controlled study of lofexidine in the treatment of children with tic disorders and attention deficit hyperactivity disorder. J Psychopharmacol. 2003;17(1):113-119. | Duplicates |
| 1153 Sato Y, Honda Y, Umeno K, et al. The prevention of hip fracture with menatetrenone and risedronate plus calcium supplementation in elderly patients with Alzheimer disease: a randomized controlled trial. The Kurume medical journal. 2010, 57(4): 117-124. | Duplicates |

| | |
|---|---|
| 1154 Sato Y, Kanoko T, Satoh K, et al. The Prevention of Hip Fracture With Risedronate and Ergocalciferol Plus Calcium Supplementation in Elderly Women With Alzheimer Disease: A Randomized Controlled Trial. Arch Intern Med. 2005;165(15):1737-1742. | Duplicates |
| 1155 Boldt J, Brosch C, Lehmann A, et al. The Prophylactic Use of the β-Blocker Esmolol in Combination with Phosphodiesterase III Inhibitor Enoximone in Elderly Cardiac Surgery Patients. Anesthesia & Analgesia, 2004, 99(4): 1009-1017. | Duplicates |
| 1156 Sam S. Chang. Randomized trial of narrow-band versus white-light cystoscopy for restaging (second-look) transurethral resection of bladder tumors. The Journal of urology. 2015. | Duplicates |
| 1157 Mukhtar S, Ishag Adam M, Martinez-Jimenez E, et al. Transversus Abdominis Plane Block Versus Local Anesthetic Wound Infiltration for Postoperative Analgesia in Adult Patients Undergoing Hernia Repair in Daycare Procedure: A Randomized Control Trial. Cureus. 2022;14(1):e21311.doi:10.7759/cureus.21311 | Duplicates |
| 1158 Leber A, Hontecillas R, Tubau-Juni N,et al. EFFICACY, SAFETY, AND TOLERABILITY OF OMILANCOR IN A PHASE 2 RANDOMIZED, DOUBLE-BLIND, PLACEBO-CONTROLLED TRIAL OF PATIENTS WITH ULCERATIVE COLITIS. Inflamm Bowel Dis. 2022;28(Suppl 1):S112. | Wrong publication type |
| 1159 Shi Y, Wu L, Yu X, et al. ORIENT-3: A randomized, open-label, phase III study of sintilimab versus docetaxel in previously treated advanced/metastatic squamous non-small cell lung cancer (sqNSCLC). Ann Oncol. 2020;31 Suppl 7:S1428. | Wrong publication type |
| 1160 Provenzano R, Fishbane S, Wei LJ, et al. Pooled efficacy and cardiovascular (CV) analyses of roxadustat in the treatment of anemia in CKD patients on and not on dialysis. Abstract FR-OR13. Washington, DC: American Society of Nephrology. 2019 Nov 5. | Wrong publication type |
| 1161 El-Araby RE, Khalifa MA, Zoheiry MM, et al. Correction: The interaction between microRNA-152 and DNA methyltransferase-1 as an epigenetic prognostic biomarker in HCV-induced liver cirrhosis and HCC patients. Cancer Gene Ther. 2022;29(8-9):1297-1298. | Wrong study design |
| 1162 Sato Y, Kaji M, Higuchi F,et al. Changes in bone and calcium metabolism following hip fracture in elderly patients. Osteoporos Int. 2001;12(6):445-449. | Wrong study design |
| 1163 Ueshima H, Iwamoto W, Otake H. Serratus Plane Block for a Contraction of the Latissimus Dorsi Muscle. Reg Anesth Pain Med. 2016;41(3):411. | Wrong study design |
| 1164 Yu D, Liu Z, Wang H, et al. Analysis on the Effect of Different Surgical Methods on the Treatment of Senile Osteoporotic Spinal Compression Fractures and the Influencing Factors of Complications. Evid Based Complement Alternat Med. 2021;2021:1599470. | Wrong study design |
| 1165 Xu X, Liu X, Ling Q, et al. Artificial liver support system combined with liver transplantation in the treatment of patients with acute-on-chronic liver failure. PLoS One. 2013;8(3):e58738. | Wrong study design |

| | |
|---|---|
| 1166 Marchis IF, Radeanu D, Cosgarea M. Tracheal intubation with the rigid tube for laryngoscopy - a new method. Ther Clin Risk Manag. 2019;15:309-313. Published 2019 Feb 25. | Wrong study design |
| 1167 Kitano M, Hoashi T, Kakuta T, et al. Primary Draining Vein Stenting for Obstructive Total Anomalous Pulmonary Venous Connection in Neonates with Right Atrial Isomerism and Functional Single Ventricle Improves Outcome. Pediatr Cardiol. 2018;39(7):1355-1365. | Wrong study design |
| 1168 Kara YA. The Measurement of Serum Tumor Necrosis Factor-alpha Levels in Patients with Lichen Planus. Indian J Dermatol. 2018;63(4):297-300. | Wrong study design |
| 1169 Ali Z, Anjum A, Khurshid L, et al. Evaluation of low-cost custom made VAC therapy compared with conventional wound dressings in the treatment of non-healing lower limb ulcers in lower socio-economic group patients of Kashmir valley. J Orthop Surg Res. 2015;10:183. | Wrong study design |
| 1170 Niu H, Zhang X, Wang B, et al. The clinical utility of image-guided iodine-125 seed in patients with unresectable pancreatic cancer. Tumour Biol. 2016;37(2):2219-2223. | Wrong study design |
| 1171 Ben Mustapha N, Mahmoudi M, Bejaoui M, et al. Mean platelet volume and neutrophil-tolymphocyte ratio as new biomarkers of predicting response to infliximab therapy in Crohn's Disease patients. InJOURNAL OF CROHNS & COLITIS 2015 Feb 1 (Vol. 9, pp. S399-S399). GREAT CLARENDON ST, OXFORD OX2 6DP, ENGLAND: OXFORD UNIV PRESS. | Wrong study design |
| 1172 Huijgen R, Boekholdt SM, Arsenault BJ, et al. Plasma PCSK9 levels and clinical outcomes in the TNT (Treating to New Targets) trial: a nested case-control study. J Am Coll Cardiol. 2012;59(20):1778-1784. | Wrong study design |
| 1173 Cha KY, Lee SH, Chung HM,et al. Quantification of mitochondrial DNA using real-time polymerase chain reaction in patients with premature ovarian failure. Fertil Steril. 2005;84(6):1712-1718. | Wrong study design |
| 1174 Ueshima H, Otake H. Limitations of the Transversus Thoracic Muscle Plane Block. Reg Anesth Pain Med. 2016;41(5):659-660. | Wrong study design |
| 1175 Brown B, Alphs L, Turkoz I, et al. Baseline Demographics and Characteristics From a Paliperidone Palmitate Study in Subjects with Recent-Onset Schizophrenia or Schizophreniform Disorder. Psychopharmacol Bull. 2017;47(3):8-16. | Wrong study design |
| 1176 Teplan V, Králová Lesná I, Piťha J,et al. Asymmetric dimethylarginine and endothelial progenitor cells after renal transplantation: the effect of exercise training. Physiol Res. 2014;63(Suppl 3):S411-7. | Wrong study design |
| 1177 Qiu NC, Liu ME, Wang B, et al. Does the hepatic branch of vagus mediate the secretion of glucagon-like peptide-1 during the Roux-en-Y gastric bypass surgery?. J Gastrointest Surg. 2014;18(11):1957-1964. | Wrong population |
| 1178 Briones TL, Darwish H. Decrease in age-related tau hyperphosphorylation and cognitive improvement following vitamin D supplementation are associated with modulation of brain energy metabolism and redox state. Neuroscience. 2014;262:143-155. | Wrong population |

**Supplementary Table S4.** List of exclusion for the full-text screen for tracked reviews via Google Scholar and Scopus (with reasons).

| | |
|---|---|
| 1 Bolland MJ, Grey A, Reid IR. Differences in overlapping meta-analyses of vitamin D supplements and falls. J Clin Endocrinol Metab. 2014;99(11):4265-4272. | Umbrella review |
| 2 Hafkamp FJ, Tio RA, Otterspoor LC, et al. Optimal effectiveness of heart failure management - an umbrella review of meta-analyses examining the effectiveness of interventions to reduce (re)hospitalizations in heart failure. Heart Fail Rev. 2022;27(5):1683-1748. | Umbrella review |
| 3 Hill A, Garratt A, Levi J, et al. Meta-analysis of Randomized Trials of Ivermectin to Treat SARS-CoV-2 Infection. Open Forum Infect Dis. 2021;8(11):ofab358. | Retracted SR |
| 4 Iwamoto J, Matsumoto H, Takeda T. Efficacy of menatetrenone (vitamin K2) against non-vertebral and hip fractures in patients with neurological diseases: meta-analysis of three randomized, controlled trials [retracted in: Clin Drug Investig. 2018;38(5):479. | Retracted SR |
| 5 Iwamoto J, Takeda T, Matsumoto H. Efficacy of oral bisphosphonates for preventing hip fracture in disabled patients with neurological diseases: a meta-analysis of randomized controlled trials among the Japanese population [retracted in: Curr Med Res Opin. 2017;33(6):1181. | Retracted SR |
| 6 Liang X, Zhou M, Feng JJ, et al. Efficacy of dexmedetomidine on postoperative nausea and vomiting: a meta-analysis of randomized controlled trials. Int J Clin Exp Med. 2015;8(6):8450-8471. | Retracted SR |
| 7 Turner JM, Russo F, Deprest J, et al. Phosphodiesterase-5 inhibitors in pregnancy: Systematic review and meta-analysis of maternal and perinatal safety and clinical outcomes. BJOG. 2022;129(11):1817-1831. | Retracted SR |
| 8 Zhang W, Zhu C, Sun M,et al. Efficacy of bisphosphonates against hip fracture in elderly patients with stroke and Parkinson diseases: meta-analysis of randomized controlled trials [retracted in: J Stroke Cerebrovasc Dis. 2021;30(4):105682. | Retracted SR |
| 9 Gagyor I, Madhok VB, Daly F, et al. WITHDRAWN. Antiviral treatment for Bell's palsy (idiopathic facial paralysis). Cochrane Database Syst Rev. 2015;(5):CD001869. | Retracted SR |
| 10 Bunn F, Trivedi D, Ashraf S. Colloid solutions for fluid resuscitation. Cochrane Database Syst Rev. 2008(1):CD001319. | Old version |
| 11 Bunn F, Trivedi D, Ashraf S. Colloid solutions for fluid resuscitation. Cochrane Database Syst Rev. 2008(1):CD001319. | Old version |
| 12 Bunn F, Trivedi D, Ashraf S. Colloid solutions for fluid resuscitation. Cochrane Database Syst Rev. 2008(1):CD001319. | Old version |
| 13 De-Regil LM, Palacios C, Lombardo LK, et al. Vitamin D supplementation for women during pregnancy. Cochrane Database Syst Rev. 2016(1):CD008873. | Old version |
| 14 Dodd JM, Grivell RM, OBrien CM, et al. Prenatal administration of progestogens for preventing spontaneous preterm birth in women with a multiple pregnancy. Cochrane Database Syst Rev. 2017(10):CD012024. | Old version |
| 15 Franik S, Kremer JA, Nelen WL,et al. Aromatase inhibitors for subfertile women with polycystic ovary syndrome. Cochrane Database Syst Rev. 2014(2):CD010287. | Old version |

| | |
|---|---|
| 16 Haas DM, Hathaway TJ, Ramsey PS. Progestogen for preventing miscarriage in women with recurrent miscarriage of unclear etiology. Cochrane Database Syst Rev. 2018(10):CD003511. | Old version |
| 17 Henry DA, Carless PA, Moxey AJ, et al. Anti-fibrinolytic use for minimising perioperative allogeneic blood transfusion. Cochrane Database Syst Rev. 2007(4):CD001886. | Old version |
| 18 Lensen SF, Manders M, Nastri CO, et al. Endometrial injury for pregnancy following sexual intercourse or intrauterine insemination. Cochrane Database Syst Rev. 2016(6):CD011424. | Old version |
| 19 O'Meara S, Al-Kurdi D, Ologun Y, et al. Antibiotics and antiseptics for venous leg ulcers. Cochrane Database Syst Rev. 2013(12):CD003557. | Old version |
| 20 Popp M, Stegemann M, Metzendorf MI, et al. Ivermectin for preventing and treating COVID-19. Cochrane Database Syst Rev. 2021(7):CD015017. | Old version |
| 21 Zhu J, Chen N, Zhou M, et al. Calcium channel blockers versus other classes of drugs for hypertension. Cochrane Database Syst Rev. 2021(10):CD003654. | Old version |
| 22 Abdullatif J, Certal V, Zaghi S, et al. Maxillary expansion and maxillomandibular expansion for adult OSA: A systematic review and meta-analysis. J Craniomaxillofac Surg. 2016;44(5):574-578. | Not included for SR |
| 23 Abril-Coello R, Correyero-León M, Ceballos-Laita L, Jiménez-Barrio S. Benefits of physical therapy in improving quality of life and pain associated with endometriosis: A systematic review and meta-analysis. Int J Gynaecol Obstet. 2023;162(1):233-243. | Review author excluded this study in SR |
| 24 Álvarez-Pérez Y, Rivero-Santana A, Perestelo-Pérez L, et al. Effectiveness of Mantra-Based Meditation on Mental Health: A Systematic Review and Meta-Analysis. Int J Environ Res Public Health. 2022;19(6):3380. | Included but not synthesized/presented |
| 25 Amraei M, Mohamadpour S, Sayehmiri K,et al. Effects of Vitamin D Deficiency on Incidence Risk of Gestational Diabetes Mellitus: A Systematic Review and Meta-analysis. Front Endocrinol (Lausanne). 2018;9:7. | Not included for SR |
| 26 Antoniou SA, Antoniou GA, Koch OO, et al. Laparoscopic versus open obesity surgery: a meta-analysis of pulmonary complications. Dig Surg. 2015;32(2):98-107. | Not included for SR |
| 27 Arevalo-Rodriguez I, Ciapponi A, Roqué i Figuls M, Muñoz L, Bonfill Cosp X. Posture and fluids for preventing post-dural puncture headache. Cochrane Database Syst Rev. 2016(3):CD009199. | Not included for SR |
| 28 Arulkumaran N, Corredor C, Hamilton MA, et al. Cardiac complications associated with goal-directed therapy in high-risk surgical patients: a meta-analysis. Br J Anaesth. 2014;112(4):648-659. | Not included for SR |
| 29 Ashor AW, Siervo M, Lara J, et al. Antioxidant vitamin supplementation reduces arterial stiffness in adults: a systematic review and meta-analysis of randomized controlled trials. J Nutr. 2014;144(10):1594-1602. | Not included for SR |
| 30 Aune D, Keum N, Giovannucci E, et al. Nut consumption and risk of cardiovascular disease, total cancer, all-cause and cause-specific mortality: a systematic review and dose-response meta-analysis of prospective studies. BMC Med. 2016;14(1):207. | Not included for SR |

| | |
|---|---|
| 31 Bainbridge D, Cheng DC, Martin JE, et al; Evidence-Based Perioperative Clinical Outcomes Research (EPiCOR) Group. NSAID-analgesia, pain control and morbidity in cardiothoracic surgery. Can J Anaesth. 2006;53(1):46-59. | Not included for SR |
| 32 Bakre AT, Chen R, Khutan R, et al. Association between fish consumption and risk of dementia: a new study from China and a systematic literature review and meta-analysis. Public Health Nutr. 2018;21(10):1921-1932. | Not included for SR |
| 33 Beck NN, Johannsen M, Støving RK,et al. Do postoperative psychotherapeutic interventions and support groups influence weight loss following bariatric surgery? A systematic review and meta-analysis of randomized and nonrandomized trials. Obes Surg. 2012;22(11):1790-1797. | Not included for SR |
| 34 Bédia-Tanoh AV, Kassi KF, Touré OA, et al. Meta-Analysis of Data from Four Clinical Trials in the Ivory Coast Assessing the Efficacy of Two Artemisinin-Based Combination Therapies (Artesunate-Amodiaquine and Artemether-Lumefantrine) between 2009 and 2016. Trop Med Infect Dis. 2023;9(1):10. | Not included for SR |
| 35 Bellis JR, Pirmohamed M, Nunn AJ, et al. Dexamethasone and haemorrhage risk in paediatric tonsillectomy: a systematic review and meta-analysis. Br J Anaesth. 2014;113(1):23-42. | Not included for SR |
| 36 Benatar JR, Sidhu K, Stewart RA. Effects of high and low fat dairy food on cardio-metabolic risk factors: a meta-analysis of randomized studies. PLoS One. 2013;8(10):e76480. | Not included for SR |
| 37 Benatar JR, Stewart RAH. Cardiometabolic risk factors in vegans; A meta-analysis of observational studies. PLoS One. 2018;13(12):e0209086. | Not included for SR |
| 38 Bernardes S, Eckert IDC, Burgel CF, et al. Increased energy and/or protein intake improves anthropometry and muscle strength in chronic obstructive pulmonary disease patients: a systematic review with meta-analysis on randomised controlled clinical trials. Br J Nutr. 2022. | Not included for SR |
| 39 Bislev LS, Wamberg L, Rolighed L, et al. Effect of Daily Vitamin D3 Supplementation on Muscle Health: An Individual Participant Meta-analysis. J Clin Endocrinol Metab. 2022;107(5):1317-1327. | Not included for SR |
| 40 Bjelakovic G, Nikolova D, Gluud LL,et al. Antioxidant supplements for prevention of mortality in healthy participants and patients with various diseases. Cochrane Database Syst Rev.2012(3):CD007176. | Duplicate |
| 41 Bloch M H, Panza K E, Landeros-Weisenberger A, et al. Meta-analysis: treatment of attention-deficit/hyperactivity disorder in children with comorbid tic disorders. Journal of the American Academy of Child & Adolescent Psychiatry, 2009, 48(9): 884-893. | Not included for SR |
| 42 Bocskai T, Kovács M, Szakács Z, et al. Is the bispectral index monitoring protective against postoperative cognitive decline? A systematic review with meta-analysis. PLoS One. 2020;15(2):e0229018. | Not included for SR |
| 43 Bonaccio M, Di Castelnuovo A, Costanzo S, et al. Mediterranean diet and mortality in the elderly: a prospective cohort study and a meta-analysis. Br J Nutr. 2018;120(8):841-854. | Not included for SR |
| 44 Bonekamp NE, van Damme I, Geleijnse JM, et al. Effect of dietary patterns on cardiovascular risk factors in people with type 2 diabetes. A systematic review and network meta-analysis. Diabetes Res Clin Pract. 2023;195:110207. | Not included for SR |

| | |
|---|---|
| 45 Bosteels J, Kasius J, Weyers S, et al. Hysteroscopy for treating subfertility associated with suspected major uterine cavity abnormalities. Cochrane Database Syst Rev. 2013(1):CD009461. | Not included for SR |
| 46 Bosteels J, van Wessel S, Weyers S, et al. Hysteroscopy for treating subfertility associated with suspected major uterine cavity abnormalities. Cochrane Database Syst Rev. 2018(12):CD009461. | Not included for SR |
| 47 Braam W, Smits MG, Didden R,et al. Exogenous melatonin for sleep problems in individuals with intellectual disability: a meta-analysis. Dev Med Child Neurol. 2009;51(5):340-349. | Not included for SR |
| 48 Brown J, Farquhar C, Beck J, et al. Clomiphene and anti-oestrogens for ovulation induction in PCOS. Cochrane Database Syst Rev. 2009(4):CD002249. | Review author excluded this study in SR |
| 49 Brown T, Moore TH, Hooper L, et al. Interventions for preventing obesity in children. Cochrane Database Syst Rev. 2019(7):CD001871. | Not included for SR |
| 50 Bui BN, Lensen SF, Gibreel A, et al. Endometrial injury for pregnancy following sexual intercourse or intrauterine insemination. Cochrane Database Syst Rev. 2022(10):CD011424. | Not included for SR |
| 51 Burns FA, Heywood EG, Challand CP,et al. Is there a role for prophylactic mesh in abdominal wall closure after emergency laparotomy? A systematic review and meta-analysis. Hernia. 2020;24(3):441-447. | Not included for SR |
| 52 Cameron ID, Dyer SM, Panagoda CE, et al. Interventions for preventing falls in older people in care facilities and hospitals. Cochrane Database Syst Rev. 2018(9):CD005465. | Not included for SR |
| 53 Cao H, Pan X, Li H, Liu J. Acupuncture for treatment of insomnia: a systematic review of randomized controlled trials. J Altern Complement Med. 2009;15(11):1171-1186. | Review author excluded this study in SR |
| 54 Cappetta K, Beyer C, Johnson JA, et al. Meta-analysis: Risk of dry mouth with second generation antidepressants. Prog Neuropsychopharmacol Biol Psychiatry. 2018;84(Pt A):282-293. | Not included for SR |
| 55 Carter P, Achana F, Troughton J, Gray LJ, Khunti K, Davies MJ. A Mediterranean diet improves HbA1c but not fasting blood glucose compared to alternative dietary strategies: a network meta-analysis. J Hum Nutr Diet. 2014;27(3):280-297. | Not included for SR |
| 56 Catapano F, Chiodini P, De Nicola L, et al. Antiproteinuric response to dual blockade of the renin-angiotensin system in primary glomerulonephritis: meta-analysis and metaregression. Am J Kidney Dis. 2008;52(3):475-485. | Not included for SR |
| 57 Chan KS, Ng STC, Tan CHB, et al. A systematic review and meta-analysis comparing postoperative outcomes of laparoscopic versus open omental patch repair of perforated peptic ulcer. J Trauma Acute Care Surg. 2023;94(1):e1-e13. | Not included for SR |
| 58 Chatzakis C, Tsakmaki E, Psomiadou A, et al. Different pregnancy outcomes according to the polycystic ovary syndrome diagnostic criteria: a systematic review and meta-analysis of 79 studies. Fertil Steril. 2022;117(4):854-881. | Review author excluded this study in SR |

| | |
|---|---|
| 59 Chen B, Guo M, Peker Y, et al. Effect of Continuous Positive Airway Pressure on Lipid Profiles in Obstructive Sleep Apnea: A Meta-Analysis. J Clin Med. 2022;11(3):596. | Not included for SR |
| 60 Chen CC, Siddiqui FJ, Chen TL,et al. Dexamethasone for prevention of postoperative nausea and vomiting in patients undergoing thyroidectomy: meta-analysis of randomized controlled trials. World J Surg. 2012;36(1):61-68. | Not included for SR |
| 61 Chen X, Qin Y, Li S, et al. Efficacy of 5-HT3 receptor antagonists (ondansetron) vs dopamine receptor antagonists (droperidol) for preventing postoperative nausea, vomiting and headache: a meta-analysis. Pteridines, 2019, 30(1): 146-152. | Not included for SR |
| 62 Chen Y, Wang T, Liu X, et al. Low molecular weight heparin and pregnancy outcomes in women with inherited thrombophilia: A systematic review and meta-analysis. J Obstet Gynaecol Res. 2022;48(8):2134-2150. | Not included for SR |
| 63 Cheng HM, Koutsidis G, Lodge JK, et al. Lycopene and tomato and risk of cardiovascular diseases: A systematic review and meta-analysis of epidemiological evidence. Crit Rev Food Sci Nutr. 2019;59(1):141-158. | Not included for SR |
| 64 Cheng J, Zhang W, Zhang XH,et al. ACEI/ARB therapy for IgA nephropathy: a meta analysis of randomised controlled trials. Int J Clin Pract. 2009;63(6):880-888. | Not included for SR |
| 65 Cheng J, Zhang X, Tian J,et al. Combination therapy an ACE inhibitor and an angiotensin receptor blocker for IgA nephropathy: a meta-analysis. Int J Clin Pract. 2012;66(10):917-923. | Not included for SR |
| 66 Cheng SP, Liu TP, Yang PS, et al. Effect of perioperative dexamethasone on subjective voice quality after thyroidectomy: a meta-analysis and systematic review. Langenbecks Arch Surg. 2015;400(8):929-936. | Not included for SR |
| 67 Chiavaroli L, Kendall CWC, Braunstein CR, et al. Effect of pasta in the context of low-glycaemic index dietary patterns on body weight and markers of adiposity: a systematic review and meta-analysis of randomised controlled trials in adults. BMJ Open. 2018;8(3):e019438. | Not included for SR |
| 68 Cirocchi R, Soreide K, Di Saverio S, et al. Meta-analysis of perioperative outcomes of acute laparoscopic versus open repair of perforated gastroduodenal ulcers. J Trauma Acute Care Surg. 2018;85(2):417-425. | Not included for SR |
| 69 Cooper TE, Teng C, Tunnicliffe DJ, et al. Angiotensin-converting enzyme inhibitors and angiotensin receptor blockers for adults with early (stage 1 to 3) non-diabetic chronic kidney disease. Cochrane Database Syst Rev. 2023(7):CD007751. | Review author excluded this study in SR |
| 70 Dai W, Liu H, Zhang T, et al. Dairy product consumption was associated with a lower likelihood of non-alcoholic fatty liver disease: A systematic review and meta-analysis. Front Nutr. 2023;10:1119118. | Not included for SR |
| 71 D'Andrea S, Spaggiari G, Barbonetti A,et al. Endogenous transient doping: physical exercise acutely increases testosterone levels-results from a meta-analysis. J Endocrinol Invest. 2020;43(10):1349-1371. | Not included for SR |
| 72 Davis S, Simpson E, Hamilton J, et al. Denosumab, raloxifene, romosozumab and teriparatide to prevent osteoporotic fragility fractures: a systematic review and economic evaluation. Health Technol Assess. 2020;24(29):1-314. | Review author excluded this study in SR |

| | |
|---|---|
| 73 de Cates AN, Farr MR, Wright N, et al. Fixed-dose combination therapy for the prevention of cardiovascular disease. Cochrane Database Syst Rev. 2014(4):CD009868. | Not included for SR |
| 74 de Oliveira RDJ, de Oliveira RG, de Oliveira LC, et al. Effectiveness of whole-body vibration on bone mineral density in postmenopausal women: a systematic review and meta-analysis of randomized controlled trials. Osteoporos Int. 2023;34(1):29-52. | Not included for SR |
| 75 De Vincentis A, Pedone C, Vespasiani-Gentilucci U, et al. Effect of Sibutramine on Plasma C-Reactive Protein, Leptin and Adipon ectin Concentrations: A Systematic Review and Meta-Analysis of Randomized Contr olled Trials. Curr Pharm Des. 2017;23(6):870-878. | Not included for SR |
| 76 Del Gobbo LC, Falk MC, Feldman R, et al. Effects of tree nuts on blood lipids, apolipoproteins, and blood pressure: systematic review, meta-analysis, and dose-response of 61 controlled intervention trials. Am J Clin Nutr. 2015;102(6):1347-1356. | Not included for SR |
| 77 Desborough M, Sandu R, Brunskill SJ, et al. Fresh frozen plasma for cardiovascular surgery. Cochrane Database Syst Rev. 2015(7):CD007614. | Not included for SR |
| 78 Di Stasi SM, De Carlo F, Pagliarulo V, et al. Hexaminolevulinate hydrochloride in the detection of nonmuscle invasive cancer of the bladder. Ther Adv Urol. 2015;7(6):339-350. | Not included for SR |
| 79 Dong J, Li Z, Luo L,et al. Efficacy of pulmonary rehabilitation in improving the quality of life for patients with chronic obstructive pulmonary disease: Evidence based on nineteen randomized controlled trials. Int J Surg. 2020;73:78-86. | Not included for SR |
| 80 Duffy ME, Hoey L, Hughes CF, et al. Biomarker responses to folic acid intervention in healthy adults: a meta-analysis of randomized controlled trials. Am J Clin Nutr. 2014;99(1):96-106. | Not included for SR |
| 81 Duncan D, Sankar A, Beattie WS, et al. Alpha-2 adrenergic agonists for the prevention of cardiac complications among adults undergoing surgery. Cochrane Database Syst Rev. 2018(3):CD004126. | Review author excluded this study in SR |
| 82 Duncan D, Sankar A, Beattie WS, et al. Alpha-2 adrenergic agonists for the prevention of cardiac complications among adults undergoing surgery. Cochrane Database Syst Rev. 2018(3):CD004126. | Review author excluded this study in SR |
| 83 Duncan S, McAuley DF, Walshe M, et al. Interventions for oropharyngeal dysphagia in acute and critical care: a systematic review and meta-analysis. Intensive Care Med. 2020;46(7):1326-1338. | Not included for SR |
| 84 Elshanbary AA, Zaazouee MS, Darwish YB, et al. Efficacy and Safety of Pectoral Nerve Block (Pecs) Compared With Control, Paravertebral Block, Erector Spinae Plane Block, and Local Anesthesia in Patients Undergoing Breast Cancer Surgeries: A Systematic Review and Meta-analysis. Clin J Pain. 2021;37(12):925-939. | Not included for SR |
| 85 Emadzadeh M, Sahebi R, Khedmatgozar H, et al. A systematic review and meta-analysis of the effect of Vitamin D-fortified food on glycemic indices. Biofactors. 2020;46(4):502-513. | Not included for SR |

| | |
|---|---|
| 86 Esposito K, Maiorino MI, Bellastella G, Chiodini P, Panagiotakos D, Giugliano D. A journey into a Mediterranean diet and type 2 diabetes: a systematic review with meta-analyses. BMJ Open. 2015;5(8):e008222. | Not included for SR |
| 87 Evans JR, Henshaw K. Antioxidant vitamin and mineral supplements for preventing age-related macular degeneration. Cochrane Database Syst Rev. 2008(1):CD000253. | Review author excluded this study in SR |
| 88 Evans JR, Lawrenson JG. Antioxidant vitamin and mineral supplements for preventing age-related macular degeneration. Cochrane Database Syst Rev. 2017(7):CD000253. | Review author excluded this study in SR |
| 89 Fabia MJ, Abdilla N, Oltra R, et al. Antihypertensive activity of angiotensin II AT1 receptor antagonists: a systematic review of studies with 24 h ambulatory blood pressure monitoring. J Hypertens. 2007;25(7):1327-1336. | Review author excluded this study in SR |
| 90 Farquhar C, Marjoribanks J, Basser R, et al. High dose chemotherapy and autologous bone marrow or stem cell transplantation versus conventional chemotherapy for women with metastatic breast cancer. Cochrane Database Syst Rev. 2005(3):CD003142. | Not included for SR |
| 91 Fekete ÁA, Givens DI, Lovegrove JA. Casein-derived lactotripeptides reduce systolic and diastolic blood pressure in a meta-analysis of randomised clinical trials. Nutrients. 2015;7(1):659-681. | Review author excluded this study in SR |
| 92 Feng Q, Fan S, Wu Y, et al. Adherence to the dietary approaches to stop hypertension diet and risk of stroke: A meta-analysis of prospective studies. Medicine (Baltimore). 2018;97(38):e12450. | Not included for SR |
| 93 Fogacci F, Cicero AFG, Derosa G, et al. Effect of pistachio on brachial artery diameter and flow-mediated dilatation: A systematic review and meta-analysis of randomized, controlled-feeding clinical studies. Crit Rev Food Sci Nutr. 2019;59(2):328-335. | Not included for SR |
| 94 Ford AH, Almeida OP. Effect of homocysteine lowering treatment on cognitive function: a systematic review and meta-analysis of randomized controlled trials. J Alzheimers Dis. 2012;29(1):133-149. | Review author excluded this study in SR |
| 95 Fossati R, Confalonieri C, Torri V, et al. Cytotoxic and hormonal treatment for metastatic breast cancer: a systematic review of published randomized trials involving 31,510 women. J Clin Oncol. 1998;16(10):3439-3460. | Not included for SR |
| 96 Freemantle N, Xu Y, Wilson FR, et al. Network meta-analysis of immune-oncology monotherapy as first-line treatment for advanced non-small-cell lung cancer in patients with PD-L1 expression ⩾50. Ther Adv Med Oncol. 2022;14:17588359221105024. | Not included for SR |
| 97 Fremes SE, Wong BI, Lee E, et al. Metaanalysis of prophylactic drug treatment in the prevention of postoperative bleeding. Ann Thorac Surg. 1994;58(6):1580-1588. | Not included for SR |

| | |
|---|---|
| 98 Fretheim A, Odgaard-Jensen J, Brørs O, et al. Comparative effectiveness of antihypertensive medication for primary prevention of cardiovascular disease: systematic review and multiple treatments meta-analysis. BMC Med. 2012;10:33. | Review author excluded this study in SR |
| 99 Friedman DJ, Black-Maier EW, Barnett AS, et al. Left Atrial Appendage Electrical Isolation for Treatment of Recurrent Atrial Fibrillation: A Meta-Analysis. JACC Clin Electrophysiol. 2018;4(1):112-120. | Not report study-level data |
| 100 Fujii T, Le Du F, Xiao L, et al. Effectiveness of an Adjuvant Chemotherapy Regimen for Early-Stage Breast Cancer: A Systematic Review and Network Meta-analysis. JAMA Oncol. 2015;1(9):1311-1318. | Not included for SR |
| 101 Gagnaire J, Verhoeven PO, Grattard F, et al. Epidemiology and clinical relevance of Staphylococcus aureus intestinal carriage: a systematic review and meta-analysis. Expert Rev Anti Infect Ther. 2017;15(8):767-785. | Not included for SR |
| 102 Garcia Lopez M, Baron JA, Omsland TK, et al. Homocysteine-Lowering Treatment and the Risk of Fracture: Secondary Analysis of a Randomized Controlled Trial and an Updated Meta-Analysis. JBMR Plus. 2018;2(5):295-303. | Not included for SR |
| 103 Gay HC, Rao SG, Vaccarino V, et al. Effects of Different Dietary Interventions on Blood Pressure: Systematic Review and Meta-Analysis of Randomized Controlled Trials. Hypertension. 2016;67(4):733-739. | Not included for SR |
| 104 Gethin G, Cowman S, Kolbach DN. Debridement for venous leg ulcers. Cochrane Database Syst Rev. 2015(9):CD008599. | Not included for SR |
| 105 Gillespie LD, Robertson MC, Gillespie WJ, et al. Interventions for preventing falls in older people living in the community. Cochrane Database Syst Rev.2012(9):CD007146. | Not included for SR |
| 106 Giraudier M, Ventura-Bort C, Burger AM, et al. Evidence for a modulating effect of transcutaneous auricular vagus nerve stimulation (taVNS) on salivary alpha-amylase as indirect noradrenergic marker: A pooled mega-analysis. Brain Stimul. 2022;15(6):1378-1388. | Not included for SR |
| 107 Gok Metin Z, Helvaci A, Gulbahar Eren M. Effects of Aloe vera in adults with mucocutaneous problems: A systematic review and meta-analysis. J Adv Nurs. 2021;77(3):1105-1126. | Not included for SR |
| 108 Gómez-Sánchez E, Hernández-Gómez A, Guzmán-Flores JM, et al. Celecoxib Decreases the Need for Rescue Analgesics after Total Knee Arthroplasty: A Meta-Analysis. Clin Pract. 2024;14(2):461-472. | Not included for SR |
| 109 Guasch-Ferré M, Li J, Hu FB,et al. Effects of walnut consumption on blood lipids and other cardiovascular risk factors: an updated meta-analysis and systematic review of controlled trials. Am J Clin Nutr. 2018 ;108(1):174-187. | Not included for SR |
| 110 Guedes HG, Moura DTH, Duarte RB, et al. A comparison of the efficiency of 22G versus 25G needles in EUS-FNA for solid pancreatic mass assessment: A systematic review and meta-analysis. Clinics (Sao Paulo). 2018;73:e261. | Review author excluded this study in SR |
| 111 Guraya SS, Alhussaini KA, Shaqrun FM,et al. Correlation of clinical, radiological and serum analysis of hypovitaminosis D with polycystic ovary syndrome: A systematic review and meta-analysis. J Taibah Univ Med Sci. 2017;12(4):277-283. | Not included for SR |

| | |
|---|---|
| 112 Haase N, Perner A, Hennings LI, et al. Hydroxyethyl starch 130/0.38-0.45 versus crystalloid or albumin in patients with sepsis: systematic review with meta-analysis and trial sequential analysis. BMJ. 2013;346:f839. | Not included for SR |
| 113 Hamel JF, Sabbagh C, Alves A, et al. Comparison of treatment to improve gastrointestinal functions after colorectal surgery within enhanced recovery programmes: a systematic review and meta-analysis. Sci Rep. 2021;11(1):7423. | Review author excluded this study in SR |
| 114 Hamulyák EN, Scheres LJ, Marijnen MC,et al. Aspirin or heparin or both for improving pregnancy outcomes in women with persistent antiphospholipid antibodies and recurrent pregnancy loss. Cochrane Database Syst Rev. 2020(5):CD012852. | Review author excluded this study in SR |
| 115 Hamulyák EN, Scheres LJJ, Goddijn M,et al. Antithrombotic therapy to prevent recurrent pregnancy loss in antiphospholipid syndrome-What is the evidence?. J Thromb Haemost. 2021;19(5):1174-1185. | Not included for SR |
| 116 Harcombe Z, Baker JS, DiNicolantonio JJ, et al. Evidence from randomised controlled trials does not support current dietary fat guidelines: a systematic review and meta-analysis. Open Heart. 2016;3(2):e000409. | Not included for SR |
| 117 Haslbeck FB, Mueller K, Karen T, et al. Musical and vocal interventions to improve neurodevelopmental outcomes for preterm infants. Cochrane Database Syst Rev. 2023(9):CD013472. | Not included for SR |
| 118 Hauger H, Laursen RP, Ritz C, et al. Effects of vitamin D supplementation on cardiometabolic outcomes in children and adolescents: a systematic review and meta-analysis of randomized controlled trials. Eur J Nutr. 2020;59(3):873-884. | Not included for SR |
| 119 He K, Li Y, Guo X, et al. Food groups and the likelihood of non-alcoholic fatty liver disease: a systematic review and meta-analysis. Br J Nutr. 2020;124(1):1-13. | Not included for SR |
| 120 Heshmati J, Sepidarkish M, Shidfar F, et al. Effect of Breastfeeding in Early Life on Cardiorespiratory and Physical Fitness: A Systematic Review and Meta-Analysis. Breastfeed Med. 2018;13(4):248-258. | Not included for SR |
| 121 Hopefl R, Ben-Eltriki M, Deb S. Association Between Vitamin D Levels and Inflammatory Markers in COVID-19 Patients: A Meta-Analysis of Observational Studies. J Pharm Pharm Sci. 2022;25:124-136. | Not included for SR |
| 122 Hsueh YW, Yeh TL, Lin CY, et al. Association of metabolically healthy obesity and elevated risk of coronary artery calcification: a systematic review and meta-analysis. PeerJ. 2020;8:e8815. | Not included for SR |
| 123 Huang J, Lin J, Lu X, et al. Delayed versus immediate frozen embryo transfer after oocyte retrieval: a systematic review and meta-analysis. J Assist Reprod Genet. 2020;37(8):1949-1957. | Not included for SR |
| 124 Huët C, Salmi LR, Fergusson D, et al. A meta-analysis of the effectiveness of cell salvage to minimize perioperative allogeneic blood transfusion in cardiac and orthopedic surgery. International Study of Perioperative Transfusion (ISPOT) Investigators. Anesth Analg. 1999;89(4):861-869. | Review author excluded this study in SR |
| 125 Iftikhar IH, Blankfield RP. Effect of continuous positive airway pressure on hemoglobin A(1c) in patients with obstructive sleep apnea: a systematic review and meta-analysis. Lung. 2012;190(6):605-611. | Not included for SR |

| | |
|---|---|
| 126 Imberger G, Orr A, Thorlund K, Wetterslev J, Myles P, Møller AM. Does anaesthesia with nitrous oxide affect mortality or cardiovascular morbidity? A systematic review with meta-analysis and trial sequential analysis. Br J Anaesth. 2014;112(3):410-426. | Not included for SR |
| 127 Isiordia-Espinoza MA, de Jesús Pozos-Guillén A, Aragon-Martinez OH. Analgesic efficacy and safety of single-dose tramadol and non-steroidal anti-inflammatory drugs in operations on the third molars: a systematic review and meta-analysis. Br J Oral Maxillofac Surg. 2014;52(9):775-783. | Review author excluded this study in SR |
| 128 Jabri A, Kumar A, Verghese E, et al. Meta-analysis of effect of vegetarian diet on ischemic heart disease and all-cause mortality. Am J Prev Cardiol. 2021;7:100182. | Not included for SR |
| 129 Jamshidi S, Moradi Y, Nameni G, Mohsenpour MA, Vafa M. Effects of cashew nut consumption on body composition and glycemic indices: A meta-analysis and systematic review of randomized controlled trials. Diabetes Metab Syndr. 2021;15(2):605-613. | Not included for SR |
| 130 Jankovic N, Geelen A, Streppel MT, et al. Adherence to a healthy diet according to the World Health Organization guidelines and all-cause mortality in elderly adults from Europe and the United States. Am J Epidemiol. 2014;180(10):978-988. | Not included for SR |
| 131 Jeevanantham V, Butler M, Saad A, et al. Adult bone marrow cell therapy improves survival and induces long-term improvement in cardiac parameters: a systematic review and meta-analysis. Circulation. 2012;126(5):551-568. | Not included for SR |
| 132 Jespersen KV, Pando-Naude V, Koenig J, et al. Listening to music for insomnia in adults. Cochrane Database Syst Rev. 2022(8):CD010459. | Not included for SR |
| 133 Jiménez-García AM, Bonnel G, Álvarez-Mota A, et al. Current perspectives on neuromodulation in ALS patients: A systematic review and meta-analysis. PLoS One. 2024;19(3):e0300671. | Not included for SR |
| 134 Kang K, Shu XL, Zhong JX, et al. Effect of L-arginine on immune function: a meta-analysis. Asia Pac J Clin Nutr. 2014;23(3):351-359. | Not included for SR |
| 135 Kang SY, Kim HB, Sunwoo S. Association between anemia and maternal depression: A systematic review and meta-analysis. J Psychiatr Res. 2020;122:88-96. | Not included for SR |
| 136 Keats EC, Oh C, Chau T,et al. Effects of vitamin and mineral supplementation during pregnancy on maternal, birth, child health and development outcomes in low- and middle-income countries: A systematic review. Campbell Syst Rev. 2021;17(2):e1127. | Not included for SR |
| 137 Kelly JT, Palmer SC, Wai SN, et al. Healthy Dietary Patterns and Risk of Mortality and ESRD in CKD: A Meta-Analysis of Cohort Studies. Clin J Am Soc Nephrol. 2017;12(2):272-279. | Not included for SR |
| 138 Khasteganan N, Lycett D, Furze G,et al. Health, not weight loss, focused programmes versus conventional weight loss programmes for cardiovascular risk factors: a systematic review and meta-analysis. Syst Rev. 2019;8(1):200. | Not included for SR |
| 139 Kim E, Je Y. Fish consumption and the risk of dementia: Systematic review and meta-analysis of prospective studies. Psychiatry Res. 2022;317:114889. | Not included for SR |

| | |
|---|---|
| 140 Kong DL, Qin Z, Wang W, et al. Association between obstructive sleep apnea and metabolic syndrome: a meta-analysis. Clin Invest Med. 2016;39(5):E161-E172. | Not included for SR |
| 141 Kontogianni MD, Panagiotakos DB. Dietary patterns and stroke: a systematic review and re-meta-analysis. Maturitas. 2014;79(1):41-47. | Not included for SR |
| 142 Kotani Y, Kataoka Y, Izawa J, et al. High versus low blood pressure targets for cardiac surgery while on cardiopulmonary bypass. Cochrane Database Syst Rev. 2022;(11):CD013494. | Not included for SR |
| 143 Lam FC, Khan TM, Faidah H, et al. Effectiveness of whey protein supplements on the serum levels of amino acid, creatinine kinase and myoglobin of athletes: a systematic review and meta-analysis. Syst Rev. 2019;8(1):130. | Not included for SR |
| 144 Lambert P, Cyna AM, Knight N, Middleton P. Clonidine premedication for postoperative analgesia in children. Cochrane Database Syst Rev.2014(1):CD009633. | Review author excluded this study in SR |
| 145 Landoni G, Biondi-Zoccai GG, Zangrillo A, et al. Desflurane and sevoflurane in cardiac surgery: a meta-analysis of randomized clinical trials. J Cardiothorac Vasc Anesth. 2007;21(4):502-511. | Not included for SR |
| 146 Lara J, Hobbs N, Moynihan PJ, et al. Effectiveness of dietary interventions among adults of retirement age: a systematic review and meta-analysis of randomized controlled trials. BMC Med. 2014;12:60. | Review author excluded this study in SR |
| 147 Latham NK, Anderson CS, Reid IR. Effects of vitamin D supplementation on strength, physical performance, and falls in older persons: a systematic review. J Am Geriatr Soc. 2003;51(9):1219-26. | Not included for SR |
| 148 Lauche R, Langhorst J, Dobos G, et al. A systematic review and meta-analysis of Tai Chi for osteoarthritis of the knee. Complement Ther Med. 2013;21(4):396-406. | Not included for SR |
| 149 Li B, Wang H. Dexamethasone reduces nausea and vomiting but not pain after thyroid surgery: a meta-analysis of randomized controlled trials. Med Sci Monit. 2014;20:2837-2845. | Not included for SR |
| 150 Li D, Li X, Cui W, et al. Liberal versus conservative fluid therapy in adults and children with sepsis or septic shock. Cochrane Database Syst Rev. 2018(12):CD010593. | Not included for SR |
| 151 Li EC, Heran BS, Wright JM. Angiotensin converting enzyme (ACE) inhibitors versus angiotensin receptor blockers for primary hypertension. Cochrane Database Syst Rev. 2014(8):CD009096. | Review author excluded this study in SR |
| 152 Li J, Gao YH, Xu L, et al. Meta-analysis of heparin combined with aspirin versus aspirin alone for unexplained recurrent spontaneous abortion. Int J Gynaecol Obstet. 2020;151(1):23-32. | Not included for SR |
| 153 Li M, Hu X, Tan Y, et al. Meta-analysis of randomized controlled trials on the efficacy and safety of ondansetron in preventing postanesthesia shivering. Int J Surg. 2016;35:34-43. | Not included for SR |

| | |
|---|---|
| 154 Li M, Kang Y, Wang Q,et al. Efficacy of Autologous Intrauterine Infusion of Platelet-Rich Plasma in Patients with Unexplained Repeated Implantation Failures in Embryo Transfer: A Systematic Review and Meta-Analysis. J Clin Med. 2022;11(22):6753. | Not included for SR |
| 155 Li SS, Blanco Mejia S, Lytvyn L, et al. Effect of Plant Protein on Blood Lipids: A Systematic Review and Meta-Analysis of Randomized Controlled Trials. J Am Heart Assoc. 2017;6(12):e006659. | Not included for SR |
| 156 Li X, Sun Z, Han C, et al. A systematic review and meta-analysis of intravenous glucocorticoids for acute pain following total hip arthroplasty. Medicine (Baltimore). 2017;96(19):e6872. | Not included for SR |
| 157 Li Z, Liu Y, Wang J, Liu J, Zhang C, Liu Y. Effectiveness of cognitive behavioural therapy for perinatal depression: A systematic review and meta-analysis. J Clin Nurs. 2020;29(17-18):3170-3182. | Not included for SR |
| 158 Li Z, Liu Y, Wang J, Zhang C, Liu Y. Effectiveness of cognitive behavioral therapy on mood symptoms in patients with implantable cardioverter defibrillator: A systematic review and meta-analysis. Complement Ther Clin Pract. 2022;47:101570. | Not included for SR |
| 159 Liang F, Zhou Y, Zhang Z, et al. Association of vitamin D in individuals with periodontitis: an updated systematic review and meta-analysis. BMC Oral Health. 2023;23(1):387. | Not included for SR |
| 160 Liang S, Xing M, Jiang S, Zou W. Effect of Intravenous Dexamethasone on Postoperative Pain in Patients Undergoing Total Knee Arthroplasty: A Systematic Review and Meta-Analysis. Pain Physician. 2022;25(2):E169-E183. | Not included for SR |
| 161 Lin J, Mo X, Yang Y,et al. Association between vitamin D deficiency and diabetic foot ulcer wound in diabetic subjects: A meta-analysis. Int Wound J. 2023;20(1):55-62. | Not included for SR |
| 162 Lin T, Chen Y, Cheng X,et al. Enoxaparin (or plus aspirin) for the prevention of recurrent miscarriage: A meta-analysis of randomized controlled studies. Eur J Obstet Gynecol Reprod Biol. 2019;234:53-57. | Not included for SR |
| 163 Lin TJ, Huang YL, Kang YN, et al. Effectiveness of Topical Conditioned Medium of Stem Cells in Facial Skin Nonsurgical Resurfacing Modalities for Antiaging: Systematic Review and Meta-Analysis of Randomized Controlled Trials. Aesthetic Plast Surg. 2023;47(2):799-807. | Not included for SR |
| 164 Lindberg T, Andersson O, Palm M,et al. A systematic review and meta-analysis of dressings used for wound healing: the efficiency of honey compared to silver on burns. Contemp Nurse. 2015;51(2-3):121-134. | Not included for SR |
| 165 Ling HQ, Chen ZH, He L, et al. Comparative Efficacy and Safety of 11 Drugs as Therapies for Adults With Neuropathic Pain After Spinal Cord Injury: A Bayesian Network Analysis Based on 20 Randomized Controlled Trials. Front Neurol. 2022;13:818522. | Not included for SR |
| 166 Liu B, Chen G, Yu Z, et al. Bone Mineral Density and Related Scores in Parkinson's Disease: A Systematic Review and Meta-Analysis. World Neurosurg. 2021;146:e1202-e1218. | Not included for SR |
| 167 Liu X, Li Y, Tobias DK,et al. Changes in Types of Dietary Fats Influence Long-term Weight Change in US Women and Men. J Nutr. 2018;148(11):1821-1829. | Not included for SR |

| | |
|---|---|
| 168 Liu X, Wang D, Zheng L,et al. Is early oral feeding after gastric cancer surgery feasible? A systematic review and meta-analysis of randomized controlled trials. PLoS One. 2014;9(11):e112062. | Not included for SR |
| 169 Ma J, Wang XY, Hu ZD, et al. Meta-analysis of the efficacy and safety of adding an angiotensin receptor blocker (ARB) to a calcium channel blocker (CCB) following ineffective CCB monotherapy. J Thorac Dis. 2015;7(12):2243-2252. | Not included for SR |
| 170 Makani H, Bangalore S, Desouza KA,et al. Efficacy and safety of dual blockade of the renin-angiotensin system: meta-analysis of randomised trials. BMJ. 2013;346:f360. | Not included for SR |
| 171 Maneeton N, Maneeton B, Eurviriyanukul K, et al. Efficacy, tolerability, and acceptability of bupropion for major depressive disorder: a meta-analysis of randomized-controlled trials comparison with venlafaxine. Drug Des Devel Ther. 2013;7:1053-1062. | Not included for SR |
| 172 Mao S, Zhang A, Huang S. Meta-analysis of Zn, Cu and Fe in the hair of Chinese children with recurrent respiratory tract infection. Scand J Clin Lab Invest. 2014;74(7):561-567. | Not included for SR |
| 173 Martos-Cobo E, Mayoral-Sanz P, Expósito-Delgado AJ, et al. Effect of rapid maxillary expansion on the apnoea-hypopnoea index during sleep in children. Systematic review. J Clin Exp Dent. 2022;14(9):e769-e775. | Not included for SR |
| 174 Marventano S, Izquierdo Pulido M, Sánchez-González C, et al. Legume consumption and CVD risk: a systematic review and meta-analysis. Public Health Nutr. 2017;20(2):245-254. | Not included for SR |
| 175 Mason BJ, Lehert P. Acamprosate for alcohol dependence: a sex-specific meta-analysis based on individual patient data. Alcohol Clin Exp Res. 2012;36(3):497-508. | Not included for SR |
| 176 Mayhew AJ, de Souza RJ, Meyre D, Anand SS, Mente A. A systematic review and meta-analysis of nut consumption and incident risk of CVD and all-cause mortality. Br J Nutr. 2016;115(2):212-225. | Not included for SR |
| 177 Mazidi M, Katsiki N, Banach M. A Greater Flavonoid Intake Is Associated with Lower Total and Cause-Specific Mortality: A Meta-Analysis of Cohort Studies. Nutrients. 2020;12(8):2350. | Not included for SR |
| 178 Mazidi M, Rezaie P, Ferns GA,et al. Impact of different types of tree nut, peanut, and soy nut consumption on serum C-reactive protein (CRP): A systematic review and meta-analysis of randomized controlled clinical trials. Medicine (Baltimore). 2016;95(44):e5165. | Not included for SR |
| 179 McAlister FA; Renin Angiotension System Modulator Meta-Analysis Investigators. Angiotensin-converting enzyme inhibitors or angiotensin receptor blockers are beneficial in normotensive atherosclerotic patients: a collaborative meta-analysis of randomized trials. Eur Heart J. 2012;33(4):505-514. | Not included for SR |
| 180 McEvoy CT, Leng Y, Peeters GM, et al. Interventions involving a major dietary component improve cognitive function in cognitively healthy adults: a systematic review and meta-analysis. Nutr Res. 2019;66:1-12. | Not included for SR |
| 181 McQueen, J., McFeely, G. Case management for return to work for individuals living with cancer: a systematic review. International Journal of Therapy And Rehabilitation, 24(5), 203-210. | Not included for SR |

| | |
|---|---|
| 182 Mei H, Pu J, Yang C, et al. Laparoscopic versus open pyeloplasty for ureteropelvic junction obstruction in children: a systematic review and meta-analysis. J Endourol. 2011;25(5):727-736. | Not included for SR |
| 183 Melton H, Meader N, Dale H, et al. Interventions for adults with a history of complex traumatic events: the INCiTE mixed-methods systematic review. Health Technol Assess. 2020;24(43):1-312. | Not included for SR |
| 184 Messina AG, Wang M, Ward MJ, et al. Anaesthetic interventions for prevention of awareness during surgery. Cochrane Database Syst Rev. 2016(10):CD007272. | Review author excluded this study in SR |
| 185 Miralpeix E, González-Comadran M, Solà I, Manau D, et al. Efficacy of luteal phase support with vaginal progesterone in intrauterine insemination: a systematic review and meta-analysis. J Assist Reprod Genet. 2014;31(1):89-100. | Not included for SR |
| 186 Mohanty S, Mohanty P, Trivedi C, et al. Long-Term Outcome of Pulmonary Vein Isolation With and Without Focal Impulse and Rotor Modulation Mapping: Insights From a Meta-Analysis. Circ Arrhythm Electrophysiol. 2018;11(3):e005789. | Not included for SR |
| 187 Monlezun DJ, Carr C, Niu T, et al. Meta-analysis and machine learning-augmented mixed effects cohort analysis of improved diets among 5847 medical trainees, providers and patients. Public Health Nutr. 2022;25(2):281-289. | Not included for SR |
| 188 Moosavian SP, Maharat M, Chambari M, et al. Effects of tart cherry juice consumption on cardio-metabolic risk factors: A systematic review and meta-analysis of randomized-controlled trials. Complement Ther Med. 2022;71:102883. | Not included for SR |
| 189 Morvaridzadeh M, Agah S, Alibakhshi P, et al. Effects of Calcium and Vitamin D Co-supplementation on the Lipid Profile: A Systematic Review and Meta-analysis. Clin Ther. 2021;43(9):274-296. | Not included for SR |
| 190 Motallaei M, Ramezani-Jolfaie N, Mohammadi M, et al. Effects of orange juice intake on cardiovascular risk factors: A systematic review and meta-analysis of randomized controlled clinical trials. Phytother Res. 2021;35(10):5427-5439. | Not included for SR |
| 191 Muir SW, Montero-Odasso M. Effect of vitamin D supplementation on muscle strength, gait and balance in older adults: a systematic review and meta-analysis. J Am Geriatr Soc. 2011;59(12):2291-2300. | Not included for SR |
| 192 Mukete BN, Cassidy M, Ferdinand KC, et al. Long-Term Anti-Hypertensive Therapy and Stroke Prevention: A Meta-Analysis. Am J Cardiovasc Drugs. 2015;15(4):243-257. | Not included for SR |
| 193 Muley A, Fernandez R, Green H, et al. Effect of thiamine supplementation on glycaemic outcomes in adults with type 2 diabetes: a systematic review and meta-analysis. BMJ Open. 2022;12(8):e059834. | Not included for SR |
| 194 Musso G, Cassader M, Olivetti C, et al. Association of obstructive sleep apnoea with the presence and severity of non-alcoholic fatty liver disease. A systematic review and meta-analysis. Obes Rev. 2013;14(5):417-431. | Not included for SR |
| 195 Nishimori M, Low JH, Zheng H, et al. Epidural pain relief versus systemic opioid-based pain relief for abdominal aortic surgery. Cochrane Database Syst Rev. 2012(7):CD005059. | Not included for SR |

| | |
|---|---|
| 196 O'Hare PE, Wilson BJ, Loga MG, et al. Effect of submucosal dexamethasone injections in the prevention of postoperative pain, trismus, and oedema associated with mandibular third molar surgery: a systematic review and meta-analysis. Int J Oral Maxillofac Surg. 2019;48(11):1456-1469. | Not included for SR |
| 197 O'Meara S, Cullum N, Nelson EA, et al. Compression for venous leg ulcers. Cochrane Database Syst Rev. 2012(11):CD000265. | Included but not synthesized/presented |
| 198 Ong KT, Delerme S, Pannier B, et al. Aortic stiffness is reduced beyond blood pressure lowering by short-term and long-term antihypertensive treatment: a meta-analysis of individual data in 294 patients. J Hypertens. 2011;29(6):1034-1042. | Not included for SR |
| 199 Palmer SC, Maggo JK, Campbell KL, et al. Dietary interventions for adults with chronic kidney disease. Cochrane Database Syst Rev. 2017(4):CD011998. | Not included for SR |
| 200 Palomba S, Falbo A, Orio F Jr,et al. Effect of preconceptional metformin on abortion risk in polycystic ovary syndrome: a systematic review and meta-analysis of randomized controlled trials. Fertil Steril. 2009;92(5):1646-1658. | Review author excluded this study in SR |
| 201 Pan X, Huang S, Gan P, et al. Endoscopic ultrasound-guided tissue acquisition for splenic lesions: A systematic review and meta-analysis of diagnostic test accuracy. PLoS One. 2022;17(10):e0276529. | Not included for SR |
| 202 Paranjothy S, Griffiths JD, Broughton HK, et al. Interventions at caesarean section for reducing the risk of aspiration pneumonitis. Cochrane Database Syst Rev. 2014(2):CD004943. | Not included for SR |
| 203 Park SW, Lee H, Ahn H. Bispectral Index Versus Standard Monitoring in Sedation for Endoscopic Procedures: A Systematic Review and Meta-Analysis. Dig Dis Sci. 2016;61(3):814-824. | Not included for SR |
| 204 Parks NE, Jackson-Tarlton CS, Vacchi L, et al. Dietary interventions for multiple sclerosis-related outcomes. Cochrane Database Syst Rev. 2020(5):CD004192. | Review author excluded this study in SR |
| 205 Peleg Hasson S, Brezis MR, Shachar E, et al. Adjuvant endocrine therapy in HER2-positive breast cancer patients: systematic review and meta-analysis. ESMO Open. 2021(3):100158. | Not included for SR |
| 206 Peou S, Milliard-Hasting B, Shah SA. Impact of avocado-enriched diets on plasma lipoproteins: A meta-analysis. J Clin Lipidol. 2016;10(1):161-171. | Not included for SR |
| 207 Perel P, Roberts I, Ker K. Colloids versus crystalloids for fluid resuscitation in critically ill patients. Cochrane Database Syst Rev. 2013;(2):CD000567. | Not included for SR |
| 208 Perel P, Roberts I. Colloids versus crystalloids for fluid resuscitation in critically ill patients. Cochrane Database Syst Rev. 2007(4):CD000567. | Review author excluded this study in SR |

| | |
|---|---|
| 209 Perritt E, Wallace H, Singh S, et al. Effect of intra-articular alpha-agonists on post-operative outcomes following arthroscopic knee surgery: A systematic review and meta-analysis[J]. Egyptian Journal of Anaesthesia, 2017, 33(2): 195-201. | Not included for SR |
| 210 Plante J, Turgeon AF, Zarychanski R, et al. Effect of systemic steroids on post-tonsillectomy bleeding and reinterventions: systematic review and meta-analysis of randomised controlled trials. BMJ. 2012;345:e5389. | Review author excluded this study in SR |
| 211 Psaltopoulou T, Sergentanis TN, Panagiotakos DB, et al. Mediterranean diet, stroke, cognitive impairment, and depression: A meta-analysis. Ann Neurol. 2013;74(4):580-591. | Not included for SR |
| 212 Qian F, Korat AA, Malik V, et al. Metabolic Effects of Monounsaturated Fatty Acid-Enriched Diets Compared With Carbohydrate or Polyunsaturated Fatty Acid-Enriched Diets in Patients With Type 2 Diabetes: A Systematic Review and Meta-analysis of Randomized Controlled Trials. Diabetes Care. 2016;39(8):1448-1457. | Not included for SR |
| 213 Qin LQ, Xu JY, Dong JY, et al. Lactotripeptides intake and blood pressure management: a meta-analysis of randomised controlled clinical trials. Nutr Metab Cardiovasc Dis. 2013;23(5):395-402. | Not included for SR |
| 214 Qin YY, Li H, Guo XJ, et al. Adjuvant chemotherapy, with or without taxanes, in early or operable breast cancer: a meta-analysis of 19 randomized trials with 30698 patients. PLoS One. 2011;6(11):e26946. | Review author excluded this study in SR |
| 215 Quotah OF, Andreeva D, Nowak KG, et al. Interventions in preconception and pregnant women at risk of gestational diabetes; a systematic review and meta-analysis of randomised controlled trials. Diabetol Metab Syndr. 2024;16(1):8. | Not included for SR |
| 216 Radd-Vagenas S, Duffy SL, Naismith SL, et al. Effect of the Mediterranean diet on cognition and brain morphology and function: a systematic review of randomized controlled trials. Am J Clin Nutr. 2018;107(3):389-404. | Not included for SR |
| 217 Rahbari NN, Zimmermann JB, Schmidt T, et al. Meta-analysis of standard, restrictive and supplemental fluid administration in colorectal surgery. Br J Surg. 2009;96(4):331-341. | Not included for SR |
| 218 Ramaswamy VV, Abiramalatha T, Bandyopadhyay T,et al. Digital tracheal intubation and finger palpation to confirm endotracheal tube tip position in neonates: A systematic review and meta-analysis. Pediatr Pulmonol. 2021;56(9):2893-2902. | Not included for SR |
| 219 Rao M, Afshin A, Singh G,et al. Do healthier foods and diet patterns cost more than less healthy options? A systematic review and meta-analysis. BMJ Open. 2013;3(12):e004277. | Not included for SR |
| 220 Rees K, Hartley L, Flowers N, et al. 'Mediterranean' dietary pattern for the primary prevention of cardiovascular disease. Cochrane Database Syst Rev. 2013(8):CD009825. | Not included for SR |
| 221 Rees K, Takeda A, Martin N, et al. Mediterranean-style diet for the primary and secondary prevention of cardiovascular disease. Cochrane Database Syst Rev. 2019(3):CD009825. | Not included for SR |
| 222 Ribeiro CT, Dias FA, Fregonezi GA. Hydrogel dressings for venous leg ulcers. Cochrane Database Syst Rev. 2022(8):CD010738. | Not included for SR |

| | |
|---|---|
| 223 Rodger MA, Carrier M, Le Gal G, et al. Meta-analysis of low-molecular-weight heparin to prevent recurrent placenta-mediated pregnancy complications. Blood. 2014;123(6):822-828. | Not included for SR |
| 224 Rodríguez-Monforte M, Flores-Mateo G, Sánchez E. Dietary patterns and CVD: a systematic review and meta-analysis of observational studies. Br J Nutr. 2015;114(9):1341-1359. | Not included for SR |
| 225 Rollins KE, Javanmard-Emamghissi H, Lobo DN. Impact of mechanical bowel preparation in elective colorectal surgery: A meta-analysis. World J Gastroenterol. 2018;24(4):519-536. | Not included for SR |
| 226 Roque M, Haahr T, Geber S,et al. Fresh versus elective frozen embryo transfer in IVF/ICSI cycles: a systematic review and meta-analysis of reproductive outcomes. Hum Reprod Update. 2019;25(1):2-14. | Not included for SR |
| 227 Rosato V, Temple NJ, La Vecchia C, et al. Mediterranean diet and cardiovascular disease: a systematic review and meta-analysis of observational studies. Eur J Nutr. 2019;58(1):173-191. | Not included for SR |
| 228 Saha S, Saha S. A comparison of the risk of cesarean section in gestational diabetes mellitus patients supplemented antenatally with vitamin D containing supplements versus placebo: A systematic review and meta-analysis of double-blinded randomized controlled trials. J Turk Ger Gynecol Assoc. 2020;21(3):201-212. | Not included for SR |
| 229 Saha S, Saha S. Changes in anthropometric and blood 25-hydroxyvitamin D measurements in antenatal vitamin supplemented gestational diabetes mellitus patients: a systematic review and meta-analysis of randomized controlled trials. J Turk Ger Gynecol Assoc. 2021;22(3):217-234. | Not included for SR |
| 230 Sajid MS, Parampalli U, Whitehouse P,et al. A systematic review comparing transanal haemorrhoidal de-arterialisation to stapled haemorrhoidopexy in the management of haemorrhoidal disease. Tech Coloproctol. 2012;16(1):1-8. | Not included for SR |
| 231 Saporito A, Ceppi M, Perren A, et al. Does spinal chloroprocaine pharmacokinetic profile actually translate into a clinical advantage in terms of clinical outcomes when compared to low-dose spinal bupivacaine? A systematic review and meta-analysis. J Clin Anesth. 2019;52:99-104. | Not included for SR |
| 232 Sau A, Howard JP, Al-Aidarous S, et al. Meta-Analysis of Randomized Controlled Trials of Atrial Fibrillation Ablation With Pulmonary Vein Isolation Versus Without. JACC Clin Electrophysiol. 2019;5(8):968-976. | Not included for SR |
| 233 Schubert A, Buchholt AT, El Khoury AC,et al. Evaluating the costs of glycemic response with canagliflozin versus dapagliflozin and empagliflozin as add-on to metformin in patients with type 2 diabetes mellitus in the United Arab Emirates. Curr Med Res Opin. 2017;33(6):1155-1163. | Not included for SR |
| 234 Schwingshackl L, Hoffmann G, Iqbal K,et al. Food groups and intermediate disease markers: a systematic review and network meta-analysis of randomized trials. Am J Clin Nutr. 2018;108(3):576-586. | Not included for SR |
| 235 Schwingshackl L, Hoffmann G, Lampousi AM, et al. Food groups and risk of type 2 diabetes mellitus: a systematic review and meta-analysis of prospective studies. Eur J Epidemiol. 2017;32(5):363-375. | Not included for SR |

| | |
|---|---|
| 236 Schwingshackl L, Hoffmann G. Adherence to Mediterranean diet and risk of cancer: a systematic review and meta-analysis of observational studies. Int J Cancer. 2014;135(8):1884-1897. | Not included for SR |
| 237 Schwingshackl L, Hoffmann G. Mediterranean dietary pattern, inflammation and endothelial function: a systematic review and meta-analysis of intervention trials. Nutr Metab Cardiovasc Dis. 2014;24(9):929-939. | Not included for SR |
| 238 Schwingshackl L, Hoffmann G. Monounsaturated fatty acids, olive oil and health status: a systematic review and meta-analysis of cohort studies. Lipids Health Dis. 2014;13:154. | Not included for SR |
| 239 Schwingshackl L, Schwedhelm C, Galbete C, Hoffmann G. Adherence to Mediterranean Diet and Risk of Cancer: An Updated Systematic Review and Meta-Analysis. Nutrients. 2017;9(10):1063. | Not included for SR |
| 240 See CJ, McCulloch M, Smikle C, et al. Chinese herbal medicine and clomiphene citrate for anovulation: a meta-analysis of randomized controlled trials. J Altern Complement Med. 2011;17(5):397-405. | Not included for SR |
| 241 Shah PS, Herbozo C, Aliwalas LL,et al. Breastfeeding or breast milk for procedural pain in neonates. Cochrane Database Syst Rev. 2012(12):CD004950. | Review author excluded this study in SR |
| 242 Shahin Y, Khan JA, Samuel N, Chetter I. Angiotensin converting enzyme inhibitors effect on endothelial dysfunction: a meta-analysis of randomised controlled trials. Atherosclerosis. 2011;216(1):7-16. | Not included for SR |
| 243 Shams G, Kazemi A, Jafaryan K, Morowvat MH, Peymani P, Karimzadeh I. Acute kidney injury in COVID-19 patients receiving remdesivir: A systematic review and meta-analysis of randomized clinical trials. Clinics (Sao Paulo). 2023;78:100200. | Review author excluded this study in SR |
| 244 Shang W, Zhang Y, Wang G, Han D. Benefits of continuous positive airway pressure on glycaemic control and insulin resistance in patients with type 2 diabetes and obstructive sleep apnoea: A meta-analysis. Diabetes Obes Metab. 2021;23(2):540-548. | Not included for SR |
| 245 Shantikumar S, Ajjan R, Porter KE, et al. Diabetes and the abdominal aortic aneurysm. Eur J Vasc Endovasc Surg. 2010;39(2):200-207. | Not included for SR |
| 246 Shargorodsky J, Hartnick CJ, Lee GS. Dexamethasone and postoperative bleeding after tonsillectomy and adenotonsillectomy in children: A meta-analysis of prospective studies. Laryngoscope. 2012;122(5):1158-1164. | Review author excluded this study in SR |
| 247 Shi J, Gao Y, Tian J, et al. Negative pressure wound therapy for treating pressure ulcers. Cochrane Database Syst Rev. 2023(5):CD011334. | Not included for SR |
| 248 Shi L, Zhu H, Ma J, et al. Intra-articular magnesium to alleviate postoperative pain after arthroscopic knee surgery: a meta-analysis of randomized controlled trials. J Orthop Surg Res. 2021;16(1):111. | Not included for SR |

| | |
|---|---|
| 249 Shi ZQ, Tang JJ, Wu H, et al. Consumption of nuts and legumes and risk of stroke: a meta-analysis of prospective cohort studies. Nutr Metab Cardiovasc Dis. 2014;24(12):1262-1271. | Not included for SR |
| 250 Shirota M, Watanabe N, Suzuki M, et al. Japanese-Style Diet and Cardiovascular Disease Mortality: A Systematic Review and Meta-Analysis of Prospective Cohort Studies. Nutrients. 2022;14(10):2008. | Not included for SR |
| 251 Siebenhofer A, Jeitler K, Horvath K, et al. Long-term effects of weight-reducing drugs in hypertensive patients. Cochrane Database Syst Rev. 2013(3):CD007654. | Not included for SR |
| 252 Siebenhofer A, Jeitler K, Horvath K, et al. Long-term effects of weight-reducing drugs in people with hypertension. Cochrane Database Syst Rev. 2016(3):CD007654. | Not included for SR |
| 253 Siebenhofer A, Winterholer S, Jeitler K, et al. Long-term effects of weight-reducing drugs in people with hypertension. | Not included for SR |
| 254 Siervo M, Lara J, Chowdhury S, et al. Effects of the Dietary Approach to Stop Hypertension (DASH) diet on cardiovascular risk factors: a systematic review and meta-analysis. Br J Nutr. 2015;113(1):1-15. | Not included for SR |
| 255 Silva R, Pizato N, da Mata F, et al. Mediterranean Diet and Musculoskeletal-Functional Outcomes in Community-Dwelling Older People: A Systematic Review and Meta-Analysis. J Nutr Health Aging. 2018;22(6):655-663. | Not included for SR |
| 256 Silvestri L, Weir WI, Gregori D, et al. Impact of Oral Chlorhexidine on Bloodstream Infection in Critically Ill Patients: Systematic Review and Meta-Analysis of Randomized Controlled Trials. J Cardiothorac Vasc Anesth. 2017;31(6):2236-2244. | Not included for SR |
| 257 Singh B, Parsaik AK, Mielke MM, et al. Association of mediterranean diet with mild cognitive impairment and Alzheimer's disease: a systematic review and meta-analysis. J Alzheimers Dis. 2014;39(2):271-282. | Not included for SR |
| 258 Siordia JA. Beta-Blockers and Abdominal Aortic Aneurysm Growth: A Systematic Review and Meta-Analysis. Curr Cardiol Rev. 2021;17(4):e230421187502. | Not included for SR |
| 259 Sipahi I, Debanne SM, Rowland DY, et al. Angiotensin-receptor blockade and risk of cancer: meta-analysis of randomised controlled trials. Lancet Oncol. 2010;11(7):627-636. | Not included for SR |
| 260 Sofi F, Macchi C, Abbate R, et al. Mediterranean diet and health status: an updated meta-analysis and a proposal for a literature-based adherence score. Public Health Nutr. 2014;17(12):2769-2782. | Not included for SR |
| 261 Solch RJ, Aigbogun JO, Voyiadjis AG, et al. Mediterranean diet adherence, gut microbiota, and Alzheimer's or Parkinson's disease risk: A systematic review. J Neurol Sci. 2022;434:120166. | Not included for SR |
| 262 Sotiriadis A, McGoldrick E, Makrydimas G, et al. Antenatal corticosteroids prior to planned caesarean at term for improving neonatal outcomes. Cochrane Database Syst Rev. 2021(12):CD006614. | Review author excluded this study in SR |
| 263 Souza RJ, Resende JAD Júnior, Miglio CG,et al. Can reducing the number of stitches compromise the outcome of laparoscopic Burch surgery in the treatment of stress urinary incontinence? Systematic review and meta-analysis. Rev Col Bras Cir. 2017;44(6):649-654. | Not included for SR |

| | |
|---|---|
| 264 Steward DL, Welge JA, Myer CM. Do steroids reduce morbidity of tonsillectomy? Meta-analysis of randomized trials. Laryngoscope. 2001;111(10):1712-1718. | Review author excluded this study in SR |
| 265 Straube S, Derry S, McQuay HJ, et al. Effect of preoperative Cox-II-selective NSAIDs (coxibs) on postoperative outcomes: a systematic review of randomized studies. Acta Anaesthesiol Scand. 2005;49(5):601-613. | Not included for SR |
| 266 Straube S, Derry S, McQuay HJ, Moore RA. Effect of preoperative Cox-II-selective NSAIDs (coxibs) on postoperative outcomes: a systematic review of randomized studies. Acta Anaesthesiol Scand. 2005;49(5):601-613. | Not report study-level data |
| 267 Sun X, Zhang X, Nian JY, et al. Chinese Herbal Medicine as Adjunctive Therapy to Chemotherapy for Breast Cancer: A Systematic Review and Meta-Analysis. Evid Based Complement Alternat Med. 2016:3281968. | Not included for SR |
| 268 Sun YE, Wang W, Qin J. Anti-hyperlipidemia of garlic by reducing the level of total cholesterol and low-density lipoprotein: A meta-analysis. Medicine (Baltimore). 2018;97(18):e0255. | Not included for SR |
| 269 Sun YQ, Wei Q, Liu Z. Efficacy of Continuous Positive Airway Pressure in the Treatment of Chronic Obstructive Pulmonary Disease Combined With Respiratory Failure. Am J Ther. 2016;23(2):e439-e450. | Not included for SR |
| 270 Takagi H, Umemoto T; ALICE (All-Literature Investigation of Cardiovascular Evidence) Group. Diabetes and Abdominal Aortic Aneurysm Growth. Angiology. 2016;67(6):513-525. | Not included for SR |
| 271 Tang C, Wang X, Qin LQ, et al. Mediterranean Diet and Mortality in People with Cardiovascular Disease: A Meta-Analysis of Prospective Cohort Studies. Nutrients. 2021;13(8):2623. | Not included for SR |
| 272 Tarro L, Llauradó E, Ulldemolins G, et al. Effectiveness of Workplace Interventions for Improving Absenteeism, Productivity, and Work Ability of Employees: A Systematic Review and Meta-Analysis of Randomized Controlled Trials. Int J Environ Res Public Health. 2020;17(6):1901. | Not included for SR |
| 273 Taverny G, Mimouni Y, LeDigarcher A, et al. Antihypertensive pharmacotherapy for prevention of sudden cardiac death in hypertensive individuals. Cochrane Database Syst Rev. 2016(3):CD011745. | Review author excluded this study in SR |
| 274 Thangatorai R, Lim FC, Nalliah S. Cervical pessary in the prevention of preterm births in multiple pregnancies with a short cervix: PRISMA compliant systematic review and meta-analysis. J Matern Fetal Neonatal Med. 2018 Jun;31(12):1638-1645. | Not included for SR |
| 275 Thomas Manapurathe D, Krishna SM, Dewdney B, et al. Effect of blood pressure lowering medications on leg ischemia in peripheral artery disease patients: A meta-analysis of randomised controlled trials. PLoS One. 2017;12(6):e0178713. | Not included for SR |
| 276 Thomas Manapurathe D, Krishna SM, Dewdney B, et al. Effect of blood pressure lowering medications on leg ischemia in peripheral artery disease patients: A meta-analysis of randomised controlled trials. PLoS One. 2017;12(6):e0178713. | Not included for SR |
| 277 Tian Z., Yan Y., Deng S., et al. Effect of valsartan on insulin resistance in patients with hypertension: a systematic review and meta-analysis. Int J Clin Exp Med, 9(7), 14047-14056. | Not included for SR |

| | |
|---|---|
| 278 Tierney AC, Rumble CE, Billings LM, et al. Effect of Dietary and Supplemental Lycopene on Cardiovascular Risk Factors: A Systematic Review and Meta-Analysis. Adv Nutr. 2020;11(6):1453-1488. | Not included for SR |
| 279 Tiippana EM, Hamunen K, Kontinen VK, et al. Do surgical patients benefit from perioperative gabapentin/pregabalin? A systematic review of efficacy and safety. Anesth Analg. 2007;104(6). | Not included for SR |
| 280 van Hoogenhuijze NE, Lahoz Casarramona G, Lensen S, et al. Endometrial scratching in women undergoing IVF/ICSI: an individual participant data meta-analysis. Hum Reprod Update. 2023;29(6):721-740. | Not included for SR |
| 281 Vaughan J, Nagendran M, Cooper J,et al. Anaesthetic regimens for day-procedure laparoscopic cholecystectomy. Cochrane Database Syst Rev.2014(1):CD009784. | Review author excluded this study in SR |
| 282 Verheyden GS, Weerdesteyn V, Pickering RM, et al. Interventions for preventing falls in people after stroke. Cochrane Database Syst Rev. 2013(5):CD008728. | Not included for SR |
| 283 Veronese N, Watutantrige-Fernando S, Luchini C, et al. Effect of magnesium supplementation on glucose metabolism in people with or at risk of diabetes: a systematic review and meta-analysis of double-blind randomized controlled trials. Eur J Clin Nutr. 2016;70(12):1354-1359. | Not included for SR |
| 284 Vitagliano A, Di Spiezio Sardo A, Saccone G, et al. Endometrial scratch injury for women with one or more previous failed embryo transfers: a systematic review and meta-analysis of randomized controlled trials. Fertil Steril. 2018;110(4):687-702.e2. | Not included for SR |
| 285 Vitagliano A, Noventa M, Saccone G, et al. Endometrial scratch injury before intrauterine insemination: is it time to re-evaluate its value? Evidence from a systematic review and meta-analysis of randomized controlled trials. Fertil Steril. 2018;109(1):84-96.e4. | Not included for SR |
| 286 Wagnew F, Alene KA, Eshetie S, et al. Effects of zinc and vitamin A supplementation on prognostic markers and treatment outcomes of adults with pulmonary tuberculosis: a systematic review and meta-analysis. BMJ Glob Health. 2022;7(9):e008625. | Not included for SR |
| 287 Wali S, Balfoussia D, Touqmatchi D, Quinn S. Misoprostol for open myomectomy: a systematic review and meta-analysis of randomised control trials. BJOG. 2021;128(3):476-483. | Not included for SR |
| 288 Wang C, Huang T, Song W, et al. A meta-analysis of the relationship between polycystic ovary syndrome and sleep disturbances risk. Front Physiol. 2022;13:957112. | Not included for SR |
| 289 Wang F, Zhao D, Yang Y, et al. Effect of palm oil consumption on plasma lipid concentrations related to cardiovascular disease: a systematic review and meta-analysis. Asia Pac J Clin Nutr. 2019;28(3):495-506. | Included but not synthesized/presented |
| 290 Wang J, Lv S, Chen G, et al. Meta-analysis of the association between vitamin D and autoimmune thyroid disease. Nutrients. 2015;7(4):2485-2498. | Not included for SR |
| 291 Wang R, Li W, Bordewijk EM, et al. First-line ovulation induction for polycystic ovary syndrome: an individual participant data meta-analysis. Hum Reprod Update. 2019;25(6):717-732. | Not included for SR |

| | |
|---|---|
| 292 Wang W, Zhou L, Wu LX,et al. 5-HT3 Receptor Antagonists for Propofol Injection Pain: A Meta-Analysis of Randomized Controlled Trials. Clin Drug Investig. 2016;36(4):243-253. | Not included for SR |
| 293 Wang XR, Xiao JP, Zhang JJ,et al. Decreased Serum/Plasma Vitamin D levels in SLE Patients: A Meta-Analysis. Curr Pharm Des. 2018;24(37):4466-4473. | Not included for SR |
| 294 Wang XX, Zhou Q, Pan DB, et al. Comparison of Postoperative Events between Spinal Anesthesia and General Anesthesia in Laparoscopic Cholecystectomy: A Systemic Review and Meta-Analysis of Randomized Controlled Trials. Biomed Res Int. 2016;2016:9480539. | Review author excluded this study in SR |
| 295 Wang Y, Shen Q, Wang C. Efficacy of Rapid Rehabilitation Nursing in Postoperative Care in China: A Meta-Analysis. Rehabil Nurs. 2023;48(5):170-179. | Not included for SR |
| 296 Wang Y, Xu L, Wang L, et al. Effects of transcutaneous neuromuscular electrical stimulation on post-stroke dysphagia: a systematic review and meta-analysis. Front Neurol. 2023;14:1163045. | Not included for SR |
| 297 Ward-Caviness CK, Xu T, Aspelund T, et al. Improvement of myocardial infarction risk prediction via inflammation-associated metabolite biomarkers. Heart. 2017;103(16):1278-1285. | Not included for SR |
| 298 Wei J, He L, Weng F, Huang F, Teng P. Effectiveness of chlorhexidine in preventing infections among patients undergoing cardiac surgeries: a meta-analysis and systematic review. Antimicrob Resist Infect Control. 2021;10(1):140. | Not included for SR |
| 299 Wells GA, Cranney A, Peterson J, et al. Etidronate for the primary and secondary prevention of osteoporotic fractures in postmenopausal women. Cochrane Database Syst Rev.2008(1):CD003376. | Not included for SR |
| 300 Wells GA, Hsieh SC, Peterson J, et al. Etidronate for the primary and secondary prevention of osteoporotic fractures in postmenopausal women. Cochrane Database Syst Rev. 2024(4):CD003376. | Not included for SR |
| 301 Wex J, Abou-Setta AM. Economic evaluation of highly purified human menopausal gonadotropin versus recombinant human follicle-stimulating hormone in fresh and frozen in vitro fertilization/intracytoplasmic sperm-injection cycles in Sweden. Clinicoecon Outcomes Res. 2013;5:381-397. | Not included for SR |
| 302 Wiedermann CJ, Dunzendorfer S, Gaioni LU, et al. Hyperoncotic colloids and acute kidney injury: a meta-analysis of randomized trials. Crit Care. 2010;14(5):R191. | Not included for SR |
| 303 Wiedermann CJ, Joannidis M. Mortality after hydroxyethyl starch 130/0.4 infusion: an updated meta-analysis of randomized trials. Swiss Med Wkly. 2012;142:w13656. | Not included for SR |
| 304 Wijeysundera DN, Bender JS, Beattie WS. Alpha-2 adrenergic agonists for the prevention of cardiac complications among patients undergoing surgery. Cochrane Database Syst Rev. 2009(4):CD004126. | Review author excluded this study in SR |

| | |
|---|---|
| 305 Wojcieszek AM, Shepherd E, Middleton P, et al. Care prior to and during subsequent pregnancies following stillbirth for improving outcomes. Cochrane Database Syst Rev. 2018(12):CD012203. | Review author excluded this study in SR |
| 306 Wojcieszek AM, Shepherd E, Middleton P, et al. Care prior to and during subsequent pregnancies following stillbirth for improving outcomes. Cochrane Database Syst Rev. 2018(12):CD012203. | Review author excluded this study in SR |
| 307 Wong WT, Lai VK, Chee YE,et al. Fast-track cardiac care for adult cardiac surgical patients. Cochrane Database Syst Rev. 2016(9):CD003587. | Not included for SR |
| 308 Wu L, Sun D. Adherence to Mediterranean diet and risk of developing cognitive disorders: An updated systematic review and meta-analysis of prospective cohort studies. Sci Rep. 2017:41317. | Not included for SR |
| 309 Wu L, Sun D. Consumption of Yogurt and the Incident Risk of Cardiovascular Disease: A Meta-Analysis of Nine Cohort Studies. Nutrients. 2017;9(3):315. | Not included for SR |
| 310 Wu QL, Liu QZ, Xi YY, et al. Closed or Unclosed Mesentery? A Meta-analysis of Internal Herniation After Laparoscopic Roux-en-Y Gastric Bypass. Obes Surg. 2023;33(6):1900-1909. | Not included for SR |
| 311 Wu SJ, Xiong XZ, Lin YX, et al. Comparison of the efficacy of ondansetron and granisetron to prevent postoperative nausea and vomiting after laparoscopic cholecystectomy: a systematic review and meta-analysis. Surg Laparosc Endosc Percutan Tech. 2013;23(1):79-87. | Not included for SR |
| 312 Wu Z, Chen F, Yu F, et al. A meta-analysis of obstructive sleep apnea in patients with cerebrovascular disease. Sleep Breath. 2018;22(3):729-742. | Not included for SR |
| 313 Xia Y, Fang H, Xu J,et al. Clinical efficacy of xenon versus propofol: A systematic review and meta-analysis. Medicine (Baltimore). 2018 ;97(20):e10758. | Not included for SR |
| 314 Xiao Y, Xia J, Ke Y, et al. Effects of nut consumption on selected inflammatory markers: a systematic review and meta-analysis of randomized controlled trials. Nutrition. 2018;54:129-143. | Not included for SR |
| 315 Xie M, Li XK, Peng Y. Magnesium sulfate for postoperative complications in children undergoing tonsillectomies: a systematic review and meta-analysis. J Evid Based Med. 2017;10(1):16-25. | Not included for SR |
| 316 Xu B, Sui Y, Zhu C, et al. Music intervention on cognitive dysfunction in healthy older adults: a systematic review and meta-analysis. Neurol Sci. 2017;38(6):983-992. | Not included for SR |
| 317 Xu JY, Qin LQ, Wang PY, et al. Effect of milk tripeptides on blood pressure: a meta-analysis of randomized controlled trials. Nutrition. 2008;24(10):933-940. | Not included for SR |
| 318 Xu Y, Song Y, Sun D, et al. Effect of Multi-Modal Therapies for Kinesiophobia Caused by Musculoskeletal Disorders: A Systematic Review and Meta-Analysis. Int J Environ Res Public Health. 2020;17(24):9439. | Not included for SR |

| | |
|---|---|
| 319 Yang B, Li DL, Dong P, et al. Effect of dexamethasone on the incidence of post-dural puncture headache after spinal anesthesia: a randomized, double-blind, placebo-controlled trial and a meta-analysis. Acta Neurol Belg. 2015;115(1):59-67. | Review author excluded this study in SR |
| 320 Yang F, Lees J, Simpkins C, et al. Interventions for preventing falls in people post-stroke: A meta-analysis of randomized controlled trials. Gait Posture. 2021;84:377-388. | Not included for SR |
| 321 Yang S, Zhou Z, Miao H,et al. Effect of weight loss on blood pressure changes in overweight patients: A systematic review and meta-analysis. J Clin Hypertens (Greenwich). 2023;25(5):404-415. | Not included for SR |
| 322 Ye X, Huang J, Pan Q, et al. Maintenance therapy with immunomodulatory drugs after autologous stem cell transplantation in patients with multiple myeloma: a meta-analysis of randomized controlled trials. PLoS One. 2013;8(8):e72635. | Not included for SR |
| 323 Yu SH, Beirne OR. Laryngeal mask airways have a lower risk of airway complications compared with endotracheal intubation: a systematic review. J Oral Maxillofac Surg. 2010;68(10):2359-2376. | Not included for SR |
| 324 Zacharias M, Conlon NP, Herbison GP, et al. Interventions for protecting renal function in the perioperative period. Cochrane Database Syst Rev. 2008(4):CD003590. | Not included for SR |
| 325 Zacharias M, Mugawar M, Herbison GP, et al. Interventions for protecting renal function in the perioperative period. Cochrane Database Syst Rev. 2013(9):CD003590. | Not included for SR |
| 326 Zeng B, Gao L, Yang Q, et al. Automated Insulin Delivery Systems in Children and Adolescents With Type 1 Diabetes: A Systematic Review and Meta-analysis of Outpatient Randomized Controlled Trials. Diabetes Care. 2023;46(12):2300-2307. | Not included for SR |
| 327 Zhang L, Quan M, Cao ZB. Effect of vitamin D supplementation on upper and lower limb muscle strength and muscle power in athletes: A meta-analysis. PLoS One. 2019;14(4):e0215826. | Not included for SR |
| 328 Zhang LK, Ma JX, Kuang MJ, et al. Comparison of Periarticular Local Infiltration Analgesia With Femoral Nerve Block for Total Knee Arthroplasty: a Meta-Analysis of Randomized Controlled Trials. J Arthroplasty. 2018;33(6):1972-1978.e4. | Not included for SR |
| 329 Zhang Y, Fang F, Tang J, et al. Association between vitamin D supplementation and mortality: systematic review and meta-analysis [published correction appears in BMJ. 2020;370:m2329. | Not included for SR |
| 330 Zhang Y, Zhang JW, Wang BH. Efficacy of tranexamic acid plus drain-clamping to reduce blood loss in total knee arthroplasty: A meta-analysis. Medicine (Baltimore). 2017;96(26):e7363. | Not included for SR |
| 331 Zhang Y, Zhang X, Wang Y, et al. Effect of Clonidine on Hemodynamic Responses During Laparoscopic Cholecystectomy: A Systematic Review and Meta-Analysis. Surg Laparosc Endosc Percutan Tech. 2017;27(5):335-340. | Not included for SR |
| 332 Zhang Z, Xu G, Wei Y, et al. Nut consumption and risk of stroke. Eur J Epidemiol. 2015;30(3):189-196. | Not included for SR |
| 333 Zhao S, Qiu Z, He J, et al. Insulin-like growth factor receptor 1 (IGF1R) expression and survival in non-small cell lung cancer patients: a meta-analysis. Int J Clin Exp Pathol. 2014;7(10):6694-6704. | Not included for SR |

| | |
|---|---|
| 334 Zhou G, Ma L, Jing J, et al. A meta-analysis of dexamethasone for pain management in patients with total knee arthroplasty. Medicine (Baltimore). 2018;97(35):e11753. | Not included for SR |
| 335 Zhou LS, Xu LJ, Wang XQ,et al. Effect of Angiotensin-Converting Enzyme Inhibitors on Physical Function in Elderly Subjects: A Systematic Review and Meta-Analysis. Drugs Aging. 2015;32(9):727-735. | Not included for SR |
| 336 Zhu F, Lee A, Chee YE. Fast-track cardiac care for adult cardiac surgical patients. Cochrane Database Syst Rev. 2012;10:CD003587. | Not included for SR |
| 337 Zhu L, Mi Y, You X, et al. A meta-analysis of the effects of the 5-hydroxytryptamine transporter gene-linked promoter region polymorphism on susceptibility to lifelong premature ejaculation. PLoS One. 2013;8(1):e54994. | Not included for SR |
| 338 Zhu Y, Bo Y, Liu Y. Dietary total fat, fatty acids intake, and risk of cardiovascular disease: a dose-response meta-analysis of cohort studies. Lipids Health Dis. 2019;18(1):91. | Not included for SR |
| 339 Zhu Y, Feng Y, Peng L. Effect of transcutaneous electrical nerve stimulation for pain control after total knee arthroplasty: A systematic review and meta-analysis. J Rehabil Med. 2017;49(9):700-704. | Not included for SR |
| 340 Zhuang Q, Chen F, Wang T. Effectiveness of short message service intervention to improve glycated hemoglobin control and medication adherence in type-2 diabetes: A meta-analysis of prospective studies. Prim Care Diabetes. 2020;14(4):356-363. | Not included for SR |
| 341 Chong MA, Wang Y, Berbenetz NM, McConachie I. Does goal-directed haemodynamic and fluid therapy improve peri-operative outcomes?: A systematic review and meta-analysis. Eur J Anaesthesiol. 2018;35(7):469-483. | Not included for SR |
| 342 Fazelian S, Rouhani MH, Bank SS, et al. Chromium supplementation and polycystic ovary syndrome: A systematic review and meta-analysis. J Trace Elem Med Biol. 2017;42:92-96. | Not included for SR |
| 343 Novara G, Galfano A, Boscolo-Berto R, et al. Complication rates of tension-free midurethral slings in the treatment of female stress urinary incontinence: a systematic review and meta-analysis of randomized controlled trials comparing tension-free midurethral tapes to other surgical procedures and different devices. Eur Urol. 2008;53(2):288-308. | Not included for SR |
| 344 Eberhart L H J, Morin A M, Bothner U, et al. Droperidol compared to 5-HT3-antagonists for prophylaxis of postoperative nausea and vomiting-A meta-analysis of controlled randomised studies. ANASTHESIOLOGIE & INTENSIVMEDIZIN, 2001, 42(2): 58-69. | Foreign language |
| 345 Eberhart LH, Morin AM, Georgieff M. Dexamethason zur Prophylaxe von Ubelkeit und Erbrechen in der postoperativen Phase. Eine Metaanalyse kontrollierter randomisierter Studien [Dexamethasone for prophylaxis of postoperative nausea and vomiting. A meta-analysis of randomized controlled studies]. Anaesthesist. 2000;49(8):713-720. | Foreign language |
| 346 Kranke P, Eberhart L H J, Morin A M, et al. Dolasetron in the prevention of postoperative nausea and vomiting-A meta-analysis of randomized controlled trials. ANASTHESIOLOGIE & INTENSIVMEDIZIN, 2002, 43(7-8): 413-427. | Foreign language |

| | |
|---|---|
| 347 Mukai J, Tada H, Miura M, et al. Meta-analysis-based examination regarding the efficacy of angiotensin II receptor blockers and calcium channel blockers in borderline diabetes and diabetes patients. Yakugaku Zasshi: Journal of the Pharmaceutical Society of Japan, 2011, 131(8): 1213-1223. | Foreign language |
| 348 Sánchez Muñoz-Torrero JF, Lorenzo-Hernández A, et al. Natural history of venous thromboembolism in patients from the Mediterranean region. A systematic review. Rev Clin Esp (Barc). 2014;214(4):184-191. | Foreign language |
| 349 Wang MM, Chen ZJ, Wang Y, et al.Effects of Vitamin D Supplementation on Serum Lipid Profiles and Neonatal Outcomes in Gestational Diabetes Mellitus:a Meta-analysis. Zhongguo Yi Xue Ke Xue Yuan Xue Bao. 2021;43(1):82-91. | Foreign language |
| 350 Abiri B, Koohi F, Ebadinejad A, et al. Transition from metabolically healthy to unhealthy overweight/obesity and risk of cardiovascular disease incidence: A systematic review and meta-analysis. Nutr Metab Cardiovasc Dis. 2022;32(9):2041-2051. | Not included for SR |
| 351 Abu-Zaid A, Gaman MA, Jamilian P, et al. The effect of 17β-estradiol plus norethisterone acetate treatment on the lipid profile in women: a dose-response meta-analysis of randomized controlled trials. Exp Gerontol. 2022;165:111855. | Not included for SR |
| 352 Afsharpaiman S, Zare M, Yasemi M, et al. The Prevalence of Infectious Keratitis after Keratorefractive Surgery: A Systematic Review and Meta-Analysis Study. J Ophthalmol. 2020:6329321. | Not included for SR |
| 353 Afshin A, Peñalvo JL, Del Gobbo L, et al. The prospective impact of food pricing on improving dietary consumption: A systematic review and meta-analysis. PLoS One. 2017;12(3):e0172277. | Not included for SR |
| 354 Al Farii H, McChesney G, Patel SS, et al. The risk of neurological deterioration while using neoadjuvant denosumab on patients with giant cell tumor of the spine presenting with epidural disease: a meta-analysis of the literature. Spine J. 2024;24(6):1056-1064. | Not included for SR |
| 355 Albrecht E, Guyen O, Jacot-Guillarmod A, et al. The analgesic efficacy of local infiltration analgesia vs femoral nerve block after total knee arthroplasty: a systematic review and meta-analysis. Br J Anaesth. 2016;116(5):597-609. | Not included for SR |
| 356 Albuloshi T, Dimala C A, Kuhnle G G C, et al. The effectiveness of vitamin D supplementation in reducing depressive symptoms: A systematic review and meta-analysis of Randomized Controlled Trials (RCTs). Nutrition and Healthy Aging, 2021, 6(4): 301-318. | Not included for SR |
| 357 Amani B, Amani B. Efficacy and safety of regdanvimab in patients with mild to moderate COVID-19: A rapid review and meta-analysis. Br J Clin Pharmacol. 2023;89(4):1282-1290. | Not included for SR |
| 358 Androulakis-Korakakis P, Fisher JP, Steele J. The Minimum Effective Training Dose Required to Increase 1RM Strength in Resistance-Trained Men: A Systematic Review and Meta-Analysis. Sports Med. 2020;50(4):751-765. | Not included for SR |
| 359 Antoniak AE, Greig CA. The effect of combined resistance exercise training and vitamin D3 supplementation on musculoskeletal health and function in older adults: a systematic review and meta-analysis. BMJ Open. 2017;7(7):e014619. | Not included for SR |
| 360 Apóstolo J, Bobrowicz-Campos E, Rodrigues M, et al. The effectiveness of non-pharmacological interventions in older adults with depressive disorders: A systematic review. Int J Nurs Stud. 2016;58:59-70. | Not included for SR |

| | |
|---|---|
| 361 Asbaghi O, Sadeghian M, Mozaffari-Khosravi H, et al. The effect of vitamin d-calcium co-supplementation on inflammatory biomarkers: A systematic review and meta-analysis of randomized controlled trials. Cytokine. 2020;129:155050. | Not included for SR |
| 362 Autier P, Gandini S. Vitamin D supplementation and total mortality: a meta-analysis of randomized controlled trials. Arch Intern Med. 2007;167(16):1730-1737. | Not included for SR |
| 363 Avenell A, Mak JC, O'Connell D. Vitamin D and vitamin D analogues for preventing fractures in post-menopausal women and older men. Cochrane Database Syst Rev. 2014(4):CD000227. | Not included for SR |
| 364 Balion C, Griffith LE, Strifler L, et al. Vitamin D, cognition, and dementia: a systematic review and meta-analysis. Neurology. 2012;79(13):1397-1405. | Not included for SR |
| 365 Bashir Y, Al-Awaysheh M M H, ul Ain Q, et al. Systematic Review & Meta-Analysis of Randomized Control Trials Comparing Stapled Hemorrhoidectomy versus Trans Anal Hemorrhoidal Dearterialization for Treatment of Hemorrhoidal Disease. Surgery, Gastroenterology and Oncology, 2022, 27(1): 5. | Not included for SR |
| 366 Batchelor F, Hill K, Mackintosh S, et al. What works in falls prevention after stroke?: a systematic review and meta-analysis. Stroke. 2010;41(8):1715-1722. | Not included for SR |
| 367 Becerra-Tomás N, Blanco Mejía S, Viguiliouk E, et al. Mediterranean diet, cardiovascular disease and mortality in diabetes: A systematic review and meta-analysis of prospective cohort studies and randomized clinical trials. Crit Rev Food Sci Nutr. 2020;60(7):1207-1227. | Not included for SR |
| 368 Bidonde J, Busch AJ, van der Spuy I, et al. Whole body vibration exercise training for fibromyalgia. Cochrane Database Syst Rev. 2017;9(9):CD011755. | Not included for SR |
| 369 Bitterman R, Hussein K, Leibovici L, et al. Systematic review of antibiotic consumption in acute care hospitals. Clin Microbiol Infect. 2016;22(6):561.e7-561.e19. | Not included for SR |
| 370 Bordewijk EM, Wang R, van Wely M, et al. To share or not to share data: how valid are trials evaluating first-line ovulation induction for polycystic ovary syndrome?. Hum Reprod Update. 2020;26(6):929-941. | Not included for SR |
| 371 Cai H, Fan X, Feng P, et al. Optimal dose of perineural dexmedetomidine to prolong analgesia after brachial plexus blockade: a systematic review and Meta-analysis of 57 randomized clinical trials. BMC Anesthesiol. 2021;21(1):233. | Not included for SR |
| 372 Caissutti C, Saccone G, Zullo F, et al. Vaginal Cleansing Before Cesarean Delivery: A Systematic Review and Meta-analysis. Obstet Gynecol. 2017;130(3):527-538. | Not included for SR |
| 373 Camacho M, Chang ET, Song SA, et al. Rapid maxillary expansion for pediatric obstructive sleep apnea: A systematic review and meta-analysis. Laryngoscope. 2017;127(7):1712-1719. | Not included for SR |
| 374 Carducci B, Keats EC, Bhutta ZA. Zinc supplementation for improving pregnancy and infant outcome. Cochrane Database Syst Rev. 2021;3(3):CD000230. | Not included for SR |

| | |
|---|---|
| 375 Chen GC, Zhang R, Martínez-González MA, et al. Nut consumption in relation to all-cause and cause-specific mortality: a meta-analysis 18 prospective studies. Food Funct. 2017;8(11):3893-3905. | Not included for SR |
| 376 Chiavaroli L, Nishi SK, Khan TA, et al. Portfolio Dietary Pattern and Cardiovascular Disease: A Systematic Review and Meta-analysis of Controlled Trials. Prog Cardiovasc Dis. 2018;61(1):43-53. | Not included for SR |
| 377 Choo YT, Jiang Y, Hong J, et al. Effectiveness of Tai Chi on quality of life, depressive symptoms and physical function among community-dwelling older adults with chronic disease: A systematic review and meta-analysis. Int J Nurs Stud. 2020;111:103737. | Not included for SR |
| 378 Costi D, Cyna AM, Ahmed S, et al. Effects of sevoflurane versus other general anaesthesia on emergence agitation in children. Cochrane Database Syst Rev.2014(9):CD007084. | Not included for SR |
| 379 Dai J, Jiang C, Chen H, et al. Vitamin D and diabetic foot ulcer: a systematic review and meta-analysis. Nutr Diabetes. 2019;9(1):8. | Not included for SR |
| 380 De Oliveira GS Jr, Castro-Alves LJ, Chang R, et al. Systemic metoclopramide to prevent postoperative nausea and vomiting: a meta-analysis without Fujii's studies. Br J Anaesth. 2012;109(5):688-697. | Not included for SR |
| 381 Dehkordi A H, Dousti M, Kiani F, et al. Tumor necrosis factor-alpha 308 G/A polymorphism and type 2 diabetes mellitus; a systematic review and meta-analysis. Journal of nephropathology, 2018, 7(1). | Not included for SR |
| 382 Desjardins PJ, Mehlisch DR, Chang DJ, et al. The time to onset and overall analgesic efficacy of rofecoxib 50 mg: a meta-analysis of 13 randomized clinical trials. Clin J Pain. 2005;21(3):241-250. | Not included for SR |
| 383 Devall AJ, Papadopoulou A, Podesek M, et al. Progestogens for preventing miscarriage: a network meta-analysis. Cochrane Database Syst Rev. 2021(4):CD013792. | Not included for SR |
| 384 Dinoff A, Herrmann N, Swardfager W, et al. The effect of acute exercise on blood concentrations of brain-derived neurotrophic factor in healthy adults: a meta-analysis. Eur J Neurosci. 2017;46(1):1635-1646. | Not included for SR |
| 385 Dong JY, Zhang YH, Qin LQ. Obstructive sleep apnea and cardiovascular risk: meta-analysis of prospective cohort studies. Atherosclerosis. 2013;229(2):489-495. | Not included for SR |
| 386 Dong T, Guo M, Zhang P, et al. The effects of low-carbohydrate diets on cardiovascular risk factors: A meta-analysis. PLoS One. 2020;15(1):e0225348. | Not included for SR |
| 387 Eberhart LH, Frank S, Lange H, et al. Systematic review on the recurrence of postoperative nausea and vomiting after a first episode in the recovery room - implications for the treatment of PONV and related clinical trials. BMC Anesthesiol. 2006;6:14. | Not included for SR |
| 388 Fan L, Zhu C, Zan P, et al. The Comparison of Local Infiltration Analgesia with Peripheral Nerve Block following Total Knee Arthroplasty (TKA): A Systematic Review with Meta-Analysis. J Arthroplasty. 2015;30(9):1664-1671. | Not included for SR |
| 389 Feng Y, Huang R, Kavanagh J, et al. Efficacy and Safety of Dual Blockade of the Renin-Angiotensin-Aldosterone System in Diabetic Kidney Disease: A Meta-Analysis. Am J Cardiovasc Drugs. 2019;19(3):259-286. | Not included for SR |

| | |
|---|---|
| 390 Ferrara R, Imbimbo M, Malouf R, et al. Single or combined immune checkpoint inhibitors compared to first-line platinum-based chemotherapy with or without bevacizumab for people with advanced non-small cell lung cancer. Cochrane Database Syst Rev. 2020(12):CD013257. | Not included for SR |
| 391 Ferrara R, Imbimbo M, Malouf R, et al. Single or combined immune checkpoint inhibitors compared to first-line platinum-based chemotherapy with or without bevacizumab for people with advanced non-small cell lung cancer. Cochrane Database Syst Rev. 2021(4):CD013257. | Not included for SR |
| 392 Figueredo E, Canosa L. Prophylactic ondansetron for postoperative emesis. Meta-analysis of its effectiveness in patients with previous history of postoperative nausea and vomiting. Acta Anaesthesiol Scand. 1999;43(6):637-644. | Not included for SR |
| 393 Figueredo E, Canosa L. Prophylactic ondansetron for post-operative emesis: meta-analysis of its effectiveness in patients with and without a previous history of motion sickness. Eur J Anaesthesiol. 1999;16(8):556-564. | Not included for SR |
| 394 Flores-Mateo G, Rojas-Rueda D, Basora J, et al. Nut intake and adiposity: meta-analysis of clinical trials. Am J Clin Nutr. 2013;97(6):1346-1355. | Not included for SR |
| 395 Forget P, Borovac JA, Thackeray EM, Pace NL. Transient neurological symptoms (TNS) following spinal anaesthesia with lidocaine versus other local anaesthetics in adult surgical patients: a network meta-analysis. Cochrane Database Syst Rev. 2019;12(12):CD003006. | Not included for SR |
| 396 Free C, Phillips G, Galli L, et al. The effectiveness of mobile-health technology-based health behaviour change or disease management interventions for health care consumers: a systematic review. PLoS Med. 2013;10(1):e1001362. | Not included for SR |
| 397 French DP, Olander EK, Chisholm A, et al. Which behaviour change techniques are most effective at increasing older adults' self-efficacy and physical activity behaviour? A systematic review. Ann Behav Med. 2014;48(2):225-234. | Not included for SR |
| 398 Gao L, Chen L, Fan L, et al. The effect of losartan on progressive aortic dilatation in patients with Marfan's syndrome: a meta-analysis of prospective randomized clinical trials. Int J Cardiol. 2016;217:190-194. | Not included for SR |
| 399 Gao M, Kong Y, Wang H, et al. Thalidomide treatment for patients with previously untreated multiple myeloma: a meta-analysis of randomized controlled trials. Tumour Biol. 2016;37(8):11081-11098. | Not included for SR |
| 400 George RB, Allen TK, Habib AS. Serotonin receptor antagonists for the prevention and treatment of pruritus, nausea, and vomiting in women undergoing cesarean delivery with intrathecal morphine: a systematic review and meta-analysis. Anesth Analg. 2009;109(1):174-182. | Not included for SR |
| 401 Ghaffari J, Alizadeh-Navaei R, Dabaghzadeh A, et al. Serum zinc level and children`s asthma: A systematic and meta-analysis review article. Caspian J Intern Med. 2021;12(3):236-242. | Not included for SR |
| 402 Ghiasi B, Sarokhani D, Dehkordi AH, et al. Quality of Life of patients with chronic kidney disease in Iran: Systematic Review and Meta-analysis. Indian J Palliat Care. 2018;24(1):104-111. | Not included for SR |

| | |
|---|---|
| 403 Gok Metin Z, Izgu N, Gulbahar Eren M, et al. Theory-based nursing interventions in adults with coronary heart disease: A systematic review and meta-analysis of randomized controlled trials. J Nurs Scholarsh. 2023;55(2):439-463. | Not included for SR |
| 404 Gorji Z, Varkaneh HK, Talaei S, et al. The effect of green-coffee extract supplementation on obesity: A systematic review and dose-response meta-analysis of randomized controlled trials.Phytomedicine. 2020;68:153199. | Not included for SR |
| 405 Grant MC, Kim J, Page AJ, et al. The Effect of Intravenous Midazolam on Postoperative Nausea and Vomiting: A Meta-Analysis. Anesth Analg. 2016;122(3):656-663. | Not included for SR |
| 406 Gravestock P, Coulthard N, Veeratterapillay R, et al. Systematic review and meta-analysis of narrow band imaging for non-muscle-invasive bladder cancer. Int J Urol. 2021;28(12):1212-1217. | Not included for SR |
| 407 Grosso G, Yang J, Marventano S, et al. Nut consumption on all-cause, cardiovascular, and cancer mortality risk: a systematic review and meta-analysis of epidemiologic studies. Am J Clin Nutr. 2015;101(4):783-793. | Not included for SR |
| 408 He J, Deng R, Wei Y, et al. Efficacy of antioxidant supplementation in improving endocrine, hormonal, inflammatory, and metabolic statuses of PCOS: a meta-analysis and systematic review. Food Funct. 2024;15(4):1779-1802. | Not included for SR |
| 409 Heinzel S, Lawrence J B, Kallies G, et al. Using exercise to fight depression in older adults: A systematic review and meta-analysis. GeroPsych: The Journal of Gerontopsychology and Geriatric Psychiatry, 28 (4), 149–162. 2015. | Not included for SR |
| 410 Houston L, Probst YC, Chandra Singh M, et al. Tree Nut and Peanut Consumption and Risk of Cardiovascular Disease: A Systematic Review and Meta-Analysis of Randomized Controlled Trials. Adv Nutr. 2023;14(5):1029-1049. | Not included for SR |
| 411 Hu C, Deng C, Zou W, et al. The Role of Consolidative Radiotherapy after a Complete Response to Chemotherapy in the Treatment of Diffuse Large B-Cell Lymphoma in the Rituximab Era: Results from a Systematic Review with a Meta-Analysis. Acta Haematol. 2015;134(2):111-118. | Not included for SR |
| 412 Hua K, Cummings M, Bernatik M,et al. Cardiovascular effects of auricular stimulation -a systematic review and meta-analysis of randomized controlled clinical trials. Front Neurosci. 2023;17:1227858. | Not included for SR |
| 413 Hurley J. Rebound Inverts the Staphylococcus aureus Bacteremia Prevention Effect of Antibiotic Based Decontamination Interventions in ICU Cohorts with Prolonged Length of Stay. Antibiotics (Basel). 2024;13(4):316. | Not included for SR |
| 414 Hurley JC. Studies of selective digestive decontamination as a natural experiment to evaluate topical antibiotic prophylaxis and cephalosporin use as population-level risk factors for enterococcal bacteraemia among ICU patients. J Antimicrob Chemother. 2019;74(10):3087-3094. | Not included for SR |
| 415 Hurley JC. Topical antibiotics as a major contextual hazard toward bacteremia within selective digestive decontamination studies: a meta-analysis. BMC Infect Dis. 2014;14:714. | Not included for SR |
| 416 Iavazzo C, Mamais I, Gkegkes ID. Use of misoprostol in myomectomy: a systematic review and meta-analysis. Arch Gynecol Obstet. 2015;292(6):1185-1191. | Not included for SR |

| | |
|---|---|
| 417 Islam MA, Khandker SS, Alam SS, et al. Vitamin D status in patients with systemic lupus erythematosus (SLE): A systematic review and meta-analysis. Autoimmun Rev. 2019;18(11):102392. | Not included for SR |
| 418 Jalali MM, Soleimani R, Soltanipour S, et al. Pharmacological Treatments of Bell's Palsy in Adults: A Systematic Review and Network Meta-Analysis. Laryngoscope. 2021;131(7):1615-1625. | Not included for SR |
| 419 Jaruvongvanich V, Ahuja W, Sanguankeo A, et al. Vitamin D and histologic severity of nonalcoholic fatty liver disease: A systematic review and meta-analysis. Dig Liver Dis. 2017;49(6):618-622. | Not included for SR |
| 420 Kagoya Y, Nannya Y, Kurokawa M. Thalidomide maintenance therapy for patients with multiple myeloma: meta-analysis. Leuk Res. 2012;36(8):1016-1021. | Not included for SR |
| 421 Kassab M, Foster JP, Foureur M, et al. Sweet-tasting solutions for needle-related procedural pain in infants one month to one year of age. Cochrane Database Syst Rev. 2012;12(12):CD008411. | Not included for SR |
| 422 Khan A, Podlasek A, Somaa F. Virtual reality in post-stroke neurorehabilitation–a systematic review and meta-analysis. Topics in stroke rehabilitation, 2023, 30(1): 53-72. | Not included for SR |
| 423 Khosroshahi M Z, Asbaghi O, Moradi S, et al. The effects of supplementation with L-arginine on anthropometric indices and body composition in overweight or obese subjects: A systematic review and meta-analysis. Journal of Functional Foods, 2020, 71: 104022. | Not included for SR |
| 424 Kim HS, Jeong CW, Kwak C, et al. Pathological T0 Following Cisplatin-Based Neoadjuvant Chemotherapy for Muscle-Invasive Bladder Cancer: A Network Meta-analysis. Clin Cancer Res. 2016;22(5):1086-1094. | Not included for SR |
| 425 Kinoshita Y, Furukawa TA, Kinoshita K, et al. Supported employment for adults with severe mental illness. Cochrane Database Syst Rev. 2013(9):CD008297. | Not included for SR |
| 426 Kirkham AM, Bailey AJM, Shorr R, et al. Systematic review and meta-analysis of randomized controlled trials of mesenchymal stromal cells to treat coronavirus disease 2019: is it too late?. Cytotherapy. 2023;25(3):341-352. | Not included for SR |
| 427 Konnyu KJ, Yogasingam S, Lépine J, et al. Quality improvement strategies for diabetes care: Effects on outcomes for adults living with diabetes. Cochrane Database Syst Rev. 2023(5):CD014513. | Not included for SR |
| 428 Lau RW, Liao LR, Yu F, et al. The effects of whole body vibration therapy on bone mineral density and leg muscle strength in older adults: a systematic review and meta-analysis. Clin Rehabil. 2011;25(11):975-988. | Not included for SR |
| 429 Law LS, Lo EA, Gan TJ. Xenon Anesthesia: A Systematic Review and Meta-Analysis of Randomized Controlled Trials. Anesth Analg. 2016;122(3):678-697. | Not included for SR |
| 430 Li F, Guo L, Huang Z, et al. Effects of dexmedetomidine as an adjuvant to ropivacaine or ropivacaine alone on duration of postoperative analgesia: A systematic review and meta-analysis of randomized controlled trials. PLoS One. 2023;18(10):e0287296. | Not included for SR |
| 431 Li J, Song Y. Transcutaneous electrical nerve stimulation for postoperative pain control after total knee arthroplasty: A meta-analysis of randomized controlled trials. Medicine (Baltimore). 2017;96(37):e8036. | Not included for SR |

| | |
|---|---|
| 432 Li J, Ye Z, Hu X, et al. Prognostic, Diagnostic, and Clinicopathological Significance of Circular RNAs in Pancreatic Cancer: A Systematic Review and Meta-Analysis. Cancers (Basel). 2022;14(24):6187. | Not included for SR |
| 433 Li W, Zhang HH, Wang Y, et al. Poor Insight in Schizophrenia Patients in China: a Meta-Analysis of Observational Studies. Psychiatr Q. 2020;91(4):1017-1031. | Not included for SR |
| 434 Li X, Kou S, Chen G, et al. The relationship between vitamin D deficiency and diabetic foot ulcer: A meta-analysis. Int Wound J. 2023;20(8):3015-3022. | Not included for SR |
| 435 Liao L, Chen Y, Tang Q, et al. Tranexamic acid plus drain-clamping can reduce blood loss in total knee arthroplasty: A systematic review and meta-analysis. Int J Surg. 2018;52:334-341. | Not included for SR |
| 436 Liu L, Sun H, Nie F, et al. Prognostic Value of Abnormal Ankle-Brachial Index in Patients With Coronary Artery Disease: A Meta-Analysis. Angiology. 2020;71(6):491-497. | Not included for SR |
| 437 Liu W, Meng Z, Wang G. The Efficacy of Nitrates for Bone Health: A Systematic Review and Meta-Analysis of Observational and Randomized Controlled Studies. Front Endocrinol (Lausanne). 2022;13:833932. | Not included for SR |
| 438 Lőrincz A, Váradi A, Hegyi P, et al. Paediatric Partial-Thickness Burn Therapy: A Meta-Analysis and Systematic Review of Randomised Controlled Trials. Life (Basel). 2022;12(5):619. | Not included for SR |
| 439 Losurdo G, Leandro G, Principi M, et al. Sequential vs. prolonged 14-day triple therapy for Helicobacter pylori eradication: the meta-analysis may be influenced by 'geographical weighting'. Int J Clin Pract. 2015;69(10):1112-1120. | Not included for SR |
| 440 Loughrey DG, Lavecchia S, Brennan S, et al. The Impact of the Mediterranean Diet on the Cognitive Functioning of Healthy Older Adults: A Systematic Review and Meta-Analysis. Adv Nutr. 2017;8(4):571-586. | Not included for SR |
| 441 Lu Y, Zhou Z, Zhang X, Cui Y, Zhang Y, Wang Y. The Influence of Tramadol on Intravaginal Ejaculatory Latency Time and Sexual Satisfaction Score in Treating Patients With Premature Ejaculation: A Network Meta-Analysis. Am J Mens Health. 2021;15(6):15579883211057713. | Not included for SR |
| 442 Luo C, Zhang Y, Ding Y, et al. Nut consumption and risk of type 2 diabetes, cardiovascular disease, and all-cause mortality: a systematic review and meta-analysis. Am J Clin Nutr. 2014;100(1):256-269. | Not included for SR |
| 443 Luque-Fernandez MA, Bain PA, Gelaye B, et al. Sleep-disordered breathing and gestational diabetes mellitus: a meta-analysis of 9,795 participants enrolled in epidemiological observational studies. Diabetes Care. 2013;36(10):3353-3360. | Not included for SR |
| 444 Machado-Júnior AJ, Zancanella E, Crespo AN. Rapid maxillary expansion and obstructive sleep apnea: A review and meta-analysis. Med Oral Patol Oral Cir Bucal. 2016;21(4):e465-e469. | Not included for SR |
| 445 Maheshwari A, Pandey S, Shetty A, et al. Obstetric and perinatal outcomes in singleton pregnancies resulting from the transfer of frozen thawed versus fresh embryos generated through in vitro fertilization treatment: a systematic review and meta-analysis. Fertil Steril. 2012;98(2):368-77.e779. | Not included for SR |

| | |
|---|---|
| 446 Majure DT, Greco T, Greco M, et al. Meta-analysis of randomized trials of effect of milrinone on mortality in cardiac surgery: an update. J Cardiothorac Vasc Anesth. 2013;27(2):220-229. | Not included for SR |
| 447 Mandema JW, Zheng J, Libanati C, et al. Time course of bone mineral density changes with denosumab compared with other drugs in postmenopausal osteoporosis: a dose-response-based meta-analysis. J Clin Endocrinol Metab. 2014;99(10):3746-3755. | Not included for SR |
| 448 Marín-Cascales E, Alcaraz PE, Ramos-Campo DJ, et al. Whole-body vibration training and bone health in postmenopausal women: A systematic review and meta-analysis. Medicine (Baltimore). 2018;97(34):e11918. | Not included for SR |
| 449 Mariotti G, Salciccia S, Viscuso P, et al. Regenerative Medicine-Based Treatment of Stress Urinary Incontinence with Mesenchymal Stem Cells: A Systematic Review and Meta-analysis. Curr Stem Cell Res Ther. 2023;18(3):429-437. | Not included for SR |
| 450 Mattishent K, Thavarajah M, Sinha A, et al. Safety of 80% vs 30-35% fraction of inspired oxygen in patients undergoing surgery: a systematic review and meta-analysis. Br J Anaesth. 2019;122(3):311-324. | Not included for SR |
| 451 Mazidi M, Rezaie P, Ferns GA, et al. Impact of Probiotic Administration on Serum C-Reactive Protein Concentrations: Systematic Review and Meta-Analysis of Randomized Control Trials. Nutrients. 2017;9(1):20. | Not included for SR |
| 452 McDermott MS, Oliver M, Simnadis T, et al. The Theory of Planned Behaviour and dietary patterns: A systematic review and meta-analysis. Prev Med. 2015;81:150-156. | Not included for SR |
| 453 Meißner M, Austenfeld E, Kranke P, et al. Pectoral nerve blocks for breast surgery: A meta-analysis. Eur J Anaesthesiol. 2021;38(4):383-393. | Not included for SR |
| 454 Middleton P, Shepherd E, Gomersall JC. Venous thromboembolism prophylaxis for women at risk during pregnancy and the early postnatal period. Cochrane Database Syst Rev. 2021(3):CD001689. | Not included for SR |
| 455 Migliorini F, Maffulli N, Spiezia F, et al. Potential of biomarkers during pharmacological therapy setting for postmenopausal osteoporosis: a systematic review. J Orthop Surg Res. 2021;16(1):351. | Not included for SR |
| 456 Mikkonen K, Yamakawa M, Tomietto M, et al. Randomised controlled trials addressing how the clinical application of information and communication technology impacts the quality of patient care-A systematic review and meta-analysis. J Clin Nurs. 2023;32(13-14):3295-3314. | Not included for SR |
| 457 Mira TAA, Buen MM, Borges MG, et al. Systematic review and meta-analysis of complementary treatments for women with symptomatic endometriosis. Int J Gynaecol Obstet. 2018;143(1):2-9. | Not included for SR |
| 458 Mousavi SM, Beatriz Pizarro A, Akhgarjand C, et al. The effects of Anethum graveolens (dill) supplementation on lipid profile and glycemic control: a systematic review and meta-analysis of randomized controlled trials. Crit Rev Food Sci Nutr. 2022;62(21):5705-5716. | Not included for SR |
| 459 Neale EP, Tapsell LC, Guan V, et al. The effect of nut consumption on markers of inflammation and endothelial function: a systematic review and meta-analysis of randomised controlled trials. BMJ Open. 2017;7(11):e016863. | Not included for SR |

| | |
|---|---|
| 460 Nissensohn M, Román-Viñas B, Sánchez-Villegas A, et al. The Effect of the Mediterranean Diet on Hypertension: A Systematic Review and Meta-Analysis. J Nutr Educ Behav. 2016;48(1):42-53.e1. | Not included for SR |
| 461 O'Connor LE, Kim JE, Campbell WW. Total red meat intake of ≥0.5 servings/d does not negatively influence cardiovascular disease risk factors: a systemically searched meta-analysis of randomized controlled trials. Am J Clin Nutr. 2017;105(1):57-69. | Not included for SR |
| 462 Pannu PK, Zhao Y, Soares MJ. Reductions in body weight and percent fat mass increase the vitamin D status of obese subjects: a systematic review and metaregression analysis. Nutr Res. 2016;36(3):201-213. | Not included for SR |
| 463 Parvizian MK, Dhaliwal M, Li J, et al. Relationship between dietary patterns and COPD: a systematic review and meta-analysis. ERJ Open Res. 2020;6(2):00168-2019. | Not included for SR |
| 464 Peng YN, Sung FC, Huang ML, et al. The use of intravenous magnesium sulfate on postoperative analgesia in orthopedic surgery: A systematic review of randomized controlled trials. Medicine (Baltimore). 2018;97(50):e13583. | Not included for SR |
| 465 Potter J, Langhorne P, Roberts M. Routine protein energy supplementation in adults: systematic review. BMJ. 1998;317(7157):495-501. | Not included for SR |
| 466 Power J, French R, Cowan F. Subdermal implantable contraceptives versus other forms of reversible contraceptives or other implants as effective methods of preventing pregnancy. Cochrane Database Syst Rev. 2007;2007(3):CD001326. | Not included for SR |
| 467 Prin M, Guglielminotti J, Moitra V, et al. Prophylactic Ondansetron for the Prevention of Intrathecal Fentanyl- or Sufentanil-Mediated Pruritus: A Meta-Analysis of Randomized Trials . Anesth Analg. 2016;122(2):402-409. | Not included for SR |
| 468 Qi KJ, Zhao ZT, Zhang W, et al. The impacts of vitamin D supplementation in adults with metabolic syndrome: A systematic review and meta-analysis of randomized controlled trials. Front Pharmacol. 2022;13:1033026. | Not included for SR |
| 469 Radkhah N, Zarezadeh M, Jamilian P, et al. The Effect of Vitamin D Supplementation on Lipid Profiles: an Umbrella Review of Meta-Analyses. Adv Nutr. 2023;14(6):1479-1498. | Not included for SR |
| 470 Ragó Z, Tóth B, Szalenko-Tőkés Á, et al. Results of a systematic review and meta-analysis of early studies on ivermectin in SARS-CoV-2 infection. Geroscience. 2023;45(4):2179-2193. | Not included for SR |
| 471 Rahmani F, Saghazadeh A, Rahmani M, et al. Plasma levels of brain-derived neurotrophic factor in patients with Parkinson disease: A systematic review and meta-analysis. Brain Res. 2019;1704:127-136. | Not included for SR |
| 472 Ramli FF, Azizi MH, Syed Hashim SA. Treatments of Sexual Dysfunction in Opioid Substitution Therapy Patients: A Systematic Review and Meta-Analysis. Int J Med Sci. 2021;18(11):2372-2380. | Not included for SR |
| 473 Redzic M, Lewis RM, Thomas DT. Relationship between 25-hydoxyvitamin D, muscle strength, and incidence of injury in healthy adults: a systematic review. Nutr Res. 2013;33(4):251-258. | Not included for SR |
| 474 Rigo J, Pieltain C, Christmann V, et al. Serum Magnesium Levels in Preterm Infants Are Higher Than Adult Levels: A Systematic Literature Review and Meta-Analysis. Nutrients. 2017;9(10):1125. | Not included for SR |

| | |
|---|---|
| 475 Rohwer AC, Oladapo OT, Hofmeyr GJ. Strategies for optimising antenatal corticosteroid administration for women with anticipated preterm birth. Cochrane Database Syst Rev. 2020;5(5):CD013633. | Not included for SR |
| 476 Rømsing J, Møiniche S, Mathiesen O, et al. Reduction of opioid-related adverse events using opioid-sparing analgesia with COX-2 inhibitors lacks documentation: a systematic review. Acta Anaesthesiol Scand. 2005;49(2):133-142. | Not included for SR |
| 477 Rosendahl-Riise H, Spielau U, Ranhoff AH, et al. Vitamin D supplementation and its influence on muscle strength and mobility in community-dwelling older persons: a systematic review and meta-analysis . in J Hum Nutr Diet. 2018;31(6):825-826. | Not included for SR |
| 478 Sanders TA. Protective effects of dietary PUFA against chronic disease: evidence from epidemiological studies and intervention trials. Proc Nutr Soc. 2014;73(1):73-79. | Not included for SR |
| 479 Sargsyan N, Das B, Robb H, et al. Outcomes of One-Anastomosis Gastric Bypass Conversion to Roux-en-Y Gastric Bypass for Severe Obesity: A Systematic Review and Meta-analysis. Obes Surg. 2024;34(3):976-984. | Not included for SR |
| 480 Sar-Shalom Nahshon C, Sagi-Dain L, Wiener-Megnazi Z, et al. The impact of intentional endometrial injury on reproductive outcomes: a systematic review and meta-analysis. Hum Reprod Update. 2019;25(1):95-113. | Not included for SR |
| 481 Saz-Lara A, Cavero-Redondo I, Martínez-Vizcaíno V, Martínez-Ortega IA, Notario-Pacheco B, Pascual-Morena C. The Comparative Effects of Different Types of Oral Vitamin Supplements on Arterial Stiffness: A Network Meta-Analysis. Nutrients. 2022;14(5):1009. | Not included for SR |
| 482 Schnabel A, Reichl SU, Weibel S, et al. Efficacy and safety of dexmedetomidine in peripheral nerve blocks: A meta-analysis and trial sequential analysis. Eur J Anaesthesiol. 2018;35(10):745-758. | Not included for SR |
| 483 Seitz DP, Adunuri N, Gill SS, et al. Prevalence of dementia and cognitive impairment among older adults with hip fractures. J Am Med Dir Assoc. 2011;12(8):556-564. | Not included for SR |
| 484 Shao C, Tang H, Zhao W, et al. Nut intake and stroke risk: A dose-response meta-analysis of prospective cohort studies. Sci Rep. 2016;6:30394. | Not included for SR |
| 485 Sharma AP, Sharma G, Tyagi S, et al. Safety and efficacy of "on-demand" tramadol in patients with premature ejaculation: an updated meta-analysis. Int Braz J Urol. 2021;47(5):921-934. | Not included for SR |
| 486 Shi H, Santos HO, de Souza IGO, et al. The Effect of Raloxifene Treatment on Lipid Profile in Elderly Individuals: A Systematic Review and Meta-analysis of Randomized Clinical Trials. Clin Ther. 2021;43(9):297-317. | Not included for SR |
| 487 Shi W, Ghisi GLM, Zhang L, et al. Systematic review, meta-analysis and meta-regression to determine the effects of patient education on health behaviour change in adults diagnosed with coronary heart disease. J Clin Nurs. 2023;32(15-16):5300-5327. | Not included for SR |
| 488 Słabuszewska-Jóźwiak A, Szymański JK, Ciebiera M, et al. Pediatrics Consequences of Caesarean Section-A Systematic Review and Meta-Analysis. Int J Environ Res Public Health. 2020;17(21):8031. | Not included for SR |
| 489 So WWY, Lu EY, Cheung WM, Tsang HWH. Comparing Mindful and Non-Mindful Exercises on Alleviating Anxiety Symptoms: A Systematic Review and Meta-Analysis. Int J Environ Res Public Health. 2020;17(22):8692. | Included but not synthesized/presented |

| | |
|---|---|
| 490 Soltani S, Hunter GR, Kazemi A, et al. The effects of weight loss approaches on bone mineral density in adults: a systematic review and meta-analysis of randomized controlled trials. Osteoporos Int. 2016;27(9):2655-2671. | Not included for SR |
| 491 Song L, Luo X, Jiang Q, et al. Vitamin D Supplementation is Beneficial for Children with Autism Spectrum Disorder: A Meta-analysis. Clin Psychopharmacol Neurosci. 2020;18(2):203-213. | Not included for SR |
| 492 Spillman LN, Madden AM, Richardson H, et al. Nutritional Intake after Liver Transplant: Systematic Review and Meta-Analysis. Nutrients. 2023;15(11):2487. | Not included for SR |
| 493 Tabrizi R, Moosazadeh M, Lankarani KB, et al. The Effects of Synbiotic Supplementation on Glucose Metabolism and Lipid Profiles in Patients with Diabetes: a Systematic Review and Meta-Analysis of Randomized Controlled Trials. Probiotics Antimicrob Proteins. 2018;10(2):329-342. | Not included for SR |
| 494 Talebi S, Miraghajani M, Hosseini R, et al. The Effect of Oral Magnesium Supplementation on Inflammatory Biomarkers in Adults: A Comprehensive Systematic Review and Dose-response Meta-analysis of Randomized Clinical Trials. Biol Trace Elem Res. 2022;200(4):1538-1550. | Not included for SR |
| 495 Tian DH, Weller J, Hasmat S, et al. Temperature Selection in Antegrade Cerebral Perfusion for Aortic Arch Surgery: A Meta-Analysis. Ann Thorac Surg. 2019;108(1):283-291. | Not included for SR |
| 496 Tiong L, Maddern GJ. Systematic review and meta-analysis of survival and disease recurrence after radiofrequency ablation for hepatocellular carcinoma. Br J Surg. 2011;98(9):1210-1224. | Not included for SR |
| 497 Toner AJ, Ganeshanathan V, Chan MT, et al. Safety of Perioperative Glucocorticoids in Elective Noncardiac Surgery: A Systematic Review and Meta-analysis. Anesthesiology. 2017;126(2):234-248. | Not included for SR |
| 498 Uhe I, Douissard J, Podetta M, et al. Roux-en-Y gastric bypass, sleeve gastrectomy, or one-anastomosis gastric bypass? A systematic review and meta-analysis of randomized-controlled trials. Obesity (Silver Spring). 2022;30(3):614-627. | Not included for SR |
| 499 Uhlig A, Uhlig J, Trojan L, et al. Surgical approaches for treatment of ureteropelvic junction obstruction - a systematic review and network meta-analysis. BMC Urol. 2019;19(1):112. | Not included for SR |
| 500 van den Brandt PA, Schouten LJ. Relationship of tree nut, peanut and peanut butter intake with total and cause-specific mortality: a cohort study and meta-analysis. Int J Epidemiol. 2015;44(3):1038-1049. | Not included for SR |
| 501 van Wijngaarden JP, Doets EL, Szczecińska A, et al. Vitamin B12, folate, homocysteine, and bone health in adults and elderly people: a systematic review with meta-analyses. J Nutr Metab. 2013;2013:486186. | Not included for SR |
| 502 Velotti N, Vitiello A, Berardi G, et al. Roux-en-Y gastric bypass versus one anastomosis-mini gastric bypass as a rescue procedure following failed restrictive bariatric surgery. A systematic review of literature with metanalysis. Updates Surg. 2021;73(2):639-647. | Not included for SR |
| 503 Vemulapalli S, Dolor RJ, Hasselblad V, et al. Supervised vs unsupervised exercise for intermittent claudication: A systematic review and meta-analysis. Am Heart J. 2015;169(6):924-937.e3. | Not included for SR |

| | |
|---|---|
| 504 Venetis CA, Kolibianakis EM, Bosdou JK, et al. Progesterone elevation and probability of pregnancy after IVF: a systematic review and meta-analysis of over 60 000 cycles. Hum Reprod Update. 2013;19(5):433-457. | Not included for SR |
| 505 Volpe M. TRANSCEND Aftermath: Angiotensin II Type 1 Receptor Antagonists (Angiotensin Receptor Blockers) at the Clinical Crossroad. High Blood Pressure & Cardiovascular Prevention, 2009, 16: 1-6. | Not included for SR |
| 506 Wang D, Chen C, Chen J, et al. The use of propofol as a sedative agent in gastrointestinal endoscopy: a meta-analysis. PLoS One. 2013;8(1):e53311. | Not included for SR |
| 507 Wang R, Xu F, Xia X, et al. The effect of vitamin D supplementation on primary depression: A meta-analysis. J Affect Disord. 2024;344:653-661. | Not included for SR |
| 508 Wetterslev J, Meyhoff CS, Jørgensen LN, et al. The effects of high perioperative inspiratory oxygen fraction for adult surgical patients. Cochrane Database Syst Rev. 2015(6):CD008884. | Not included for SR |
| 509 Wilkes MM, Navickis RJ. Patient survival after human albumin administration. A meta-analysis of randomized, controlled trials. Ann Intern Med. 2001;135(3):149-164. | Included but not synthesized/presented |
| 510 Wu S, Ding Y, Wu F, et al. Omega-3 fatty acids intake and risks of dementia and Alzheimer's disease: a meta-analysis. Neurosci Biobehav Rev. 2015;48:1-9. | Not included for SR |
| 511 Wu Z, Zhang H, Jin W, et al. The Effect of Renin-Angiotensin-Aldosterone System Blockade Medications on Contrast-Induced Nephropathy in Patients Undergoing Coronary Angiography: A Meta-Analysis. PLoS One. 2015;10(6):e0129747. | Not included for SR |
| 512 Xia JY, Yu JH, Xu DF, Yang, et al. The Effects of Peanuts and Tree Nuts on Lipid Profile in Type 2 Diabetic Patients: A Systematic Review and Meta-Analysis of Randomized, Controlled-Feeding Clinical Studies. Front Nutr. 2021;8:765571. | Not included for SR |
| 513 Xiang W, Jiang L, Shi L, et al. The effect of magnesium added to bupivacaine for arthroscopy: a meta-analysis of randomized controlled trials. J Orthop Surg Res. 2021;16(1):583. | Not included for SR |
| 514 Xu CP, Li X, Wang ZZ, et al. Efficacy and safety of single-dose local infiltration of analgesia in total knee arthroplasty: a meta-analysis of randomized controlled trials. Knee. 2014;21(3):636-646. | Not included for SR |
| 515 Xu S, Wan Y, Xu M, et al. The association between obstructive sleep apnea and metabolic syndrome: a systematic review and meta-analysis. BMC Pulm Med. 2015;15:105. | Not included for SR |
| 516 Yang CC, Chou YC, Kuo TN, et al. Prophylactic Intraoperative Uterine Artery Embolization During Cesarean Section or Cesarean Hysterectomy in Patients with Abnormal Placentation: A Systematic Review and Meta-Analysis. Cardiovasc Intervent Radiol. 2022;45(4):488-501. | Not included for SR |
| 517 Yang G, Huang W, Xie W, et al. Patellar non-eversion in primary TKA reduces the complication rate. Knee Surg Sports Traumatol Arthrosc. 2016;24(3):921-930. | Not included for SR |
| 518 Yang G, Zheng B, Yu Y. Risk Assessment of Intermittent and Continuous Nasogastric Enteral Feeding Methods in Adult Inpatients: A Meta-Analysis. Evid Based Complement Alternat Med. 2021;2021:8875002. | Not included for SR |

| | |
|---|---|
| 519 Yang Q, Zhang Z, Xin W, et al. Preoperative intravenous glucocorticoids can decrease acute pain and postoperative nausea and vomiting after total hip arthroplasty: A PRISMA-compliant meta-analysis. Medicine (Baltimore). 2017;96(47):e8804. | Not included for SR |
| 520 Yang Z, Shen X, Zhou C, et al. Prevention of recurrent miscarriage in women with antiphospholipid syndrome: A systematic review and network meta-analysis. Lupus. 2021;30(1):70-79. | Not included for SR |
| 521 Yin WW, Huang CC, Chen YR, et al. The effect of medication on serum anti-müllerian hormone (AMH) levels in women of reproductive age: a meta-analysis. BMC Endocr Disord. 2022;22(1):158. | Not included for SR |
| 522 Zaric D, Pace NL. Transient neurologic symptoms (TNS) following spinal anaesthesia with lidocaine versus other local anaesthetics. Cochrane Database Syst Rev. 2009(2):CD003006. | Not included for SR |
| 523 Zhang C, Guo Y, Li J, et al. The role of the phosphatase and tensin homolog status in predicting pathological complete response to neoadjuvant anti-HER2 therapies in HER2-positive primary breast cancer: A meta-analysis. Medicine (Baltimore). 2019;98(5):e14261. | Not included for SR |
| 524 Zhang H, Zhu XH, Dong W, et al. Vitamin D Status and Patient Outcomes after Knee or Hip Surgery: A Meta-Analysis. Ann Nutr Metab. 2018;73(2):121-130. | Not included for SR |
| 525 Zhang L, Xie Y, Huang D, et al. LigaSure hemorrhoidectomy versus the procedure for prolapse and hemorrhoids: A meta-analysis of randomized controlled trials. Medicine (Baltimore). 2022;101(3):e28514. | Not included for SR |
| 526 Zhang MX, Pan GT, Guo JF, et al. Vitamin D Deficiency Increases the Risk of Gestational Diabetes Mellitus: A Meta-Analysis of Observational Studies. Nutrients. 2015;7(10):8366-8375. | Not included for SR |
| 527 Zhang Y, Gong Y, Xue H, et al. Vitamin D and gestational diabetes mellitus: a systematic review based on data free of Hawthorne effect. BJOG. 2018;125(7):784-793. | Not included for SR |
| 528 Zhao J, Shi X, Wang T, et al. The Prognostic and Clinicopathological Significance of IGF-1R in NSCLC: a Meta-Analysis. Cell Physiol Biochem. 2017;43(2):697-704. | Not included for SR |
| 529 Zhao Y, Shen L, Ji H F. Osteoporosis risk and bone mineral density levels in patients with Parkinson's disease: a meta-analysis. Bone, 2013, 52(1): 498-505. | Not included for SR |
| 530 Zheng G, Liu F, Li S, et al. Tai Chi and the Protection of Cognitive Ability: A Systematic Review of Prospective Studies in Healthy Adults. Am J Prev Med. 2015;49(1):89-97. | Not included for SR |
| 531 Zhou D, Yu H, He F, et al. Nut consumption in relation to cardiovascular disease risk and type 2 diabetes: a systematic review and meta-analysis of prospective studies. Am J Clin Nutr. 2014;100(1):270-277. | Not included for SR |
| 532 Zhou Z, Zhou R, Zhang Z, et al. The Association Between Vitamin D Status, Vitamin D Supplementation, Sunlight Exposure, and Parkinson's Disease: A Systematic Review and Meta-Analysis. Med Sci Monit. 2019;25:666-674. | Not included for SR |
| 533 Zou Z, Yuan HB, Yang B, et al. Perioperative angiotensin-converting enzyme inhibitors or angiotensin II type 1 receptor blockers for preventing mortality and morbidity in adults. Cochrane Database Syst Rev. 2016(1):CD009210. | Not included for SR |

| | |
|---|---|
| 534 Zou ZY, He LX, Yao YT; Evidence in Cardiovascular Anesthesia (EICA) Group. The effects of tranexamic acid on platelets in patients undergoing cardiac surgery: a systematic review and meta-analysis. J Thromb Thrombolysis. 2024;57(2):235-247. | Not included for SR |
| 535 Bui BN, Lensen SF, Gibreel A,et al. Endometrial injury for pregnancy following sexual intercourse or intrauterine insemination. Cochrane Database Syst Rev. 2021(3):CD011424. | Included but not synthesized/presented |
| 536 Dexter F, Bayman EO, Epstein RH. Statistical modeling of average and variability of time to extubation for meta-analysis comparing desflurane to sevoflurane. Anesth Analg. 2010;110(2):570-580. | Not report study-level data |
| 537 Fergusson D, Glass KC, Hutton B, et al. Randomized controlled trials of aprotinin in cardiac surgery: could clinical equipoise have stopped the bleeding?. Clin Trials. 2005;2(3):218-232. | Included but not synthesized/presented |
| 538 Kaufmann T, Clement RP, Scheeren TWL, et al. Perioperative goal-directed therapy: A systematic review without meta-analysis. Acta Anaesthesiol Scand. 2018;62(10):1340-1355. | Included but not synthesized/presented |
| 539 Maheshwari A, Pandey S, Amalraj Raja E,et al. Is frozen embryo transfer better for mothers and babies? Can cumulative meta-analysis provide a definitive answer?. Hum Reprod Update. 2018;24(1):35-58. | Cummulative meta-analysis |
| 540 Mishu MP, Uphoff E, Aslam F, et al. Interventions for preventing type 2 diabetes in adults with mental disorders in low- and middle-income countries. Cochrane Database Syst Rev. 2021(2):CD013281. | Included but not synthesized/presented |
| 541 Yu Y, Fang L, Zhang R, et al. Comparative effectiveness of 9 ovulation-induction therapies in patients with clomiphene citrate-resistant polycystic ovary syndrome: a network meta-analysis. Sci Rep. 2017;7(1):3812. | Not report study-level data |
| 542 Endesfelder D, McGranahan N, Birkbak NJ, et al. A breast cancer meta-analysis of two expression measures of chromosomal instability reveals a relationship with younger age at diagnosis and high risk histopathological variables. Oncotarget. 2011;2(7):529-537. | Not report study-level data |
| 543 Gaudino M, Sandner S, An KR, et al. Graft Failure After Coronary Artery Bypass Grafting and Its Association With Patient Characteristics and Clinical Events: A Pooled Individual Patient Data Analysis of Clinical Trials With Imaging Follow-Up. Circulation. 2023;148(17):1305-1315. | Not report study-level data |
| 544 Guo J, Jin X, Wang H, et al. Emergence and Recovery Characteristics of Five Common Anesthetics in Pediatric Anesthesia: a Network Meta-analysis. Mol Neurobiol. 2017;54(6):4353-4364. | Not report study-level data |
| 545 Mo L, Jiang B, Mei T, et al. Exercise Therapy for Knee Osteoarthritis: A Systematic Review and Network Meta-analysis. Orthop J Sports Med. 2023;11(5):23259671231172773. | Not report study-level data |
| 546 Plock N, Bax L, Lee D, et al. Exploratory Literature Meta-Analysis to Characterize the Relationship Between Early and Longer Term Body Weight Loss for Antiobesity Compounds. J Clin Pharmacol. 2017;57(1):52-63. | Not report study-level data |
| 547 Qu HC, Huang Y, Mu ZY, et al. Efficacy and Safety of Chemotherapy Regimens in Advanced or Metastatic Bladder and Urothelial Carcinomas: An Updated Network Meta-Analysis. Front Pharmacol. 2020;10:1507. | Not report study-level data |

| | |
|---|---|
| 548 Ren R, Bao S, Qian W,et al. Efficacy and Safety of Picosecond Laser in the Treatment of Melasma: A Network Meta-analysis. Dermatol Surg. 2023;49(5S):S49-S55. | Not report study-level data |
| 549 Safabakhsh M, Imani H, Shahinfar H, et al. Efficacy of dietary supplements on mortality and clinical outcomes in adults with sepsis and septic shock: A systematic review and network meta-analysis. Clin Nutr. 2024;43(6):1299-1307. | Not report study-level data |
| 550 Agoliati A, Dexter F, Lok J, et al. Meta-analysis of average and variability of time to extubation comparing isoflurane with desflurane or isoflurane with sevoflurane. Anesth Analg. 2010;110(5):1433-1439. | Not report study-level data |
| 551 Al-Ejeh F, Simpson PT, Saunus JM, et al. Meta-analysis of the global gene expression profile of triple-negative breast cancer identifies genes for the prognostication and treatment of aggressive breast cancer. Oncogenesis. 2014;3(10):e124. | Not report study-level data |
| 552 Bothe W, Olschewski M, Beyersdorf F,et al. Glucose-insulin-potassium in cardiac surgery: a meta-analysis. Ann Thorac Surg. 2004;78(5):1650-1657. | Not report study-level data |
| 553 Clayton AH, Hwang E, Kornstein SG, et al. Effects of 50 and 100 mg desvenlafaxine versus placebo on sexual function in patients with major depressive disorder: a meta-analysis. Int Clin Psychopharmacol. 2015;30(6):307-315. | Not report study-level data |
| 554 Di Stefano L, Ogburn EL, Ram M, et al. Hydroxychloroquine/chloroquine for the treatment of hospitalized patients with COVID-19: An individual participant data meta-analysis. PLoS One. 2022;17(9):e0273526. | Not report study-level data |
| 555 EPPPIC Group. Evaluating Progestogens for Preventing Preterm birth International Collaborative (EPPPIC): meta-analysis of individual participant data from randomised controlled trials.Lancet. 2021;397(10283):1446. | Not report study-level data |
| 556 Erford B T, Gunther C, Duncan K, et al. Meta-analysis of counseling outcomes for the treatment of posttraumatic stress disorder. Journal of Counseling & Development, 2016, 94(1): 13-30. | Not report study-level data |
| 557 Henzi I, Sonderegger J, Tramèr MR. Efficacy, dose-response, and adverse effects of droperidol for prevention of postoperative nausea and vomiting. Can J Anaesth. 2000;47(6):537-551. | Not report study-level data |
| 558 Hurley JC. Incidence of coagulase-negative staphylococcal bacteremia among ICU patients: decontamination studies as a natural experiment. Eur J Clin Microbiol Infect Dis. 2020;39(4):657-664. | Not report study-level data |
| 559 Jin S, Sha L, Dong J, et al. Effects of Nutritional Strategies on Glucose Homeostasis in Gestational Diabetes Mellitus: A Systematic Review and Network Meta-Analysis. J Diabetes Res. 2020;2020:6062478. | Not report study-level data |
| 560 Jin S, Sha L, Dong J, et al. Effects of Nutritional Strategies on Glucose Homeostasis in Gestational Diabetes Mellitus: A Systematic Review and Network Meta-Analysis. J Diabetes Res. 2020;2020:6062478. | Not report study-level data |
| 561 Kovac AL. Meta-analysis of the use of rescue antiemetics following PONV prophylactic failure with 5-HT3 antagonist/dexamethasone versus single-agent therapies. Ann Pharmacother. 2006;40(5):873-887. | Not report study-level data |
| 562 Macario A, Dexter F, Lubarsky D. Meta-analysis of trials comparing postoperative recovery after anesthesia with sevoflurane or desflurane. Am J Health Syst Pharm. 2005;62(1):63-68. | Not report study-level data |

| | |
|---|---|
| 563 Machado A, Quadflieg K, Oliveira A, et al. Exercise Training in Patients with Chronic Respiratory Diseases: Are Cardiovascular Comorbidities and Outcomes Taken into Account?-A Systematic Review. J Clin Med. 2019;8(9):1458. | Not report study-level data |
| 564 Osborn DA, Sinn JK, Jones LJ. Infant formulas containing hydrolysed protein for prevention of allergic disease and food allergy. Cochrane Database Syst Rev. 2017(3):CD003664. | Not report study-level data |
| 565 Osborn DA, Sinn JK, Jones LJ. Infant formulas containing hydrolysed protein for prevention of allergic disease. Cochrane Database Syst Rev. 2018(10):CD003664. | Not report study-level data |
| 566 Patki A. Laryngeal mask airway vs the endotracheal tube in paediatric airway management: A meta-analysis of prospective randomised controlled trials. Indian J Anaesth. 2011;55(5):537-541. | Not report study-level data |
| 567 Søreide K, Thorsen K, Søreide JA. Strategies to improve the outcome of emergency surgery for perforated peptic ulcer. Br J Surg. 2014;101(1):e51-e64. | Not report study-level data |
| 568 Thomopoulos C, Parati G, Zanchetti A. Effects of blood pressure-lowering on outcome incidence in hypertension: 5. Head-to-head comparisons of various classes of antihypertensive drugs - overview and meta-analyses. J Hypertens. 2015;33(7):1321-1341. | Not report study-level data |
| 569 Wiedermann CJ. Systematic review of randomized clinical trials on the use of hydroxyethyl starch for fluid management in sepsis. BMC Emerg Med. 2008;8:1. | Not report study-level data |
| 570 Behera BK, Misra S, Jena SS, et al. The effect of perioperative dexmedetomidine on postoperative bowel function recovery in adult patients receiving general anesthesia. Minerva Anestesiol. 2022;88(1-2):51-61. | No full-text |
| 571 Chan KY, Wong MMH, Pang SSH, Lo KKH. Dietary supplementation for gestational diabetes prevention and management: a meta-analysis of randomized controlled trials. Arch Gynecol Obstet. 2021;303(6):1381-1391. | Included but not synthesized/presented |
| 572 Himpe D. Colloids versus crystalloids as priming solutions for cardiopulmonary bypass: a meta-analysis of prospective, randomised clinical trials. Acta Anaesthesiol Belg. 2003;54(3):207-215. | No full-text |
| 573 Huang Q, Li K, Li M, Xu G. Comparisons of Three Main Treatments on Renoprotective Effects in Diabetes Mellitus. Iran J Kidney Dis. 2019;13(1):36-47. | No full-text |
| 574 Nowbar AN, Mielewczik M, Karavassilis M, et al. Discrepancies in autologous bone marrow stem cell trials and enhancement of ejection fraction (DAMASCENE): weighted regression and meta-analysis. BMJ. 2014;348:g2688. | Included but not synthesized/presented |
| 575 Patki A. Laryngeal mask airway vs the endotracheal tube in paediatric airway management: A meta-analysis of prospective randomised controlled trials. Indian Journal of Anaesthesia, 2011, 55(5): 537-541. | No full-text |
| 576 Qiang H, Hang L, Shui SY. The curative effect of early use of enteral immunonutrition in postoperative gastric cancer: a meta-analysis. Minerva Gastroenterol Dietol. 2017;63(3):285-292. | No full-text |
| 577 Takagi H, Umemoto T. A contemporary meta-analysis of the association of diabetes with abdominal aortic aneurysm. Int Angiol. 2015;34(4):375-382. | No full-text |

| | |
|---|---|
| 578 Xiao-Hui T, Lu-Mei H U, Xiang-Long Y I, et al. Meta-analysis of the efficacy and safety of 0.05% cyclosporine A in the treatment of dry eye. International Eye Science, 2023: 248-255. | No full-text |
| 579 Gabbai-Armelin PR, Sales LS, Ferrisse TM, et al. A systematic review and meta-analysis of the effect of thymol as an anti-inflammatory and wound healing agent: A review of thymol effect on inflammation and wound healing: A review of thymol effect on inflammation and wound healing. Phytother Res. 2022;36(9):3415-3443. | Not report study-level data |
| 580 Gupta A, Bodin L, Holmström B, Berggren L. A systematic review of the peripheral analgesic effects of intraarticular morphine. Anesth Analg. 2001;93(3):761-770. | Not report study-level data |
| 581 Hartog CS, Kohl M, Reinhart K. A systematic review of third-generation hydroxyethyl starch (HES 130/0.4) in resuscitation: safety not adequately addressed. Anesth Analg. 2011;112(3):635-645. | Not report study-level data |
| 582 Jiang G, Xu X, Ren S, et al. Combining transarterial chemoembolization with radiofrequency ablation for hepatocellular carcinoma. Tumour Biol. 2014;35(4):3405-3408. | Not report study-level data |
| 583 Perel P, Roberts I, Ker K. Colloids versus crystalloids for fluid resuscitation in critically ill patients. Cochrane Database Syst Rev. 2013(2):CD000567. | Not report study-level data |
| 584 Abdelgadir IS, Gordon MA, Akobeng AK. Melatonin for the management of sleep problems in children with neurodevelopmental disorders: a systematic review and meta-analysis. Arch Dis Child. 2018;103(12):1155-1162. | Included but not synthesized/presented |
| 585 Alqalyoobi S, Boctor N, Sarkeshik AA, et al. Therapeutic hypothermia and mortality in the intensive care unit: systematic review and meta-analysis. Crit Care Resusc. 2019;21(4):287-298. | Included but not synthesized/presented |
| 586 Bar-Yoseph H, Hussein K, Braun E, et al. Natural history and decolonization strategies for ESBL/carbapenem-resistant Enterobacteriaceae carriage: systematic review and meta-analysis. J Antimicrob Chemother. 2016;71(10):2729-2739. | Included but not synthesized/presented |
| 587 Berger, A. Cochrane Injuries Group Albumin ReviewersWhy albumin may not work. Bmj, 317(7153), 235-240. | Included but not synthesized/presented |
| 588 Bloomfield HE, Koeller E, Greer N, et al. Effects on Health Outcomes of a Mediterranean Diet With No Restriction on Fat Intake: A Systematic Review and Meta-analysis. Ann Intern Med. 2016;165(7):491-500. | Included but not synthesized/presented |
| 589 Christofi MD, Giannakou K, Mpouzika M, et al. The effectiveness of oral bovine lactoferrin compared to iron supplementation in patients with a low hemoglobin profile: A systematic review and meta-analysis of randomized clinical trials. BMC Nutr. 2024;10(1):20. | Included but not synthesized/presented |
| 590 Contardo Ayala AM, Parker K, Mazzoli E, et al. Effectiveness of Intervention Strategies to Increase Adolescents' Physical Activity and Reduce Sedentary Time in Secondary School Settings, Including Factors Related to Implementation: A Systematic Review and Meta-Analysis. Sports Med Open. 2024;10(1):25. | Included but not synthesized/presented |
| 591 Dahmani S, Brasher C, Stany I, et al. Premedication with clonidine is superior to benzodiazepines. A meta analysis of published studies. Acta Anaesthesiol Scand. 2010;54(4):397-402. | Included but not synthesized/presented |

| | |
|---|---|
| 592 Demiri M, Antunes T, Fletcher D, et al. Perioperative adverse events attributed to α2-adrenoceptor agonists in patients not at risk of cardiovascular events: systematic review and meta-analysis. Br J Anaesth. 2019;123(6):795-807. | Included but not synthesized/presented |
| 593 Dewansingh P, Melse-Boonstra A, Krijnen WP, et al. Supplemental protein from dairy products increases body weight and vitamin D improves physical performance in older adults: a systematic review and meta-analysis. Nutr Res. 2018;49:1-22. | Included but not synthesized/presented |
| 594 Dissanayake HA, de Silva NL, Sumanatilleke M, et al. Prognostic and Therapeutic Role of Vitamin D in COVID-19: Systematic Review and Meta-analysis. J Clin Endocrinol Metab. 2022;107(5):1484-1502. | Included but not synthesized/presented |
| 595 Enthoven WT, Roelofs PD, Deyo RA, et al. Non-steroidal anti-inflammatory drugs for chronic low back pain. Cochrane Database Syst Rev. 2016;2(2):CD012087. | Included but not synthesized/presented |
| 596 Fan Y, Zhang AM, Xiao YB, et al. Glucose-insulin-potassium therapy in adult patients undergoing cardiac surgery: a meta-analysis. Eur J Cardiothorac Surg. 2011;40(1):192-199. | Included but not synthesized/presented |
| 597 Farah D, Fonseca MCM. Short-term Evidence in Adults of Anorexigenic Drugs Acting in the Central Nervous System: A Meta-Analysis. Clin Ther. 2019;41(9):1798-1815. | Included but not synthesized/presented |
| 598 Ford AA, Rogerson L, Cody JD, et al. Mid-urethral sling operations for stress urinary incontinence in women. Cochrane Database Syst Rev. 2017(7):CD006375. | Included but not synthesized/presented |
| 599 Gao C, Li B, Xu L, et al. Efficacy and safety of ramosetron versus ondansetron for postoperative nausea and vomiting after general anesthesia: a meta-analysis of randomized clinical trials. Drug Des Devel Ther. 2015;9:2343-2350. | Included but not synthesized/presented |
| 600 Greco M, Landoni G, Biondi-Zoccai G, et al. Remifentanil in cardiac surgery: a meta-analysis of randomized controlled trials. J Cardiothorac Vasc Anesth. 2012;26(1):110-116. | Included but not synthesized/presented |
| 601 Guo H, Lu Q. Efficacy of dydrogesterone on treating recurrent miscarriage and its influence on immune factors: a systematic review and meta-analysis. Ann Palliat Med. 2021;10(10):10971-10985. | Included but not synthesized/presented |
| 602 He C, Xia P, Xu J, et al. Evaluation of the efficacy of atorvastatin in the treatment for chronic subdural hematoma: a meta-analysis. Neurosurg Rev. 2021;44(1):479-484. | Included but not synthesized/presented |
| 603 He T, Liu X, Li Y, et al. High-dose calcium channel blocker (CCB) monotherapy vs combination therapy of standard-dose CCBs and angiotensin receptor blockers for hypertension: a meta-analysis. J Hum Hypertens. 2017;31(2):79-88. | Included but not synthesized/presented |
| 604 Ibañez-Perez J, Santos-Zorrozua B, Lopez-Lopez E, et al. An update on the implication of physical activity on semen quality: a systematic review and meta-analysis. Arch Gynecol Obstet. 2019;299(4):901-921. | Included but not synthesized/presented |
| 605 Jespersen KV, Koenig J, Jennum P, et al. Music for insomnia in adults. Cochrane Database Syst Rev. 2015(8):CD010459. | Included but not synthesized/presented |
| 606 Leung LYL, Tam HL, Ho JKM. Effectiveness of Tai Chi on older adults: A systematic review of systematic reviews with re-meta-analysis. Arch Gerontol Geriatr. 2022;103:104796. | Included but not synthesized/presented |

| | |
|---|---|
| 607 Li C, Ni Q, Pei Y, et al. Meta-analysis of the efficacy and safety of structured triglyceride lipid emulsions in parenteral nutrition therapy in China. Clin Nutr. 2019;38(4):1524-1535. | Included but not synthesized/presented |
| 608 Li YT, Cai HF, Zhang ZL. Timing of the initiation of bisphosphonates after surgery for fracture healing: a systematic review and meta-analysis of randomized controlled trials. Osteoporos Int. 2015;26(2):431-441. | Included but not synthesized/presented |
| 609 Luo X, Zhang J, Zhang C, et al. The effect of whole-body vibration therapy on bone metabolism, motor function, and anthropometric parameters in women with postmenopausal osteoporosis. Disabil Rehabil. 2017;39(22):2315-2323. | Included but not synthesized/presented |
| 610 MacKinnon M, Shurraw S, Akbari A, et al. Combination therapy with an angiotensin receptor blocker and an ACE inhibitor in proteinuric renal disease: a systematic review of the efficacy and safety data. Am J Kidney Dis. 2006;48(1):8-20. | Included but not synthesized/presented |
| 611 Mahoney CB. Platelet-rich plasmapheresis: a meta-analysis of clinical outcomes and costs. J Extra Corpor Technol. 1998;30(1):10-19. | Included but not synthesized/presented |
| 612 Martin Z, Spry G, Hoult J, et al. What is the efficacy of dietary, nutraceutical, and probiotic interventions for the management of gastroesophageal reflux disease symptoms? A systematic literature review and meta-analysis. Clin Nutr ESPEN. 2022;52:340-352. | Included but not synthesized/presented |
| 613 Messina A, Pelaia C, Bruni A, et al. Fluid Challenge During Anesthesia: A Systematic Review and Meta-analysis. Anesth Analg. 2018;127(6):1353-1364. | Included but not synthesized/presented |
| 614 Navaneethan SD, Nigwekar SU, Sehgal AR, et al. Aldosterone antagonists for preventing the progression of chronic kidney disease: a systematic review and meta-analysis. Clin J Am Soc Nephrol. 2009;4(3):542-551. | Included but not synthesized/presented |
| 615 Ngaage DL, Bland JM. Lessons from aprotinin: is the routine use and inconsistent dosing of tranexamic acid prudent? Meta-analysis of randomised and large matched observational studies. Eur J Cardiothorac Surg. 2010;37(6):1375-1383. | Included but not synthesized/presented |
| 616 Parker M, Raval P, Gjertsen JE. Nail or plate fixation for A3 trochanteric hip fractures: A systematic review of randomised controlled trials. Injury. 2018;49(7):1319-1323. | Included but not synthesized/presented |
| 617 Rasmussen-Barr E, Held U, Grooten WJ, et al. Non-steroidal anti-inflammatory drugs for sciatica. Cochrane Database Syst Rev. 2016(10):CD012382. | Included but not synthesized/presented |
| 618 Reviewers C I G A. Human albumin administration in critically ill patients: systematic review of randomised controlled trials. BMJ: British Medical Journal, 1998: 235-240. | Included but not synthesized/presented |
| 619 Richy F, Schacht E, Bruyere O, et al. Vitamin D analogs versus native vitamin D in preventing bone loss and osteoporosis-related fractures: a comparative meta-analysis. Calcif Tissue Int. 2005;76(3):176-186. | Included but not synthesized/presented |
| 620 Rodrigues Junior JI, Vasconcelos JKG, Xavier LEMDS, et al. Antioxidant Therapy in Inflammatory Bowel Disease: A Systematic Review and a Meta-Analysis of Randomized Clinical Trials. Pharmaceuticals (Basel). 2023;16(10):1374. | Included but not synthesized/presented |
| 621 Schnabel A, Meyer-Frießem CH, Reichl SU, et al. Is intraoperative dexmedetomidine a new option for postoperative pain treatment? A meta-analysis of randomized controlled trials. Pain. 2013;154(7):1140-1149. | Included but not synthesized/presented |

| | |
|---|---|
| 622 Schuch FB, Deslandes AC, Stubbs B, et al. Neurobiological effects of exercise on major depressive disorder: A systematic review. Neurosci Biobehav Rev. 2016;61:1-11. | Included but not synthesized/presented |
| 623 Simnett SJ, Stewart LA, Sweetenham J,et al. Autologous stem cell transplantation for malignancy: a systematic review of the literature. Clin Lab Haematol. 2000;22(2):61-72. | Included but not synthesized/presented |
| 624 Singh A, Sheth PG, Dhaneria S,et al. Efficacy and safety of ivermectin for COVID-19: A systematic review and meta-analysis. Asian Pacific Journal of Tropical Medicine. 2021;14(10):440-50. | Included but not synthesized/presented |
| 625 Sitjà-Rabert M, Rigau D, Fort Vanmeerghaeghe A, et al. Efficacy of whole body vibration exercise in older people: a systematic review. Disabil Rehabil. 2012;34(11):883-893. | Included but not synthesized/presented |
| 626 Smedslund G, Ringdal GI. Meta-analysis of the effects of psychosocial interventions on survival time in cancer patients. J Psychosom Res. 2004;57(2):123-135. | Included but not synthesized/presented |
| 627 Stratigaki E, Tong HJ, Seremidi K,et al. Contemporary management of deep caries in primary teeth: a systematic review and meta-analysis. Eur Arch Paediatr Dent. 2022;23(5):695-725. | Included but not synthesized/presented |
| 628 Stuijver DJ, Romualdi E, van Zaane B, et al. Under-reporting of venous and arterial thrombotic events in randomized clinical trials: a meta-analysis. Intern Emerg Med. 2015;10(2):219-246. | Included but not synthesized/presented |
| 629 Sutar R, Sahu S. Pharmacotherapy for dissociative disorders: A systematic review. Psychiatry Res. 2019;281:112529. | Included but not synthesized/presented |
| 630 Tena MÁ, Urso S, González JM, et al. Levosimendan versus placebo in cardiac surgery: a systematic review and meta-analysis. Interact Cardiovasc Thorac Surg. 2018;27(5):677-685. | Included but not synthesized/presented |
| 631 Tsiami AP, Goulis DG, Sotiriadis AI, et al. Higher ovulation rate with letrozole as compared with clomiphene citrate in infertile women with polycystic ovary syndrome: a systematic review and meta-analysis. Hormones (Athens). 2021;20(3):449-461. | Included but not synthesized/presented |
| 632 Verheyden GS, Weerdesteyn V, Pickering RM, et al. Interventions for preventing falls in people after stroke. Cochrane Database Syst Rev. 2013(5):CD008728. | Included but not synthesized/presented |
| 633 Wiley E, Khattab S, Tang A. Examining the effect of virtual reality therapy on cognition post-stroke: a systematic review and meta-analysis. Disabil Rehabil Assist Technol. 2022;17(1):50-60. | Included but not synthesized/presented |
| 634 Yang J, Hu X, Zhang Q, et al. Homocysteine level and risk of fracture: A meta-analysis and systematic review. Bone. 2012;51(3):376-382. | Included but not synthesized/presented |
| 635 Yang YS, Wu SH, Chen WC, et al. Effects of xenon anesthesia on postoperative neurocognitive disorders: a systematic review and meta-analysis. BMC Anesthesiol. 2023;23(1):366. | Included but not synthesized/presented |
| 636 Yu J, Zhang C, Li L, et al. Internal fixation treatments for intertrochanteric fracture: a systematic review and meta-analysis of randomized evidence. Sci Rep. 2015;5:18195. | Included but not synthesized/presented |

| | |
|---|---|
| 637 Zhang J, Zhou K, Luo X, et al. Variation of Laparoscopic Ovarian Drilling for Clomiphene Citrate-resistant Patients with Polycystic Ovary Syndrome and Infertility: A Meta-analysis. J Minim Invasive Gynecol. 2020;27(5):1048-1058. | Included but not synthesized/presented |
| 638 Zhou C, Wang W, Wang J, et al. An Updated Meta-Analysis of Laparoscopic Versus Open Repair for Perforated Peptic Ulcer. Sci Rep. 2015;5:13976. | Included but not synthesized/presented |
| 639 Guan B, Chong TH, Peng J, et al. Mid-long-term Revisional Surgery After Sleeve Gastrectomy: a Systematic Review and Meta-analysis. Obes Surg. 2019;29(6):1965-1975. | Incidence |
| 640 Nagraj S, Varrias D, Hernandez Romero G, et al. Incidence of Stroke in Randomized Trials of COVID-19 Therapeutics: A Systematic Review and Meta-Analysis. Stroke. 2022;53(11):3410-3418. | Incidence |
| 641 Yayan J. Denosumab for Effective Tumor Size Reduction in Patients With Giant Cell Tumors of the Bone: A Systematic Review and Meta-Analysis. Cancer Control. 2020;27(3):1073274820934822. | Incidence |
| 642 Lan T, Chang L, Mn R,et al. Comparative Efficacy of Interventional Therapies for Early-stage Hepatocellular Carcinoma: A PRISMA-compliant Systematic Review and Network Meta-analysis. Medicine (Baltimore). 2016;95(15):e3185. | Forest plot was vague |
| 643 Yu Y, Fu J, Xia P, et al. A systematic review and meta-analysis on the efficacy and safety of transcatheter arterial chemoembolization combined with radiofrequency ablation in the treatment of primary liver cancer. Transl Cancer Res. 2022;11(5):1297-1308. | Figure file cannot open |
| 644 Shah PS, Torgalkar R, Shah VS. Breastfeeding or breast milk for procedural pain in neonates. Cochrane Database Syst Rev. 2023(8):CD004950. | Duplicate |
| 645 Yang Y, Li L, Qu C,et al. Endoscopic ultrasound-guided fine needle core biopsy for the diagnosis of pancreatic malignant lesions: a systematic review and Meta-Analysis. Sci Rep. 2016;6:22978. | Diagnostic meta |
| 646 Abdelhamid AS, Brown TJ, Brainard JS, et al. Omega-3 fatty acids for the primary and secondary prevention of cardiovascular disease. Cochrane Database Syst Rev. 2018(7):CD003177. | Review author excluded this study in SR |
| 647 Abdelhamid AS, Brown TJ, Brainard JS, et al. Omega-3 fatty acids for the primary and secondary prevention of cardiovascular disease. Cochrane Database Syst Rev. 2020(3):CD003177. | Review author excluded this study in SR |
| 648 Abud R, Salgueiro M, Drake L, et al. Efficacy of continuous positive airway pressure (CPAP) preventing type 2 diabetes mellitus in patients with obstructive sleep apnea hypopnea syndrome (OSAHS) and insulin resistance: a systematic review and meta-analysis. Sleep Med. 2019;62:14-21. | Review author excluded this study in SR |
| 649 Agur W, Riad M, Secco S, et al. Surgical treatment of recurrent stress urinary incontinence in women: a systematic review and meta-analysis of randomised controlled trials. Eur Urol. 2013;64(2):323-336. | Review author excluded this study in SR |

| | |
|---|---|
| 650 Allen NE, Canning CG, Almeida LRS, et al. Interventions for preventing falls in Parkinson's disease. Cochrane Database Syst Rev. 2022(6):CD011574. | Review author excluded this study in SR |
| 651 Ardern CL, Webster KE, Taylor NF, et al. Return to sport following anterior cruciate ligament reconstruction surgery: a systematic review and meta-analysis of the state of play. Br J Sports Med. 2011;45(7):596-606. | Review author excluded this study in SR |
| 652 Argano C, Mallaci Bocchio R, Natoli G, et al. Protective Effect of Vitamin D Supplementation on COVID-19-Related Intensive Care Hospitalization and Mortality: Definitive Evidence from Meta-Analysis and Trial Sequential Analysis. Pharmaceuticals (Basel). 2023;16(1):130. | Review author excluded this study in SR |
| 653 Bain E, Wilson A, Tooher R, et al. Prophylaxis for venous thromboembolic disease in pregnancy and the early postnatal period. Cochrane Database Syst Rev. 2014(2):CD001689. | Review author excluded this study in SR |
| 654 Bampoe S, Odor PM, Dushianthan A, et al. Perioperative administration of buffered versus non-buffered crystalloid intravenous fluid to improve outcomes following adult surgical procedures. Cochrane Database Syst Rev. 2017(9):CD004089. | Review author excluded this study in SR |
| 655 Berstock JR, Murray JR, Whitehouse MR, . Medial subvastus versus the medial parapatellar approach for total knee replacement: A systematic review and meta-analysis of randomized controlled trials. EFORT Open Rev. 2018;3(3):78-84. | Review author excluded this study in SR |
| 656 Bislev LS, Grove-Laugesen D, Rejnmark L. Vitamin D and Muscle Health: A Systematic Review and Meta-analysis of Randomized Placebo-Controlled Trials. J Bone Miner Res. 2021;36(9):1651-1660. | Review author excluded this study in SR |
| 657 Bolignano D, Palmer SC, Navaneethan SD, et al. Aldosterone antagonists for preventing the progression of chronic kidney disease. Cochrane Database Syst Rev. 2014;(4):CD007004. | Review author excluded this study in SR |
| 658 Bolton CM, Myles PS, Nolan T, et al. Prophylaxis of postoperative vomiting in children undergoing tonsillectomy: a systematic review and meta-analysis. Br J Anaesth. 2006;97(5):593-604. | Review author excluded this study in SR |
| 659 Bosteels J, Kasius J, Weyers S, et al. Hysteroscopy for treating subfertility associated with suspected major uterine cavity abnormalities. Cochrane Database Syst Rev. 2015(2):CD009461. | Review author excluded this study in SR |

| | |
|---|---|
| 660 Boyle RJ, Ierodiakonou D, Khan T, et al. Hydrolysed formula and risk of allergic or autoimmune disease: systematic review and meta-analysis. BMJ. 2016;352:i974. | Review author excluded this study in SR |
| 661 Bulley S, Derry S, Moore RA, et al. Single dose oral rofecoxib for acute postoperative pain in adults. Cochrane Database Syst Rev.2009(4):CD004604. | Review author excluded this study in SR |
| 662 Bunn F, Trivedi D. Colloid solutions for fluid resuscitation. Cochrane Database Syst Rev. 2012(7):CD001319. | Review author excluded this study in SR |
| 663 Burdett E, Dushianthan A, Bennett-Guerrero E, et al. Perioperative buffered versus non-buffered fluid administration for surgery in adults. Cochrane Database Syst Rev. 2012;12:CD004089. | Review author excluded this study in SR |
| 664 Cantineau AE, Rutten AG, Cohlen BJ. Agents for ovarian stimulation for intrauterine insemination (IUI) in ovulatory women with infertility. Cochrane Database Syst Rev. 2021(11):CD005356. | Review author excluded this study in SR |
| 665 Cao M, He C, Gong M, et al. The effects of vitamin D on all-cause mortality in different diseases: an evidence-map and umbrella review of 116 randomized controlled trials. Front Nutr. 2023;10:1132528. | Umbrella review |
| 666 Carvalho FR, Lentini-Oliveira DA, Prado LB, et al. Oral appliances and functional orthopaedic appliances for obstructive sleep apnoea in children. Cochrane Database Syst Rev. 2016(10):CD005520. | Review author excluded this study in SR |
| 667 Chafen JJ, Newberry SJ, Riedl MA, et al. Diagnosing and managing common food allergies: a systematic review. JAMA. 2010;303(18):1848-1856. | Review author excluded this study in SR |
| 668 Chaparro LE, Smith SA, Moore RA, et al. Pharmacotherapy for the prevention of chronic pain after surgery in adults. Cochrane Database Syst Rev.2013(7):CD008307. | Review author excluded this study in SR |
| 669 Chen LD, Lin L, Lin XJ, et al. Effect of continuous positive airway pressure on carotid intima-media thickness in patients with obstructive sleep apnea: A meta-analysis. PLoS One. 2017;12(9):e0184293. | Review author excluded this study in SR |
| 670 Chen QW, Ying HF, Gao S, et al. Radiofrequency ablation plus chemoembolization versus radiofrequency ablation alone for hepatocellular carcinoma: A systematic review and meta-analysis. Clin Res Hepatol Gastroenterol. 2016;40(3):309-314. | Review author excluded this study in SR |

| | |
|---|---|
| 671 Chooi C, Cox JJ, Lumb RS, et al. Techniques for preventing hypotension during spinal anaesthesia for caesarean section. Cochrane Database Syst Rev. 2020(7):CD002251. | Review author excluded this study in SR |
| 672 Chung EY, Ruospo M, Natale P, et al. Aldosterone antagonists in addition to renin angiotensin system antagonists for preventing the progression of chronic kidney disease. Cochrane Database Syst Rev. 2020(10):CD007004. | Review author excluded this study in SR |
| 673 Clark O, Botrel TE, Paladini L, et al. Targeted therapy in triple-negative metastatic breast cancer: a systematic review and meta-analysis. Core Evid. 2014;9:1-11. | Review author excluded this study in SR |
| 674 Coomarasamy A, Papaioannou S, Gee H, Khan KS. Aspirin for the prevention of preeclampsia in women with abnormal uterine artery Doppler: a meta-analysis. Obstet Gynecol. 2001;98(5 Pt 1):861-866. | Review author excluded this study in SR |
| 675 Corbett MS, Rice SJ, Madurasinghe V, et al. Acupuncture and other physical treatments for the relief of pain due to osteoarthritis of the knee: network meta-analysis. Osteoarthritis Cartilage. 2013;21(9):1290-1298. | Review author excluded this study in SR |
| 676 Curley GF, Shehata N, Mazer CD, et al. Transfusion triggers for guiding RBC transfusion for cardiovascular surgery: a systematic review and meta-analysis*. Crit Care Med. 2014;42(12):2611-2624. | Review author excluded this study in SR |
| 677 Dahabreh IJ, Steele DW, Shah N, et al. Oral Mechanical Bowel Preparation for Colorectal Surgery: Systematic Review and Meta-Analysis. Dis Colon Rectum. 2015;58(7):698-707. | Review author excluded this study in SR |
| 678 Dahmani S, Stany I, Brasher C, et al. Pharmacological prevention of sevoflurane- and desflurane-related emergence agitation in children: a meta-analysis of published studies. Br J Anaesth. 2010;104(2):216-223. | Review author excluded this study in SR |
| 679 Daly BJ, Sharif MO, Jones K, Worthington HV, Beattie A. Local interventions for the management of alveolar osteitis (dry socket). Cochrane Database Syst Rev. 2022(9):CD006968. | Review author excluded this study in SR |
| 680 de Jonge S, Egger M, Latif A, et al. Effectiveness of 80% vs 30-35% fraction of inspired oxygen in patients undergoing surgery: an updated systematic review and meta-analysis. Br J Anaesth. 2019;122(3):325-334. | Review author excluded this study in SR |

| | |
|---|---|
| 681 De Oliveira GS Jr, Almeida MD, Benzon HT, et al. Perioperative single dose systemic dexamethasone for postoperative pain: a meta-analysis of randomized controlled trials. Anesthesiology. 2011;115(3):575-588. | Review author excluded this study in SR |
| 682 de Vaan MD, Ten Eikelder ML, Jozwiak M, et al. Mechanical methods for induction of labour. Cochrane Database Syst Rev. 2023(3):CD001233. | Review author excluded this study in SR |
| 683 Di Spiezio Sardo A, Di Carlo C, Minozzi S, et al. Efficacy of hysteroscopy in improving reproductive outcomes of infertile couples: a systematic review and meta-analysis. Hum Reprod Update. 2016;22(4):479-496. | Review author excluded this study in SR |
| 684 Dieamant FC, Petersen CG, Mauri AL, et al. Fresh embryos versus freeze-all embryos - transfer strategies: Nuances of a meta-analysis. JBRA Assist Reprod. 2017;21(3):260-272. | Review author excluded this study in SR |
| 685 Duley L, Meher S, Hunter KE, et al. Antiplatelet agents for preventing pre-eclampsia and its complications. Cochrane Database Syst Rev. 2019(10):CD004659. | Review author excluded this study in SR |
| 686 El Maleh Y, Fasquel C, Quesnel C, et al. Updated meta-analysis on intraoperative inspired fraction of oxygen and the risk of surgical site infection in adults undergoing general and regional anesthesia. Sci Rep. 2023;13(1):2465. | Review author excluded this study in SR |
| 687 Engelman E, Salengros JC, Barvais L. How much does pharmacologic prophylaxis reduce postoperative vomiting in children? Calculation of prophylaxis effectiveness and expected incidence of vomiting under treatment using Bayesian meta-analysis. Anesthesiology. 2008;109(6):1023-1035. | Review author excluded this study in SR |
| 688 Euasobhon P, Dej-Arkom S, Siriussawakul A, et al. Lidocaine for reducing propofol-induced pain on induction of anaesthesia in adults. Cochrane Database Syst Rev. 2016(2):CD007874. | Review author excluded this study in SR |
| 689 Franik S, Le QK, Kremer JA, et al. Aromatase inhibitors (letrozole) for ovulation induction in infertile women with polycystic ovary syndrome. Cochrane Database Syst Rev. 2022(9):CD010287. | Review author excluded this study in SR |
| 690 Freites J, Stewart F, Omar MI, et al. Laparoscopic colposuspension for urinary incontinence in women. Cochrane Database Syst Rev. 2019(12):CD002239. | Review author excluded this study in SR |

| | |
|---|---|
| 691 Gagyor I, Madhok VB, Daly F, et al. Antiviral treatment for Bell's palsy (idiopathic facial paralysis). Cochrane Database Syst Rev. 2015(11):CD001869. | Review author excluded this study in SR |
| 692 Gagyor I, Madhok VB, Daly F, et al. Antiviral treatment for Bell's palsy (idiopathic facial paralysis). Cochrane Database Syst Rev. 2019(9):CD001869. | Review author excluded this study in SR |
| 693 García-Perdomo HA, Correa-Ochoa JJ, Contreras-García R, Daneshmand S. Effectiveness of extended pelvic lymphadenectomy in the survival of prostate cancer: a systematic review and meta-analysis. Cent European J Urol. 2018;71(3):262-269. | Review author excluded this study in SR |
| 694 Gomes ET, Carbogim FDC, Lins RS, et al. Effectiveness of supplemental oxygenation to prevent surgical site infections: A systematic review with meta-analysis. Rev Lat Am Enfermagem. 2022;30:e3648. | Review author excluded this study in SR |
| 695 Griffiths JD, Gyte GM, Popham PA, et al. Interventions for preventing nausea and vomiting in women undergoing regional anaesthesia for caesarean section. Cochrane Database Syst Rev. 2021(5):CD007579. | Review author excluded this study in SR |
| 696 Grundeis F, Ansems K, Dahms K, et al. Remdesivir for the treatment of COVID-19. Cochrane Database Syst Rev. 2023(1):CD014962. | Review author excluded this study in SR |
| 697 Gu L, Liu H, Fan L, et al. Treatment outcomes of transcatheter arterial chemoembolization combined with local ablative therapy versus monotherapy in hepatocellular carcinoma: a meta-analysis. J Cancer Res Clin Oncol. 2014;140(2):199-210. | Review author excluded this study in SR |
| 698 Guay J, Kopp S. Epidural pain relief versus systemic opioid-based pain relief for abdominal aortic surgery. Cochrane Database Syst Rev. 2016(1):CD005059. | Review author excluded this study in SR |
| 699 Haas DM, Morgan S, Contreras K, et al. Vaginal preparation with antiseptic solution before cesarean section for preventing postoperative infections. Cochrane Database Syst Rev. 2018;7(7):CD007892. | Review author excluded this study in SR |
| 700 Haas DM, Morgan S, Contreras K, et al. Vaginal preparation with antiseptic solution before cesarean section for preventing postoperative infections. Cochrane Database Syst Rev. 2020;4(4):CD007892. | Review author excluded this study in SR |

| | |
|---|---|
| 701 Hohlfeld A, Ebrahim S, Shaik MZ, et al. Circumcision devices versus standard surgical techniques in adolescent and adult male circumcisions. Cochrane Database Syst Rev. 2021;3(3):CD012250. | Review author excluded this study in SR |
| 702 Hooper L, Martin N, Abdelhamid A, et al. Reduction in saturated fat intake for cardiovascular disease. Cochrane Database Syst Rev. 2015;(6):CD011737. | Review author excluded this study in SR |
| 703 Hooper L, Martin N, Jimoh OF, et al. Reduction in saturated fat intake for cardiovascular disease. Cochrane Database Syst Rev. 2020;8(8):CD011737. | Review author excluded this study in SR |
| 704 Huey SL, Acharya N, Silver A, et al. Effects of oral vitamin D supplementation on linear growth and other health outcomes among children under five years of age. Cochrane Database Syst Rev. 2020;12(12):CD012875. | Review author excluded this study in SR |
| 705 Iftikhar IH, Hoyos CM, Phillips CL, et al. Meta-analyses of the Association of Sleep Apnea with Insulin Resistance, and the Effects of CPAP on HOMA-IR, Adiponectin, and Visceral Adipose Fat. J Clin Sleep Med. 2015;11(4):475-485. | Review author excluded this study in SR |
| 706 Jacobson Vann JC, Jacobson RM, Coyne-Beasley T, et al. Patient reminder and recall interventions to improve immunization rates. Cochrane Database Syst Rev. 2018(1):CD003941. | Review author excluded this study in SR |
| 707 Jacobson Vann JC, Szilagyi P. Patient reminder and patient recall systems to improve immunization rates. Cochrane Database Syst Rev.2005(3):CD003941. | Review author excluded this study in SR |
| 708 Jia X, McNeill G, Avenell A. Does taking vitamin, mineral and fatty acid supplements prevent cognitive decline? A systematic review of randomized controlled trials. J Hum Nutr Diet. 2008;21(4):317-336. | Review author excluded this study in SR |
| 709 Johns K, Beddall MJ, Corrin RC. Anabolic steroids for the treatment of weight loss in HIV-infected individuals. Cochrane Database Syst Rev. 2005;(4):CD005483. | Review author excluded this study in SR |
| 710 Jull AB, Cullum N, Dumville JC, et al. Honey as a topical treatment for wounds. Cochrane Database Syst Rev.2015(3):CD005083. | Review author excluded this study in SR |

| | |
|---|---|
| 711 Kamath MS, Bosteels J, D'Hooghe TM, et al. Screening hysteroscopy in subfertile women and women undergoing assisted reproduction. Cochrane Database Syst Rev. 2019(4):CD012856. | Review author excluded this study in SR |
| 712 Kang YN, Chi SC, Wu MH, et al. The effects of losartan versus beta-blockers on cardiovascular protection in marfan syndrome: A systematic review and meta-analysis. J Formos Med Assoc. 2020;119(1 Pt 1):182-190. | Review author excluded this study in SR |
| 713 Kip A, Priebe S, Holling H, et al. Psychological interventions for posttraumatic stress disorder and depression in refugees: A meta-analysis of randomized controlled trials. Clin Psychol Psychother. 2020;27(4):489-503. | Review author excluded this study in SR |
| 714 Kirchin V, Page T, Keegan PE, et al. Urethral injection therapy for urinary incontinence in women. Cochrane Database Syst Rev. 2017(7):CD003881. | Review author excluded this study in SR |
| 715 Kong LJ, Lauche R, Klose P, et al. Tai Chi for Chronic Pain Conditions: A Systematic Review and Meta-analysis of Randomized Controlled Trials. Sci Rep. 2016;6:25325. | Review author excluded this study in SR |
| 716 Kramer MS, Kakuma R. Maternal dietary antigen avoidance during pregnancy or lactation, or both, for preventing or treating atopic disease in the child. Evid Based Child Health. 2014;9(2):447-483. | Review author excluded this study in SR |
| 717 Lai LY, Tafuri SM, Ginier EC, et al. Narrow band imaging versus white light cystoscopy alone for transurethral resection of non-muscle invasive bladder cancer. Cochrane Database Syst Rev. 2022(4):CD014887. | Review author excluded this study in SR |
| 718 Lane DA, Lip GY. Treatment of hypertension in peripheral arterial disease. Cochrane Database Syst Rev. 2013;(12):CD003075. | Review author excluded this study in SR |
| 719 Lee A, Chan DL, Wong MH, et al. Systematic Review of the Role of Targeted Therapy in Metastatic Neuroendocrine Tumors. Neuroendocrinology. 2017;104(3):209-222. | Review author excluded this study in SR |
| 720 Levi M, Cromheecke ME, de Jonge E, et al. Pharmacological strategies to decrease excessive blood loss in cardiac surgery: a meta-analysis of clinically relevant endpoints. Lancet. 1999;354(9194):1940-1947. | Review author excluded this study in SR |

| | |
|---|---|
| 721 Lewis SR, Nicholson A, Smith AF, et al. Alpha-2 adrenergic agonists for the prevention of shivering following general anaesthesia. Cochrane Database Syst Rev.2015(8):CD011107. | Review author excluded this study in SR |
| 722 Li B, Xu Y, Zhang X, et al. The effect of vitamin D supplementation in treatment of children with autism spectrum disorder: a systematic review and meta-analysis of randomized controlled trials. Nutr Neurosci. 2022;25(4):835-845. | Review author excluded this study in SR |
| 723 Li R, Chen H, Feng J, et al. Effectiveness of Traditional Chinese Exercise for Symptoms of Knee Osteoarthritis: A Systematic Review and Meta-Analysis of Randomized Controlled Trials. Int J Environ Res Public Health. 2020;17(21):7873. | Review author excluded this study in SR |
| 724 Liao M, Huang J, Zhang T, et al. Transarterial chemoembolization in combination with local therapies for hepatocellular carcinoma: a meta-analysis. PLoS One. 2013;8(7):e68453. | Review author excluded this study in SR |
| 725 Lin MT, Lin HH, Lee PL, et al. Beneficial effect of continuous positive airway pressure on lipid profiles in obstructive sleep apnea: a meta-analysis. Sleep Breath. 2015;19(3):809-817. | Review author excluded this study in SR |
| 726 Liu HM, Chen JH, Chen C, et al. Prophylactic antiemetic effects of dexamethasone versus 5-HT3 receptor antagonists in ear surgery: a systematic review and meta-analysis. Int J Clin Pharm. 2021;43(3):476-485. | Review author excluded this study in SR |
| 727 Liu S, Athar A, Quach D, et al. Risks and benefits of oral modified-release compared with oral immediate-release opioid use after surgery: a systematic review and meta-analysis. Anaesthesia. 2023;78(10):1225-1236. | Review author excluded this study in SR |
| 728 Liu Z, Gao F, Yang G, et al. Combination of radiofrequency ablation with transarterial chemoembolization for hepatocellular carcinoma: an up-to-date meta-analysis. Tumour Biol. 2014;35(8):7407-7413. | Review author excluded this study in SR |
| 729 Liu Z, Geng Y, Huang Y, et al. Letrozole Compared With Clomiphene Citrate for Polycystic Ovarian Syndrome: A Systematic Review and Meta-analysis. Obstet Gynecol. 2023;141(3):523-534. | Review author excluded this study in SR |
| 730 Long G, Liu C, Liang T, et al. The efficacy of thoracolumbar interfascial plane block for lumbar spinal surgeries: a systematic review and meta-analysis. J Orthop Surg Res. 2023;18(1):318. | Review author excluded this study in SR |

| | |
|---|---|
| 731 Maione A, Navaneethan SD, Graziano G, et al. Angiotensin-converting enzyme inhibitors, angiotensin receptor blockers and combined therapy in patients with micro- and macroalbuminuria and other cardiovascular risk factors: a systematic review of randomized controlled trials. Nephrol Dial Transplant. 2011;26(9):2827-2847. | Review author excluded this study in SR |
| 732 Manoj P, Derwin R, George S. What is the impact of daily oral supplementation of vitamin D3 (cholecalciferol) plus calcium on the incidence of hip fracture in older people? A systematic review and meta-analysis. Int J Older People Nurs. 2023;18(1):e12492. | Review author excluded this study in SR |
| 733 Marcolino MS, Meira KC, Guimarães NS, et al. Systematic review and meta-analysis of ivermectin for treatment of COVID-19: evidence beyond the hype. BMC Infect Dis. 2022;22(1):639. | Review author excluded this study in SR |
| 734 Martin N, Germanò R, Hartley L, et al. Nut consumption for the primary prevention of cardiovascular disease. Cochrane Database Syst Rev. 2015(9):CD011583. | Review author excluded this study in SR |
| 735 McCleery J, Abraham RP, Denton DA, et al. Vitamin and mineral supplementation for preventing dementia or delaying cognitive decline in people with mild cognitive impairment. Cochrane Database Syst Rev. 2018(11):CD011905. | Review author excluded this study in SR |
| 736 McDaid C, Maund E, Rice S, et al. Paracetamol and selective and non-selective non-steroidal anti-inflammatory drugs (NSAIDs) for the reduction of morphine-related side effects after major surgery: a systematic review. Health Technol Assess. 2010;14(17):1-iv. | Review author excluded this study in SR |
| 737 McKenzie KM, Lee CM, Mijatovic J, et al. Medium-Chain Triglyceride Oil and Blood Lipids: A Systematic Review and Meta-Analysis of Randomized Trials. J Nutr. 2021;151(10):2949-2956. | Review author excluded this study in SR |
| 738 Metwally M, Raybould G, Cheong YC, et al. Surgical treatment of fibroids for subfertility. Cochrane Database Syst Rev. 2020(1):CD003857. | Review author excluded this study in SR |
| 739 Mikola T, Marx W, Lane MM, et al. The effect of vitamin D supplementation on depressive symptoms in adults: A systematic review and meta-analysis of randomized controlled trials. Crit Rev Food Sci Nutr. 2023;63(33):11784-11801. | Review author excluded this study in SR |
| 740 Minozzi S, Pifferi S, Brazzi L, et al. Topical antibiotic prophylaxis to reduce respiratory tract infections and mortality in adults receiving mechanical ventilation. Cochrane Database Syst Rev. 2021(1):CD000022. | Review author excluded this study in SR |

| | |
|---|---|
| 741 Mishriky BM, Cummings DM, Tanenberg RJ. The efficacy and safety of DPP4 inhibitors compared to sulfonylureas as add-on therapy to metformin in patients with Type 2 diabetes: A systematic review and meta-analysis. Diabetes Res Clin Pract. 2015;109(2):378-388. | Review author excluded this study in SR |
| 742 Muley A, Fernandez R, Ellwood L,et al. Effect of tree nuts on glycemic outcomes in adults with type 2 diabetes mellitus: a systematic review. JBI Evid Synth. 2021;19(5):966-1002. | Review author excluded this study in SR |
| 743 Musini VM, Gueyffier F, Puil L, et al. Pharmacotherapy for hypertension in adults aged 18 to 59 years. Cochrane Database Syst Rev. 2017(8):CD008276. | Review author excluded this study in SR |
| 744 Musini VM, Tejani AM, Bassett K, et al. Pharmacotherapy for hypertension in adults 60 years or older. Cochrane Database Syst Rev. 2019(6):CD000028. | Review author excluded this study in SR |
| 745 Navaneethan SD, Nigwekar SU, Sehgal AR,et al. Aldosterone antagonists for preventing the progression of chronic kidney disease. Cochrane Database Syst Rev. 2009(3):CD007004. | Review author excluded this study in SR |
| 746 Neil-Sztramko SE, Caldwell H, Dobbins M. School-based physical activity programs for promoting physical activity and fitness in children and adolescents aged 6 to 18. Cochrane Database Syst Rev. 2021;9(9):CD007651. | Review author excluded this study in SR |
| 747 Nijjar SK, D'Amico MI, Wimalaweera NA, et al. Participation in clinical trials improves outcomes in women's health: a systematic review and meta-analysis. BJOG. 2017;124(6):863-871. | Review author excluded this study in SR |
| 748 Novara G, Ficarra V, Boscolo-Berto R, et al. Tension-free midurethral slings in the treatment of female stress urinary incontinence: a systematic review and meta-analysis of randomized controlled trials of effectiveness Eur Urol. 2007;52(3):663-678. | Review author excluded this study in SR |
| 749 Oines MN, Krarup PM, Jorgensen LN, et al. Pharmacological interventions for improved colonic anastomotic healing: a meta-analysis. World J Gastroenterol. 2014;20(35):12637-12648. | Review author excluded this study in SR |
| 750 Osborn DA, Sinn J. Soy formula for prevention of allergy and food intolerance in infants. Cochrane Database Syst Rev.2006(4):CD003741. | Review author excluded this study in SR |

| | |
|---|---|
| 751 Osland EJ, Yunus RM, Khan S,et al. Five-year Comorbidity Outcomes in Laparoscopic Vertical Sleeve Gastrectomy (LVSG) and Laparoscopic Roux-en-Y Gastric Bypass (LRYGB): A Systematic Review and Meta-analysis of Randomized Controlled Trials. Surg Laparosc Endosc Percutan Tech. 2023;33(3):241-248. | Review author excluded this study in SR |
| 752 Osland ST, Steeves TD, Pringsheim T. Pharmacological treatment for attention deficit hyperactivity disorder (ADHD) in children with comorbid tic disorders. Cochrane Database Syst Rev. 2018(6):CD007990. | Review author excluded this study in SR |
| 753 Patel N, Kellezi B, Williams AC. Psychological, social and welfare interventions for psychological health and well-being of torture survivors. Cochrane Database Syst Rev.2014(11):CD009317. | Review author excluded this study in SR |
| 754 Pei YY, Zhang Y, Peng XC,et al. Association of Vitamin D Supplementation with Cardiovascular Events: A Systematic Review and Meta-Analysis. Nutrients. 2022;14(15):3158. | Review author excluded this study in SR |
| 755 Pham A, Liu G. Dexamethasone for antiemesis in laparoscopic gynecologic surgery: a systematic review and meta-analysis. Obstet Gynecol. 2012;120(6):1451-1458. | Review author excluded this study in SR |
| 756 Polderman JAW, Farhang-Razi V, van Dieren S, et al. Adverse side-effects of dexamethasone in surgical patients - an abridged Cochrane systematic review. Anaesthesia. 2019;74(7):929-939. | Review author excluded this study in SR |
| 757 Punjasawadwong Y, Boonjeungmonkol N, Phongchiewboon A. Bispectral index for improving anaesthetic delivery and postoperative recovery. Cochrane Database Syst Rev. 2007;(4):CD003843. | Review author excluded this study in SR |
| 758 Roccarina D, Majumdar A, Thorburn D, et al. Management of people with intermediate-stage hepatocellular carcinoma: an attempted network meta-analysis. Cochrane Database Syst Rev. 2017;3(3):CD011649. | Review author excluded this study in SR |
| 759 Rosenberg JE, Ergun O, Hwang EC, et al. Non-surgical therapies for Peyronie's disease. Cochrane Database Syst Rev. 2023;7(7):CD012206. | Review author excluded this study in SR |
| 760 Roviello G, Zanotti L, Venturini S, et al. Role of targeted agents in neuroendocrine tumors: Results from a meta-analysis. Cancer Biol Ther. 2016;17(9):883-888. | Review author excluded this study in SR |

| | |
|---|---|
| 761 Saha S, Saha S, Gayen M. The dietary supplements effect on metabolic markers in non-pharmacologically managed gestational diabetes mellitus patients: a systematic review and meta-analysis and meta-regression of randomized controlled trials. J Diabetes Metab Disord. 2023;23(1):943-966. | Review author excluded this study in SR |
| 762 Salhab M, Canelo R. An overview of evidence-based management of hepatocellular carcinoma: a meta-analysis. J Cancer Res Ther. 2011;7(4):463-475. | Review author excluded this study in SR |
| 763 Salman MA, Issa M, Salman A, et al. Surgical Management of Perforated Peptic Ulcer: A Comparative Meta-analysis of Laparoscopic Versus Open Surgery. Surg Laparosc Endosc Percutan Tech. 2022;32(5):586-594. | Review author excluded this study in SR |
| 764 Schnabel A, Weibel S, Reichl SU, et al. Efficacy and adverse events of selective serotonin noradrenaline reuptake inhibitors in the management of postoperative pain: A systematic review and meta-analysis. J Clin Anesth. 2021;75:110451. | Review author excluded this study in SR |
| 765 Schrijver J, Lenferink A, Brusse-Keizer M, et al. Self-management interventions for people with chronic obstructive pulmonary disease. Cochrane Database Syst Rev. 2022(1):CD002990. | Review author excluded this study in SR |
| 766 Sokolakis I, Hatzichristodoulou G. Clinical studies on low intensity extracorporeal shockwave therapy for erectile dysfunction: a systematic review and meta-analysis of randomised controlled trials. Int J Impot Res. 2019;31(3):177-194. | Review author excluded this study in SR |
| 767 Solé T, Januel L, Denneval A, et al. Time impact on the antidiabetic effects of key bariatric surgeries: a network meta-analysis of randomized controlled trials with meta-regression. Surg Obes Relat Dis. 2022;18(6):832-845. | Review author excluded this study in SR |
| 768 Song Z, Shi S, Zhang Y. Ivermectin for treatment of COVID-19: A systematic review and meta-analysis. Heliyon, 2024, 10(6). | Review author excluded this study in SR |
| 769 Song Z, Shi S, Zhang Y. Ivermectin for treatment of COVID-19: A systematic review and meta-analysis. Heliyon. 2024;10(6):e27647. | Review author excluded this study in SR |
| 770 Stevens B, Yamada J, Lee GY, et al. Sucrose for analgesia in newborn infants undergoing painful procedures. Cochrane Database Syst Rev. 2013;(1):CD001069. | Review author excluded this study in SR |

| | |
|---|---|
| 771 Stevens B, Yamada J, Ohlsson A, et al. Sucrose for analgesia in newborn infants undergoing painful procedures. Cochrane Database Syst Rev. 2016(7):CD001069. | Review author excluded this study in SR |
| 772 Steward DL, Grisel J, Meinzen-Derr J. Steroids for improving recovery following tonsillectomy in children. Cochrane Database Syst Rev. 2011(8):CD003997. | Review author excluded this study in SR |
| 773 Supbumrung S, Kaewborisutsakul A, Kitsiripant C,et al. Effect of the enhanced recovery protocol in patients with brain tumors undergoing elective craniotomies: a systematic review and meta-analysis. Neurosurg Focus. 2023;55(6):E7. | Review author excluded this study in SR |
| 774 Teixeira DM, Hadyme Miyague A, Barbosa MA, et al. Regular (ICSI) versus ultra-high magnification (IMSI) sperm selection for assisted reproduction. Cochrane Database Syst Rev. 2020(2):CD010167. | Review author excluded this study in SR |
| 775 Vale N, Nordmann AJ, Schwartz GG, et al. Statins for acute coronary syndrome. Cochrane Database Syst Rev.2014(9):CD006870. | Review author excluded this study in SR |
| 776 Veronese N, Dominguez LJ, Pizzol D, et al. Oral Magnesium Supplementation for Treating Glucose Metabolism Parameters in People with or at Risk of Diabetes: A Systematic Review and Meta-Analysis of Double-Blind Randomized Controlled Trials. Nutrients. 2021;13(11):4074. | Review author excluded this study in SR |
| 777 Wang W, Song X, Wang T, et al. 5-HT3 Receptor Antagonists for the Prevention of Perioperative Shivering: A Meta-Analysis. J Clin Pharmacol. 2017;57(4):428-439. | Review author excluded this study in SR |
| 778 Wang X, Hu Y, Ren M, et al. Efficacy and Safety of Radiofrequency Ablation Combined with Transcatheter Arterial Chemoembolization for Hepatocellular Carcinomas Compared with Radiofrequency Ablation Alone: A Time-to-Event Meta-Analysis. Korean J Radiol. 2016;17(1):93-102. | Review author excluded this study in SR |
| 779 Weis S, Franke A, Mössner J, et al. Radiofrequency (thermal) ablation versus no intervention or other interventions for hepatocellular carcinoma. Cochrane Database Syst Rev. 2013(12):CD003046. | Review author excluded this study in SR |
| 780 Wennerholm UB, Bergman L, Kuusela P, et al. Progesterone, cerclage, pessary, or acetylsalicylic acid for prevention of preterm birth in singleton and multifetal pregnancies - A systematic review and meta-analyses. Front Med (Lausanne). 2023;10:1111315. | Review author excluded this study in SR |

| | |
|---|---|
| 781 Wessel JA, Danhof NA, van Eekelen R, et al. Ovarian stimulation strategies for intrauterine insemination in couples with unexplained infertility: a systematic review and individual participant data meta-analysis. Hum Reprod Update. 2022;28(5):733-746. | Review author excluded this study in SR |
| 782 Williams AC, Eccleston C, Morley S. Psychological therapies for the management of chronic pain (excluding headache) in adults. Cochrane Database Syst Rev. 2012(11):CD007407. | Review author excluded this study in SR |
| 783 Wilson A, Hodgetts-Morton VA, Marson EJ, et al. Tocolytics for delaying preterm birth: a network meta-analysis (0924). Cochrane Database Syst Rev. 2022(8):CD014978. | Review author excluded this study in SR |
| 784 Wrzosek A, Jakowicka-Wordliczek J, Zajaczkowska R, et al. Perioperative restrictive versus goal-directed fluid therapy for adults undergoing major non-cardiac surgery. Cochrane Database Syst Rev. 2019(12):CD012767. | Review author excluded this study in SR |
| 785 Yamada J, Bueno M, Santos L, et al. Sucrose analgesia for heel-lance procedures in neonates. Cochrane Database Syst Rev. 2023(8):CD014806. | Review author excluded this study in SR |
| 786 Yan JH, Gu WJ, Sun J, et al. Efficacy of Tai Chi on pain, stiffness and function in patients with osteoarthritis: a meta-analysis. PLoS One. 2013;8(4):e61672. | Review author excluded this study in SR |
| 787 Zaat T, Zagers M, Mol F,et al. Fresh versus frozen embryo transfers in assisted reproduction. Cochrane Database Syst Rev. 2021(2):CD011184. | Review author excluded this study in SR |
| 788 Zhang J, Sun R, Cai Y,et al. Efficacy and Safety of Antidiabetic Agents for Major Depressive Disorder and Bipolar Depression: A Meta-Analysis of Randomized, Double-Blind, Placebo-Controlled Trials. J Clin Med. 2024;13(4):1172.. | Review author excluded this study in SR |
| 789 Zhang Z, Yang C, Zhang LL, et al. Pharmacotherapies to tics: a systematic review. Oncotarget. 2018;9(46):28240-28266. | Review author excluded this study in SR |
| 790 Zhao YT, Li PY, Zhang JQ,et al. Angiotensin II Receptor Blockers and Cancer Risk: A Meta-Analysis of Randomized Controlled Trials. Medicine (Baltimore). 2016;95(18):e3600. | Review author excluded this study in SR |

| | |
|---|---|
| 791 Zhong Z, Zhao L, Zhao Y, et al. High-dose vitamin D supplementation in patients with COVID-19: A meta-analysis of randomized controlled trials. Food Sci Nutr. 2023;12(3):1808-1817. | Review author excluded this study in SR |
| 792 Zwetsloot PP, Végh AM, Jansen of Lorkeers SJ, et al. Cardiac Stem Cell Treatment in Myocardial Infarction: A Systematic Review and Meta-Analysis of Preclinical Studies. Circ Res. 2016;118(8):1223-1232. | Animal |
| 793 Tu X, Dong Y, Zhang H, et al. Corticosteroids for Graves' Ophthalmopathy: Systematic Review and Meta-Analysis. Biomed Res Int. 2018;2018:4845894. | Included but not synthesized/presented |
| 794 Abdelhamid A, Hooper L, Sivakaran R, et al; PUFAH Group. The Relationship Between Omega-3, Omega-6 and Total Polyunsaturated Fat and Musculoskeletal Health and Functional Status in Adults: A Systematic Review and Meta-analysis of RCTs. Calcif Tissue Int. 2019;105(4):353-372. | Insufficient information for reproducing |
| 795 Abdelhamid AS, Martin N, Bridges C, et al. Polyunsaturated fatty acids for the primary and secondary prevention of cardiovascular disease. Cochrane Database Syst Rev. 2018(7):CD012345. | Included but not synthesized/presented |
| 796 Abdel-Maboud M, Menshawy A, Hasabo EA, et al. The comparative effectiveness of 55 interventions in obese patients with polycystic ovary syndrome: A network meta-analysis of 101 randomized trials. PLoS One. 2021;16(7):e0254412. | Not report study-level data |
| 797 Ajabnoor SM, Thorpe G, Abdelhamid A, et al. Long-term effects of increasing omega-3, omega-6 and total polyunsaturated fats on inflammatory bowel disease and markers of inflammation: a systematic review and meta-analysis of randomized controlled trials. Eur J Nutr. 2021;60(5):2293-2316. | Insufficient information for reproducing |
| 798 Alotaibi FF, Rocchietta I, Buti J, et al. Comparative evidence of different surgical techniques for the management of vertical alveolar ridge defects in terms of complications and efficacy: A systematic review and network meta-analysis. J Clin Periodontol. 2023;50(11):1487-1519. | Not report study-level data |
| 799 Annweiler C, Milea D, Whitson HE, et al. Vitamin D insufficiency and cognitive impairment in Asians: a multi-ethnic population-based study and meta-analysis. J Intern Med. 2016;280(3):300-311. | Insufficient information for reproducing |
| 800 Arnold DM, Fergusson DA, Chan AK, et al. Avoiding transfusions in children undergoing cardiac surgery: a meta-analysis of randomized trials of aprotinin. Anesth Analg. 2006;102(3):731-737. | Insufficient information for reproducing |
| 801 Azeez TA, Lakoh S, Adeleke AA,et al. Chemoprophylaxis against COVID-19 among health-care workers using Ivermectin in low- and middle-income countries: A systematic review and meta-analysis. Indian J Pharmacol. 2021;53(6):493-498. | Insufficient information for reproducing |
| 802 Babazadeh-Zavieh SS, Bashardoust Tajali S, Haeri SMJ, et al. Effects of Transcutaneous Electrical Nerve Stimulation on Chronic Pelvic Pain in Women: A Systematic Review and Meta-Analysis. Auswirkungen der transkutanen elektrischen | Insufficient information for reproducing |

| | |
|---|---|
| Nervenstimulation auf chronischen Unterleibsschmerz bei Frauen: eine systematische Übersicht und Metaanalyse. Complement Med Res. 2023;30(2):161-173. | |
| 803 Barron ME, Wilkes MM, Navickis RJ. A systematic review of the comparative safety of colloids. Arch Surg. 2004;139(5):552-563. | Insufficient information for reproducing |
| 804 Bettiol A, Avagliano L, Lombardi N, et al. Pharmacological Interventions for the Prevention of Fetal Growth Restriction: A Systematic Review and Network Meta-Analysis. Clin Pharmacol Ther. 2021;110(1):189-199. | Not report study-level data |
| 805 Carron M, Zarantonello F, Tellaroli P, et al. Perioperative noninvasive ventilation in obese patients: a qualitative review and meta-analysis. Surg Obes Relat Dis. 2016;12(3):681-691. | Insufficient information for reproducing |
| 806 Chatzakis C, Sotiriadis A, Tsakmaki E, et al. The Effect of Dietary Supplements on Oxidative Stress in Pregnant Women with Gestational Diabetes Mellitus: A Network Meta-Analysis. Nutrients. 2021;13(7):2284. | Not report study-level data |
| 807 Cheng Q, Chen J, Jia Q,et al. Efficacy and safety of current medications for treating severe and non-severe COVID-19 patients: an updated network meta-analysis of randomized placebo-controlled trials. Aging (Albany NY). 2021;13(18):21866-21902. | Not report study-level data |
| 808 Cheng Q, Zhao G, Chen J, et al. Efficacy and safety of current treatment interventions for patients with severe COVID-19 infection: A network meta-analysis of randomized controlled trials. J Med Virol. 2022;94(4):1617-1626. | Not report study-level data |
| 809 Chi G, Memar Montazerin S, Lee JJ, et al. Effect of azithromycin and hydroxychloroquine in patients hospitalized with COVID-19: Network meta-analysis of randomized controlled trials. J Med Virol. 2021;93(12):6737-6749. | Not report study-level data |
| 810 Cho YJ, Choi GJ, Ahn EJ, et al. Pharmacologic interventions for postoperative nausea and vomiting after thyroidectomy: A systematic review and network meta-analysis. PLoS One. 2021;16(1):e0243865. | Not report study-level data |
| 811 Chow R, Simone CB 2nd, Jairam MP, et al. Radiofrequency ablation vs radiation therapy vs transarterial chemoembolization vs yttrium 90 for local treatment of liver cancer - a systematic review and network meta-analysis of survival data. Acta Oncol. 2022;61(4):484-494. | Not report study-level data |
| 812 Cottrell AM, Schneider MP, Goonewardene S, et al. Benefits and Harms of Electrical Neuromodulation for Chronic Pelvic Pain: A Systematic Review. Eur Urol Focus. 2020;6(3):559-571. | Insufficient information for reproducing |
| 813 D'Antonio F, Berghella V, Di Mascio D, et al. Role of progesterone, cerclage and pessary in preventing preterm birth in twin pregnancies: A systematic review and network meta-analysis. Eur J Obstet Gynecol Reprod Biol. 2021;261:166-177. | Not report study-level data |
| 814 de Grooth HJ, Postema J, Loer SA, et al. Unexplained mortality differences between septic shock trials: a systematic analysis of population characteristics and control-group mortality rates. Intensive Care Med. 2018;44(3):311-322. | Insufficient information for reproducing |

| | |
|---|---|
| 815 Di Lorenzo N, Antoniou SA, Batterham RL, et al. Clinical practice guidelines of the European Association for Endoscopic Surgery (EAES) on bariatric surgery: update 2020 endorsed by IFSO-EC, EASO and ESPCOP. Surg Endosc. 2020;34(6):2332-2358. | Insufficient information for reproducing |
| 816 D'Journo XB, Rolain JM, Doddoli C, et al. Airways colonizations in patients undergoing lung cancer surgery. Eur J Cardiothorac Surg. 2011;40(2):309-319. | Insufficient information for reproducing |
| 817 Domino KB, Anderson EA, Polissar NL, et al. Comparative efficacy and safety of ondansetron, droperidol, and metoclopramide for preventing postoperative nausea and vomiting: a meta-analysis. Anesth Analg. 1999;88(6):1370-1379. | Insufficient information for reproducing |
| 818 Dong P, Tang X, Cheng R,et al. Comparison of the Efficacy of Different Analgesia Treatments for Total Knee Arthroplasty: A Network Meta-Analysis. Clin J Pain. 2018;34(11):1047-1060. | Not report study-level data |
| 819 Edel Y, Avni T, Shepshelovich D, et al. The safety of pulse corticosteroid therapy- Systematic review and meta-analysis. Semin Arthritis Rheum. 2020;50(3):534-545. | Insufficient information for reproducing |
| 820 Elia N, Lysakowski C, Tramèr MR. Does multimodal analgesia with acetaminophen, nonsteroidal antiinflammatory drugs, or selective cyclooxygenase-2 inhibitors and patient-controlled analgesia morphine offer advantages over morphine alone? Meta-analyses of randomized trials. Anesthesiology. 2005;103(6):1296-1304. | Insufficient information for reproducing |
| 821 Elliott WJ. Rationale for a single-pill combination of perindopril arginine and amlodipine besylate. J Am Soc Hypertens. 2015;9(4):257-265. | Insufficient information for reproducing |
| 822 Escalante Y, Saavedra JM, García-Hermoso A, et al. Physical exercise and reduction of pain in adults with lower limb osteoarthritis: a systematic review. J Back Musculoskelet Rehabil. 2010;23(4):175-186. | Insufficient information for reproducing |
| 823 Fan Y, Cao D, Wei Q, et al. The characteristics of circular disposable devices and in situ devices for optimizing male circumcision: a network meta-analysis. Sci Rep. 2016;6:25514. | Not report study-level data |
| 824 Fan Z, Shi H, Luo J, et al. Diagnostic and therapeutic effects of fluorescence cystoscopy and narrow-band imaging in bladder cancer: a systematic review and network meta-analysis. Int J Surg. 2023;109(10):3169-3177. | Not report study-level data |
| 825 Feng F, Zhang Y, Hou J, et al. Can music improve sleep quality in adults with primary insomnia? A systematic review and network meta-analysis. Int J Nurs Stud. 2018;77:189-196. | Not report study-level data |
| 826 Fernández-Rodríguez R, Mesas AE, Garrido-Miguel M, et al. The Relationship of Tree Nuts and Peanuts with Adiposity Parameters: A Systematic Review and Network Meta-Analysis. Nutrients. 2021;13(7):2251. | Not report study-level data |

| | |
|---|---|
| 827 Fitzgerald JP, Fedoruk KA, Jadin SM, et al. Prevention of hypotension after spinal anaesthesia for caesarean section: a systematic review and network meta-analysis of randomised controlled trials. Anaesthesia. 2020;75(1):109-121. | Not report study-level data |
| 828 Fu X, Tang L, Wang C, et al. A Network Meta-Analysis to Compare the Efficacy of Steroid and Antiviral Medications for Facial Paralysis from Bell´s Palsy. Pain Physician. 2018;21(6):559-569. | Not report study-level data |
| 829 Fujii T, Salanti G, Belletti A, et al. Effect of adjunctive vitamin C, glucocorticoids, and vitamin B1 on longer-term mortality in adults with sepsis or septic shock: a systematic review and a component network meta-analysis. Intensive Care Med. 2022;48(1):16-24. | Not report study-level data |
| 830 Habib AS, El-Moalem HE, Gan TJ. The efficacy of the 5-HT3 receptor antagonists combined with droperidol for PONV prophylaxis is similar to their combination with dexamethasone. A meta-analysis of randomized controlled trials. Can J Anaesth. 2004;51(4):311-319. | Insufficient information for reproducing |
| 831 Hagerman A, Schorer R, Putzu A,et al. Cardioprotective Effects of Glucose-Insulin-Potassium Infusion in Patients Undergoing Cardiac Surgery: A Systematic Review and Meta-Analysis. Semin Thorac Cardiovasc Surg. 2024;36(2):167-181. | Insufficient information for reproducing |
| 832 Hajizadeh Maleki B, Tartibian B, Chehrazi M. Effectiveness of Exercise Training on Male Factor Infertility: A Systematic Review and Network Meta-analysis. Sports Health. 2022;14(4):508-517. | Not report study-level data |
| 833 Hao Z, Wang X, Zhang X. Comparing surgical interventions for intertrochanteric hip fracture by blood loss and operation time: a network meta-analysis. J Orthop Surg Res. 2018;13(1):157. | Not report study-level data |
| 834 Hauth JC, Goldenberg RL, Parker CR Jr, et al. Low-dose aspirin: lack of association with an increase in abruptio placentae or perinatal mortality. Obstet Gynecol. 1995;85(6):1055-1058. | Insufficient information for reproducing |
| 835 Henzi I, Walder B, Tramèr MR. Dexamethasone for the prevention of postoperative nausea and vomiting: a quantitative systematic review. Anesth Analg. 2000;90(1):186-194. | Insufficient information for reproducing |
| 836 Henzi I, Walder B, Tramèr MR. Metoclopramide in the prevention of postoperative nausea and vomiting: a quantitative systematic review of randomized, placebo-controlled studies. Br J Anaesth. 1999;83(5):761-771. | Insufficient information for reproducing |
| 837 Hermans MP, Bouenizabila E, Amoussou-Guenou DK, et al. Baseline diabetes as a way to predict CV outcomes in a lipid-modifying trial: a meta-analysis of 330,376 patients from 47 landmark studies. Cardiovasc Diabetol. 2015;14:60. | Insufficient information for reproducing |
| 838 Ho KM, Tan JA. Benefits and risks of maintaining normothermia during cardiopulmonary bypass in adult cardiac surgery: a systematic review. Cardiovasc Ther. 2011;29(4):260-279. | Included but not synthesized/presented |

| | |
|---|---|
| 839 Hou D, Jia Y, Han A, et al. Effect of urinary catheter removal at different times after caesarean section: A systematic review and network meta-analysis. Eur J Obstet Gynecol Reprod Biol. 2023;280:160-167. | Not report study-level data |
| 840 Huang J, Xu Y, Xuan R, et al. A Mixed Comparison of Interventions for Kinesiophobia in Individuals With Musculoskeletal Pain: Systematic Review and Network Meta-Analysis. Front Psychol. 2022;13:886015. | Not report study-level data |
| 841 Jarde A, Lutsiv O, Park CK, et al. Preterm birth prevention in twin pregnancies with progesterone, pessary, or cerclage: a systematic review and meta-analysis. BJOG. 2017;124(8):1163-1173. | Not report study-level data |
| 842 Jian Z, Wei X, Ye D, et al. Pharmacotherapy of premature ejaculation: a systematic review and network meta-analysis. Int Urol Nephrol. 2018;50(11):1939-1948. | Not report study-level data |
| 843 Kabboul NN, Tomlinson G, Francis TA, et al. Comparative Effectiveness of the Core Components of Cardiac Rehabilitation on Mortality and Morbidity: A Systematic Review and Network Meta-Analysis. J Clin Med. 2018;7(12):514. | Not report study-level data |
| 844 Khaing W, Vallibhakara SA, Tantrakul V, et al. Calcium and Vitamin D Supplementation for Prevention of Preeclampsia: A Systematic Review and Network Meta-Analysis. Nutrients. 2017;9(10):1141. | Insufficient information for reproducing |
| 845 Kim DH, Lee J, Kim SW, et al. The Efficacy of Hypotensive Agents on Intraoperative Bleeding and Recovery Following General Anesthesia for Nasal Surgery: A Network Meta-Analysis. Clin Exp Otorhinolaryngol. 2021;14(2):200-209. | Not report study-level data |
| 846 Kong QF, Jiao JB, Chen QQ, et al. Comparative effectiveness of radiofrequency ablation with or without transarterial chemoembolization for hepatocellular carcinoma. Tumor Biology, 2014, 35(3): 2655-2659. | Not report study-level data |
| 847 Labarca G, Montenegro R, Oscullo G, et al. Placebo response in objective and subjective measures of hypersomnia in randomized clinical trials on obstructive sleep apnea. A systematic review and meta-analysis. Sleep Med Rev. 2023;67:101720. | Insufficient information for reproducing |
| 848 Laupacis A, Fergusson D. Drugs to minimize perioperative blood loss in cardiac surgery: meta-analyses using perioperative blood transfusion as the outcome. The International Study of Peri-operative Transfusion (ISPOT) Investigators. Anesth Analg. 1997;85(6):1258-1267. | Insufficient information for reproducing |
| 849 Lee JM, Cho YJ, Ahn EJ, et al. Pharmacological strategies to prevent postoperative delirium: a systematic review and network meta-analysis. Anesth Pain Med (Seoul). 2021;16(1):28-48. | Not report study-level data |
| 850 Leslie JB, Gan TJ. Meta-analysis of the safety of 5-HT3 antagonists with dexamethasone or droperidol for prevention of PONV. Ann Pharmacother. 2006;40(5):856-872. | Insufficient information for reproducing |
| 851 Li H, Yang L, Song Y, et al. Comparative effectiveness of different treatment modalities for active, moderate-to-severe Graves' orbitopathy: a systematic review and network meta-analysis. Acta Ophthalmol. 2022;100(6):e1189-e1198. | Not report study-level data |

| | |
|---|---|
| 852 Lin J, Liao Y, Gong C, et al. Regional Analgesia in Video-Assisted Thoracic Surgery: A Bayesian Network Meta-Analysis. Front Med (Lausanne). 2022;9:842332. | Not report study-level data |
| 853 Liu H, Zhang M, Huang M, et al. Comparative efficacy and safety of drug treatment for premature ejaculation: A systemic review and Bayesian network meta-analysis. Andrologia. 2020;52(11):e13806. | Not report study-level data |
| 854 Liu T, Li W, Zhou H, et al. Verifying the Relative Efficacy between Continuous Positive Airway Pressure Therapy and Its Alternatives for Obstructive Sleep Apnea: A Network Meta-analysis. Front Neurol. 2017;8:289. | Not report study-level data |
| 855 Liu X, Qiu Y, Yu ED, et al. Comparison of therapeutic interventions for recurrent pregnancy loss in association with antiphospholipid syndrome: A systematic review and network meta-analysis. Am J Reprod Immunol. 2020;83(4):e13219. | Not report study-level data |
| 856 Lock CA, Lecouturier J, Mason JM, et al. Lifestyle interventions to prevent osteoporotic fractures: a systematic review. Osteoporos Int. 2006;17(1):20-28. | Insufficient information for reproducing |
| 857 Long YX, Sun Y, Liu RZ, et al. Immune-Related Pneumonitis Was Decreased by Addition of Chemotherapy with PD-1/L1 Inhibitors: Systematic Review and Network Meta-Analysis of Randomized Controlled Trials (RCTs). Curr Oncol. 2022;29(1):267-282. | Not report study-level data |
| 858 Lu J, Ying Z, Wang P, et al. Effects of continuous glucose monitoring on glycaemic control in type 2 diabetes: A systematic review and network meta-analysis of randomized controlled trials. Diabetes Obes Metab. 2024;26(1):362-372. | Not report study-level data |
| 859 Luan S, Zhou B, Wu Q, et al. Brain-derived neurotrophic factor blood levels after electroconvulsive therapy in patients with major depressive disorder: A systematic review and meta-analysis. Asian J Psychiatr. 2020;51:101983. | Insufficient information for reproducing |
| 860 Lv S, Yu J, Xu X. A comparison of effectiveness among frequent treatments of recurrent spontaneous abortion: A Bayesian network meta-analysis. Am J Reprod Immunol. 2018;80(1):e12856. | Not report study-level data |
| 861 Machado A, Matos Silva P, Afreixo V,et al. Design of pulmonary rehabilitation programmes during acute exacerbations of COPD: a systematic review and network meta-analysis.in Eur Respir Rev. 2021;30(159):215039. | Not report study-level data |
| 862 MacLean C, Newberry S, Maglione M, et al. Systematic review: comparative effectiveness of treatments to prevent fractures in men and women with low bone density or osteoporosis. Ann Intern Med. 2008;148(3):197-213. | Insufficient information for reproducing |
| 863 Mearns ES, Sobieraj DM, White CM, et al. Comparative efficacy and safety of antidiabetic drug regimens added to metformin monotherapy in patients with type 2 diabetes: a network meta-analysis. PLoS One. 2015;10(4):e0125879. | Not report study-level data |
| 864 Meco M, Giustiniano E, Cecconi M, et al. Pharmacological prevention of postoperative delirium in patients undergoing cardiac surgery: a bayesian network meta-analysis. J Anesth. 2023;37(2):294-310. | Not report study-level data |

| | |
|---|---|
| 865 Messerli F. Renin Angiotensin System Inhibitors for Patients with Coronary Artery Disease and Preserved Left Ventricular Function Insights from a Systematic Review and Meta-Analysis of Randomized Trials. | Insufficient information for reproducing |
| 866 Migliorini F, Maffulli N, Colarossi G, et al. Effect of drugs on bone mineral density in postmenopausal osteoporosis: a Bayesian network meta-analysis. J Orthop Surg Res. 2021;16(1):533. | Not report study-level data |
| 867 Mo M, Wang S, Chen Z, et al. A systematic review and meta-analysis of the response of serum 25-hydroxyvitamin D concentration to vitamin D supplementation from RCTs from around the globe. Eur J Clin Nutr. 2019;73(6):816-834. | Not report study-level data |
| 868 Montesi SB, Edwards BA, Malhotra A, et al. The effect of continuous positive airway pressure treatment on blood pressure: a systematic review and meta-analysis of randomized controlled trials. J Clin Sleep Med. 2012;8(5):587-596. | Insufficient information for reproducing |
| 869 Murad MH, Drake MT, Mullan RJ, et al. Clinical review. Comparative effectiveness of drug treatments to prevent fragility fractures: a systematic review and network meta-analysis. J Clin Endocrinol Metab. 2012;97(6):1871-1880. | Not report study-level data |
| 870 Orr L, Reisinger-Kindle K, Roy A, et al. Combination of Foley and prostaglandins versus Foley and oxytocin for cervical ripening: a network meta-analysis. Am J Obstet Gynecol. 2020;223(5):743.e1-743.e17. | Not report study-level data |
| 871 Park SK, Lim T, Cho H, et al. Comparative effectiveness of pharmacological interventions to prevent postoperative delirium: a network meta-analysis. Sci Rep. 2021;11(1):11922. | Not report study-level data |
| 872 Park SM, Mangat HS, Berger K, et al. Efficacy spectrum of antishivering medications: meta-analysis of randomized controlled trials. Crit Care Med. 2012;40(11):3070-3082. | Insufficient information for reproducing |
| 873 Pei H, Qu J, Chen JM, et al. The effects of antioxidant supplementation on short-term mortality in sepsis patients. Heliyon. 2024;10(8):e29156. | Not report study-level data |
| 874 Probst P, Ohmann S, Klaiber U, et al. Meta-analysis of immunonutrition in major abdominal surgery. Br J Surg. 2017;104(12):1594-1608. | Insufficient information for reproducing |
| 875 Ravidà A, Serroni M, Borgnakke WS, et al. Short (≤6 mm) compared with ≥10-mm dental implants in different clinical scenarios: A systematic review of randomized clinical trials with meta-analysis, trial sequential analysis and quality of evidence grading. J Clin Periodontol. 2024;51(7):936-965. | Insufficient information for reproducing |
| 876 Remonti LR, Dias S, Leitão CB, et al. Classes of antihypertensive agents and mortality in hypertensive patients with type 2 diabetes-Network meta-analysis of randomized trials. J Diabetes Complications. 2016 Aug;30(6):1192-200. | Not report study-level data |

| | |
|---|---|
| 877 Richy F, Ethgen O, Bruyere O, et al. Efficacy of alphacalcidol and calcitriol in primary and corticosteroid-induced osteoporosis: a meta-analysis of their effects on bone mineral density and fracture rate. Osteoporos Int. 2004;15(4):301-310. | Insufficient information for reproducing |
| 878 Rogozińska E, Daru J, Nicolaides M, et al. Iron preparations for women of reproductive age with iron deficiency anaemia in pregnancy (FRIDA): a systematic review and network meta-analysis. Lancet Haematol. 2021;8(7):e503-e512. | Not report study-level data |
| 879 Rømsing J, Møiniche S, Ostergaard D, et al. Local infiltration with NSAIDs for postoperative analgesia: evidence for a peripheral analgesic action. Acta Anaesthesiol Scand. 2000;44(6):672-683. | Insufficient information for reproducing |
| 880 Sahebari M, Nabavi N, Salehi M. Correlation between serum 25(OH)D values and lupus disease activity: an original article and a systematic review with meta-analysis focusing on serum VitD confounders. Lupus. 2014;23(11):1164-1177. | Insufficient information for reproducing |
| 881 Samy A, Abbas AM, Mahmoud M, et al. Evaluating different pain lowering medications during intrauterine device insertion: a systematic review and network meta-analysis. Fertil Steril. 2019;111(3):553-561.e4. | Not report study-level data |
| 882 Samy A, Raslan AN, Talaat B, et al. Perioperative nonhormonal pharmacological interventions for bleeding reduction during open and minimally invasive myomectomy: a systematic review and network meta-analysis. Fertil Steril. 2020;113(1):224-233.e6. | Not report study-level data |
| 883 Sandner S, Redfors B, Angiolillo DJ, et al. Association of Dual Antiplatelet Therapy With Ticagrelor With Vein Graft Failure After Coronary Artery Bypass Graft Surgery: A Systematic Review and Meta-analysis. JAMA. 2022;328(6):554-562. | Insufficient information for reproducing |
| 884 Sathianathen NJ, Hwang EC, Mian R, et al. Selective Serotonin Re-Uptake Inhibitors for Premature Ejaculation in Adult Men: A Cochrane Systematic Review. World J Mens Health. 2022;40(2):257-263. | Insufficient information for reproducing |
| 885 Schouten ES, van de Pol AC, Schouten AN, et al. The effect of aprotinin, tranexamic acid, and aminocaproic acid on blood loss and use of blood products in major pediatric surgery: a meta-analysis. Pediatr Crit Care Med. 2009;10(2):182-190. | Insufficient information for reproducing |
| 886 Schubert AK, Seneviratne V, Stolz J, et al. The effect of adjuvants added to local anaesthetics for single-injection upper extremity peripheral regional anaesthesia: A systematic review with network meta-analysis of randomised trials. Eur J Anaesthesiol. 2023;40(9):672-690. | Not report study-level data |
| 887 Schütze R, Rees C, Smith A, et al. How Can We Best Reduce Pain Catastrophizing in Adults With Chronic Noncancer Pain? A Systematic Review and Meta-Analysis. J Pain. 2018;19(3):233-256. | Insufficient information for reproducing |

| | |
|---|---|
| 888 Sciarretta S, Palano F, Tocci G, Baldini R, Volpe M. Antihypertensive treatment and development of heart failure in hypertension: a Bayesian network meta-analysis of studies in patients with hypertension and high cardiovascular risk. Arch Intern Med. 2011;171(5):384-394. | Not report study-level data |
| 889 Sehmbi H, Brull R, Ceballos KR, et al. Perineural and intravenous dexamethasone and dexmedetomidine: network meta-analysis of adjunctive effects on supraclavicular brachial plexus block. Anaesthesia. 2021;76(7):974-990. | Not report study-level data |
| 890 Selvarajan S, Anandaradje A, Shivabasappa S, et al. Efficacy of pharmacological interventions in COVID-19: A network meta-analysis. Br J Clin Pharmacol. 2022;88(9):4080-4091. | Not report study-level data |
| 891 Shah AA, Donovan K, Seeley C, et al. Risk of Infection Associated With Administration of Intravenous Iron: A Systematic Review and Meta-analysis. JAMA Netw Open. 2021;4(11):e2133935. | Not report study-level data |
| 892 Shang L, Hou M, Guo F. Postoperative Application of Dexmedetomidine is the Optimal Strategy to Reduce the Incidence of Postoperative Delirium After Cardiac Surgery: A Network Meta-Analysis of Randomized Controlled Trials. Ann Pharmacother. 2023;57(3):221-231. | Not report study-level data |
| 893 Shunan F, Jiqing Y, Xue D. Effects of angiotensin-converting enzyme inhibitors and angiotensin receptor blockers on cardiovascular events in patients with diabetes and overt nephropathy: a meta-analysis of randomised controlled trials. J Renin Angiotensin Aldosterone Syst. 2018;19(4):1470320318803495. | Insufficient information for reproducing |
| 894 Song P, Wen Y, Huang C, et al. The efficacy and safety comparison of surgical treatments for stress urinary incontinence: A network meta-analysis. Neurourol Urodyn. 2018;37(4):1199-1211. | Not report study-level data |
| 895 Sridharan K, Sivaramakrishnan G. Drugs for preventing post-operative nausea and vomiting in patients undergoing laparoscopic cholecystectomy: Network meta-analysis of randomized clinical trials and trial sequential analysis. Int J Surg. 2019;69:1-12. | Not report study-level data |
| 896 Stephen AI, Avenell A. A systematic review of multivitamin and multimineral supplementation for infection. J Hum Nutr Diet. 2006;19(3):179-190. | Insufficient information for reproducing |
| 897 Stockler M, Wilcken NR, Ghersi D, et al. Systematic reviews of chemotherapy and endocrine therapy in metastatic breast cancer. Cancer Treat Rev. 2000;26(3):151-168. | Insufficient information for reproducing |
| 898 Stockton KA, Mengersen K, Paratz JD, et al. Effect of vitamin D supplementation on muscle strength: a systematic review and meta-analysis. Osteoporos Int. 2011;22(3):859-871. | Insufficient information for reproducing |
| 899 Stubbs B, Vancampfort D, Rosenbaum S, et al. Dropout from exercise randomized controlled trials among people with depression: A meta-analysis and meta regression. J Affect Disord. 2016;190:457-466. | Insufficient information for reproducing |

| | |
|---|---|
| 900 Sung VW, Schleinitz MD, Rardin CR,et al. Comparison of retropubic vs transobturator approach to midurethral slings: a systematic review and meta-analysis. Am J Obstet Gynecol. 2007;197(1):3-11. | Insufficient information for reproducing |
| 901 Tang DH, Malone DC. A network meta-analysis on the efficacy of serotonin type 3 receptor antagonists used in adults during the first 24 hours for postoperative nausea and vomiting prophylaxis. Clin Ther. 2012;34(2):282-294. | Not report study-level data |
| 902 Tanner-Smith EE, Wilson SJ, Lipsey MW. The comparative effectiveness of outpatient treatment for adolescent substance abuse: a meta-analysis. J Subst Abuse Treat. 2013;44(2):145-158. | Insufficient information for reproducing |
| 903 Taylor MJ, Rudkin L, Bullemor-Day P, et al. Strategies for managing sexual dysfunction induced by antidepressant medication. Cochrane Database Syst Rev. 2013;(5):CD003382. | Insufficient information for reproducing |
| 904 Thomopoulos C, Parati G, Zanchetti A. Effects of blood pressure-lowering on outcome incidence in hypertension: 5. Head-to-head comparisons of various classes of antihypertensive drugs - overview and meta-analyses. J Hypertens. 2015;33(7):1321-1341. | Insufficient information for reproducing |
| 905 Thomopoulos C, Parati G, Zanchetti A. Effects of blood pressure-lowering treatment. 6. Prevention of heart failure and new-onset heart failure--meta-analyses of randomized trials. J Hypertens. 2016;34(3):373-384. | Insufficient information for reproducing |
| 906 Thomopoulos C, Parati G, Zanchetti A. Effects of blood-pressure-lowering treatment in hypertension: 9. Discontinuations for adverse events attributed to different classes of antihypertensive drugs: meta-analyses of randomized trials. J Hypertens. 2016;34(10):1921-1932. | Insufficient information for reproducing |
| 907 Tian G, Ye Z, Zhao Q, et al. Complication incidence of EUS-guided pancreas biopsy: A systematic review and meta-analysis of 11 thousand population from 78 cohort studies. Asian J Surg. 2020;43(11):1049-1055. | Insufficient information for reproducing |
| 908 Trigo-Vicente C, Gimeno-Ballester V, García-López S, et al. Systematic review and network meta-analysis of treatment for moderate-to-severe ulcerative colitis. Int J Clin Pharm. 2018;40(6):1411-1419. | Not report study-level data |
| 909 Turrini G, Tedeschi F, Cuijpers P, et al. A network meta-analysis of psychosocial interventions for refugees and asylum seekers with PTSD. BMJ Glob Health. 2021;6(6):e005029. | Not report study-level data |
| 910 van Hout D, Plantinga NL, Bruijning-Verhagen PC, et al. Cost-effectiveness of selective digestive decontamination (SDD) versus selective oropharyngeal decontamination (SOD) in intensive care units with low levels of antimicrobial resistance: an individual patient data meta-analysis. BMJ Open. 2019;9(9):e028876. | Insufficient information for reproducing |

| | |
|---|---|
| 911 Vincent JL, Navickis RJ, Wilkes MM. Morbidity in hospitalized patients receiving human albumin: a meta-analysis of randomized, controlled trials. Crit Care Med. 2004;32(10):2029-2038. | Insufficient information for reproducing |
| 912 Wang G, Sui L, Gai P, et al. The efficacy and safety of vertebral fracture prevention therapies in post-menopausal osteoporosis treatment: Which therapies work best? a network meta-analysis. Bone Joint Res. 2017;6(7):452-463. | Not report study-level data |
| 913 Wang H, Liu J, Fang K, et al. Transobturator tape, tension-free vaginal tape, and transvaginal tension-free vaginal tape-obturator for the treatment of female stress urinary incontinence: A systematic review and network meta-analysis. Int J Gynaecol Obstet. 2022;157(3):527-535. | Not report study-level data |
| 914 Wang R, Kim BV, van Wely M, et al. Treatment strategies for women with WHO group II anovulation: systematic review and network meta-analysis. BMJ. 2017;356:j138. | Not report study-level data |
| 915 Wang XC, Zhang D, Yang ZX, et al. Mesh reinforcement for the prevention of incisional hernia formation: a systematic review and meta-analysis of randomized controlled trials. J Surg Res. 2017;209:17-29. | Insufficient information for reproducing |
| 916 Wang Y, Xu L, Meng X, et al. Different Chemotherapy Regimens in the Management of Advanced or Metastatic Urothelial Cancer: a Bayesian Network Meta-Analysis of Randomized Controlled Trials. Cell Physiol Biochem. 2018;50(1):1-14. | Not report study-level data |
| 917 Welte T, Ambrose LJ, Sibbring GC, et al. Current evidence for COVID-19 therapies: a systematic literature review. Eur Respir Rev. 2021;30(159):200384. | Insufficient information for reproducing |
| 918 Weng Y, Zhang J, Chen Z. Effect of non-pharmacological interventions on pain in preterm infants in the neonatal intensive care unit: a network meta-analysis of randomized controlled trials. BMC Pediatr. 2024;24(1):9. | Not report study-level data |
| 919 Whegang Youdom S, Chiabi A, Basco LK. Monitoring the Efficacy and Safety of Artemisinin-Based Combination Therapies: A Review and Network Meta-analysis of Antimalarial Therapeutic Efficacy Trials in Cameroon. Drugs R D. 2019;19(1):1-14. | Not report study-level data |
| 920 Willeit P, Tschiderer L, Allara E, et al. Carotid Intima-Media Thickness Progression as Surrogate Marker for Cardiovascular Risk: Meta-Analysis of 119 Clinical Trials Involving 100 667 Patients. Circulation. 2020;142(7):621-642. | Insufficient information for reproducing |
| 921 Xie X, Liu Y, Perkovic V, et al. Renin-Angiotensin System Inhibitors and Kidney and Cardiovascular Outcomes in Patients With CKD: A Bayesian Network Meta-analysis of Randomized Clinical Trials. Am J Kidney Dis. 2016;67(5):728-741. | Not report study-level data |
| 922 Xu H, Liu Y, Sezgin EA, et al. Comparative effectiveness research on proximal femoral nail versus dynamic hip screw in patients with trochanteric fractures: a systematic review and meta-analysis of randomized trials. J Orthop Surg Res. 2022;17(1):292. | Not report study-level data |
| 923 Xu M, Hu J, Yan J, et al. Paravertebral Block versus Thoracic Epidural Analgesia for Postthoracotomy Pain Relief: A Meta-Analysis of Randomized Trials. Thorac Cardiovasc Surg. 2022;70(5):413-421. | Insufficient information for reproducing |

| | |
|---|---|
| 924 Xu T, Zhan M, Jiang X. Efficacy and safety of vildagliptin combined with metformin in the treatment of type 2 diabetes mellitus: a systematic review and meta-analysis. Int J Clin Exp Med, 2016, 9(6): 9495-9503. | Insufficient information for reproducing |
| 925 Yang K, Zeng J, Dai W,et al. A systematic review and Bayesian network meta-analysis for comparative safety assessment of favipiravir interventions in hospitalized COVID-19 patients. J Infect Dev Ctries. 2022;16(9):1406-1412. | Not report study-level data |
| 926 Yang L, Kang N, Yang JC, et al. Drug efficacies on bone mineral density and fracture rate for the treatment of postmenopausal osteoporosis: a network meta-analysis. Eur Rev Med Pharmacol Sci. 2019;23(6):2640-2668. | Not report study-level data |
| 927 Yu L, Zhu Y, Geng L, et al. Effect of different nutrients on blood glucose, inflammatory response and oxidative stress in gestational diabetes mellitus: a network meta-analysis. Br J Nutr. 2024;131(9):1513-1527. | Not report study-level data |
| 928 Zhang C, Jin H, Wen YF, et al. Efficacy of COVID-19 Treatments: A Bayesian Network Meta-Analysis of Randomized Controlled Trials. Front Public Health. 2021;9:729559. | Not report study-level data |
| 929 Zhang T, Ye X, Zhu T, et al. Antithrombotic Treatment for Recurrent Miscarriage: Bayesian Network Meta-Analysis and Systematic Review. Medicine (Baltimore). 2015;94(45):e1732. | Not report study-level data |
| 930 Zhang X, Zhou D, Song S,et al. Efficacy and Safety of Long-Term Dual Antiplatelet Therapy: A Systematic Review and Meta-Analysis. Clin Appl Thromb Hemost. 2024;30:10760296241244772. | Insufficient information for reproducing |
| 931 Zhao J, Zhang H, Wei L, Xie S, Suo Z. Comparing the long-term efficacy of standard and combined minimally invasive procedures for unresectable HCC: a mixed treatment comparison. Oncotarget. 2017;8(9):15101-15113. | Not report study-level data |
| 932 Zhao JL, Zeng LF, Pan JK, et al. Comparisons of the Efficacy and Safety of Total Knee Arthroplasty by Different Surgical Approaches: A Systematic Review and Network Meta-analysis. Orthop Surg. 2022;14(3):472-485. | Not report study-level data |
| 933 Zhou J, Wang T, Zhao X, et al. Comparative Efficacy of Bisphosphonates to Prevent Fracture in Men with Osteoporosis: A Systematic Review with Network Meta-Analyses. Rheumatol Ther. 2016;3(1):117-128. | Not report study-level data |
| 934 Zhou X, Zhou D, Wang J, et al. Treatment strategies for Graves' ophthalmopathy: a network meta-analysis. Br J Ophthalmol. 2020;104(4):551-556. | Not report study-level data |
| 935 Xinyu X, Xintong T, Youping L, et al. Motherwort Injection for Preventing Uterine Hemorrhage in Women With Induced Abortion: A Systematic Review and Meta-Analysis of Randomized Evidence. Frontiers in Pharmacology, 2022, 13: 916665. | Not included for SR |
| 936 Liang F, Zhou Y, Zhang Z, et al. Association of vitamin D in individuals with periodontitis: an updated systematic review and meta-analysis. BMC Oral Health, 2023, 23(1): 387. | Not included for SR |
| 937 Pagali S R, Kumar R, LeMahieu A M, et al. Efficacy and safety of transcranial magnetic stimulation on cognition in mild cognitive impairment, Alzheimer’s disease, Alzheimer’s disease-related dementias, and other cognitive disorders: a systematic review and meta-analysis. International psychogeriatrics, 2024: 1-49. | Included but not synthesized/presented |

| | |
|---|---|
| 938 Desborough M, Sandu R, Brunskill SJ,et al. Fresh frozen plasma for cardiovascular surgery. Cochrane Database Syst Rev.2015(7):CD007614. | Not included for SR |
| 939 Mahoney C B. Platelet-rich plasmapheresis: a meta-analysis of clinical outcomes and costs. The Journal of ExtraCorporeal Technology, 1998, 30(1): 10-19. | Not report study-level data |
| 940 Loro A, Borg M B, Battaglia M, et al. Balance rehabilitation through robot-assisted gait training in post-stroke patients: a systematic review and meta-analysis. Brain sciences, 2023, 13(1): 92. | Not included for SR |
| 941 Xie Y, Yang Y, Jiang H, et al. Brain-machine interface-based training for improving upper extremity function after stroke: A meta-analysis of randomized controlled trials. Frontiers in Neuroscience, 2022, 16: 949575. | Not included for SR |
| 942 Salaminia S, Sayehmiri F, Angha P, et al. Evaluating the effect of magnesium supplementation and cardiac arrhythmias after acute coronary syndrome: a systematic review and meta-analysis. BMC Cardiovascular Disorders, 2018, 18: 1-10. | Included but not synthesized/presented |
| 943 Li D, Huang L T, Zhang F, et al. Comparative effectiveness of ehealth self-management interventions for patients with heart failure: A Bayesian network meta-analysis. Patient Education and Counseling, 2024, 124: 108277. | Not report study-level data |
| 944 Yu Y, Fang L, Zhang R, et al. Comparative effectiveness of 9 ovulation-induction therapies in patients with clomiphene citrate-resistant polycystic ovary syndrome: a network meta-analysis. Scientific reports, 2017, 7(1): 3812. | Not report study-level data |
| 945 Xue T, Li S W, Wang Y. Effectiveness of bromocriptine monotherapy or combination treatment with clomiphene for infertility in women with galactorrhea and normal prolactin: A systematic review and meta-analysis. Current therapeutic research, 2010, 71(4): 199-210. | Not included for SR |
| 946 Abdel-Maboud M, Menshawy A, Hasabo E A, et al. The comparative effectiveness of 55 interventions in obese patients with polycystic ovary syndrome: a network meta-analysis of 101 randomized trials. PloS one, 2021, 16(7): e0254412. | Not report study-level data |
| 947 Gao Z, Fang L, Yin P, et al. Effects of Nursing Care for the Treatment of Patients with Bladder Cancer: A Systematic Review and Meta-analysis. Comput Math Methods Med.2022:9554223. | Not included for SR |
| 948 Shimada K, Inokuchi R, Ohigashi T, et al. Artificial intelligence-assisted interventions for perioperative anesthetic management: a systematic review and meta-analysis. BMC anesthesiology, 2024, 24(1): 306. | Not included for SR |
| 949 Glazachev O S, Kryzhanovskaya S Y, Zapara M A, et al. Safety and efficacy of intermittent hypoxia conditioning as a new rehabilitation/secondary prevention strategy for patients with cardiovascular diseases: a systematic review and meta-analysis. Current cardiology reviews, 2021, 17(6): 88-99. | Not included for SR |
| 950 Stewart L A, Simmonds M, Duley L, et al. Evaluating Progestogens for Preventing Preterm birth International Collaborative (EPPPIC): meta-analysis of individual participant data from randomised controlled trials. The Lancet, 2021, 397(10280): 1183-1194. | Included but not synthesized/presented |

| | |
|---|---|
| 951 Boelig R C, Locci M, Saccone G, et al. Vaginal progesterone compared with intramuscular 17-alpha-hydroxyprogesterone caproate for prevention of recurrent preterm birth in singleton gestations: a systematic review and meta-analysis. American Journal of Obstetrics & Gynecology MFM, 2022, 4(5): 100658. | Not included for SR |
| 952 Saccone G, Khalifeh A, Elimian A, et al. Vaginal progesterone vs intramuscular 17α-hydroxyprogesterone caproate for prevention of recurrent spontaneous preterm birth in singleton gestations: systematic review and meta-analysis of randomized controlled trials. Ultrasound in Obstetrics & Gynecology, 2017, 49(3): 315-321. | Not included for SR |
| 953 Jarde A, Lutsiv O, Park C K, et al. Effectiveness of progesterone, cerclage and pessary for preventing preterm birth in singleton pregnancies: a systematic review and network meta-analysis. BJOG: An International Journal of Obstetrics & Gynaecology, 2017, 124(8): 1176-1189. | Not report study-level data |
| 954 Szabó A, Váncsa S, Hegyi P, et al. Lifestyle-, environmental-, and additional health factors associated with an increased sperm DNA fragmentation: a systematic review and meta-analysis. Reproductive Biology and Endocrinology, 2023, 21(1): 5. | Not included for SR |
| 955 Boedt T, Vanhove AC, Vercoe MA, et al. Preconception lifestyle advice for people with infertility. Cochrane Database Syst Rev. 2021(4):CD008189. | Not included for SR |
| 956 Hajizadeh Maleki B, Tartibian B, Chehrazi M. Effectiveness of exercise training on male factor infertility: a systematic review and network meta-analysis. Sports Health, 2022, 14(4): 508-517. | Not report study-level data |
| 957 Chen Z, Hong Z, Wang S, et al. Effectiveness of non-pharmaceutical intervention on sperm quality: a systematic review and network meta-analysis. Aging (Albany NY), 2023, 15(10): 4253. | Not included for SR |
| 958 Knapik M, Żelazo D A, Osowiecka K, et al. Efficacy of Anti-Interleukin-1 Therapeutics in the Treatment of Knee Osteoarthritis: A Systematic Review and Meta-Analysis of Randomized Controlled Trials from the Years 2000 to 2023. Journal of Clinical Medicine, 2024, 13(10): 2859. | Not included for SR |
| 959 Zhang W, Nuki G, Moskowitz R W, et al. OARSI recommendations for the management of hip and knee osteoarthritis: part III: Changes in evidence following systematic cumulative update of research published through January 2009. Osteoarthritis and cartilage, 2010, 18(4): 476-499. | clinical guideline |
| 960 Sa M P, Jacquemyn X, Van den Eynde J, et al. Midterm outcomes of endovascular versus medical therapy for uncomplicated type B aortic dissection: meta-analysis of reconstructed time to event data. European Journal of Vascular and Endovascular Surgery, 2023. | Not report study-level data |
| 961 Wang Y, Shen Q, Wang C. Efficacy of Rapid Rehabilitation Nursing in Postoperative Care in China: A Meta-Analysis. Rehabilitation Nursing Journal, 2023, 48(5): 170-179. | Not included for SR |
| 962 Lv X, Zhou M, Liu X, et al. Efficacy and Safety of Zhenwu Decoction in the Treatment of Diabetic Nephropathy: A Systematic Review and Meta-Analysis. Evidence-Based Complementary and Alternative Medicine, 2022, 2022(1): 2133705. | Not included for SR |

| | |
|---|---|
| 963 Gill G S, Gadre A, Thandra A, et al. Comparative efficacy and safety of adenosine and regadenoson for assessment of fractional flow reserve: A systematic review and meta-analysis. Journal of the American College of Cardiology, 2022, 79(9_Supplement): 673-673. | Not included for SR |
| 964 Liao D, Liu X, Yuan X, et al. Clinical evidence of the effects of carnitine supplementation on body weight, glycemic control and serum lipids in women with polycystic ovary syndrome: a systematic review and meta-analysis. Gynecological Endocrinology, 2022, 38(2): 110-115. | Not report study-level data |
| 965 Gong Y, Jiang T, He H, et al. Effects of carnitine on glucose and lipid metabolic profiles and fertility outcomes in women with polycystic ovary syndrome: A systematic review and meta-analysis. Clinical Endocrinology, 2023, 98(5): 682-691. | Not included for SR |
| 966 Dehkordi S R, Malekahmadi M, Nikbaf-Shandiz M, et al. The effects of L-carnitine supplementation on lipid profiles in adults: A systematic review and dose-response meta-analysis. PharmaNutrition, 2024, 27: 100374. | Not included for SR |
| 967 Abdel-Maboud M, Menshawy A, Hasabo E A, et al. The comparative effectiveness of 55 interventions in obese patients with polycystic ovary syndrome: a network meta-analysis of 101 randomized trials. PloS one, 2021, 16(7): e0254412. | Not report study-level data |
| 968 Chen Z, Hong Z, Wang S, et al. Effectiveness of non-pharmaceutical intervention on sperm quality: a systematic review and network meta-analysis. Aging (Albany NY), 2023, 15(10): 4253. | Not included for SR |
| 969 Vrontaras N, Koulierakis G, Ntourou I, et al. Psychosocial interventions on the posttraumatic growth of adults with cancer: A systematic review and meta-analysis of clinical trials. Psycho-Oncology, 2023, 32(12): 1798-1826. | Included but not synthesized/presented |
| 970 Tan L, Fang P, Cui J, et al. Effects of progressive muscle relaxation on health-related outcomes in cancer patients: A systematic review and meta-analysis of randomized controlled trials. Complementary Therapies in Clinical Practice, 2022, 49: 101676. | Included but not synthesized/presented |
| 971 Zhou Z, Cui Y, Zhang X, et al. The role of N-acetyl-cysteine (NAC) orally daily on the sperm parameters and serum hormones in idiopathic infertile men: A systematic review and meta-analysis of randomised controlled trials. Andrologia, 2021, 53(2): e13953. | Not included for SR |
| 972 Wang Y, Li Y, Liang J, et al. Chemotherapy-induced amenorrhea and its prognostic significance in premenopausal women with breast cancer: An updated meta-analysis. Frontiers in Oncology, 2022, 12: 859974. | Not included for SR |
| 973 Lambertini M, Moore H C F, Leonard R C F, et al. Gonadotropin-releasing hormone agonists during chemotherapy for preservation of ovarian function and fertility in premenopausal patients with early breast cancer: a systematic review and meta-analysis of individual patient–level data. Journal of clinical oncology, 2018, 36(19): 1981-1990. | Included but not synthesized/presented |
| 974 Vitek W S, Shayne M, Hoeger K, et al. Gonadotropin-releasing hormone agonists for the preservation of ovarian function among women with breast cancer who did not use tamoxifen after chemotherapy: a systematic review and meta-analysis. Fertility and sterility, 2014, 102(3): 808-815. | Not included for SR |

| | |
|---|---|
| 975 Zhang Y, Xiao Z, Wang Y, et al. Gonadotropin-releasing hormone for preservation of ovarian function during chemotherapy in lymphoma patients of reproductive age: a summary based on 434 patients. PLoS One, 2013, 8(11): e80444. | Not included for SR |
| 976 Hickman L C, Llarena N C, Valentine L N, et al. Preservation of gonadal function in women undergoing chemotherapy: a systematic review and meta-analysis of the potential role for gonadotropin-releasing hormone agonists. Journal of Assisted Reproduction and Genetics, 2018, 35: 571-581. | Not included for SR |
| 977 Elgindy E, Sibai H, Abdelghani A, et al. Protecting ovaries during chemotherapy through gonad suppression: a systematic review and meta-analysis. Obstetrics & Gynecology, 2015, 126(1): 187-195. | Not included for SR |
| 978 van Zuuren EJ, Fedorowicz Z, Carter B, et al. Interventions for hirsutism (excluding laser and photoepilation therapy alone). Cochrane Database Syst Rev.2015(4):CD010334. | Not included for SR |
| 979 Van Zuuren E J, Fedorowicz Z. Interventions for hirsutism excluding laser and photoepilation therapy alone: abridged Cochrane systematic review including GRADE assessments. British Journal of Dermatology, 2016, 175(1): 45-61. | Included but not synthesized/presented |
| 980 Zhang J, Zhou K, Luo X, et al. Variation of laparoscopic ovarian drilling for clomiphene citrate-resistant patients with polycystic ovary syndrome and infertility: A meta-analysis. Journal of Minimally Invasive Gynecology, 2020, 27(5): 1048-1058. | Not report study-level data |
| 981 Ma X H, Chen Y, Huang X Y, et al. Characteristics and Efficacy of Traditional Chinese Medicine in the Therapeutic Strategy of Chronic Coronary Syndrome: a systematic review and meta-analysis. Phytomedicine, 2024: 155579. | Not included for SR |
| 982 Kanakaraj M, Bhat AD, Singh NP, et al. Choice of supraglottic airway devices: a network meta-analysis of randomised controlled trials. Br J Anaesth. 2024;133(6):1284-1306. | Not included for SR |
| 983 Brown J, Farquhar C. Clomiphene and other antioestrogens for ovulation induction in polycystic ovarian syndrome. Cochrane Database Syst Rev. 2016(12):CD002249. | Not included for SR |
| 984 Pammi M, Haque KN. Pentoxifylline for treatment of sepsis and necrotizing enterocolitis in neonates. Cochrane Database Syst Rev. 2015(3):CD004205. | Not included for SR |
| 985 Pammi M, Haque KN. Pentoxifylline for treatment of sepsis and necrotising enterocolitis in neonates. Cochrane Database Syst Rev. 2023;6(6):CD004205. | Not included for SR |
| 986 Haase N, Perner A, Hennings LI,et al. Hydroxyethyl starch 130/0.38-0.45 versus crystalloid or albumin in patients with sepsis: systematic review with meta-analysis and trial sequential analysis. BMJ. 2013;346:f839. | Not included for SR |
| 987 Curley G F, Shehata N, Mazer C D, et al. Transfusion triggers for guiding RBC transfusion for cardiovascular surgery: a systematic review and meta-analysis. Critical care medicine, 2014, 42(12): 2611-2624. | Not included for SR |
| 988 Samy A, Abbas A M, Mahmoud M, et al. Evaluating different pain lowering medications during intrauterine device insertion: a systematic review and network meta-analysis. Fertility and Sterility, 2019, 111(3): 553-561. | Not report study-level data |
| 989 Kalampokas T, Pandian Z, Keay SD, et al. Glucocorticoid supplementation during ovarian stimulation for IVF or ICSI. Cochrane Database Syst Rev. 2017 (3):CD004752. | Not included for SR |

| | |
|---|---|
| 990 Craciunas L, Zdoukopoulos N, Vinayagam S, et al. Hormone therapy for uterine and endometrial development in women with premature ovarian insufficiency. Cochrane Database Syst Rev. 2022(10):CD008209. | Not included for SR |
| 991 Mao X, Wu L, Chen Q, et al. Effect of hysteroscopy before starting in-vitro fertilization for women with recurrent implantation failure: a meta-analysis and systematic review. Medicine, 2019, 98(7): e14075. | Not included for SR |
| 992 Marchand G J, Masoud A T, Ulibarri H, et al. Effect of the decision to perform hysteroscopy on asymptomatic patients before undergoing assisted reproduction technologies—a systematic review and meta-analysis. AJOG Global Reports, 2023, 3(2): 100178. | Not included for SR |
| 993 Di Spiezio Sardo A, Di Carlo C, Minozzi S, et al. Efficacy of hysteroscopy in improving reproductive outcomes of infertile couples: a systematic review and meta-analysis. Human reproduction update, 2016, 22(4): 479-496. | Not included for SR |
| 994 Busnelli A, Somigliana E, Cirillo F, et al. Efficacy of therapies and interventions for repeated embryo implantation failure: a systematic review and meta-analysis. Scientific Reports, 2021, 11(1): 1747. | Not included for SR |
| 995 Van Hoogenhuijze N E, Lahoz Casarramona G, Lensen S, et al. Endometrial scratching in women undergoing IVF/ICSI: an individual participant data meta-analysis. Human reproduction update, 2023, 29(6): 721-740. | Included but not synthesized/presented |
| 996 Cao H, You D, Yuan M, et al. Hysteroscopy after repeated implantation failure of assisted reproductive technology: A meta-analysis. Journal of Obstetrics and Gynaecology Research, 2018, 44(3): 365-373. | Included but not synthesized/presented |
| 997 Pundir J, Pundir V, Omanwa K, et al. Hysteroscopy prior to the first IVF cycle: a systematic review and meta-analysis. Reproductive BioMedicine Online, 2014, 28(2): 151-161. | Not included for SR |
| 998 Yang S Y, Chon S J, Lee S H. The effects of diagnostic hysteroscopy on the reproductive outcomes of infertile women without intrauterine pathologies: a systematic review and meta-analysis. Korean journal of women health nursing, 2020, 26(4): 300-317. | Not included for SR |
| 999 Abdel-Maboud M, Menshawy A, Hasabo E A, et al. The comparative effectiveness of 55 interventions in obese patients with polycystic ovary syndrome: a network meta-analysis of 101 randomized trials. PloS one, 2021, 16(7): e0254412. | Not report study-level data |
| 1000 Qin F, Zhou Y, Huan L, et al. Comparison of clomiphene and letrozole for superovulation in patients with unexplained infertility undergoing intrauterine insemination: A systematic review and meta-analysis. Medicine, 2020, 99(31): e21006. | Not included for SR |
| 1001 Qin Y. Effects of using letrozole in combination with the GnRH antagonist protocol for patients with poor ovarian response: A meta-analysis. Journal of Gynecology Obstetrics and Human Reproduction, 2021, 50(8): 102139. | Not included for SR |
| 1002 Danhof N A, Wang R, Van Wely M, et al. IUI for unexplained infertility—a network meta-analysis. Human reproduction update, 2020, 26(1): 1-15. | Not report study-level data |
| 1003 Liu A, Zheng C, Lang J, et al. Letrozole versus clomiphene citrate for unexplained infertility: A systematic review and meta-analysis. Journal of Obstetrics and Gynaecology Research, 2014, 40(5): 1205-1216. | Not included for SR |
| 1004 Polyzos N P, Tzioras S, Mauri D, et al. Treatment of unexplained infertility with aromatase inhibitors or clomiphene citrate: a systematic review and meta-analysis. Obstetrical & gynecological survey, 2008, 63(7): 472-479. | Not included for SR |

| | |
|---|---|
| 1005 Meng L, Zhao X, Sun Y, et al. Characteristics associated with effectiveness in postoperative delirium research: a systematic review of randomised controlled trials with meta-regression and meta-analysis. Br J Anaesth. 2024;133(3):565-583. | Not included for SR |
| 1006 Yiewong T, Corry M, Mooney M. Early sedation with dexmedetomidine in post-operative adult intensive care unit patients. Nursing in Critical Care, 2023, 28(5): 718-726. | Not included for SR |
| 1007 Liu H, Wei H, Qian S, et al. Effects of dexmedetomidine on postoperative sleep quality: a systematic review and meta-analysis of randomized controlled trials. BMC anesthesiology, 2023, 23(1): 88. | Not included for SR |
| 1008 Xu W, Zheng Y, Wang Q,et al. Impact of the addition of dexmedetomidine to patient-controlled intravenous analgesia on postoperative pain-sleep interaction cycle and delirium: A systematic review and meta-analysis of randomized controlled trials. Heliyon. 2024;10(6):e27623. | Included but not synthesized/presented |
| 1009 Park S K, Lim T, Cho H, et al. Comparative effectiveness of pharmacological interventions to prevent postoperative delirium: a network meta-analysis. Scientific Reports, 2021, 11(1): 11922. | Not report study-level data |
| 1010 Yu D, Shen X, Lai L, et al. Application of dexmedetomidine as an opioid substitute in opioid-free anesthesia: A systematic review and meta-analysis. Pain Physician, 2023, 26(6): E635-E649. | Not included for SR |
| 1011 Cedeno E, Vo MAJL, Tubog TD. Dexmedetomidine versus Opioids on Labor Analgesia: A Systematic Review and Meta-Analysis of Randomized Controlled Trials. AANA J. 2023;91(6):437-445. | Not included for SR |
| 1012 Asadi M, Rahimlou M, Shishehbor F, et al. The effect of l-carnitine supplementation on lipid profile and glycaemic control in adults with cardiovascular risk factors: A systematic review and meta-analysis of randomized controlled clinical trials. Clinical nutrition, 2020, 39(1): 110-122. | Not included for SR |
| 1013 Bone J N, Sandhu A, Abalos E D, et al. Oral antihypertensives for nonsevere pregnancy hypertension: systematic review, network meta-and trial sequential analyses. Hypertension, 2022, 79(3): 614-628. | Not included for SR |
| 1014 Bongiovanni T, Lancaster E, Ledesma Y, et al. Systematic review and meta–analysis of the association between non–steroidal anti–inflammatory drugs and operative bleeding in the perioperative period. Journal of the American College of Surgeons, 2021, 232(5): 765-790. | Included but not synthesized/presented |
| 1015 Zeng A M, Nami N F, Wu C L, et al. The analgesic efficacy of nonsteroidal anti-inflammatory agents (NSAIDs) in patients undergoing cesarean deliveries: a meta-analysis. Regional Anesthesia & Pain Medicine, 2016, 41(6): 763-772. | Included but not synthesized/presented |
| 1016 Albadrani M S, Elhusein A M, Ali Fadlalmola H, et al. The Clinical Effectiveness of Transcutaneous Electrical Nerve Stimulation on Enhancing Recovery after Cesarean Section: A Systematic Review and Meta-Analysis. Current Women's Health Reviews, 2024, 20(5): 53-64. | Not included for SR |
| 1017 Wang B, Yang X, Yu H, et al. The comparison of ibuprofen versus acetaminophen for blood pressure in preeclampsia: a meta-analysis of randomized controlled studies. The Journal of Maternal-Fetal & Neonatal Medicine, 2022, 35(3): 592-597. | Not included for SR |

| | |
|---|---|
| 1018 Murdoch I, Carver A L, Sultan P, et al. Comparison of different nonsteroidal anti-inflammatory drugs for cesarean section: a systematic review and network meta-analysis. Korean Journal of Anesthesiology, 2023, 76(6): 597. | Not report study-level data |
| 1019 Yu Y, Fang L, Zhang R, et al. Comparative effectiveness of 9 ovulation-induction therapies in patients with clomiphene citrate-resistant polycystic ovary syndrome: a network meta-analysis. Scientific reports, 2017, 7(1): 3812. | Not report study-level data |
| 1020 Wang A, Mo T, Li Q, et al. The effectiveness of metformin, oral contraceptives, and lifestyle modification in improving the metabolism of overweight women with polycystic ovary syndrome: a network meta-analysis. Endocrine, 2019, 64: 220-232. | Not included for SR |
| 1021 Baradwan S, Alshahrani M S, Alnoury A, et al. Does Ultrasound Guidance Provide Pain Relief During Intrauterine Contraceptive Device Insertion? A Systematic Review and Meta-Analysis of Randomized Controlled Trials. Journal of Ultrasound in Medicine, 2023, 42(7): 1401-1411. | Not included for SR |
| 1022 Ibanez-Perez J, Santos-Zorrozua B, Lopez-Lopez E, et al. An update on the implication of physical activity on semen quality: a systematic review and meta-analysis. Archives of gynecology and obstetrics, 2019, 299: 901-921. | Included but not synthesized/presented |
| 1023 Boedt T, Vanhove AC, Vercoe MA, et al. Preconception lifestyle advice for people with infertility. Cochrane Database Syst Rev. 2021(4):CD008189. | Not included for SR |
| 1024 Lu Z, Xu Y, Song Y, et al. A mixed comparisons of different intensities and types of physical exercise in patients with diseases related to oxidative stress: a systematic review and network meta-analysis. Frontiers in physiology, 2021, 12: 700055. | Not included for SR |
| 1025 Hajizadeh Maleki B, Tartibian B, Chehrazi M. Effectiveness of exercise training on male factor infertility: a systematic review and network meta-analysis. Sports Health, 2022, 14(4): 508-517. | Not report study-level data |
| 1026 Gao R, Tao Y, Zhou C, et al. Exercise therapy in patients with constipation: a systematic review and meta-analysis of randomized controlled trials. Scandinavian journal of gastroenterology, 2019, 54(2): 169-177. | Not included for SR |
| 1027 Ferreira R O, Corrêa M G, Magno M B, et al. Physical activity reduces the prevalence of periodontal disease: Systematic review and meta-analysis. Frontiers in physiology, 2019, 10: 234. | Not included for SR |
| 1028 Lu Z, Xu Y, Song Y, et al. A mixed comparisons of different intensities and types of physical exercise in patients with diseases related to oxidative stress: a systematic review and network meta-analysis. Frontiers in physiology, 2021, 12: 700055. | Not report study-level data |
| 1029 Yu L, Li J, Bian J, et al. Exercise improves alveolar bone loss and the inflammatory profile of periodontal disease. Revista Brasileira de Medicina do Esporte, 2022, 29: e2021_0333. | Not included for SR |
| 1030 Galdos-Bejar M, Mendoza-Rivera S, Orco-Leon A, et al. Does the route of administration matter? Systematic review and meta-analysis of randomized clinical trials between vaginal versus intramuscular progesterone administration in the prevention of preterm birth. Italian Journal of Gynaecology and Obstetrics.2023 | Not included for SR |
| 1031 Devall AJ, Papadopoulou A, Podesek M, et al. Progestogens for preventing miscarriage: a network meta-analysis. Cochrane Database Syst Rev. 2021(4):CD013792. | Not included for SR |

| | |
|---|---|
| 1032 Yu D, Shen X, Lai L, et al. Application of dexmedetomidine as an opioid substitute in opioid-free anesthesia: A systematic review and meta-analysis. Pain Physician, 2023, 26(6): E635-E649. | Not included for SR |
| 1033 Gao M, Meng J, Hu X, et al. Application of opioid-free general anesthesia in laparoscopy: a meta-analysis of randomized controlled studies. Signa Vitae, 2024, 20(7):10-18. | Not included for SR |
| 1034 Huang X, Cai J, Lv Z, et al. Postoperative pain after different doses of remifentanil infusion during anaesthesia: a meta-analysis. BMC anesthesiology, 2024, 24(1): 25. | Included but not synthesized/presented |
| 1035 Lee J, Kim S W, Hwang S H. The efficacy of hypotensive agents on intraoperative bleeding and recovery following general anesthesia for nasal surgery: a network meta-analysis. Clinical and experimental otorhinolaryngology, 2020, 14(2): 200-209. | Not report study-level data |
| 1036 Zhou K, Li D, Song G. Comparison of regional anesthetic techniques for postoperative analgesia after adult cardiac surgery: bayesian network meta-analysis. Frontiers in Cardiovascular Medicine, 2023, 10: 1078756. | Not report study-level data |
| 1037 Suleiman-Martos N, García-Lara R A, Membrive-Jiménez M J, et al. Effect of a game-based intervention on preoperative pain and anxiety in children: A systematic review and meta-analysis. Journal of Clinical Nursing, 2022, 31(23-24): 3350-3367. | Not included for SR |
| 1038 Abd-Alrazaq A, Alajlani M, Alhuwail D, et al. The effectiveness of serious games in alleviating anxiety: systematic review and meta-analysis. JMIR serious games, 2022, 10(1): e29137. | Included but not synthesized/presented |
| 1039 Jiang D Q, Zang Q M, Jiang L L, et al. Comparison of pramipexole and levodopa/benserazide combination therapy versus levodopa/benserazide monotherapy in the treatment of Parkinson’s disease: a systematic review and meta-analysis. Naunyn-Schmiedeberg's Archives of Pharmacology, 2021, 394: 1893-1905. | Not included for SR |
| 1040 Wang Y, Jiang D Q, Lu C S, et al. Efficacy and safety of combination therapy with pramipexole and levodopa vs levodopa monotherapy in patients with Parkinson disease: a systematic review and meta-analysis. Medicine, 2021, 100(44): e27511. | Not included for SR |
| 1041 Li T, Zou S, Zhang Z, et al. Efficacy of pramipexole on quality of life in patients with Parkinson’s disease: a systematic review and meta-analysis. BMC neurology, 2022, 22(1): 320. | Not included for SR |
| 1042 Jiang D Q, Jiang L L, Wang Y, et al. The role of pramipexole in the treatment of patients with depression and Parkinson's disease: A meta-analysis of randomized controlled trials. Asian Journal of Psychiatry, 2021, 61: 102691. | Not included for SR |
| 1043 Yu H, Lei T, Su X, et al. A systematic review and Bayesian meta-analysis of the antibiotic treatment courses in AECOPD. Frontiers in Pharmacology, 2023, 14: 1024807. | Not included for SR |
| 1044 Ghosh J, Papadopoulou A, Devall AJ, et al. Methods for managing miscarriage: a network meta-analysis. Cochrane Database Syst Rev. 2021(6):CD012602. | Not included for SR |
| 1045 Monticone M, Cedraschi C, Ambrosini E,et al. Cognitive-behavioural treatment for subacute and chronic neck pain. Cochrane Database Syst Rev.2015(5):CD010664. | Not included for SR |
| 1046 Xu Y, Song Y, Sun D, et al. Effect of multi-modal therapies for kinesiophobia caused by musculoskeletal disorders: a systematic review and meta-analysis. International journal of environmental research and public health, 2020, 17(24): 9439. | Not included for SR |

| | |
|---|---|
| 1047 Varangot-Reille C, Suso-Martí L, Romero-Palau M, et al. Effects of different therapeutic exercise modalities on migraine or tension-type headache: a systematic review and meta-analysis with a replicability analysis. The Journal of Pain, 2022, 23(7): 1099-1122. | Not included for SR |
| 1048 Cuenca-Martínez F, Sempere-Rubio N, Varangot-Reille C, et al. Effects of high-intensity interval training (HIIT) on patients with musculoskeletal disorders: a systematic review and meta-analysis with a meta-regression and mapping report. Diagnostics, 2022, 12(10): 2532. | Not included for SR |
| 1049 Baroncini A, Maffulli N, Schäfer L, et al. Physiotherapeutic and non-conventional approaches in patients with chronic low-back pain: a level I Bayesian network meta-analysis. Scientific Reports, 2024, 14(1): 11546. | Not report study-level data |
| 1050 Jurak I, Delaš K, Erjavec L, et al. Effects of Multidisciplinary Biopsychosocial Rehabilitation on Short-Term Pain and Disability in Chronic Low Back Pain: A Systematic Review with Network Meta-Analysis. Journal of clinical medicine, 2023, 12(23): 7489. | Not report study-level data |
| 1051 Wang W, Wen L, Song Z, et al. Balloon pulmonary angioplasty vs riociguat in patients with inoperable chronic thromboembolic pulmonary hypertension: A systematic review and meta-analysis. Clinical cardiology, 2019, 42(8): 741-752. | Not included for SR |
| 1052 Liu C, Chen J, Gao Y, et al. Endothelin receptor antagonists for pulmonary arterial hypertension. Cochrane Database of Systematic Reviews, 2021 (3):CD00443. | Not included for SR |
| 1053 Hsieh W C, Jansa P, Huang W C, et al. Residual pulmonary hypertension after pulmonary endarterectomy: a meta-analysis. The Journal of Thoracic and Cardiovascular Surgery, 2018, 156(3): 1275-1287. | Not included for SR |
| 1054 Ismail N R, Makhlouf H A, Hassan A, et al. An in-depth evaluation of the efficacy and safety of various treatment modalities for chronic thromboembolic pulmonary hypertension: A systematic review and network meta-analysis. American Heart Journal Plus: Cardiology Research and Practice, 2024: 100466. | Not report study-level data |
| 1055 Chen Y, Li F, Luo J, et al. Comparative efficacy and safety of targeted therapies for chronic thromboembolic pulmonary hypertension: a systematic review and network meta-analysis. Canadian Respiratory Journal, 2021, 2021(1): 1626971. | Not report study-level data |
| 1056 Schorer R, Dombret A L, Hagerman A, et al. Impact of pharmacological interventions on intrapulmonary shunt during one-lung ventilation in adult thoracic surgery: a systematic review and component network meta-analysis. British journal of anaesthesia, 2023, 130(1): e92-e105. | Not report study-level data |
| 1057 Módolo N S P, Modolo M P, Marton M A, et al. Intravenous versus inhalation anaesthesia for one-lung ventilation. Cochrane Database of Systematic Reviews, 2013 (7):CD006313. | Not included for SR |
| 1058 Xu F, Chen X Y, Liu G J, et al. Perioperative angiotensin-converting enzyme inhibitors or angiotensin II type 1 receptor blockers for preventing mortality and morbidity in adults. Cochrane Database of Systematic Reviews, 2016 (1):CD009210. | Not included for SR |
| 1059 Desborough M J R, Sandu R, Brunskill S J, et al. Fresh frozen plasma for cardiovascular surgery. Cochrane Database of Systematic Reviews, 2015 (7):CD007614. | Not included for SR |

| | |
|---|---|
| 1060 Azizi Kutenaei M, Hosseini Teshnizi S, Ghaemmaghami P, et al. The effects of myo-inositol vs. metformin on the ovarian function in the polycystic ovary syndrome: a systematic review and meta-analysis. Eur Rev Med Pharmacol Sci. 2021;25(7):3105-3115. | Not report study-level data |
| 1061 Abdel-Maboud M, Menshawy A, Hasabo E A, et al. The comparative effectiveness of 55 interventions in obese patients with polycystic ovary syndrome: a network meta-analysis of 101 randomized trials. PloS one, 2021, 16(7): e0254412. | Not report study-level data |
| 1062 Su Q, Pan Z, Yin R, et al. The value of G-CSF in women experienced at least one implantation failure: a systematic review and meta-analysis. Frontiers in Endocrinology, 2024, 15: 1370114. | Not included for SR |
| 1063 Maged A M, El-Mazny A, Kamal N, et al. The value of platelet-rich plasma in women with previous implantation failure: A systematic review and meta-analysis. Journal of assisted reproduction and genetics, 2023, 40(5): 969-983. | Not included for SR |
| 1064 Franik S, Le Q K, Kremer J A M, et al. Aromatase inhibitors (letrozole) for ovulation induction in infertile women with polycystic ovary syndrome. Cochrane Database of Systematic Reviews, 2022 (9):CD010287 | Included but not synthesized/presented |
| 1065 Sharpe A, Morley L C, Tang T, et al. Metformin for ovulation induction (excluding gonadotrophins) in women with polycystic ovary syndrome. Cochrane Database of Systematic Reviews, 2019(12):CD013505 | Not included for SR |
| 1066 Previtali D, Albanese J, Romandini I, et al. Placebo Effect in the Treatment of Patellar Tendinopathy and Its Influencing Factors: Systematic Review With Meta-analysis and Meta Regression of Randomized Controlled Trials. Orthopaedic Journal of Sports Medicine, 2024, 12(8): 23259671241258477. | Not included for SR |
| 1067 Ragone F, Pérez-Guillén S, Carrasco-Uribarren A, et al. The Effects of Soft-Tissue Techniques and Exercise in the Treatment of Patellar Tendinopathy-Systematic Review and Meta-Analysis. Healthcare (Basel). 2024;12(4):427. | Included but not synthesized/presented |
| 1068 Wang S, Lyu B. Effectiveness of Injection Strategies on Patients With Patellar Tendonitis (Jumpers' Knee): A Network Meta-analysis of Randomized Controlled Trials. Sports Health, 2024: 19417381241263338. | Not report study-level data |
| 1069 Bunn F, Trivedi D. Colloid solutions for fluid resuscitation. Cochrane Database Syst Rev. 2012(7):CD001319. | Not included for SR |
| 1070 Bunn F, Trivedi D, Ashraf S. Colloid solutions for fluid resuscitation. Cochrane Database Syst Rev. 2008(1):CD001319. | Not included for SR |
| 1071 Martin C, Jacob M, Vicaut E, et al. Effect of waxy maize-derived hydroxyethyl starch 130/0.4 on renal function in surgical patients. Survey of Anesthesiology, 2013, 57(5): 246-247. | Not included for SR |
| 1072 Mutter T C, Ruth C A, Dart A B. Hydroxyethyl starch (HES) versus other fluid therapies: effects on kidney function. Cochrane Database of Systematic Reviews, 2013 (7):CD007594. | Not included for SR |
| 1073 Shi X Y, Zou Z, He X Y, et al. Hydroxyethyl starch for cardiovascular surgery: a systematic review of randomized controlled trials. European journal of clinical pharmacology, 2011, 67: 767-782. | Not report study-level data |
| 1074 Zacharias M, Conlon NP, Herbison GP, et al. Interventions for protecting renal function in the perioperative period. Cochrane Database Syst Rev. 2008 (4):CD003590. | Not included for SR |

| | |
|---|---|
| 1075 Zacharias M, Mugawar M, Herbison GP, et al. Interventions for protecting renal function in the perioperative period. Cochrane Database Syst Rev. 2013(9):CD003590. | Not included for SR |
| 1076 Munoz J J, Birkmeyer N J O, Birkmeyer J D, et al. Is ε-aminocaproic acid as effective as aprotinin in reducing bleeding with cardiac surgery? A meta-analysis. Circulation, 1999, 99(1): 81-89. | Not report study-level data |
| 1077 Brown J R, Birkmeyer N J O, O'Connor G T. Meta-analysis comparing the effectiveness and adverse outcomes of antifibrinolytic agents in cardiac surgery. Circulation, 2007, 115(22): 2801-2813. | Not report study-level data |
| 1078 Geurts M, Macleod M R, Kollmar R, et al. Therapeutic hypothermia and the risk of infection: a systematic review and meta-analysis. Critical care medicine, 2014, 42(2): 231-242. | Not included for SR |
| 1079 Walder B, Schafer M, Henzi I, et al. Efficacy and safety of patient-controlled opioid analgesia for acute postoperative pain: a quantitative systematic review. Acta Anaesthesiologica Scandinavica, 2001, 45(7): 795-804. | Not report study-level data |
| 1080 Bainbridge D, Martin J E, Cheng D C. Patient-controlledversus nurse-controlled analgesia after cardiac surgery—a meta-analysis. Canadian Journal of Anesthesia/Journal canadien d'anesthésie, 2006, 53(5): 492-499. | Not report study-level data |
| 1081 Crowe L, Chang A, Fraser J A, et al. Systematic review of the effectiveness of nursing interventions in reducing or relieving post-operative pain. International Journal of Evidence-Based Healthcare, 2008, 6(4): 396-430. | Included but not synthesized/presented |
| 1082 Cochrane Pregnancy and Childbirth Group, Pels A, Ganzevoort W, et al. Interventions affecting the nitric oxide pathway versus placebo or no therapy for fetal growth restriction in pregnancy. Cochrane Database of Systematic Reviews, 1996, 2023(7):CD014498. | Not included for SR |
| 1083 Turner J M, Russo F, Deprest J, et al. Phosphodiesterase-5 inhibitors in pregnancy: Systematic review and meta-analysis of maternal and perinatal safety and clinical outcomes. BJOG: An International Journal of Obstetrics & Gynaecology, 2022, 129(11): 1817-1831. | Not included for SR |
| 1084 Heshmati J, Farsi F, Shokri F, et al. A systematic review and meta-analysis of the probiotics and synbiotics effects on oxidative stress. Journal of functional foods, 2018, 46: 66-84. | Included but not synthesized/presented |
| 1085 Saadati S, Naseri K, Asbaghi O, et al. Beneficial effects of the probiotics and synbiotics supplementation on anthropometric indices and body composition in adults: A systematic review and meta-analysis. Obesity Reviews, 2024, 25(3): e13667. | Not report study-level data |
| 1086 Hadi A, Moradi S, Ghavami A, et al. Effect of probiotics and synbiotics on selected anthropometric and biochemical measures in women with polycystic ovary syndrome: a systematic review and meta-analysis. European journal of clinical nutrition, 2020, 74(4): 543-547. | Not report study-level data |
| 1087 Liao D, Zhong C, Li C, et al. Meta-analysis of the effects of probiotic supplementation on glycemia, lipidic profiles, weight loss and C-reactive protein in women with polycystic ovarian syndrome. Minerva medica, 2018, 109(6): 479-487. | Not report study-level data |

| | |
|---|---|
| 1088 Shamasbi S G, Ghanbari-Homayi S, Mirghafourvand M. The effect of probiotics, prebiotics, and synbiotics on hormonal and inflammatory indices in women with polycystic ovary syndrome: a systematic review and meta-analysis. European journal of nutrition, 2020, 59: 433-450. | Included but not synthesized/presented |
| 1089 Stewart L A, Simmonds M, Duley L, et al. Evaluating Progestogens for Preventing Preterm birth International Collaborative (EPPPIC): meta-analysis of individual participant data from randomised controlled trials. The Lancet, 2021, 397(10280): 1183-1194. | Not included for SR |
| 1090 Sotiriadis A, Papatheodorou S, Makrydimas G. Perinatal outcome in women treated with progesterone for the prevention of preterm birth: a meta-analysis. Ultrasound in obstetrics & gynecology, 2012, 40(3): 257-266. | Not included for SR |
| 1091 Likis F E, Edwards D R V, Andrews J C, et al. Progestogens for preterm birth prevention: a systematic review and meta-analysis. Obstetrics & Gynecology, 2012, 120(4): 897-907. | Included but not synthesized/presented |
| 1092 D'Antonio F, Berghella V, Di Mascio D, et al. Role of progesterone, cerclage and pessary in preventing preterm birth in twin pregnancies: A systematic review and network meta-analysis. European Journal of Obstetrics & Gynecology and Reproductive Biology, 2021, 261: 166-177. | Not report study-level data |
| 1093 Ahn K H, Bae N Y, Hong S C, et al. The safety of progestogen in the prevention of preterm birth: meta-analysis of neonatal mortality. Journal of perinatal medicine, 2017, 45(1): 11-20. | Not included for SR |
| 1094 Jarde A, Lutsiv O, Park C K, et al. Effectiveness of progesterone, cerclage and pessary for preventing preterm birth in singleton pregnancies: a systematic review and network meta-analysis. BJOG: An International Journal of Obstetrics & Gynaecology, 2017, 124(8): 1176-1189. | Not report study-level data |
| 1095 Conde-Agudelo A, Romero R, Da Fonseca E, et al. Vaginal progesterone is as effective as cervical cerclage to prevent preterm birth in women with a singleton gestation, previous spontaneous preterm birth, and a short cervix: updated indirect comparison meta-analysis. American journal of obstetrics and gynecology, 2018, 219(1): 10-25. | Not report study-level data |
| 1096 Combs C A, Schuit E, Caritis S N, et al. 17-Hydroxyprogesterone caproate in triplet pregnancy: an individual patient data meta-analysis. BJOG: An International Journal of Obstetrics & Gynaecology, 2016, 123(5): 682-690. | Not included for SR |
| 1097 Storr B, Peiris C L, Snowdon D A. Community reintegration after rehabilitation for hip fracture: a systematic review and meta-analysis. Archives of physical medicine and rehabilitation, 2022, 103(8): 1638-1650. e7. | Not included for SR |
| 1098 Zhang X, Butts W J, You T. Exercise interventions, physical function, and mobility after hip fracture: a systematic review and meta-analysis. Disability and Rehabilitation, 2022, 44(18): 4986-4996. | Not included for SR |
| 1099 Fatoye F, Wright JM, Yeowell G, et al. Clinical and cost-effectiveness of physiotherapy interventions following total hip replacement: a systematic review and meta-analysis. Rheumatol Int. 2020 Sep;40(9):1385-1398. | Included but not synthesized/presented |
| 1100 Alfirevic Z, Stampalija T, Medley N. Cervical stitch (cerclage) for preventing preterm birth in singleton pregnancy. Cochrane Database Syst Rev. 2017(6):CD008991. | Not included for SR |

| | |
|---|---|
| 1101 Eleje GU, Eke AC, Ikechebelu JI, et al. Cervical stitch (cerclage) in combination with other treatments for preventing spontaneous preterm birth in singleton pregnancies. Cochrane Database Syst Rev. 2020(9):CD012871. | Not included for SR |
| 1102 Aubin A M, McAuliffe L, Williams K, et al. Combined vaginal progesterone and cervical cerclage in the prevention of preterm birth: a systematic review and meta-analysis. American Journal of Obstetrics & Gynecology MFM, 2023, 5(8): 101024. | Included but not synthesized/presented |
| 1103 Franik S, Le Q K, Kremer J A M, et al. Aromatase inhibitors (letrozole) for ovulation induction in infertile women with polycystic ovary syndrome. Cochrane Database of Systematic Reviews, 2022 (9). | Not included for SR |
| 1104 Akinoso-Imran A Q, Adetunji H. Systematic review and meta-analysis of letrozole and clomiphene citrate in polycystic ovary syndrome. Middle East Fertility Society Journal, 2018, 23(3): 163-170. | Not included for SR |
| 1105 Moradkhani A, Azami M, Assadi S, et al. Association of vitamin D receptor genetic polymorphisms with the risk of infertility: a systematic review and meta-analysis. BMC Pregnancy and Childbirth, 2024, 24(1): 398. | Not included for SR |
| 1106 Franik S, Le QK, Kremer JA, et al. Aromatase inhibitors (letrozole) for ovulation induction in infertile women with polycystic ovary syndrome. Cochrane Database Syst Rev. 2022(9):CD010287 | Not included for SR |
| 1107 Franik S, Eltrop SM, Kremer JA, et al. Aromatase inhibitors (letrozole) for subfertile women with polycystic ovary syndrome. Cochrane Database Syst Rev. 2018(5):CD010287. | Not included for SR |
| 1108 Franik S, Kremer JA, Nelen WL, et al. Aromatase inhibitors for subfertile women with polycystic ovary syndrome. Cochrane Database Syst Rev. 2014(2):CD010287. | Not included for SR |
| 1109 Rizzuto I, Behrens RF, Smith LA. Risk of ovarian cancer in women treated with ovarian stimulating drugs for infertility. Cochrane Database Syst Rev. 2019(6):CD008215. | Not included for SR |
| 1110 Rizzuto I, Behrens RF, Smith LA. Risk of ovarian cancer in women treated with ovarian stimulating drugs for infertility. Cochrane Database Syst Rev. 2013(8):CD008215 | Not included for SR |
| 1111 Abdel-Maboud M, Menshawy A, Hasabo E A, et al. The comparative effectiveness of 55 interventions in obese patients with polycystic ovary syndrome: a network meta-analysis of 101 randomized trials. PloS one, 2021, 16(7): e0254412. | Not report study-level data |
| 1112 Naseri K, Saadati S, Yari Z, et al. Beneficial effects of probiotic and synbiotic supplementation on some cardiovascular risk factors among individuals with prediabetes and type 2 diabetes mellitus: A grade-assessed systematic review, meta-analysis, and meta-regression of randomized clinical trials. Pharmacological research, 2022, 182: 106288. | Not included for SR |
| 1113 Saadati S, Naseri K, Asbaghi O, et al. Beneficial effects of the probiotics and synbiotics supplementation on anthropometric indices and body composition in adults: A systematic review and meta-analysis. Obesity Reviews, 2024, 25(3): e13667. | Not included for SR |
| 1114 Khalili L, Valdes-Ramos R, Harbige L S. Effect of n-3 (Omega-3) polyunsaturated fatty acid supplementation on metabolic and inflammatory biomarkers and body weight in patients with type 2 diabetes mellitus: A systematic review and meta-analysis of RCTs. Metabolites, 2021, 11(11): 742. | Not included for SR |

| | |
|---|---|
| 1115 Hong J, Fu T, Liu W, et al. Specific alterations of gut microbiota in diabetic microvascular complications: A systematic review and meta-analysis. Frontiers in Endocrinology, 2022, 13: 1053900. | Not included for SR |
| 1116 Moravejolahkami A R, Hojjati Kermani M A, Balouch Zehi Z, et al. The effect of probiotics on lipid profile & anthropometric indices in diabetic nephropathy; a systematic review and meta-analysis of clinical trials. Journal of Diabetes & Metabolic Disorders, 2021, 20: 893-904. | Not included for SR |
| 1117 Khalili L, Nammi S. The Effects of Curcumin Supplementation on Metabolic Biomarkers and Body Mass Index in Patients with Nonalcoholic Fatty Liver Disease: A Systematic Review and Meta-analysis of Randomized Controlled Trials. Curr Pharm Des. 2022;28(23):1911-1925. | Not included for SR |
| 1118 Harsinay A, Patil A, Ali-Khan S, et al. Needles, Herbs, and Electricity: A Meta-Analysis of Traditional Eastern Medicine in the Management of Facial Paralysis. Facial Plast Surg. 2024;40(4):441-449. | Not report study-level data |
| 1119 Wang D, Fang X. Meta-analysis of the efficacy of neoadjuvant chemotherapy for locally advanced cervical cancer. Eur J Obstet Gynecol Reprod Biol. 2024;297:202-208. | Not included for SR |
| 1120 Nagels HE, Rishworth JR, Siristatidis CS, et al. Androgens (dehydroepiandrosterone or testosterone) for women undergoing assisted reproduction. Cochrane Database Syst Rev. 2015(11):CD009749. | Review author excluded this study in SR |
| 1121 Purgato M, Prina E, Ceccarelli C, et al. Primary-level and community worker interventions for the prevention of mental disorders and the promotion of well-being in low- and middle-income countries. Cochrane Database Syst Rev. 2023(10):CD014722. | Not included for SR |
| 1122 Palacios C, Kostiuk L K, Peña-Rosas J P. Vitamin D supplementation for women during pregnancy. Cochrane Database of Systematic Reviews, 2019 (7):CD00887. | Not included for SR |
| 1123 Zhu R, Yang F, Li C, et al. Effect of enhanced recovery after surgery on the prognosis of patients with hip fractures: a systematic review and meta-analysis. Journal of Trauma Nursing\| JTN, 2023, 30(5): 271-281. | Not included for SR |
| 1124 Huang Q, Li Y, Luo X, et al. Effect of peri operative whole high quality nursing care on psychological status, vital signs and anesthetic medication of patients undergoing painless gastrointestinal endoscopy: A systematic review and meta analysis. Tropical Journal of Pharmaceutical Research, 2023, 22(11): 2389-2397. | Not included for SR |
| 1125 Poursalehian M, Tajvidi M, Ghaderpanah R, et al. Efficacy and Safety of Oral Tranexamic Acid vs. Other Routes in Total Joint Arthroplasty: A Systematic Review and Network Meta-Analysis. JBJS reviews, 2024, 12(6): e23. | Not report study-level data |
| 1126 Zhu R, Yang F, Li C, et al. Effect of Enhanced Recovery After Surgery on the Prognosis of Patients With Hip Fractures: A Systematic Review and Meta-Analysis. J Trauma Nurs. 2023;30(5):271-281. | Not included for SR |
| 1127 Zhao YK, Zhang C, Zhang YW, et al. Efficacy and safety of tranexamic acid in elderly patients with femoral neck fracture treated with hip arthroplasty: A systematic review and meta-analysis. J Orthop Sci. 2024;29(2):542-551. | Not included for SR |

| | |
|---|---|
| 1128 Das S, Panda S, Nayak R, et al. Predictability and clinical stability of barrier membranes in treatment of periodontal intrabony defects: A systematic review and meta-analysis. Applied Sciences, 2022, 12(10): 4835. | Not included for SR |
| 1129 Pessano S, Gloeck NR, Tancredi L, et al. Ibuprofen for acute postoperative pain in children. Cochrane Database Syst Rev. 2024(1):CD015432. | Review author excluded this study in SR |
| 1130 Zhou Z, Li C, Yuan J, et al. Effectiveness of sports intervention: A meta-analysis of the effects of different interventions on adolescent internet addiction. Journal of Affective Disorders, 2024. | Not included for SR |
| 1131 Shu J, Lu T, Tao B, et al. Effects of aerobic exercise on body self-esteem among Chinese college students: A meta-analysis. Plos one, 2023, 18(9): e0291045. | Not included for SR |
| 1132 Zhu Y, Chen H, Li J, et al. Effects of different interventions on internet addiction: a systematic review and network meta-analysis. BMC psychiatry, 2023, 23(1): 921. | Not report study-level data |
| 1133 Du Y, Zhang H, Ma Z, et al. High‐Flow Nasal Oxygen versus Noninvasive Ventilation in Acute Exacerbation of Chronic Obstructive Pulmonary Disease Patients: A Meta‐Analysis of Randomized Controlled Trials. Canadian Respiratory Journal, 2023, 2023(1): 7707010. | Not included for SR |
| 1134 Wu J L, Zhang Q, Zhang L H, et al. Effect of comprehensive nursing intervention on wound pain and wound complications in patients with tonsillectomy: A meta‐analysis. International Wound Journal, 2024, 21(4): e14619. | Not included for SR |
| 1135 He Y, Chen L, Li X, et al. Clinical Evidence and Potential Mechanisms of Traditional Chinese Medicine for the Treatment of Chronic Gastritis: An Updated Systematic Review and Meta-analysis. Journal of Herbal Medicine, 2023, 42: 100761. | Not included for SR |
| 1136 Alameddine S, Lucidi A, Jurkovic D, et al. Treatments for cesarean scar pregnancy: A systematic review and meta-analysis. The Journal of Maternal-Fetal & Neonatal Medicine, 2024, 37(1): 2327569. | Not report study-level data |
| 1137 Ehrenzeller S, Klompas M. Association Between Daily Toothbrushing and Hospital-Acquired Pneumonia: A Systematic Review and Meta-Analysis. JAMA Intern Med. 2024;184(2):131-142. | Not included for SR |
| 1138 Zhang J, Wu W, Ren Y, et al. Electroacupuncture for the treatment of cancer pain: a systematic review and meta-analysis of randomized clinical trials. Frontiers in Pain Research, 2023, 4: 1186506. | Not included for SR |
| 1139 Parsaei M, Hasehmi S M, Seyedmirzaei H, et al. Perioperative esketamine administration for prevention of postpartum depression after the cesarean section: A systematic review and meta-analysis. J Affect Disord. 2024;361:564-580 | Included but not synthesized/presented |
| 1140 Zhao Y, Xiao X. Efficacy of ultrasound-guided stellate ganglion block in relieving acute postoperative pain: a systematic review and meta-analysis. Journal of International Medical Research, 2024, 52(5): 03000605241252237. | Not included for SR |
| 1141 Luo M, Xiong L, Zhang L, et al. Efficacy and safety of Bifidobacterium quadruple viable tablets combined with mosapride citrate in the treatment of constipation in China: a systematic review and meta-analysis. BMC gastroenterology, 2023, 23(1): 245. | Not included for SR |

| | |
|---|---|
| 1142 Gao L, Wang S, Zhang L, et al. Efficacy of Shenlingbaizhusan for antibiotic-associated diarrhea: A systematic review and meta-analysis. Journal of Herbal Medicine, 2023, 40: 100676. | Not included for SR |
| 1143 Chen J, Liu L, Wang Y, et al. Effects of psychotherapy interventions on anxiety and depression in patients with gastrointestinal cancer: A systematic review and network meta-analysis. Journal of Psychosomatic Research, 2024: 111609. | Not included for SR |
| 1144 Wang Y, Shen Q, Wang C. Efficacy of Rapid Rehabilitation Nursing in Postoperative Care in China: A Meta-Analysis. Rehabilitation Nursing Journal, 2023, 48(5): 170-179. | Not report study-level data |
| 1145 Huang Q, Zhang Q, Li S. Efficacy and Safety of Integrated Maxing Shigan Decoction and Azithromycin for Mycoplasma Pneumoniae Pneumonia in Children: A Systematic Review and Meta-Analysis. Journal of Clinical Pharmacy and Therapeutics, 2023: 2596562. | Not included for SR |
| 1146 Xiao Y, Nie M, Xu W, et al. The efficiency of human fat products in wound healing: A systematic review and meta-analysis. International wound journal, 2024, 21(9): e70016. | Included but not synthesized/presented |
| 1147 Li C, Zhang M, Karthijekan K, et al. How nurses contribute to the elimination of hepatitis B? A systematic review and meta-analysis. International Journal of Nursing Studies, 2024, 149: 104622. | Included but not synthesized/presented |
| 1148 Yan X, Zhou L, He G, et al. Pregnancy rate and outcomes after uterine artery embolization for women: a systematic review and meta-analysis with trial sequential analysis. Frontiers in Medicine, 2023, 10: 1283279. | Not included for SR |
| 1149 Luo J, Huang B, Zheng H, et al. Acupuncture combined with balloon dilation for post-stroke cricopharyngeal achalasia: A meta-analysis of randomized controlled trials. Frontiers in Neuroscience, 2023, 16: 1092443. | Not included for SR |
| 1150 Lin J, Kong Y, Chen H, et al. Effects of acupuncture on sleep quality in patients with Parkinson's disease: A systematic review and meta-analysis. Clinical Rehabilitation, 2024, 38(4): 478-496. | Included but not synthesized/presented |
| 1151 Lei S, Fan J, Liu X, et al. Qualitative and quantitative meta-analysis of acupuncture effects on the motor function of Parkinson's disease patients. Frontiers in Neuroscience, 2023, 17: 1125626. | Not included for SR |
| 1152 Wang R, Miao C, Chen Y, et al. Antioxidant supplements relieve insulin resistance but do not improve lipid metabolism in women with polycystic ovary syndrome: a meta-analysis of randomized clinical trials. Gynecological Endocrinology, 2022, 38(12): 1047-1059. | Not included for SR |
| 1153 Farsi F, Heshmati J, Keshtkar A, et al. Can coenzyme Q10 supplementation effectively reduce human tumor necrosis factor-α and interleukin-6 levels in chronic inflammatory diseases? A systematic review and meta-analysis of randomized controlled trials. Pharmacological Research, 2019, 148: 104290. | Not included for SR |
| 1154 Alimohammadi M, Rahimi A, Faramarzi F, et al. Effects of coenzyme Q10 supplementation on inflammation, angiogenesis, and oxidative stress in breast cancer patients: a systematic review and meta-analysis of randomized controlled-trials. Inflammopharmacology, 2021, 29(3): 579-593. | Not included for SR |

| | |
|---|---|
| 1155 Zhang J, Xing C, Zhao H, et al. The effectiveness of coenzyme Q10, vitamin E, inositols, and vitamin D in improving the endocrine and metabolic profiles in women with polycystic ovary syndrome: a network meta-analysis. Gynecological Endocrinology, 2021, 37(12): 1063-1071. | Not report study-level data |
| 1156 Chen H, Xiao L, Li J, et al. Adjuvant gonadotropin-releasing hormone analogues for the prevention of chemotherapy-induced premature ovarian failure in premenopausal women. Cochrane Database Syst Rev. 2019 (3):CD008018. | old version |
| 1157 Showell MG, Mackenzie-Proctor R, Jordan V, Hart RJ. Antioxidants for female subfertility. Cochrane Database Syst Rev. 2017(7):CD007807. | old version |
| 1158 Showell MG, Brown J, Clarke J, et al. Antioxidants for female subfertility. Cochrane Database Syst Rev. 2013 (8):CD007807. | old version |
| 1159 Showell MG, Mackenzie-Proctor R, Jordan V, Hart RJ. Antioxidants for female subfertility. Cochrane Database Syst Rev. 2017(7):CD007807. | old version |
| 1160 Showell MG, Mackenzie-Proctor R, Jordan V, Hart RJ. Antioxidants for female subfertility. Cochrane Database Syst Rev. 2017(7):CD007807. | old version |
| 1161 Duncan D, Sankar A, Beattie WS, et al. Alpha-2 adrenergic agonists for the prevention of cardiac complications among adults undergoing surgery. Cochrane Database Syst Rev. 2018(3):CD004126. | Included but not synthesized/presented |
| 1162 Ho K M, Tan J A. Benefits and risks of maintaining normothermia during cardiopulmonary bypass in adult cardiac surgery: a systematic review. Cardiovascular therapeutics, 2011, 29(4): 260-279. | Included but not synthesized/presented |
| 1163 Fidelix TS, Macedo CR, Maxwell LJ, et al. Diacerein for osteoarthritis. Cochrane Database Syst Rev. 2014(2):CD005117 | Not report study-level data |
| 1164 Recchia F, Leung CK, Yu AP, et al. Dose-response effects of exercise and caloric restriction on visceral adiposity in overweight and obese adults: a systematic review and meta-analysis of randomised controlled trials. Br J Sports Med. 2023(16):1035-1041. | Insufficient information for reproducing |
| 1165 Di Girolamo R, Galliani C, Buca D, et al. Outcomes of second stage cesarean section following the use of a fetal head elevation device: A systematic review and meta-analysis. Eur J Obstet Gynecol Reprod Biol. 2021:262:1-6. | Included but not synthesized/presented |
| 1166 Lepine S, Jo J, Metwally M, et al. Ovarian surgery for symptom relief in women with polycystic ovary syndrome. Cochrane Database Syst Rev. 2017(11):CD009526. | Included but not synthesized/presented |
| 1167 Zamani M, Pahlavani N, Nikbaf-Shandiz M, et al. The effects of L-carnitine supplementation on glycemic markers in adults: A systematic review and dose-response meta-analysis. Front Nutr. 2023;9:1082097. | Not report study-level data |
| 1168 Franik S, Eltrop SM, Kremer JA, et al. Aromatase inhibitors (letrozole) for subfertile women with polycystic ovary syndrome. Cochrane Database Syst Rev. 2018;5(5):CD010287. | Included but not synthesized/presented |

| | |
|---|---|
| 1169 Sun J, Rahmati M, Xie W, et al. Efficacy and safety of zoledronic acid in the treatment of osteoporosis: A meta-analysis of randomized controlled trials. Heliyon, 2024, 10(13). | Not report study-level data |
| 1170 Nastri CO, Gibreel A, Raine-Fenning N, et al. Endometrial injury in women undergoing assisted reproductive techniques. Cochrane Database Syst Rev. 2012;(7):CD009517. | old version |
| 1171 Kamper SJ, Apeldoorn AT, Chiarotto A, et al. Multidisciplinary biopsychosocial rehabilitation for chronic low back pain. Cochrane Database Syst Rev.2014(9):CD000963. | Duplicate |
| 1172 Hudcova J, McNicol E, Quah C, et al. Patient controlled opioid analgesia versus conventional opioid analgesia for postoperative pain. Cochrane Database Syst Rev. 2006(4):CD003348. | old version |
| 1173 Chen Z, Wang J, Carru C, et al. Treatment for mild hypertension in pregnancy with different strategies: A systematic review and meta-analysis. Int J Gynaecol Obstet. 2023;162(1):202-210. | Included but not synthesized/presented |
| 1174 Zhang J, Tang L, Kong L, et al. Ultrasound-guided transvaginal ovarian needle drilling for clomiphene-resistant polycystic ovarian syndrome in subfertile women. Cochrane Database Syst Rev. 2019(7):CD008583. | Included but not synthesized/presented |
| 1175 Bruno V, Chiofalo B, Logoteta A, et al. Urological Complications in Radical Surgery for Cervical Cancer: A Comparative Meta-Analysis before and after LACC Trial. Journal of Clinical Medicine, 2023, 12(17): 5677. | Incidence |

**Supplementary Table S5. Systematic reviews that incorporated retracted clinical trails eligible for analysis (n = 847)**

1 Bwire GM, Njiro BJ, Ndumwa HP, et al. Impact of differentiated service delivery models on retention in HIV care and viral suppression among people living with HIV in sub-Saharan Africa: A systematic review and meta-analysis of randomised controlled trials. Rev Med Virol. 2023;33(6):e2479.

2 Zhang M, Zhu F, Jia F, et al. Efficacy of brain-computer interfaces on upper extremity motor function rehabilitation after stroke: A systematic review and meta-analysis. NeuroRehabilitation. 2024;54(2):199-212.

3 Guo Z, Gao Q, Jiang Y, et al. Theraputic Effects of Brain-Computer Interface on Motor Recovery of Stroke Patients: A Meta-analysis. medRxiv, 2023: 2023.04. 11.23288439.

4 Chen CW, Lee MC, Wu SV. Effects of a collaborative health management model on people with congestive heart failure: A systematic review and meta-analysis. J Adv Nurs. 2024;80(6):2290-2307.

5 Farquhar C, Brown J, Marjoribanks J. Laparoscopic drilling by diathermy or laser for ovulation induction in anovulatory polycystic ovary syndrome. Cochrane Database Syst Rev. 201213;(6):CD001122.

6 Abu Hashim H, Foda O, El Rakhawy M. Unilateral or bilateral laparoscopic ovarian drilling in polycystic ovary syndrome: a meta-analysis of randomized trials. Arch Gynecol Obstet. 2018;297(4):859-870.

7 Bosteels J, Weyers S, Mathieu C, et al. The effectiveness of reproductive surgery in the treatment of female infertility: facts, views and vision. Facts Views Vis Obgyn. 2010;2(4):232-52.

8 Jarde A, Lutsiv O, Beyene J, et al. Vaginal progesterone, oral progesterone, 17-OHPC, cerclage, and pessary for preventing preterm birth in at-risk singleton pregnancies: an updated systematic review and network meta-analysis. BJOG. 2019 Apr;126(5):556-567.

9 Li W, Gleeson J, Fraser MI, et al. The efficacy of personalized psychological interventions in adolescents: a scoping review and meta-analysis. Front Psychol. 2024 Sep 6;15:1470817.

10 Honvo G, Reginster JY, Rabenda V, et al. Safety of Symptomatic Slow-Acting Drugs for Osteoarthritis: Outcomes of a Systematic Review and Meta-Analysis. Drugs Aging. 2019;36(Suppl 1):65-99.

11 Kongtharvonskul J, Anothaisintawee T, McEvoy M, et al. Efficacy and safety of glucosamine, diacerein, and NSAIDs in osteoarthritis knee: a systematic review and network meta-analysis. Eur J Med Res. 2015;20(1):24.

12 Bartels EM, Bliddal H, Schøndorff PK, et al. Symptomatic efficacy and safety of diacerein in the treatment of osteoarthritis: a meta-analysis of randomized placebo-controlled trials. Osteoarthritis Cartilage. 2010;18(3):289-96.

13 Aghamohammadi D, Dolatkhah N, Bakhtiari F, et al. Nutraceutical supplements in management of pain and disability in osteoarthritis: a systematic review and meta-analysis of randomized clinical trials. Sci Rep. 2020;10(1):20892.

14 Yu L, Luo R, Qin G, et al. Efficacy and safety of anti-interleukin-1 therapeutics in the treatment of knee osteoarthritis: a systematic review and meta-analysis of randomized controlled trials. J Orthop Surg Res. 2023;18(1):100.

15 Li G, Zhang Z, Ye Y,et al. Efficacy, residual effectiveness and safety of diacerein in the treatment of knee osteoarthritis: A meta-analysis of randomized placebo-controlled trials. Medicine (Baltimore). 2022;101(46):e31700.

16 Ahmed S H, Hasan S U, Samad S, et al. A network meta-analysis comparing the efficacy and safety of thoracic endovascular aortic repair with open surgical repair and optimal medical therapy for type B aortic dissection. JVS-Vascular Insights, 2024: 100068.
17 Chen X, Wen Q, Kou L, et al. Incidence and risk of hypertension associated with PARP inhibitors in cancer patients: a systematic review and meta-analysis. BMC Cancer. 2023;23(1):107.
18 Qi W, Hu G, Zou T, et al. Effects of self-management education integrated nursing on cancer patients with PICC placement: a systematic review and meta-analysis. J Res Nurs. 2024:17449871241268513.
19 Showell M G, Mackenzie-Proctor R, Jordan V, et al. Antioxidants for female subfertility. Cochrane database of systematic reviews, 2020 (8).
20 Shukri M F M, Norhayati M N, Badrin S, et al. Effects of L-carnitine supplementation for women with polycystic ovary syndrome: a systematic review and meta-analysis. PeerJ, 2022, 10: e13992.
21 Zhang S, Li N, Mao X, et al. Effect of comprehensive nursing care for the liver cancer patients undergoing interventional therapy in China: A systematic review and meta-analysis. International Journal of Nursing Practice, 2024, 30(2): e13243.
22 Asl Z S, Parastouei K, Eskandari E. The effects of N-acetylcysteine on ovulation and sex hormones profile in women with polycystic ovary syndrome: a systematic review and meta-analysis. British Journal of Nutrition, 2023, 130(2): 202-210.
23 Devi N, Boya C, Chhabra M, et al. N-acetyl-cysteine as adjuvant therapy in female infertility: a systematic review and meta-analysis. Journal of basic and clinical physiology and pharmacology, 2021, 32(5): 899-910.
24 Lambertini M, Ceppi M, Poggio F, et al. Ovarian suppression using luteinizing hormone-releasing hormone agonists during chemotherapy to preserve ovarian function and fertility of breast cancer patients: a meta-analysis of randomized studies. Annals of Oncology, 2015, 26(12): 2408-2419.
25 Del Mastro L, Ceppi M, Poggio F, et al. Gonadotropin-releasing hormone analogues for the prevention of chemotherapy-induced premature ovarian failure in cancer women: systematic review and meta-analysis of randomized trials. Cancer treatment reviews, 2014, 40(5): 675-683.
26 Munhoz R R, Pereira A A L, Sasse A D, et al. Gonadotropin-releasing hormone agonists for ovarian function preservation in premenopausal women undergoing chemotherapy for early-stage breast cancer: a systematic review and meta-analysis. JAMA oncology, 2016, 2(1): 65-73.
27 Bedaiwy M A, Abou-Setta A M, Desai N, et al. Gonadotropin-releasing hormone analog cotreatment for preservation of ovarian function during gonadotoxic chemotherapy: a systematic review and meta-analysis. Fertility and sterility, 2011, 95(3): 906-914. e4.
28 Senra J C, Roque M, Talim M C T, et al. Gonadotropin-releasing hormone agonists for ovarian protection during cancer chemotherapy: systematic review and meta-analysis. Ultrasound in Obstetrics & Gynecology, 2018, 51(1): 77-86.
29 Zheng F, Zhu B, Feng Q, et al. Protective effect of gonadotropin-releasing hormone agonist against chemotherapy-induced ovarian dysfunction: A meta-analysis. Oncology Letters, 2019, 17(6): 5319-5326.
30 Yang B, Shi W, Yang J, et al. Concurrent treatment with gonadotropin-releasing hormone agonists for chemotherapy-induced ovarian damage in premenopausal women with breast cancer: a meta-analysis of randomized controlled trials. The Breast, 2013, 22(2): 150-157.
31 Ben-Aharon I, Gafter-Gvili A, Leibovici L, et al. Pharmacological interventions for fertility preservation during chemotherapy: a systematic review and meta-analysis. Breast cancer research and treatment, 2010, 122: 803-811.

32 Silva C, Caramelo O, Almeida-Santos T, et al. Factors associated with ovarian function recovery after chemotherapy for breast cancer: a systematic review and meta-analysis. Human reproduction, 2016, 31(12): 2737-2749.
33 Kim S S, Lee J R, Jee B C, et al. Use of hormonal protection for chemotherapy-induced gonadotoxicity. Clinical obstetrics and gynecology, 2010, 53(4): 740-752.
34 Shen Y W, Zhang X M, Lv M, et al. Utility of gonadotropin-releasing hormone agonists for prevention of chemotherapy-induced ovarian damage in premenopausal women with breast cancer: a systematic review and meta-analysis. OncoTargets and therapy, 2015: 3349-3359.
35 Senra JC, Roque M, Talim MCT, et al. Gonadotropin-releasing hormone agonists for ovarian protection during cancer chemotherapy: systematic review and meta-analysis. Ultrasound Obstet Gynecol. 2018;51(1):77-86.
36 Wang C, Chen M, Fu F, et al. Gonadotropin-releasing hormone analog cotreatment for the preservation of ovarian function during gonadotoxic chemotherapy for breast cancer: a meta-analysis. PloS one, 2013, 8(6): e66360.
37 Sofiyeva N, Siepmann T, Barlinn K, et al. Gonadotropin-releasing hormone analogs for gonadal protection during gonadotoxic chemotherapy: a systematic review and meta-analysis. Reproductive Sciences, 2019, 26(7): 939-953.
38 White R, Wilson A, Bechman N, et al. Fertility preservation, its effectiveness and its impact on disease status in pre-menopausal women with breast cancer: a systematic review and meta-analysis. European Journal of Obstetrics & Gynecology and Reproductive Biology, 2023, 287: 8-19.
39 Bai F, Lu Y, Wu K, et al. Protecting effects of gonadotropin-releasing hormone agonist on chemotherapy-induced ovarian damage in premenopausal breast cancer patients: a systematic review and meta-analysis. Breast Care, 2017, 12(1): 46-50.
40 Sun X, Dongol S, Jiang J, et al. Protection of ovarian function by GnRH agonists during chemotherapy: a meta-analysis. International Journal of Oncology, 2014, 44(4): 1335-1340.
41 Chen H, Xiao L, Li J, et al.Adjuvant gonadotropin-releasing hormone analogues for the prevention of chemotherapy-induced premature ovarian failure in premenopausal women. Cochrane Database Syst Rev. 2019;3(3):CD008018.
42 Baradwan S, Abuzaid M, Sabban H, et al. Transvaginal needle versus laparoscopic ovarian drilling in hormonal profile and pregnancy outcomes of polycystic ovary syndrome: A systematic review and meta-analysis. Journal of Gynecology Obstetrics and Human Reproduction, 2023, 52(6): 102606.
43 Ding N, Chang J, Jian Q, et al. Luteal phase clomiphene citrate for ovulation induction in women with polycystic ovary syndrome: a systematic review and meta-analysis. Gynecological Endocrinology, 2016, 32(11): 866-871.
44 Segal J B, Blasco-Colmenares E, Norris E J, et al. Preoperative acute normovolemic hemodilution: a meta-analysis. Transfusion, 2004, 44(5): 632-644.
45 Sze Y Y, Berendes S, Russel S, et al. A systematic review of randomised controlled trials of the effects of digital health interventions on postpartum contraception use. BMJ sexual & reproductive health, 2023, 49(1): 50-59.
46 Hu D, Tang Y, Pei K. Strategies for improving postpartum contraception compared with routine maternal care: a systematic review and meta-analysis. International Journal of Public Health, 2023, 68: 1605564.
47 Perez-Lopez F R, Martinez-Dominguez S J, Perez-Roncero G R, et al. Uterine or paracervical lidocaine application for pain control during intrauterine contraceptive device insertion: a meta-analysis of randomised controlled trials. The European Journal of Contraception & Reproductive Health Care, 2018, 23(3): 207-217.

48 Tan L, Liao F, Long L, et al. Essential oils for treating anxiety: a systematic review of randomized controlled trials and network meta-analysis. Frontiers in Public Health, 2023, 11: 1144404.
49 Florou P, Anagnostis P, Theocharis P, et al. Does coenzyme Q 10 supplementation improve fertility outcomes in women undergoing assisted reproductive technology procedures? A systematic review and meta-analysis of randomized-controlled trials. Journal of assisted reproduction and genetics, 2020, 37: 2377-2387.
50 Zhang T, He Q, Xiu H, et al. Efficacy and safety of Coenzyme Q10 supplementation in the treatment of Polycystic Ovary Syndrome: A systematic review and meta-analysis. Reproductive Sciences, 2023, 30(4): 1033-1048.
51 Vitagliano A, Sardo A D S, Saccone G, et al. Endometrial scratch injury for women with one or more previous failed embryo transfers: a systematic review and meta-analysis of randomized controlled trials. Fertility and sterility, 2018, 110(4): 687-702.
52 Iakovidou M C, Kolibianakis E, Zepiridis L, et al. The role of endometrial scratching prior to in vitro fertilization: an updated systematic review and meta-analysis. Reproductive Biology and Endocrinology, 2023, 21(1): 89.
53 Sar-Shalom Nahshon C, Sagi-Dain L, Wiener-Megnazi Z, et al. The impact of intentional endometrial injury on reproductive outcomes: a systematic review and meta-analysis. Human Reproduction Update, 2019, 25(1): 95-113.
54 Lensen S F, Armstrong S, Gibreel A, et al. Endometrial injury in women undergoing in vitro fertilisation (IVF). Cochrane Database Syst Rev. 20216:CD009517.
55 Nahshon C, Sagi-Dain L, Dirnfeld M. The impact of endometrial injury on reproductive outcomes: results of an updated meta-analysis. Reproductive Medicine and Biology, 2020, 19(4): 334-349.
56 Papanikolaou E, Peitsidis N, Tsakiridis I, et al. Endometrial scratching during hysteroscopy in women undergoing in vitro fertilization: a systematic review and meta-analysis. Frontiers in Surgery, 2023, 10: 1225111.
57 Unfer V, Facchinetti F, Orrù B, et al. Myo-inositol effects in women with PCOS: a meta-analysis of randomized controlled trials. Endocrine Connections, 2017, 6(8): 647-658.
58 Pundir J, Psaroudakis D, Savnur P, et al. Inositol treatment of anovulation in women with polycystic ovary syndrome: a meta-analysis of randomised trials. BJOG: An International Journal of Obstetrics & Gynaecology, 2018, 125(3): 299-308.
59 Zarezadeh M, Dehghani A, Faghfouri A H, et al. Inositol supplementation and body mass index: A systematic review and meta-analysis of randomized clinical trials. Obesity Science & Practice, 2022, 8(3): 387-397.
60 Jethaliya H, Gajjar N, Patel V, et al. Efficacy of Myo-inositol on anthropometric, metabolic, and endocrine outcomes in PCOS patients: a meta-analysis of randomized controlled trial. Reproductive Sciences, 2022, 29(8): 2282-2298.
61 Xing X, Guo J, Mo J, et al. Qili Qiangxin capsules for chronic heart failure: A GRADE-assessed clinical evidence and preclinical mechanism. Frontiers in Cardiovascular Medicine, 2023, 9: 1090616.
62 Wang H, Zhang L, Zhang Z, et al. Perioperative sleep disturbances and postoperative delirium in adult patients: a systematic review and meta-analysis of clinical trials. Frontiers in Psychiatry, 2020, 11: 570362.

63 Chen R, Sun S, Li Y, et al. Efficacy and safety evaluation of dexmedetomidine for postoperative patient controlled intravenous analgesia: A systematic review and meta-analysis. Frontiers in Pharmacology, 2022, 13: 1028704.
64 Govêia C S, Miranda D B, Oliveira L V B, et al. Dexmedetomidine reduces postoperative cognitive and behavioral dysfunction in adults submitted to general anesthesia for non-cardiac surgery: meta-analysis of randomized clinical trials. Brazilian Journal of Anesthesiology, 2021, 71: 413-420.
65 Xiao Y, Tang L, Chen N. The effects of dexmedetomidine on postoperative sleep in elderly patients: a systematic review and meta-analysis. Journal of Anesthesia and Translational Medicine, 2023, 2(3): 11-20.
66 Tang X, Li J, Yang B, et al. Efficacy of sleep interventions on postoperative delirium: a systematic review and meta-analysis of randomized controlled trials. Anesthesiology and Perioperative Science, 2023, 1(4): 29.
67 Yang Y L, Hu B J, Yi J, et al. Effects of dexmedetomidine on cardioprotection and other postoperative complications in elderly patients after cardiac and non-cardiac surgerie. World Journal of Meta-Analysis, 2022, 10(1): 25-36.
68 Bellos I, Pergialiotis V. Tranexamic acid for the prevention of postpartum hemorrhage in women undergoing cesarean delivery: an updated meta-analysis. American Journal of Obstetrics and Gynecology, 2022, 226(4): 510-523. e22.
69 Yang F, Wang H, Shen M. Effect of preoperative prophylactic intravenous tranexamic acid on perioperative blood loss control in patients undergoing cesarean delivery: a systematic review and meta-analysis. BMC Pregnancy and Childbirth, 2023, 23(1): 420.
70 Cheema H A, Ahmad A B, Ehsan M, et al. Tranexamic acid for the prevention of blood loss after cesarean section: an updated systematic review and meta-analysis of randomized controlled trials. American Journal of Obstetrics & Gynecology MFM, 2023, 5(8): 101049.
71 Al-Dardery N M, Abdelwahab O A, Abouzid M, et al. Efficacy and safety of tranexamic acid in prevention of postpartum hemorrhage: a systematic review and meta-analysis of 18,649 patients. BMC Pregnancy and Childbirth, 2023, 23(1): 817.
72 Lee A, Wang M Y F, Roy D, et al. Prophylactic tranexamic acid prevents postpartum hemorrhage and transfusions in cesarean deliveries: a systematic review and meta-analysis. American Journal of Perinatology, 2024, 41(S 01): e2254-e2268.
73 Yuldasheva A, Omarova G, Begniyazova Z, et al. Comparison of different cesarean delivery techniques: A systematic review and meta-analysis. Electronic Journal of General Medicine, 2023, 20(6).
74 Yu Y, Xiao W, Du L Y, et al. Acupuncture for dyspnea and breathing physiology in chronic respiratory diseases: A systematic review and meta-analysis of randomized controlled trials. Heliyon, 2024, 10(10).
75 Zhang D, Sun Y, Li J. Application of dexmedetomidine in epidural labor analgesia: a systematic review and meta-analysis on randomized controlled trials. The Clinical journal of pain, 2024, 40(1): 57-65.
76 Holzer K J, Bollepalli H, Carron J, et al. The impact of compassion-based interventions on perioperative anxiety and depression: A systematic review and meta-analysis. J Affect Disord. 2024;365:476-491.
77 Fan S, Cheng X, Wang X, et al. Bronchial artery embolization versus conservative treatment for hemoptysis: a systematic review and meta-analysis. BMC Pulmonary Medicine, 2024, 24(1): 428.
78 Gong Y, Jiang T, He H, et al. Effects of carnitine on glucose and lipid metabolic profiles and fertility outcomes in women with polycystic ovary syndrome: A systematic review and meta-analysis. Clinical Endocrinology, 2023, 98(5): 682-691.

79 Fernandez T, Vinuela M, Vidal C, et al. Lifestyle changes in patients with non-alcoholic fatty liver disease: A systematic review and meta-analysis. PloS one, 2022, 17(2): e0263931.
80 Battista F, Ermolao A, van Baak M A, et al. Effect of exercise on cardiometabolic health of adults with overweight or obesity: Focus on blood pressure, insulin resistance, and intrahepatic fat—A systematic review and meta-analysis. Obesity Reviews, 2021, 22: e13269.
81 Liu Y, Xie W, Li J, et al. Effects of aerobic exercise on metabolic indicators and physical performance in adult NAFLD patients: A systematic review and network meta-analysis. Medicine, 2023, 102(14): e33147.
82 Zeng Y, Zhang X, Luo W, et al. Effect of exercise intervention on clinical parameters in patients with non-alcoholic fatty liver disease and type 2 diabetes mellitus: a meta-analysis of randomized controlled trials. European Journal of Gastroenterology & Hepatology, 2024, 36(1): 1-12.
83 Abe M, Arima H, Yoshida Y, et al. Optimal blood pressure target to prevent severe hypertension in pregnancy: a systematic review and meta-analysis. Hypertension Research, 2022, 45(5): 887-899.
84 Maged A M, Wali A A, Metwally A A, et al. The efficacy of misoprostol in reducing intraoperative blood loss in women undergoing elective cesarean section. A systematic review and meta-analysis. Journal of Obstetrics and Gynaecology Research, 2022, 48(8): 2038-2070.
85 Khorshidi M, Zarezadeh M, Moradi Moghaddam O, et al. Effect of evening primrose oil supplementation on lipid profile: A systematic review and meta-analysis of randomized clinical trials. Phytotherapy Research, 2020, 34(10): 2628-2638.
86 Avelino C M S F, Araújo R F F. Effects of vitamin D supplementation on oxidative stress biomarkers of Iranian women with polycystic ovary syndrome: a meta-analysis study. Revista Brasileira de Ginecologia e Obstetrícia, 2024, 46: e-rbgo37.
87 Luo J, Li T, Yuan J. Effectiveness of vitamin D supplementation on lipid profile in polycystic ovary syndrome women: a meta-analysis of randomized controlled trials. Annals of Palliative Medicine, 2021, 10(1): 11429-11129.
88 He J, Lei X C, Deng R, et al. Efficacy of antioxidant supplementation in improving endocrine, hormonal, inflammatory, and metabolic statuses of PCOS: a meta-analysis and systematic review. Food Funct. 2024;15(4):1779-1802.
89 Aman A, Salim B, Munshi K, et al. Effect on neonatal outcome of pharmacological interventions for attenuation of the maternal haemodynamic response to tracheal intubation: a systematic review. Anaesthesia and intensive care, 2018, 46(3): 258-271.
90 Gobble R M, Hoang H L T, Kachniarz B, et al. Ketorolac does not increase perioperative bleeding: a meta-analysis of randomized controlled trials. Plastic and reconstructive Surgery, 2014, 133(3): 741-755.
91 Bongiovanni T, Lancaster E, Ledesma Y, et al. Systematic review and meta–analysis of the association between non–steroidal anti–inflammatory drugs and operative bleeding in the perioperative period. Journal of the American College of Surgeons, 2021, 232(5): 765-790. e1.
92 Zeng A M, Nami N F, Wu C L, et al. The analgesic efficacy of nonsteroidal anti-inflammatory agents (NSAIDs) in patients undergoing cesarean deliveries: a meta-analysis. Regional Anesthesia & Pain Medicine, 2016, 41(6): 763-772.
93 Giudice A L, Asmundo M G, Cimino S, et al. Effects of Physical Activity on Fertility Parameters: A Meta-Analysis of Randomized Controlled Trials. The World Journal of Men's Health, 2024, 42(3): 555.
94 Nunan D, Cai T, Gardener A D, et al. Physical activity for treatment of irritable bowel syndrome. Cochrane Database Syst Rev. 2022(6):CD011497.

95 Liu W, Meng Z, Wang G. The efficacy of nitrates for Bone health: a systematic review and Meta-analysis of observational and randomized controlled studies. Frontiers in Endocrinology, 2022, 13: 833932.
96 Aubin A M, McAuliffe L, Williams K, et al. Combined vaginal progesterone and cervical cerclage in the prevention of preterm birth: a systematic review and meta-analysis. American Journal of Obstetrics & Gynecology MFM, 2023, 5(8): 101024.
97 Wennerholm U B, Bergman L, Kuusela P, et al. Progesterone, cerclage, pessary, or acetylsalicylic acid for prevention of preterm birth in singleton and multifetal pregnancies–A systematic review and meta-analyses. Frontiers in medicine, 2023, 10: 1111315.
98 Frauenknecht J, Kirkham K R, Jacot-Guillarmod A, et al. Analgesic impact of intra-operative opioids vs. opioid-free anaesthesia: a systematic review and meta-analysis. Anaesthesia, 2019, 74(5): 651-662.
99 Lee H S, Yoon H Y, Jin H J, et al. Can dexmedetomidine influence recovery profiles from general anesthesia in nasal surgery?. Otolaryngology–Head and Neck Surgery, 2018, 158(1): 43-53.
100 Fan X, Cai H, Pan B, et al. Comparison of dexmedetomidine and remifentanil on reducing coughing during emergence from anesthesia with tracheal intubation: A meta-analysis. Frontiers in Pharmacology, 2022, 13: 993239.
101 Janipour M, Bastaninejad S, Amali A, et al. Dexmedetomidine versus remifentanil in nasal surgery: a systematic review and meta-analysis. BMC anesthesiology, 2024, 24(1): 1-15.
102 Zhang J, Yu Y, Miao S, et al. Effects of peri-operative intravenous administration of dexmedetomidine on emergence agitation after general anesthesia in adults: a meta-analysis of randomized controlled trials. Drug design, development and therapy, 2019: 2853-2864.
103 Grape S, Kirkham K R, Frauenknecht J, et al. Intra-operative analgesia with remifentanil vs. dexmedetomidine: a systematic review and meta-analysis with trial sequential analysis. Anaesthesia, 2019, 74(6): 793-800.
104 Sin J C K, Tabah A, Campher M J J, et al. The effect of dexmedetomidine on postanesthesia care unit discharge and recovery: a systematic review and meta-analysis. Anesthesia & Analgesia, 2022, 134(6): 1229-1244.
105 Jin C, Cheng Y, Sun Y. The effects of continuous intravenous infusion of dexmedetomidine and remifentanil on postoperative pain: a systematic review and meta-analysis. Int J Clin Exp Med, 2019, 12(1): 1165-1178.
106 Guay J, Kopp S. Epidural analgesia for adults undergoing cardiac surgery with or without cardiopulmonary bypass. Cochrane Database Syst Rev. 2019 (3):CD006715.
107 Chiew J K, Low C J W, Zeng K, et al. Thoracic epidural anesthesia in cardiac surgery: a systematic review, meta-analysis, and trial sequential analysis of randomized controlled trials. Anesthesia & Analgesia, 2023, 137(3): 587-600.
108 Mustafa M S, Shafique M A, Zaidi S D E Z, et al. Preoperative anxiety management in pediatric patients: a systemic review and meta-analysis of randomized controlled trials on the efficacy of distraction techniques. Frontiers in Pediatrics, 2024, 12: 1353508.
109 Chen G, Wu M, Chen J, et al. Biomarkers associated with functional improvement after stroke rehabilitation: a systematic review and meta-analysis of randomized controlled trials. Frontiers in Neurology, 2023, 14: 1241521.
110 Eskew A M, Bedrick B S, Hardi A, et al. Letrozole compared with clomiphene citrate for unexplained infertility: a systematic review and meta-analysis. Obstetrics & Gynecology, 2019, 133(3): 437-444.

111 Lopez L M, Bernholc A, Zeng Y, et al. Interventions for pain with intrauterine device insertion. Cochrane Database Syst Rev. 2015(7):CD007373.
112 Masoud A T, Samy A, Abdelmageed H G, et al. Efficacy and safety of misoprostol for intrauterine device insertion in women with no previous vaginal delivery: a systematic review and meta-analysis of randomized controlled trials. Proceedings in Obstetrics and Gynecology, 2020, 10(1).
113 Kulier R, Kapp N, Gülmezoglu A M, et al. Medical Methods for First-Trimester Abortion. Am Fam Physician. 2023 Jan;107(1):24-25.
114 Christelle K, Norhayati M N, Jaafar S H. Interventions to prevent or treat heavy menstrual bleeding or pain associated with intrauterine-device use. Cochrane Database Syst Rev. 2022(8):CD006034.
115 Kamper S J, Apeldoorn A T, Chiarotto A, et al. Multidisciplinary biopsychosocial rehabilitation for chronic low back pain: Cochrane systematic review and meta-analysis. Bmj, 2015, 350.
116 Kamper SJ, Apeldoorn AT, Chiarotto A, et al. Multidisciplinary biopsychosocial rehabilitation for chronic low back pain. Cochrane Database Syst Rev. 2014(9):CD000963.
117 Yang J, Lo W L A, Zheng F, et al. Evaluation of Cognitive Behavioral Therapy on Improving Pain, Fear Avoidance, and Self-Efficacy in Patients with Chronic Low Back Pain: A Systematic Review and Meta-Analysis. Pain Research and Management, 2022, 2022(1): 4276175.
118 Richmond H, Hall A M, Copsey B, et al. The effectiveness of cognitive behavioural treatment for non-specific low back pain: a systematic review and meta-analysis. PloS one, 2015, 10(8): e0134192.
119 Petrucci G, Papalia G F, Russo F, et al. Psychological approaches for the integrative care of chronic low back pain: A systematic review and metanalysis. International journal of environmental research and public health, 2021, 19(1): 60.
120 Hochheim M, Ramm P, Amelung V. The effectiveness of low-dosed outpatient biopsychosocial interventions compared to active physical interventions on pain and disability in adults with nonspecific chronic low back pain: A systematic review with meta-analysis. Pain Practice, 2023, 23(4): 409-436.
121 Fadli A, Prasetya H, Kristyanto A. Effectivity biopsychosocial intervention with cognitive behavioral therapy and exercise therapy program in chronic low back pain: Meta-analysis. Indonesian Journal of Medicine, 2021, 6(2): 177-193.
122 Nawras M, Beran A, Yazdi V, et al. Phosphodiesterase inhibitor and selective serotonin reuptake inhibitor combination therapy versus monotherapy for the treatment of major depressive disorder: a systematic review and meta-analysis. International Clinical Psychopharmacology, 2023, 38(4): 261-268.
123 Patel A N, Varma J, Ganguly B. Evidence of the Immunomodulatory Effects of Selective Serotonin Reuptake Inhibitors in Patients With Depression Through a Systematic Review. Cureus, 2024, 16(6): e62991.
124 Alhamdah Y, Li W Y, Nagappa M, et al. Perioperative approaches to prevent delayed neurocognitive recovery and postoperative neurocognitive disorder in older surgical patients: A systematic review and meta-analysis of randomized controlled trials. Journal of Anaesthesiology Clinical Pharmacology, 2024: 10.4103.
125 Wang D, Liu Z, Zhang W, et al. Intravenous infusion of dexmedetomidine during the surgery to prevent postoperative delirium and postoperative cognitive dysfunction undergoing non-cardiac surgery: a meta-analysis of randomized controlled trials. European Journal of Medical Research, 2024, 29(1): 239.

126 Du D, Yuan Y D. Efficacy and safety of macitentan for pulmonary hypertension: A meta-analysis. The Clinical Respiratory Journal, 2023, 17(11): 1117-1129.
127 Kalra R, Duval S, Thenappan T, et al. Comparison of balloon pulmonary angioplasty and pulmonary vasodilators for inoperable chronic thromboembolic pulmonary hypertension: a systematic review and meta-analysis. Scientific reports, 2020, 10(1): 8870.
128 Wang G, Qin J, Han D. Long-term safety of macitentan in patients with pulmonary hypertension: A meta-analysis of randomised controlled trials. European Journal of Clinical Investigation, 2023, 53(11): e14059.
129 Qin J, Wang G, Han D. Macitentan in Patients with Pulmonary Hypertension: A Systematic Review and Meta-Analysis of Randomized Controlled Trials. Glob Heart. 2023;18(1):58.
130 Zhang J, Li J M, Huang Z S, et al. A meta-analysis of randomized controlled trials in targeted treatments of chronic thromboembolic pulmonary hypertension. The Clinical Respiratory Journal, 2019, 13(7): 467-479.
131 Abu-Zaid A, Alshahrani M S, Al-Matary A, et al. Isosorbide mononitrate for cervical ripening during labour induction: A systematic review and meta-analysis of 23 randomized controlled trials. European Journal of Obstetrics & Gynecology and Reproductive Biology, 2022, 276: 38-46.
132 Greff D, Juhász A E, Váncsa S, et al. Inositol is an effective and safe treatment in polycystic ovary syndrome: a systematic review and meta-analysis of randomized controlled trials. Reproductive Biology and Endocrinology, 2023, 21(1): 10.
133 Moulton C D, Hopkins C W P, Ismail K, et al. Repositioning of diabetes treatments for depressive symptoms: a systematic review and meta-analysis of clinical trials. Psychoneuroendocrinology, 2018, 94: 91-103.
134 Magzoub R, Kheirelseid E A H, Perks C, et al. Does metformin improve reproduction outcomes for non-obese, infertile women with polycystic ovary syndrome? Meta-analysis and systematic review. European Journal of Obstetrics & Gynecology and Reproductive Biology, 2022, 271: 38-62.
135 Song Z, Shi S, Zhang Y. Ivermectin for treatment of COVID-19: A systematic review and meta-analysis. Heliyon. 2024 Mar 11;10(6):e27647
136 Gattas D J, Dan A, Myburgh J, et al. Fluid resuscitation with 6% hydroxyethyl starch (130/0.4 and 130/0.42) in acutely ill patients: systematic review of effects on mortality and treatment with renal replacement therapy. Intensive care medicine, 2013, 39: 558-568.
137 McNicol E D, Ferguson M K C. Patient controlled opioid analgesia versus non-patient controlled opioid analgesia for postoperative pain. Cochrane Database Syst Rev. 2015(6):CD003348
138 Hudcova J, McNicol E, Quah C, et al. Patient controlled intravenous opioid analgesia versus conventional opioid analgesia for postoperative pain control: A quantitative systematic review. Acute Pain, 2005, 7(3): 115-132.
139 Kelly B, Ng S H, Carrad A, et al. The Potential Effectiveness of Front-of-Pack Nutrition Labeling for Improving Population Diets. Annual Review of Nutrition, 2024, 44.
140 Rakhanova Y, Almawi W Y, Aimagambetova G, et al. The effects of sildenafil citrate on intrauterine growth restriction: a systematic review and meta-analysis. BMC Pregnancy and Childbirth, 2023, 23(1): 409.
141 Liu Y, Un E M W, Bai Y, et al. Safety and Efficacy of phosphodiesterase-5 (PDE-5) inhibitors in fetal growth restriction: a systematic literature review and meta-analysis. Journal of Pharmacy & Pharmaceutical Sciences, 2024, 27: 13206.

142 Damghanian M, Farnam F, Kharaghani R. The Effects of Sildenafil on Fetal Doppler Indices: A Systematic Review and Meta-Analysis. Journal of Advances in Medical and Biomedical Research, 2020, 28(131): 307-315.
143 Derakhshi F, Abbasalizadeh F. The Effect of Sildenafil Citrate on Fetal and Maternal Ultrasound Indices in IUGR-complicated Pregnancies; A Systematic Review and Meta-analysis of Randomized Clinical Trials. 2022.
144 Cozzolino M, Vitagliano A, Pellegrini L, et al. Therapy with probiotics and synbiotics for polycystic ovarian syndrome: a systematic review and meta-analysis. European Journal of Nutrition, 2020, 59: 2841-2856.
145 Heshmati J, Farsi F, Yosaee S, et al. The effects of probiotics or synbiotics supplementation in women with polycystic ovarian syndrome: a systematic review and meta-analysis of randomized clinical trials. Probiotics and Antimicrobial Proteins, 2019, 11: 1236-1247.
146 Li Y, Tan Y, Xia G, et al. Effects of probiotics, prebiotics, and synbiotics on polycystic ovary syndrome: a systematic review and meta-analysis. Critical Reviews in Food Science and Nutrition, 2023, 63(4): 522-538.
147 Tabrizi R, Ostadmohammadi V, Akbari M, et al. The effects of probiotic supplementation on clinical symptom, weight loss, glycemic control, lipid and hormonal profiles, biomarkers of inflammation, and oxidative stress in women with polycystic ovary syndrome: a systematic review and meta-analysis of randomized controlled trials. Probiotics and antimicrobial proteins, 2019: 1-14.
148 Ghorbani Z, Kazemi A, UP Bartolomaeus T, et al. The effect of probiotic and synbiotic supplementation on lipid parameters among patients with cardiometabolic risk factors: a systematic review and meta-analysis of clinical trials. Cardiovascular Research, 2023, 119(4): 933-956.
149 Zhang C, Sheng Y, Jiang J, et al. Probiotics supplementation for management of type II diabetes risk factors in adults with polycystic ovarian syndrome: a meta-analysis of randomized clinical trial. Food Science and Human Wellness, 2023, 12(4): 1053-1063.
150 Romero R, Conde-Agudelo A, Da Fonseca E, et al. Vaginal progesterone for preventing preterm birth and adverse perinatal outcomes in singleton gestations with a short cervix: a meta-analysis of individual patient data. American journal of obstetrics and gynecology, 2018, 218(2): 161-180.
151 Romero R, Nicolaides K, Conde-Agudelo A, et al. Vaginal progesterone in women with an asymptomatic sonographic short cervix in the midtrimester decreases preterm delivery and neonatal morbidity: a systematic review and metaanalysis of individual patient data. American journal of obstetrics and gynecology, 2012, 206(2): 124. e1-124. e19.
152 Romero R, Nicolaides K H, Conde-Agudelo A, et al. Vaginal progesterone decreases preterm birth≤ 34 weeks of gestation in women with a singleton pregnancy and a short cervix: an updated meta-analysis including data from the OPPTIMUM study. Ultrasound in Obstetrics & Gynecology, 2016, 48(3): 308-317.
153 Dodd J M, Jones L, Flenady V, et al. Prenatal administration of progesterone for preventing preterm birth in women considered to be at risk of preterm birth. Cochrane Database Syst Rev.2013(7):CD004947.
154 Jarde A, Lutsiv O, Park C K, et al. Preterm birth prevention in twin pregnancies with progesterone, pessary, or cerclage: a systematic review and meta-analysis. BJOG: An International Journal of Obstetrics & Gynaecology, 2017, 124(8): 1163-1173.
155 Conde-Agudelo A, Romero R. Does vaginal progesterone prevent recurrent preterm birth in women with a singleton gestation and a history of spontaneous preterm birth? Evidence from a systematic review and meta-analysis. American journal of obstetrics and gynecology, 2022, 227(3): 440-461. e2.

156 Conde-Agudelo A, Rehal A, Da Fonseca E, et al. Vaginal progesterone for the prevention of preterm birth and adverse perinatal outcomes in twin gestations with a short cervix: an updated individual patient data meta-analysis. Ultrasound in obstetrics & gynecology: the official journal of the International Society of Ultrasound in Obstetrics and Gynecology, 2022, 59(2): 263.

157 Conde-Agudelo A, Romero R, Rehal A, et al. Vaginal progesterone for preventing preterm birth and adverse perinatal outcomes in twin gestations: a systematic review and meta-analysis. American Journal of Obstetrics and Gynecology, 2023, 229(6): 599-616. e3.

158 Phung J, Williams KP, McAullife L, et al. Vaginal progesterone for prevention of preterm birth in asymptomatic high-risk women with a normal cervical length: a systematic review and meta-analysis. J Matern Fetal Neonatal Med. 2022 Dec;35(25):7093-7101.

159 Chaman-Ara K, Bahrami M A, Bahrami E, et al. Efficacy of progesterone therapy in the prevention of preterm labor in women with mixed risk-factors: A systematic review and meta-analysis of randomized clinical trials. Journal of Clinical Practice and Research, 2016, 38(2): 48.

160 Hwang S, Song C S. Assistive technology involving postural control and gait performance for adults with stroke: A systematic review and meta-analysis. Healthcare. MDPI, 2023, 11(15): 2225.

161 Nam J H, Kim H. How assistive devices affect activities of daily living and cognitive functions of people with brain injury: a meta-analysis. Disability and Rehabilitation: Assistive Technology, 2018, 13(3): 305-311.

162 Lee S Y, Jung S H, Lee S U, et al. Effect of balance training after hip fracture surgery: a systematic review and meta-analysis of randomized controlled studies. The Journals of Gerontology: Series A, 2019, 74(10): 1679-1685.

163 Hulsbæk S, Juhl C, Røpke A, et al. Exercise therapy is effective at improving short-and long-term mobility, activities of daily living, and balance in older patients following hip fracture: a systematic review and meta-analysis. The Journals of Gerontology: Series A, 2022, 77(4): 861-871.

164 Beckmann M, Bruun-Olsen V, Pripp A H, et al. Effect of exercise interventions in the early phase to improve physical function after hip fracture–a systematic review and meta-analysis. Physiotherapy, 2020, 108: 90-97.

165 Wu J, Mao L, Wu J. Efficacy of balance training for hip fracture patients: a meta-analysis of randomized controlled trials. Journal of orthopaedic surgery and research, 2019, 14: 1-11.

166 Fairhall N J, Dyer S M, Mak J C, et al. Interventions for improving mobility after hip fracture surgery in adults. Cochrane Database Syst Rev. 2022(9):CD001704.

167 Chen X, Yang W, Wang X. Balance training can enhance hip fracture patients' independence in activities of daily living: a meta-analysis of randomized controlled trials. Medicine, 2020, 99(16): e19641.

168 Chang H, Luan C, Li C. Effect of Comprehensive Rehabilitation Training Based on Balance Function on Postoperative Recovery and Function of Hip Fracture in the Elderly: A Systematic Review and Meta-Analysis. Geriatric orthopaedic surgery & rehabilitation, 2024, 15: 21514593241261506.

169 Wu J Q, Mao L B, Wu J. Efficacy of exercise for improving functional outcomes for patients undergoing total hip arthroplasty: A meta-analysis. Medicine, 2019, 98(10): e14591.

170 Saueressig T, Owen P J, Zebisch J, et al. Evaluation of exercise interventions and outcomes after hip arthroplasty: a systematic review and meta-analysis. JAMA network open, 2021, 4(2): e210254-e210254.

171 Tugni C, Sansoni J, Vanacore N, et al. Rehabilitation effects in patients with total hip replacement: a systematic review and meta-analysis. Minerva Ortopedica e Traumatologica, 2019, 70(4): 205-218.
172 Huang L, Han W, Qi W, et al. Early unrestricted vs. partial weight bearing after uncemented total hip arthroplasty: a systematic review and meta-analysis. Frontiers in Surgery, 2023, 10: 1225649.
173 Tang W, Flavell C A, Grant A, et al. The effects of exercise on function and pain following total hip arthroplasty: a systematic literature review and meta-analysis. Physical Therapy Reviews, 2022, 27(4): 247-266.
174 Mu F, Yang X, Wang M, et al. Effect of Granulocyte Colony-Stimulating Factor on Clinical Pregnancy Outcomes of Recurrent Miscarriage and Recurrent Implantation Failure Cases: A Meta-Analysis. 2023.
175 Conforti A, Longobardi S, Carbone L, et al. Does intrauterine injection of hCG improve IVF outcome? A systematic review and a meta-analysis. International Journal of Molecular Sciences, 2022, 23(20): 12193.
176 Luo X, Wu Y, Xu Y, et al. Meta-analysis of intrauterine hCG perfusion efficacy in recurrent implantation failure as defined by ESHRE guidelines. BMC Pregnancy and Childbirth, 2024, 24(1): 468.
177 Xie Q, Quan X, Lan Y, et al. Uterine infusion strategies for infertile patients with recurrent implantation failure: a systematic review and network meta-analysis. Reproductive Biology and Endocrinology, 2024, 22(1): 44.
178 Su Q, Pan Z, Yin R, et al. The value of G-CSF in women experienced at least one implantation failure: a systematic review and meta-analysis. Frontiers in Endocrinology, 2024, 15: 1370114.
179 Wang Y, Ji R, Yu B. Assessing the anti-inflammatory effects of whole-body vibration: a meta-analysis based on pre-clinical and clinical evidences. American Journal of Clinical and Experimental Immunology, 2024, 13(3): 68.
180 Wei Y, Wang S. Comparison of emergency cervical cerclage and expectant treatment in cervical insufficiency in singleton pregnancy: A meta-analysis. PLoS One, 2023, 18(2): e0278342.
181 Hulshoff C C, Bosgraaf R P, Spaanderman M E A, et al. The efficacy of emergency cervical cerclage in singleton and twin pregnancies: a systematic review with meta-analysis. American Journal of Obstetrics & Gynecology MFM, 2023, 5(7): 100971.
182 Pilarski N, Bhogal G, Hamer J, et al. Interventions for women with premature cervical dilatation and exposed fetal membranes to prevent pregnancy loss and preterm birth–A systematic review and meta-analysis. European Journal of Obstetrics & Gynecology and Reproductive Biology, 2024.
183 Jafarabadi M A, Dehghani A, Khalili L, et al. A meta-analysis of randomized controlled trials of the effect of probiotic food or supplement on glycemic response and body mass index in patients with type 2 diabetes, updating the evidence. Current diabetes reviews, 2021, 17(3): 356-364.
184 Naseri K, Saadati S, Yari Z, et al. Beneficial effects of probiotic and synbiotic supplementation on some cardiovascular risk factors among individuals with prediabetes and type 2 diabetes mellitus: A grade-assessed systematic review, meta-analysis, and meta-regression of randomized clinical trials. Pharmacological research, 2022, 182: 106288.
185 Soltani S, Ashoori M, Dehghani F, et al. Effects of probiotic/synbiotic supplementation on body weight in patients with diabetes: a systematic review and meta-analyses of randomized-controlled trials. BMC endocrine disorders, 2023, 23(1): 86.

186 Qiu X, Wu Q, Li W, et al. Effects of Lactobacillus supplementation on glycemic and lipid indices in overweight or obese adults: A systematic review and meta-analysis. Clinical Nutrition, 2022, 41(8): 1787-1797.
187 Gohari S, Ismail-Beigi F, Mahjani M, et al. The effect of sodium-glucose co-transporter-2 (SGLT2) inhibitors on blood interleukin-6 concentration: a systematic review and meta-analysis of randomized controlled trials. BMC Endocrine Disorders, 2023, 23(1): 257.
188 Huang Y, Xu X, Liu J, et al. Effects of new hypoglycemic drugs on cardiac remodeling: a systematic review and network meta-analysis. BMC Cardiovascular Disorders, 2023, 23(1): 293.
189 Hu C, Ou S, Zheng X, et al. Effect of SGLT2 inhibitors on heart failure outcomes and cardiovascular death across the cardiometabolic disease spectrum: a systematic review and meta-analysis. Lancet Diabetes Endocrinol. 2024;12(7):447-461.
190 Schnabel A, Meyer-Frießem C H, Reichl S U, et al. Is intraoperative dexmedetomidine a new option for postoperative pain treatment? A meta-analysis of randomized controlled trials. PAIN®, 2013, 154(7): 1140-1149.
191 Geng J, Qian J, Cheng H, et al. The influence of perioperative dexmedetomidine on patients undergoing cardiac surgery: a meta-analysis. PLoS One, 2016, 11(4): e0152829.
192 Pasin L, Greco T, Feltracco P, et al. Dexmedetomidine as a sedative agent in critically ill patients: a meta-analysis of randomized controlled trials. PLoS One, 2013, 8(12): e82913.
193 Poon W H, Ling R R, Yang I X, et al. Dexmedetomidine for adult cardiac surgery: a systematic review, meta-analysis and trial sequential analysis. Anaesthesia, 2023, 78(3): 371-380.
194 Le Bot A, Michelet D, Hilly J, et al. Efficacy of intraoperative dexmedetomidine compared with placebo for surgery in adults: a meta-analysis of published studies. Minerva anestesiologica, 2015, 81(10): 1105-1117.
195 Peng K, Ji F, Liu H, et al. Effects of perioperative dexmedetomidine on postoperative mortality and morbidity: a systematic review and meta-analysis. Clinical Therapeutics, 2019, 41(1): 138-154. e4.
196 Ng K T, Shubash C J, Chong J S. The effect of dexmedetomidine on delirium and agitation in patients in intensive care: systematic review and meta-analysis with trial sequential analysis. Anaesthesia, 2019, 74(3): 380-392.
197 Liu H T, Deng N H, Wu Z F, et al. Statin's role on blood pressure levels: Meta-analysis based on randomized controlled trials. The Journal of Clinical Hypertension, 2023, 25(3): 238-250.
198 Beswick A D, Wylde V, Bertram W, et al. The effectiveness of non-pharmacological sleep interventions for improving inpatient sleep in hospital: a systematic review and meta-analysis. Sleep Medicine, 2023, 107: 243-267.
199 Wang H, Su W, Lowe S, et al. Association of apatinib and breast cancer: A systematic review and meta-analysis. Surgical Oncology, 2022, 44: 101818.
200 Zhang Y, Qi L, Wang R. Meta-analysis: reducing the recurrence rate of allergic rhinitis through oral administration of traditional Chinese medicine. European Review for Medical & Pharmacological Sciences, 2023, 27(17).
201 Wang X, Ge L, Hu H, et al. Effects of non-invasive brain stimulation on post-stroke spasticity: a systematic review and meta-analysis of randomized controlled trials. Brain sciences, 2022, 12(7): 836.

202 Tao J, Yan Z, Bai G, et al. Enhanced recovery after surgery rehabilitation protocol in the perioperative period of orthopedics: a systematic review. Journal of Personalized Medicine, 2023, 13(3): 421.
203 Tan P, Huo M, Zhou X, et al. The safety and effectiveness of enhanced recovery after surgery (ERAS) in older patients undergoing orthopedic surgery: a systematic review and meta-analysis. Archives of Orthopaedic and Trauma Surgery, 2023, 143(11): 6535-6545.
204 Aljohani K A, Fadlalmola H A, Fadila D E S. Effects of the Nurse-led Program on Disability Improvement in Patients with Stroke: A Systematic Review and Meta-analysis. Journal of Disability Research, 2024, 3(2): 20240014.
205 Gao J, Zhang X, Xu M, et al. The efficacy and safety of sacubitril/valsartan compared with ACEI/ARB in the treatment of heart failure following acute myocardial infarction: a systematic review and meta-analysis of randomized controlled trials. Frontiers in Pharmacology, 2023, 14: 1237210.
206 Wang F, Li C, Zhang X. Sacubitril/valsartan improves the prognosis of acute myocardial infarction: a meta-analysis. Coronary Artery Disease, 2024, 35(3): 231-238.
207 Liu Y Y, Zhao Y, Yin Y Y, et al. Effects of transitional care interventions on quality of life in people with lung cancer: A systematic review and meta-analysis. Journal of Clinical Nursing, 2024, 33(5): 1976-1994.
208 Kim S Y, Jo H Y, Na H S, et al. The effect of peripheral nerve block on postoperative delirium in older adults undergoing hip surgery: a systematic review and meta-analysis of randomized controlled trials. Journal of Clinical Medicine, 2023, 12(7): 2459.
209 Safiejko K, Pedziwiatr M, Pruc M, et al. Robotic versus Laparoscopic Liver Resections for Colorectal Metastases: A Systematic Review and Meta-Analysis. Cancers, 2024, 16(8): 1596.
210 Zhang M, Meng S Q, Hasan A J, et al. Network meta-analysis of the effectiveness of different interventions for internet addiction in college students. J Affect Disord. 2024;363:26-38.
211 Sun D, Zhang X, Xu Q, et al. Duhamel and transanal endorectal pull-throughs for Hirschsprung disease: a Bayesian network meta-analysis. BMC surgery, 2024, 24(1): 132.
212 Wei Q, Li M, Jiang Y, et al. Efficacy of perioperatively application of ketamine on postoperative depressive symptoms in adult patients: A systematic review and meta-analysis with trial sequential analysis. J Affect Disord. 2024;353:27-35
213 Parsaei M, Hasehmi S M, Seyedmirzaei H, et al. Perioperative esketamine administration for prevention of postpartum depression after the cesarean section: A systematic review and meta-analysis. J Affect Disord. 2024;361:564-580.
214 Li S, Zhou W, Li P, et al. Effects of ketamine and esketamine on preventing postpartum depression after cesarean delivery: A meta-analysis. J Affect Disord. 2024;351:720-728.
215 Ma S, Dou Y, Wang W, et al. Association between esketamine interventions and postpartum depression and analgesia following cesarean delivery: a systematic review and meta-analysis. American Journal of Obstetrics & Gynecology MFM, 2024, 6(3): 101241.
216 Wang J, Liu H, Wei X. Effect of Intraoperative and/or Postoperative Esketamine Administration on Preventing Postpartum Depression: A Systematic Review and Meta-Analysis. Psychiatry Research, 2024: 115890.
217 Yu L, Wang Y, Ma D, et al. In-hospital nursing care intervention increasing the effect of vacuum sealing drainage on wound healing: a meta-analysis. International wound journal, 2023, 20(8): 3371-3379.

218 Abuelazm M T, Ghanem A, Johanis A, et al. Reno-protective effects of perioperative dexmedetomidine in kidney transplantation: a systematic review and meta-analysis of randomized controlled trials. International Urology and Nephrology, 2023, 55(10): 2545-2556.
219 Zhuang K, Yang H, Long Y, et al. Dexmedetomidine and acute kidney injury after non-cardiac surgery: a meta-analysis with trial sequential analysis. Anaesthesia Critical Care & Pain Medicine, 2024: 101359.
220 Guo S, Jia D, Liu X, et al. The positive efficacy of dexmedetomidine on the clinical outcomes of patients undergoing renal transplantation: evidence from meta-analysis. Aging (Albany NY), 2023, 15(23): 14192.
221 Ng K T, Lim W E, Teoh W Y, et al. Effects of perioperative dexmedetomidine on delayed graft function following renal transplant: a systematic review and meta-analysis. Brazilian Journal of Anesthesiology (English Edition), 2024, 74(6): 844534.
222 Tong T, Mei G, Zhang Y. Reno-protective effects of perioperative dexmedetomidine in kidney transplantation: a systematic review and meta-analysis of randomized controlled trials. Int Urol Nephrol. 2023;55(10):2545-2556
223 Peng C, Li D, Guo T, et al. The Efficacy of Different Exercises on Mild to Moderate Adolescent Idiopathic Scoliosis: A Systematic Review and Meta-analysis. American Journal of Physical Medicine & Rehabilitation, 2023: 10.1097.
224 Sun P, Liu D, Cheng R, et al. Short-Term Benefit from Core Stabilization Exercises in Adolescent Idiopathic Scoliosis: A Meta-Analysis of Randomized Controlled Trials. Health & Social Care in the Community, 2023, 2023(1): 5014254.
225 Hsu W T, Hsu C M, Hung S C, et al. Acupuncture improves sleep disorders and depression among patients with Parkinson’s disease: a meta-analysis. Healthcare. MDPI, 2023, 11(14): 2042.
226 Sun Y, Sheng J, Liu T, et al. Combination treatment of acupoint therapy and conventional medication for motor function of Parkinson's disease: A systematic review and meta-analysis. Complementary Therapies in Clinical Practice, 2023, 50: 101677.
227 Yan F, Chen C, Feng Q, et al. Acupuncture and sleep disorders in Parkinson’s disease: A systematic evaluation with meta-analysis. Medicine, 2024, 103(1): e36286.
228 Saboori S, Rad E Y, Mardani M, et al. Effect of Q10 supplementation on body weight and body mass index: A systematic review and meta-analysis of randomized controlled clinical trials. Diabetes & Metabolic Syndrome: Clinical Research & Reviews, 2019, 13(2): 1179-1185.
229 Ghavami A, Mohammadi H, Hadi A, et al. Effects of coenzyme Q10 supplementation on anthropometric indices in adults: a systematic review and meta-analysis of randomized controlled trials. International Journal of Preventive Medicine, 2020, 11(1): 181.
230 Jagan S, Mohd Daud T I, Chia L C, et al. Evidence for the effectiveness of psychological interventions for internalized stigma among adults with schizophrenia spectrum disorders: A systematic review and meta-analyses. International Journal of Environmental Research and Public Health, 2023, 20(8): 5570.
231 Lamarca M, Espinosa V, Acuña V, et al. Reducing self-stigma in psychosis: A systematic review and meta-analysis of psychological interventions. Psychiatry Research, 2024, 342: 116262.
232 Barile L, Fominskiy E, Di Tomasso N, et al. Acute normovolemic hemodilution reduces allogeneic red blood cell transfusion in cardiac surgery: a systematic review and meta-analysis of randomized trials. Anesthesia & Analgesia, 2017, 124(3): 743-752.

233 Carless P, Moxey A, O'Connell D, et al. Autologous transfusion techniques: a systematic review of their efficacy. Transfusion Medicine, 2004, 14(2): 123-144.
234 Li S, Liu Y, Zhu Y. Effect of acute normovolemic hemodilution on coronary artery bypass grafting: a systematic review and meta-analysis of 22 randomized trials. International Journal of Surgery, 2020, 83: 131-139.
235 Abedian S, Abedi P, Jahanfar S, et al. The effect of Lavender on pain and healing of episiotomy: A systematic review. Complement Ther Med. 2020;53:102510.
236 Abner EL, Schmitt FA, Mendiondo MS, et al. Vitamin E and all-cause mortality: a meta-analysis. Curr Aging Sci. 2011;4(2):158-170.
237 Abowali HA, Paganini M, Enten Get al. Critical Review and Meta-Analysis of Postoperative Sedation after Adult Cardiac Surgery: Dexmedetomidine Versus Propofol. J Cardiothorac Vasc Anesth. 2021;35(4):1134-1142.
238 Abu Hashim H, Foda O, Ghayaty E. Combined metformin-clomiphene in clomiphene-resistant polycystic ovary syndrome: a systematic review and meta-analysis of randomized controlled trials. Acta Obstet Gynecol Scand. 2015;94(9):921-930.
239 Adil MT, Perera M, Whitelaw D, et al. Systematic Review and Meta-analysis of the Effects of Laparoscopic Roux-en-Y Gastric Bypass and Laparoscopic Sleeve Gastrectomy on Dyslipidemia. Obes Surg. 2024;34(3):967-975.
240 Afshin A, Micha R, Khatibzadeh S,et al. Consumption of nuts and legumes and risk of incident ischemic heart disease, stroke, and diabetes: a systematic review and meta-analysis. Am J Clin Nutr. 2014;100(1):278-288.
241 Agarwal A, Basmaji J, Fernando SM, et al. Parenteral Vitamin C in Patients with Severe Infection: A Systematic Review. NEJM Evid. 2022;1(9):EVIDoa2200105.
242 Ahn KH, Bae NY, Hong SC, et al. The safety of progestogen in the prevention of preterm birth: meta-analysis of neonatal mortality. J Perinat Med. 2017;45(1):11-20.
243 Akbari A, Razmi M, Sedaghat A, et al. Comparative effectiveness of pharmacological interventions on mortality and the average length of hospital stay of patients with COVID-19: a systematic review and meta-analysis of randomized controlled trials. Expert Rev Anti Infect Ther. 2022;20(4):585-609.
244 Akbari M, Moosazaheh M, Lankarani KB, et al. The Effects of Vitamin D Supplementation on Glucose Metabolism and Lipid Profiles in Patients with Gestational Diabetes: A Systematic Review and Meta-Analysis of Randomized Controlled Trials [published correction appears in Horm Metab Res. 2017 Sep;49(9):e3.
245 Akbari M, Ostadmohammadi V, Lankarani KB, et al. The Effects of Vitamin D Supplementation on Biomarkers of Inflammation and Oxidative Stress Among Women with Polycystic Ovary Syndrome: A Systematic Review and Meta-Analysis of Randomized Controlled Trials. Horm Metab Res. 2018;50(4):271-279.
246 Akinoso-Imran A Q, Adetunji H. Systematic review and meta-analysis of letrozole and clomiphene citrate in polycystic ovary syndrome. Middle East Fertility Society Journal, 2018, 23(3): 163-170.
247 Al Khalaf MM, Thalib L, Doi SA. Cardiovascular outcomes in high-risk patients without heart failure treated with ARBs: a systematic review and meta-analysis. Am J Cardiovasc Drugs. 2009;9(1):29-43.

248 Albert SG, Reddy S. CLINICAL EVALUATION OF COST EFFICACY OF DRUGS FOR TREATMENT OF OSTEOPOROSIS: A META-ANALYSIS. Endocr Pract. 2017 Jul;23(7):841-856.
249 Albrecht E, Vorobeichik L, Jacot-Guillarmod A,et al. Dexamethasone Is Superior to Dexmedetomidine as a Perineural Adjunct for Supraclavicular Brachial Plexus Block: Systematic Review and Indirect Meta-analysis. Anesth Analg. 2019;128(3):543-554.
250 Alghamdi S, Mirghani H, Alhazmi K, et al. Roux-en-Y gastric bypass and laparoscopic sleeve gastrectomy effects on obesity comorbidities: A systematic review and meta-analysis. Front Surg. 2022;9:953804.
251 Ali M, Wang Y, Ji J, et al. One Anastomosis Gastric Bypass Versus Sleeve Gastrectomy for Obesity: a Systemic Review and Meta-analysis. J Gastrointest Surg. 2023;27(10):2226-2244.
252 Almeida RAC, Lemos CAA, de Moraes SLD, Pellizzer EP, Vasconcelos BC. Efficacy of corticosteroids versus placebo in impacted third molar surgery: systematic review and meta-analysis of randomized controlled trials. Int J Oral Maxillofac Surg. 2019;48(1):118-131.
253 Alozkan-Sever C, Uppendahl JR, Cuijpers P, et al. Research Review: Psychological and psychosocial interventions for children and adolescents with depression, anxiety, and post-traumatic stress disorder in low- and middle-income countries - a systematic review and meta-analysis. J Child Psychol Psychiatry. 2023;64(12):1776-1788.
254 Alsanosi, S. M. M. Blood pressure-lowering agents response-a systematic review and genome wide study.University of Glasgow, 2017.
255 Amani B, Khanijahani A, Amani B. Hydroxychloroquine plus standard of care compared with standard of care alone in COVID-19: a meta-analysis of randomized controlled trials. Sci Rep. 2021;11(1):11974.
256 Aminian A. Bariatric procedure selection in patients with type 2 diabetes: choice between Roux-en-Y gastric bypass or sleeve gastrectomy. Surg Obes Relat Dis. 2020;16(2):332-339.
257 Andersen JH, Karlsen A, Geisler A, et al. Alpha2 -receptor agonists as adjuvants for brachial plexus nerve blocks-A systematic review with meta-analyses. Acta Anaesthesiol Scand. 2022;66(2):186-206.
258 Annweiler C, Llewellyn DJ, Beauchet O. Low serum vitamin D concentrations in Alzheimer's disease: a systematic review and meta-analysis. J Alzheimers Dis. 2013;33(3):659-674.
259 Anzai A, Utino A, Tosello G, et al. Sugammadex in awakening from general anesthesia: systematic review and meta-analysis. Rev Assoc Med Bras (1992). 2022;68(9):1130-1153.
260 Aoki Y, Aoshima Y, Atsumi K, et al. Perioperative Amino Acid Infusion for Preventing Hypothermia and Improving Clinical Outcomes During Surgery Under General Anesthesia: A Systematic Review and Meta-analysis. Anesth Analg. 2017;125(3):793-802.
261 Atteya A A, El-Semary M, Elbaz S K A. Role of physical therapy interventions in orofacial dysfunction after stroke: systematic review. Egyptian Journal of Applied Science, 2020, 34(1): 23-33.
262 Avenell A, Gillespie WJ, Gillespie LD, et al. Vitamin D and vitamin D analogues for preventing fractures associated with involutional and post-menopausal osteoporosis. Cochrane Database Syst Rev. 2009;(2):CD000227.
263 Awad K, Zaki MM, Mohammed M, et al. Effect of the Renin-Angiotensin System Inhibitors on Inflammatory Markers: A Systematic Review and Meta-analysis of Randomized Controlled Trials. Mayo Clin Proc. 2022;97(10):1808-1823.

264 Axfors C, Schmitt AM, Janiaud P, et al. Mortality outcomes with hydroxychloroquine and chloroquine in COVID-19 from an international collaborative meta-analysis of randomized trials . Nat Commun. 2021;12(1):2349.
265 Badely M, Sepandi M, Samadi M,et al. The effect of whey protein on the components of metabolic syndrome in overweight and obese individuals; a systematic review and meta-analysis. Diabetes Metab Syndr. 2019;13(6):3121-3131.
266 Baghdadi LR, Abu Hashim H, Amer SA, et al. Impact of obesity on reproductive outcomes after ovarian ablative therapy in PCOS: a collaborative meta-analysis. Reprod Biomed Online. 2012;25(3):227-241.
267 Bahrami LS, Ranjbar G, Norouzy A, et al. Vitamin D supplementation effects on the clinical outcomes of patients with coronary artery disease: a systematic review and meta-analysis. Sci Rep. 2020;10(1):12923.
268 Baird E, Williams ACC, Hearn L, et al. Interventions for treating persistent pain in survivors of torture. Cochrane Database Syst Rev. 2017;8(8):CD012051.
269 Bala MM, Paszek E, Lesniak W, et al. Antiplatelet and anticoagulant agents for primary prevention of thrombosis in individuals with antiphospholipid antibodies. Cochrane Database Syst Rev. 2018;7(7):CD012534.
270 Bangalore S, Fakheri R, Toklu B, et al. Diabetes mellitus as a compelling indication for use of renin angiotensin system blockers: systematic review and meta-analysis of randomized trials. BMJ. 2016,14;352:i1525.
271 Bangalore S, Kumar S, Kjeldsen SE, et al. Antihypertensive drugs and risk of cancer: network meta-analyses and trial sequential analyses of 324,168 participants from randomised trials. Lancet Oncol. 2011;12(1):65-82.
272 Bangalore S, Kumar S, Wetterslev J,et al. Angiotensin receptor blockers and risk of myocardial infarction: meta-analyses and trial sequential analyses of 147 020 patients from randomised trials. BMJ. 2011;342:d2234.
273 Bangalore S, Singh A, Toklu B, et al. Efficacy of cilostazol on platelet reactivity and cardiovascular outcomes in patients undergoing percutaneous coronary intervention: insights from a meta-analysis of randomised trials. Open Heart. 2014;1(1):e000068.
274 Barrons RW, Woods JA. The Roles of ACE Inhibitors in Lower Extremity Peripheral Artery Disease. Am J Ther. 2016;23(1):e7-e15.
275 Beaudart C, Buckinx F, Rabenda V, et al. The effects of vitamin D on skeletal muscle strength, muscle mass, and muscle power: a systematic review and meta-analysis of randomized controlled trials. J Clin Endocrinol Metab. 2014;99(11):4336-4345.
276 Bekiari E, Rizava C, Athanasiadou E, et al. Systematic review and meta-analysis of vildagliptin for treatment of type 2 diabetes. Endocrine. 2016;52(3):458-480.
277 Beran A, Mhanna M, Srour O, et al. Clinical significance of micronutrient supplements in patients with coronavirus disease 2019: A comprehensive systematic review and meta-analysis. Clin Nutr ESPEN. 2022;48:167-177.
278 Berger MD, Trelle S, Büchi AE, et al. Impact on survival through consolidation radiotherapy for diffuse large B-cell lymphoma: a comprehensive meta-analysis. Haematologica. 2021;106(7):1923-1931.
279 Bernardo WM, Aires FT. Efficacy of dexamethasone in the prophylaxis of nausea and vomiting during the postoperative period of laparoscopic cholecystectomy. Rev Assoc Med Bras (1992). 2013;59(4):387-391.

280 Beveridge LA, Struthers AD, Khan F, et al. Effect of Vitamin D Supplementation on Blood Pressure: A Systematic Review and Meta-analysis Incorporating Individual Patient Data. JAMA Intern Med. 2015;175(5):745-754.
281 Bi X, Wei J, Zhang X. Effects of dexmedetomidine on neurocognitive disturbance after elective non-cardiac surgery in senile patients: a systematic review and meta-analysis. J Int Med Res. 2021;49(5):3000605211014294.
282 Bignardi PR, Vengrus CS, Aquino BM,et al. Use of hydroxychloroquine and chloroquine in patients with COVID-19: a meta-analysis of randomized clinical trials. Pathog Glob Health. 2021;115(3):139-150.
283 Bjelakovic G, Gluud LL, Nikolova D, et al. Vitamin D supplementation for prevention of mortality in adults. Cochrane Database Syst Rev. 2014;(1):CD007470.
284 Bjelakovic G, Nikolova D, Gluud LL, et al. Antioxidant supplements for prevention of mortality in healthy participants and patients with various diseases. Cochrane Database Syst Rev. 2008;(2):CD007176.
285 Bjelakovic G, Nikolova D, Gluud LL, et al. Mortality in randomized trials of antioxidant supplements for primary and secondary prevention: systematic review and meta-analysis . JAMA. 2007;297(8):842-857.
286 Blanco Mejia S, Kendall CW, Viguiliouk E, et al. Effect of tree nuts on metabolic syndrome criteria: a systematic review and meta-analysis of randomised controlled trials. BMJ Open. 2014;4(7):e004660.
287 Bordewijk EM, Ng KYB, Rakic L, et al. Laparoscopic ovarian drilling for ovulation induction in women with anovulatory polycystic ovary syndrome. Cochrane Database Syst Rev. 2020;2(2):CD001122.
288 Borgeraas H, Hofsø D, Hertel JK,et al. Comparison of the effect of Roux-en-Y gastric bypass and sleeve gastrectomy on remission of type 2 diabetes: A systematic review and meta-analysis of randomized controlled trials [published correction appears in Obes Rev. 2022;23(4):e13432.
289 Brazzelli M, Javanbakht M, Imamura M, et al. Surgical treatments for women with stress urinary incontinence: the ESTER systematic review and economic evaluation. Health Technol Assess. 2019;23(14):1-306.
290 Brown J, Farquhar C. Clomiphene and other antioestrogens for ovulation induction in polycystic ovarian syndrome. Cochrane Database Syst Rev. 2016;12(12):CD002249.
291 Bryant A, Lawrie TA, Dowswell T, et al. Ivermectin for Prevention and Treatment of COVID-19 Infection: A Systematic Review, Meta-analysis, and Trial Sequential Analysis to Inform Clinical Guidelines. Am J Ther. 2021;28(4):e434-e460.
292 Bunn F, Trivedi D. Colloid solutions for fluid resuscitation. Cochrane Database Syst Rev. 2012;2012(7):CD001319.
293 Cai X, Han X, Luo Y,et al. Comparisons of the efficacy of alpha glucosidase inhibitors on type 2 diabetes patients between Asian and Caucasian. PLoS One. 2013;8(11):e79421.
294 Cao F, Li J, Li F. Mechanical bowel preparation for elective colorectal surgery: updated systematic review and meta-analysis. Int J Colorectal Dis. 2012;27(6):803-810.
295 CaoY R, Shi H, Zhai J. Effects of endometrial stimulation timings and techniques on pregnancy outcomes in patients without prior embryo transfer: a systematic review and meta-analysis. Reproductive and Developmental Medicine, 4(03), 169-176.

296 Carless PA, Rubens FD, Anthony DM, et al. Platelet-rich-plasmapheresis for minimising peri-operative allogeneic blood transfusion. Cochrane Database Syst Rev. 2011;(3):CD004172.
297 Carlisle JB, Stevenson CA. Drugs for preventing postoperative nausea and vomiting. Cochrane Database Syst Rev. 2006;2006(3):CD004125.
298 Carp H. A systematic review of dydrogesterone for the treatment of recurrent miscarriage. Gynecol Endocrinol. 2015;31(6):422-430.
299 Carp HJ. Progestogens in the prevention of miscarriage. Horm Mol Biol Clin Investig. 2016;27(2):55-62.
300 Casey MB, Smart KM, Segurado R, et al. Multidisciplinary-based Rehabilitation (MBR) Compared With Active Physical Interventions for Pain and Disability in Adults With Chronic Pain: A Systematic Review and Meta-analysis. Clin J Pain. 2020;36(11):874-886.
301 Castellana M, Procino F, Biacchi E, et al. Roux-en-Y Gastric Bypass vs Sleeve Gastrectomy for Remission of Type 2 Diabetes. J Clin Endocrinol Metab. 2021;106(3):922-933.
302 Chang WD, Chen S, Lee CL, et al. The Effects of Tai Chi Chuan on Improving Mind-Body Health for Knee Osteoarthritis Patients: A Systematic Review and Meta-Analysis. Evid Based Complement Alternat Med. 2016;2016:1813979.
303 Chappell D, van der Linden P, Ripollés-Melchor J, et al. Safety and efficacy of tetrastarches in surgery and trauma: a systematic review and meta-analysis of randomised controlled trials. Br J Anaesth. 2021;127(4):556-568.
304 Chaugai S, Sherpa LY, Sepehry AA, et al. Effects of Long- and Intermediate-Acting Dihydropyridine Calcium Channel Blockers in Hypertension: A Systematic Review and Meta-Analysis of 18 Prospective, Randomized, Actively Controlled Trials. J Cardiovasc Pharmacol Ther. 2018;23(5):433-445.
305 Chen C, Fang J, Chen S, et al. The efficacy and safety of remdesivir alone and in combination with other drugs for the treatment of COVID-19: a systematic review and meta-analysis. BMC Infect Dis. 2023;23(1):672.
306 Chen CK, Weng TS, Chen YH,et al. Clinical efficacy of sofosbuvir/daclatasvir in patients with COVID-19: a systematic review and meta-analysis of randomized trials. Expert Rev Clin Pharmacol. 2022 Aug;15(8):997-1002.
307 Chen CT, Tung HH, Fang CJ, et al. Effect of music therapy on improving sleep quality in older adults: A systematic review and meta-analysis. J Am Geriatr Soc. 2021;69(7):1925-1932.
308 Chen CY, Chiu CT, Lee HS, et al. The impact of vitamin C-containing treatment on the mortality of patients with sepsis: A systematic review and meta-analysis of randomized controlled trials. J Infect Public Health. 2022;15(12):1514-1520.
309 Chen HL, Woo XB, Cui J, et al. Ligasure versus stapled hemorrhoidectomy in the treatment of hemorrhoids: a meta-analysis of randomized control trials. Surg Laparosc Endosc Percutan Tech. 2014;24(4):285-289.
310 Chen J, Gan L, Wu G. The Meta analysis of postoperative nausea and vomiting after taking granisetron with hexadecadrol for laparoscopic cholecystemy. Life Science Journal, 2013, 10(4).
311 Chen L, Wang G, Zheng F, et al. Efficacy of bisphosphonates against osteoporosis in adult men: a meta-analysis of randomized controlled trials. Osteoporos Int. 2015;26(9):2355-2363.
312 Chen N, Wan Z, Han SF, et al. Effect of vitamin D supplementation on the level of circulating high-sensitivity C-reactive protein: a meta-analysis of randomized controlled trials. Nutrients. 2014;6(6):2206-2216.

313 Chen P, Li X, Sang L, et al. Perioperative intravenous glucocorticoids can decrease postoperative nausea and vomiting and pain in total joint arthroplasty: A meta-analysis and trial sequence analysis. Medicine (Baltimore). 2017;96(13):e6382.
314 Chen YH, Fogel L, Sun AY, et al. The Efficacy and Safety of Tandem Transplant Versus Single Stem Cell Transplant for Multiple Myeloma Patients: A Systematic Review and Meta-Analysis. Diagnostics (Basel). 2024;14(10):1030.
315 Chen, H. B., Chen, P., Li, K., Shao, J. Application of Intravenous Vitamin C in Adult Patients with Sepsis: A Meta-Analysis of Randomized Controlled Trials.
316 Cheng JOS, Cheng ST. Effectiveness of physical and cognitive-behavioural intervention programmes for chronic musculoskeletal pain in adults: A systematic review and meta-analysis of randomised controlled trials. PLoS One. 2019;14(10):e0223367.
317 Cheng YC, Huang YC, Huang WL. The effect of vitamin D supplement on negative emotions: A systematic review and meta-analysis. Depress Anxiety. 2020;37(6):549-564.
318 Cheuk DK, Yeung WF, Chung KF, Wong V. Acupuncture for insomnia. Cochrane Database Syst Rev. 2007;(3):CD005472.
319 Chiang MH, Wu SC, Hsu SW,et al. Bispectral Index and non-Bispectral Index anesthetic protocols on postoperative recovery outcomes. Minerva Anestesiol. 2018;84(2):216-228.
320 Chooi C, Cox JJ, Lumb RS, et al. Techniques for preventing hypotension during spinal anaesthesia for caesarean section. Cochrane Database Syst Rev. 2017;8(8):CD002251.
321 Chua ME, Gnech M, Ming JM, et al. Preoperative hormonal stimulation effect on hypospadias repair complications: Meta-analysis of observational versus randomized controlled studies. J Pediatr Urol. 2017;13(5):470-480.
322 Climent E, Goday A, Pedro-Botet J, et al. Laparoscopic Roux-en-Y gastric bypass versus laparoscopic sleeve gastrectomy for 5-year hypertension remission in obese patients: a systematic review and meta-analysis. J Hypertens. 2020;38(2):185-195.
323 Cockayne S, Adamson J, Lanham-New S, et al. Vitamin K and the prevention of fractures: systematic review and meta-analysis of randomized controlled trials. JAMA Intern Med. 2018,178(6):875-876.
324 Cohen B, Schacham YN, Ruetzler K, et al. Effect of intraoperative hyperoxia on the incidence of surgical site infections: a meta-analysis. Br J Anaesth. 2018;120(6):1176-1186.
325 Coll JA, Seale NS, Vargas K, et al. Primary Tooth Vital Pulp Therapy: A Systematic Review and Meta-analysis. Pediatr Dent. 2017;39(1):16-123.
326 Cooper K, Martyn-St James M, Kaltenthaler E, et al. Interventions to treat premature ejaculation: a systematic review short report. Health Technol Assess. 2015;19(21):1-vi.
327 Crichton ML, Goeminne PC, Tuand K, et al. The impact of therapeutics on mortality in hospitalised patients with COVID-19: systematic review and meta-analyses informing the European Respiratory Society living guideline. Eur Respir Rev. 2021;30(162):210171.
328 Cruciani M, Pati I, Masiello F,et al. Ivermectin for Prophylaxis and Treatment of COVID-19: A Systematic Review and Meta-Analysis [published correction appears in Diagnostics (Basel). 2021;11(12):2359.
329 Cruz-Lemini M, Vázquez JC, Ullmo J, et al. Low-molecular-weight heparin for prevention of preeclampsia and other placenta-mediated complications: a systematic review and meta-analysis. Am J Obstet Gynecol. 2022;226(2S):S1126-S1144.e17.

330 Csiki E, Szabó H, Hanák L, et al. Oral Proton Pump Inhibitors May Be as Effective as Intravenous in Peptic Ulcer Bleeding: A Systematic Review and Meta-analysis. Clin Transl Gastroenterol. 2021;12(4):e00341.
331 Dai W, Tang M, He K. The effect and safety of dexmedetomidine added to ropivacaine in brachial plexus block: A meta-analysis of randomized controlled trials. Medicine (Baltimore). 2018;97(41):e12573.
332 Daien V, Duny Y, Ribstein J, et al. Treatment of hypertension with renin-angiotensin system inhibitors and renal dysfunction: a systematic review and meta-analysis. Am J Hypertens. 2012;25(1):126-132.
333 Dale O, Somogyi AA, Li Y, Sullivan T, Shavit Y. Does intraoperative ketamine attenuate inflammatory reactivity following surgery? A systematic review and meta-analysis. Anesth Analg. 2012;115(4):934-943.
334 Danbala I A, Sheng W, Tang H, et al. Systematic review and meta-analysis on safety and efficacy of immune checkpoint inhibitors and radiotherapy for advanced pancreatic cancer. World Journal of Advanced Research and Reviews, 2023, 20(3): 638-648.
335 Dashti F, Mousavi SM, Larijani B, et al. The effects of vitamin D supplementation on inflammatory biomarkers in patients with abnormal glucose homeostasis: A systematic review and meta-analysis of randomized controlled trials. Pharmacol Res. 2021;170:105727.
336 Dathini H, Sharoni SKA, Robert KT. Parental Reminder Strategies and the Cost Implication for Improved Immunisation Outcomes: A Systematic Review and Meta-Analysis. Healthcare (Basel). 2022;10(10):1996.
337 Datzmann T, Fuchs S, Andree D, et al. Systematic review and meta-analysis of randomised controlled clinical trial evidence refutes relationship between pharmacotherapy with angiotensin-receptor blockers and an increased risk of cancer. Eur J Intern Med. 2019;64:1-9.
338 De Carvalho MR, Peixoto BU, Silveira IA, Oliveria BGRB. A Meta-analysis to Compare Four-layer to Short-stretch Compression Bandaging for Venous Leg Ulcer Healing. Ostomy Wound Manage. 2018;64(5):30-37.
339 de Jong PG, Kaandorp S, Di Nisio M, et al. Aspirin and/or heparin for women with unexplained recurrent miscarriage with or without inherited thrombophilia. Cochrane Database Syst Rev. 2014;2014(7):CD004734.
340 de Liyis BG, Sutedja JC, Tjandra DC, et al. Serotonin norepinephrine reuptake inhibitors in managing neuropathic pain following spinal and non-spinal surgery: A systematic review and meta-analysis of randomized controlled trials. Clin Neurol Neurosurg. 2024;239:108223.
341 de Melo PS, Gianlorenco AC, Marduy A, et al. A Mechanistic Analysis of the Neural Modulation of the Inflammatory System Through Vagus Nerve Stimulation: A Systematic Review and Meta-analysis. Neuromodulation.2025;28(1):43-53.
342 De Oliveira GS Jr, Castro-Alves LJ, Ahmad S, Kendall MC, McCarthy RJ. Dexamethasone to prevent postoperative nausea and vomiting: an updated meta-analysis of randomized controlled trials. Anesth Analg. 2013;116(1):58-74.
343 de Vaan MD, Ten Eikelder ML, Jozwiak M, et al. Mechanical methods for induction of labour. Cochrane Database Syst Rev. 2019;10(10):CD001233
344 Deane KHO, Jimoh OF, Biswas P, et al. Omega-3 and polyunsaturated fat for prevention of depression and anxiety symptoms: systematic review and meta-analysis of randomised trials. Br J Psychiatry. 2021;218(3):135-142.
345 Delaney AP, Dan A, McCaffrey J, et al. The role of albumin as a resuscitation fluid for patients with sepsis: a systematic review and meta-analysis. Crit Care Med. 2011;39(2):386-391.

346 Deng J, Zhou F, Ali S, et al. Efficacy and safety of ivermectin for the treatment of COVID-19: a systematic review and meta-analysis [published correction appears in QJM. 2022;115(10):706.
347 Deng J, Zhou F, Heybati K, et al. Efficacy of chloroquine and hydroxychloroquine for the treatment of hospitalized COVID-19 patients: a meta-analysis. Future Virol. 2021;10.2217/fvl-2021-0119.
348 Deng W, Yang C, Yang S, et al. Evaluation of favipiravir in the treatment of COVID-19 based on the real-world. Expert Rev Anti Infect Ther. 2022;20(4):555-565.
349 Di Castelnuovo A, Costanzo S, Cassone A, et al. Hydroxychloroquine and mortality in COVID-19 patients: a systematic review and a meta-analysis of observational studies and randomized controlled trials. Pathog Glob Health. 2021;115(7-8):456-466.
350 Dias ATB, Modesto TB, Oliveira SA. Effectiveness of the use of Low Molecular Heparin in patients with repetition abortion history: Systematic review and meta-analysis. JBRA Assist Reprod. 2021;25(1):10-27.
351 Dibaba DT. Effect of vitamin D supplementation on serum lipid profiles: a systematic review and meta-analysis. Nutr Rev. 2019;77(12):890-902.
352 Dicembrini I, Mannucci E, Monami M, et al. Impact of technology on glycaemic control in type 2 diabetes: A meta-analysis of randomized trials on continuous glucose monitoring and continuous subcutaneous insulin infusion. Diabetes Obes Metab. 2019;21(12):2619-2625.
353 Dieng M, Watts CG, Kasparian NA, et al. Improving subjective perception of personal cancer risk: systematic review and meta-analysis of educational interventions for people with cancer or at high risk of cancer. Psychooncology. 2014;23(6):613-625.
354 Do MT, Kim L, Im YJ, et al. Effect of Preoperative Androgen Stimulation on Penile Size and Postoperative Complication Rate in Patients with Hypospadias: A Systematic Review and Meta-Analysis. World J Mens Health. 2023;41(3):558-574.
355 Dodd JM, Grivell RM, OBrien CM, et al. Prenatal administration of progestogens for preventing spontaneous preterm birth in women with a multiple pregnancy. Cochrane Database Syst Rev. 2019;2019(11):CD012024.
356 Dong W, Zhang T, Wang ZG,et al. Clinical outcome of small hepatocellular carcinoma after different treatments: a meta-analysis. World J Gastroenterol. 2014;20(29):10174-82.
357 Dong Y, Wang W, Zheng J, et al. Whole Body Vibration Exercise for Chronic Musculoskeletal Pain: A Systematic Review and Meta-analysis of Randomized Controlled Trials. Arch Phys Med Rehabil. 2019;100(11):2167-2178.
358 Doulton TW, He FJ, MacGregor GA. Systematic review of combined angiotensin-converting enzyme inhibition and angiotensin receptor blockade in hypertension. Hypertension. 2005;45(5):880-886.
359 Dower A, Mulcahy M, Maharaj M, et al. Surgical decompression for malignant cerebral oedema after ischaemic stroke. Cochrane Database Syst Rev. 2022;11(11):CD014989.
360 Duran-Retamal M, Morris G, Achilli C, et al. Live birth and miscarriage rate following intracytoplasmic morphologically selected sperm injection vs intracytoplasmic sperm injection: An updated systematic review and meta-analysis. Acta Obstet Gynecol Scand. 2020;99(1):24-33.
361 El-Kadiki A, Sutton AJ. Role of multivitamins and mineral supplements in preventing infections in elderly people: systematic review and meta-analysis of randomised controlled trials. BMJ. 2005;330(7496):871.

362 Ellahi A, Stewart F, Kidd EA, et al. Strategies for the removal of short-term indwelling urethral catheters in adults. Cochrane Database Syst Rev. 2021;6(6):CD004011.
363 EM Ramos, MD de Lima, JEC Gonzalez, et al. Vitamin D3 Supplementation: An Option Associated with The Treatment of Multiple Sclerosis: A Systematic Review and Meta-Analysis. International Journal for Innovation Education and Research. 2020;8(5):464-478.
364 Escalante Y, García-Hermoso A, Saavedra JM. Effects of exercise on functional aerobic capacity in lower limb osteoarthritis: a systematic review. J Sci Med Sport. 2011;14(3):190-198.
365 Eze P, Mezue KN, Nduka CU, et al. Efficacy and safety of chloroquine and hydroxychloroquine for treatment of COVID-19 patients-a systematic review and meta-analysis of randomized controlled trials. Am J Cardiovasc Dis. 2021;11(1):93-107.
366 Fan Y, Huang Z, Yu D. Incontinence-specific quality of life measures used in trials of sling procedures for female stress urinary incontinence: a meta-analysis. Int Urol Nephrol. 2015;47(8):1277-1295.
367 Fan Z, Ma J, Kuang M, et al. The efficacy of dexamethasone reducing postoperative pain and emesis after total knee arthroplasty: A systematic review and meta-analysis. Int J Surg. 2018;52:149-155.
368 Farahmand MA, Daneshzad E, Fung TT, et al. What is the impact of vitamin D supplementation on glycemic control in people with type-2 diabetes: a systematic review and meta-analysis of randomized controlled trails. BMC Endocr Disord. 2023;23(1):15.
369 Farmer AJ, Stevens R, Hirst J, et al. Optimal strategies for identifying kidney disease in diabetes: properties of screening tests, progression of renal dysfunction and impact of treatment - systematic review and modelling of progression and cost-effectiveness. Health Technol Assess. 2014;18(14):1-128.
370 Farquhar C, Brown J, Marjoribanks J. Laparoscopic drilling by diathermy or laser for ovulation induction in anovulatory polycystic ovary syndrome. Cochrane Database Syst Rev. 2012;(6):CD001122.
371 Feng H, Feng ML, Cheng JB, et al. Meta-analysis of factors influencing anterior knee pain after total knee arthroplasty. World J Orthop. 2024;15(2):180-191.
372 Feng LS, Hong G, Yan Z, et al. Intrathecal Sufentanil Does Not Reduce Shivering During Neuraxial Anesthesia: A Meta-Analysis. Med Sci Monit. 2016;22:258-266.
373 Feng W, Hu Y, Zhang C, et al. Efficacy and safety of mycophenolate mofetil in the treatment of moderate to severe Graves' orbitopathy: a meta-analysis. Bioengineered. 2022;13(6):14719-14729.
374 Fernandes N, Bryant D, Griffith L, et al. Outcomes for patients with the same disease treated inside and outside of randomized trials: a systematic review and meta-analysis. CMAJ. 2014;186(16):E596-E609.
375 Fernández-Pérez P, Leirós-Rodríguez R, Marqués-Sánchez MP, et al. Effectiveness of physical therapy interventions in women with dyspareunia: a systematic review and meta-analysis. BMC Womens Health. 2023;23(1):387.
376 Fernández-Rodríguez R, Martínez-Vizcaíno V, Garrido-Miguel M, et al. Nut consumption, body weight, and adiposity in patients with type 2 diabetes: a systematic review and meta-analysis of randomized controlled trials. Nutr Rev. 2022;80(4):645-655.
377 Ferrari R, Boersma E. The impact of ACE inhibition on all-cause and cardiovascular mortality in contemporary hypertension trials: a review. Expert Rev Cardiovasc Ther. 2013;11(6):705-717.

378 Ferrari R. RAAS inhibition and mortality in hypertension. Glob Cardiol Sci Pract. 2013;2013(3):269-278.
379 Ferreira RDDS, Negrini R, Bernardo WM, et al. The effects of sildenafil in maternal and fetal outcomes in pregnancy: A systematic review and meta-analysis. PLoS One. 2019;14(7):e0219732.
380 Fiki Cahya Ningrum, Bhisma Murti, Vitri Widyaningsih.comparative efficacy between intravenous iron and oral iron on enhancing hemoglobin level among pregnant women with iron deficiency anemia in low and middle income countries a meta-analysis.Indonesian Journal of Medicine. 2019,4(2): 135-144.
381 Fisher SA, Doree C, Mathur A, et al. Meta-analysis of cell therapy trials for patients with heart failure. Circ Res. 2015;116(8):1361-1377.
382 Fogacci S, Fogacci F, Banach M, et al. Vitamin D supplementation and incident preeclampsia: A systematic review and meta-analysis of randomized clinical trials. Clin Nutr. 2020;39(6):1742-1752.
383 Ford AA, Rogerson L, Cody JD, et al. Mid-urethral sling operations for stress urinary incontinence in women. Cochrane Database Syst Rev. 2015;(7):CD006375.
384 Franco AS, Freitas TQ, Bernardo WM, et al. Vitamin D supplementation and disease activity in patients with immune-mediated rheumatic diseases: A systematic review and meta-analysis. Medicine (Baltimore). 2017;96(23):e7024.
385 Franik S, Eltrop SM, Kremer JA, et al. Aromatase inhibitors (letrozole) for subfertile women with polycystic ovary syndrome. Cochrane Database Syst Rev. 2018;5(5):CD010287.
386 Friedrich JO, Adhikari N, Herridge MS, et al. Meta-analysis: low-dose dopamine increases urine output but does not prevent renal dysfunction or death. Ann Intern Med. 2005;142(7):510-524.
387 Gadalla MA, Huang S, Wang R, et al. Effect of clomiphene citrate on endometrial thickness, ovulation, pregnancy and live birth in anovulatory women: systematic review and meta-analysis. Ultrasound Obstet Gynecol. 2018;51(1):64-76.
388 Gallo S, McDermid JM, Al-Nimr RI, et al. Vitamin D Supplementation during Pregnancy: An Evidence Analysis Center Systematic Review and Meta-Analysis. J Acad Nutr Diet. 2020;120(5):898-924.e4.
389 Gandhi GY, Murad MH, Flynn DN, et al. Effect of perioperative insulin infusion on surgical morbidity and mortality: systematic review and meta-analysis of randomized trials.7. Mayo Clin Proc. 2008;83(4):418-430.
390 Gao L, Yang L, Lv X, et al. A systematic review and meta-analysis of comparative studies on the efficacy of extended pelvic lymph node dissection in patients with clinically localized prostatic carcinoma. J Cancer Res Clin Oncol. 2014;140(2):243-256.
391 Gao Y, Liu X, Gu Y, et al. The Effect of Bisphosphonates on Fracture Healing Time and Changes in Bone Mass Density: A Meta-Analysis. Front Endocrinol (Lausanne). 2021;12:688269.
392 Garcia-Argibay M, Santed MA, Reales JM. Efficacy of binaural auditory beats in cognition, anxiety, and pain perception: a meta-analysis. Psychol Res. 2019;83(2):357-372.
393 Gattas DJ, Dan A, Myburgh J, et al. Fluid resuscitation with 6% hydroxyethyl starch (130/0.4) in acutely ill patients: an updated systematic review and meta-analysis. Anesth Analg. 2012;114(1):159-169.

394 Gdalevich M, Mimouni D, David M, Mimouni M. Breast-feeding and the onset of atopic dermatitis in childhood: a systematic review and meta-analysis of prospective studies. J Am Acad Dermatol. 2001;45(4):520-527.
395 Geng DF, Jin DM, Wu W, et al. Angiotensin receptor blockers for prevention of new-onset type 2 diabetes: a meta-analysis of 59,862 patients. Int J Cardiol. 2012;155(2):236-242.
396 Ghasemi Darestani N, Bahrami A, Mozafarian MR, et al. Association of Polyunsaturated Fatty Acid Intake on Inflammatory Gene Expression and Multiple Sclerosis: A Systematic Review and Meta-Analysis. Nutrients. 2022;14(21):4627.
397 Giglio M, Dalfino L, Puntillo F,et al. Hemodynamic goal-directed therapy and postoperative kidney injury: an updated meta-analysis with trial sequential analysis. Crit Care. 2019;23(1):232.
398 Gillanders SL, Anderson S, Mellon L, et al. A systematic review and meta-analysis: Do absorbable or non-absorbable suture materials differ in cosmetic outcomes in patients requiring primary closure of facial wounds?. J Plast Reconstr Aesthet Surg. 2018;71(12):1682-1692.
399 Gillespie LD, Robertson MC, Gillespie WJ, et al. Interventions for preventing falls in older people living in the community. Cochrane Database Syst Rev. 2009;(2):CD007146.
400 Godoi A, Reis Marques I, Padrão EMH, et al. Glucose control and psychosocial outcomes with use of automated insulin delivery for 12 to 96 weeks in type 1 diabetes: a meta-analysis of randomised controlled trials. Diabetol Metab Syndr. 2023;15(1):190.
401 Govindappagari S, Burwick RM. Treatment of Iron Deficiency Anemia in Pregnancy with Intravenous versus Oral Iron: Systematic Review and Meta-Analysis. Am J Perinatol. 2019;36(4):366-376.
402 Grosso G, Marventano S, Yang J, et al. A comprehensive meta-analysis on evidence of Mediterranean diet and cardiovascular disease: Are individual components equal?. Crit Rev Food Sci Nutr. 2017;57(15):3218-3232.
403 Gu L, Fu R, Chen P, et al. In Terms of Nutrition, the Most Suitable Method for Bariatric Surgery: Laparoscopic Sleeve Gastrectomy or Roux-en-Y Gastric Bypass? A Systematic Review and Meta-analysis. Obes Surg. 2020;30(5):2003-2014.
404 Gu L, Huang X, Li S, et al. A meta-analysis of the medium- and long-term effects of laparoscopic sleeve gastrectomy and laparoscopic Roux-en-Y gastric bypass. BMC Surg. 2020;20(1):30.
405 Güenaga K F, Matos D, Wille-Jørgensen P. Mechanical bowel preparation for elective colorectal surgery. Cochrane database of systematic reviews, 2011,7;2011(9):CD001544.
406 Güenaga K F, Matos D, Wille-Jørgensen P. Preoperative mechanical bowel preparation in elective colorectal surgery: an update of systematic review of the literature and meta-analysis. Journal of Coloproctology (Rio de Janeiro), 2012, 32: 7-17.
407 Gupta T, Thakkar P, Kalra B,et al. Hydroxychloroquine in the treatment of coronavirus disease 2019: Rapid updated systematic review and meta-analysis. Rev Med Virol. 2022;32(2):e2276.
408 Gurgel ST, do Nascimento P Jr. Maintaining tissue perfusion in high-risk surgical patients: a systematic review of randomized clinical trials. Anesth Analg. 2011;112(6):1384-1391.
409 Haas DM, Hathaway TJ, Ramsey PS. Progestogen for preventing miscarriage in women with recurrent miscarriage of unclear etiology. Cochrane Database Syst Rev. 2019;2019(11):CD003511.

410 Hansel J, Rogers AM, Lewis SR, et al. Videolaryngoscopy versus direct laryngoscopy for adults undergoing tracheal intubation. Cochrane Database Syst Rev. 2022;4(4):CD011136.
411 Hao G, Wang Z, Guo R, et al. Effects of ACEI/ARB in hypertensive patients with type 2 diabetes mellitus: a meta-analysis of randomized controlled studies. BMC Cardiovasc Disord. 2014;14:148.
412 Hariyanto TI, Halim DA, Rosalind J, et al. Ivermectin and outcomes from Covid-19 pneumonia: a systematic review and meta-analysis of randomized clinical trial studies. Rev Med Virol. 2022; 32(2):e2265.
413 Harvey H, Reissland N, Mason J. Parental reminder, recall and educational interventions to improve early childhood immunisation uptake: A systematic review and meta-analysis. Vaccine. 2015;33(25):2862-2880.
414 Hasan SU, Pervez A, Shah AA, et al. Safety outcomes of anti-platelet therapy post coronary artery bypass graft surgery: A systematic review and network meta-analysis of randomized control trials. Perfusion. 2024;39(4):684-697.
415 Hassanipour S, Arab-Zozani M, Amani B, et al. The efficacy and safety of Favipiravir in treatment of COVID-19: a systematic review and meta-analysis of clinical trials [published correction appears in Sci Rep. 2022;12(1):1996.
416 He D, Jiang F. Meta-analysis of letrozole versus clomiphene citrate in polycystic ovary syndrome. Reprod Biomed Online. 2011;23(1):91-96.
417 Helvaci A, Gok Metin Z. The effects of nurse-driven self-management programs on chronic obstructive pulmonary disease: A systematic review and meta-analysis. J Adv Nurs. 2020;76(11):2849-2871.
418 Henry DA, Carless PA, Moxey AJ, et al. Anti-fibrinolytic use for minimising perioperative allogeneic blood transfusion. Cochrane Database Syst Rev. 2011;(1):CD001886.
419 Hernandez AV, Phan MT, Rocco J, et al. Efficacy and Safety of Hydroxychloroquine for Hospitalized COVID-19 Patients: A Systematic Review and Meta-Analysis. J Clin Med. 2021;10(11):2503.
420 Hicks LK, Haynes AE, Reece DE, et al. A meta-analysis and systematic review of thalidomide for patients with previously untreated multiple myeloma. Cancer Treat Rev. 2008;34(5):442-452.
421 Horvath A, Dziechciarz P, Szajewska H. Meta-analysis: sequential therapy for Helicobacter pylori eradication in children. Aliment Pharmacol Ther. 2012;36(6):534-541.
422 Hosseini B, El Abd A, Ducharme FM. Effects of Vitamin D Supplementation on COVID-19 Related Outcomes: A Systematic Review and Meta-Analysis. Nutrients. 2022;14(10):2134.
423 Hou J, Xiong W, Cao L, et al. Spironolactone Add-on for Preventing or Slowing the Progression of Diabetic Nephropathy: A Meta-analysis. Clin Ther. 2015;37(9):2086-2103.e10.
424 Houston BL, Uminski K, Mutter T, et al. Efficacy and Safety of Tranexamic Acid in Major Non-Cardiac Surgeries at High Risk for Transfusion: A Systematic Review and Meta-Analysis. Transfus Med Rev. 2020;34(1):51-62.
425 Hovaguimian F, Lysakowski C, Elia N, et al. Effect of intraoperative high inspired oxygen fraction on surgical site infection, postoperative nausea and vomiting, and pulmonary function: systematic review and meta-analysis of randomized controlled trials. Anesthesiology. 2013;119(2):303-316.

426 Howe TE, Shea B, Dawson LJ, et al. Exercise for preventing and treating osteoporosis in postmenopausal women. Cochrane Database Syst Rev. 2011;(7):CD000333.
427 Hsu CK, Chen CY, Chen WC, et al. Effect of sofosbuvir-based treatment on clinical outcomes of patients with COVID-19: a systematic review and meta-analysis of randomised controlled trials. Int J Antimicrob Agents. 2022;59(3):106545.
428 Hu L, Wang Y, Liu X, et al. Tai Chi exercise can ameliorate physical and mental health of patients with knee osteoarthritis: systematic review and meta-analysis. Clin Rehabil. 2021;35(1):64-79.
429 Hu S, Yu Q, Wang Y, et al. Letrozole versus clomiphene citrate in polycystic ovary syndrome: a meta-analysis of randomized controlled trials. Arch Gynecol Obstet. 2018;297(5):1081-1088.
430 Hu Z, Han J, Jiao B, et al. Efficacy of Thoracolumbar Interfascial Plane Block for Postoperative Analgesia in Lumbar Spine Surgery: A Meta-analysis of Randomized Clinical Trials. Pain Physician. 2021;24(7):E1085-E1097.
431 Huang C, Lu TL, Lin L. Remdesivir Treatment Lacks the Effect on Mortality Reduction in Hospitalized Adult COVID-19 Patients Who Required High-Flow Supplemental Oxygen or Invasive Mechanical Ventilation. Medicina (Kaunas). 2023;59(6):1027.
432 Huang X, Leung F, Liu M, et al. Is helical blade superior to screw design in terms of cut-out rate for elderly trochanteric fractures? A meta-analysis of randomized controlled trials. Eur J Orthop Surg Traumatol. 2014;24(8):1461-1468.
433 Huang Y, Li F, Li C,et al. Efficacy of Low Molecular Weight Heparin in Preventing Perinatal Venous Thrombosis: A Meta-Analysis. Comput Math Methods Med. 2022;2022:1248577.
434 Huang Z, Chen J, Hu QS, et al. Meta-analysis of pain and function placebo responses in pharmacological osteoarthritis trials. Arthritis Res Ther. 2019;21(1):173.
435 Huang ZM, Xiao H, Ji ZG, et al. TVT versus TOT in the treatment of female stress urinary incontinence: a systematic review and meta-analysis. Ther Clin Risk Manag. 2018;14:2293-2303.
436 Hussain N, Grzywacz VP, Ferreri CA, et al. Investigating the Efficacy of Dexmedetomidine as an Adjuvant to Local Anesthesia in Brachial Plexus Block: A Systematic Review and Meta-Analysis of 18 Randomized Controlled Trials. Reg Anesth Pain Med. 2017;42(2):184-196.
437 Hwang SH, Park IJ, Cho YJ, et al. The efficacy of gabapentin/pregabalin in improving pain after tonsillectomy: A meta-analysis. Laryngoscope. 2016;126(2):357-366.
438 Iftikhar IH, Khan MF, Das A, et al. Meta-analysis: continuous positive airway pressure improves insulin resistance in patients with sleep apnea without diabetes . Ann Am Thorac Soc. 2013;10(2):115-120.
439 Indah Sari N N, Zamzam Zein A F M, Budhy T I, et al. The Role of Vitamin D Supplementation in Preventing Osteoporosis as Complication among Patients with Systemic Lupus Erythematosus: A Systematic Review of Clinical Trials. Malaysian Journal of Medicine & Health Sciences, 2023, 19.
440 Intzes S, Symeonidou M, Zagoridis K, et al. Hold your needles in women with recurrent pregnancy losses with or without hereditary thrombophilia: Meta-analysis and review of the literature. J Gynecol Obstet Hum Reprod. 2021;50(4):101935.
441 Irwinda R, Hiksas R, Lokeswara AW, et al. Vitamin D supplementation higher than 2000 IU/day compared to lower dose on maternal-fetal outcome: Systematic review and meta-analysis. Womens Health (Lond). 2022;18:17455057221111066.

442 Izcovich A, Peiris S, Ragusa M, et al. Bias as a source of inconsistency in ivermectin trials for COVID-19: A systematic review. Ivermectin's suggested benefits are mainly based on potentially biased results. J Clin Epidemiol. 2022;144:43-55.
443 Jacob M, Chappell D, Conzen P, et al. Small-volume resuscitation with hyperoncotic albumin: a systematic review of randomized clinical trials. Crit Care. 2008;12(2):R34.
444 Jacobson B, Rambiritch V, Paek D, et al. Safety and Efficacy of Enoxaparin in Pregnancy: A Systematic Review and Meta-Analysis. Adv Ther. 2020;37(1):27-40.
445 Jahanjoo F, Farshbaf-Khalili A, Shakouri SK, et al. Maternal and Neonatal Metabolic Outcomes of Vitamin D Supplementation in Gestational Diabetes Mellitus: A Systematic Review and Meta-Analysis. Ann Nutr Metab. 2018;73(2):145-159.
446 Jalota L, Kalira V, George E, et al. Prevention of pain on injection of propofol: systematic review and meta-analysis. BMJ. 2011;342:d1110.
447 Jamilian H, Amirani E, Milajerdi A, et al. The effects of vitamin D supplementation on mental health, and biomarkers of inflammation and oxidative stress in patients with psychiatric disorders: A systematic review and meta-analysis of randomized controlled trials. Prog Neuropsychopharmacol Biol Psychiatry. 2019;94:109651.
448 Jepsen DB, Thomsen K, Hansen S,et al. Effect of whole-body vibration exercise in preventing falls and fractures: a systematic review and meta-analysis. BMJ Open. 2017;7(12):e018342.
449 Jia D, Tan H, Faramand A, et al. One Anastomosis Gastric Bypass Versus Roux-en-Y Gastric Bypass for Obesity: a Systematic Review and Meta-Analysis of Randomized Clinical Trials. Obes Surg. 2020;30(4):1211-1218.
450 Jiang F, Hu X, Jiang K, et al. The role of low molecular weight heparin on recurrent pregnancy loss: A systematic review and meta-analysis. Taiwan J Obstet Gynecol. 2021;60(1):1-8.
451 Jie L, Li D, Yang C, Haiying Z. Tamoxifen versus clomiphene citrate for ovulation induction in infertile women. Eur J Obstet Gynecol Reprod Biol. 2018;228:57-64.
452 Jing C, Lin L, Zhou T,et al. Does Dexmedetomidine Reduce the Risk of Atrial Fibrillation and Stroke After Adult Cardiac Surgery? A Systematic Review and Meta-analysis of Randomized Controlled Trials. Anatol J Cardiol. 2022;26(5):354-365.
453 Jirarattanaphochai K, Jung S. Nonsteroidal antiinflammatory drugs for postoperative pain management after lumbar spine surgery: a meta-analysis of randomized controlled trials. J Neurosurg Spine. 2008;9(1):22-31.
454 Jorna L S, Spikman J M, Schoemaker R, et al. The efficacy of anti-inflammatory medication in postoperative cognitive decline: a meta-analysis. Journal of Neurology and Neuroscience, 2020, 11(3): 1-11.
455 Juhl C, Christensen R, Roos EM, et al. Impact of exercise type and dose on pain and disability in knee osteoarthritis: a systematic review and meta-regression analysis of randomized controlled trials. Arthritis Rheumatol. 2014;66(3):622-636.
456 Jull AB, Walker N, Deshpande S. Honey as a topical treatment for wounds. Cochrane Database Syst Rev. 2013;(2):CD005083.
457 Juul S, Nielsen EE, Feinberg J, et al. Interventions for treatment of COVID-19: Second edition of a living systematic review with meta-analyses and trial sequential analyses (The LIVING Project). PLoS One. 2021;16(3):e0248132.

458 Kaka AS, MacDonald R, Linskens EJ, et al. Major Update 2: Remdesivir for Adults With COVID-19: A Living Systematic Review and Meta-analysis for the American College of Physicians Practice Points. Ann Intern Med. 2022;175(5):701-709.
459 Kang JW, Lee MS, Posadzki P, et al. T'ai chi for the treatment of osteoarthritis: a systematic review and meta-analysis. BMJ Open. 2011;1(1):e000035.
460 Kang W, Cui Z, Chen Q, et al. Narrow band imaging-assisted transurethral resection reduces the recurrence risk of non-muscle invasive bladder cancer: A systematic review and meta-analysis. Oncotarget. 2017;8(14):23880-23890.
461 Karanicolas PJ, Smith SE, Kanbur B, et al. The impact of prophylactic dexamethasone on nausea and vomiting after laparoscopic cholecystectomy: a systematic review and meta-analysis. Ann Surg. 2008;248(5):751-762.
462 Kashour Z, Kashour T, Gerberi D, et al. Mortality, viral clearance, and other clinical outcomes of hydroxychloroquine in COVID-19 patients: A systematic review and meta-analysis of randomized controlled trials. Clin Transl Sci. 2021;14(3):1101-1112.
463 Ke Y, Zhu Y, Zhu M. A comparison of treatment effectiveness between clear aligner and fixed appliance therapies. BMC Oral Health. 2019;19(1):24.
464 Kearney RS, Parsons N, Metcalfe D, et al. Injection therapies for Achilles tendinopathy. Cochrane Database Syst Rev. 2015;2015(5):CD010960.
465 Kendall MC, Alves L, Traill LL,et al. The effect of ultrasound-guided erector spinae plane block on postsurgical pain: a meta-analysis of randomized controlled trials. BMC Anesthesiol. 2020;20(1):99.
466 Kim DH, Kim SW, Basurrah MA, et al. The Efficacy of Tranexamic Acid on the Postoperative Bleeding in Patients Receiving Head-and-Neck Surgery: A Meta-Analysis. Ear Nose Throat J.2023:1455613231155855
467 Kim KH, Lee MS, Kim TH, Kang JW, Choi TY, Lee JD. Acupuncture and related interventions for symptoms of chronic kidney disease. Cochrane Database Syst Rev. 2016;2016(6):CD009440.
468 Kim WO, Koo BN, Kim YK, et al. Ramosetron for the prevention of postoperative nausea and vomiting (PONV): a meta-analysis. Korean J Anesthesiol. 2011;61(5):405-412.
469 Kloping YP, Yogiswara N, Azmi Y. The role of preoperative dutasteride in reducing bleeding during transurethral resection of the prostate: A systematic review and meta-analysis of randomized controlled trials. Asian J Urol. 2022;9(1):18-26.
470 Kollmann M, Martins WP, Lima ML, et al. Strategies for improving outcome of assisted reproduction in women with polycystic ovary syndrome: systematic review and meta-analysis. Ultrasound Obstet Gynecol. 2016;48(6):709-718.
471 Kongnyuy EJ, Wiysonge CS. Interventions to reduce haemorrhage during myomectomy for fibroids. Cochrane Database Syst Rev. 2014;2014(8):CD005355.
472 Kongstad MB, Valentiner LS, Ried-Larsen M, et al. Effectiveness of remote feedback on physical activity in persons with type 2 diabetes: A systematic review and meta-analysis of randomized controlled trials. J Telemed Telecare. 2019;25(1):26-34.
473 Koo CH, Hwang JY, Shin HJ, et al. The Effects of Erector Spinae Plane Block in Terms of Postoperative Analgesia in Patients Undergoing Laparoscopic Cholecystectomy: A Meta-Analysis of Randomized Controlled Trials. J Clin Med. 2020;9(9):2928.
474 Kory P, Meduri GU, Varon J, et al. Review of the Emerging Evidence Demonstrating the Efficacy of Ivermectin in the Prophylaxis and Treatment of COVID-19. Am J Ther. 2021;28(3):e299-e318.

475 Kow CS, Javed A, Ramachandram D,et al. Clinical outcomes of sofosbuvir-based antivirals in patients with COVID-19: a systematic review and meta-analysis of randomized trials. Expert Rev Anti Infect Ther. 2022;20(4):567-575.
476 Kow CS, Merchant HA, Mustafa ZU, et al. The association between the use of ivermectin and mortality in patients with COVID-19: a meta-analysis. Pharmacol Rep. 2021;73(5):1473-1479.
477 Kranke P, Apfel CC, Eberhart LH, et al. The influence of a dominating centre on a quantitative systematic review of granisetron for preventing postoperative nausea and vomiting. Acta Anaesthesiol Scand. 2001;45(6):659-670.
478 Kranke P, Eberhart LH, Roewer N, et al. Single-dose parenteral pharmacological interventions for the prevention of postoperative shivering: a quantitative systematic review of randomized controlled trials. Anesth Analg. 2004;99(3):718-727.
479 Kujur M, Kiran KA, Nag AR, et al. Effect of Ivermectin prophylaxis in prevention of COVID 19: Meta-analysis and systematic review. J Family Med Prim Care. 2022;11(11):6660-6667.
480 Kumar A, Kharfan-Dabaja MA, Glasmacher A, et al. Tandem versus single autologous hematopoietic cell transplantation for the treatment of multiple myeloma: a systematic review and meta-analysis. J Natl Cancer Inst. 2009;101(2):100-106.
481 Kumar J, Jain S, Meena J, et al. Efficacy and safety of hydroxychloroquine/chloroquine against SARS-CoV-2 infection: A systematic review and meta-analysis. J Infect Chemother. 2021;27(6):882-889.
482 Kümmel LS, Krumbein H, Fragkou PC, et al. Vitamin D supplementation for the treatment of COVID-19: A systematic review and meta-analysis of randomized controlled trials. Front Immunol. 2022;13:1023903.
483 Kvam S, Kleppe CL, Nordhus IH, et al. Exercise as a treatment for depression: A meta-analysis. J Affect Disord. 2016;202:67-86.
484 Kwon Y, Ha J, Lee YH, et al. Comparative risk of anemia and related micronutrient deficiencies after Roux-en-Y gastric bypass and sleeve gastrectomy in patients with obesity: An updated meta-analysis of randomized controlled trials. Obes Rev. 2022;23(4):e13419.
485 Kwon Y, Lee S, Kim D, et al. Biliopancreatic Limb Length as a Potential Key Factor in Superior Glycemic Outcomes After Roux-en-Y Gastric Bypass in Patients With Type 2 Diabetes: A Meta-Analysis. Diabetes Care. 2022;45(12):3091-3100.
486 Lai D, Zhou S, Cheng S, et al. Laser therapy in the treatment of melasma: a systematic review and meta-analysis. Lasers Med Sci. 2022;37(4):2099-2110.
487 LAKSEMI D A Y U A S R I, TUNAS I K, WIDYADHARMA P E K A, et al. Ivermectin in the treatment of COVID-19 disease: a systematic review and meta-analysis. Int J App Pharm, 2022, 14(4): 18-25.
488 Landoni G, Biondi-Zoccai G, Greco M, et al. Effects of levosimendan on mortality and hospitalization. A meta-analysis of randomized controlled studies. Crit Care Med. 2012;40(2):634-646.
489 Landoni G, Mizzi A, Biondi-Zoccai G, et al. Levosimendan reduces mortality in critically ill patients. A meta-analysis of randomized controlled studies. Minerva Anestesiol. 2010;76(4):276-286.
490 Landoni G, Mizzi A, Biondi-Zoccai G, et al. Reducing mortality in cardiac surgery with levosimendan: a meta-analysis of randomized controlled trials. J Cardiothorac Vasc Anesth. 2010;24(1):51-57.

491 Landoni G, Turi S, Biondi-Zoccai G, et al. Esmolol reduces perioperative ischemia in noncardiac surgery: a meta-analysis of randomized controlled studies. J Cardiothorac Vasc Anesth. 2010;24(2):219-229.
492 Lang B, Zhang L, Lin Y, et al. Comparison of effects and safety in providing controlled hypotension during surgery between dexmedetomidine and magnesium sulphate: A meta-analysis of randomized controlled trials. PLoS One. 2020;15(1):e0227410.
493 Lara J, Evans EH, O'Brien N, et al. Association of behaviour change techniques with effectiveness of dietary interventions among adults of retirement age: a systematic review and meta-analysis of randomised controlled trials. BMC Med. 2014;12:177.
494 Latthe PM, Foon R, Toozs-Hobson P. Transobturator and retropubic tape procedures in stress urinary incontinence: a systematic review and meta-analysis of effectiveness and complications. BJOG. 2007;114(5):522-531.
495 Latthe PM, Singh P, Foon R, et al. Two routes of transobturator tape procedures in stress urinary incontinence: a meta-analysis with direct and indirect comparison of randomized trials. BJU Int. 2010;106(1):68-76.
496 Lee J, Park D, Koo KT, et al. Validity of a regenerative procedure for a minor bone defect with immediate implant placement: a systematic review and meta-analysis. Acta Odontol Scand. 2019;77(2):99-106.
497 Lee KC, Chen HH, Chung KC, et al. Meta-analysis of randomized controlled trials comparing outcomes for stapled hemorrhoidopexy versus LigaSure hemorrhoidectomy for symptomatic hemorrhoids in adults. Int J Surg. 2013;11(9):914-918.
498 Lee SY, Jung SH, Lee SU,et al. Can Bisphosphonates Prevent Recurrent Fragility Fractures? A Systematic Review and Meta-Analysis of Randomized Controlled Trials. J Am Med Dir Assoc. 2018;19(5):384-390.e1.
499 Lee TC, Murthy S, Del Corpo O, et al. Remdesivir for the treatment of COVID-19: a systematic review and meta-analysis. Clin Microbiol Infect. 2022;28(9):1203-1210.
500 Lee Y, Doumouras AG, Yu J, et al. Laparoscopic Sleeve Gastrectomy Versus Laparoscopic Roux-en-Y Gastric Bypass: A Systematic Review and Meta-analysis of Weight Loss, Comorbidities, and Biochemical Outcomes From Randomized Controlled Trials. Ann Surg. 2021;273(1):66-74.
501 Lee ZY, Ortiz-Reyes L, Lew CCH, et al. Intravenous vitamin C monotherapy in critically ill patients: a systematic review and meta-analysis of randomized controlled trials with trial sequential analysis. Ann Intensive Care. 2023;13(1):14.
502 Lehtinen ML, Harik L, Soletti G, et al. Sex differences in saphenous vein graft patency: A systematic review and meta-analysis. J Card Surg. 2022;37(12):4573-4578.
503 Lei D, Sha Y, Wen S, et al. Dexmedetomidine May Reduce IL-6 Level and the Risk of Postoperative Cognitive Dysfunction in Patients After Surgery: A Meta-Analysis. Dose Response. 2020;18(1):1559325820902345.
504 Lepine S, Jo J, Metwally M, et al. Ovarian surgery for symptom relief in women with polycystic ovary syndrome. Cochrane Database Syst Rev. 2017;11(11):CD009526.
505 Leslie K, Clavisi O, Hargrove J. Target-controlled infusion versus manually-controlled infusion of propofol for general anaesthesia or sedation in adults. Cochrane Database Syst Rev. 2008;(3):CD006059.
506 Lewkowitz AK, Gupta A, Simon L, et al. Intravenous compared with oral iron for the treatment of iron-deficiency anemia in pregnancy: a systematic review and meta-analysis. J Perinatol. 2019;39(4):519-532.

507 Li B, Kong I, McGrath M,et al. Evaluating the literature on preoperative androgen stimulation for hypospadias repair using the fragility index - can we trust observational studies?. J Pediatr Urol. 2021;17(5):661-669.
508 Li D, Cai Z, Pan Z, et al. The effects of vitamin and mineral supplementation on women with gestational diabetes mellitus. BMC Endocr Disord. 2021;21(1):106.
509 Li D, Wang C, Yang Z, et al. Effect of Intravenous Corticosteroids on Pain Management and Early Rehabilitation in Patients Undergoing Total Knee or Hip Arthroplasty: A Meta-Analysis of Randomized Controlled Trials. Pain Pract. 2018;18(4):487-499.
510 Li H, Cao Y, Ma P, et al. Novel Visualization Methods Assisted Transurethral Resection for Bladder Cancer: An Updated Survival-Based Systematic Review and Meta-Analysis. Front Oncol. 2021;11:644341.
511 Li J, Xiang QL, Zhu JX, et al. Comparison of enteral immunonutrition and enteral nutrition in patients undergoing gastric cancer surgery: a systematic review and meta-analysis of randomized, controlled trials. J Int Med Res. 2024;52(1):3000605231220870.
512 Li M, Yang Y, Ma Y, et al. Pharmacological Agents That Prevent Postoperative Cognitive Dysfunction in Patients With General Anesthesia: A Network Meta-analysis. Am J Ther. 2020;28(4):e420-e433.
513 Li P, Li LX, Zhao ZZ, et al. Dexmedetomidine reduces the incidence of postoperative delirium after cardiac surgery: a meta-analysis of randomized controlled trials. BMC Anesthesiol. 2021;21(1):153.
514 Li Q, Zhang Z, Cai Z. High-dose ketorolac affects adult spinal fusion: a meta-analysis of the effect of perioperative nonsteroidal anti-inflammatory drugs on spinal fusion. Spine (Phila Pa 1976). 2011;36(7):E461-E468.
515 Li S, Chang SM, Niu WX,et al. Comparison of tip apex distance and cut-out complications between helical blades and lag screws in intertrochanteric fractures among the elderly: a meta-analysis. J Orthop Sci. 2015;20(6):1062-1069.
516 Li T, Zeng J, Li DH, et al. Efficacy of intravenous vitamin C intervention for septic patients: A systematic review and meta-analysis based on randomized controlled trials. Am J Emerg Med. 2021;50:242-250.
517 Li W, Liu H, Yang C. Prophylactic dexmedetomidine use did not decrease the incidence of delirium in patients undergoing cardiac surgery: A meta-analysis. Perfusion. 2023;38(3):539-546.
518 Li X, Chang P, Wang Q, et al. Effects of Angiotensin-Converting Enzyme Inhibitors on Arterial Stiffness: A Systematic Review and Meta-Analysis of Randomized Controlled Trials. Cardiovasc Ther. 2020;2020:7056184.
519 Li X, Dai D, Chen B, et al. Efficacy of PI3K/AKT/mTOR pathway inhibitors for the treatment of advanced solid cancers: A literature-based meta-analysis of 46 randomised control trials. PLoS One. 2018;13(2):e0192464.
520 Li X, Hu X, Fu C, et al. Efficacy and Safety of One Anastomosis Gastric Bypass Versus Roux-en-Y Gastric Bypass for Obesity: a Meta-analysis and Systematic Review. Obes Surg. 2023;33(2):611-622.
521 Li X, Wang Y, Liu J, et al. Effects of perioperative interventions for preventing postoperative delirium: A protocol for systematic review and meta-analysis of randomized controlled trials. Medicine (Baltimore). 2021;100(29):e26662.
522 Li X, Zhao J. The influence of zinc supplementation on metabolic status in gestational diabetes: a meta-analysis of randomized controlled studies. J Matern Fetal Neonatal Med. 2021;34(13):2140-2145.

523 Liang B, Su J, Shao H, et al. The outcome of IV vitamin C therapy in patients with sepsis or septic shock: a meta-analysis of randomized controlled trials. Crit Care. 2023;27(1):109.
524 Liang H, Mu Q, Sun W, et al. Effect of intravenous vitamin C on adult septic patients: a systematic review and meta-analysis. Front Nutr. 2023;10:1211194.
525 Liang W, Zhang W, Wu Y, et al. Efficacy and safety of ultrasound-guided serratus anterior plane block for postoperative analgesia in thoracic surgery and breast surgery: A systematic review and meta-analysis of randomized controlled studies. Frontiers in Anesthesiology, 2022, 1: 980483.
526 Lin C, Ren Y, Lu A. The effectiveness of virtual reality games in improving cognition, mobility, and emotion in elderly post-stroke patients: a systematic review and meta-analysis. Neurosurg Rev. 2023;46(1):167.
527 Lin C, Tu H, Jie Z, et al. Effect of Dexmedetomidine on Delirium in Elderly Surgical Patients: A Meta-analysis of Randomized Controlled Trials. Ann Pharmacother. 2021;55(5):624-636.
528 Lin J, Zhang L, Yang H. Perioperative administration of selective cyclooxygenase-2 inhibitors for postoperative pain management in patients after total knee arthroplasty. J Arthroplasty. 2013;28(2):207-213.e2.
529 Lin L J, Liu J, Xu L Z, et al. The impact of endometrial mechanical stimulation in women with normal hysteroscopic findings undergoing IVF/ICSI: a meta-analysis. Clinical and Experimental Obstetrics & Gynecology, 2022, 49(1): 27.
530 Lin T, Yan SG, Cai XZ,et al. Alendronate versus Raloxifene for Postmenopausal Women: A Meta-Analysis of Seven Head-to-Head Randomized Controlled Trials. Int J Endocrinol. 2014;2014:796510.
531 Lin WF, Zhong MF, Zhou QH, et al. Efficacy of complementary and integrative medicine on health-related quality of life in cancer patients: a systematic review and meta-analysis. Cancer Manag Res. 2019;11:6663-6680.
532 Lin X, Nie Y. Pregnant Populations which Benefit from Vaginal Progesterone for Preventing Preterm Birth at <34 Weeks and Neonatal Morbidities: A Systematic Review and Meta-analysis. Am J Perinatol. 2024;41(1):1-16.
533 Ling Y, Xu F, Xia X, et al. Vitamin D supplementation reduces the risk of fall in the vitamin D deficient elderly: An updated meta-analysis. Clin Nutr. 2021;40(11):5531-5537.
534 Liu A, Zheng C, Lang J, et al. Letrozole versus clomiphene citrate for unexplained infertility: a systematic review and meta-analysis. J Obstet Gynaecol Res. 2014;40(5):1205-1216.
535 Liu C, Wang W, Shan Z, et al. Dexmedetomidine as an adjuvant for patients undergoing breast cancer surgery: A meta-analysis. Medicine (Baltimore). 2020;99(50):e23667.
536 Liu X, Liu J, Sun G. Preoperative intravenous glucocorticoids can reduce postoperative acute pain following total knee arthroplasty: A meta-analysis. Medicine (Baltimore). 2017;96(35):e7836.
537 Liu X, Men P, Wang B, et al. Effect of dipeptidyl-peptidase-4 inhibitors on C-reactive protein in patients with type 2 diabetes: a systematic review and meta-analysis. Lipids Health Dis. 2019;18(1):144.
538 Liu X, Men P, Wang Y,et al. Impact of dipeptidyl peptidase-4 inhibitors on serum adiponectin: a meta-analysis. Lipids Health Dis. 2016;15(1):204.

539 Liu Y, Shan N, Yuan Y, et al. The efficacy of enoxaparin for recurrent abortion: a meta-analysis of randomized controlled studies. J Matern Fetal Neonatal Med. 2021;34(3):473-478.
540 Liyanage T, Ninomiya T, Wang A, et al. Effects of the Mediterranean Diet on Cardiovascular Outcomes-A Systematic Review and Meta-Analysis. PLoS One. 2016;11(8):e0159252.
541 Loewen PS, Marra CA, Zed PJ. 5-HT3 receptor antagonists vs traditional agents for the prophylaxis of postoperative nausea and vomiting. Can J Anaesth. 2000;47(10):1008-1018.
542 Lu C, Liu Y, Jiang HL. Aspirin or heparin or both in the treatment of recurrent spontaneous abortion in women with antiphospholipid antibody syndrome: a meta-analysis of randomized controlled trials. J Matern Fetal Neonatal Med. 2019;32(8):1299-1311.
543 Lu Z, Wen F, Guo Q, et al. Radiofrequency ablation plus chemoembolization versus radiofrequency ablation alone for hepatocellular carcinoma: a meta-analysis of randomized-controlled trials. Eur J Gastroenterol Hepatol. 2013;25(2):187-194.
544 Luo L, Zhang Y, Wang H, et al. The efficacy of magnesium supplementation for gestational diabetes: A meta-analysis of randomized controlled trials. Eur J Obstet Gynecol Reprod Biol. 2024;293:84-90.
545 Lv M, Wang X, Qu W, et al. Nefopam for the prevention of perioperative shivering: a meta-analysis of randomized controlled trials. BMC Anesthesiol. 2015;15:87.
546 Lv Z, Qi H, Wang L, et al. Vitamin D status and Parkinson's disease: a systematic review and meta-analysis. Neurol Sci. 2014;35(11):1723-1730.
547 Ma C, Liu A, Sun M, et al Effect of whole-body vibration on reduction of bone loss and fall prevention in postmenopausal women: a meta-analysis and systematic review. J Orthop Surg Res. 2016;11:24.
548 Ma KL, Wang X, Luan FJ, et al. Proximal femoral nails antirotation, Gamma nails, and dynamic hip screws for fixation of intertrochanteric fractures of femur: A meta-analysis. Orthop Traumatol Surg Res. 2014;100(8):859-866.
549 Maagaard M, Barbateskovic M, Andersen-Ranberg NC, et al. Dexmedetomidine for the prevention of delirium in adults admitted to the intensive care unit or post-operative care unit: A systematic review of randomised clinical trials with meta-analysis and Trial Sequential Analysis. Acta Anaesthesiol Scand. 2023;67(4):382-411.
550 Machado GC, Maher CG, Ferreira PH, et al. Efficacy and safety of paracetamol for spinal pain and osteoarthritis: systematic review and meta-analysis of randomised placebo controlled trials. BMJ. 2015;350:h1225.
551 Machado GC, Maher CG, Ferreira PH, et al. Non-steroidal anti-inflammatory drugs for spinal pain: a systematic review and meta-analysis. Ann Rheum Dis. 2017;76(7):1269-1278.
552 Macpherson H, Pipingas A, Pase MP. Multivitamin-multimineral supplementation and mortality: a meta-analysis of randomized controlled trials. Am J Clin Nutr. 2013;97(2):437-444.
553 Maged AM, El-Mazny A, Lasheen Y,et al. Endometrial scratch injury in infertile women undergoing in vitro fertilization cycles: a systematic review and meta-analysis. J Int Med Res. 2023;51(7):3000605231175365.
554 Magouliotis DE, Tasiopoulou VS, Tzovaras G. One Anastomosis Gastric Bypass Versus Roux-en-Y Gastric Bypass for Morbid Obesity: an Updated Meta-Analysis. Obes Surg. 2019;29(9):2721-2730.

555 Maharaj R, Metaxa V. Levosimendan and mortality after coronary revascularisation: a meta-analysis of randomised controlled trials. Crit Care. 2011;15(3):R140.

556 Malihi Z, Wu Z, Mm Lawes C, et al. Noncalcemic adverse effects and withdrawals in randomized controlled trials of long-term vitamin D2 or D3 supplementation: a systematic review and meta-analysis. Nutr Rev. 2017;75(12):1007-1034.

557 Malihi Z, Wu Z, Stewart AW,et al. Hypercalcemia, hypercalciuria, and kidney stones in long-term studies of vitamin D supplementation: a systematic review and meta-analysis. Am J Clin Nutr. 2016;104(4):1039-1051.

558 Manouchehri E, Makvandi S, Razi M,et al. Efficient administration of a combination of nifedipine and sildenafil citrate versus only nifedipine on clinical outcomes in women with threatened preterm labor: a systematic review and meta-analysis. BMC Pediatr. 2024;24(1):106.

559 Mansournia MA, Ostadmohammadi V, Doosti-Irani A, et al. The Effects of Vitamin D Supplementation on Biomarkers of Inflammation and Oxidative Stress in Diabetic Patients: A Systematic Review and Meta-Analysis of Randomized Controlled Trials. Horm Metab Res. 2018;50(6):429-440.

560 Marques EM, Jones HE, Elvers KT, et al. Local anaesthetic infiltration for peri-operative pain control in total hip and knee replacement: systematic review and meta-analyses of short- and long-term effectiveness. BMC Musculoskelet Disord. 2014;15:220.

561 Martimbianco ALC, Pacheco RL, Bagattini ÂM, et al. Vitamin C-based regimens for sepsis and septic shock: Systematic review and meta-analysis of randomized clinical trials. J Crit Care. 2022;71:154099.

562 Martinez M P. Meta-Analysis with Meta-Regression and Systematic Review of the Efficacy of On-Demand Tramadol for the Treatment of Lifelong Premature Ejaculation. PJSS, 2013, 68(3).

563 Martinez-Gonzalez MA, Bes-Rastrollo M. Dietary patterns, Mediterranean diet, and cardiovascular disease . Curr Opin Lipidol. 2014;25(1):20-26.

564 Martínez-González MA, Dominguez LJ, Delgado-Rodríguez M. Olive oil consumption and risk of CHD and/or stroke: a meta-analysis of case-control, cohort and intervention studies. Br J Nutr. 2014;112(2):248-259.

565 Martínez-González MÁ, Hershey MS, Zazpe I, et al. Transferability of the Mediterranean Diet to Non-Mediterranean Countries. What Is and What Is Not the Mediterranean Diet. Nutrients. 2017;9(11):1226.

566 Martins, L.D., Rezende, M., Chibinski, et al. Does Ketorolac reduce the intensity of postoperative pain after impacted third molars surgery in adults compared to the use of tramadol? A systematic review and meta-analysis. Research, Society and Development. 2021,10(3),e19410313137.

567 Martyn-St James M, Cooper K, Kaltenthaler E, et al. Tramadol for premature ejaculation: a systematic review and meta-analysis. BMC urology, 2015, 15: 1-11.

568 Mastrolia SA, Novack L, Thachil J, et al. LMWH in the prevention of preeclampsia and fetal growth restriction in women without thrombophilia. A systematic review and meta-analysis. Thromb Haemost. 2016;116(5):868-878.

569 McGaughey TJ, Fletcher EA, Shah SA. Impact of Antihypertensive Agents on Central Systolic Blood Pressure and Augmentation Index: A Meta-Analysis. Am J Hypertens. 2016;29(4):448-457.

570 Mei S, Jin S, Chen Z, et al. Analgesia for total knee arthroplasty: a meta-analysis comparing local infiltration and femoral nerve block. Clinics (Sao Paulo). 2015;70(9):648-653.

571 Mekinian A, Cohen J, Alijotas-Reig J, et al. Unexplained Recurrent Miscarriage and Recurrent Implantation Failure: Is There a Place for Immunomodulation?. Am J Reprod Immunol. 2016;76(1):8-28.
572 Meng T, Hu SF, Cheng YQ, et al. Qigong for women with breast cancer: An updated systematic review and meta-analysis. Complement Ther Med. 2021;60:102743.
573 Meshkat S, Alnefeesi Y, Jawad MY, et al. Brain-Derived Neurotrophic Factor (BDNF) as a biomarker of treatment response in patients with Treatment Resistant Depression (TRD): A systematic review & meta-analysis. Psychiatry Res. 2022;317:114857.
574 Metwally M, Chatters R, Pye C, et al. Endometrial scratch to increase live birth rates in women undergoing first-time in vitro fertilisation: RCT and systematic review. Health Technol Assess. 2022;26(10):1-212.
575 Metwally M, Chatters R, White D, et al. Endometrial scratch in women undergoing first-time IVF treatment: a systematic review and meta-analysis of randomized controlled trials. Reprod Biomed Online. 2022;44(4):617-629.
576 Miao J, Bachmann KN, Huang S, et al. Effects of Vitamin D Supplementation on Cardiovascular and Glycemic Biomarkers. J Am Heart Assoc. 2021;10(10):e017727.
577 Michard F, Giglio MT, Brienza N. Perioperative goal-directed therapy with uncalibrated pulse contour methods: impact on fluid management and postoperative outcome. Br J Anaesth. 2017;119(1):22-30.
578 Mirhosseini N, Rainsbury J, Kimball SM. Vitamin D Supplementation, Serum 25(OH)D Concentrations and Cardiovascular Disease Risk Factors: A Systematic Review and Meta-Analysis. Front Cardiovasc Med. 2018;5:87.
579 Mishriky BM, Habib AS. Metoclopramide for nausea and vomiting prophylaxis during and after Caesarean delivery: a systematic review and meta-analysis. Br J Anaesth. 2012;108(3):374-383.
580 Misso ML, Wong JL, Teede HJ, et al. Aromatase inhibitors for PCOS: a systematic review and meta-analysis. Hum Reprod Update. 2012;18(3):301-312.
581 MM Asla, AA Nawar, E Elsayed, et al. Vitamin D on COVID19 Patients During the Pandemic, 2022. A Systematic Review and MetaAnalysis. Current Research in Nutrition & Food Science. 2023; 11(1):37-60.
582 Mohamed HHB, Serag Eldien AM, Zahran A. Augmentation versus No Augmentation for Immediate Postextraction Implants. Int J Dent. 2018;2018:5209108.
583 Mohammadifard N, Salehi-Abargouei A, Salas-Salvadó J, et al. The effect of tree nut, peanut, and soy nut consumption on blood pressure: a systematic review and meta-analysis of randomized controlled clinical trials. Am J Clin Nutr. 2015;101(5):966-982.
584 Moll E, van der Veen F, van Wely M. The role of metformin in polycystic ovary syndrome: a systematic review. Hum Reprod Update. 2007;13(6):527-537.
585 Montroy J, Fergusson NA, Hutton B, et al. The Safety and Efficacy of Lysine Analogues in Cancer Patients: A Systematic Review and Meta-Analysis. Transfus Med Rev. 2017;31(3):141-148.
586 Moraes VY, Marra AR, Matos LL, et al. Hydroxychloroquine for treatment of COVID-19 patients: a systematic review and meta-analysis of randomized controlled trials. Einstein (Sao Paulo). 2022;20:eRW0045.

587 Moran GW, Kurtzman JT, Carpenter CP. Biologic adjuvant urethral coverings for single-stage primary hypospadias repairs: A systematic review and pooled proportional meta-analysis of postoperative urethrocutaneous fistulas. J Pediatr Urol. 2022;18(5):598-608.
588 Motamed S, Nikooyeh B, Anari R, et al. The effect of vitamin D supplementation on oxidative stress and inflammatory biomarkers in pregnant women: a systematic review and meta-analysis of clinical trials. BMC Pregnancy Childbirth. 2022;22(1):816.
589 Mousa A, Naderpoor N, Teede H, et al. Vitamin D supplementation for improvement of chronic low-grade inflammation in patients with type 2 diabetes: a systematic review and meta-analysis of randomized controlled trials. Nutr Rev. 2018;76(5):380-394.
590 Moya E, Phiri N, Choko AT,et al. Effect of postpartum anaemia on maternal health-related quality of life: a systematic review and meta-analysis. BMC Public Health. 2022;22(1):364.
591 Muhammad M, Jahangir A, Kassem A, et al. The Role and Efficacy of Vitamin C in Sepsis: A Systematic Review and Meta-Analysis. Adv Respir Med. 2022;90(4):281-299.
592 Murad M H, Elamin K B, Abu Elnour N O, et al. The effect of vitamin D on falls: a systematic review and meta-analysis. The Journal of Clinical Endocrinology & Metabolism, 2011, 96(10): 2997-3006.
593 Mutter TC, Ruth CA, Dart AB. Hydroxyethyl starch (HES) versus other fluid therapies: effects on kidney function. Cochrane Database Syst Rev. 2013;(7):CD007594.
594 Nasioudis D, Kim SW, Schoen C, et al. Maternal and neonatal outcomes with mechanical cervical dilation plus misoprostol compared to misoprostol alone for cervical ripening; a systematic review of literature and metaanalysis. Am J Obstet Gynecol MFM. 2019;1(2):101-111.
595 Natale P, Ruospo M, Saglimbene VM, et al. Interventions for improving sleep quality in people with chronic kidney disease. Cochrane Database Syst Rev. 2019;5(5):CD012625.
596 Nazari S S H, Karimi R, Mohammadian M, et al. Effects of Remdesivir and Favipiravir on Covid-19 Clinical Outcomes: A Systematic Review and Meta-Analysis. Biomedical Research and Therapy, 10(5), 5701-5716.
597 Ng TK, Wong DFK. The efficacy of cognitive behavioral therapy for Chinese people: A meta-analysis. Aust N Z J Psychiatry. 2018;52(7):620-637.
598 Ni JY, Liu SS, Xu LF, et al. Meta-analysis of radiofrequency ablation in combination with transarterial chemoembolization for hepatocellular carcinoma. World J Gastroenterol. 2013;19(24):3872-3882.
599 Noel JE, Kligerman MP, Megwalu UC. Intraoperative Corticosteroids for Voice Outcomes among Patients Undergoing Thyroidectomy: A Systematic Review and Meta-analysis. Otolaryngol Head Neck Surg. 2018;159(5):811-816.
600 Nosè M, Ballette F, Bighelli I, et al. Psychosocial interventions for post-traumatic stress disorder in refugees and asylum seekers resettled in high-income countries: Systematic review and meta-analysis. PLoS One. 2017;12(2):e0171030.
601 Nunes R, Santos-Sousa H, Vieira S, et al. Vitamin B Complex Deficiency After Roux-en-Y Gastric Bypass and Sleeve Gastrectomy-a Systematic Review and Meta-Analysis. Obes Surg. 2022;32(3):873-891.
602 Oh C, Keats EC, Bhutta ZA. Vitamin and Mineral Supplementation During Pregnancy on Maternal, Birth, Child Health and Development Outcomes in Low- and Middle-Income Countries: A Systematic Review and Meta-Analysis. Nutrients. 2020;12(2):491.

603 Ojo O, Weldon SM, Thompson T, et al. The Effect of Vitamin D Supplementation on Glycaemic Control in Women with Gestational Diabetes Mellitus: A Systematic Review and Meta-Analysis of Randomised Controlled Trials. Int J Environ Res Public Health. 2019;16(10):1716.
604 Oktavian P, Budi DS, Wibowo IN, et al. Automated glycemic control with a bionic pancreas for type 1 diabetes mellitus: A systematic review and meta-analysis. Diabetes Metab Syndr. 2023;17(9):102847.
605 Oliva Morgado Ferreira R, Trevisan T, Pasqualotto E, et al. Efficacy of the hybrid closedloop insulin delivery system in children and adolescents with type 1 diabetes: a meta-analysis with trial sequential analysis. Arch Endocrinol Metab. 2024;68:e230280.
606 Oliveira LC, Oliveira RG, Pires-Oliveira DA. Effects of whole body vibration on bone mineral density in postmenopausal women: a systematic review and meta-analysis. Osteoporos Int. 2016;27(10):2913-33.
607 O'Meara S, Al-Kurdi D, Ologun Y, et al. Antibiotics and antiseptics for venous leg ulcers. Cochrane Database Syst Rev. 2014;2014(1):CD003557.
608 Onakpoya IJ, Spencer EA, Thompson MJ, et al. The effect of chlorogenic acid on blood pressure: a systematic review and meta-analysis of randomized clinical trials. J Hum Hypertens. 2015;29(2):77-81.
609 Ornat L, Alonso-Ventura V, Bueno-Notivol J, et al. Misoprostol combined with cervical single or double balloon catheters versus misoprostol alone for labor induction of singleton pregnancies: a meta-analysis of randomized trials. J Matern Fetal Neonatal Med. 2020;33(20):3453-3468.
610 Ortiz-Seller D, Panach-Navarrete J, Valls-González L, Martínez-Jabaloyas JM. Comparison between open and minimally invasive pyeloplasty in infants: A systematic review and meta-analysis. J Pediatr Urol. 2024;20(2):244-252.
611 Osland EJ, Yunus RM, Khan S,et al. Five-Year Weight Loss Outcomes in Laparoscopic Vertical Sleeve Gastrectomy (LVSG) Versus Laparoscopic Roux-en-Y Gastric Bypass (LRYGB) Procedures: A Systematic Review and Meta-Analysis of Randomized Controlled Trials. Surg Laparosc Endosc Percutan Tech. 2020;30(6):542-553.
612 Ostadmohammadi V, Milajerdi A, Ghayour-Mobarhan M, et al. The Effects of Vitamin D Supplementation on Glycemic Control, Lipid Profiles and C-Reactive Protein Among Patients with Cardiovascular Disease: a Systematic Review and Meta-Analysis of Randomized Controlled Trials. Curr Pharm Des. 2019;25(2):201-210.
613 Özlüşen B, Kozan Ş, Akcan RE, et al. Effectiveness of favipiravir in COVID-19: a live systematic review. Eur J Clin Microbiol Infect Dis. 2021;40(12):2575-2583.
614 Pal R, Banerjee M, Bhadada SK, et al. Vitamin D supplementation and clinical outcomes in COVID-19: a systematic review and meta-analysis. J Endocrinol Invest. 2022;45(1):53-68.
615 Palacios C, De-Regil LM, Lombardo LK,et al. Vitamin D supplementation during pregnancy: Updated meta-analysis on maternal outcomes. J Steroid Biochem Mol Biol. 2016;164:148-155.
616 Palacios C, Kostiuk LK, Peña-Rosas JP. Vitamin D supplementation for women during pregnancy. Cochrane Database Syst Rev. 2019;7(7):CD008873.
617 Palma-Duran SA, Vlassopoulos A, Lean M, et al. Nutritional intervention and impact of polyphenol on glycohemoglobin (HbA1c) in non-diabetic and type 2 diabetic subjects: Systematic review and meta-analysis. Crit Rev Food Sci Nutr. 2017;57(5):975-986.

618 Pamilih A T, Tamtomo D G, Murti B. The effectiveness of ivermectin on the risk of mortality in COVID-19 patients: a meta analysis. Journal of Epidemiology and Public Health, 2021, 6(4): 402-414.
619 Panin SI, Sazhin VP. Improvement of Russian clinical guidelines and reduction of mortality in perforated ulcers. Khirurgiia (Mosk). 2024;(2):5-13.
620 Papageorgiou SN, Koletsi D, Iliadi A, et al. Treatment outcome with orthodontic aligners and fixed appliances: a systematic review with meta-analyses. Eur J Orthod. 2020;42(3):331-343.
621 Partap U, Chowdhury R, Taneja S, et al. Preconception and periconception interventions to prevent low birth weight, small for gestational age and preterm birth: a systematic review and meta-analysis. BMJ Glob Health. 2022;7(8):e007537.
622 Patel A, Laffan MA, Waheed U, et al. Randomised trials of human albumin for adults with sepsis: systematic review and meta-analysis with trial sequential analysis of all-cause mortality . BMJ. 2014;349:g4561.
623 Patel JJ, Ortiz-Reyes A, Dhaliwal R, et al. IV Vitamin C in Critically Ill Patients: A Systematic Review and Meta-Analysis. Crit Care Med. 2022;50(3):e304-e312.
624 Patel M, Onwochei DN, Desai N. Influence of perioperative dexmedetomidine on the incidence of postoperative delirium in adult patients undergoing cardiac surgery. Br J Anaesth. 2022;129(1):67-83.
625 Patel NN, Rogers CA, Angelini GD, et al. Pharmacological therapies for the prevention of acute kidney injury following cardiac surgery: a systematic review. Heart Fail Rev. 2011;16(6):553-567.
626 Peng K, Liu HY, Wu SR, et al. Effects of Combining Dexmedetomidine and Opioids for Postoperative Intravenous Patient-controlled Analgesia: A Systematic Review and Meta-analysis. Clin J Pain. 2015;31(12):1097-1104.
627 Peng K, Zhang J, Meng XW, et al. Optimization of Postoperative Intravenous Patient-Controlled Analgesia with Opioid-Dexmedetomidine Combinations: An Updated Meta-Analysis with Trial Sequential Analysis of Randomized Controlled Trials. Pain Physician. 2017;20(7):569-596.
628 Peng S, Wang J, Yu H,et al. Influence of Dexmedetomidine on Post-operative Atrial Fibrillation After Cardiac Surgery: A Meta-Analysis of Randomized Controlled Trials. Front Cardiovasc Med. 2021;8:721264.
629 Pérez-López FR, Pasupuleti V, Mezones-Holguin E, et al. Effect of vitamin D supplementation during pregnancy on maternal and neonatal outcomes: a systematic review and meta-analysis of randomized controlled trials. Fertil Steril. 2015;103(5):1278-88.e4.
630 Ping Y, Ye Q, Wang W, et al. Dexmedetomidine as an adjuvant to local anesthetics in brachial plexus blocks: A meta-analysis of randomized controlled trials. Medicine (Baltimore). 2017;96(4):e5846.
631 Plantinga NL, de Smet AMGA, Oostdijk EAN, et al. Selective digestive and oropharyngeal decontamination in medical and surgical ICU patients: individual patient data meta-analysis. Clin Microbiol Infect. 2018;24(5):505-513.
632 Ploutarchou G, Savva C, Karagiannis C, et al. The effectiveness of cognitive behavioural therapy in chronic neck pain: A systematic review with meta-analysis. Cogn Behav Ther. 2023;52(5):523-563.
633 Poeze M, Greve JW, Ramsay G. Meta-analysis of hemodynamic optimization: relationship to methodological quality. Crit Care. 2005;9(6):R771-R779.

634 Polderman JA, Farhang-Razi V, Van Dieren S, et al. Adverse side effects of dexamethasone in surgical patients. Cochrane Database Syst Rev. 2018;8(8):CD011940.
635 Poot CC, Meijer E, Kruis AL, et al. Integrated disease management interventions for patients with chronic obstructive pulmonary disease. Cochrane Database Syst Rev. 2021;9(9):CD009437.
636 Popp M, Reis S, Schießer S, et al. Ivermectin for preventing and treating COVID-19. Cochrane Database Syst Rev. 2022;6(6):CD015017.
637 Pramono I, Siregar S, Sibarani J. Preoperative hormonal stimulation in hypospadias: a systematic review. Annals of African Surgery, 2023, 20(1): 14-26.
638 Pripp AH. Effect of peptides derived from food proteins on blood pressure: a meta-analysis of randomized controlled trials. Food Nutr Res. 2008;52.
639 Punjasawadwong Y, Phongchiewboon A, Bunchungmongkol N. Bispectral index for improving anaesthetic delivery and postoperative recovery. Cochrane Database Syst Rev. 2014;2014(6):CD003843.
640 Purgato M, Richards J, Prina E, et al. Efficacy of physical activity interventions on psychological outcomes in refugee, asylum seeker and migrant populations: a systematic review and meta-analysis. Psychology of Sport and Exercise, 2021, 54: 101901.
641 Qian H, Lei T, Hu Y. Negative pressure wound therapy versus gauze dressings in managing open fracture wound of lower limbs: A meta-analysis of randomized controlled trials. Foot Ankle Surg. 2022;28(7):1120-1128.
642 Qiao L, Xu C, Li X, et al. Heart calcium sensitizer on morbidity and mortality of high-risk surgical patients with MODS: systematic review and meta-analysis. Int J Clin Exp Med. 2015;8(10):17712-17720.
643 Qin C, Jiang Y, Lin C, et al. Perioperative dexmedetomidine administration to prevent delirium in adults after non-cardiac surgery: A systematic review and meta-analysis. J Clin Anesth. 2021;73:110308.
644 Qin F, Zhou Y, Huan L, et al. Comparison of clomiphene and letrozole for superovulation in patients with unexplained infertility undergoing intrauterine insemination: A systematic review and meta-analysis. Medicine (Baltimore). 2020;99(31):e21006.
645 Qing W, Shi X, Zhang Q, et al. Effect of Therapeutic Ultrasound for Neck Pain: A Systematic Review and Meta-Analysis. Arch Phys Med Rehabil. 2021;102(11):2219-2230.
646 Qiu R, Li J, Xiao Y, et al. The therapeutic effect and safety of the drugs for COVID-19: A systematic review and meta-analysis. Medicine (Baltimore). 2021;100(16):e25532.
647 Quah GS, Eslick GD, Cox MR. Laparoscopic Repair for Perforated Peptic Ulcer Disease Has Better Outcomes Than Open Repair. J Gastrointest Surg. 2019;23(3):618-625.
648 Ramadhani M Z, Renaldo J. Pre-Operative Hormonal Administration in Hypospadias Patients Undergoing Urethroplasty. Folia Medica Indonesiana, 2022, 58(1): 80-87.
649 Rasmark Roepke E, Hellgren M, Hjertberg R, et al. Treatment efficacy for idiopathic recurrent pregnancy loss - a systematic review and meta-analyses. Acta Obstet Gynecol Scand. 2018;97(8):921-941.
650 Requena A, Herrero J, Landeras J, et al. Use of letrozole in assisted reproduction: a systematic review and meta-analysis. Hum Reprod Update. 2008;14(6):571-582.

651 Ridgewell C, Heaton KJ, Hildebrandt A, et al. The effects of transcutaneous auricular vagal nerve stimulation on cognition in healthy individuals: A meta-analysis. Neuropsychology. 2021;35(4):352-365.
652 Rijs K, Mercier FJ, Lucas DN,et al. Fluid loading therapy to prevent spinal hypotension in women undergoing elective caesarean section: Network meta-analysis, trial sequential analysis and meta-regression. Eur J Anaesthesiol. 2020;37(12):1126-1142.
653 Roberts I, Blackhall K, Alderson P,et al. Human albumin solution for resuscitation and volume expansion in critically ill patients. Cochrane Database Syst Rev. 2011;2011(11):CD001208.
654 Rodrigues MRK, Lima SAM, Mazeto GMFDS, et al. Efficacy of vitamin D supplementation in gestational diabetes mellitus: Systematic review and meta-analysis of randomized trials. PLoS One. 2019;14(3):e0213006.
655 Rogan S, de Bruin E D, Radlinger L, et al. Effects of whole-body vibration on proxies of muscle strength in old adults: a systematic review and meta-analysis on the role of physical capacity level. European Review of Aging and Physical Activity, 2015, 12: 1-26.
656 Rollins KE, Lobo DN. Intraoperative Goal-directed Fluid Therapy in Elective Major Abdominal Surgery: A Meta-analysis of Randomized Controlled Trials. Ann Surg. 2016;263(3):465-476.
657 Romero R, Conde-Agudelo A, El-Refaie W, et al. Vaginal progesterone decreases preterm birth and neonatal morbidity and mortality in women with a twin gestation and a short cervix: an updated meta-analysis of individual patient data. Ultrasound Obstet Gynecol. 2017;49(3):303-314.
658 Roque M, Lattes K, Serra S, et al. Fresh embryo transfer versus frozen embryo transfer in in vitro fertilization cycles: a systematic review and meta-analysis. Fertil Steril. 2013;99(1):156-162.
659 Roque M, Tostes AC, Valle M, et al. Letrozole versus clomiphene citrate in polycystic ovary syndrome: systematic review and meta-analysis. Gynecol Endocrinol. 2015;31(12):917-921.
660 Rösner S, Leucht S, Lehert P, et al. Acamprosate supports abstinence, naltrexone prevents excessive drinking: evidence from a meta-analysis with unreported outcomes. J Psychopharmacol. 2008;22(1):11-23.
661 Rotella F, Cassioli E, Falone A, et al. Homeopathic Remedies in Psychiatric Disorders: A Meta-analysis of Randomized Controlled Trials. J Clin Psychopharmacol. 2020;40(3):269-275.
662 Ruan J, Gong X, Kong J, et al. Effect of B vitamin (folate, B6, and B12) supplementation on osteoporotic fracture and bone turnover markers: a meta-analysis. Med Sci Monit. 2015;21:875-881.
663 Rubens FD, Fergusson D, Wells PS, et al. Platelet-rich plasmapheresis in cardiac surgery: a meta-analysis of the effect on transfusion requirements. J Thorac Cardiovasc Surg. 1998;116(4):641-647.
664 Russell JA, Navickis RJ, Wilkes MM. Albumin versus crystalloid for pump priming in cardiac surgery: meta-analysis of controlled trials. J Cardiothorac Vasc Anesth. 2004;18(4):429-437.
665 Saarto T, Wiffen PJ. Antidepressants for neuropathic pain. Cochrane Database Syst Rev. 2007;2007(4):CD005454.
666 Saccone G, Berghella V. Antenatal corticosteroids for maturity of term or near term fetuses: systematic review and meta-analysis of randomized controlled trials [published correction appears in BMJ. 2016;355:i6416.

667 Saccone G, Schoen C, Franasiak JM, et al. Supplementation with progestogens in the first trimester of pregnancy to prevent miscarriage in women with unexplained recurrent miscarriage: a systematic review and meta-analysis of randomized, controlled trials. Fertil Steril. 2017;107(2):430-438.e3.
668 Sahebi R, Rezayi M, Emadzadeh M, et al. The effects of vitamin D supplementation on indices of glycemic control in Iranian diabetics: A systematic review and meta-analysis. Complement Ther Clin Pract. 2019;34:294-304.
669 Sahebkar A, Ponzo V, Bo S. Effect of Dipeptidyl Peptidase-4 Inhibitors on Plasma Adiponectin: A Systematic Review and Meta-Analysis of Randomized Controlled Trials. Curr Med Chem. 2016;23(13):1356-1369.
670 Sathianathen NJ, Hwang EC, Mian R, et al. Selective serotonin re-uptake inhibitors for premature ejaculation in adult men. Cochrane Database Syst Rev. 2021;3(3):CD012799.
671 Sato R, Hasegawa D, Prasitlumkum N, et al. Effect of IV High-Dose Vitamin C on Mortality in Patients With Sepsis: A Systematic Review and Meta-Analysis of Randomized Controlled Trials. Crit Care Med. 2021;49(12):2121-2130.
672 Schnabel A, Eberhart LH, Muellenbach R, et al. Efficacy of perphenazine to prevent postoperative nausea and vomiting: a quantitative systematic review. Eur J Anaesthesiol. 2010;27(12):1044-1051.
673 Seangleulur A, Vanasbodeekul P, Prapaitrakool S, et al. The efficacy of local infiltration analgesia in the early postoperative period after total knee arthroplasty: A systematic review and meta-analysis. Eur J Anaesthesiol. 2016;33(11):816-831.
674 Sebastian SA, Padda I, Johal G. Long-term impact of mediterranean diet on cardiovascular disease prevention: A systematic review and meta-analysis of randomized controlled trials. Curr Probl Cardiol. 2024;49(5):102509.
675 Sepidarkish M, Farsi F, Akbari-Fakhrabadi M, et al. The effect of vitamin D supplementation on oxidative stress parameters: A systematic review and meta-analysis of clinical trials. Pharmacol Res. 2019;139:141-152.
676 Shafiee A, Teymouri Athar MM, Kohandel Gargari O, et al. Ivermectin under scrutiny: a systematic review and meta-analysis of efficacy and possible sources of controversies in COVID-19 patients. Virol J. 2022;19(1):102.
677 Shahin Y, Barnes R, Barakat H, et al. Meta-analysis of angiotensin converting enzyme inhibitors effect on walking ability and ankle brachial pressure index in patients with intermittent claudication. Atherosclerosis. 2013;231(2):283-290.
678 Shahin Y, Khan JA, Chetter I. Angiotensin converting enzyme inhibitors effect on arterial stiffness and wave reflections: a meta-analysis and meta-regression of randomised controlled trials. Atherosclerosis. 2012;221(1):18-33.
679 Shahin Y, Mazari F, Chetter I. Do angiotensin converting enzyme inhibitors improve walking distance in patients with symptomatic lower limb arterial disease? A systematic review and meta-analysis of randomised controlled trials. Int J Surg. 2011;9(3):209-213.
680 Shang Y, Li H, Ma J, et al. Colloid preloading versus crystalloid preloading to prevent hypotension after spinal anesthesia for cesarean delivery: A protocol for systematic review and meta-analysis. Medicine (Baltimore). 2021;100(7):e24607.
681 Sharafi SM, Yazdi M, Goodarzi-Khoigani M,et al. Effect of Vitamin D Supplementation on Serum 25-Hydroxyvitamin D and Homeostatic Model of Insulin Resistance Levels in Healthy Pregnancy: A Systematic Review and Meta-Analysis. Iran J Med Sci. 2023;48(1):4-12.
682 Sharpe A, Morley LC, Tang T, et al. Metformin for ovulation induction (excluding gonadotrophins) in women with polycystic ovary syndrome. Cochrane Database Syst Rev. 2019;12(12):CD013505.

683 Sharples LD, Clutterbuck-James AL, Glover MJ, et al. Meta-analysis of randomised controlled trials of oral mandibular advancement devices and continuous positive airway pressure for obstructive sleep apnoea-hypopnoea. Sleep Med Rev. 2016;27:108-124.
684 Shen L, Zhang Y, Shen Y,et al. Antirotation proximal femoral nail versus dynamic hip screw for intertrochanteric fractures: a meta-analysis of randomized controlled studies. Orthop Traumatol Surg Res. 2013;99(4):377-383.
685 Shen QH, Li HF, Zhou XY,et al. Dexmedetomidine in the prevention of postoperative delirium in elderly patients following non-cardiac surgery: A systematic review and meta-analysis. Clin Exp Pharmacol Physiol. 2020;47(8):1333-1341.
686 Shepherd J, Jones J, Frampton G, et al. Clinical effectiveness and cost-effectiveness of depth of anaesthesia monitoring (E-Entropy, Bispectral Index and Narcotrend): a systematic review and economic evaluation. Health Technol Assess. 2013 Aug;17(34):1-264.
687 Shi C, Dumville JC, Cullum N, et al. Compression bandages or stockings versus no compression for treating venous leg ulcers. Cochrane Database Syst Rev. 2021;7(7):CD013397.
688 Shi C, Tang J, Li W,et al. Comparative Efficacy of Breastfeeding or Feeding of Breast Milk on Blood Sampling Pain Relief in Full-Term Neonates: A Systematic Review and Meta-Analysis. Breastfeed Med. 2024;19(2):81-90.
689 Shi XY, Zou Z, He XY, et al. Hydroxyethyl starch for cardiovascular surgery: a systematic review of randomized controlled trials. Eur J Clin Pharmacol. 2011;67(8):767-782.
690 Shields GS, Spahr CM, Slavich GM. Psychosocial Interventions and Immune System Function: A Systematic Review and Meta-analysis of Randomized Clinical Trials. JAMA Psychiatry. 2020;77(10):1031-1043.
691 Shih YW, Wang MH, Monsen KA, et al. Effectiveness of Acupuncture for Relieving Chemotherapy-Induced Bone Marrow Suppression: A Systematic Review with a Meta-analysis and Trial Sequential Analysis. J Integr Complement Med. 2023;29(10):621-636.
692 should remdesivir be used in the treatment of children with covid-19 infection?
693 Shrestha DB, Budhathoki P, Sedhai YR, et al. Vitamin C in Critically Ill Patients: An Updated Systematic Review and Meta-Analysis. Nutrients. 2021;13(10):3564.
694 Si HB, Yang TM, Zeng Y, et al. No clear benefit or drawback to the use of closed drainage after primary total knee arthroplasty: a systematic review and meta-analysis. BMC Musculoskelet Disord. 2016;17:183.
695 Si XY, Wu LP, Li XD,et al. Dexamethasone combined with other antiemetics for prophylaxis after laparoscopic cholecystectomy. Asian J Surg. 2015;38(1):21-27.
696 Simental-Mendia LE, Sahebkar A, Rodriguez-Moran M, et al. Effect of Magnesium Supplementation on Plasma C-reactive Protein Concentrations: A Systematic Review and Meta-Analysis of Randomized Controlled Trials. Curr Pharm Des. 2017;23(31):4678-4686.
697 Singh B, Ryan H, Kredo T, et al. Chloroquine or hydroxychloroquine for prevention and treatment of COVID-19. Cochrane Database Syst Rev. 2021;2(2):CD013587.
698 Singhal AK, Kannan S, Gota VS. 5HT3 antagonists for prophylaxis of postoperative nausea and vomiting in breast surgery: a meta-analysis. J Postgrad Med. 2012;58(1):23-31.

699 Siongco PRL, Rosales RL, Moore AP, et al. Botulinum neurotoxin injections for muscle-based (dystonia and spasticity) and non-muscle-based (neuropathic pain) pain disorders: a meta-analytic study. J Neural Transm (Vienna). 2020;127(6):935-951.
700 Slatkovska L, Alibhai SM, Beyene J, et al. Effect of whole-body vibration on BMD: a systematic review and meta-analysis. Osteoporos Int. 2010;21(12):1969-1980.
701 Smith CA, Hill E, Denejkina A, et al. The effectiveness and safety of complementary health approaches to managing postpartum pain: A systematic review and meta-analysis. Integr Med Res. 2022;11(1):100758.
702 Som A, Maitra S, Bhattacharjee S,et al. Goal directed fluid therapy decreases postoperative morbidity but not mortality in major non-cardiac surgery: a meta-analysis and trial sequential analysis of randomized controlled trials. J Anesth. 2017;31(1):66-81.
703 Song X, Jia H, Jiang Y, et al. Anti-atherosclerotic effects of the glucagon-like peptide-1 (GLP-1) based therapies in patients with type 2 Diabetes Mellitus: A meta-analysis. Sci Rep. 2015;5:10202.
704 Song YP, Wang L, Yuan BF, et al. Negative-pressure wound therapy for III/IV pressure injuries: A meta-analysis. Wound Repair Regen. 2021;29(1):20-33.
705 Sotiriadis A, Makrydimas G, Papatheodorou S, et al. Corticosteroids for preventing neonatal respiratory morbidity after elective caesarean section at term. Cochrane Database Syst Rev. 2018;8(8):CD006614.
706 Srinivasjois R, Silva D. Antenatal steroid administration in medically uncomplicated pregnancy beyond 37 weeks of gestation for the prevention of neonatal morbidities prior to elective caesarean section: a systematic review and meta-analysis of randomised controlled trials. J Matern Fetal Neonatal Med. 2017;30(10):1151-1157.
707 Stubbs B, Vancampfort D, Rosenbaum S, et al Challenges Establishing the Efficacy of Exercise as an Antidepressant Treatment: A Systematic Review and Meta-Analysis of Control Group Responses in Exercise Randomised Controlled Trials. Sports Med. 2016;46(5):699-713.
708 Su D, McBride C, Zhou J, et al. Does nutritional counseling in telemedicine improve treatment outcomes for diabetes? A systematic review and meta-analysis of results from 92 studies. J Telemed Telecare. 2016;22(6):333-347.
709 Suijkerbuijk YB, Schaafsma FG, van Mechelen JC, et al. Interventions for obtaining and maintaining employment in adults with severe mental illness, a network meta-analysis. Cochrane Database Syst Rev. 2017;9(9):CD011867.
710 Sun X, Yang Q, Sun F, et al. Comparison between the retropubic and transobturator approaches in the treatment of female stress urinary incontinence: a systematic review and meta-analysis of effectiveness and complications. Int Braz J Urol. 2015;41(2):220-229.
711 Sun Y, Chai F, Pan C, et al. Effect of perioperative goal-directed hemodynamic therapy on postoperative recovery following major abdominal surgery-a systematic review and meta-analysis of randomized controlled trials. Crit Care. 2017;21(1):141.
712 Sun Y, Chen X, Qiao J, et al. Effects of Transcutaneous Neuromuscular Electrical Stimulation on Swallowing Disorders: A Systematic Review and Meta-Analysis. Am J Phys Med Rehabil. 2020;99(8):701-711.
713 Tan H, Zhou Z, Cui Y, et al. A systematic review and meta-analysis of randomized controlled trials of "on-demand" use of tramadol vs "on-demand" use of paroxetine in the management of patients with premature ejaculation. Int J Clin Pract. 2021;75(11):e14825.

714 Tan PF, Yang LL, Ou RB, et al. Effectiveness and complication rates of tension-free vaginal tape, transobturator tape, and tension-free vaginal tape-obturator in the treatment of female stress urinary incontinence in a medium- to long-term follow up. Meta-analysis of randomized controlled trials. Saudi Med J. 2014;35(1):20-32.
715 Tan S, Wu G, Zhuang Q, et al. Laparoscopic versus open repair for perforated peptic ulcer: A meta analysis of randomized controlled trials. Int J Surg. 2016;33 Pt A:124-132.
716 Tan X, Hu Jbo. ACEIs/ARBs for the prevention of type 2 diabetes in patients with cardiovascular diseases: a systematic review and meta-analysis. Int J Clin Exp Med. 2016;9(6):9628-9641
717 Tan X, Huang Y. Magnesium supplementation for glycemic status in women with gestational diabetes: a systematic review and meta-analysis. Gynecol Endocrinol. 2022;38(3):202-206.
718 Tanaka R, Ozawa J, Umehara T, et al. Exercise intervention to improve the bone mineral density and bone metabolic markers as risk factors for fracture in Japanese subjects with osteoporosis: a systematic review and meta-analysis of randomized controlled trials. Journal of Physical Therapy Science, 2012, 24(12): 1349-1353.
719 Tassi A, Parisi N, Londero AP. Misoprostol administration prior to intrauterine contraceptive device insertion: a systematic review and meta-analysis of randomised controlled trials. Eur J Contracept Reprod Health Care. 2020;25(1):76-86.
720 Thy M, Montmayeur J, Julien-Marsollier F, et al. Safety and efficacy of peri-operative administration of hydroxyethyl starch in children undergoing surgery: A systematic review and meta-analysis. Eur J Anaesthesiol. 2018;35(7):484-495.
721 Ting B, Tsai CL, Hsu WT, et al. Music Intervention for Pain Control in the Pediatric Population: A Systematic Review and Meta-Analysis. J Clin Med. 2022;11(4):991.
722 Titirungruang C, Seresirikachorn K, Kasemsuwan P, et al. The use of steroids to reduce complications after tonsillectomy: a systematic review and meta-analysis of randomized controlled studies. Eur Arch Otorhinolaryngol. 2019;276(2):585-604.
723 Tocci G, Paneni F, Palano F, et al. Angiotensin-converting enzyme inhibitors, angiotensin II receptor blockers and diabetes: a meta-analysis of placebo-controlled clinical trials. Am J Hypertens. 2011;24(5):582-590.
724 Tran KC, Leung AA, Tang KL,et al. Efficacy of Calcium Channel Blockers on Major Cardiovascular Outcomes for the Treatment of Hypertension in Asian Populations: A Meta-analysis. Can J Cardiol. 2017;33(5):635-643.
725 Tsai Z, Shah N, Tahir U, et al. Dietary interventions for perinatal depression and anxiety: a systematic review and meta-analysis of randomized controlled trials. Am J Clin Nutr. 2023;117(6):1130-1142.
726 Turner JM, Robertson NT, Hartel G, et al. Impact of low-dose aspirin on adverse perinatal outcome: meta-analysis and meta-regression. Ultrasound Obstet Gynecol. 2020;55(2):157-169.
727 Turrini G, Purgato M, Acarturk C, et al. Efficacy and acceptability of psychosocial interventions in asylum seekers and refugees: systematic review and meta-analysis. Epidemiol Psychiatr Sci. 2019;28(4):376-388.
728 Unanyan A, Pivazyan L, Krylova E, et al. Effectiveness of inositol, metformin and their combination in women with PCOS undergoing assisted reproduction: systematic review and meta-analysis. Gynecol Endocrinol. 2022;38(12):1035-1046.

729 Valenzuela D A R, Caro A B. Use of intra-operative supplemental oxygen to reduce morbidity and mortality in general anesthesia: systematic review and meta-analysis of randomized controlled trials. Colombian Journal of Anesthesiology, 2012, 40(1): 34-51.
730 van Hoogenhuijze NE, Kasius JC, Broekmans FJM, et al. Endometrial scratching prior to IVF; does it help and for whom? A systematic review and meta-analysis. Hum Reprod Open. 2019;2019(1):hoy025.
731 van Vark LC, Bertrand M, Akkerhuis KM, et al. Angiotensin-converting enzyme inhibitors reduce mortality in hypertension: a meta-analysis of randomized clinical trials of renin-angiotensin-aldosterone system inhibitors involving 158,998 patients. Eur Heart J. 2012;33(16):2088-2097.
732 Varikasuvu SR, Thangappazham B, Vykunta A, et al. COVID-19 and vitamin D (Co-VIVID study): a systematic review and meta-analysis of randomized controlled trials. Expert Rev Anti Infect Ther. 2022;20(6):907-913.
733 Vasamsetti D B, Pachava S, Talluri D. Remineralizing Agents in the Prevention of Caries: A Systematic Review and Meta-analysis of Randomized Controlled Trials. Journal of Orofacial Sciences, 2021, 13(2): 121-128.
734 Vejakama P, Thakkinstian A, Lertrattananon D, et al. Reno-protective effects of renin-angiotensin system blockade in type 2 diabetic patients: a systematic review and network meta-analysis. Diabetologia. 2012;55(3):566-578.
735 Vellekkatt F, Menon V. Efficacy of vitamin D supplementation in major depression: A meta-analysis of randomized controlled trials. J Postgrad Med. 2019;65(2):74-80.
736 Veronese N, Pizzol D, Smith L, et al. Effect of Magnesium Supplementation on Inflammatory Parameters: A Meta-Analysis of Randomized Controlled Trials. Nutrients. 2022;14(3):679.
737 Vignon Zomahoun HT, de Bruin M, Guillaumie L, et al. Effectiveness and Content Analysis of Interventions to Enhance Oral Antidiabetic Drug Adherence in Adults with Type 2 Diabetes: Systematic Review and Meta-Analysis. Value Health. 2015;18(4):530-540.
738 Viguiliouk E, Kendall CW, Blanco Mejia S, et al. Effect of tree nuts on glycemic control in diabetes: a systematic review and meta-analysis of randomized controlled dietary trials. PLoS One. 2014;9(7):e103376.
739 Vitagliano A, Andrisani A, Alviggi C, et al. Endometrial scratching for infertile women undergoing a first embryo transfer: a systematic review and meta-analysis of published and unpublished data from randomized controlled trials. Fertil Steril. 2019;111(4):734-746.e2.
740 Volk T, Peters J, Sessler DI. The WHO recommendation for 80% perioperative oxygen is poorly justified. Anaesthesist. 2017;66(4):227-229.
741 Volpe M, Tocci G, Sciarretta S,et al. Angiotensin II receptor blockers and myocardial infarction: an updated analysis of randomized clinical trials. J Hypertens. 2009;27(5):941-946.
742 Vorobeichik L, Brull R, Abdallah FW. Evidence basis for using perineural dexmedetomidine to enhance the quality of brachial plexus nerve blocks: a systematic review and meta-analysis of randomized controlled trials. Br J Anaesth. 2017;118(2):167-181.
743 Wanas Y, Bashir R, Islam N, et al. Assessing the risk of angiotensin receptor blockers on major cardiovascular events: a systematic review and meta-analysis of randomized controlled trials. BMC Cardiovasc Disord. 2020;20(1):188.
744 Wang B, He KH, Jiang MB,et al. Effect of prophylactic dexamethasone on nausea and vomiting after laparoscopic gynecological operation: meta-analysis. Middle East J Anaesthesiol. 2011;21(3):397-402.

745 Wang CF, Sun YL, Zang HX. Music therapy improves sleep quality in acute and chronic sleep disorders: a meta-analysis of 10 randomized studies. Int J Nurs Stud. 2014;51(1):51-62.

746 Wang G, Zhang R, Li C,et al. Evaluation of the effect of low molecular weight heparin in unexplained recurrent pregnancy loss: a meta-analysis of randomized controlled trials. J Matern Fetal Neonatal Med. 2022;35(25):7601-7608.

747 Wang JP, Zhang YM, Yang RJ, et al. Efficacy and safety of active abdominal compression-decompression versus standard CPR for cardiac arrests: A systematic review and meta-analysis of 17 RCTs. Int J Surg. 2019;71:132-139.

748 Wang K, Wu M, Xu J, et al. Effects of dexmedetomidine on perioperative stress, inflammation, and immune function: systematic review and meta-analysis. Br J Anaesth. 2019;123(6):777-794.

749 Wang M, Chen Z, Hu Y, et al. The effects of vitamin D supplementation on glycemic control and maternal-neonatal outcomes in women with established gestational diabetes mellitus: A systematic review and meta-analysis. Clin Nutr. 2021;40(5):3148-3157.

750 Wang T, Dong L, Cong X, et al. Comparative efficacy of non-invasive neurostimulation therapies for poststroke dysphagia: A systematic review and meta-analysis. Neurophysiol Clin. 2021;51(6):493-506.

751 Wang W, Liu J, Ye H, et al. Effect of Dexmedetomidine on Tachyarrhythmias After Cardiac Surgery: A Systematic Review and Meta-Analysis. J Cardiovasc Pharmacol. 2022;79(3):315-324.

752 Wang X, Gu J, Liu J,et al. Clinical evidence for acupressure with the improvement of sleep disorders in hemodialysis patients: A systematic review and meta-analysis. Complement Ther Clin Pract. 2020;39:101151.

753 Wang X, Liu N, Chen J, et al. Effect of Intravenous Dexmedetomidine During General Anesthesia on Acute Postoperative Pain in Adults: A Systematic Review and Meta-Analysis of Randomized Controlled Trials. Clin J Pain. 2018;34(12):1180-1191.

754 Wang Y, Bu X, Zhao N, et al. Dexmedetomidine effect on delirium in elderly patients undergoing general anesthesia: A protocol for systematic review and meta-analysis. Medicine (Baltimore). 2021;100(48):e27782.

755 Weiss NS, van Vliet MN, Limpens J, et al. Endometrial thickness in women undergoing IUI with ovarian stimulation. How thick is too thin? A systematic review and meta-analysis. Hum Reprod. 2017;32(5):1009-1018.

756 Wen C, Li Y, Hu Q, et al. IV Vitamin C in Sepsis: A Latest Systematic Review and Meta-Analysis. Int J Clin Pract. 2023;2023:6733465.

757 Wilkes MM, Navickis RJ, Sibbald WJ. Albumin versus hydroxyethyl starch in cardiopulmonary bypass surgery: a meta-analysis of postoperative bleeding. Ann Thorac Surg. 2001;72(2):527-534.

758 Williams ACC, Fisher E, Hearn L, et al. Psychological therapies for the management of chronic pain (excluding headache) in adults. Cochrane Database Syst Rev. 2020;8(8):CD007407.

759 Wilson, S., Cramp, F. Combining a psychological intervention with physiotherapy: A systematic review to determine the effect on physical function and quality of life for adults with chronic pain.Physical Therapy Reviews, 2018, 23(3): 214-226.

760 Wu C, Bai R, Yan W, et al. Clinical Outcomes of One Anastomosis Gastric Bypass Versus Sleeve Gastrectomy for Morbid Obesity. Obes Surg. 2020;30(3):1021-1031.

761 Wu C, Song Y, Wang X. Vitamin D Supplementation for the Outcomes of Patients with Gestational Diabetes Mellitus and Neonates: A Meta-Analysis and Systematic Review. Int J Clin Pract. 2023;2023:1907222.
762 Wu CK, Tseng PT, Wu MK, et al. Antidepressants during and after Menopausal Transition: A Systematic Review and Meta-Analysis [published correction appears in Sci Rep. 2022;12(1):14781.
763 Wu H, Pang Q. The effect of vitamin D and calcium supplementation on falls in older adults : A systematic review and meta-analysis. Einfluss der Vitamin-D- und Kalziumsupplementierung auf Stürze bei älteren Erwachsenen : Eine systematische Übersicht und Metaanalyse. Orthopade. 2017;46(9):729-736.
764 Wu H, Zhang S, Lin X, et al. Pregnancy-related complications and perinatal outcomes following progesterone supplementation before 20 weeks of pregnancy in spontaneously achieved singleton pregnancies: a systematic review and meta-analysis. Reprod Biol Endocrinol. 2021;19(1):165.
765 Wu L, Deng SB, She Q. Calcium channel blocker compared with angiotensin receptor blocker for patients with hypertension: a meta-analysis of randomized controlled trials. J Clin Hypertens (Greenwich). 2014;16(11):838-845.
766 Wu T, Wu Y, Chen S, et al. Curative Effect and Survival Assessment Comparing Gemcitabine and Cisplatin Versus Methotrexate, Vinblastine, Doxorubicin and Cisplatin as Neoadjuvant Therapy for Bladder Cancer: A Systematic Review and Meta-Analysis. Front Oncol. 2021;11:678896.
767 Wu T, Yue X, Duan X, et al. Efficacy and safety of tramadol for premature ejaculation: a systematic review and meta-analysis. Urology. 2012;80(3):618-624.
768 Xiang F, Lin Y, Chen B. Ticagrelor for patients undergoing coronary artery bypass grafting: A meta-analysis of randomized controlled trials. Perfusion. 2023;38(4):698-705.
769 Xiang YY, Chen Q, Tang XX,et al. Comparison of the effect of double-lumen endotracheal tubes and bronchial blockers on lung collapse in video-assisted thoracoscopic surgery: a systematic review and meta-analysis. BMC Anesthesiol. 2022;22(1):330.
770 Xie F, Huang T, Lou D, et al. Effect of vitamin D supplementation on the incidence and prognosis of depression: An updated meta-analysis based on randomized controlled trials. Front Public Health. 2022;10:903547.
771 Xing LZ, Li L, Zhang LJ. Can intravenous steroid administration reduce postoperative pain scores following total knee arthroplasty?: A meta-analysis. Medicine (Baltimore). 2017;96(24):e7134.
772 Xiong X, Chen D, Shi J. Is Perioperative Dexmedetomidine Associated With a Reduced Risk of Perioperative Neurocognitive Disorders Following Cardiac Surgery? A Systematic Review and Meta-Analysis With Trial Sequential Analysis of Randomized Controlled Trials. Front Med (Lausanne). 2021;8:645975.
773 Xiu H, Zhang Y, Shan Z, et al. Effects of dexmedetomidine as a local anesthetic adjuvant for brachial plexus block: a systematic review and meta-analysis. Int J Clin Exp Med, 10(1), 357-366.
774 Xu C, Yi T, Tan S, et al. Association of Oral or Intravenous Vitamin C Supplementation with Mortality: A Systematic Review and Meta-Analysis. Nutrients. 2023;15(8):1848.
775 Xu H, Yi H, Guan J, et al. Effect of continuous positive airway pressure on lipid profile in patients with obstructive sleep apnea syndrome: a meta-analysis of randomized controlled trials. Atherosclerosis. 2014;234(2):446-453.

776 Xu L, Xie X, Gu X. Dexamethasone for preventing postoperative nausea and vomiting after mastectomy. Medicine (Baltimore). 2020;99(30):e21417.
777 Xu Y, Wu H, Lu X. Influence of continuous positive airway pressure on lipid profiles of obstructive sleep apnea: A systematic review and meta-analysis. Medicine (Baltimore). 2022;101(42):e31258.
778 Yan S, Xu D, Sun B. Combination of radiofrequency ablation with transarterial chemoembolization for hepatocellular carcinoma: a meta-analysis. Dig Dis Sci. 2012;57(11):3026-3031.
779 Yan X, Wang D, Yan P,et al. Low molecular weight heparin or LMWH plus aspirin in the treatment of unexplained recurrent miscarriage with negative antiphospholipid antibodies: A meta-analysis of randomized controlled trial. Eur J Obstet Gynecol Reprod Biol. 2022;268:22-30.
780 Yan X, Zhou FX, Lan T, et al. Optimal postoperative nutrition support for patients with gastrointestinal malignancy: A systematic review and meta-analysis. Clin Nutr. 2017;36(3):710-721.
781 Yang J, Cui PJ, Han HZ, et al. Meta-analysis of stapled hemorrhoidopexy vs LigaSure hemorrhoidectomy. World J Gastroenterol. 2013;19(29):4799-4807.
782 Yang L, Qian S, Liu H, et al. Role of tramadol in premature ejaculation: a systematic review and meta-analysis. Urol Int. 2013;91(2):197-205.
783 Yang L, Stanworth S, Hopewell S, et al. Is fresh-frozen plasma clinically effective? An update of a systematic review of randomized controlled trials. Transfusion. 2012;52(8):1673.
784 Yang M, Lin L, Sha C, et al. Which is better for mothers and babies: fresh or frozen-thawed blastocyst transfer?. BMC Pregnancy Childbirth. 2020;20(1):559.
785 Yang S, Jiang Q, Li H. The role of telenursing in the management of diabetes: a systematic review and meta-analysis. Public Health Nursing, 2019, 36(4): 575-586.
786 Yang W, Liu Y, Zhang Y, et al. Effect of intra-operative high inspired oxygen fraction on surgical site infection: a meta-analysis of randomized controlled trials. J Hosp Infect. 2016;93(4):329-338.
787 Yano Y, Briasoulis A, Bakris GL, et al. Effects of antihypertensive treatment in Asian populations: a meta-analysis of prospective randomized controlled studies (CARdiovascular protectioN group in Asia: CARNA). J Am Soc Hypertens. 2014;8(2):103-116.
788 Yao Y, Li B, Xu Y, Yang L, Zou B, Wang L. East Asian patients who received immunotherapy-based therapy associated with improved survival benefit in advanced non-small cell lung cancer: An updated meta-analysis. Cancer Med. 2024;13(4):e7080.
789 Ye J, Mani R. A Systematic Review and Meta-Analysis of Nutritional Supplementation in Chronic Lower Extremity Wounds. Int J Low Extrem Wounds. 2016;15(4):296-302.
790 Ye Y, Bi Y, Ma J, et al. Thoracolumbar interfascial plane block for postoperative analgesia in spine surgery: A systematic review and meta-analysis. PLoS One. 2021;16(5):e0251980.
791 Yin Si, Xing Dai, Dangfeng Zhang,et al.Is proximal femoral nail antirotation superior to gamma nail and dynamic hip screw in treatment of intertrochanteric fractures? A pairwise and network meta-analysis. Int J Clin. 2016,12: 22993-23007.
792 Yong S, Suping L, Peng Z, et al. The effects of vitamin C supplementation in the critically ill patients outcomes: A systematic review and meta-analysis of randomized controlled trials. Medicine (Baltimore). 2024;103(12):e37420.

793 Yoshida Y, Boren SA, Soares J, et al. Effect of Health Information Technologies on Glycemic Control Among Patients with Type 2 Diabetes. Curr Diab Rep. 2018;18(12):130.
794 Yu C, Hequn C, Jinbo C,et al. Gemcitabine/cisplatin versus methotrexate/vinblastine/doxorubicin/cisplatin for muscle-invasive bladder cancer: A systematic review and meta-analysis. J Cancer Res Ther. 2018;14(6):1260-1265.
795 Yu K, Zheng X, Wang G, et al. Immunonutrition vs Standard Nutrition for Cancer Patients: A Systematic Review and Meta-Analysis (Part 1). JPEN J Parenter Enteral Nutr. 2020;44(5):742-767.
796 Yu Q, Hu S, Wang Y, et al. Letrozole versus laparoscopic ovarian drilling in clomiphene citrate-resistant women with polycystic ovary syndrome: a systematic review and meta-analysis of randomized controlled trials. Reprod Biol Endocrinol. 2019;17(1):17.
797 Yu SK, Tait G, Karkouti K, et al. The safety of perioperative esmolol: a systematic review and meta-analysis of randomized controlled trials. Anesth Analg. 2011;112(2):267-281.
798 Yu X, Wang H, Duan X, et al. Intramedullary versus extramedullary internal fixation for unstable intertrochanteric fracture, a meta-analysis. Acta Orthop Traumatol Turc. 2018;52(4):299-307.
799 Yu Y, Tian L, Xiao Y, et al. Effect of Vitamin D Supplementation on Some Inflammatory Biomarkers in Type 2 Diabetes Mellitus Subjects: A Systematic Review and Meta-Analysis of Randomized Controlled Trials. Ann Nutr Metab. 2018;73(1):62-73.
800 Yuan J, Sun Y, Pan C,et al. Goal-directed fluid therapy for reducing risk of surgical site infections following abdominal surgery - A systematic review and meta-analysis of randomized controlled trials. Int J Surg. 2017;39:74-87.
801 Yuan L, Wu H, Huang W, et al. The function of metformin in endometrial receptivity (ER) of patients with polycyclic ovary syndrome (PCOS): a systematic review and meta-analysis. Reprod Biol Endocrinol. 2021;19(1):89.
802 Yue C, Wei R, Liu Y. Perioperative systemic steroid for rapid recovery in total knee and hip arthroplasty: a systematic review and meta-analysis of randomized trials. J Orthop Surg Res. 2017;12(1):100.
803 Zamani M, Nikbaf-Shandiz M, Aali Y, et al. The effects of acarbose treatment on cardiovascular risk factors in impaired glucose tolerance and diabetic patients: a systematic review and dose-response meta-analysis of randomized clinical trials. Front Nutr. 2023;10:1084084.
804 Zamani M, Sohrabi Z, Aghakhani L, et al. The effects of vitamin D and omega-3 co-supplementation on lipid profile in adults: a systematic review and meta-analysis. Nutrition & Food Science, 2024, 54(2): 285-301.
805 Zangrillo A, Biondi-Zoccai G, Ponschab M, et al. Milrinone and mortality in adult cardiac surgery: a meta-analysis. J Cardiothorac Vasc Anesth. 2012;26(1):70-77.
806 Zangrillo A, Turi S, Crescenzi G, et al. Esmolol reduces perioperative ischemia in cardiac surgery: a meta-analysis of randomized controlled studies. J Cardiothorac Vasc Anesth. 2009;23(5):625-632.
807 Zarychanski R, Abou-Setta AM, Turgeon AF, et al. Association of hydroxyethyl starch administration with mortality and acute kidney injury in critically ill patients requiring volume resuscitation: a systematic review and meta-analysis. JAMA. 2013;309(7):678-688.
808 Zarychanski R, Turgeon AF, Fergusson DA, et al. Renal outcomes and mortality following hydroxyethyl starch resuscitation of critically ill patients: systematic review and meta-analysis of randomized trials. Open Med. 2009;3(4):e196-e209.

809 Zein AFMZ, Sulistiyana CS, Raffaelo WM,et al. Ivermectin and mortality in patients with COVID-19: A systematic review, meta-analysis, and meta-regression of randomized controlled trials. Diabetes Metab Syndr. 2021;15(4):102186.
810 Zeng C, Wang YR, Wei J, et al. Treatment of trochanteric fractures with proximal femoral nail antirotation or dynamic hip screw systems: a meta-analysis. J Int Med Res. 2012;40(3):839-851.
811 Zeng H, Li Z, He J, et al. Dexmedetomidine for the prevention of postoperative delirium in elderly patients undergoing noncardiac surgery: A meta-analysis of randomized controlled trials. PLoS One. 2019;14(8):e0218088.
812 Zeng Y, Liu Z, Xu F, et al. Intravenous high-dose vitamin C monotherapy for sepsis and septic shock: A meta-analysis of randomized controlled trials. Medicine (Baltimore). 2023;102(42):e35648.
813 Zhai Q, Wang Y, Yuan Z, et al. Effects of platelet-rich plasmapheresis during cardiovascular surgery: A meta-analysis of randomized controlled clinical trials. J Clin Anesth. 2019;56:88-97.
814 Zhang B, Yao X, Zhong X, et al. Vitamin D supplementation in the treatment of polycystic ovary syndrome: A meta-analysis of randomized controlled trials. Heliyon. 2023;9(3):e14291.
815 Zhang J, Gao R, Cao P,et al. Additive effects of antiresorptive agents and exercise on lumbar spine bone mineral density in adults with low bone mass: a meta-analysis. Osteoporos Int. 2014;25(5):1585-1594.
816 Zhang J, Zhang X, Wang H,et al. Dexmedetomidine as a neuraxial adjuvant for prevention of perioperative shivering: Meta-analysis of randomized controlled trials. PLoS One. 2017;12(8):e0183154.
817 Zhang L, Zhu J, Xu L, et al. Efficacy and safety of flurbiprofen axetil in the prevention of pain on propofol injection: a systematic review and meta-analysis. Med Sci Monit. 2014;20:995-1002.
818 Zhang Q, Fu Y, Lu Y, et al. Impact of Virtual Reality-Based Therapies on Cognition and Mental Health of Stroke Patients: Systematic Review and Meta-analysis. J Med Internet Res. 2021;23(11):e31007.
819 Zhang Q, Huo Q, Chen P, et al. Effects of white noise on preterm infants in the neonatal intensive care unit: A meta-analysis of randomised controlled trials. Nurs Open. 2024;11(1):e2094.
820 Zhang Y, Huang L, Su Y, et al. The Effects of Traditional Chinese Exercise in Treating Knee Osteoarthritis: A Systematic Review and Meta-Analysis. PLoS One. 2017;12(1):e0170237.
821 Zhang Y, Shan Z, Kuang L, et al. The effect of different doses of intrathecal dexmedetomidine on spinal anesthesia: A meta analysis. Int J Clin Exp Med, 2016, 9(10): 18860-86.
822 Zhao C, Liu S, Zhang H,et al. Does dexmedetomidine reduce the risk of acute kidney injury after cardiac surgery? A meta-analysis of randomized controlled trials. Braz J Anesthesiol. 2024;74(3):744446.
823 Zhao D, Song J, Gao X, et al. Selective oropharyngeal decontamination versus selective digestive decontamination in critically ill patients: a meta-analysis of randomized controlled trials. Drug Des Devel Ther. 2015;9:3617-3624.
824 Zhao J, Sui X, Shi Q,et al. Effects of antioxidant intervention in patients with polycystic ovarian syndrome: A systematic review and meta-analysis. Medicine (Baltimore). 2022;101(32):e30006.

825 Zhao JF, Li BX, Zhang Q. Vitamin D improves levels of hormonal, oxidative stress and inflammatory parameters in polycystic ovary syndrome: a meta-analysis study. Ann Palliat Med. 2021;10(1):169-183.
826 Zhao LQ, Yu DY, Cheng JW. Intravenous glucocorticoids therapy in the treatment of Graves' ophthalmopathy: a systematic review and Meta-analysis. Int J Ophthalmol. 2019;12(7):1177-1186.
827 Zhao M, Ma S, Yu Y, et al. Efficacy and Safety of Angiotensin-Converting Enzyme Inhibitors in Combination with Angiotensin-Receptor Blockers in Nondiabetic Chronic Kidney Disease: A Systematic Review and Meta-Analysis. Curr Med Chem. 2021;28(38):7961-7973.
828 Zhao M, Qu H, Wang R, et al. Efficacy and safety of dual vs single renin-angiotensin-aldosterone system blockade in chronic kidney disease: An updated meta-analysis of randomized controlled trials. Medicine (Baltimore). 2021;100(35):e26544.
829 Zhao M, Wang R, Yu Y, et al. Efficacy and Safety of Angiotensin-Converting Enzyme Inhibitor in Combination with Angiotensin-Receptor Blocker in Chronic Kidney Disease Based on Dose: A Systematic Review and Meta-Analysis. Front Pharmacol. 2021;12:638611.
830 Zhao Y, Shen L, Ji HF. Alzheimer's disease and risk of hip fracture: a meta-analysis study. ScientificWorldJournal. 2012;2012:872173.
831 Zhao Y, Sun Y, Ji HF, et al. Vitamin D levels in Alzheimer's and Parkinson's diseases: a meta-analysis. Nutrition. 2013;29(6):828-832.
832 Zheng R, Gonzalez A, Yue J, et al. Efficacy and Safety of Vitamin D Supplementation in Patients With Systemic Lupus Erythematosus: A Meta-analysis of Randomized Controlled Trials. Am J Med Sci. 2019;358(2):104-114.
833 Zheng RN, You ZJ, Lin SH, et al. Efficacy of percutaneous radiofrequency ablation for the treatment of hepatocellular carcinoma. Genet Mol Res. 2015;14(4):17982-17994.
834 Zheng XG, Wang MZ, Wang F, et al. The Impact of Glucocorticoid Treatment on Hypocalcemia Following Thyroid Surgery: A Systematic Review and Meta-Analysis. Endocr Res. 2024;49(2):77-85.
835 Zhong JZ, Wei D, Pan HF, et al. Colloid solutions for fluid resuscitation in patients with sepsis: systematic review of randomized controlled trials. J Emerg Med. 2013;45(4):485-495.
836 Zhong X, Sun J, Zeng N, et al. The Effect of Sex on the Therapeutic Efficiency of Immune Checkpoint Inhibitors: A Systematic Review and Meta-Analysis Based on Randomized Controlled Trials. Cancers (Basel). 2024;16(2):382.
837 Zhou LM, Xu JY, Rao CP, et al. Effect of whey supplementation on circulating C-reactive protein: a meta-analysis of randomized controlled trials. Nutrients. 2015;7(2):1131-1143.
838 Zhou S, Hu H, Ru J. Efficacy and safety of sugammadex sodium in reversing rocuronium-induced neuromuscular blockade in children: An updated systematic review and meta-analysis. Heliyon. 2023;9(8):e18356.
839 Zhou S, Zheng F, Zhan CG. Clinical data mining reveals analgesic effects of lapatinib in cancer patients. Sci Rep. 2021 11;11(1):3528.
840 Zhou SS, Tao YH, Huang K, et al. Vitamin D and risk of preterm birth: Up-to-date meta-analysis of randomized controlled trials and observational studies [published correction appears in J Obstet Gynaecol Res. 2017;43(4):783.
841 Zhu H, Xu X, Zhang K, et al. The effect of intravenous vitamin C on clinical outcomes in patients with sepsis or septic shock: A meta-analysis of randomized controlled trials. Front Nutr. 2022;9:964484.

842 Zhu J, Chen N, Zhou M, et al. Calcium channel blockers versus other classes of drugs for hypertension. Cochrane Database Syst Rev. 2022;1(1):CD003654.

843 Zhu M, Li X. Meta-analysis of structured triglyceride versus other lipid emulsions for parenteral nutrition. Nutrition. 2013;29(6):833-840.

844 Zhu M, Zhou C, Huang B, et al. Granisetron plus dexamethasone for prevention of postoperative nausea and vomiting in patients undergoing laparoscopic surgery: A meta-analysis. J Int Med Res. 2017;45(3):904-911.

845 Zhuo Y, Yu R, Wu C, et al. The role of perioperative intravenous low-dose dexamethasone in rapid recovery after total knee arthroplasty: a meta-analysis. J Int Med Res. 2021;49(3):300060521998220.

846 Zou Y, Guo B, Yu S, et al. Effect of vitamin D supplementation on glycose homeostasis and islet function in vitamin D deficient or insufficient diabetes and prediabetes: a systematic review and meta-analysis. J Clin Biochem Nutr. 2021;69(3):229-237.

847 Zou Z, Jiang Y, Xiao M, et al. The impact of prophylactic dexamethasone on nausea and vomiting after thyroidectomy: a systematic review and meta-analysis. PLoS One. 2014;9(10):e109582.

**Methods to identifiy retacted trials within each systematic review**

One important step in this research is the method to identify retracted trials within each systematic review. The task of identifying how many retracted trials were involved in a systematic review would only be able to use forward citation searching to identify one retracted trial for a systematic review.

There were two potential methods to solve the problem. First, matching all the references of each systematic review to the included retracted trials. We have tested the Web of Science and Scopus to trying to get the references of each systematic review while finally we obversved that there were two problems of this method — 1) the references of the systematic reviews were not always correct that recorded in Web of Science and Scopus; 2) Some of the references could not be exported due to some restrictions. To this end, this method is not the best choice to accurately identify all retracted trials within each systematic review.

We then took the second method, improvised by checking the duplicates — if a systematic review was tracked by several trials via forward citation searching, it will appear the same number of times in our data collection sheet and a checking of the duplicates could find out all of the retracted trials that were included in the systematic review. This is a valid method to avoid the above two problems faced by the first method.

**Supplementary Table S6. Details of guideline searching for the 68 SRs of the related clinical practice guidelines.**

| SR_ID | Title | Citations Google (Title) | Scopus (Title) | GIN (Topic) | Search (Filter: English) | TRIP (Topic) | Search (Filter: guidelines) |
|---|---|---|---|---|---|---|---|
| 10_9 | Lin LJ, Liu J, Xu LZ, Chen H. The impact of endometrial mechanical stimulation in women with normal hysteroscopic findings undergoing IVF/ICSI: a meta-analysis. Clinical and Experimental Obstetrics & Gynecology. 2022 Jan 19;49(1):27. | 2 | 2 | 5 | 1. Infertility (5) | 45 | 1. Endometrial scratching (1)<br>2. Infertile & in vitro fertilization (0)<br>3. Infertile & Intracytoplasmic sperm injection (0)<br>4. Infertility & In vitro fertilization (0)<br>5. Infertility & In vitro fertilization (0)<br>6. Infertile (44)<br>7. Infertility (44) |
| 120_12 | Jia D, Tan H, Faramand A, Fang F. One Anastomosis Gastric Bypass Versus Roux-en-Y Gastric Bypass for Obesity: a Systematic Review and Meta-Analysis of Randomized Clinical Trials. Obes Surg. 2020 Apr;30(4):1211-1218. doi: 10.1007/s11695-019-04288-3. | 51 | 37 | 27 | 1. Obesity (27) | 21 | 1. Obesity & surgery (6)<br>1. Obesity & treatment (15) |
| 133_20 | Shi XY, Zou Z, He XY, Xu HT, Yuan HB, Liu H. Hydroxyethyl starch for cardiovascular surgery: a systematic review of randomized controlled trials. European journal of clinical pharmacology. 2011 Aug;67:767-82. | 21 | 9 | 0 | 1. Hydroxyethyl starch (0) | 0 | 1. Hydroxyethyl starch (0) |
| 13_3 | Tassi A, Parisi N, Londero AP. Misoprostol administration prior to intrauterine contraceptive device insertion: a systematic review and meta-analysis of randomised controlled trials. The European Journal of Contraception & Reproductive Health Care. 2020 Jan 2;25(1):76-86. | 13 | 7 | 0 | 1. Contraceptive device (0)<br>2. Intrauterine device (0) | 11 | 1. Contraceptive device (4)<br>2. Intrauterine device (7) |
| 144_13 | Liu A, Zheng C, Lang J, Chen W. Letrozole versus clomiphene citrate for unexplained infertility: a systematic review and meta-analysis. J Obstet Gynaecol Res. 2014 May;40(5):1205-16. doi: 10.1111/jog.12393. | 33 | 21 | 5 | 1. Infertility (5) | 44 | 1. Infertility & Letrozole (0)<br>2. Infertility & Clomiphene citrate (0)<br>3. Infertile (44)<br>4. Infertility (44) |

| | | | | | | |
|---|---|---|---|---|---|---|
| 18_2 | de Jong PG, Kaandorp S, Di Nisio M, Goddijn M, Middeldorp S. Aspirin and/or heparin for women with unexplained recurrent miscarriage with or without inherited thrombophilia. Cochrane Database Syst Rev. 2014 Jul 4;2014(7):CD004734. doi: 10.1002/14651858.CD004734.pub4. | 286 | 159 | 1 | 1. Miscarriage (1) | 4 | 1. Recurrent miscarriage (4) |
| 1_13 | Jacob M, Chappell D, Conzen P, Wilkes MM, Becker BF, Rehm M. Small-volume resuscitation with hyperoncotic albumin: a systematic review of randomized clinical trials. Crit Care. 2008;12(2):R34. doi: 10.1186/cc6812. | 115 | 67 | 5 | 1. Critically ill (5) | 9 | 1. Critically ill patients & Hyperoncotic albumin solutions (2)<br>2. Critically ill patients & albumin solutions (2)<br>3. Critically ill patients & colloid volume therapy (3)<br>4. Critically ill patients & fluid (2) |
| 201_6 | Bjelakovic G, Gluud LL, Nikolova D, Whitfield K, Wetterslev J, Simonetti RG, Bjelakovic M, Gluud C. Vitamin D supplementation for prevention of mortality in adults. Cochrane Database Syst Rev. 2014 Jan 10;(1):CD007470. doi: 10.1002/14651858.CD007470.pub3 | 1108 | 223 | 5 | 1. Vitamin D (5) | 14 | 1. Vitamin D & mortarlity (0)<br>2. Vitamin D & prevention (14) |
| 239_2 | Zhang L, Zhu J, Xu L, Zhang X, Wang H, Luo Z, Zhao Y, Yu Y, Zhang Y, Shi H, Bao H. Efficacy and safety of flurbiprofen axetil in the prevention of pain on propofol injection: a systematic review and meta-analysis. Medical Science Monitor: International Medical Journal of Experimental and Clinical Research. 2014;20:995. | 49 | 27 | 60 | 1. Propofol injection (0)<br>2. Pain (60) | 7 | 1. Pain & propofol injection (0)<br>2. Pain & flurbiprofen axetil (0)<br>3. Propofol injection & flurbiprofen axetil (0)<br>4. Pain & injection (7) |
| 258_2 | Mohamed HHB, Serag Eldien AM, Zahran A. Augmentation versus No Augmentation for Immediate Postextraction Implants. Int J Dent. 2018 Oct 16;2018:5209108. doi: 10.1155/2018/5209108. | 14 | 4 | 0 | 1. Tooth implants (0) | 0 | 1. Immediate Postextraction Implants (0)<br>2. Postextraction Implants (0)<br>3. Tooth implants (0) |

| | | | | | | | |
|---|---|---|---|---|---|---|---|
| 268_1 | Gillespie LD, Robertson MC, Gillespie WJ, Lamb SE, Gates S, Cumming RG, Rowe BH. Interventions for preventing falls in older people living in the community. Cochrane Database of Systematic Reviews 2009, Issue 2. Art. No.: CD007146. DOI: 10.1002/14651858.CD007146.pub2 | | 1368 | 28 | 1. Falls (28) | 25 | 1. Falls (25) |
| 281_1 | Plantinga NL, de Smet AM, Oostdijk EA, de Jonge E, Camus C, Krueger WA, Bergmans D, Reitsma JB, Bonten MJ. Selective digestive and oropharyngeal decontamination in medical and surgical ICU patients: individual patient data meta-analysis. Clinical Microbiology and Infection. 2018 May 1;24(5):505-13. | 105 | 69 | 2 | 1. Antibiotic resistance (2) | 0 | 1. Antibiotic resistance (0) |
| 28_28 | Hernandez AV, Phan MT, Rocco J, Pasupuleti V, Barboza JJ, Piscoya A, Roman YM, White CM. Efficacy and safety of hydroxychloroquine for hospitalized COVID-19 patients: a systematic review and meta-analysis. Journal of Clinical Medicine. 2021 Jun 5;10(11):2503. | 13 | 9 | 13 | 1. COVID-19 (13) | 44 | 1. COVID-19 & Hydroxychloroquine (4)<br>2. COVID-19 & treatment (40) |
| 28_6 | Juul S, Nielsen EE, Feinberg J, Siddiqui F, Jørgensen CK, Barot E, Holgersson J, Nielsen N, Bentzer P, Veroniki AA, Thabane L. Interventions for treatment of COVID-19: of a living systematic review with meta-analyses and trial sequential analyses (The LIVING Project). PLoS One. 2021 Mar 11;16(3):e0248132. | 57 | 38 | 13 | 1. COVID-19 (13) | 44 | 1. COVID-19 & Hydroxychloroquine (4)<br>2. COVID-19 & treatment (40) |
| 2_11 | Lepine S, Jo J, Metwally M, Cheong YC. Ovarian surgery for symptom relief in women with polycystic ovary syndrome. Cochrane Database Syst Rev. 2017 Nov 10;11(11):CD009526. | 36 | 23 | 1 | 1. Polycystic ovary syndrome (1)<br>2. PCOS (0) | 34 | 1. Polycystic ovary syndrome (22)<br>2. PCOS (12) |

| | | | | | | | |
|---|---|---|---|---|---|---|---|
| 2_4 | Franik S, Eltrop SM, Kremer JA, Kiesel L, Farquhar C. Aromatase inhibitors (letrozole) for subfertile women with polycystic ovary syndrome. Cochrane Database Syst Rev. 2018 May 24;5(5):CD010287. doi: 10.1002/14651858.CD010287.pub3. | 256 | 89 | 1 | 1. Polycystic ovary syndrome (1)<br>2. PCOS (0) | 34 | 1. Polycystic ovary syndrome (22)<br>2. PCOS (12) |
| 310_3 | Li H, Cao Y, Ma P, Ma Z, Li C, Yang W, Zhou L. Novel visualization methods assisted transurethral resection for bladder cancer: an updated survival-based systematic review and meta-analysis. Frontiers in Oncology. 2021 Jul 13;11:644341. | 9 | 9 | 7 | 1. Bladder cancer (7) | 38 | 1. Bladder cancer (38) |
| 332_1 | Shahin Y, Barnes R, Barakat H, Chetter IC. Meta-analysis of angiotensin converting enzyme inhibitors effect on walking ability and ankle brachial pressure index in patients with intermittent claudication. Atherosclerosis. 2013 Dec 1;231(2):283-90. | 63 | 44 | 0 | 1. Intermittent claudication (0) | 2 | 1. Intermittent claudication (2) |
| 332_2 | Barrons RW, Woods JA. The roles of ACE inhibitors in lower extremity peripheral artery disease. American Journal of Therapeutics. 2016 Jan 1;23(1):e7-15. | 5 | 4 | 4 | 1. Lower extremity peripheral artery disease (0)<br>2. Peripheral artery disease (4) | 45 | 1. Lower extremity peripheral artery disease (6)<br>2. Peripheral artery disease (39) |
| 371_1 | O'Meara S, Al-Kurdi D, Ologun Y, Ovington LG, Martyn-St James M, Richardson R. Antibiotics and antiseptics for venous leg ulcers. Cochrane Database of Systematic Reviews. 2014(1).Doi: 10.1002/14651858.CD003557.pub5. | 692 | 124 | 2 | 1. Venous leg ulcers (2) | 20 | 1. Venous leg ulcers (20) |
| 391_22 | Turrini G, Purgato M, Acarturk C, Anttila M, Au T, Ballette F, Bird M, Carswell K, Churchill R, Cuijpers P, Hall J. Efficacy and acceptability of psychosocial interventions in asylum seekers and refugees: systematic review and meta-analysis. Epidemiology and psychiatric sciences. 2019 Aug;28(4):376-88. | 225 | 150 | 0 | 1. Asylum seeker (0)<br>2. Refugee (0) | 13 | 1. Asylum seeker (4)<br>2. Refugee (9) |

| | | | | | | | |
|---|---|---|---|---|---|---|---|
| 43_3 | Bala MM, Paszek E, Lesniak W, Wloch-Kopec D, Jasinska K, Undas A. Antiplatelet and anticoagulant agents for primary prevention of thrombosis in individuals with antiphospholipid antibodies. Cochrane Database Syst Rev. 2018 Jul 13;7(7):CD012534. doi: 10.1002/14651858.CD012534.pub2. | 55 | 4 | 42 | 1. Thrombosis (42)<br>2. Antiphospholipid antibodies (0) | 73 | 1. Thrombosis (73) |
| 459_1 | Carlisle JB, Stevenson CA. Drugs for preventing postoperative nausea and vomiting. Cochrane Database Syst Rev. 2006 Jul 19;2006(3):CD004125. doi: 10.1002/14651858.CD004125.pub2. | 491 | 184 | 0 | 1. Postoperative nausea and vomiting (0)<br>2. PONV (0) | 3 | 1. Postoperative nausea and vomiting (3) |
| 46_16 | Izcovich A, Peiris S, Ragusa M, Tortosa F, Rada G, Aldighieri S, Reveiz L. Bias as a source of inconsistency in ivermectin trials for COVID-19: A systematic review. Ivermectin's suggested benefits are mainly based on potentially biased results. Journal of Clinical Epidemiology. 2022 Apr 1;144:43-55. | 31 | 20 | 13 | 1. COVID-19 (13) | 40 | 1. COVID-19 & Ivermectin (0)<br>2. COVID-19 & treatment (40) |
| 515_17 | Yu C, Hequn C, Jinbo C, Feng Z, Xiongbing Z, Jian D. Gemcitabine/cisplatin versus methotrexate/vinblastine/doxorubicin/cisplatin for muscle-invasive bladder cancer: A systematic review and meta-analysis. J Cancer Res Ther. 2018 Oct-Dec;14(6):1260-1265. doi: 10.4103/0973-1482.188434. | 22 | 11 | 7 | 1. Bladder cancer (7) | 38 | 1. Bladder cancer (38) |
| 58_2 | Sathianathen NJ, Hwang EC, Mian R, Bodie JA, Soubra A, Lyon JA, Sultan S, Dahm P. Selective serotonin re-uptake inhibitors for premature ejaculation in adult men. Cochrane Database Syst Rev. 2021 Mar 21;3(3):CD012799. doi: 10.1002/14651858.CD012799.pub2. | 21 | 12 | 0 | 1. Premature ejaculation (0) | 2 | 1. Premature ejaculation (2) |

| | | | | | | | |
|---|---|---|---|---|---|---|---|
| 58_3 | Cooper K, James MS, Kaltenthaler E, Dickinson K, Cantrell A. Interventions to treat premature ejaculation: a systematic review short report. Health Technology Assessment. 2015 Mar 1;19(21). | 60 | 33 | 0 | 1. Premature ejaculation (0) | 2 | 1. Premature ejaculation (2) |
| 59_19 | Wu T, Yue X, Duan X, Luo D, Cheng Y, Tian Y, Wang K. Efficacy and safety of tramadol for premature ejaculation: a systematic review and meta-analysis. Urology. 2012 Sep;80(3):618-24. doi: 10.1016/j.urology.2012.05.035. | 63 | 33 | 0 | 1. Premature ejaculation (0) | 2 | 1. Premature ejaculation (2) |
| 5_21 | Zhao LQ, Yu DY, Cheng JW. Intravenous glucocorticoids therapy in the treatment of Graves' ophthalmopathy: a systematic review and Meta-analysis. International Journal of Ophthalmology. 2019;12(7):1177. | 22 | 15 | 0 | 1. Graves ophthalmopathy (0) | 0 | 1. Graves' ophthalmopathy (0) |
| 641_2 | Shi C, Dumville JC, Cullum N, Connaughton E, Norman G. Compression bandages or stockings versus no compression for treating venous leg ulcers. Cochrane Database Syst Rev. 2021 Jul 26;7(7):CD013397. doi: 10.1002/14651858.CD013397.pub2. | 128 | 47 | 2 | 1. Venous leg ulcers (2) | 20 | 1. Venous leg ulcers (20) |
| 64_1 | Kongnyuy EJ, Wiysonge CS. Interventions to reduce haemorrhage during myomectomy for fibroids. Cochrane Database Syst Rev. 2014 Aug 15;2014(8):CD005355. doi: 10.1002/14651858.CD005355.pub5. | 434 | 95 | 0 | 1. Fibroids (0) | 7 | 1. Fibroids & myomectomy & haemorrhage (0)<br>2. Fibroids & myomectomy (0)<br>3. Fibroids & haemorrhage (0)<br>4. Fibroids myomectomy (0)<br>5. Fibroids (7)<br>6. Myomectomy & haemorrhage (0)<br>7. Myomectomy (0) |
| 652_22 | Howe TE, Shea B, Dawson LJ, Downie F, Murray A, Ross C, Harbour RT, Caldwell LM, Creed G. Exercise for preventing and treating osteoporosis in postmenopausal women. Cochrane Database of Systematic Reviews 2011, Issue 7. Art. No.: CD000333. DOI: 10.1002/14651858.CD000333.pub2. | 1093 | 324 | 12 | 1. Osteoporosis (12) | 4 | 1. Osteoporosis & Postmenopausal women (4) |

| | | | | | | | |
|---|---|---|---|---|---|---|---|
| 67_4 | de Vaan MDT, ten Eikelder MLG, Jozwiak M, Palmer KR, Davies-Tuck M, Bloemenkamp KWM, Mol BWJ, Boulvain M. Mechanical methods for induction of labour. Cochrane Database of Systematic Reviews 2019, Issue 10. Art. No.: CD001233. DOI: 10.1002/14651858.CD001233.pub3. | 207 | 49 | 0 | 1. Induction of labour (0) | 22 | 1. Induction of labour (22) |
| 711_10 | Deane KH, Jimoh OF, Biswas P, O'Brien A, Hanson S, Abdelhamid AS, Fox C, Hooper L. Omega-3 and polyunsaturated fat for prevention of depression and anxiety symptoms: systematic review and meta-analysis of randomised trials. The British Journal of Psychiatry. 2021 Mar;218(3):135-42. | 99 | 65 | 41 | 1. Depression (31)<br>2. Anxiety (10) | 14 | 1. Depression & prevention (3)<br>2. Depression & Anxiety (11) |
| 727_3 | Haas DM, Hathaway TJ, Ramsey PS. Progestogen for preventing miscarriage in women with recurrent miscarriage of unclear etiology. Cochrane Database Syst Rev. 2019 Nov 20;2019(11):CD003511. doi: 10.1002/14651858.CD003511.pub5. | 667 | 54 | 1 | 1. Progesterone (1) | 0 | 1. Recurrent miscarriage & progestogen (0)<br>1. Recurrent miscarriage & prevention (0) |
| 772_15 | Cheuk DKL, Yeung J, Chung KF,Wong V. Acupuncture for insomnia. Cochrane Database of Systematic Reviews 2007, Issue 3. Art. No.: CD005472. DOI: 10.1002/14651858.CD005472.pub2 | | 121 | 4 | 1. Insomnia (4) | 24 | 1. Insomnia (24) |
| 777_5 | Godoi A, Reis Marques I, Padrão EMH, Mahesh A, Hespanhol LC, Riceto Loyola Júnior JE, de Souza IAF, Moreira VCS, Silva CH, Miyawaki IA, Oommen C, Gomes C, Silva AC, Advani K, de Sa JR. Glucose control and psychosocial outcomes with use of automated insulin delivery for 12 to 96 weeks in type 1 diabetes: a meta-analysis of randomised controlled trials. Diabetol Metab Syndr. 2023 Sep 28;15(1):190. doi: 10.1186/s13098-023-01144-4. | 16 | 14 | 24 | 1. Type 1 diabetes (24) | 10 | 1. Type 1 diabetes & Insulin (10) |

| | | | | | | | |
|---|---|---|---|---|---|---|---|
| 78_2 | Dodd JM, Grivell RM, OBrien CM, Dowswell T, Deussen AR. Prenatal administration of progestogens for preventing spontaneous preterm birth in women with a multiple pregnancy. Cochrane Database Syst Rev. 2017 Oct 31;10(10):CD012024. doi: 10.1002/14651858.CD012024.pub2 | 138 | 32 | 2 | 1. Progesterone (1)<br>2. Multiple pregnancy (1) | 2 | 1. Progestogen & preterm birth (2) |
| 912_4 | Sharpe A, Morley LC, Tang T, Norman RJ, Balen AH. Metformin for ovulation induction (excluding gonadotrophins) in women with polycystic ovary syndrome. Cochrane Database Syst Rev. 2019 Dec 17;12(12):CD013505. doi: 10.1002/14651858.CD013505. | 144 | 91 | 1 | 1. Polycystic ovary syndrome (1) | 34 | 1. Polycystic ovary syndrome (22)<br>2. PCOS (12) |
| 912_5 | Moll E, van der Veen F, van Wely M. The role of metformin in polycystic ovary syndrome: a systematic review. Hum Reprod Update. 2007 Nov-Dec;13(6):527-37. | 294 | 157 | 1 | 1. Polycystic ovary syndrome (1) | 34 | 1. Polycystic ovary syndrome (22)<br>2. PCOS (12) |
| 930_15 | Gao L, Yang L, Lv X, Bu S, Wan F, Qian S, Wei Q, Han P, Fan T. A systematic review and meta-analysis of comparative studies on the efficacy of extended pelvic lymph node dissection in patients with clinically localized prostatic carcinoma. J Cancer Res Clin Oncol. 2014 Feb;140(2):243-56. doi: 10.1007/s00432-013-1574-2. | 20 | 10 | 10 | 1. Localized prostatic carcinoma (1)<br>2. Local prostate cancer (9) | 19 | 1.Local prostate cancer (19) |
| 93_4 | Palacios C, Kostiuk LK, Peña-Rosas JP. Vitamin D supplementation for women during pregnancy. Cochrane Database of Systematic Reviews. 2019(7). CD008873. DOI: 10.1002/14651858.CD008873.pub4. | 435 | 159 | 5 | 1. Vitamin D (5) | 10 | 1. Pregnancy & Vitamin D (10) |
| 96_49 | Li D, Cai Z, Pan Z, Yang Y, Zhang J. The effects of vitamin and mineral supplementation on women with gestational diabetes mellitus. BMC Endocr Disord. 2021 May 24;21(1):106. doi: 10.1186/s12902-021-00712-x. | 24 | 13 | 3 | 1. Gestational diabetes mellitus (3) | 15 | 1. Gestational diabetes mellitus (15) |

| | | | | | | | |
|---|---|---|---|---|---|---|---|
| 1071_3 | Antioxidants for female subfertility. 10.1002/14651858.CD007807.pub4 | 366 | 65 | 0 | 1. Subfertility (0) | 257 | 1. Subfertility & Antioxidants (0)<br>2. Infertility & Antioxidants (0)<br>3. Antioxidant (257) |
| 1106_3 | Transvaginal needle versus laparoscopic ovarian drilling in hormonal profile and pregnancy outcomes of polycystic ovary syndrome: a systematic review and meta-analysis. 10.1016/j.jogoh.2023.102606 | 6 | 4 | 1 | 1. Polycystic ovary syndrome (1) | 2 | 1. Polycystic ovary syndrome & Transvaginal needle (1)<br>2. PCOS & Transvaginal needle (1)<br>3. Polycystic ovary syndrome & needle (0)<br>4. PCOS & needle (0) |
| 1114_3 | Luteal phase clomiphene citrate for ovulation induction in women with polycystic ovary syndrome: a systematic review and meta-analysis. 10.1080/09513590.2016.1197196 | 13 | 9 | 1 | 1. Polycystic ovary syndrome (1) | 0 | 1. Polycystic ovary syndrome & Clomiphene citrate (0)<br>2. PCOS & Clomiphene citrate (0) |
| 1129_21 | Endometrial scratching during hysteroscopy in women undergoing in vitro fertilization: a systematic review and meta-analysis. 10.3389/fsurg.2023.1225111 | 1 | 1 | 0 | 1. Vitro fertilization (0) | 8 | 1. Endometrial scratching (1)<br>2. Infertility & Women (7) |
| 1129_4 | Endometrial scratch injury for women with one or more previous failed embryo transfers: a systematic review and meta-analysis of randomized controlled trials. 10.1016/j.fertnstert.2018.04.040 | 104 | 75 | 0 | 1. Embryo transfers (0) | 8 | 1. Endometrial scratching (1)<br>2. Infertility & Women (7) |
| 1132_13 | Inositol treatment of anovulation in women with polycystic ovary syndrome: a meta-analysis of randomised trials. 10.1111/1471-0528.14754 | 167 | 88 | 1 | 1. Polycystic ovary syndrome (1) | 0 | 1. Polycystic ovary syndrome & Inositol (0)<br>2. PCOS & Inositol (0) |
| 1135_22 | The effects of dexmedetomidine on postoperative sleep in elderly patients: a systematic review and meta-analysis | 2 | NA | 1 | 1. Postoperative elderly (1) | 0 | 1. Postoperative & Dexmedetomidine (0)<br>2. Elderly & Dexmedetomidine (0)<br>3. Old & Dexmedetomidine (0) |
| 1163_5 | Effect of exercise intervention on clinical parameters in patients with non-alcoholic fatty liver disease and type 2 diabetes mellitus: a meta-analysis of randomized controlled trials. 10.1097/MEG.0000000000002662 | 3 | 3 | 1 | 1. Non-alcoholic fatty liver disease (1) | 0 | 1. Non-alcoholic fatty liver disease & diabetes (0) |

| | | | | | | | |
|---|---|---|---|---|---|---|---|
| 1178_4 | Can dexmedetomidine influence recovery profiles from general anesthesia in nasal surgery? 10.1177/0194599817733735 | 45 | 30 | 0 | 1. Nasal surgery (0) | 0 | 1. Surgery & Dexmedetomidine (0) |
| 1200_1 | Interventions for pain with intrauterine device insertion. 10.1002/14651858.CD007373.pub3. | 189 | 67 | 60 | 1. Intrauterine device (0)<br>2. Pain (60) | 0 | 1. Pain & Intrauterine device (0) |
| 1205_2 | Interventions to prevent or treat heavy menstrual bleeding or pain associated with intrauterine-device use. 10.1002/14651858.CD006034.pub3. | 9 | 5 | 1 | 1. Menstrual bleeding (1)<br>2. Menstrual pain (1) | 0 | 1. Pain & Intrauterine device (0) |
| 1207_3 | Multidisciplinary biopsychosocial rehabilitation for chronic low back pain: cochrane systematic review and meta-analysis. 10.1002/14651858.CD000963.pub3 | 826 | 246 | 1 | 1. Chronic low back pain (1) | 0 | 1. Pain & biopsychosocial (0) |
| 1235_11 | The effect of sildenafil citrate on fetal and maternal ultrasound indices in iugr-complicated pregnancies; a systematic review and meta-analysis of randomized clinical trials | 0 | 0 | 0 | 1. Intrauterine Growth Restriction (0) | 0 | 1. Intrauterine Growth Restriction & Sildenafil (0) |
| 1236_14 | The effects of probiotics or synbiotics supplementation in women with polycystic ovarian syndrome: a systematic review and meta-analysis of randomized clinical trials. 10.1007/s12602-018-9493-9 | 74 | 54 | 1 | 1. Polycystic ovary syndrome (1) | 0 | 1. Polycystic ovary syndrome & Probiotics (0)<br>2. Polycystic ovary syndrome & Synbiotics (0) |
| 1239_9 | Interventions for improving mobility after hip fracture surgery in adults. 10.1002/14651858.CD001704.pub5 | 46 | 66 | 9 | 1. Hip fracture (9) | 0 | 1. Hip fracture & Mobility (0) |
| 1242_2 | Effect of granulocyte colony-stimulating factor on clinical pregnancy outcomes of recurrent miscarriage and recurrent implantation failure cases: a meta-analysis | 0 | 0 | 0 | 1. Implantation failure (0) | 0 | 1. Granulocyte colony-stimulating & Pregnancy (0)<br>2. Granulocyte colony-stimulating & Implantation failure (0) |

| | | | | | | | |
|---|---|---|---|---|---|---|---|
| 1242_9 | The value of g-csf in women experienced at least one implantation failure: a systematic review and meta-analysis. 10.3389/fendo.2024.1370114 | 3 | 3 | 0 | 1. Implantation failure (0) | 0 | 1. Granulocyte colony-stimulating & Pregnancy (0)<br>2. Granulocyte colony-stimulating & Implantation failure (0) |
| 1286_6 | The influence of perioperative dexmedetomidine on patients undergoing cardiac surgery: a meta-analysi. 10.1371/journal.pone.0152829 | 93 | 58 | 1 | 1. Cardiac surgery (1) | 0 | 1. Surgery & Dexmedetomidine (0) |
| 146_12 | Combined metforminclomiphene in clomipheneresistant polycystic ovary syndrome A systematic review and metaanalysis of randomized controlled trials. 10.1111/aogs.12673 | 54 | 30 | 1 | 1. Polycystic ovary syndrome (1) | 1 | 1. Metforminclomiphene & Polycystic ovary syndrome (1) |
| 148_13 | Colloid solutions for fluid resuscitation in patients with sepsis Systematic review of randomized controlled trials. 10.1016/j.jemermed.2013.05.018 | 24 | 15 | 7 | 1. Sepsis (7) | 2 | 1. Sepsis & Fluid resuscitation (0)<br>2. Sepsis & Colloid solutions (2) |
| 156_1 | Clinical data mining reveals analgesic effects of lapatinib in cancer patients. Sci Rep. 2021 Feb 11;11(1):3528. Doi: 10.1038/s41598-021-82318-w. | 2 | 2 | 0 | 1. Lapatinib (0) | 0 | 1. Lapatinib (0)<br>2. Cancer & Lapatinib (0) |
| 1_1 | Association of hydroxyethyl starch administration with mortality and acute kidney injury in critically ill patients requiring volume resuscitation A systematic review and metaanalysis. 10.1001/jama.2013.430 | 804 | 538 | 5 | 1. Critically ill (5) | 0 | 1. Hydroxyethyl starch & Critically ill (0)<br>2. Hydroxyethyl starch (0) |
| 204_13 | Platelet-rich-plasmapheresis for minimising peri-operative allogeneic blood transfusion. 10.1002/14651858.cd004172.pub2 | 54 | 16 | 4 | 1. Blood transfusion (4) | 1 | 1. Allogeneic transfusion (1) |
| 308_12 | Effect of B vitamin Folate, B6, and B12 supplementation on osteoporotic fracture and bone turnover markers A metaanalysis. 10.12659/MSM.893310 | 39 | 25 | 2 | 1. Osteoporotic fracture (2) | 1 | 1. Osteoporotic fracture & Vitamin (0)<br>2. Fracture & Vitamin (1)<br>3. Fracture & Folate (0) |
| 41_11 | Clomiphene and other antioestrogens for ovulation induction in polycystic ovarian syndrome. 10.1002/14651858.CD002249.pub5 | 129 | 77 | 1 | 1. Polycystic ovary syndrome (1) | 4 | 1. Polycystic ovary syndrome & Clomiphene (0)<br>2. Polycystic ovary syndrome & antioestrogen (4) |

**Supplementary Table S7. List of exclusions for potential guideline documents (with reasons).**

| **A: Exclusion lists (N = 89) during full-text downloading** | | **Reasons for exclusion** |
|---|---|---|
| 1 | Shaulov T, Sierra S, Sylvestre C. Recurrent implantation failure in IVF: A Canadian Fertility and Andrology Society Clinical Practice Guideline. Reprod Biomed Online. 2020;41(5):819-833. doi:10.1016/j.rbmo.2020.08.007 | Duplicates |
| 2 | ESHRE Add-ons working group, Lundin K, Bentzen JG, et al. Good practice recommendations on add-ons in reproductive medicine†. Hum Reprod. 2023;38(11):2062-2104. doi:10.1093/humrep/dead184 | Duplicates |
| 3 | EDe Luca M, Piatto G, Merola G, et al. IFSO Update Position Statement on One Anastomosis Gastric Bypass (OAGB). Obes Surg. 2021;31(7):3251-3278. doi:10.1007/s11695-021-05413-x | Duplicates |
| 4 | Ghiassi S, Nimeri A, Aleassa EM, et al. American Society for Metabolic and Bariatric Surgery position statement on one-anastomosis gastric bypass. Surg Obes Relat Dis. 2024;20(4):319-335. doi:10.1016/j.soard.2023.11.003 | Duplicates |
| 5 | American College of Obstetricians and Gynecologists' Committee on Practice Bulletins—Gynecology. ACOG Practice Bulletin No. 200: Early Pregnancy Loss. Obstet Gynecol. 2018;132(5):e197-e207. doi:10.1097/AOG.0000000000002899 | Duplicates |
| 6 | Toth B, Würfel W, Bohlmann M, et al. Recurrent Miscarriage: Diagnostic and Therapeutic Procedures. Guideline of the DGGG, OEGGG and SGGG (S2k-Level, AWMF Registry Number 015/050). Geburtshilfe Frauenheilkd. 2018;78(4):364-381. doi:10.1055/a-0586-4568 | Duplicates |
| 7 | Reinhart K, Perner A, Sprung CL, et al. Consensus statement of the ESICM task force on colloid volume therapy in critically ill patients. Intensive Care Med. 2012;38(3):368-383. doi:10.1007/s00134-012-2472-9 | Duplicates |
| 8 | Yu YT, Liu J, Hu B, et al. Expert consensus on the use of human serum albumin in critically ill patients. Chin Med J (Engl). 2021;134(14):1639-1654. doi:10.1097/CM9.0000000000001661 | Duplicates |
| 9 | Pilz S, März W, Cashman KD, et al. Rationale and Plan for Vitamin D Food Fortification: A Review and Guidance Paper. Front Endocrinol (Lausanne). 2018;9:373. Published 2018 Jul 17. doi:10.3389/fendo.2018.00373 | Duplicates |
| 10 | Nuti R, Brandi ML, Checchia G, et al. Guidelines for the management of osteoporosis and fragility fractures. Intern Emerg Med. 2019;14(1):85-102. doi:10.1007/s11739-018-1874-2 | Duplicates |
| 11 | Klompas M, Branson R, Cawcutt K, et al. Strategies to prevent ventilator-associated pneumonia, ventilator-associated events, and nonventilator hospital-acquired pneumonia in acute-care hospitals: 2022 Update. Infect Control Hosp Epidemiol. 2022;43(6):687-713. doi:10.1017/ice.2022.88 | Duplicates |
| 12 | Neuzillet Y, Pradère B, Xylinas E, et al. French AFU Cancer Committee Guidelines - Update 2022-2024: Non-muscle-invasive bladder cancer (NMIBC). Prog Urol. 2022;32(15):1102-1140. doi:10.1016/j.purol.2022.08.006 | Duplicates |
| 13 | Hensley PJ, Seiler R, Herr H, et al. Bladder preservation after neoadjuvant therapy - 2021 IBCN updates part 1. Urol Oncol. 2023;41(7):307-312. doi:10.1016/j.urolonc.2023.01.001 | Duplicates |
| 14 | Jin YH, Zeng XT, Liu TZ, et al. Treatment and surveillance for non-muscle-invasive bladder cancer: a clinical practice guideline (2021 edition). Mil Med Res. 2022;9(1):44. Published 2022 Aug 17. doi:10.1186/s40779-022-00406-y | Duplicates |
| 15 | Aboyans V, Ricco JB, Bartelink MEL, et al. 2017 ESC Guidelines on the Diagnosis and Treatment of Peripheral Arterial Diseases, in collaboration with the European Society for Vascular Surgery (ESVS). Rev Esp Cardiol (Engl Ed). 2018;71(2):111. doi:10.1016/j.rec.2017.12.014 | Duplicates |
| 16 | Umemura S, Arima H, Arima S, et al. The Japanese Society of Hypertension Guidelines for the Management of Hypertension (JSH 2019). Hypertens Res. 2019;42(9):1235-1481. doi:10.1038/s41440-019-0284-9 | Duplicates |
| 17 | Lawall H, Huppert P, Espinola-Klein C, Zemmrich CS, Ruemenapf G. German guideline on the diagnosis and treatment of peripheral artery disease - a comprehensive update 2016. Vasa. 2017;46(2):79-86. doi:10.1024/0301-1526/a000603 | Duplicates |

| | | |
|---|---|---|
| 18 | Zbigniew Krasiński , Zbigniew A. Gaciong , Filip M. Szymański, et al. The position of Polish experts on conservative management in patients with artery diseases of lower limbs. Acta Angiologica. 2019;25(2):41–76. doi:10.5603/aa.2019.0007 | Duplicates |
| 19 | Visseren FLJ, Mach F, Smulders YM, et al. 2021 ESC Guidelines on cardiovascular disease prevention in clinical practice. Eur J Prev Cardiol. 2022;29(1):5-115. doi:10.1093/eurjpc/zwab154 | Duplicates |
| 20 | Kramer A, Dissemond J, Kim S, et al. Consensus on Wound Antisepsis: Update 2018. Skin Pharmacol Physiol. 2018;31(1):28- 58. doi:10.1159/000481545 | Duplicates |
| 21 | Sinha S, Sreedharan S. Management of venous leg ulcers in general practice - a practical guideline. Aust Fam Physician. 2014;43(9):594-598. | Duplicates |
| 22 | Maessen-Visch MB, de Roos KP. Dutch Venous Ulcer guideline update. Phlebology. 2014;29(1 suppl):153-156. doi:10.1177/0268355514529693 | Duplicates |
| 23 | Kelechi TJ, Johnson JJ; WOCN Society. Guideline for the management of wounds in patients with lower-extremity venous disease: an executive summary. J Wound Ostomy Continence Nurs. 2012;39(6):598-606.doi:10.1097/WON.0b013e31827179e9 | Duplicates |
| 24 | Melloul E, Hübner M, Scott M, et al. Guidelines for Perioperative Care for Liver Surgery: Enhanced Recovery After Surgery (ERAS) Society Recommendations. World J Surg. 2016;40(10):2425-2440. doi:10.1007/s00268-016-3700-1 | Duplicates |
| 25 | Mortensen K, Nilsson M, Slim K, et al. Consensus guidelines for enhanced recovery after gastrectomy: Enhanced Recovery After Surgery (ERAS®) Society recommendations. Br J Surg. 2014;101(10):1209-1229. doi:10.1002/bjs.9582 | Duplicates |
| 26 | Gan TJ, Diemunsch P, Habib AS, et al. Consensus guidelines for the management of postoperative nausea and vomiting . Anesth Analg. 2014;118(1):85-113. doi:10.1213/ANE.0000000000000002 | Duplicates |
| 27 | Lassen K, Coolsen MM, Slim K, et al. Guidelines for perioperative care for pancreaticoduodenectomy: Enhanced Recovery After Surgery (ERAS®) Society recommendations. World J Surg. 2013;37(2):240-258. doi:10.1007/s00268-012-1771-1 | Duplicates |
| 28 | Gustafsson UO, Scott MJ, Schwenk W, et al. Guidelines for perioperative care in elective colonic surgery: Enhanced Recovery After Surgery (ERAS(®)) Society recommendations. World J Surg. 2013;37(2):259-284. doi:10.1007/s00268-012-1772-0 | Duplicates |
| 29 | Lassen K, Coolsen MM, Slim K, et al. Guidelines for perioperative care for pancreaticoduodenectomy: Enhanced Recovery After Surgery (ERAS®) Society recommendations. Clin Nutr. 2012;31(6):817-830. doi:10.1016/j.clnu.2012.08.011 | Duplicates |
| 30 | Lassen K, Soop M, Nygren J, et al. Consensus review of optimal perioperative care in colorectal surgery: Enhanced Recovery After Surgery (ERAS) Group recommendations. Arch Surg. 2009;144(10):961-969. doi:10.1001/archsurg.2009.170 | Duplicates |
| 31 | Denlinger CS, Sanft T, Baker KS, et al. Survivorship, Version 2.2017, NCCN Clinical Practice Guidelines in Oncology. J Natl Compr Canc Netw. 2017;15(9):1140-1163. doi:10.6004/jnccn.2017.0146 | Duplicates |
| 32 | Avasthi A, Grover S, Sathyanarayana Rao TS. Clinical Practice Guidelines for Management of Sexual Dysfunction. Indian J Psychiatry. 2017;59(Suppl 1):S91-S115. doi:10.4103/0019-5545.196977 | Duplicates |
| 33 | Burch HB, Perros P, Bednarczuk T, et al. Management of thyroid eye disease: a Consensus Statement by the American Thyroid Association and the European Thyroid Association. Eur Thyroid J. 2022;11(6):e220189. Published 2022 Dec 8. doi:10.1530/ETJ-22-0189 | Duplicates |
| 34 | Vedantham S, Weinberg I, Desai KR, et al. Society of Interventional Radiology Position Statement on the Management of Chronic Iliofemoral Venous Obstruction with Endovascular Placement of Metallic Stents. J Vasc Interv Radiol. 2023;34(10):1643-1657.e6. doi:10.1016/j.jvir.2023.06.013 | Duplicates |
| 35 | Sanchez-Rodriguez D, Bergmann P, Body JJ, et al. The Belgian Bone Club 2020 guidelines for the management of osteoporosis in postmenopausal women. Maturitas. 2020;139:69-89. doi:10.1016/j.maturitas.2020.05.006 | Duplicates |
| 36 | Reyes BJ, Mendelson DA, Mujahid N, et al. Postacute Management of Older Adults Suffering an Osteoporotic Hip Fracture: A Consensus Statement From the International Geriatric Fracture Society. Geriatr Orthop Surg Rehabil. 2020;11:2151459320935100. Published 2020 Jul 16. doi:10.1177/2151459320935100 | Duplicates |

| | | |
|---|---|---|
| 37 | Compston J, Cooper A, Cooper C, et al. UK clinical guideline for the prevention and treatment of osteoporosis. Arch Osteoporos. 2017;12(1):43. doi:10.1007/s11657-017-0324-5 | Duplicates |
| 38 | Tarantino U, Iolascon G, Cianferotti L, et al. Clinical guidelines for the prevention and treatment of osteoporosis: summary statements and recommendations from the Italian Society for Orthopaedics and Traumatology. J Orthop Traumatol. 2017;18(Suppl 1):3-36. doi:10.1007/s10195-017-0474-7 | Duplicates |
| 39 | Hadji P, Aapro MS, Body JJ, et al. Management of Aromatase Inhibitor-Associated Bone Loss (AIBL) in postmenopausal women with hormone sensitive breast cancer: Joint position statement of the IOF, CABS, ECTS, IEG, ESCEO IMS, and SIOG. J Bone Oncol. 2017;7:1-12. Published 2017 Mar 23. doi:10.1016/j.jbo.2017.03.001 | Duplicates |
| 40 | Beck BR, Daly RM, Singh MA, Taaffe DR. Exercise and Sports Science Australia (ESSA) position statement on exercise prescription for the prevention and management of osteoporosis. J Sci Med Sport. 2017;20(5):438-445. doi:10.1016/j.jsams.2016.10.001 | Duplicates |
| 41 | Fletcher JA. Canadian Academy of Sport and Exercise Medicine position statement: osteoporosis and exercise. Clin J Sport Med. 2013;23(5):333-338. doi:10.1097/JSM.0000000000000002 | Duplicates |
| 42 | Mendoza N, Sánchez-Borrego R, Villero J, et al. 2013 Up-date of the consensus statement of the Spanish Menopause Society on postmenopausal osteoporosis. Maturitas. 2013;76(1):99-107. doi:10.1016/j.maturitas.2013.05.021 | Duplicates |
| 43 | Yeap SS, Hew FL, Lee JK, et al. The Malaysian Clinical Guidance on the management of postmenopausal osteoporosis, 2012: a summary. Int J Rheum Dis. 2013;16(1):30-40. doi:10.1111/1756-185x.12037 | Duplicates |
| 44 | Shehata H, Elfituri A, Doumouchtsis SK, et al. FIGO Good Practice Recommendations on the use of progesterone in the management of recurrent first-trimester miscarriage. Int J Gynaecol Obstet. 2023;161 Suppl 1:3-16. doi:10.1002/ijgo.14717 | Duplicates |
| 45 | Regan L, Rai R, Saravelos S, Li TC; Royal College of Obstetricians and Gynaecologists. Recurrent MiscarriageGreen-top Guideline No. 17. BJOG. 2023;130(12):e9-e39. doi:10.1111/1471-0528.17515 | Duplicates |
| 46 | Zucchini S, Tumini S, Scaramuzza AE, et al. Recommendations for recognizing, risk stratifying, treating, and managing children and adolescents with hypoglycemia. Front Endocrinol (Lausanne). 2024;15:1387537. doi:10.3389/fendo.2024.1387537 | Duplicates |
| 47 | Mei-Dan E, Jain V, Melamed N, et al. Guideline No. 428: Management of Dichorionic Twin Pregnancies. J Obstet Gynaecol Can. 2022;44(7):819-834.e1. doi:10.1016/j.jogc.2022.05.002 | Duplicates |
| 48 | Shennan A, Suff N, Leigh Simpson J, et al. FIGO good practice recommendations on progestogens for prevention of preterm delivery. Int J Gynaecol Obstet. 2021;155(1):16-18. doi:10.1002/ijgo.13852 | Duplicates |
| 49 | Metin ALTAY, Merih BAYRAM, Aydan BİRİ, et al. Guideline on preterm labor and delivery by the society of specialists in perinatology (perinatoloji uzmanları derneği-puder), Turkey. J Clin Obstet Gynecol. 2020;30(3):118-30. 10.5336/jcog.2020-78741 | Duplicates |
| 50 | Berger R, Abele H, Bahlmann F, et al. Prevention and Therapy of Preterm Birth. Guideline of the DGGG, OEGGG and SGGG (S2k Level, AWMF Registry Number 015/025, February 2019) - Part 2 with Recommendations on the Tertiary Prevention of Preterm Birth and the Management of Preterm Premature Rupture of Membranes. Geburtshilfe Frauenheilkd. 2019;79(8):813- 833. doi:10.1055/a-0903-2735 | Duplicates |
| 51 | Berger R, Abele H, Bahlmann F, et al. Prevention and Therapy of Preterm Birth. Guideline of the DGGG, OEGGG and SGGG (S2k Level, AWMF Registry Number 015/025, February 2019) - Part 1 with Recommendations on the Epidemiology, Etiology, Prediction, Primary and Secondary Prevention of Preterm Birth. Geburtshilfe Frauenheilkd. 2019;79(8):800-812. doi:10.1055/a-0903-2671 | Duplicates |
| 52 | Palomba S, Viganò P, Chamayou S, et al. Diagnosis and management of infertility: NICE-adapted guidelines from the Italian Society of Human Reproduction. Reprod Biol Endocrinol. 2024;22(1):9. Published 2024 Jan 5. doi:10.1186/s12958-023-01179-2 | Duplicates |
| 53 | Hoeger KM, Dokras A, Piltonen T. Update on PCOS: Consequences, Challenges, and Guiding Treatment. J Clin Endocrinol Metab. 2021;106(3):e1071-e1083. doi:10.1210/clinem/dgaa839 | Duplicates |

| | | |
|---|---|---|
| 54 | Practice Committee of the American Society for Reproductive Medicine. Electronic address: ASRM@asrm.org; Practice Committee of the American Society for Reproductive Medicine. Role of metformin for ovulation induction in infertile patients with polycystic ovary syndrome (PCOS): a guideline. Fertil Steril. 2017;108(3):426-441. doi:10.1016/j.fertnstert.2017.06.026 | Duplicates |
| 55 | Moghetti P, Carmina E, De Leo V, et al. How to manage the reproductive issues of PCOS: a 2015 integrated endocrinological and gynecological consensus statement of the Italian Society of Endocrinology. J Endocrinol Invest. 2015;38(9):1025-1037. doi:10.1007/s40618-015-0274-y | Duplicates |
| 56 | Legro RS, Arslanian SA, Ehrmann DA, et al. Diagnosis and treatment of polycystic ovary syndrome: an Endocrine Society clinical practice guideline [published correction appears in J Clin Endocrinol Metab. 2021 May 13;106(6):e2462. doi: 10.1210/clinem/dgab248]. J Clin Endocrinol Metab. 2013;98(12):4565-4592. doi:10.1210/jc.2013-2350 | Duplicates |
| 57 | Panidis D, Tziomalos K, Papadakis E, Kandaraki EA, Katsikis I. The guidelines issued by the European Society for Human Reproduction and Embryology and the American Society for Reproductive Medicine regarding the induction of ovulation with metformin in patients with the polycystic ovary syndrome potentially require reconsideration. Hormones (Athens). 2013;12(2):192-200. doi:10.14310/horm.2002.1403 | Duplicates |
| 58 | Teede HJ, Misso ML, Deeks AA, et al. Assessment and management of polycystic ovary syndrome: summary of an evidence-based guideline. Med J Aust. 2011;195(6):S65-S112. doi:10.5694/mja11.10915 | Duplicates |
| 59 | Lecorguillé M, Camier A, Kadawathagedara M. Weight Changes, Nutritional Intake, Food Contaminants, and Supplements in Women of Childbearing Age, including Pregnant Women: Guidelines for Interventions during the Perinatal Period from the French National College of Midwives. J Midwifery Womens Health. 2022;67 Suppl 1:S135-S148. doi:10.1111/jmwh.13423 | Duplicates |
| 60 | Magee LA, Smith GN, Bloch C, et al. Guideline No. 426: Hypertensive Disorders of Pregnancy: Diagnosis, Prediction, Prevention, and Management. J Obstet Gynaecol Can. 2022;44(5):547-571.e1. doi:10.1016/j.jogc.2022.03.002 | Duplicates |
| 61 | Greinert R, de Vries E, Erdmann F, et al. European Code against Cancer 4th Edition: Ultraviolet radiation and cancer. Cancer Epidemiol. 2015;39 Suppl 1:S75-S83. doi:10.1016/j.canep.2014.12.014 | Duplicates |
| 62 | Aboyans V, Ricco JB, Bartelink ML, et al. Editor's Choice–2017 ESC guidelines on the diagnosis and treatment of peripheral arterial diseases, in collaboration with the European Society for Vascular Surgery (ESVS). European Journal of Vascular and Endovascular Surgery. 2018 Mar 1;55(3):305-68. | Duplicates |
| 63 | Aboyans V, Ricco JB, Bartelink ML, et al. 2017 ESC guidelines on the diagnosis and treatment of peripheral arterial diseases, in collaboration with the European Society for Vascular Surgery (ESVS). Kardiologia Polska (Polish Heart Journal). 2017;75(11):1065-160. | Duplicates |
| 64 | Greber-Platzer S, Haiden N, Hauer AC, et al. Combined vitamin D and vitamin K supplements for children and adolescents: benefit or risk? Recommendations of the nutrition committee of the Austrian Society for Pediatric and Adolescent Medicine (ÖGKJ). Monatsschrift Kinderheilkunde. 2021 Jul;169:649-53. | Not written in English |
| 65 | Uebach B, Krull E, Simon ST, et al. Leitliniengerechte Versorgung von Patienten mit malignen Wunden : Die neue S3-Leitlinie für Patienten mit einer nichtheilbaren Krebserkrankung [Guideline-based Care for patients with malignant lesions : The new S3 guideline for patients with incurable cancer]. HNO. 2022;70(3):167-178. doi:10.1007/s00106-022-01145-1 | Not written in English |
| 66 | Park I, Kim JY, Lee H, et al. Draft Revision of Clinical Practice Guidelines for Varicose Veins-Treatment. Annals of Phlebology. 2020 Aug 31;18(2):29-36. | Not written in English |
| 67 | Kırmızı İ, Bayram Dİ, Tamirci M, et al. The Contribution of Community Pharmacists to the Rational Management of Drug Use in Pregnancy: Practical Recommendations. Journal of Literature Pharmacy Sciences. 2018;7(3). doi:10.5336/pharmsci.2018-62398 | Not written in English |
| 68 | Dedov, I. I., Mel'nichenko, G. A., Mokrysheva, N. G., Pigarova, E. A., et al. Draft federal clinical practice guidelines for the diagnosis, treatment, and prevention of vitamin D deficiency. Osteoporosis and Bone Diseases. 2022;24(4), 4-26. doi:10.14341/osteo12937 | Not written in English |
| 69 | Duaso E, Casas A, Formiga F, et al. Unidades de prevención de caídas y de fracturas osteoporóticas. Propuesta del Grupo de Osteoporosis, Caídas y | Not written in English |

| | | |
|---|---|---|
| | Fracturas de la Sociedad Española de Geriatría y Gerontología [Falls and osteoporotic fractures prevention units: proposed Osteoporosis, Falls and Fractures Group of the Spanish Society of Geriatrics and Gerontology] [published correction appears in Rev Esp Geriatr Gerontol. 2012 Jul;47(4):185]. Rev Esp Geriatr Gerontol. 2011;46(5):268-274. doi:10.1016/j.regg.2011.05.002 | |
| 70 | Chinese Diabetes Society. Guideline for the prevention and treatment of type 2 diabetes mellitus in China (2020 edition).Zhonghua Shi Yong Nei Ke Za Zhi.2021,41(08):668-695.doi:10.19538/j.nk2021080106. | Not written in English |
| 71 | Rüsch D, Becke K, Eberhart LH, et al. Übelkeit und Erbrechen nach Operationen in Allgemeinanästhesie - Empfehlungen zur Risikoeinschätzung, Prophylaxe und Therapie [Postoperative nausea and vomiting (PONV) - recommendations for risk assessment, prophylaxis and therapy - results of an expert panel meeting]. Anasthesiol Intensivmed Notfallmed Schmerzther. 2011;46(3):158-170. doi:10.1055/s-0031-1274927. | Not written in English |
| 72 | Huyghe E, Cuzin B, Grellet L, et al. Recommandations pour le traitement de l'éjaculation prématurée [Recommendations for the treatment of premature ejaculation]. Prog Urol. 2023;33(5):237-246. doi:10.1016/j.purol.2023.02.003 | Not written in English |
| 73 | Qiu ML, Xie Y, Wang XH, et al. Practice guideline for patients with osteoporosis. Zhonghua Nei Ke Za Zhi. 2020;59(12):953-959. doi:10.3760/cma.j.cn112138-20200904-00792 | Not written in English |
| 74 | Pérez Edo L, Alonso Ruiz A, Roig Vilaseca D, et al. Actualización 2011 del consenso Sociedad Española de Reumatología de osteoporosis [2011 Update of the consensus statement of the Spanish Society of Rheumatology on osteoporosis]. Reumatol Clin. 2011;7(6):357-379. doi:10.1016/j.reuma.2011.05.013 | Not written in English |
| 75 | Qiao J. Clinical practice guidelines for progesterone in pregnancy maintenance and luteal phase support. Chin J Reprod Contracept. 2021;41: 95-105. | Not written in English |
| 76 | Update on the French position statement about closed-loop automated insulin delivery. 10.1016/j.mmm.2024.04.002. | Not written in English |
| 77 | Pollak F, Araya V, Lanas A, et al. II Consenso de la Sociedad Chilena de Endocrinología y Diabetes sobre resistencia a la insulina [Second Consensus of the Chilean Society of Endocrinology and Diabetes about insulin resistance]. Rev Med Chil. 2015;143(5):627-636. doi:10.4067/S0034-98872015000500012 | Not written in English |
| 78 | Guevara Ríos, E., et al. "Prevention and clinical management of pre-eclampsia and eclampsia: evidence-based clinical practice guidelines from the national reference institute in maternal health of Peru." (2019): 243-249. | Not written in English |
| 79 | Bouillon R, Rosen C. The IOM–Endocrine Society controversy on recommended vitamin D targets: in support of the IOM position. InVitamin D 2018 Jan 1 (pp. 1065-1089). Academic Press. | Book Chapter |
| 80 | Fletcher J, Harding K, Richards A. Treatment strategies for wound infection. Essential Microbiology for Wound Care. 2016 Jan 7:149-64. | Book Chapter |
| 81 | Benjamin K. Weeks; Belinda Ruth Beck. Exercise and Physical Activity Recommendations for Optimizing Musculoskeletal Health in Older Adults.2019-01-01. Elsevier eBooks | Book Chapter |
| 82 | Reinhold Vieth; Michael F. Holick. The IOM—Endocrine Society Controversy on Recommended Vitamin D Targets. 2018-01-01.Elsevier eBooks | Book Chapter |
| 83 | FIMS position statement 2014: Physical activity and bone health. International SportMed Journal - Volume 15, Issue 2, pp. 113-122 - published 2014-01-01. | Book Chapter |
| 84 | Management of osteoporosis in a post-menopausal woman. MeReC Bulletin - Volume 20, Issue 1, pp. 1-6 - published 2009-01-01. | Book Chapter |
| 85 | Bernecker R, Landkammer YT, Herfert J, Wicker A. Physical activity and bone health: FIMS Position Statement 2014. International SportMed Journal. 2014 Jun 1;15(2):113-22. | No full-text |
| 86 | Faucher LD, Gibson AL, Schurr MJ. 37 MANAGEMENT OF CHRONIC WOUNDS. | No full-text |

| | | |
|---|---|---|
| 87 | Juliet Compston. Guidelines for prevention and treatment of glucocorticoid-induced osteoporosis. Bone. Doi:10.1016/j.bone.2009.07.039 | No full-text |
| 88 | OR WS, ULCER AI. Quick Reference Guide for Primary Care, for consultation & local adaptation. | No full-text |
| 89 | Warburton, D. E., Katzmarzyk, P. T., Rhodes, R. E., & Shephard, R. J. (2007). Evidence-informed physical activity guidelines for Canadian adults. Canadian journal of public health = Revue canadienne de sante publique, 98 Suppl 2, S16–S68. | No full-text |
| 90 | Alabbas A, Kirpalani A, Morgan C, Mammen C, Licht C, Phan V, Wade A, Harvey E, Zappitelli M, Clark EG, Hiremath S, Soroka SD, Wald R, Weir MA, Chanchlani R, Lemaire M. Canadian Association of Paediatric Nephrologists COVID-19 Rapid Response: Guidelines for Management of Acute Kidney Injury in Children. Can J Kidney Health Dis. 2021 Feb 5;8:2054358121990135. | Duplicates |
| 91 | Scott MJ, Aggarwal G, Aitken RJ, et al. Consensus Guidelines for Perioperative Care for Emergency Laparotomy Enhanced Recovery After Surgery (ERAS®) Society Recommendations Part 2-Emergency Laparotomy: Intra- and Postoperative Care. *World J Surg*. 2023;47(8):1850-1880. | Duplicates |
| 92 | Palevsky PM, Liu KD, Brophy PD, et al. KDOQI US commentary on the 2012 KDIGO clinical practice guideline for acute kidney injury. *Am J Kidney Dis*. 2013;61(5):649-672. | Duplicates |
| 93 | Perner A, Junttila E, Haney M, et al. Scandinavian clinical practice guideline on choice of fluid in resuscitation of critically ill patients with acute circulatory failure. *Acta Anaesthesiol Scand*. 2015;59(3):274-285. | Duplicates |
| 94 | Society for Maternal-Fetal Medicine (SMFM), Shields AD, Plante LA, Pacheco LD, Louis JM; SMFM Publications Committee. Electronic address: pubs@smfm.org. Society for Maternal-Fetal Medicine Consult Series #67: Maternal sepsis. *Am J Obstet Gynecol*. 2023;229(3):B2-B19. | Duplicates |
| 95 | Sanft T, Day AT, Goldman M, et al. NCCN Guidelines® Insights: Survivorship, Version 2.2024. J Natl Compr Canc Netw. 2024;22(10):648-658. | Duplicates |
| 96 | Llau JV, Acosta FJ, Escolar G, et al. Multidisciplinary consensus document on the management of massive haemorrhage (HEMOMAS document). *Med Intensiva*. 2015;39(8):483-504. | Duplicates |
| 97 | Gontero P, Birtle A, Capoun O, et al. European Association of Urology Guidelines on Non-muscle-invasive Bladder Cancer (TaT1 and Carcinoma In Situ)-A Summary of the 2024 Guidelines Update. *Eur Urol*. 2024;86(6):531-549. | Duplicates |
| 98 | Suker A, Li Y, Robson D, Marren A; Australasian CREI (Certificate of Reproductive Endocrinology and Infertility) Consensus Expert Panel on Trial Evidence (ACCEPT) Group. Australasian recurrent pregnancy loss clinical management guideline 2024, part II. Aust N Z J Obstet Gynaecol. 2024;64(5):445-458. | Duplicates |
| 99 | ESHRE Add-ons working group, Lundin K, Bentzen JG, et al. Good practice recommendations on add-ons in reproductive medicine†. *Hum Reprod*. 2023;38(11):2062-2104. | Duplicates |
| 100 | Mascarenhas M, Jeve Y, Polanski L, et al. Management of recurrent implantation failure: British Fertility Society policy and practice guideline. *Hum Fertil (Camb)*. 2022;25(5):813-837. | Duplicates |
| 101 | Guideline Group on Unexplained Infertility, Romualdi D, Ata B, et al. Evidence-based guideline: unexplained infertility†. *Hum Reprod*. 2023;38(10):1881-1890. | Duplicates |
| 102 | ESHRE Add-ons working group, Lundin K, Bentzen JG, et al. Good practice recommendations on add-ons in reproductive medicine†. *Hum Reprod*. 2023;38(11):2062-2104. | Duplicates |
| 103 | Shaulov T, Sierra S, Sylvestre C. Recurrent implantation failure in IVF: A Canadian Fertility and Andrology Society Clinical Practice Guideline. *Reprod Biomed Online*. 2020;41(5):819-833. | Duplicates |
| 104 | Curtis KM, Jatlaoui TC, Tepper NK, et al. U.S. Selected Practice Recommendations for Contraceptive Use, 2016. *MMWR Recomm Rep*. 2016;65(4):1-66. | Duplicates |
| 105 | FSRH Guideline (March 2023) Intrauterine contraception. *BMJ Sex Reprod Health*. 2023;49(Suppl 1):1-142. | Duplicates |

| | |
|---|---|
| 106 Castelnuovo G, Giusti EM, Manzoni GM, et al. Psychological Treatments and Psychotherapies in the Neurorehabilitation of Pain: Evidences and Recommendations from the Italian Consensus Conference on Pain in Neurorehabilitation. *Front Psychol*. 2016;7:115. | Duplicates |
| 107 Makkad B, Heinke TL, Sheriffdeen R, et al. Practice Advisory for Preoperative and Intraoperative Pain Management of Cardiac Surgical Patients: Part 2. *Anesth Analg*. 2023;137(1):26-47. | Duplicates |
| **B: Exclusion lists (N = 68) based on citation check** | |
| 1 Maessen-Visch MB, de Roos KP. Dutch Venous Ulcer guideline update. Phlebology. 2014;29(1 suppl):153-156. doi:10.1177/0268355514529693 | Not cited |
| 2 Kelechi TJ, Johnson JJ; WOCN Society. Guideline for the management of wounds in patients with lower-extremity venous disease: an executive summary. J Wound Ostomy Continence Nurs. 2012;39(6):598-606. doi:10.1097/WON.0b013e3 1827179e9 | Not cited |
| 3 El Hachem M, Zambruno G, Bourdon-Lanoy E, et al. Multicentre consensus recommendations for skin care in inherited epidermolysis bullosa. Orphanet J Rare Dis. 2014;9:76. Published 2014 May 20. doi:10.1186/1750-1172-9-76 | Not cited |
| 4 Swanson T, Haesler E, Angel D, et al. IWII wound infection in clinical practice consensus document 2016 update. Wound Practice & Research: Journal of the Australian Wound Management Association. 2016 Dec;24(4):194-8. | Not cited |
| 5 Wittens C, Davies AH, Bækgaard N, et al. Editor's Choice - Management of Chronic Venous Disease: Clinical Practice Guidelines of the European Society for Vascular Surgery (ESVS) [published correction appears in Eur J Vasc Endovasc Surg. 2020 Mar;59(3):495. doi: 10.1016/j.ejvs.2019.11.027]. Eur J Vasc Endovasc Surg. 2015;49(6):678-737. doi:10.1016/j.ejvs.2015.02.007 | Not cited |
| 6 O'Donnell TF Jr, Passman MA, Marston WA, et al. Management of venous leg ulcers: clinical practice guidelines of the Society for Vascular Surgery ® and the American Venous Forum. J Vasc Surg. 2014;60(2 Suppl):3S-59S. doi:10.1016/j.jvs.2014.04.049 | Not cited |
| 7 Goldberg MT; WOCN Wound Guidelines Task Force. From the chair, WOCN Wound Guidelines Task Force. J Wound Ostomy Continence Nurs. 2012;39(6):597. doi:10.1097/01.WON.0000423194.44492.89 | Not cited |
| 8 Rai R. Standard guidelines for management of venous leg ulcer. Indian Dermatol Online J. 2014;5(3):408-411. doi:10.4103/2229-5178.137830 | Not cited |
| 9 O'Donnell TF Jr, Passman MA. Clinical practice guidelines of the Society for Vascular Surgery (SVS) and the American Venous Forum (AVF)--Management of venous leg ulcers. Introduction. J Vasc Surg. 2014;60(2 Suppl):1S-2S. doi:10.1016/j.jvs.2014.04.058 | Not cited |
| 10 Dogra S, Sarangal R. Summary of recommendations for leg ulcers. Indian Dermatol Online J. 2014;5(3):400-407. doi:10.4103/2229-5178.137829 | Not cited |
| 11 Back DA, Scheuermann-Poley C, Willy C. Recommendations on negative pressure wound therapy with instillation and antimicrobial solutions - when, where and how to use: what does the evidence show?. Int Wound J. 2013;10 Suppl 1(Suppl 1):32-42. doi:10.1111/iwj.12183 | Not cited |
| 12 van Gent WB, Wilschut ED, Wittens C. Management of venous ulcer disease. BMJ. 2010;341:c6045. Published 2010 Nov 12. doi:10.1136/bmj.c6045 | Not cited |
| 13 Narbutt J, Bowszyc-Dmochowska M, Kapińska-Mrowiecka M, et al. Chronic venous insufficiency–pathogenesis, diagnosis and pharmacological treatment. Diagnostic and therapeutic recommendations of the Polish Dermatological Society. Part II. Dermatology Review/Przegląd Dermatologiczny. 2018 Jan 1;105(4):486-97. | Not cited |
| 14 Ito T, Kukino R, Takahara M, et al. The wound/burn guidelines - 5: Guidelines for the management of lower leg ulcers/varicose veins. J Dermatol. 2016;43(8):853-868. doi:10.1111/1346-8138.13286 | Not cited |
| 15 Gould L, Stuntz M, Giovannelli M, et al. Wound Healing Society 2015 update on guidelines for pressure ulcers. Wound Repair Regen. 2016;24(1):145-162. doi:10.1111/wrr.12396 | Not cited |
| 16 Collins L, Seraj S. Diagnosis and treatment of venous ulcers. Am Fam Physician. 2010;81(8):989-996. | Not cited |

| | | |
|---|---|---|
| 17 | Song JJ, Salcido R. Use of honey in wound care: an update. Adv Skin Wound Care. 2011 Jan;24(1):40-4; quiz 45-6. doi: 10.1097/01.ASW.0000392731.34723.06. | Not cited |
| 18 | Sibbald RG, Goodman L, Woo KY, et al. Special considerations in wound bed preparation 2011: an update©. Adv Skin Wound Care. 2011 Sep;24(9):415-36; quiz 437-8. doi: 10.1097/01.ASW.0000405216.27050.97. | Not cited |
| 19 | Messinger-Rapport BJ, Gammack JK, Thomas DR, et al. Clinical update on nursing home medicine: 2013. J Am Med Dir Assoc. 2013 Dec;14(12):860-76. doi: 10.1016/j.jamda.2013.09.015. | Not cited |
| 20 | Inoue Y, Hasegawa M, Maekawa T, et al; Wound/Burn Guidelines Committee. The wound/burn guidelines - 1: Wounds in general. J Dermatol. 2016 Apr;43(4):357-75. doi: 10.1111/1346-8138.13276. | Not cited |
| 21 | Gottrup F, Apelqvist J, Bjarnsholt T, et al. EWMA document: Antimicrobials and non-healing wounds. Evidence, controversies and suggestions. J Wound Care. 2013;22(5 Suppl):S1-89. doi: 10.12968/jowc.2013.22.Sup5.S1. | Not cited |
| 22 | Gustafsson UO, Scott MJ, Schwenk W, et al; Enhanced Recovery After Surgery (ERAS) Society, for Perioperative Care; European Society for Clinical Nutrition and Metabolism (ESPEN); International Association for Surgical Metabolism and Nutrition (IASMEN). Guidelines for perioperative care in elective colonic surgery: Enhanced Recovery After Surgery (ERAS(®)) Society recommendations. World J Surg. 2013 Feb;37(2):259-84. doi: 10.1007/s00268-012-1772-0. | Not cited |
| 23 | Gan TJ, Diemunsch P, Habib AS, et al; Society for Ambulatory Anesthesia. Consensus guidelines for the management of postoperative nausea and vomiting. Anesth Analg. 2014 Jan;118(1):85-113. doi: 10.1213/ANE.0000000000000002. Erratum in: Anesth Analg. 2014 Mar;118(3):689. Erratum in: Anesth Analg. 2015 Feb;120(2):494. | Not cited |
| 24 | Nelson G, Altman AD, Nick A, et al. Guidelines for postoperative care in gynecologic/oncology surgery: Enhanced Recovery After Surgery (ERAS®) Society recommendations--Part II. Gynecol Oncol. 2016 Feb;140(2):323-32. doi: 10.1016/j.ygyno.2015.12.019. Epub 2016 Jan 3. PMID: 26757238; PMCID: PMC6038804. | Not cited |
| 25 | Chang SS, Bochner BH, Chou R, et al. Treatment of Non-Metastatic Muscle-Invasive Bladder Cancer: AUA/ASCO/ASTRO/SUO Guideline. J Urol. 2017 Sep;198(3):552-559. doi: 10.1016/j.juro.2017.04.086. Epub 2017 Apr 26. Erratum in: J Urol. 2017 Nov;198(5):1175. doi: 10.1016/j.juro.2017.09.002. PMID: 28456635; PMCID: PMC5626446. | Not cited |
| 26 | Shindel AW, Althof SE, Carrier S, et al. Disorders of Ejaculation: An AUA/SMSNA Guideline. J Urol. 2022Mar; 207(3):504-512. doi: 10.1097/JU.0000000000002392. Epub 2021 Dec 28. PMID: 34961344. | Not cited |
| 27 | Vilos GA, Allaire C, Laberge PY, Leyland N; SPECIAL CONTRIBUTORS. The management of uterine leiomyomas. J Obstet Gynaecol Can. 2015;37(2):157-178. doi:10.1016/S1701-2163(15)30338-8 | Not cited |
| 28 | Reyes BJ, Mendelson DA, Mujahid N, et al. Postacute Management of Older Adults Suffering an Osteoporotic Hip Fracture: A Consensus Statement From the International Geriatric Fracture Society. Geriatr Orthop Surg Rehabil. 2020;11:2151459320935100. Published 2020 Jul 16. doi:10.1177/2151459320935100 | Not cited |
| 29 | Brooke-Wavell K, Skelton DA, Barker KL, et al. Strong, steady and straight: UK consensus statement on physical activity and exercise for osteoporosis. Br J Sports Med. Published online May 16, 2022. doi:10.1136/bjsports-2021-104634 | Not cited |
| 30 | Grygorieva NV, Kovalenko VM, Korzh MO, et al. Guideline for diagnostic, prevention and treatment of postmenopausal osteoporosis. Pain, joints, spine. 2023 Sep 28;13(3):128-54. | Not cited |
| 31 | Shehata H, Elfituri A, Doumouchtsis SK, et al. FIGO Good Practice Recommendations on the use of progesterone in the management of recurrent first-trimester miscarriage. Int J Gynaecol Obstet. 2023;161 Suppl 1:3-16. doi:10.1002/ijgo.14717 | Not cited |
| 32 | Regan L, Rai R, Saravelos S, Li TC; Royal College of Obstetricians and Gynaecologists. Recurrent MiscarriageGreen-top Guideline No. 17. BJOG. 2023;130(12):e9-e39. doi:10.1111/1471-0528.17515 | Not cited |

| | |
|---|---|
| 33 Prine LW, MacNaughton H. Office management of early pregnancy loss. Am Fam Physician. 2011;84(1):75-82. | Not cited |
| 34 Schindler AE, Carp H, Druckmann R, et al. European Progestin Club Guidelines for prevention and treatment of threatened or recurrent (habitual) miscarriage with progestogens. Gynecol Endocrinol. 2015;31(6):447-449. doi:10.3109/09513590.2015.1017459 | Not cited |
| 35 American College of Obstetricians and Gynecologists' Committee on Practice Bulletins—Gynecology. ACOG Practice Bulletin No. 200: Early Pregnancy Loss. Obstet Gynecol. 2018;132(5):e197-e207. doi:10.1097/AOG.0000000000002899 | Not cited |
| 36 Demir SC, Gedikbaşı A, Timur H, et al. Threatened miscarriage and recurrent miscarriage: Expert opinions on progesterone therapy and treatment challenges. Turk J Obstet Gynecol. 2023;20(3):242-248. doi:10.4274/tjod.galenos.2023.66789 | Not cited |
| 37 Paul R, B. Early Pregnancy Loss: A Management Guide. Obstetrics and Gynaecology Cases - Reviews.2018. doi:10.23937/2377-9004/1410124 | Not cited |
| 38 Mei-Dan E, Jain V, Melamed N, et al. Guideline No. 428: Management of Dichorionic Twin Pregnancies. J Obstet Gynaecol Can. 2022;44(7):819-834.e1. doi:10.1016/j.jogc.2022.05.002 | Not cited |
| 39 Shennan A, Suff N, Leigh Simpson J, et al. FIGO good practice recommendations on progestogens for prevention of preterm delivery. Int J Gynaecol Obstet. 2021;155(1):16-18. doi:10.1002/ijgo.13852 | Not cited |
| 40 Metin ALTAY, Merih BAYRAM, Aydan BİRİ, et al. Guideline on preterm labor and delivery by the society of specialists in perinatology (perinatoloji uzmanları derneği-puder), Turkey. J Clin Obstet Gynecol. 2020;30(3):118-30. doi:10.5336/jcog.2020-78741 | Not cited |
| 41 Coutinho CM, Sotiriadis A, Odibo A, et al. ISUOG Practice Guidelines: role of ultrasound in the prediction of spontaneous preterm birth. Ultrasound Obstet Gynecol. 2022;60(3):435-456. doi:10.1002/uog.26020 | Not cited |
| 42 Pereira WVC, Vancea DMM, de Andrade Oliveira R, et al. 2022: Position of Brazilian Diabetes Society on exercise recommendations for people with type 1 and type 2 diabetes. Diabetol Metab Syndr. 2023;15(1):2. Published 2023 Jan 2. doi:10.1186/s13098-022-00945-3 | Not cited |
| 43 Bertoldo F, Cianferotti L, Di Monaco M, et al. Definition, Assessment, and Management of Vitamin D Inadequacy: Suggestions, Recommendations, and Warnings from the Italian Society for Osteoporosis, Mineral Metabolism and Bone Diseases (SIOMMMS). Nutrients. 2022;14(19):4148. Published 2022 Oct 6. doi:10.3390/nu14194148 | Not cited |
| 44 Guasti L, Dilaveris P, Mamas MA, et al. Digital health in older adults for the prevention and management of cardiovascular diseases and frailty. A clinical consensus statement from the ESC Council for Cardiology Practice/Taskforce on Geriatric Cardiology, the ESC Digital Health Committee and the ESC Working Group on e-Cardiology. ESC Heart Fail. 2022;9(5):2808-2822. doi:10.1002/ehf2.14022 | Not cited |
| 45 LeBoff MS, Greenspan SL, Insogna KL, et al. The clinician's guide to prevention and treatment of osteoporosis [published correction appears in Osteoporos Int. 2022 Oct;33(10):2243. doi: 10.1007/s00198-022-06479-8]. Osteoporos Int. 2022;33(10):2049-2102. doi:10.1007/s00198-021-05900-y | Not cited |
| 46 Pelliccia A, Sharma S, Gati S, et al. 2020 ESC Guidelines on sports cardiology and exercise in patients with cardiovascular disease [published correction appears in Eur Heart J. 2021 Feb 1;42(5):548-549. doi: 10.1093/eurheartj/ehaa835]. Eur Heart J. 2021;42(1):17-96. doi:10.1093/eurheartj/ehaa605 | Not cited |
| 47 Meeta M, Harinarayan CV, Marwah R, Sahay R, Kalra S, Babhulkar S. Clinical Practice Guidelines on Postmenopausal Osteoporosis: *An Executive Summary and Recommendations - Update 2019-2020. J Midlife Health. 2020;11(2):96-112. doi:10.4103/jmh.JMH_143_20 | Not cited |
| 48 Gupta A, Jayes LR, Holmes S, et al. Management of Fracture Risk in Patients with Chronic Obstructive Pulmonary Disease (COPD): Building a UK Consensus Through Healthcare Professional and Patient Engagement. Int J Chron Obstruct Pulmon Dis. 2020;15:1377-1390. Published 2020 Jun 15. doi:10.2147/COPD.S233398 | Not cited |
| 49 Pottie K, Thompson W, Davies S, et al. Deprescribing benzodiazepine receptor agonists: Evidence-based clinical practice guideline. Can Fam Physician. 2018;64(5):339-351. | Not cited |
| 50 Kim KI, Jung HK, Kim CO, et al. Evidence-based guidelines for fall prevention in Korea. Korean J Intern Med. 2017;32(1):199-210. doi:10.3904/kjim.2016.218 | Not cited |

| | | |
|---|---|---|
| 51 | Blain H, Masud T, Dargent-Molina P, et al. A comprehensive fracture prevention strategy in older adults: the European Union Geriatric Medicine Society (EUGMS) statement. Aging Clin Exp Res. 2016;28(4):797-803. doi:10.1007/s40520-016-0588-4 | Not cited |
| 52 | Day AC, Wormald R, Coronini-Cronberg S, Smith R; Royal College of Ophthalmologists Cataract Surgery Commissioning Guidance Development Group. The Royal College of Ophthalmologists' Cataract Surgery Commissioning Guidance: executive summary. Eye (Lond). 2016;30(3):498-502. doi:10.1038/eye.2015.271 | Not cited |
| 53 | Camacho PM, Petak SM, Binkley N, et al. American association of clinical endocrinologists and American college of endocrinology clinical practice guidelines for the diagnosis and treatment of postmenopausal osteoporosis - 2016. Endocr Pract. 2016;22(Suppl 4):1-42. doi:10.4158/EP161435.GL | Not cited |
| 54 | Rajavi Z, Javadi MA, Daftarian N, et al. Customized Clinical Practice Guidelines for Management of Adult Cataract in Iran. J Ophthalmic Vis Res. 2015;10(4):445-460. doi:10.4103/2008-322X.176913 | Not cited |
| 55 | Kim EJ, Arai H, Chan P, Chen LK, Hill KD, Kong B et al. Strategies on fall prevention for older people living in the community: A report from a round-table meeting in IAGG 2013. Journal of Clinical Gerontology and Geriatrics. 2015 Jun;6(2):39-44. 154. doi: 10.1016/j.jcgg.2015.02.004 | Not cited |
| 56 | Avin KG, Hanke TA, Kirk-Sanchez N, et al. Management of falls in community-dwelling older adults: clinical guidance statement from the Academy of Geriatric Physical Therapy of the American Physical Therapy Association. Phys Ther. 2015;95(6):815-834. doi:10.2522/ptj.20140415 | Not cited |
| 57 | Russo, G.I., Serefoglu, E.C. Premature Ejaculation: 2020 Update. Curr Sex Health Rep 11, 411–420 (2019). doi:10.1007/s11930-019-00232-9 | Not cited |
| 58 | Morris RK, Johnstone E, Lees C, Morton V, Smith G; Royal College of Obstetricians and Gynaecologists. Investigation and Care of a Small-for-Gestational-Age Fetus and a Growth Restricted Fetus (Green-top Guideline No. 31). BJOG. 2024;131(9):e31-e80. doi:10.1111/1471-0528.17814 | Not cited |
| 59 | Fetal Growth Restriction: ACOG Practice Bulletin, Number 227. Obstet Gynecol. 2021;137(2):e16-e28. doi:10.1097/AOG.0000000000004251 | Not cited |
| 60 | American College of Obstetricians and Gynecologists' Committee on Practice Bulletins—Obstetrics and the Society forMaternal-FetalMedicin. ACOG Practice Bulletin No. 204: Fetal Growth Restriction. Obstet Gynecol. 2019;133(2):e97-e109. doi:10.1097/AOG.0000000000003070 | Not cited |
| 61 | Saggese G, Vierucci F, Prodam F, et al. Vitamin D in pediatric age: consensus of the Italian Pediatric Society and the Italian Society of Preventive and Social Pediatrics, jointly with the Italian Federation of Pediatricians. Ital J Pediatr. 2018;44(1):51. Published 2018 May 8. doi:10.1186/s13052-018-0488-7 | Not cited |
| 62 | Cacua Sanchez MT, Vargas Abello LM, Orrego Á, et al. Use of Intralesional and Perilesional Human Recombinant Epidermal Growth Factor (hrEGF) in the Local Treatment of Venous Ulcer - Review Article - Expert Recommendation. Vasc Health Risk Manag. 2023;19:595-603. Published 2023 Sep 7. doi:10.2147/VHRM.S417447 | Not used for evidence |
| 63 | Wang M, Bolland M, Grey A. Management recommendations for osteoporosis in clinical guidelines. Clin Endocrinol (Oxf). 2016;84(5):687-692. doi:10.1111/cen.13000 | Not used for evidence |
| 64 | Crandall M, Duncan T, Mallat A, et al. Prevention of fall-related injuries in the elderly: An Eastern Association for the Surgery of Trauma practice management guideline. J Trauma Acute Care Surg. 2016;81(1):196-206. doi:10.1097/TA.0000000000001025 | Not used for evidence |
| 65 | van der Marck MA, Klok MP, Okun MS, et al. Consensus-based clinical practice recommendations for the examination and management of falls in patients with Parkinson's disease. Parkinsonism Relat Disord. 2014;20(4):360-369. doi:10.1016/j.parkreldis.2013.10.030 | Not used for evidence |
| 66 | Mak JC, Cameron ID, March LM; National Health and Medical Research Council. Evidence-based guidelines for the management of hip fractures in older persons: an update. Med J Aust. 2010;192(1):37-41. doi:10.5694/j.1326-5377.2010.tb03400.x | Not used for evidence |
| 67 | Brenin DR, Dietz JR, Baima J, et al. Pain Management in Breast Surgery: Recommendations of a Multidisciplinary Expert Panel-The American Society of Breast Surgeons. Ann Surg Oncol. 2020 Nov;27(12):4588-4602. doi: 10.1245/s10434-020-08892-x. Epub 2020 Aug 11. PMID: 32783121. | Not written in English |
| 68 | World Health Organization. (2020). WHO antenatal care recommendations for a positive pregnancy experience. Nutritional interventions update: multiple micronutrient supplements during pregnancy. World Health Organization. | Not cited |

| C: Exclusion lists (N = 79) based on full-text screen for Google & Scopus | |
|---|---|
| 1 Hoeger KM, Dokras A, Piltonen T. Update on PCOS: Consequences, Challenges, and Guiding Treatment. J Clin Endocrinol Metab. 2021 Mar 8;106(3):e1071-e1083. | Review |
| 2 Homer HA. Modern management of recurrent miscarriage. Aust N Z J Obstet Gynaecol. 2019 Feb;59(1):36-44. | Review |
| 3 Russo, G.I., Serefoglu, E.C. Premature Ejaculation: 2020 Update. Curr Sex Health Rep. *2019;* 11, 411–420. | Review |
| 4 Goodfellow L, Care A, Alfirevic Z. Controversies in the prevention of spontaneous preterm birth in asymptomatic women: an evidence summary and expert opinion. BJOG. 2021 Jan;128(2):177-194. | Expert opinion |
| 5 Gunjan K, et al. Importance of Nutrition during the Gestation Period and Infant Development: A Nutrition Guide for Mother and Infant Health. Journal of Namibian Studies, 33 (2023): 4491–4513. | Review |
| 6 Berger MM, Amrein K, Barazzoni R, Bindels L, Bretón I, Calder PC, Cappa S, Cuerda C, D'Amelio P, de Man A, Delzenne NM, Forbes A, Genton L, Gombart AF, Joly F, Laviano A, Matthys C, Phyo PP, Ravasco P, Serlie MJ, Shenkin A, Stoffel NU, Talwar D, van Zanten ARH. The science of micronutrients in clinical practice - Report on the ESPEN symposium. Clin Nutr. 2024 Jan;43(1):268-283. | No clear recommendations |
| 7 Pilz S, März W, Cashman KD, Kiely ME, et al. Rationale and Plan for Vitamin D Food Fortification: A Review and Guidance Paper. Front Endocrinol (Lausanne). 2018 Jul 17;9:373. | Review |
| 8 Cianferotti L, Bertoldo F, Bischoff-Ferrari HA, Bruyere O, Cooper C, Cutolo M, Kanis JA, Kaufman JM, Reginster JY, Rizzoli R, Brandi ML. Vitamin D supplementation in the prevention and management of major chronic diseases not related to mineral homeostasis in adults: research for evidence and a scientific statement from the European society for clinical and economic aspects of osteoporosis and osteoarthritis (ESCEO). Endocrine. 2017 May;56(2):245-261. | No clear recommendations |
| 9 Melamed ML, Chonchol M, Gutiérrez OM, et al. The Role of Vitamin D in CKD Stages 3 to 4: Report of a Scientific Workshop Sponsored by the National Kidney Foundation. *Am J Kidney Dis*. 2018;72(6):834-845. | No clear recommendations |
| 10 Li-Ru Chen, Yu-Tang Wen, Chih-Lin Kuo, Kuo-Hu Chen,Calcium and Vitamin D Supplementation on Bone Health: Current Evidence and Recommendations,International Journal of Gerontology,Volume 8, Issue 4,2014,Pages 183-188. | Review |
| 11 Fernandez MA, Griffin XL, Costa ML. Management of hip fracture. *Br Med Bull*. 2015;115(1):165-172. | Review |
| 12 Lewiecki EM, Bilezikian JP, Bonewald L, et al. Osteoporosis update: proceedings of the 2013 Santa Fe Bone Symposium. *J Clin Densitom*. 2014;17(3):330-343. | Expert opinion |
| 13 Tiedemann A, Sherrington C, Close JC, Lord SR; Exercise and Sports Science Australia. Exercise and Sports Science Australia position statement on exercise and falls prevention in older people. *J Sci Med Sport*. 2011;14(6):489-495. | No clear recommendations |
| 14 Masud T, Binkley N, Boonen S, Hannan MT; FRAX(®) Position Development Conference Members. Official Positions for FRAX® clinical regarding falls and frailty: can falls and frailty be used in FRAX®? From Joint Official Positions Development Conference of the International Society for Clinical Densitometry and International Osteoporosis Foundation on FRAX®. *J Clin Densitom*. 2011;14(3):194-204. | No clear recommendations |
| 15 R. Rizzoli. Management of the oldest old with osteoporosis. European Geriatric Medicine, Volume 1, Issue 1, February 2010, Pages 15-21. | Review |
| 16 Liu CK, Fielding RA. Exercise as an intervention for frailty. *Clin Geriatr Med*. 2011;27(1):101-110. | Review |
| 17 Johnson MA, Kimlin MG, Porter KN. Vitamin D and Injury Prevention. *American Journal of Lifestyle Medicine*. 2010;4(1):21-24. | Review |
| 18 Hensley PJ, Seiler R, Herr H, et al. Bladder preservation after neoadjuvant therapy - 2021 IBCN updates part 1. *Urol Oncol*. 2023;41(7):307-312. | Review |

| | | |
|---|---|---|
| 19 | Sinha S, Sreedharan S. Management of venous leg ulcers in general practice - a practical guideline. *Aust Fam Physician*. 2014;43(9):594-598. | Expert Opinion |
| 20 | Lim CS, Baruah M, Bahia SS. Diagnosis and management of venous leg ulcers. *BMJ*. 2018;362:k3115. | Opinion |
| 21 | Franks PJ, Barker J, Collier M, et al. Management of Patients With Venous Leg Ulcers: Challenges and Current Best Practice. *J Wound Care*. 2016;25 Suppl 6:S1-S67. | No clear recommendations |
| 22 | Clark M, Adcock L. Honey for Wound Management: A Review of Clinical Effectiveness and Guidelines. Ottawa (ON): Canadian Agency for Drugs and Technologies in Health; November 6, 2018. | Review of guidelines |
| 23 | Kovac AL. Update on the management of postoperative nausea and vomiting. *Drugs*. 2013;73(14):1525-1547. | No clear recommendations |
| 24 | Tsao CW, Aday AW, Almarzooq ZI, et al. Heart Disease and Stroke Statistics-2023 Update: A Report From the American Heart Association [published correction appears in Circulation. 2023 Feb 21;147(8):e622. | Statistics summarizing |
| 25 | Martin SS, Aday AW, Almarzooq ZI, et al. 2024 Heart Disease and Stroke Statistics: A Report of US and Global Data From the American Heart Association [published correction appears in Circulation. 2024 May 7;149(19):e1164. | Statistics summarizing |
| 26 | Kanis JA, McCloskey EV, Johansson H, et al. European guidance for the diagnosis and management of osteoporosis in postmenopausal women. *Osteoporos Int*. 2013;24(1):23-57. | Review |
| 27 | Howe TE, Skelton DA. Consensus on core outcome measures of function are needed to progress our knowledge of 'best practice' exercise components for older people. *Age Ageing*. 2011;40(5):532-533. | Opinion |
| 28 | CCAUEPK Position Statement. The Role of Kinesiologists and the Promotion of Physical Activity and Exercise in the Canadian Health Care System. | No clear recommendations |
| 29 | Elisabeth Robson. Cost and impact for different degrees of implementation of the S3-guideline on osteoporosis in Germany (Master Thesis). | Orignal research |
| 30 | Practice Committees of the American Society for Reproductive Medicine and the Society for Reproductive Endocrinology and Infertility. Diagnosis and treatment of luteal phase deficiency: a committee opinion. *Fertil Steril*. 2021;115(6):1416-1423. | Opinion |
| 31 | Palomba S, Viganò P, Chamayou S, et al. Diagnosis and management of infertility: NICE-adapted guidelines from the Italian Society of Human Reproduction. *Reprod Biol Endocrinol*. 2024;22(1):9. | Review |
| 32 | Hoeger KM, Dokras A, Piltonen T. Update on PCOS: Consequences, Challenges, and Guiding Treatment. *J Clin Endocrinol Metab*. 2021;106(3):e1071-e1083. | Review |
| 33 | Coomarasamy A, Dhillon-Smith RK, Papadopoulou A, et al. Recurrent miscarriage: evidence to accelerate action. *Lancet*. 2021;397(10285):1675-1682. | Review |
| 34 | Bahri Khomami M, Teede HJ, Joham AE, Moran LJ, Piltonen TT, Boyle JA. Clinical management of pregnancy in women with polycystic ovary syndrome: An expert opinion. *Clin Endocrinol (Oxf)*. 2022;97(2):227-236. | Opinion |
| 35 | Panidis D, Tziomalos K, Papadakis E, Kandaraki EA, Katsikis I. The guidelines issued by the European Society for Human Reproduction and Embryology and the American Society for Reproductive Medicine regarding the induction of ovulation with metformin in patients with the polycystic ovary syndrome potentially require reconsideration. *Hormones (Athens)*. 2013;12(2):192-200. | Opinion |
| 36 | Gnanapragasam V, Hori S, Johnston T, et al. Clinical management and research priorities for high-risk prostate cancer in the UK: Meeting report of a multidisciplinary panel in conjunction with the NCRI Prostate Cancer Clinical Studies Localised Subgroup. *Journal of Clinical Urology*. 2016;9(6):369-379. | No clear recommendations |
| 37 | Giustina A, Bouillon R, Binkley N, Sempos C, Adler RA, Bollerslev J, Dawson-Hughes B, Ebeling PR, Feldman D, Heijboer A, Jones G, Kovacs CS, Lazaretti-Castro M, Lips P, Marcocci C, Minisola S, Napoli N, Rizzoli R, Scragg R, White JH, Formenti AM, Bilezikian JP. Controversies in Vitamin D: A Statement From the Third International Conference. JBMR Plus. 2020 Nov 10;4(12):e10417. | A summary of key points |

| | | |
|---|---|---|
| 38 | Greinert R, de Vries E, Erdmann F, Espina C, Auvinen A, Kesminiene A, Schüz J. European Code against Cancer 4th Edition: Ultraviolet radiation and cancer. Cancer Epidemiol. 2015 Dec;39 Suppl 1:S75-83. | Review |
| 39 | Giustina A, Bilezikian JP, Adler RA, Banfi G, Bikle DD, Binkley NC, Bollerslev J, Bouillon R, Brandi ML, Casanueva FF, di Filippo L, Donini LM, Ebeling PR, Fuleihan GE, Fassio A, Frara S, Jones G, Marcocci C, Martineau AR, Minisola S, Napoli N, Procopio M, Rizzoli R, Schafer AL, Sempos CT, Ulivieri FM, Virtanen JK. Consensus Statement on Vitamin D Status Assessment and Supplementation: Whys, Whens, and Hows. Endocr Rev. 2024 Apr 27:bnae009. | Discussion |
| 40 | Cappola AR, Auchus RJ, El-Hajj Fuleihan G, Handelsman DJ, Kalyani RR, McClung M, Stuenkel CA, Thorner MO, Verbalis JG. Hormones and Aging: An Endocrine Society Scientific Statement. J Clin Endocrinol Metab. 2023 Jul 14;108(8):1835-1874. | No clear recommendations |
| 41 | Harvey NC, Biver E, Kaufman JM, Bauer J, Branco J, Brandi ML, Bruyère O, Coxam V, Cruz-Jentoft A, Czerwinski E, Dimai H, Fardellone P, Landi F, Reginster JY, Dawson-Hughes B, Kanis JA, Rizzoli R, Cooper C. The role of calcium supplementation in healthy musculoskeletal ageing : An expert consensus meeting of the European Society for Clinical and Economic Aspects of Osteoporosis, Osteoarthritis and Musculoskeletal Diseases (ESCEO) and the International Foundation for Osteoporosis (IOF). Osteoporos Int. 2017 Feb;28(2):447-462 | No clear recommendations |
| 42 | Harvey NC, Biver E, Kaufman JM, Bauer J, Branco J, Brandi ML, Bruyère O, Coxam V, Cruz-Jentoft A, Czerwinski E, Dimai H, Fardellone P, Landi F, Reginster JY, Dawson-Hughes B, Kanis JA, Rizzoli R, Cooper C. The role of calcium supplementation in healthy musculoskeletal ageing : An expert consensus meeting of the European Society for Clinical and Economic Aspects of Osteoporosis, Osteoarthritis and Musculoskeletal Diseases (ESCEO) and the International Foundation for Osteoporosis (IOF). Osteoporos Int. 2017 Feb;28(2):447-462. | Duplicate |
| 43 | Australian and New Zealand Society for Geriatric Medicine. Australian and New Zealand Society for Geriatric Medicine: Position Statement--Exercise guidelines for older adults. Australas J Ageing. 2014 Dec;33(4):287-94. | No clear recommendations |
| 44 | Body JJ, Bergmann P, Boonen S, Boutsen Y, Bruyere O, Devogelaer JP, Goemaere S, Hollevoet N, Kaufman JM, Milisen K, Rozenberg S, Reginster JY. Non-pharmacological management of osteoporosis: a consensus of the Belgian Bone Club. Osteoporos Int. 2011 Nov;22(11):2769-88. | No clear recommendations |
| 45 | Fairhall N, Langron C, Sherrington C, Lord SR, Kurrle SE, Lockwood K, Monaghan N, Aggar C, Gill L, Cameron ID. Treating frailty--a practical guide. BMC Med. 2011 Jul 6;9:83. doi: 10.1186/1741-7015-9-83. | Review |
| 46 | International Wound Infection Institute. IWII Wound Infection in Clinical Practice. https://woundinfection-institute.com/wp-content/uploads/IWII-CD-2022-web.pdf | No clear recommendations |
| 47 | Songpatanasilp T, Sritara C, Kittisomprayoonkul W, Chaiumnuay S, Nimitphong H, Charatcharoenwitthaya N, Pongchaiyakul C, Namwongphrom S, Kitumnuaypong T, Srikam W, Dajpratham P, Kuptniratsaikul V, Jaisamrarn U, Tachatraisak K, Rojanasthien S, Damrongwanich P, Wajanavisit W, Pongprapai S, Ongphiphadhanakul B, Taechakraichana N. Thai Osteoporosis Foundation (TOPF) position statements on management of osteoporosis. Osteoporos Sarcopenia. 2016 Dec;2(4):191-207. | No clear recommendations |
| 48 | Guidelines on the Management of Co-occurring Alcohol and Other Drug and Mental Health Conditions in Alcohol and Other Drug Treatment Settings. | No clear recommendations |
| 49 | Falls - Prevention of Falls and Harm from Falls among Older People: 2011-2015. New South Wales Government. | Government policy |
| 50 | Burté C, Cuzin B, Grellet L, et al. Clinical recommendations of the AIUS for the management of premature ejaculation. Sexologies, 2024, 33(2): 72-96. | No full-text |
| 51 | Toro Merlo J, Pérez Alonso M M. Consenso Anticoncepción Hormonal. Actualización 2023. Capítulo 4. Situaciones especiales en obstetricia y ginecología[J]. Revista de Obstetricia y Ginecología de Venezuela, 2024, 84: 61-83. | Not written by English |
| 52 | Vidal F, Paret L, Linet T, et al. Contraception intra-utérine. RPC Contraception CNGOF. Gynécologie Obstétrique Fertilité & Sénologie, 2018, 46(12): 806-822. | Not written by English |

| | | |
|---|---|---|
| 53 | Suker A, Li Y, Robson D, Marren A; Australasian CREI (Certificate of Reproductive Endocrinology and Infertility) Consensus Expert Panel on Trial Evidence (ACCEPT) Group. Australasian recurrent pregnancy loss clinical management guideline 2024, part II. *Aust N Z J Obstet Gynaecol*. 2024;64(5):445-458. | Duplicate |
| 54 | Ghiassi S, Nimeri A, Aleassa EM, et al. American Society for Metabolic and Bariatric Surgery position statement on one-anastomosis gastric bypass. *Surg Obes Relat Dis*. 2024;20(4):319-335. | Duplicate |
| 55 | Practice Committee of the American Society for Reproductive Medicine. Electronic address: ASRM@asrm.org; Practice Committee of the American Society for Reproductive Medicine. Role of metformin for ovulation induction in infertile patients with polycystic ovary syndrome (PCOS): a guideline. *Fertil Steril*. 2017;108(3):426-441. | Duplicate |
| 56 | Costello MF, Misso ML, Balen A, et al. Evidence summaries and recommendations from the international evidence-based guideline for the assessment and management of polycystic ovary syndrome: assessment and treatment of infertility. *Hum Reprod Open*. 2019;2019(1):hoy021. | Duplicate |
| 57 | Malik S, Verma S, Jain K, Talwar P, Dhorepatil B, Devi G, et al. Good clinical practice recommendations on management of infertility in patients from India with polycystic ovary syndrome. Fertil Sci Res 2015;2:107-32. | Duplicate |
| 58 | ESHRE Add-ons working group, Lundin K, Bentzen JG, et al. Good practice recommendations on add-ons in reproductive medicine†. *Hum Reprod*. 2023;38(11):2062-2104. | Duplicate |
| 59 | Guideline Group on Unexplained Infertility, Romualdi D, Ata B, et al. Evidence-based guideline: unexplained infertility†. Hum Reprod. 2023;38(10):1881-1890. | Duplicate |
| 60 | ESHRE Add-ons working group, Lundin K, Bentzen JG, et al. Good practice recommendations on add-ons in reproductive medicine†. *Hum Reprod*. 2023;38(11):2062-2104. | Duplicate |
| 61 | Shaulov T, Sierra S, Sylvestre C. Recurrent implantation failure in IVF: A Canadian Fertility and Andrology Society Clinical Practice Guideline. *Reprod Biomed Online*. 2020;41(5):819-833. | Duplicate |
| 62 | FSRH Guideline (March 2023) Intrauterine contraception. *BMJ Sex Reprod Health*. 2023;49(Suppl 1):1-142. | Duplicate |
| 63 | FSRH Guideline (March 2023) Intrauterine contraception. *BMJ Sex Reprod Health*. 2023;49(Suppl 1):1-142. | Duplicate |
| 64 | Manchikanti L, Kaye AM, Knezevic NN, et al. Responsible, Safe, and Effective Prescription of Opioids for Chronic Non-Cancer Pain: American Society of Interventional Pain Physicians (ASIPP) Guidelines. *Pain Physician*. 2017;20(2S):S3-S92. | Duplicate |
| 65 | Mercado MG, Smith DK, Guard EL. Acute Kidney Injury: Diagnosis and Management. *Am Fam Physician*. 2019;100(11):687-694. | Review |
| 66 | Perner A, Cecconi M, Cronhjort M, et al. Expert statement for the management of hypovolemia in sepsis. *Intensive Care Med*. 2018;44(6):791-798. | Review |
| 67 | Argenziano G, Ardigò M, Micali G, et al. Review - Expert Opinion on Antibiotics and Antibiotic Resistance in Dermatology. *Dermatol Pract Concept*. 2024;14(4):e2024282. | Review |
| 68 | Abu-Musa A, Haahr T, Humaidan P. Novel Physiology and Definition of Poor Ovarian Response; Clinical Recommendations. *Int J Mol Sci*. 2020;21(6):2110. | Review |
| 69 | Qureshi R N. Evidence-based recommendations for clinical practice (future directions: Research and practice)[M]//Polycystic Ovary Syndrome. Elsevier, 2024: 181-184. | Book Chapter |
| 70 | Baker CC, Creinin MD. Long-Acting Reversible Contraception. *Obstet Gynecol*. 2022;140(5):883-897. | Review |
| 71 | Hoopes AJ, Simmons KB, Godfrey EM, Sucato GS. 2016 Updates to US Medical Eligibility Criteria for Contraceptive Use and Selected Practice Recommendations for Contraceptive Use: Highlights for Adolescent Patients. *J Pediatr Adolesc Gynecol*. 2017;30(2):149-155. | Discussion paper |

| | | |
|---|---|---|
| 72 | Manchikanti L, Hirsch JA. An update on the management of chronic lumbar discogenic pain. *Pain Manag*. 2015;5(5):373-386. | Review |
| 73 | Müller D, Keller B, Ferrante A N, et al. Therapy for Musculoskeletal Pain. International Journal of General Practice Nursing, 2023, 1(1): 22-35. | Discussion paper |
| 74 | Auckburally, Adam, Ilaria Petruccione, and Sarah Voss. Providing fluid therapy to equine colic patients. Part 1. In Practice 41.9 (2019): 445-454. | Discussion paper |
| 75 | Peng Z, Yu K, Ostermann M, et al. Pragmatic studies for acute kidney injury: Consensus report of the Acute Disease Quality Initiative (ADQI) 19 Workgroup. *J Crit Care*. 2018;44:337-344. | Not CPGs |
| 76 | Buckett W, Sierra S. The management of unexplained infertility: an evidence-based guideline from the Canadian Fertility and Andrology Society. *Reprod Biomed Online*. 2019;39(4):633-640. | Duplicate |
| 77 | Regan L, Rai R, Saravelos S, Li TC; Royal College of Obstetricians and Gynaecologists. Recurrent MiscarriageGreen-top Guideline No. 17. *BJOG*. 2023;130(12):e9-e39. | Duplicate |
| 78 | Suker A, Li Y, Robson D, Marren A; Australasian CREI (Certificate of Reproductive Endocrinology and Infertility) Consensus Expert Panel on Trial Evidence (ACCEPT) Group. Australasian recurrent pregnancy loss clinical management guideline 2024, part II. *Aust N Z J Obstet Gynaecol*. 2024;64(5):445-458. | Duplicate |
| 79 | Shindel AW, Althof SE, Carrier S, Chou R, McMahon CG, Mulhall JP, Paduch DA, Pastuszak AW, Rowland D, Tapscott AH, Sharlip ID. Disorders of Ejaculation: An AUA/SMSNA Guideline. J Urol. 2022 Mar;207(3):504-512. | Duplicate |
| 80 | Buckett W, Sierra S. The management of unexplained infertility: an evidence-based guideline from the Canadian Fertility and Andrology Society. Reprod Biomed Online. 2019 Oct;39(4):633-640. | Duplicate |

**Data extraction and replication of meta-analyses**

For the metadata of each ‘contaminated’ meta-analyses, we recruited student volunteers for the data extraction based on pre-designed data extraction forms in Excel (Microsoft, version 2016) in terms of the data type. All recruited student volunteers were trained for two rounds, and only those who achieved an accuracy of 90% and above in both rounds of training were allowed to participate in the formal data extraction. After the training, 60 student volunteers met the accuracy criteria, with 27 in group one and 33 in group two, who extracted the metadata independently. The following data type were considered to design the data extraction forms: i) binary outcomes composed of 2 by 2 table data, which refers to *Binary-Type 1*, ii) binary outcomes composed of effect sizes and confidence intervals, which refers to *Binary-Type 2*, iii) binary outcomes composed of effect sizes and standard errors, which refers to *Binary-Type 3*, iv) continuous outcomes composed of 2 by 3 table data, which refers to *Continuous-Type 1*, v) continuous outcomes composed of effect sizes and confidence intervals, which refers to *Continuous-Type 2,* and vi) continuous outcomes composed of effect sizes and standard errors, which refers to *Continuous-Type 3*. For Cochrane reviews, the data file was downloaded automatically via the .rm5 files.

Replication of the meta-analyses that incorporated retracted trials was conducted by three methodologists (CX, SQF, and YT) of evidence synthesis. This was done in two steps. We first used the same data of the original meta-analyses with the same methods and effect estimate to replicate the results, and when we were able to obtain the same results as the original meta-analysis, we excluded retracted trials and re-did the analysis under the same settings, manually or through semi-automated programming steps that we wrote. For the six pre-defined data type, the one of ‘*Binary-Type 3*’ did not occurs in any of the meta-analyses, and therefore, the rest five were applied to formulate the data extraction. The *Binary-Type 1* data was replicated manually, because of the methods used for odds ratio, risk ratio were abuntant and differs a lot, for example, the classical odds ratio, the peto odds ratio. The Comprehensive meta-analysis software was used for the replication of this type of data. For the other four, the Stata 16.0/SE was used for the replication by self-wroten programs. For Cochrane revews, the replication was done by RevMan 5.4. For those meta-analyses with the original results cannot be replicated in any of the above situations, the lead author use Stata 16.0/SE and applies all existing methods for the relavant data type to re-check whether the methods reported in the original systematic reviews were incorrect. The programs for the replication could be obtained upon request.

**Number of studies and the extent of impact on the results**

Figures S2 to S4 present the relationship between the number of trials and the proportion that changed direction, significance, or magnitude. We noticed that beyond 20 included studies, there was not much change for either direction (1.5%, 7/455), significance (5.9%, 27/455) or magnitude (2.6%, 12/455), implying that the impact of retracted trials was more evident when the number of studies in a meta-analysis was below 20. Therefore, we took the reference odds for this analysis to be the meta-analyses with 20 or more studies.

Our regression analysis suggested that, compared to meta-analyses with 20 or more studies, for a change in the direction of effect, the OR for 15 studies was 1.54 (95%CI: 0.99 to 2.40, P = 0.06), for 10 studies was 2.63 (95%CI: 1.29 to 5.38, P < 0.01), for 5 studies was 5.45 (95%CI: 2.66 to 11.16, P < 0.01), and for 2 studies was 9.18 (95%CI: 4.59 to 18.33, P < 0.01), Figure S2. For change in the significance, the OR for 15 studies was 0.92 (95%CI: 0.72 to 1.18, P = 0.51), for 10 studies was 1.17 (95%CI: 0.79 to 1.72, P = 0.43), for 5 studies was 2.72 (95%CI: 1.88 to 3.95, P < 0.01), and for 2 studies was 5.89 (95%CI: 4.08 to 8.52, P < 0.01), Figure S3. For the magnitude of the effects that changed 50% or more, the OR for 15 studies was 1.13 (95%CI: 0.80 to 1.59, P = 0.49), for 10 studies was 1.92 (95%CI: 1.10 to 3.34, P = 0.02), for 5 studies was 7.27 (95%CI: 4.16 to 12.69, P < 0.01), and for 2 studies was 22.73 (95%CI: 13.09 to 39.48, P < 0.01), Figure S4. The non-linearity tests (Wald test) suggested that there was a non-linear relationship between the number of studies and the likelihood of the change in the direction of the effects (P < 0.01), significance of the p-value (P < 0.01), and magnitude of the effects (P < 0.01). When stratifying by the proportion of retracted trials within each meta-analysis, similar trends were observed (supplementary Figure S5 to S7).

**Supplementary Figure S1.** Number of retractions of trials by year and the year of publication of the trials.

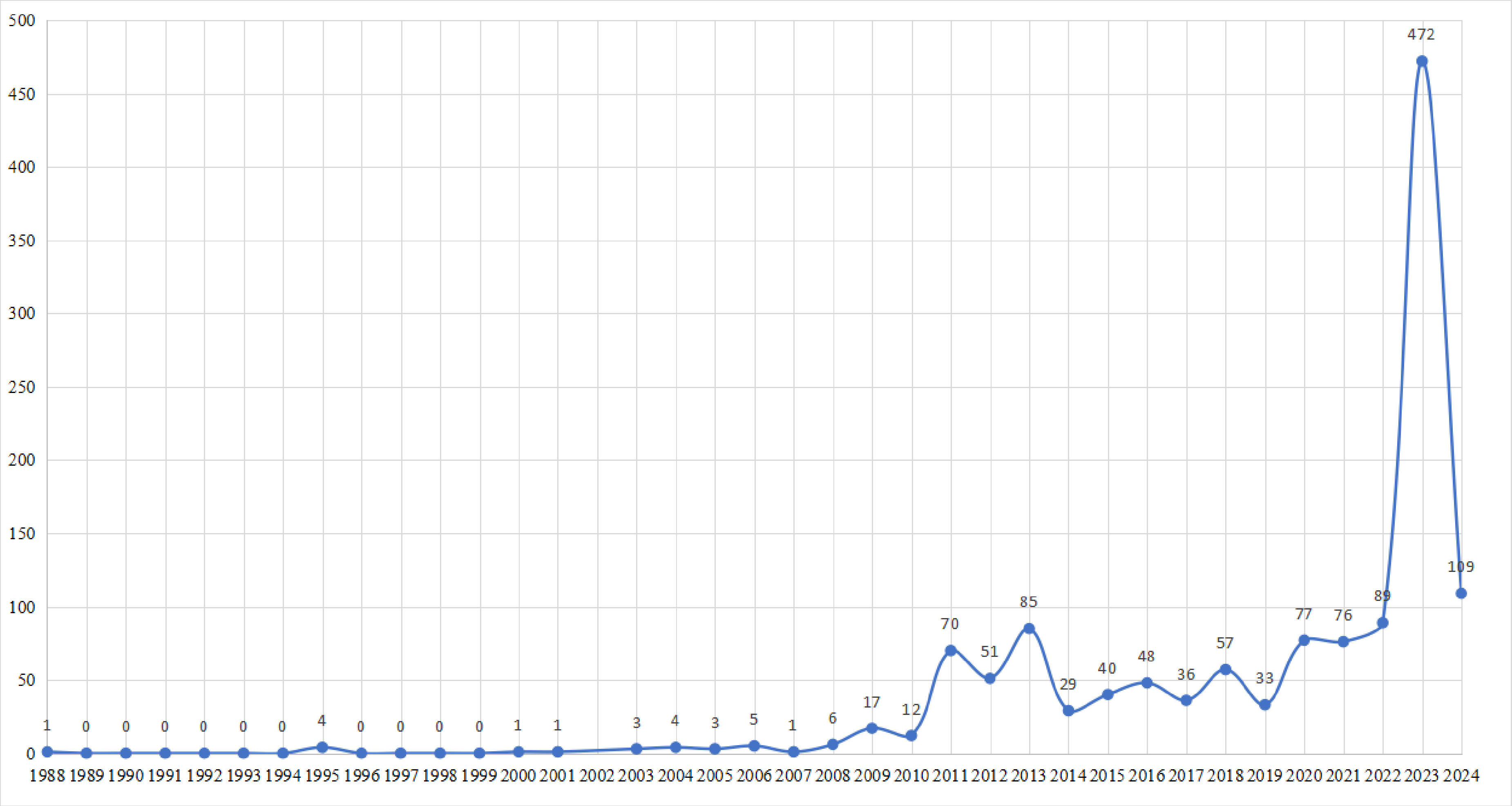

**Figure S2.** Number of included studies and the likelihood of meta-analyses that had the direction of the effects being changed.

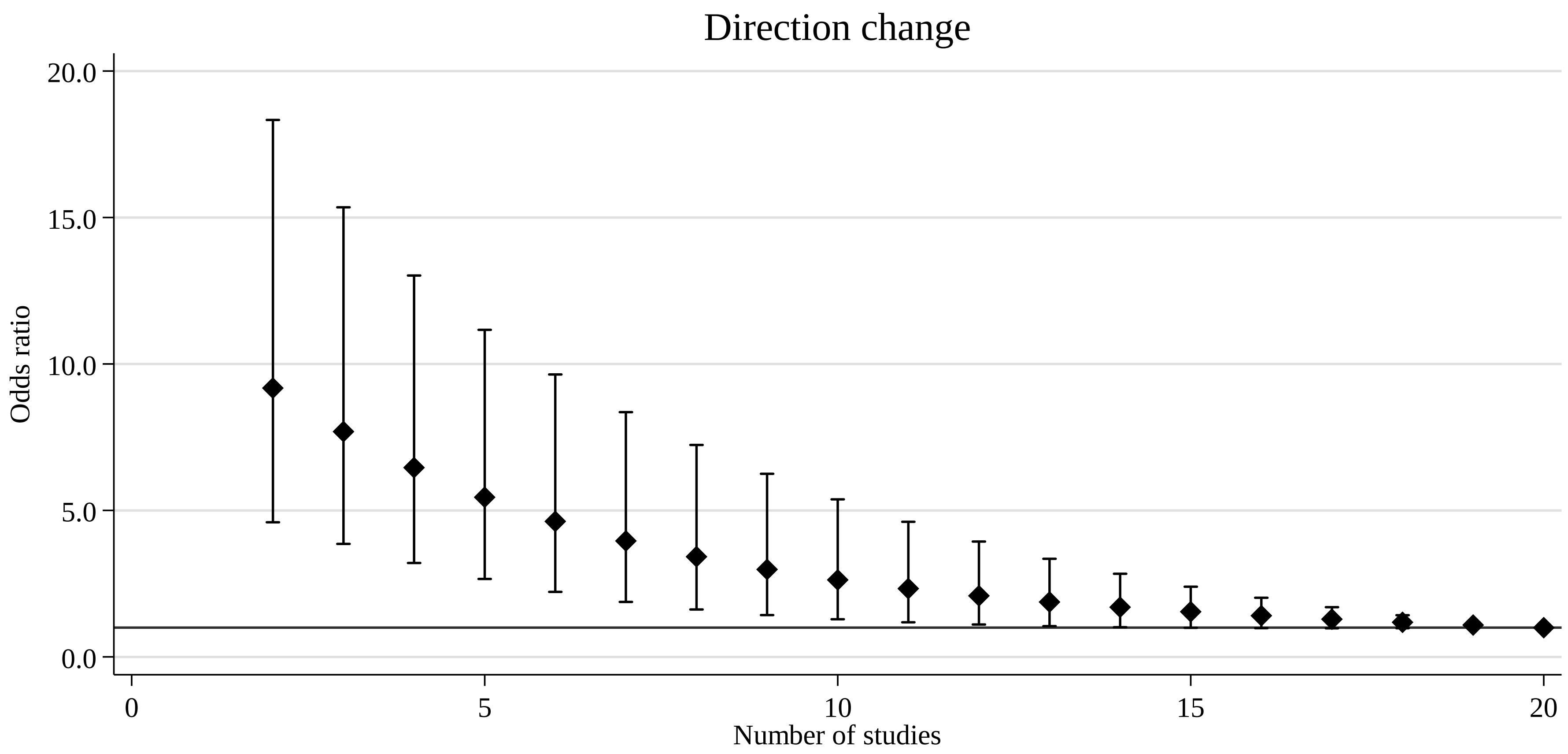

**Figure S3.** Number of included studies and the likelihood of meta-analyses that had the significance of p-value being changed.

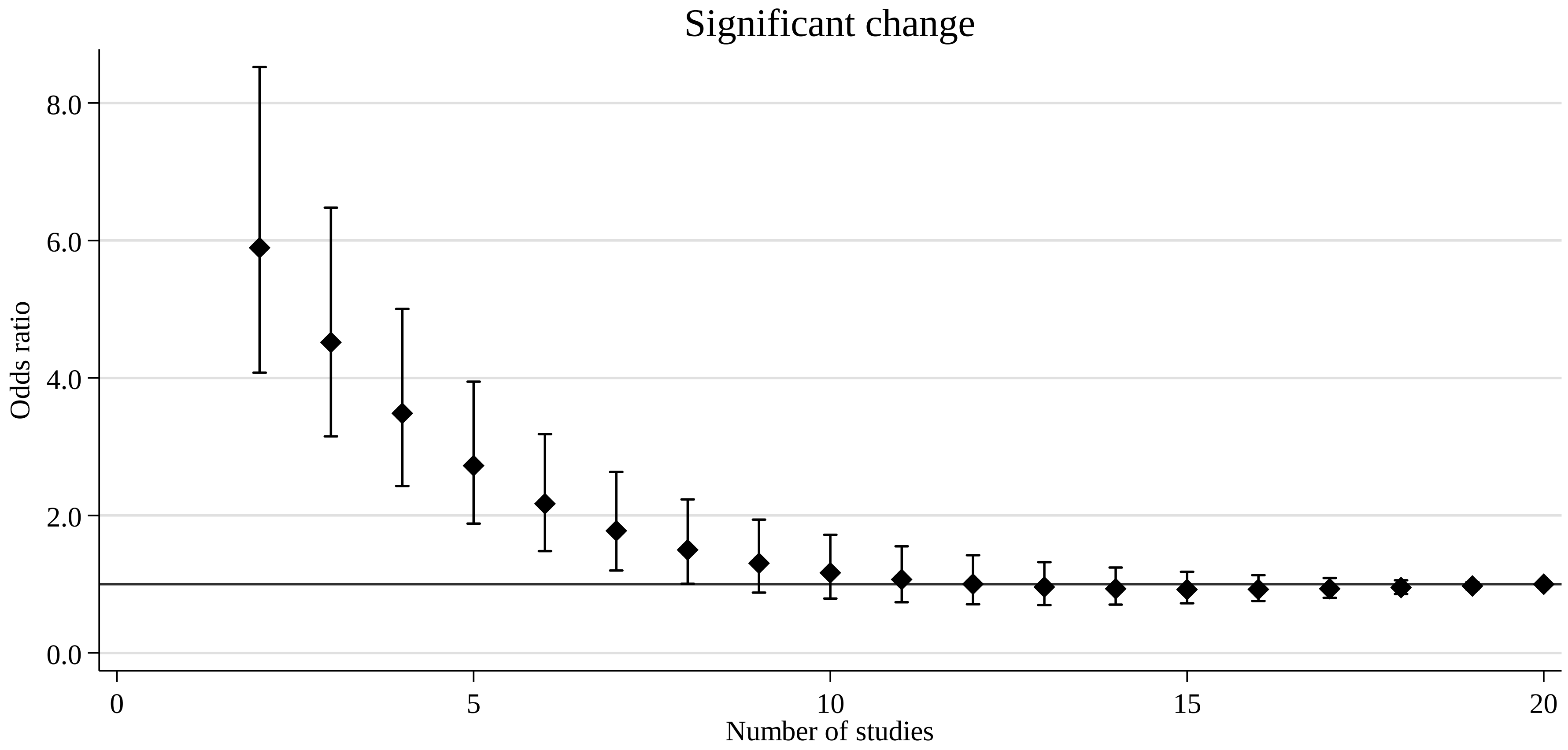

**Figure S4.** Number of included studies and the likelihood of meta-analyses that had the magnitude being changed by 50% and above.

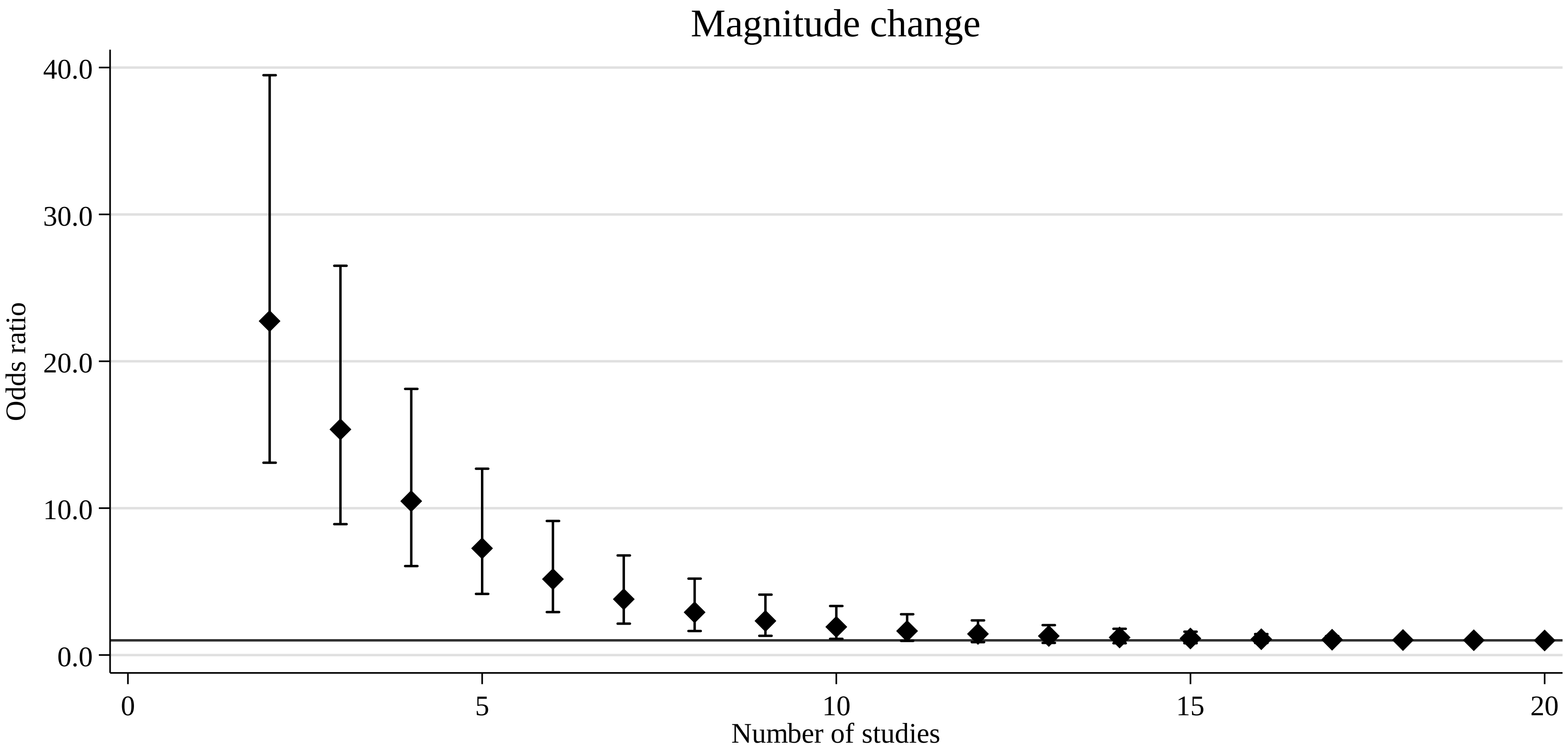

**Figure S5.** Number of included studies and the likelihood of meta-analyses that had the direction of the effects being changed, after adjusting the proportion of retractions by stratification.

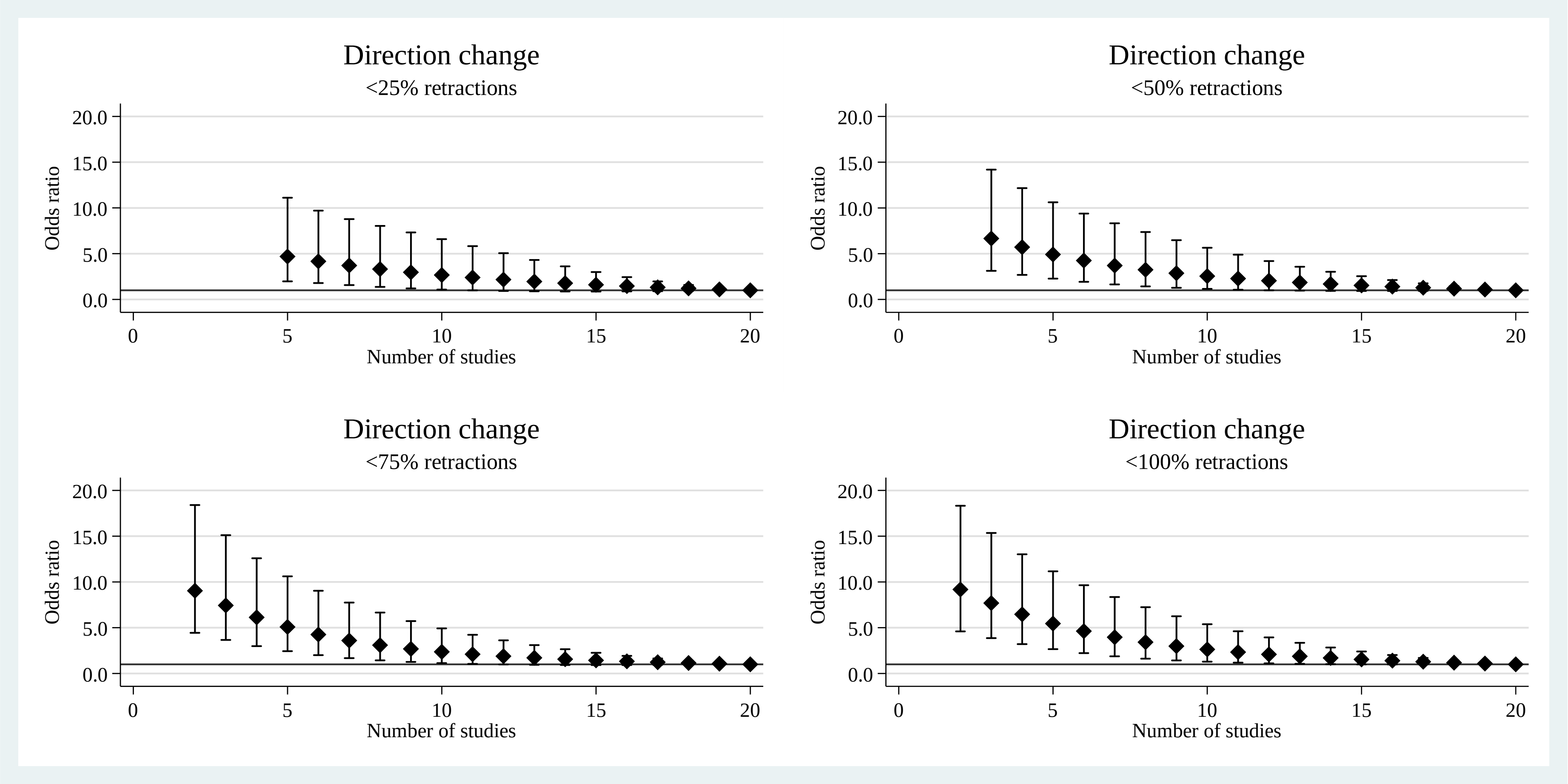

**Figure S6.** Number of included studies and the likelihood of meta-analyses that had the significance of *p*-value being changed, after adjusting the proportion of retractions by stratification.

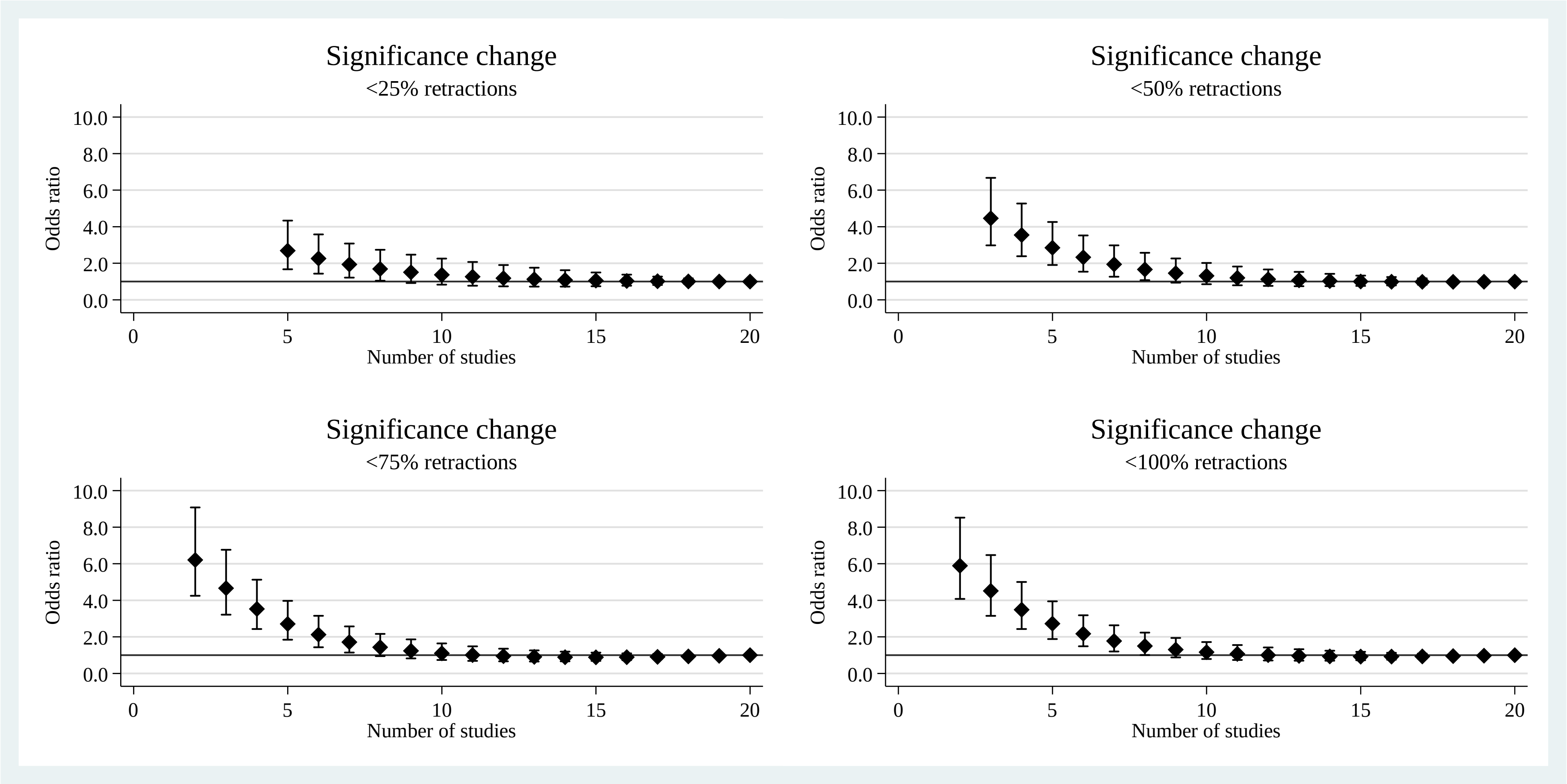

**Figure S7.** Number of included studies and the likelihood of meta-analyses that had the magnitude being changed by more than 50%, after adjusting the proportion of retractions by stratification.

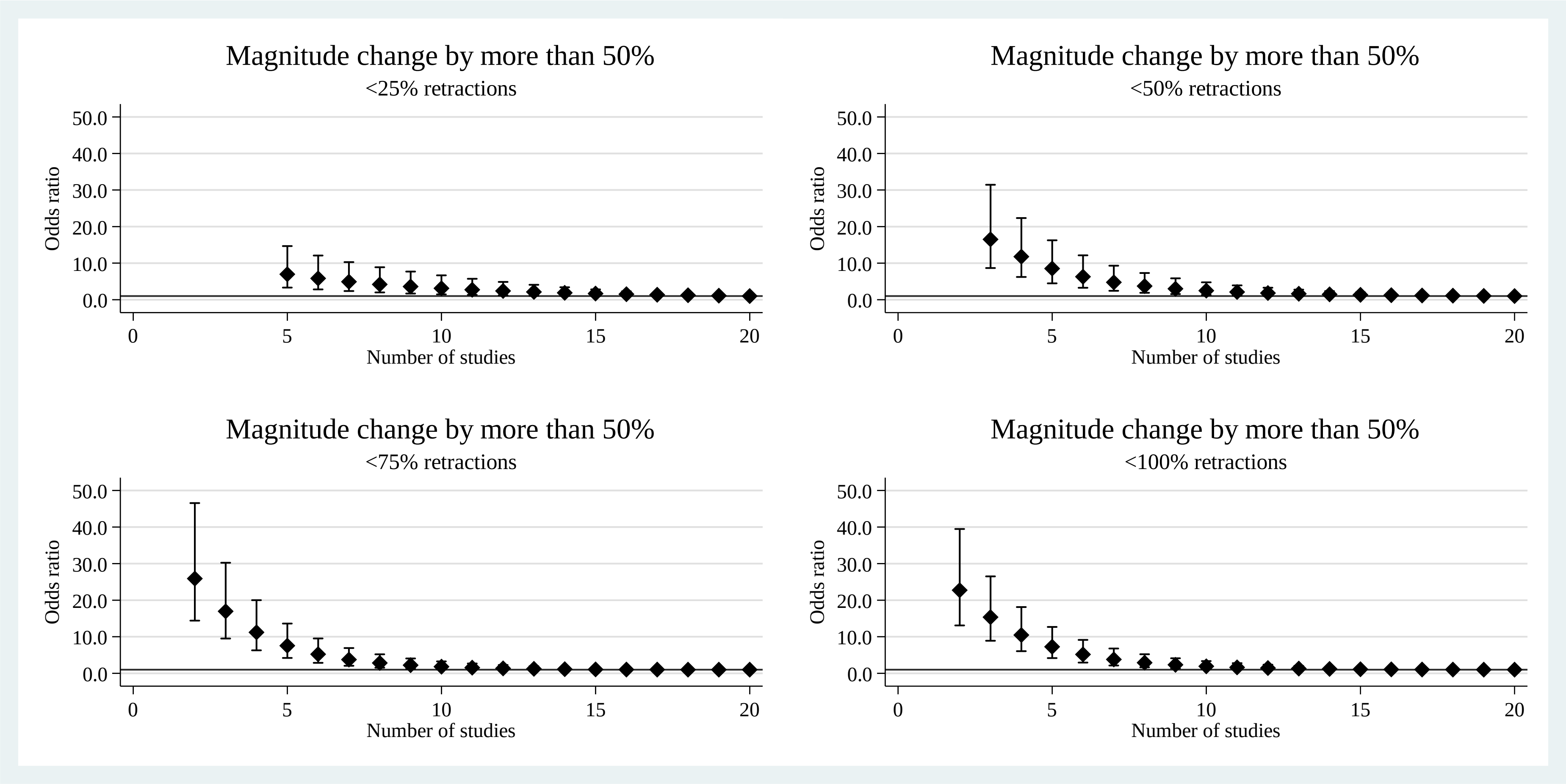

**Supplementary Figure S8.** Guideline screen for the titles/topics of 68 systematic reviews that substantially impacted.

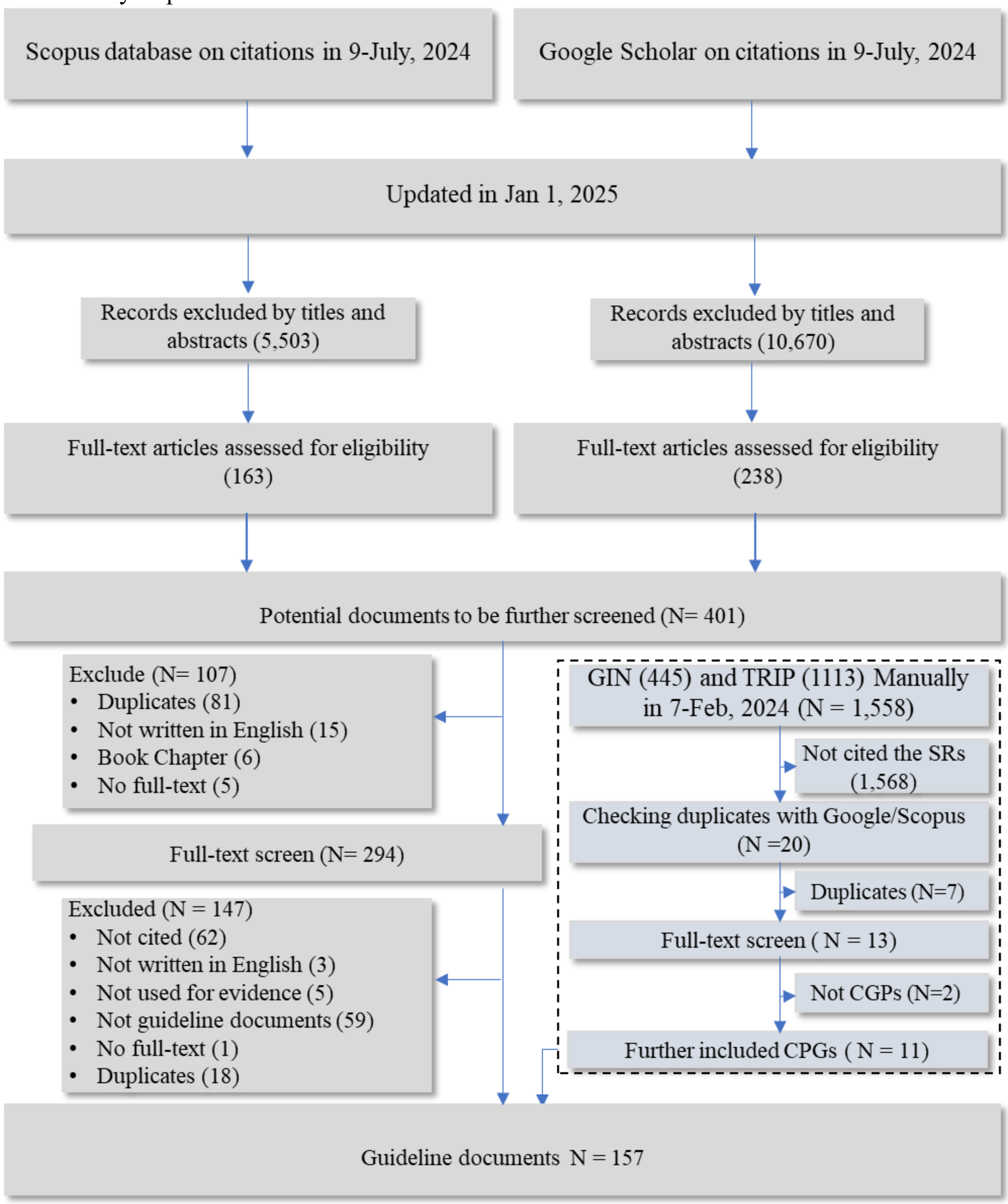

**Table S8.** Guideline documents that used evidence from the 69 systematic reviews which with the results of at least one meta-analyses substantially impacted.

| Guideline ID | Citations | Year | Organizations | Category |
|---|---|---|---|---|
| 1 | Reinhart K, Perner A, Sprung CL, Jaeschke R, Schortgen F, Johan Groeneveld AB, Beale R, Hartog CS; European Society of Intensive Care Medicine. Consensus statement of the ESICM task force on colloid volume therapy in critically ill patients. Intensive Care Med. 2012 Mar;38(3):368-83. | 2012 | European Society of Intensive Care Medicine | Consensus |
| 2 | Yu YT, Liu J, Hu B, Wang RL, Yang XH, Shang XL, Wang G, Wang CS, Li BL, Gong Y, Zhang S, Li X, Wang L, Shao M, Meng M, Zhu F, Shang Y, Xu QH, Wu ZX, Chen DC. Expert consensus on the use of human serum albumin in critically ill patients. Chin Med J (Engl). 2021 Jul 20;134(14):1639-1654. | 2021 | Chinese experts | Consensus |
| 3 | Shaulov T, Sierra S, Sylvestre C. Recurrent implantation failure in IVF: A Canadian Fertility and Andrology Society Clinical Practice Guideline. Reprod Biomed Online. 2020 Nov;41(5):819-833. | 2020 | Canadian Fertility and Andrology Society | Guideline |
| 4 | ESHRE Add-ons working group; Lundin K, Bentzen JG, Bozdag G, Ebner T, Harper J, Le Clef N, Moffett A, Norcross S, Polyzos NP, Rautakallio-Hokkanen S, Sfontouris I, Sermon K, Vermeulen N, Pinborg A. Good practice recommendations on add-ons in reproductive medicine†. Hum Reprod. 2023 Nov 2;38(11):2062-2104. | 2023 | European Society of Human Reproduction and Embryology | Practice Bulletins |
| 5 | De Luca M, Piatto G, Merola G, Himpens J, Chevallier JM, Carbajo MA, Mahawar K, Sartori A, Clemente N, Herrera M, Higa K, Brown WA, Shikora S. IFSO Update Position Statement on One Anastomosis Gastric Bypass (OAGB). Obes Surg. 2021 Jul;31(7):3251-3278. | 2021 | The International Federation for the Surgery of Obesity and Metabolic Disorders | Position Statement |
| 6 | FSRH Guideline (March 2023) Intrauterine contraception. | 2023 | Faculty of Sexual and Reproductive Healthcare (UK) | Guideline |
| 7 | Buckett W, Sierra S. The management of unexplained infertility: an evidence-based guideline from the Canadian Fertility and Andrology Society. Reprod Biomed Online. 2019 Oct;39(4):633-640. | 2019 | Canadian Fertility and Andrology Society | Guideline |
| 8 | Practice Committee of the American Society for Reproductive Medicine. Evidence-based treatments for couples with unexplained infertility: a guideline. Fertil Steril. 2020 Feb;113(2):305-322. | 2020 | American Society for Reproductive Medicine | Guideline |
| 9 | American College of Obstetricians and Gynecologists' Committee on Practice Bulletins—Gynecology. ACOG Practice Bulletin No. 200: Early Pregnancy Loss. Obstet Gynecol. 2018 Nov;132(5):e197-e207. | 2018 | American College of Obstetricians and Gynecologists | Practice Bulletins |
| 10 | American College of Obstetricians and Gynecologists' Committee on Practice Bulletins–Obstetrics. ACOG Practice Bulletin No. 197: Inherited Thrombophilias in Pregnancy. Obstet Gynecol. 2018 Jul;132(1):e18-e34. | 2018 | American College of Obstetricians and Gynecologists | Practice Bulletins |
| 11 | ESHRE Guideline Group on RPL; Bender Atik R, Christiansen OB, Elson J, Kolte AM, Lewis S, Middeldorp S, Nelen W, Peramo B, Quenby S, Vermeulen N, Goddijn M. ESHRE guideline: recurrent pregnancy loss. Hum Reprod Open. 2018 Apr 6;2018(2):hoy004. | 2018 | European Society of Human Reproduction and Embryology | Guideline |
| 12 | ACOG Committee Opinion No. 743: Low-Dose Aspirin Use During Pregnancy. Obstet Gynecol. 2018 Jul;132(1):e44-e52. | 2018 | American College of Obstetricians and Gynecologists | Opinion |
| 13 | Suker A, Li Y, Robson D, Marren A; Australasian CREI (Certificate of Reproductive Endocrinology and Infertility) Consensus Expert Panel on Trial Evidence (ACCEPT) Group. Australasian recurrent pregnancy loss clinical management guideline 2024, part II. Aust N Z J Obstet Gynaecol. 2024 Jun 27. doi: 10.1111/ajo.13820. | 2024 | Australasian Certificate of Reproductive Endocrinology and Infertility | Consensus |
| 14 | Toth B, Würfel W, Bohlmann M, Zschocke J, Rudnik-Schöneborn S, Nawroth F, Schleußner E, Rogenhofer N, Wischmann T, von Wolff M, Hancke K, von Otte S, Kuon R, Feil K, Tempfer C. Recurrent Miscarriage: Diagnostic and Therapeutic Procedures. Guideline of the DGGG, OEGGG and SGGG (S2k-Level, AWMF Registry Number 015/050). Geburtshilfe Frauenheilkd. 2018 Apr;78(4):364-381. | 2018 | German Society of Gynecology and Obstetrics & the Austrian Society of Gynecology and Obstetrics & the | Guideline |

| | | | | |
|---|---|---|---|---|
| | | | Swiss Society of Gynecology and Obstetrics | |
| 15 | Berger R, Abele H, Bahlmann F, Bedei I, Doubek K, Felderhoff-Müser U, Fluhr H, Garnier Y, Grylka-Baeschlin S, Helmer H, Herting E, Hoopmann M, Hösli I, Hoyme U, Jendreizeck A, Krentel H, Kuon R, Lütje W, Mader S, Maul H, Mendling W, Mitschdörfer B, Nicin T, Nothacker M, Olbertz D, Rath W, Roll C, Schlembach D, Schleußner E, Schütz F, Seifert-Klauss V, Steppat S, Surbek D. Prevention and Therapy of Preterm Birth. Guideline of the DGGG, OEGGG and SGGG (S2k Level, AWMF Registry Number 015/025, February 2019) - Part 2 with Recommendations on the Tertiary Prevention of Preterm Birth and the Management of Preterm Premature Rupture of Membranes. Geburtshilfe Frauenheilkd. 2019 Aug;79(8):813-833. | 2019 | German Society of Gynecology and Obstetrics & the Austrian Society of Gynecology and Obstetrics & the Swiss Society of Gynecology and Obstetrics | Guideline |
| 16 | Berger R, Abele H, Bahlmann F, Bedei I, Doubek K, Felderhoff-Müser U, Fluhr H, Garnier Y, Grylka-Baeschlin S, Helmer H, Herting E, Hoopmann M, Hösli I, Hoyme U, Jendreizeck A, Krentel H, Kuon R, Lütje W, Mader S, Maul H, Mendling W, Mitschdörfer B, Nicin T, Nothacker M, Olbertz D, Rath W, Roll C, Schlembach D, Schleußner E, Schütz F, Seifert-Klauss V, Steppat S, Surbek D. Prevention and Therapy of Preterm Birth. Guideline of the DGGG, OEGGG and SGGG (S2k Level, AWMF Registry Number 015/025, February 2019) - Part 1 with Recommendations on the Epidemiology, Etiology, Prediction, Primary and Secondary Prevention of Preterm Birth. Geburtshilfe Frauenheilkd. 2019 Aug;79(8):800-812. | 2019 | German Society of Gynecology and Obstetrics & the Austrian Society of Gynecology and Obstetrics & the Swiss Society of Gynecology and Obstetrics | Guideline |
| 17 | Jain V, McDonald SD, Mundle WR, Farine D. Guideline No. 398: Progesterone for Prevention of Spontaneous Preterm Birth. J Obstet Gynaecol Can. 2020 Jun;42(6):806-812. | 2020 | Society of Obstetricians and Gynaecologists of Canada | Guideline |
| 18 | Berger R, Abele H, Bahlmann F, Doubek K, Felderhoff-Müser U, Fluhr H, Garnier Y, Grylka-Baeschlin S, Hayward A, Helmer H, Herting E, Hoopmann M, Hösli I, Hoyme U, Kunze M, Kuon RH, Kyvernitakis I, Lütje W, Mader S, Maul H, Mendling W, Mitschdörfer B, Nothacker M, Olbertz D, Ramsell A, Rath W, Roll C, Schlembach D, Schleußner E, Schütz F, Seifert-Klauss V, Stubert J, Surbek D. Prevention and Therapy of Preterm Birth. Guideline of the DGGG, OEGGG and SGGG (S2k-Level, AWMF Registry Number 015/025, September 2022) - Part 1 with Recommendations on the Epidemiology, Etiology, Prediction, Primary and Secondary Prevention of Preterm Birth. Geburtshilfe Frauenheilkd. 2023 May 4;83(5):547-568. | 2023 | German Society of Gynecology and Obstetrics & the Austrian Society of Gynecology and Obstetrics & the Swiss Society of Gynecology and Obstetrics | Guideline |
| 19 | Berger R, Abele H, Bahlmann F, Doubek K, Felderhoff-Müser U, Fluhr H, Garnier Y, Grylka-Baeschlin S, Hayward A, Helmer H, Herting E, Hoopmann M, Hösli I, Hoyme U, Kunze M, Kuon RH, Kyvernitakis I, Lütje W, Mader S, Maul H, Mendling W, Mitschdörfer B, Nothacker M, Olbertz D, Ramsell A, Rath W, Roll C, Schlembach D, Schleußner E, Schütz F, Seifert-Klauss V, Stubert J, Surbek D. Prevention and Therapy of Preterm Birth. Guideline of the DGGG, OEGGG and SGGG (S2k Level, AWMF Registry Number 015/025, September 2022) - Part 2 with Recommendations on the Tertiary Prevention of Preterm Birth and on the Management of Preterm Premature Rupture of Membranes. Geburtshilfe Frauenheilkd. 2023 May 4;83(5):569-601. | 2023 | German Society of Gynecology and Obstetrics & the Austrian Society of Gynecology and Obstetrics & the Swiss Society of Gynecology and Obstetrics | Guideline |

| | | | | |
|---|---|---|---|---|
| 20 | Lecorguillé M, Camier A, Kadawathagedara M. Weight Changes, Nutritional Intake, Food Contaminants, and Supplements in Women of Childbearing Age, including Pregnant Women: Guidelines for Interventions during the Perinatal Period from the French National College of Midwives. J Midwifery Womens Health. 2022 Nov;67 Suppl 1:S135-S148. | 2022 | French National College of Midwives | Guideline |
| 21 | Magee LA, Smith GN, Bloch C, Côté AM, Jain V, Nerenberg K, von Dadelszen P, Helewa M, Rey E. Guideline No. 426: Hypertensive Disorders of Pregnancy: Diagnosis, Prediction, Prevention, and Management. J Obstet Gynaecol Can. 2022 May;44(5):547-571.e1. | 2022 | Society of Obstetrician and Gynaecologists of Canada | Guideline |
| 22 | Gupta P, Dabas A, Seth A, Bhatia VL, Khadgawat R, Kumar P, Balasubramanian S, Khadilkar V, Mallikarjuna HB, Godbole T, Krishnamurthy S, Goyal JP, Bhakhri BK, Ahmad A, Angadi K, Basavaraj GV, Parekh BJ, Kurpad A, Marwaha RK, Shah D, Munns C, Sachdev HPS. Indian Academy of Pediatrics Revised (2021) Guidelines on Prevention and Treatment of Vitamin D Deficiency and Rickets. Indian Pediatr. 2022 Feb 15;59(2):142-158. | 2022 | Indian Academy of Pediatrics | Guideline |
| 23 | Demay MB, Pittas AG, Bikle DD, Diab DL, Kiely ME, Lazaretti-Castro M, Lips P, Mitchell DM, Murad MH, Powers S, Rao SD, Scragg R, Tayek JA, Valent AM, Walsh JME, McCartney CR. Vitamin D for the Prevention of Disease: An Endocrine Society Clinical Practice Guideline. J Clin Endocrinol Metab. 2024 Jul 12;109(8):1907-1947. | 2024 | American Association of Clinical Endocrinology & European Society of Endocrinology & Pediatric Endocrine Society & American Society for Bone and Mineral Research & Vitamin D Workshop, American Society for Nutrition & Brazilian Society of Endocrinology and Metabolism & Society of General Internal Medicine & Endocrine Society of India | Guideline |
| 24 | Zimmer M, Sieroszewski P, Oszukowski P, Huras H, Fuchs T, Pawlosek A. Polish Society of Gynecologists and Obstetricians recommendations on supplementation during pregnancy. Ginekol Pol. 2020;91(10):644-653. | 2020 | Polish Society of Gynecologists and Obstetricians | Consensus |
| 25 | Jaisamrarn U, Esteban-Habana MA, Padolina CS, Decena DCD, Dee MT, Damodaran P, Bhaskaran V, Garg V, Dorado E, Hu H. Vitamins and minerals, education, and self-care need during preconception to 1000 days of life in Southeast Asia: An expert panel opinion. SAGE Open Med. 2023 May 19;11:20503121231173377. | 2023 | Southeast Asia experts | Opinion |
| 26 | García Martín A, Alhambra Expósito MR, Cortés Berdonces M, Jódar Gimeno E, Huguet I, Rozas Moreno P, Varsavsky M, Ávila Rubio V, Muñoz Garach A, Muñoz Torres M. Guide of management of alterations in mineral and bone metabolism during gestation and lactation. Endocrinol Diabetes Nutr (Engl Ed). 2022 Aug-Sep;69(7):530-539. | 2022 | Spanish Society of Endocrinology and Nutr | Consensus |

| | | | | |
|---|---|---|---|---|
| 27 | Nuti R, Brandi ML, Checchia G, Di Munno O, Dominguez L, Falaschi P, Fiore CE, Iolascon G, Maggi S, Michieli R, Migliaccio S, Minisola S, Rossini M, Sessa G, Tarantino U, Toselli A, Isaia GC. Guidelines for the management of osteoporosis and fragility fractures. Intern Emerg Med. 2019 Jan;14(1):85-102. | 2019 | Italian Scientific Associations | Guideline |
| 28 | Anagnostis P, Livadas S, Goulis DG, Bretz S, Ceausu I, Durmusoglu F, Erkkola R, Fistonic I, Gambacciani M, Geukes M, Hamoda H, Hartley C, Hirschberg AL, Meczekalski B, Mendoza N, Mueck A, Smetnik A, Stute P, van Trotsenburg M, Rees M, Lambrinoudaki I. EMAS position statement: Vitamin D and menopausal health. Maturitas. 2023 Mar;169:2-9. | 2023 | European Menopause and Andropause Society | Position Statement |
| 29 | Pérez-López FR, Brincat M, Erel CT, Tremollieres F, Gambacciani M, Lambrinoudaki I, Moen MH, Schenck-Gustafsson K, Vujovic S, Rozenberg S, Rees M. EMAS position statement: Vitamin D and postmenopausal health. Maturitas. 2012 Jan;71(1):83-8. | 2012 | European Menopause and Andropause Society | Position Statement |
| 30 | Blumberg JB, Cena H, Barr SI, Biesalski HK, Dagach RU, Delaney B, Frei B, Moreno González MI, Hwalla N, Lategan-Potgieter R, McNulty H, van der Pols JC, Winichagoon P, Li D. The Use of Multivitamin/Multimineral Supplements: A Modified Delphi Consensus Panel Report. Clin Ther. 2018 Apr;40(4):640-657. | 2018 | International experts in nutritional science | Consensus |
| 31 | Lambrinoudaki I, Ceasu I, Depypere H, Erel T, Rees M, Schenck-Gustafsson K, Simoncini T, Tremollieres F, van der Schouw YT, Pérez-López FR. EMAS position statement: Diet and health in midlife and beyond. Maturitas. 2013 Jan;74(1):99-104. | 2013 | European Menopause and Andropause Society | Position Statement |
| 32 | Mohanty S, Rosenthal RA, Russell MM, Neuman MD, Ko CY, Esnaola NF. Optimal Perioperative Management of the Geriatric Patient: A Best Practices Guideline from the American College of Surgeons NSQIP and the American Geriatrics Society. J Am Coll Surg. 2016 May;222(5):930-47. | 2016 | American College of Surgeons & American Geriatrics Society | Practice Bulletins |
| 33 | Klompas M, Branson R, Cawcutt K, Crist M, Eichenwald EC, Greene LR, Lee G, Maragakis LL, Powell K, Priebe GP, Speck K, Yokoe DS, Berenholtz SM. Strategies to prevent ventilator-associated pneumonia, ventilator-associated events, and nonventilator hospital-acquired pneumonia in acute-care hospitals: 2022 Update. Infect Control Hosp Epidemiol. 2022 Jun;43(6):687-713. | 2022 | Society for Healthcare Epidemiology | Guideline |
| 34 | Jin YH, Zeng XT, Liu TZ, Bai ZM, Dou ZL, Ding DG, Fan ZL, Han P, Huang YR, Huang X, Li M, Li XD, Li YN, Li XH, Liang CZ, Liu JM, Ma HS, Qi J, Shi JQ, Wang J, Wang DL, Wang ZP, Wang YY, Wang YB, Wei Q, Xia HB, Xing JC, Yan SY, Zhang XP, Zheng GY, Xing NZ, He DL, Wang XH; Chinese Urological Doctor Association (CUDA), Urological Association of Chinese Research Hospital Association (CRHA-UA), Uro-Health Promotive Association of China International Exchange, Promotive Association for Medical, Health Care (CPAM-UHPA). Treatment and surveillance for non-muscle-invasive bladder cancer: a clinical practice guideline (2021 edition). Mil Med Res. 2022 Aug 17;9(1):44. | 2022 | Chinese Urological Doctor Association & Urological Association of Chinese Research Hospital Association & Uro-Health Promotive Association of China International Exchange, Promotive Association for Medical, Health Care | Guideline |
| 35 | Neuzillet Y, Pradère B, Xylinas E, Allory Y, Audenet F, Loriot Y, Masson-Lecomte A, Roumiguié M, Seisen T, Traxer O, Leon P, Roupret M. French AFU Cancer Committee Guidelines - Update 2022-2024: Non-muscle-invasive bladder cancer (NMIBC). Prog Urol. 2022 Nov;32(15):1102-1140. | 2022 | French Association Française d'Urologie | Guideline |
| 36 | Visseren FLJ, Mach F, Smulders YM, Carballo D, Koskinas KC, Bäck M, Benetos A, Biffi A, Boavida JM, Capodanno D, Cosyns B, Crawford C, Davos CH, Desormais I, Di Angelantonio E, Franco OH, Halvorsen S, Hobbs FDR, Hollander M, Jankowska EA, Michal M, Sacco S, Sattar N, Tokgozoglu L, Tonstad S, Tsioufis KP, van Dis I, van Gelder IC, Wanner C, Williams B; ESC National Cardiac Societies; ESC Scientific Document Group. 2021 ESC Guidelines on cardiovascular disease prevention in clinical practice. Eur Heart J. 2021 Sep 7;42(34):3227-3337. | 2021 | European Society of Cardiology | Guideline |

| | | | | |
|---|---|---|---|---|
| 37 | Aboyans V, Ricco JB, Bartelink MEL, Björck M, Brodmann M, Cohnert T, Collet JP, Czerny M, De Carlo M, Debus S, Espinola-Klein C, Kahan T, Kownator S, Mazzolai L, Naylor AR, Roffi M, Röther J, Sprynger M, Tendera M, Tepe G, Venermo M, Vlachopoulos C, Desormais I; ESC Scientific Document Group. 2017 ESC Guidelines on the Diagnosis and Treatment of Peripheral Arterial Diseases, in collaboration with the European Society for Vascular Surgery (ESVS): Document covering atherosclerotic disease of extracranial carotid and vertebral, mesenteric, renal, upper and lower extremity arteriesEndorsed by: the European Stroke Organization (ESO)The Task Force for the Diagnosis and Treatment of Peripheral Arterial Diseases of the European Society of Cardiology (ESC) and of the European Society for Vascular Surgery (ESVS). Eur Heart J. 2018 Mar 1;39(9):763-816. | 2018 | European Society of Cardiology & European Society for Vascular Surgery | Guideline |
| 38 | Umemura S, Arima H, Arima S, Asayama K, Dohi Y, Hirooka Y, Horio T, Hoshide S, Ikeda S, Ishimitsu T, Ito M, Ito S, Iwashima Y, Kai H, Kamide K, Kanno Y, Kashihara N, Kawano Y, Kikuchi T, Kitamura K, Kitazono T, Kohara K, Kudo M, Kumagai H, Matsumura K, Matsuura H, Miura K, Mukoyama M, Nakamura S, Ohkubo T, Ohya Y, Okura T, Rakugi H, Saitoh S, Shibata H, Shimosawa T, Suzuki H, Takahashi S, Tamura K, Tomiyama H, Tsuchihashi T, Ueda S, Uehara Y, Urata H, Hirawa N. The Japanese Society of Hypertension Guidelines for the Management of Hypertension (JSH 2019). Hypertens Res. 2019 Sep;42(9):1235-1481. | 2019 | Japanese Society of Hypertension | Guideline |
| 39 | Lawall H, Huppert P, Espinola-Klein C, Rümenapf G. The Diagnosis and Treatment of Peripheral Arterial Vascular Disease. Dtsch Arztebl Int. 2016 Oct 28;113(43):729-736. | 2016 | German experts | Guideline |
| 40 | Lawall H, Huppert P, Espinola-Klein C, Zemmrich CS, Ruemenapf G. German guideline on the diagnosis and treatment of peripheral artery disease - a comprehensive update 2016. Vasa. 2017 Mar;46(2):79-86. | 2017 | German experts | Guideline |
| 41 | Zbigniew K, Zbigniew G, Filip S, Radosław K, Tomasz U. The position of Polish experts on conservative management in patients with artery diseases of lower limbs. Acta Angiologica 2019;25(2):41-76. | 2019 | Polish experts | Position Statement |
| 42 | Rajavi Z, Javadi MA, Daftarian N, Safi S, Nejat F, Shirvani A, Ahmadieh H, Shahraz S, Ziaei H, Moein H, Motlagh BF, Feizi S, Foroutan A, Hashemi H, Hashemian SJ, Jabbarvand M, Jafarinasab MR, Karimian F, Mohammad-Rabei H, Mohammadpour M, Nassiri N, Panahi-Bazaz M, Rohani MR, Sedaghat MR, Sheibani K. Customized Clinical Practice Guidelines for Management of Adult Cataract in Iran. J Ophthalmic Vis Res. 2015 Oct-Dec;10(4):445-60. | 2015 | Iranian Ministry of Health and Medical Education | Guideline |
| 43 | Rizzoli R, Stevenson JC, Bauer JM, van Loon LJ, Walrand S, Kanis JA, Cooper C, Brandi ML, Diez-Perez A, Reginster JY; ESCEO Task Force. The role of dietary protein and vitamin D in maintaining musculoskeletal health in postmenopausal women: a consensus statement from the European Society for Clinical and Economic Aspects of Osteoporosis and Osteoarthritis (ESCEO). Maturitas. 2014 Sep;79(1):122-32. | 2014 | European Society for Clinical and Economic Aspects of Osteoporosis and Osteoarthritis | Consensus |
| 44 | Moyer VA; U.S. Preventive Services Task Force. Prevention of falls in community-dwelling older adults: U.S. Preventive Services Task Force recommendation statement. Ann Intern Med. 2012 Aug 7;157(3):197-204. | 2012 | U.S. Preventive Services Task Force | Position Statement |
| 45 | Garber CE, Blissmer B, Deschenes MR, Franklin BA, Lamonte MJ, Lee IM, Nieman DC, Swain DP; American College of Sports Medicine. American College of Sports Medicine position stand. Quantity and quality of exercise for | 2011 | American College of Sports Medicine | Guideline |

| | | | | |
|---|---|---|---|---|
| | developing and maintaining cardiorespiratory, musculoskeletal, and neuromotor fitness in apparently healthy adults: guidance for prescribing exercise. Med Sci Sports Exerc. 2011 Jul;43(7):1334-59. | | | |
| 46 | Papaioannou A, Morin S, Cheung AM, Atkinson S, Brown JP, Feldman S, Hanley DA, Hodsman A, Jamal SA, Kaiser SM, Kvern B, Siminoski K, Leslie WD; Scientific Advisory Council of Osteoporosis Canada. 2010 clinical practice guidelines for the diagnosis and management of osteoporosis in Canada: summary. CMAJ. 2010 Nov 23;182(17):1864-73. | 2010 | Scientific Advisory Council of Osteoporosis Canada | Guideline |
| 47 | O'Donovan G, Blazevich AJ, Boreham C, Cooper AR, Crank H, Ekelund U, Fox KR, Gately P, Giles-Corti B, Gill JM, Hamer M, McDermott I, Murphy M, Mutrie N, Reilly JJ, Saxton JM, Stamatakis E. The ABC of Physical Activity for Health: a consensus statement from the British Association of Sport and Exercise Sciences. J Sports Sci. 2010 Apr;28(6):573-91. | 2010 | British Association of Sport and Exercise Sciences | Consensus |
| 48 | Elsawy B, Higgins KE. Physical activity guidelines for older adults. Am Fam Physician. 2010 Jan 1;81(1):55-9. | 2010 | American Academy of Family Physicians | Guideline |
| 49 | Kramer A, Dissemond J, Kim S, Willy C, Mayer D, Papke R, Tuchmann F, Assadian O. Consensus on Wound Antisepsis: Update 2018. Skin Pharmacol Physiol. 2018;31(1):28-58. | 2018 | International Society of Chemotherapy for Infection and Cancer & German Society for Hospital Hygiene & Chronic Wound Initiative & Austrian Society for Infection Control & Organization of all German-speaking Societies and Groups | Consensus |
| 50 | Rabe E, Partsch H, Morrison N, Meissner MH, Mosti G, Lattimer CR, Carpentier PH, Gaillard S, Jünger M, Urbanek T, Hafner J, Patel M, Wu S, Caprini J, Lurie F, Hirsch T. Risks and contraindications of medical compression treatment - A critical reappraisal. An international consensus statement. Phlebology. 2020 Aug;35(7):447-460. | 2020 | International experts | Consensus |
| 51 | Sibbald RG, Elliott JA. The role of Inadine in wound care: a consensus document. Int Wound J. 2017 Apr;14(2):316-321. doi: 10.1111/iwj.12602. | 2017 | Canada experts | Consensus |
| 52 | Wu YW, Wang CY, Cheng NC, Lin HJ, Huang HL, Huang JH, Chen CC, Lee JK, Chen PL, Hsu PC, Wu IH, Yeh JT, Tsai HY, Tzeng YS, Cheng CC, Lin CH, Wu SH, Tan JWH, Wu CH, Hsueh SK, Chang CH, Wu HP, Hsu CH, Yen HT, Lin PC, Lin CH, Tai HC, Chen WJ. 2024 TSOC/TSPS Joint Consensus: Strategies for Advanced Vascular Wound Management in Arterial and Venous Diseases. Acta Cardiol Sin. 2024 Jan;40(1):1-44. | 2024 | Taiwan Society of Cardiology & Taiwan Society of Plastic Surgery (China) | Consensus |
| 53 | Valesky EM, Hach-Wunderle V, Protz K, Zeiner KN, Erfurt-Berge C, Goedecke F, Jäger B, Kahle B, Kluess H, Knestele M, Kuntz A, Lüdemann C, Meissner M, Mühlberg K, Mühlberger D, Pannier F, Schmedt CG, Schmitz-Rixen T, Strölin A, Wilm S, Rabe E, Stücker M, Dissemond J. Diagnosis and treatment of venous leg ulcers: S2k Guideline of the German Society of Phlebology and Lymphology (DGPL) e.V. J Dtsch Dermatol Ges. 2024 Jul;22(7):1039-1051. | 2024 | German Society of Phlebology and Lymphology | Guideline |

| | | | | |
|---|---|---|---|---|
| 54 | De Maeseneer MG, Kakkos SK, Aherne T, Baekgaard N, Black S, Blomgren L, Giannoukas A, Gohel M, de Graaf R, Hamel-Desnos C, Jawien A, Jaworucka-Kaczorowska A, Lattimer CR, Mosti G, Noppeney T, van Rijn MJ, Stansby G, Esvs Guidelines Committee, Kolh P, Bastos Goncalves F, Chakfé N, Coscas R, de Borst GJ, Dias NV, Hinchliffe RJ, Koncar IB, Lindholt JS, Trimarchi S, Tulamo R, Twine CP, Vermassen F, Wanhainen A, Document Reviewers, Björck M, Labropoulos N, Lurie F, Mansilha A, Nyamekye IK, Ramirez Ortega M, Ulloa JH, Urbanek T, van Rij AM, Vuylsteke ME. Editor's Choice - European Society for Vascular Surgery (ESVS) 2022 Clinical Practice Guidelines on the Management of Chronic Venous Disease of the Lower Limbs. Eur J Vasc Endovasc Surg. 2022 Feb;63(2):184-267. | 2022 | European Society for Vascular Surgery | Guideline |
| 55 | Jindal R, Dekiwadia DB, Krishna PR, Khanna AK, Patel MD, Padaria S, Varghese R. Evidence-Based Clinical Practice Points for the Management of Venous Ulcers. Indian J Surg. 2018 Apr;80(2):171-182. | 2018 | The Association of Colon & Rectal Surgeons of India | Guideline |
| 56 | Gustafsson UO, Scott MJ, Schwenk W, Demartines N, Roulin D, Francis N, McNaught CE, Macfie J, Liberman AS, Soop M, Hill A, Kennedy RH, Lobo DN, Fearon K, Ljungqvist O; Enhanced Recovery After Surgery (ERAS) Society, for Perioperative Care; European Society for Clinical Nutrition and Metabolism (ESPEN); International Association for Surgical Metabolism and Nutrition (IASMEN). Guidelines for perioperative care in elective colonic surgery: Enhanced Recovery After Surgery (ERAS(®)) Society recommendations. World J Surg. 2013 Feb;37(2):259-84. | 2013 | Enhanced Recovery After Surgery Society | Guideline |
| 57 | Burch HB, Perros P, Bednarczuk T, Cooper DS, Dolman PJ, Leung AM, Mombaerts I, Salvi M, Stan MN. Management of thyroid eye disease: a Consensus Statement by the American Thyroid Association and the European Thyroid Association. Eur Thyroid J. 2022 Dec 8;11(6):e220189. | 2022 | American Thyroid Association & the European Thyroid Association | Consensus |
| 58 | Denlinger CS, Sanft T, Baker KS, Baxi S, et al. Survivorship, Version 2.2017, NCCN Clinical Practice Guidelines in Oncology. J Natl Compr Canc Netw. 2017 Sep;15(9):1140-1163. | 2017 | National Comprehensive Cancer Network (USA) | Guideline |
| 59 | Avasthi A, Grover S, Sathyanarayana Rao TS. Clinical Practice Guidelines for Management of Sexual Dysfunction. Indian J Psychiatry. 2017 Jan;59(Suppl 1):S91-S115. | 2017 | Indian experts | Guideline |
| 60 | Shindel AW, Althof SE, Carrier S, Chou R, McMahon CG, Mulhall JP, Paduch DA, Pastuszak AW, Rowland D, Tapscott AH, Sharlip ID. Disorders of Ejaculation: An AUA/SMSNA Guideline. J Urol. 2022 Mar;207(3):504-512. | 2022 | American Urological Association & Sexual Medicine Society of North America | Guideline |
| 61 | Melloul E, Hübner M, Scott M, Snowden C, Prentis J, Dejong CH, Garden OJ, Farges O, Kokudo N, Vauthey JN, Clavien PA, Demartines N. Guidelines for Perioperative Care for Liver Surgery: Enhanced Recovery After Surgery (ERAS) Society Recommendations. World J Surg. 2016 Oct;40(10):2425-40. | 2016 | Enhanced Recovery After Surgery Society | Guideline |
| 62 | Gan TJ, Diemunsch P, Habib AS, Kovac A, Kranke P, Meyer TA, Watcha M, Chung F, Angus S, Apfel CC, Bergese SD, Candiotti KA, Chan MT, Davis PJ, Hooper VD, Lagoo-Deenadayalan S, Myles P, Nezat G, Philip BK, Tramèr MR; Society for Ambulatory Anesthesia. Consensus guidelines for the management of postoperative nausea and vomiting. Anesth Analg. 2014 Jan;118(1):85-113. | 2014 | Society for Ambulatory Anesthesia | Consensus |

| | | | | |
|---|---|---|---|---|
| 63 | Feldheiser A, Aziz O, Baldini G, Cox BP, Fearon KC, Feldman LS, Gan TJ, Kennedy RH, Ljungqvist O, Lobo DN, Miller T, Radtke FF, Ruiz Garces T, Schricker T, Scott MJ, Thacker JK, Ytrebø LM, Carli F. Enhanced Recovery After Surgery (ERAS) for gastrointestinal surgery, part 2: consensus statement for anaesthesia practice. Acta Anaesthesiol Scand. 2016 Mar;60(3):289-334. | 2016 | Enhanced Recovery After Surgery Society | Consensus |
| 64 | Joliat GR, Kobayashi K, Hasegawa K, Thomson JE, Padbury R, Scott M, Brustia R, Scatton O, Tran Cao HS, Vauthey JN, Dincler S, Clavien PA, Wigmore SJ, Demartines N, Melloul E. Guidelines for Perioperative Care for Liver Surgery: Enhanced Recovery After Surgery (ERAS) Society Recommendations 2022. World J Surg. 2023 Jan;47(1):11-34. | 2023 | Enhanced Recovery After Surgery Society | Guideline |
| 65 | Lassen K, Coolsen MM, Slim K, Carli F, de Aguilar-Nascimento JE, Schäfer M, Parks RW, Fearon KC, Lobo DN, Demartines N, Braga M, Ljungqvist O, Dejong CH; ERAS® Society; European Society for Clinical Nutrition and Metabolism; International Association for Surgical Metabolism and Nutrition. Guidelines for perioperative care for pancreaticoduodenectomy: Enhanced Recovery After Surgery (ERAS®) Society recommendations. Clin Nutr. 2012 Dec;31(6):817-30. | 2012 | Enhanced Recovery After Surgery Society | Guideline |
| 66 | Mortensen K, Nilsson M, Slim K, Schäfer M, Mariette C, Braga M, Carli F, Demartines N, Griffin SM, Lassen K; Enhanced Recovery After Surgery (ERAS®) Group. Consensus guidelines for enhanced recovery after gastrectomy: Enhanced Recovery After Surgery (ERAS®) Society recommendations. Br J Surg. 2014 Sep;101(10):1209-29. | 2014 | Enhanced Recovery After Surgery Society | Consensus |
| 67 | Gustafsson UO, Scott MJ, Schwenk W, Demartines N, Roulin D, Francis N, McNaught CE, MacFie J, Liberman AS, Soop M, Hill A, Kennedy RH, Lobo DN, Fearon K, Ljungqvist O; Enhanced Recovery After Surgery Society. Guidelines for perioperative care in elective colonic surgery: Enhanced Recovery After Surgery (ERAS®) Society recommendations. Clin Nutr. 2012 Dec;31(6):783-800. | 2012 | Enhanced Recovery After Surgery Society | Guideline |
| 68 | Brenin DR, Dietz JR, Baima J, Cheng G, Froman J, Laronga C, Ma A, Manahan MA, Mariano ER, Rojas K, Schroen AT, Tiouririne NA, Wiechmann LS, Rao R. Pain Management in Breast Surgery: Recommendations of a Multidisciplinary Expert Panel-The American Society of Breast Surgeons. Ann Surg Oncol. 2020 Nov;27(12):4588-4602. | 2020 | American Society of Breast Surgeons | Consensus |
| 69 | Vickers A, Bali S, Baxter A, Bruce G, England J, Heafield R, Langford R, Makin R, Power I, Trim J. Consensus statement on the anticipation and prevention of acute postoperative pain: multidisciplinary RADAR approach. Curr Med Res Opin. 2009 Oct;25(10):2557-69. | 2009 | UK experts | Consensus |
| 70 | Vedantham S, Weinberg I, Desai KR, Winokur R, Kolli KP, Patel S, Nelson K, Marston W, Azene E. Society of Interventional Radiology Position Statement on the Management of Chronic Iliofemoral Venous Obstruction with Endovascular Placement of Metallic Stents. J Vasc Interv Radiol. 2023 Oct;34(10):1643-1657.e6. | 2023 | Society of Interventional Radiology | Position Statement |
| 71 | Sanchez-Rodriguez D, Bergmann P, Body JJ, Cavalier E, Gielen E, Goemaere S, Lapauw B, Laurent MR, Rozenberg S, Honvo G, Beaudart C, Bruyère O. The Belgian Bone Club 2020 guidelines for the management of osteoporosis in postmenopausal women. Maturitas. 2020 Sep;139:69-89. | 2020 | Belgian Bone Club | Guideline |
| 72 | Compston J, Cooper A, Cooper C, Gittoes N, Gregson C, Harvey N, Hope S, Kanis JA, McCloskey EV, Poole KES, Reid DM, Selby P, Thompson F, Thurston A, Vine N; National Osteoporosis Guideline Group (NOGG). UK clinical guideline for the prevention and treatment of osteoporosis. Arch Osteoporos. 2017 Dec;12(1):43. | 2017 | UK National Osteoporosis Guideline Group | Guideline |

| | | | | |
|---|---|---|---|---|
| 73 | Tarantino U, Iolascon G, Cianferotti L, Masi L, Marcucci G, Giusti F, Marini F, Parri S, Feola M, Rao C, Piccirilli E, Zanetti EB, Cittadini N, Alvaro R, Moretti A, Calafiore D, Toro G, Gimigliano F, Resmini G, Brandi ML. Clinical guidelines for the prevention and treatment of osteoporosis: summary statements and recommendations from the Italian Society for Orthopaedics and Traumatology. J Orthop Traumatol. 2017 Nov;18(Suppl 1):3-36. | 2017 | Italian Society for Orthopaedics and Traumatology | Guideline |
| 74 | Hadji P, Aapro MS, Body JJ, Gnant M, Brandi ML, Reginster JY, Zillikens MC, Glüer CC, de Villiers T, Baber R, Roodman GD, Cooper C, Langdahl B, Palacios S, Kanis J, Al-Daghri N, Nogues X, Eriksen EF, Kurth A, Rizzoli R, Coleman RE. Management of Aromatase Inhibitor-Associated Bone Loss (AIBL) in postmenopausal women with hormone sensitive breast cancer: Joint position statement of the IOF, CABS, ECTS, IEG, ESCEO IMS, and SIOG. J Bone Oncol. 2017 Mar 23;7:1-12. | 2017 | International Osteoporosis Foundation & Cancer and Bone Society. & European Calcified Tissue Society & International Expert Group & European Society for Clinical and Economics Aspects of Osteoporosis, Osteoarthritis and Musculoskeletal Disease & International Menopause Society & International Society for Geriattric Onco | Position Statement |
| 75 | Beck BR, Daly RM, Singh MA, Taaffe DR. Exercise and Sports Science Australia (ESSA) position statement on exercise prescription for the prevention and management of osteoporosis. J Sci Med Sport. 2017 May;20(5):438-445. | 2017 | Exercise and Sports Science Australia | Position Statement |
| 76 | Fletcher JA. Canadian Academy of Sport and Exercise Medicine position statement: osteoporosis and exercise. Clin J Sport Med. 2013 Sep;23(5):333-8. | 2013 | Canadian Academy of Sport and Exercise Medicine | Position Statement |
| 77 | Mendoza N, Sánchez-Borrego R, Villero J, Baró F, Calaf J, Cancelo MJ, Coronado P, Estévez A, Fernández-Moya JM, González S, Llaneza P, Neyro JL, del Pino J, Rodríguez E, Ruiz E, Cano A; Spanish Menopause Society. 2013 Up-date of the consensus statement of the Spanish Menopause Society on postmenopausal osteoporosis. Maturitas. 2013 Sep;76(1):99-107. | 2013 | Spanish Menopause Society | Consensus |
| 78 | Yeap SS, Hew FL, Lee JK, Goh EM, Chee W, Mumtaz M, Damodaran P, Lim HH, Chan SP; Malaysian Osteoporosis Society Committee Working Group for the Clinical Guidance on the Management of Osteoporosis, 2012. The Malaysian Clinical Guidance on the management of postmenopausal osteoporosis, 2012: a summary. Int J Rheum Dis. 2013 Feb;16(1):30-40. | 2013 | Malaysian Osteoporosis Society | Guideline |
| 79 | Brooke-Wavell K, Skelton DA, Barker KL, Clark EM, De Biase S, Arnold S, Paskins Z, Robinson KR, Lewis RM, Tobias JH, Ward KA, Whitney J, Leyland S. Strong, steady and straight: UK consensus statement on physical activity and exercise for osteoporosis. Br J Sports Med. 2022 May 16;56(15):837–46. | 2022 | UK experts | Consensus |
| 80 | Conley RB, Adib G, Adler RA, Åkesson KE, Alexander IM, Amenta KC, Blank RD, Brox WT, Carmody EE, Chapman-Novakofski K, Clarke BL, Cody KM, Cooper C, Crandall CJ, Dirschl DR, Eagen TJ, Elderkin AL, Fujita M, Greenspan SL, Halbout P, Hochberg MC, Javaid M, Jeray KJ, Kearns AE, King T, Koinis TF, Koontz JS, Kužma M, Lindsey C, Lorentzon M, Lyritis GP, Michaud LB, Miciano A, Morin SN, Mujahid N, Napoli N, Olenginski TP, Puzas | 2020 | American Society for Bone and Mineral Research | Consensus |

| | | | | |
|---|---|---|---|---|
| | JE, Rizou S, Rosen CJ, Saag K, Thompson E, Tosi LL, Tracer H, Khosla S, Kiel DP. Secondary Fracture Prevention: Consensus Clinical Recommendations from a Multistakeholder Coalition. J Bone Miner Res. 2020 Jan;35(1):36-52. | | | |
| 81 | Shapiro CL, Van Poznak C, Lacchetti C, Kirshner J, Eastell R, Gagel R, Smith S, Edwards BJ, Frank E, Lyman GH, Smith MR, Mhaskar R, Henderson T, Neuner J. Management of Osteoporosis in Survivors of Adult Cancers With Nonmetastatic Disease: ASCO Clinical Practice Guideline. J Clin Oncol. 2019 Nov 1;37(31):2916-2946. | 2019 | American Society of Clinical Oncology | Guideline |
| 82 | Committee on Practice Bulletins-Gynecology, The American College of Obstetricians and Gynecologists. ACOG Practice Bulletin N. 129. Osteoporosis. Obstet Gynecol. 2012 Sep;120(3):718-34. | 2012 | American College of Obstetricians and Gynecologists | Practice Bulletins |
| 83 | Hartley GW, Roach KE, Nithman RW, Betz SR, Lindsey C, Fuchs RK, Avin KG. Physical Therapist Management of Patients With Suspected or Confirmed Osteoporosis: A Clinical Practice Guideline From the Academy of Geriatric Physical Therapy. J Geriatr Phys Ther. 2022 Apr-Jun 01;44(2):E106-E119. | 2022 | Academy of Geriatric Physical Therapy | Guideline |
| 84 | Grygorieva NV, Kovalenko VM, Korzh M, et al (2023). Guideline for diagnostic, prevention and treatment of postmenopausal osteoporosis. Pain, joints, spine. 2023;13(3):128-154. | 2023 | Ukrainian experts | Guideline |
| 85 | Bardosono S, Andon H, et al. Relevance of Calcium and Vitamin D in Supporting Bone Health: An Expert Panel Recommendation in Indonesia. International Journal of Nutrition and Food Sciences. 2020; 9(2): 54-62 | 2020 | Indonesia experts | Consensus |
| 86 | Gregson CL, Armstrong DJ, Bowden J, Cooper C, Edwards J, Gittoes NJL, Harvey N, Kanis J, Leyland S, Low R, McCloskey E, Moss K, Parker J, Paskins Z, Poole K, Reid DM, Stone M, Thomson J, Vine N, Compston J. UK clinical guideline for the prevention and treatment of osteoporosis. Arch Osteoporos. 2022 Apr 5;17(1):58. | 2022 | UK National Osteoporosis Guideline Group | Guideline |
| 87 | Osteoporosis Prevention, Screening, and Diagnosis: ACOG Clinical Practice Guideline No. 1. Obstet Gynecol. 2021 Sep 1;138(3):494-506. | 2021 | American College of Obstetricians and Gynecologists | Guideline |
| 88 | Giangregorio LM, Papaioannou A, Macintyre NJ, Ashe MC, Heinonen A, Shipp K, Wark J, McGill S, Keller H, Jain R, Laprade J, Cheung AM. Too Fit To Fracture: exercise recommendations for individuals with osteoporosis or osteoporotic vertebral fracture. Osteoporos Int. 2014 Mar;25(3):821-35. | 2014 | International experts | Consensus |
| 89 | Hill QA, Grainger JD, Thachil J, Provan D, Evans G, Garg M, Bradbury C, Bagot C, Kanis JA, Compston JE; British Society of Haematology in conjunction with the UK ITP forum. The prevention of glucocorticoid-induced osteoporosis in patients with immune thrombocytopenia receiving steroids: a British Society for Haematology Good Practice Paper. Br J Haematol. 2019 May;185(3):410-417. | 2019 | British Society for Haematology | Practice Bulletins |
| 90 | Howell D, Oliver TK, Keller-Olaman S, Davidson J, Garland S, Samuels C, Savard J, Harris C, Aubin M, Olson K, Sussman J, Macfarlane J, Taylor C; Sleep Disturbance Expert Panel on behalf of the Cancer Journey Advisory Group of the Canadian Partnership Against Cancer. A Pan-Canadian practice guideline: prevention, screening, assessment, and treatment of sleep disturbances in adults with cancer. Support Care Cancer. 2013 Oct;21(10):2695-706. | 2013 | Canadian Partnership Against Cancer | Guideline |
| 91 | Zucchini S, Tumini S, Scaramuzza AE, Bonfanti R, Delvecchio M, Franceschi R, Iafusco D, Lenzi L, Mozzillo E, Passanisi S, Piona C, Rabbone I, Rapini N, Rigamonti A, Ripoli C, Salzano G, Savastio S, Schiaffini R, Zanfardino A, Cherubini V. Recommendations for recognizing, risk stratifying, treating, and managing children and adolescents with hypoglycemia. Front Endocrinol (Lausanne). 2024 Jun 4;15:1387537. | 2024 | Italian Society for Pediatric Endocrinology Diabetes | Consensus |

| | | | | |
|---|---|---|---|---|
| 92 | Yeap SS, Hew FL, Damodaran P, Chee W, Lee JK, Goh EML, Mumtaz M, Lim HH, Chan SP. A summary of the Malaysian Clinical Guidance on the management of postmenopausal and male osteoporosis, 2015. Osteoporos Sarcopenia. 2016 Mar;2(1):1-12. | 2016 | Malaysian experts | Guideline |
| 93 | Trémollieres FA, Chabbert-Buffet N, Plu-Bureau G, Rousset-Jablonski C, Lecerf JM, Duclos M, Pouilles JM, Gosset A, Boutet G, Hocke C, Maris E, Hugon-Rodin J, Maitrot-Mantelet L, Robin G, André G, Hamdaoui N, Mathelin C, Lopes P, Graesslin O, Fritel X. Management of postmenopausal women: Collège National des Gynécologues et Obstétriciens Français (CNGOF) and Groupe d'Etude sur la Ménopause et le Vieillissement (GEMVi) Clinical Practice Guidelines. Maturitas. 2022 Sep;163:62-81. | 2022 | Collège National des Gynécologues et Obstétriciens Français | Guideline |
| 94 | Lee DO, Hong YH, Cho MK, Choi YS, Chun S, Chung YJ, Hong SH, Hwang KR, Kim J, Kim H, Lee DY, Lee SR, Park HT, Seo SK, Shin JH, Song JY, Yi KW, Paik H, Lee JY. The 2024 Guidelines for Osteoporosis - Korean Society of Menopause: Part I. J Menopausal Med. 2024 Apr;30(1):1-23. | 2024 | Korean Society of Menopause | Guideline |
| 95 | Regan L, Rai R, Saravelos S, Li TC; Royal College of Obstetricians and Gynaecologists. Recurrent MiscarriageGreen-top Guideline No. 17. BJOG. 2023 Nov;130(12):e9-e39. | 2023 | Royal College of Obstetricians and Gynaecologists | Guideline |
| 96 | Toth B, Bohlmann M, Hancke K, Kuon R, Nawroth F, von Otte S, Rogenhofer N, Rudnik-Schöneborn S, Schleußner E, Tempfer C, Vomstein K, Wischmann T, von Wolff M, Würfel W, Zschocke J. Recurrent Miscarriage: Diagnostic and Therapeutic Procedures. Guideline of the DGGG, OEGGG and SGGG (S2k-Level, AWMF Registry No. 015/050, May 2022). Geburtshilfe Frauenheilkd. 2022 Nov 25;83(1):49-78. | 2022 | German Society of Gynecology and Obstetrics & the Austrian Society of Gynecology and Obstetrics & the Swiss Society of Gynecology and Obstetrics | Guideline |
| 97 | Practice Committee of the American Society for Reproductive Medicine. Role of metformin for ovulation induction in infertile patients with polycystic ovary syndrome (PCOS): a guideline. Fertil Steril. 2017 Sep;108(3):426-441. | 2017 | American Society for Reproductive Medicine | Guideline |
| 98 | Moghetti P, Carmina E, De Leo V, Lanzone A, Orio F, Pasquali R, Toscano V. How to manage the reproductive issues of PCOS: a 2015 integrated endocrinological and gynecological consensus statement of the Italian Society of Endocrinology. J Endocrinol Invest. 2015 Sep;38(9):1025-37. | 2015 | Italian Society of Endocrinology. | Consensus |
| 99 | Legro RS, Arslanian SA, Ehrmann DA, Hoeger KM, Murad MH, Pasquali R, Welt CK; Endocrine Society. Diagnosis and treatment of polycystic ovary syndrome: an Endocrine Society clinical practice guideline. J Clin Endocrinol Metab. 2013 Dec;98(12):4565-92. | 2013 | European Society of Endocrinology | Guideline |
| 100 | Teede HJ, Misso ML, Deeks AA, Moran LJ, Stuckey BG, Wong JL, Norman RJ, Costello MF; Guideline Development Groups. Assessment and management of polycystic ovary syndrome: summary of an evidence-based guideline. Med J Aust. 2011 Sep 19;195(6):S65-112. | 2011 | Australian Government Department of Health and Ageing | Guideline |
| 101 | Malik S, Verma S, Jain K, Talwar P, Dhorepatil B, Devi G, et al. Good clinical practice recommendations on management of infertility in patients from India with polycystic ovary syndrome. Fertil Sci Res. 2015;2:107-32. | 2015 | Indian Fertility Society | Practice Bulletins |
| 102 | Crandall M, Duncan T, Mallat A, Greene W, Violano P, Christmas AB, Barraco R. Prevention of fall-related injuries in the elderly: An Eastern Association for the Surgery of Trauma practice management guideline. J Trauma Acute Care Surg. 2016 Jul;81(1):196-206. | 2016 | Eastern Association for the Surgery of Trauma Injury Control and Violence Prevention | Guideline |

| | | | | |
|---|---|---|---|---|
| 103 | Evans R, Kuhnke JL, Burrows C, Kayssi A, Labreque C, O'Sullivan-Drombolis D, et al. Best practice recommendations for the prevention and management of venous leg ulcers. In: Foundations of Best Practice for Skin and Wound Management. A supplement of Wound Care Canada; 2019. 70 pp. | 2019 | Canadian Association of Wound Care | Practice Bulletins |
| 104 | Management of osteoporosis in postmenopausal women: the 2021 position statement of The North American Menopause Society. Menopause. 2021 Sep 1;28(9):973-997. | 2021 | North American Menopause Society | Position Statement |
| 105 | Osteoporosis prevention, diagnosis and management in postmenopausal women and men over 50 years of age (2nd edition). Royal Australian College of General Practitioners | 2017 | Royal Australian College of General Practitioners | Guideline |
| 106 | Scottish Intercollegiate Guidelines Network (SIGN). Management of osteoporosis and the prevention of fragility fractures. Edinburgh: SIGN; 2021. (SIGN publication no. 142). [January 2021]. Available from URL: http://www.sign.ac.uk | 2021 | Scottish Intercollegiate Guidelines Network | Guideline |
| 107 | WHO antenatal care recommendations for a positive pregnancy experience: Nutritional interventions update: Multiple micronutrient supplements during pregnancy [Internet]. Geneva: World Health Organization; 2020. | 2020 | WHO | Guideline |
| 108 | WHO recommendations on mechanical methods for induction of labour. Geneva: World Health Organization; 2022. | 2022 | WHO | Guideline |
| 109 | Ghiassi S, Nimeri A, Aleassa EM, et al. American Society for Metabolic and Bariatric Surgery position statement on one-anastomosis gastric bypass. Surg Obes Relat Dis. 2024;20(4):319-335. | 2024 | American Society for Metabolic and Bariatric Surgery | Position Statement |
| 110 | Choice of fluid in acute illness: what should be given? An international consensus. Br J Anaesth. 2014 Nov;113(5):772-83. | 2014 | ADQI XII Investigators Group | Consensus |
| 111 | Intravascular volume therapy in adults: Guidelines from the Association of the Scientific Medical Societies in Germany. Eur J Anaesthesiol. 2016 Jul;33(7):488-521. | 2016 | Association of the Scientific Medical Societies in Germany | Guideline |
| 112 | Canadian Association of Paediatric Nephrologists COVID-19 Rapid Response: Guidelines for Management of Acute Kidney Injury in Children. Canadian Journal of Kidney Health and Disease. 2021;8. | 2021 | Canadian Association of Paediatric Nephrologists | Guideline |
| 113 | 2024 EACTS/EACTAIC Guidelines on patient blood management in adult cardiac surgery in collaboration with EBCP. Eur J Cardiothorac Surg. 2024 Oct 10: ezae352. | 2024 | European Association for Cardio-Thoracic Surgery (EACTS) & European Association of Cardiothoracic Anaesthesiology and Intensive Care | Guideline |
| 114 | Consensus Guidelines for Perioperative Care for Emergency Laparotomy Enhanced Recovery After Surgery (ERAS®) Society Recommendations Part 2-Emergency Laparotomy: Intra- and Postoperative Care. World J Surg. 2023 Aug;47(8):1850-1880. | 2023 | International Enhanced Recovery After Surgery Society | Consensus |
| 115 | Prevention of acute kidney injury and protection of renal function in the intensive care unit: update 2017: Expert opinion of the Working Group on Prevention, AKI section, European Society of Intensive Care Medicine. Intensive Care Med. 2017 Jun;43(6):730-749. | 2017 | European Society of Intensive Care Medicine | Opinion |
| 116 | Acute kidney injury in the perioperative period and in intensive care units (excluding renal replacement therapies). Ann Intensive Care. 2016 Dec;6(1):48. | 2016 | Société française d'anesthésie et de réanimation & Société de réanimation de langue française | Opinion |

| | | | | |
|---|---|---|---|---|
| 117 | Recommendations for the implementation of a Patient Blood Management programme. Application to elective major orthopaedic surgery in adults. Blood Transfus. 2016 Jan;14(1):23-65. | 2016 | Italian Society of Transfusion Medicine and Immunohaematology (SIMTI); Italian Society of Italian Society of Orthopaedics and Traumatology (SIOT); Italian Society of Anaesthesia, Analgesia, Resuscitation and Intensive Therapy (S.I.A.A.R.T.I.); Italian Society for the Study of Haemostasis and Thrombosis (SISET), and the National Association of Hospital Medical Directors (ANMDO) working group | Guideline |
| 118 | KDOQI US commentary on the 2012 KDIGO clinical practice guideline for acute kidney injury. Am J Kidney Dis. 2013 May;61(5):649-72. | 2013 | Kidney Disease: Improving Global Outcomes (International Initiative) | Guideline |
| 119 | Optimization of kidney function in cardiac surgery patients with intra-abdominal hypertension: expert opinion. Perioper Med (Lond). 2024 Jul 12;13(1):72. | 2024 | Experts from USA | Opinion |
| 120 | Perioperative fluid management and outcomes in adult deceased donor liver transplantation - A systematic review of the literature and expert panel recommendations. Clin Transplant. 2022 Oct;36(10): e14651. | 2022 | ERAS4OLT Scientific Committee | Guideline |
| 121 | Scandinavian clinical practice guideline on choice of fluid in resuscitation of critically ill patients with acute circulatory failure. Acta Anaesthesiol Scand. 2015 Mar;59(3):274-85. | 2015 | Acute Circulatory Failure of the Scandinavian Society of Anaesthesiology and Intensive Care Medicine | Guideline |
| 122 | Society for Maternal-Fetal Medicine Consult Series #67: Maternal sepsis. Am J Obstet Gynecol. 2023 Sep;229(3): B2-B19. | 2023 | Society for Maternal-Fetal Medicine | Guideline |
| 123 | Risk factors and prognosis assessment for acute kidney injury: The 2020 consensus of the Taiwan AKI Task Force. J Formos Med Assoc. 2021 Jul;120(7):1424-1433. | 2021 | Taiwan AKI Task Force of China | Consensus |
| 124 | Surviving sepsis campaign international guidelines for the management of septic shock and sepsis-associated organ dysfunction in children. Intensive Care Med. 2020 Feb;46(Suppl 1):10-67. | 2020 | International experts from 12 international organization | Guideline |
| 125 | Evidence summaries and recommendations from the international evidence-based guideline for the assessment and management of polycystic ovary syndrome: assessment and treatment of infertility. Hum Reprod Open. 2019 Jan 4;2019(1): hoy021. | 2019 | European Society of Human Reproduction and Embryology | Guideline |
| 126 | International evidence-based guideline for the assessment and management of polycystic ovary syndrome 2018 | 2018 | Australasian National Health and Medical Research Council | Guideline |

| | | | | |
|---|---|---|---|---|
| 127 | NCCN Guidelines® Insights: Survivorship, Version 2.2024. J Natl Compr Canc Netw 2024;22(10):648–658 | 2024 | National Comprehensive Cancer Network | Guideline |
| 128 | European Society for Gynaecological Endoscopy (ESGE) Good Practice Recommendations on surgical techniques for removal of fibroids: part 1 abdominal (laparoscopic and open) myomectomy. Facts Views Vis Obgyn. 2024 Sep;16(3):263-280. | 2024 | European Society for Gynaecological Endoscopy | Practice Bulletins |
| 129 | Chinese Expert Consensus on the Management of Non-Blood Hemostatic Drugs During Perioperative Period. Chinese Journal of Hospital Pharmacy, 2024, 44(5): 485-494 | 2024 | The Pharmacovigilance Committee of GuangDong Pharmaceutical Association | Consensus |
| 130 | Fetal death: Expert consensus of the French College of Obstetricians and Gynecologists. Int J Gynaecol Obstet. 2024 Dec 10. | 2024 | French College of Obstetricians and Gynecologists | Consensus |
| 131 | Consensus Statement on Vitamin D Status Assessment and Supplementation: Whys, Whens, and Hows. Endocr Rev. 2024 Sep 12;45(5):625-654. | 2024 | International experts | Consensus |
| 132 | Multidisciplinary consensus document on the management of massive haemorrhage (HEMOMAS document). Med Intensiva. 2015 Nov;39(8):483-504. | 2015 | Experts from Spain | Consensus |
| 133 | European Association of Urology Guidelines on Non-muscle-invasive Bladder Cancer (TaT1 and Carcinoma In Situ)-A Summary of the 2024 Guidelines Update. Eur Urol. 2024 Dec;86(6):531-549. | 2024 | European Association of Urology | Guideline |
| 134 | Wound, Pressure Ulcer, and Burn Guidelines-5: Guidelines for the management of lower leg ulcers and varicose veins, second edition. J Dermatol. 2024 Dec 26. | 2024 | Japanese Dermatological Association | Guideline |
| 135 | Australasian Recurrent Pregnancy Loss Clinical Management Guideline 2024 Part I. Aust N Z J Obstet Gynaecol. 2024 Oct;64(5):432-444. | 2024 | Australasian Certificate of Reproductive Endocrinology and Infertility | Consensus |
| 136 | Consensus Report on Glucagon-Like Peptide-1 Receptor Agonists as Adjunctive Treatment for Individuals With Type 1 Diabetes Using an Automated Insulin Delivery System. J Diabetes Sci Technol. 2025 Jan;19(1):191-216. | 2025 | The Diabetes Technology Society | Consensus |
| 137 | Perioperative Management of Adult Patients with Diabetes Wearing Devices: A Society for Perioperative Assessment and Quality Improvement (SPAQI) Expert Consensus Statement. J Clin Anesth. 2024 Dec; 99:111627. | 2024 | Society for Perioperative Assessment and Quality Improvement | Consensus |
| 138 | Evidence-based guideline: Unexplained Infertility. 2023. ESHRE, https://www.eshre.eu/guideline/UI. | 2023 | European Society of Human Reproduction and Embryology | Guideline |
| 139 | Expert consensus on the role of supplementation in obstetrics and gynecology using modified delphi method. Arch Gynecol Obstet. 2024 Feb;309(2):639-650. | 2024 | Egyptian experts | Consensus |
| 140 | Management of recurrent implantation failure: British Fertility Society policy and practice guideline. Hum Fertil (Camb). 2022 Dec;25(5):813-837. | 2022 | British Fertility Society | Guideline |
| 141 | Use of intrauterine devices in nulliparous women. Contraception. 2017 Jun;95(6):529-537. | 2017 | Experts from UK and USA | Guideline |
| 142 | Consensus of best practice in intrauterine contraception in France. Eur J Contracept Reprod Health Care. 2019 Aug;24(4):305-313. | 2019 | Experts from France | Consensus |

| | | | | |
|---|---|---|---|---|
| 143 | Practical aspects related to the insertion of intrauterine systems. Italian Journal of Gynaecology & Obstetrics. 2016; 28: N.3 | 2016 | Experts from Italian | Consensus |
| 144 | Selected Practice Recommendations for Contraceptive Use, 2016. MMWR Recomm Rep. 2016 Jul 29;65(4):1-66. | 2016 | US Center for Disease Control and Prevention | Guideline |
| 145 | Responsible, Safe, and Effective Prescription of Opioids for Chronic Non-Cancer Pain: American Society of Interventional Pain Physicians (ASIPP) Guidelines. Pain Physician. 2017 Feb;20(2S): S3-S92. | 2017 | American Society of Interventional Pain Physicians | Guideline |
| 146 | Functional Somatic Symptoms. Dtsch Arztebl Int. 2019 Aug 9;116(33-34):553-560 | 2019 | Guideline group “Functional Somatic Symptoms" | Guideline |
| 147 | WHO guideline for non-surgical management of chronic primary low back pain in adults in primary and community care settings [Internet]. Geneva: World Health Organization; 2023. PMID: 38198579. | 2023 | WHO | Guideline |
| 148 | Noninvasive Treatments for Acute, Subacute, and Chronic Low Back Pain: A Clinical Practice Guideline From the American College of Physicians. Ann Intern Med. 2017 Apr 4;166(7):514-530. | 2017 | American College of Physicians | Guideline |
| 149 | The Chinese Association for the Study of Pain (CASP): Consensus on the Assessment and Management of Chronic Nonspecific Low Back Pain. Pain Res Manag. 2019 Aug 15;2019: 8957847. | 2019 | Chinese Association for the Study of Pain | Consensus |
| 150 | Optimizing the Management and Outcomes of Failed Back Surgery Syndrome: A Consensus Statement on Definition and Outlines for Patient Assessment. Pain Res Manag. 2019 Feb 18;2019: 3126464. | 2019 | The Chronic Back and Leg Pain Network | Consensus |
| 151 | Psychological Treatments and Psychotherapies in the Neurorehabilitation of Pain: Evidences and Recommendations from the Italian Consensus Conference on Pain in Neurorehabilitation. Front Psychol. 2016 Feb 19; 7:115. | 2016 | Italian Consensus Conference on Pain in Neurorehabilitation | Consensus |
| 152 | Non-Specific Low Back Pain. Dtsch Arztebl Int. 2017 Dec 25;114(51-52):883-890. | 2017 | National Care Guideline development group for non-specific back pain (German) | Guideline |
| 153 | VA/DoD Clinical Practice Guideline: Diagnosis and Treatment of Low Back Pain. J Gen Intern Med. 2019 Nov;34(11):2620-2629. | 2019 | Veterans Affairs (VA) and U.S. Department of Defense (DoD) Evidence-Based Practice Work Group | Guideline |
| 154 | World guidelines for falls prevention and management for older adults: a global initiative. Age Ageing. 2022 Sep 2;51(9): afac205. | 2022 | World Falls Guidelines (WFG) Task Force | Guideline |
| 155 | Practice Advisory for Preoperative and Intraoperative Pain Management of Cardiac Surgical Patients: Part 2. Anesth Analg. 2023 Jul 1;137(1):26-47. | 2023 | Society of Cardiovascular Anesthesiologists (SCA) & Quality, Safety, and Leadership (QSL) Committee's Opioid Working Group | Consensus |
| 156 | Clinical practice guide for the choice of perioperative volume-restoring fluid in adult patients undergoing non-cardiac surgery. Rev Esp Anestesiol Reanim. 2016 Jan;63(1):29-47. | 2016 | Spanish Society of Anaesthesiology and Critical Care Medicine & | Guideline |

| | | | | |
|---|---|---|---|---|
| 157 | International Evidence-based Guideline for the assessment and management of polycystic ovary syndrome 2023. | 2023 | Australasian National Health and Medical Research Council | Guideline |

**VITALITY Study I Case studies for retracted trials and the impact of the evidence ecosystem.**

**Case 1.** Zinc supplementation on metabolic status in gestational diabetes

Heidarzadeh et al. reported the results of a randomized trial of zinc supplementation in people with gestational diabetes mellitus (ref *Biol Trace Elem Res*. 2017;175(2):271-277).

The retraction notes of Nov 24, 2022 states that there were issues of overlapping datasets (with other published articles) and concerns surrounding data integrity (study sites, dates of recruitment).

Forward citation searching identified one systematic review (prior to date of retraction note) where Heidarzadeh's data had been included in a meta-analysis of change in zinc concentrations. (ref: Li et al. J Matern Fetal Neonatal Med. 2021;34(13):2140-2145).

One outcome (depicted in Figure 6 of the published review) was affected by the retraction.

The pooled SMD for zinc change with supplementation was 0.90; 95% CI=0.58 to 1.21; P<0.00001; n =4 trials. After exclusion of the retracted trial, we estimated the pooled SMD to be 0.87; 95% CI=0.45 to 1.30; P<0.00001; n =3 trials.

In this instance, trial retraction had the impact of a minor reduction in pooled effect size without any change of direction or statistical significance.
Further forward citation searching did not identify any related guideline documents that had appraised the data from this systematic review.

**Case 2.** Clomiphene citrate or aromatase inhibitors for superovulation in women with unexplained infertility

Badawy et al. reported the results of a randomized trial of clomiphene or letrozole (aromatase inhibitor) in participants with unexplained infertility who were undergoing intrauterine insemination (ref *Fertil Steril*. 2009;92(4):1355-1359).

The retraction notes of Sep 2020 states that there were issues of duplicate datasets (with other published articles) and concerns that the data could not be validated.
Forward citation searching identified three systematic reviews (prior to date of retraction note) where Badawy's data had been incorporated into twenty separate meta-analyses covering a diverse range of outcomes. (ref: Weiss NS, et al. Hum Reprod. 2017;32(5):1009-1018, Liu A, et al. J Obstet Gynaecol Res. 2014;40(5):1205-1216, Qin F, et al. Medicine (Baltimore). 2020 July;99(31): e21006).

The effect estimates in one particular meta-analysis (depicted in Figure 9 of Liu's published review) had a change in direction and statistical significance after the removal of Badawy's data.

The pooled MD for number of dominant follicles with letrozole versus clomiphene was -0.40 (95%CI: -1.68 to 0.89); n =4 trials. Following exclusion of the retracted trial, we estimated the pooled MD to be 0.20 (95%CI: 0.17 to 0.23); n =3 trials,

The original meta-analysis could be interpreted as showing no significant benefit of letrozole over clomiphene with regards to dominant follicles. In contrast, pooled effect estimate after trial retraction suggests that letrozole leads to a statistically significant increase in number of dominant follicles compared to clomiphene.

Further forward citation searching identified two guideline documents that had appraised the data from this systematic review.

1) Buckett W, Sierra S. The management of unexplained infertility: an evidence-based guideline from the Canadian Fertility and Andrology Society. Reprod Biomed Online. 2019; 39(4): 633-640.
2) Practice Committee of the American Society for Reproductive Medicine. Practice Committee of the American Society for Reproductive Medicine. Evidence-based treatments for couples with unexplained infertility: a guideline. Fertil Steril. 2020;113(2):305-322.

Our review of these two guidelines revealed that one particular recommendation by Buckett et al. may potentially merit looking at again in light of the post-retraction pooled effect estimate suggesting possible benefit in terms of increased number of dominant follicles:

**Recommendation on:** Ovarian stimulation with oral agents alone
"*Aromatase inhibitors alone do not offer any benefit in comparison to clomiphene citrate alone and should not be offered to couples with UEI (Level 1A).*"

**Case 3.** Ramipril markedly improves walking ability in patients with peripheral arterial disease: a randomized trial

Ahimastos et al. reported the results of a randomized trial of ramipril in people with peripheral vascular disease (ref *Ann Intern Med*. 2006;144(9):660-664).

The retraction notes of Dec 1, 2015 states that there were issues pertaining to admission of data fabrication in a related trial (Ahimastos 2013), and a subsequent wider investigation that was unable to validate the primary data for this specific trial.

Forward citation searching identified two systematic reviews (one prior to date of retraction note, and one with a publication date after the Ahimastos trial had already been retracted) where Ahimastos' data had been included in a meta-analysis of change in walking time. (ref: Shahin Y, Barnes R, Barakat H, Chetter IC. Meta-analysis of angiotensin converting enzyme inhibitors effect on walking ability and ankle brachial pressure index in patients with intermittent claudication. Atherosclerosis. 2013;231(2):283-290, Barrons RW, Woods JA. The Roles of ACE Inhibitors in Lower Extremity Peripheral Artery Disease. Am J Ther. 2016;23(1):e7-e15).

Both reviews had meta-analyses that incorporated data from this specific trial (Ahimastos 2006) as well as the later trial from the same author (Ahimastos 2013) that had been retracted due to admission of data fabrication.

There were a total of nine meta-analyses where both the retracted Ahimastos trials were found to have contributed outcome data. One outcome (Figure 4 in the Barrons

review) was markedly affected (with changes in direction of effect and statistical significance) following retraction of the Ahimastos data.

The pooled MD for maximum treadmill walking distance with ramipril was 126 meters (95%CI: -95 to 346); n =4 trials. After exclusion of the retracted trial, we estimated the pooled MD to be -47 meters (95% CI: -71 to -24); n =2 trials.

The original meta-analysis could be interpreted as showing that ramipril was associated with a non-statistically significant increase (benefit) in the distance that participants were able to walk. In contrast, pooled effect estimate after trial retraction suggests that ramipril led to a statistically significant reduction in the walking distance, thus raising the possibility that ramipril actually causes harm to participants.

Further forward citation searching did not identify any related guideline documents that had appraised the data from the systematic reviews.

**Stata code for the main analyses**

```
**define the effects of meta-analyses with all studies were double-zero studies after exclusion of retracted RCTS
gen rate = noofretraction/noofstudies
replace exclude_es =1 if exclude_es ==0 & exclude_lci==0 & exclude_uci ==0 & exclude_p ==0 & rate < 1
replace exclude_p =1 if exclude_es ==0 & exclude_lci==0 & exclude_uci ==0 & exclude_p ==0 & rate < 1

***change conclusions
gen dir = 0
replace dir = 1 if ((all_es > 1 & exclude_es < 1) | (all_es < 1 & exclude_es > 1)) &    (effectsize == "OR" | effectsize == "RR" | effectsize == "HR" | effectsize == "Ratio of mean" | effectsize == "IRR")
replace dir = 1 if ((all_es > 0 & exclude_es < 0) | (all_es < 0 & exclude_es > 0)) &    effectsize == "RD"
replace dir = 1 if ((all_es > 0 & exclude_es < 0) | (all_es < 0 & exclude_es > 0)) &    (effectsize == "MD" | effectsize == "WMD" | effectsize == "SMD" | effectsize == "SMD-Cochen" | effectsize == "SMD-Hedge" | effectsize == "WMD" | effectsize == "hedges" | effectsize == "NA")
replace dir = 1 if exclude_es ==0 & exclude_lci==0 & exclude_uci ==0 & exclude_p ==0

gen sig = 0
replace sig = 1 if ((all_p > 0.05 & exclude_p < 0.05) | (all_p < 0.05 & exclude_p > 0.05))
replace sig = 1 if exclude_es ==0 & exclude_lci==0 & exclude_uci ==0 & exclude_p ==0

*(change of ci also indicate conclusion change, while this situation has already been coverred in comb_all, when consider evidence from have-to-no as dir = 1 and sig =1)
*gen ci = 0
*replace ci = 1 if (all_p < 0.05 & exclude_p < 0.05 & dir == 1)

gen mag50 = 0
gen d = ( all_es - exclude_es )/all_es
replace mag50 = 1 if d > 0.5 | d < -0.5

gen comb_all =0
replace comb_all = 1 if dir ==1 & sig ==1

gen comb_either =0
replace comb_either = 1 if dir ==1 | sig ==1
```

```
gen outtype = 0
replace outtype = 1 if effectsize == "OR" | effectsize == "RR" | effectsize == "HR" | effectsize == "RD" | effectsize == "IRR"
gen harmsnum = 0
replace harmsnum = 1 if harms =="Yes"


gen reason = 0
replace reason = 1 if retractionofdataissues =="Y"

keep if notes == "Replicated"

***proportions
*****nested model*****
meglm dir ||_all:R.srid ||rcts:
meglm sig ||_all:R.srid ||rcts:
meglm mag50 ||_all:R.srid ||rcts:
meglm comb_all ||_all:R.srid ||rcts:
meglm comb_either ||_all:R.srid ||rcts:

bysort outtype: meglm dir ||_all:R.srid ||rcts:
bysort outtype: meglm sig ||_all:R.srid ||rcts:
bysort outtype: meglm mag50 ||_all:R.srid ||rcts:
bysort outtype: meglm comb_all ||_all:R.srid ||rcts:
bysort outtype: meglm comb_either ||_all:R.srid ||rcts:

bysort harmsnum: meglm dir ||_all:R.srid ||rcts:
bysort harmsnum: meglm sig ||_all:R.srid ||rcts:
bysort harmsnum: meglm mag50 ||_all:R.srid ||rcts:
bysort harmsnum: meglm comb_all ||_all:R.srid ||rcts:
bysort harmsnum: meglm comb_either || rcts: ||srid:

bysort reason: meglm dir ||_all:R.srid ||rcts:
bysort reason: meglm sig ||_all:R.srid ||rcts:
bysort reason: meglm mag50 ||_all:R.srid ||rcts:
```

```
bysort reason: meglm comb_all ||_all:R.srid ||rcts:
bysort reason: meglm comb_either ||_all:R.srid ||rcts:


*subgroup interaction
meglm dir i.outtype ||_all:R.srid ||rcts:
meglm sig i.outtype ||_all:R.srid ||rcts:
meglm mag50 i.outtype ||_all:R.srid ||rcts:
meglm comb_all i.outtype ||_all:R.srid ||rcts:
meglm comb_either i.outtype ||_all:R.srid ||rcts:

meglm dir i.harmsnum ||_all:R.srid ||rcts:
meglm sig i.harmsnum ||_all:R.srid ||rcts:
meglm mag50 i.harmsnum ||_all:R.srid ||rcts:
meglm comb_all i.harmsnum ||_all:R.srid ||rcts:
meglm comb_either i.harmsnum ||_all:R.srid ||rcts:

meglm dir i.reason ||_all:R.srid ||rcts:
meglm sig i.reason ||_all:R.srid ||rcts:
meglm mag50 i.reason ||_all:R.srid ||rcts:
meglm comb_all i.reason ||_all:R.srid ||rcts:
meglm comb_either i.reason ||_all:R.srid ||rcts:

*** extent of retracted RCTs
recode rate(min/0.249999    = 1 "<25%")(0.25/0.49999 = 2 "25-<50%") (0.5/0.749999 = 3 "50 - <75%") (.75/.9999=4 "75 - <100%") (1=.)      , gen(extent)


***RCS
gen nos = noofstudies
replace nos = 20 if noofstudies > 19
mkspline nums = nos, nk(3) cubic

***GLM relationship of number of studies and change
graph drop _all
```

```
meglm dir nums* if rate < 1 ||_all:R.srid ||rcts: , family(bin) link(logit)
qui levelsof nos if noofstudies>1
xblc nums*, covname( nos ) at(`r(levels)') ref(20) eform scatter ytitle(Odds ratio) title(Direction change) xtitle(Number of studies) yline(1) name(f1)ylabel(, grid)
test nums1 nums2

meglm sig nums* if rate < 1 ||_all:R.srid ||rcts: ,    family(bin) link(logit)
qui levelsof nos if noofstudies>1
xblc nums*, covname( nos ) at(`r(levels)') ref(20) eform scatter ytitle(Odds ratio) title(Significant change) xtitle(Number of studies) yline(1) name(f2) ylabel(, grid)
test nums1 nums2

meglm mag50 nums* if rate < 1 ||_all:R.srid ||rcts: ,    family(bin) link(logit)
qui levelsof nos if noofstudies>1
xblc nums*, covname( nos ) at(`r(levels)') ref(20) eform scatter ytitle(Odds ratio) title(Magnitude change) xtitle(Number of studies) yline(1) name(f3) ylabel(, grid)
test nums1 nums2
*graph combine f1 f2 f3, xcommon


***adjusting for proportion of retracted trials
graph drop _all
meglm dir nums* if extent<=1 & rate<1 ||_all:R.srid ||rcts: ,    family(bin) link(logit)
qui levelsof nums1 if noofstudies>4
xblc nums*, covname( nums1 ) at(`r(levels)') ref(20) eform scatter ytitle(Odds ratio) title("Direction change") subtitle("<25% retractions") xtitle(Number of studies)
yline(1) name(g1) ylabel(, grid)
meglm dir nums* if extent <=2 & rate<1 ||_all:R.srid ||rcts: ,    family(bin) link(logit)
qui levelsof nums1 if noofstudies>2
xblc nums*, covname( nums1 ) at(`r(levels)') ref(20) eform scatter ytitle(Odds ratio) title("Direction change") subtitle("<50% retractions") xtitle(Number of studies)
yline(1) name(g2) ylabel(, grid)
meglm dir nums* if extent <=3 & rate<1 ||_all:R.srid ||rcts: ,    family(bin) link(logit)
qui levelsof nums1 if noofstudies>1
xblc nums*, covname( nums1 ) at(`r(levels)') ref(20) eform scatter ytitle(Odds ratio) title("Direction change") subtitle("<75% retractions") xtitle(Number of studies)
yline(1) name(g3) ylabel(, grid)
meglm dir nums* if extent <=4 & rate<1 ||_all:R.srid ||rcts: ,    family(bin) link(logit)
qui levelsof nums1 if noofstudies>1
```

```
xblc nums*, covname( nums1 ) at(`r(levels)') ref(20) eform scatter ytitle(Odds ratio) title("Direction change") subtitle("<100% retractions") xtitle(Number of studies) yline(1) name(g4) ylabel(, grid)
graph combine g1 g2 g3 g4, ycommon xcommon

graph drop _all
meglm sig nums* if extent<=1 & rate<1 ||_all:R.srid ||rcts: , family(bin) link(logit)
qui levelsof nums1 if noofstudies>4
xblc nums*, covname( nums1 ) at(`r(levels)') ref(20) eform scatter ytitle(Odds ratio) title("Significance change") subtitle("<25% retractions") xtitle(Number of studies) yline(1) name(g5) ylabel(, grid)
meglm sig nums* if extent <=2 & rate<1 ||_all:R.srid ||rcts: , family(bin) link(logit)
qui levelsof nums1 if noofstudies>2
xblc nums*, covname( nums1 ) at(`r(levels)') ref(20) eform scatter ytitle(Odds ratio) title("Significance change") subtitle("<50% retractions") xtitle(Number of studies) yline(1) name(g6) ylabel(, grid)
meglm sig nums* if extent <=3 & rate<1 ||_all:R.srid ||rcts: , family(bin) link(logit)
qui levelsof nums1 if noofstudies>1
xblc nums*, covname( nums1 ) at(`r(levels)') ref(20) eform scatter ytitle(Odds ratio) title("Significance change") subtitle("<75% retractions") xtitle(Number of studies) yline(1) name(g7) ylabel(, grid)
meglm sig nums* if extent <=4 & rate<1 ||_all:R.srid ||rcts: ,    family(bin) link(logit)
qui levelsof nums1 if noofstudies>1
xblc nums*, covname( nums1 ) at(`r(levels)') ref(20) eform scatter ytitle(Odds ratio) title("Significance change") subtitle("<100% retractions") xtitle(Number of studies) yline(1) name(g8) ylabel(, grid)
graph combine g5 g6 g7 g8, ycommon xcommon


graph drop _all
meglm mag50 nums* if extent<=1 & rate<1 ||_all:R.srid ||rcts: , family(bin) link(logit)
qui levelsof nums1 if noofstudies>4
xblc nums*, covname( nums1 ) at(`r(levels)') ref(20) eform scatter ytitle(Odds ratio) title("Magnitude change by more than 50%") subtitle("<25% retractions") xtitle(Number of studies) yline(1) name(g9) ylabel(, grid)
meglm mag50 nums* if extent <=2 & rate<1 ||_all:R.srid ||rcts: , family(bin) link(logit)
qui levelsof nums1 if noofstudies>2
xblc nums*, covname( nums1 ) at(`r(levels)') ref(20) eform scatter ytitle(Odds ratio) title("Magnitude change by more than 50%") subtitle("<50% retractions") xtitle(Number of studies) yline(1) name(g10) ylabel(, grid)
meglm mag50 nums* if extent <=3 & rate<1 ||_all:R.srid ||rcts: , family(bin) link(logit)
```

```
qui levelsof nums1 if noofstudies>1
xblc nums*, covname( nums1 ) at(`r(levels)') ref(20) eform scatter ytitle(Odds ratio) title("Magnitude change by more than 50%") subtitle("<75% retractions")
xtitle(Number of studies) yline(1) name(g11) ylabel(, grid)
meglm mag50 nums* if extent <=4 & rate<1 ||_all:R.srid ||rcts: , family(bin) link(logit)
qui levelsof nums1 if noofstudies>1
xblc nums*, covname( nums1 ) at(`r(levels)') ref(20) eform scatter ytitle(Odds ratio) title("Magnitude change by more than 50%") subtitle("<100% retractions")
xtitle(Number of studies) yline(1) name(g12) ylabel(, grid)
graph combine g9 g10 g11 g12, ycommon xcommon


***get meta-analyse substantially impacted
gen mag = (all_es - exclude_es)/all_es
gen bigimpact = 0
replace bigimpact = 1 if comb_all == 1
keep if bigimpact == 1 & notes == "Replicated"
```
